# Genome-wide identification of MITE-derived microRNAs and their targets in bread wheat

**DOI:** 10.1186/s12864-022-08364-4

**Published:** 2022-02-22

**Authors:** Juan M. Crescente, Diego Zavallo, Mariana del Vas, Sebastián Asurmendi, Marcelo Helguera, Elmer Fernandez, Leonardo S. Vanzetti

**Affiliations:** 1 Consejo Nacional de Investigaciones Científicas y Técnicas (CONICET), Godoy Cruz 2290, Buenos Aires, CP C1425FQB Argentina; 2 Instituto de Agrobiotecnología y Biología Molecular (IABIMO), CICVyA – Instituto Nacional de Tecnología Agropecuaria (INTA), Consejo Nacional de Investigaciones Científicas y Técnicas (CONICET), Los Reseros y Nicolás Repetto, Hurlingham, CP 1686 Argentina; 3 Instituto Nacional de Tecnología Agropecuaria (INTA). EEA INTA Marcos Juárez, Ruta 12 s/n, Marcos Juarez, CP 2850 Argentina; 4 Centro de Investigación y Desarrollo en Inmunología y Enfermedades Infecciosas (CIDIE-CONICET), Universidad Católica de Córdoba, Córdoba, Argentina; 5 Facultad de Ciencias Exactas, Físicas y Naturales, Universidad Nacional de Córdoba, Córdoba, Argentina

**Keywords:** Miniature Inverted-repeat Transposable Elements, microRNAs, *Triticum aestivum*, Post-transcriptional regulation, Gene expression, 3’ UTR

## Abstract

**Background:**

Plant miRNAs are a class of small non-coding RNAs that can repress gene expression at the post-transcriptional level by targeting RNA degradation or promoting translational repression. There is increasing evidence that some miRNAs can derive from a group of non-autonomous class II transposable elements called Miniature Inverted-repeat Transposable Elements (MITEs).

**Results:**

We used public small RNA and degradome libraries from *Triticum aestivum* to screen for microRNAs production and predict their cleavage target sites. In parallel, we also created a comprehensive wheat MITE database by identifying novel elements and compiling known ones. When comparing both data sets, we found high homology between MITEs and 14% of all the miRNAs production sites detected. Furthermore, we show that MITE-derived miRNAs have preference for targeting degradation sites with MITE insertions in the 3’ UTR regions of the transcripts.

**Conclusions:**

Our results revealed that MITE-derived miRNAs can underlay the origin of some miRNAs and potentially shape a regulatory gene network. Since MITEs are found in millions of insertions in the wheat genome and are closely linked to genic regions, this kind of regulatory network could have a significant impact on the post-transcriptional control of gene expression.

**Supplementary Information:**

The online version contains supplementary material available at (10.1186/s12864-022-08364-4).

## Background

In plants, the miRNA biogenesis pathway is an intricate, well-studied mechanism, which requires Dicer or Dicer-like enzymes, Argonaute proteins as part of the RISC (RNA Induced Silencing Complex) complex, and nucleotide complementarity to exert their function [1–3]. The miRNA precursors are single-stranded transcripts that can fold into characteristic hairpin structures, which are transcribed from pol II *loci*. Usually, each miRNA gene in a specific location gives rise to only one functional RNA duplex formed by miRNA and miRNA*. Mature miRNAs (20-24 nt) can downregulate gene expression of target transcripts by either of two post-transcriptional mechanisms: mRNA cleavage or translational repression [3, 4].

Knowledge of the origin and diversification of miRNAs contributes to a better understanding of the complexity of the regulatory networks where they participate [5]. Several hypotheses have been proposed to explain the origin of miRNA genes. The more accepted ones are miRNA genes originated by inverted duplication of target genes [6] or by fortuitous foldbacks in the genomes [7]. Furthermore, there is increasing evidence that some miRNAs can derive from certain transposable elements (TEs) [2, 8].

TEs are ubiquitous components of genomes and one of the forces driving genome evolution [9]. The hexaploid (bread) wheat (*Triticum aestivum* L. 2n = 6x = 42, genome AABBDD) is one of the most important crop species. Remarkably, 85% of its 17Gb genome is derived from repetitive elements that underwent massive amplification [10]. Therefore, wheat is a particularly useful system to study the contribution of TEs in regulatory networks involving small RNAs (sRNAs) and/or miRNAs [11]. Miniature Inverted-repeat Transposable Elements (MITEs) are a small type of TEs (about 500 bp) and have an inverted repeat structure. In rice, 80% of the TE-derived miRNAs came from MITEs, while 10% are derived from retrotransposons and 9% from other DNA transposons [2].

According to a previous study, MITEs account for approximately 0.16% of the wheat genome, are distributed along the 21 chromosomes and map within gene-rich regions. Approximately 8% of the wheat genes contain MITEs within their coding or regulatory sequences [12].

In this work, using public wheat sRNAs libraries, we identified several miRNA *loci* showing high homology with MITEs. Through bioinformatics analyses and the use of public degradome libraries we established that several of them have target transcripts. Furthermore, many of the target genes have a MITE inserted, exclusively in the 3’ UTR region of the transcripts. These results allow us to propose that MITE-derived miRNA integrate a genetic regulatory network in the wheat genome. Since MITEs are found in millions across the wheat genome and are closely linked to genic regions, this kind of regulatory network could have a great impact on the post-transcriptional control of gene expression of this important crop.

## Results

### Identification and annotation of MITEs in the wheat genome

To identify and annotate MITEs in the wheat genome, we started by creating a comprehensive MITE wheat database using 569 sequences from the TREP database [13] and 6,013 sequences obtained from the wheat genome using the software MITE Tracker [12]. After clustering all sequences using VSEARCH [14], a FASTA file containing 2,002 unique MITEs was obtained, where 223 elements (11%) showed high homology with elements already described in the TREP database and 1.779 (89%) displayed high homology to new elements discovered by MITE Tracker (Additional file 1). Next, a BLAST search was performed against the wheat genome using the MITEs FASTA file as input. After filtering the results using the parameters described in Zavallo et al. for transposable element annotation [15], 1,211,340 complete MITEs sequences were finally identified and annotated. These results are available as a GFF file in Additional file 2.

### Genome wide identification of wheat miRNAs derived from sRNAs libraries

To detect valid wheat miRNAs *loci*, ShortStack [16] software was run combining 11 wheat sRNAs libraries from [17] and four wheat sRNAs libraries described in [18].

First, ShortStack identified sRNA clusters using a de-novo method; next, a *loci* file was fed to the program containing the MITE annotation file obtained in the previous step. A total of 280,891 non-duplicated sRNA clusters with at least 10 reads of coverage were identified using all 15 sRNA libraries. Of those, only 270 (0.1%) sRNA clusters met all ShortStack requirements to be classified as valid miRNAs (Table 1). Most of the valid miRNAs (65%) were 21-nt in length, followed by 22 and 24-nt (13% each), and in a lower proportion, 20 (7%) and 23-nt (3%) in length, according to the ShortStack results (Additional file 4). Additional file 3 shows the predicted secondary structure plots of all found pre-miRNAs. Regarding the position in the genome, valid miRNAs were detected in all wheat chromosomes: 195 (72%) were found in intergenic regions of the genome, 12 (5%) in promoter regions, and the remaining 63 (23%) within genes.

**Table 1:** Aggregated Shortstack outputs for all sRNA libraries and the counts of sRNA clusters for each step

Code	Description	Counts
N0	Not analyzed due to run in –nohp mode	280891
N1	No reads at all aligned in locus	280891
N2	DicerCall was invalid (<80% of reads in the Dicer size range defined by –dicermin and –dicermax)	18426
N3	Major RNA abundance was less than 2 reads	1834
N4	Major RNA length is not in the Dicer size range defined by –dicermin and –dicermax	1576
N5	Locus size is > than maximum allowed for RNA folding per option –foldsize (default is 300 nts)	22553
N6	Locus is not stranded (>20% and <80% of reads aligned to top strand)	163879
N7	RNA folding attempt failed at locus	0
N8	Strand of possible mature miRNA is opposite to that of the locus	246
N9	Retrieval of possible mature miRNA position failed	0
N10	General failure to compute miRNA-star position	0
N11	Possible mature miRNA had >5 unpaired bases in predicted precursor secondary structure	53007
N12	Possible mature miRNA was not contained in a single predicted vhairpin	4763
N13	Possible miRNA/miRNA* duplex had >2 bulges and/or >3 bulged nts v	4901
N14	Imprecise processing: Reads for possible miRNA, miRNA-star, and their 3p variants added up to less than 50% of the total reads at the locus	7355
N15	Maybe. Passed all tests EXCEPT that the miRNA-star was not sequenced. INSUFFICIENT evidence to support a de novo annotation of a new miRNA family	2081
Y	Yes. Passed all tests INCLUDING sequencing of the exact miRNA-star. Can support a de novo annotation of a new miRNA family	270

To explore how well we covered the already described miRNAs candidates, the 270 mature miRNA sequences were compared using BLAST against three well-known comprehensive wheat miRNA libraries: miRBase [19], Plant sRNA gene server [20] and PmiREN [21].

Our de-novo approach was able to cover 42 miRNAs (34.4%) of the 125 *Triticum aestivum* elements reported in miRBase, 114 (80%) of the 141 available in Plant sRNA gene server and 496 (46.6%) of the 1,063 elements described in PmiREN. These results indicate that, due to the high similarity between elements, some mature miRNA sequences found in our analysis match with more than one entry in the database we used for comparison. Additionally, based on the level of stringency we chose for comparing mature miRNAs, we found 87 novel miRNAs *locus* in our analysis.

### MITE-derived miRNAs

Regions surrounding each miRNA *loci* reported by ShortStack were compared against all MITEs sequences. Interestingly, of the 270 putative miRNAs detected, 38 (14.1%) had high homology (80% of identity in more than 80% of the sequence) with a MITE sequence (see Table 2 and Additional file 4). The alignments between each pre-miRNA and MITE are shown in Additional file 5.

**Table 2:** MITE-derived miRNAs detected in wheat genome

miRNA name	Locus	Strand	miRNA sequence	Size (nt)	MITE	mirbase	annotation	Gene
MITE_miRNA_1	1A:117385432-117385825	+	UCUGCACCCUGAAUGAUGAAUAGU	24	MITE_524		intergenic	
MITE_miRNA_2	1A:169645857-169646029	+	UAGAGAUUUCAAAUGGAACAC	21	DTT_Tdur_Thalos_103H9-1		intron	TraesCS1A02G130800
MITE_miRNA_3	1B:161992200-161992280	+	UCUGUUCACAAAUGUAAGACG	21	MITE_1125	hvu-miR6197	intron	TraesCS1B02G130500
MITE_miRNA_4	1D:147896240-147896319	+	UAUAUUUUGGUACGGAGGGAU	21	MITE_1351		intron	TraesCS1D02G131000
MITE_miRNA_5	2A:133702671-133702770	+	UGAGACGGGUAAUUUGGAACGGAG	24	DTT_Tmon_Icarus_BG607724-1		promoter	TraesCS2A02G175300
MITE_miRNA_6	2A:168984136-168984233	-	UCGGAAUUAGUUGACACUCAAA	22	MITE_926		intron	TraesCS2A02G197900
MITE_miRNA_7	2B:5671327-5671460	-	UAUCUGGACAAAUCUGAGACA	21	DTT_Hvul_Pan_M801L24-1		intron	TraesCS2B02G010300
MITE_miRNA_8	2B:84797244-84797457	-	CAUAAUCUUGAGAAUUGACCCUCC	24	MITE_1243		intergenic	
MITE_miRNA_9	2B:482720682-482720772	-	AUCUUCUAUCGUGGGACGAAG	21	DTT_Taes_Athos_BJ320318-1		intron	TraesCS2B02G337800
MITE_miRNA_10	3A:19302861-19302943	+	UCCAAUUACUCGUCGUGGUUU	21	DTT_Bdis_BdisStowawayT_	tae-miR5175-5p	intron	TraesCS3A02G033400
					consensus-1			
MITE_miRNA_11	3B:253488744-253488823	-	AUAUUAUGUGACAGAAGGAGU	21	DTT_Taes_Athos_BJ275764-1		intron	TraesCS3B02G213700
MITE_miRNA_12	3B:587564305-587564383	+	AUUGUGUACAGAGGGAGUAGU	21	MITE_33		intron	TraesCS3B02G374400
MITE_miRNA_13	3D:76935517-76935620	-	AUCCAUAUUAGUUGUCGCUGA	21	DTT_Tmon_Icarus_BG607724-1	bdi-miR5067	promoter, intron	TraesCS3D02G121000,
								TraesCS3D02G120900
MITE_miRNA_14	3D:472405006-472405092	-	ACACUUAUUUCCGAUCGGAGGG	22	MITE_361		intron	TraesCS3D02G358900
MITE_miRNA_15	3D:508204974-508205052	+	AACUGCUCCCUCCGUAAACUA	21	DTT_Taes_Athos_BJ282680-1		intergenic	
MITE_miRNA_16	4A:67171695-67171971	-	ACUUCGAGGACCUGGAUGACU	21	MITE_1191		intergenic	
MITE_miRNA_17	4B:429353827-429354091	+	UAAACAUCACAAACUUUGGCC	21	MITE_1569		exon, three_	TraesCS4B02G201300
							prime_UTR	
MITE_miRNA_18	4B:559539958-559540201	+	ACGGUCAAACUUGAAUCUCGGGAA	24	MITE_1490		intergenic	
MITE_miRNA_19	4D:18693428-18693534	-	AUUUGAGCGUCAAGUAAUUCU	21	DTT_Taes_Icarus_BQ281801-1		intron	TraesCS4D02G040200
MITE_miRNA_20	4D:142764762-142764979	+	UAGGGUGUAGAAUAAGCUAUU	21	MITE_1731		intergenic	
MITE_miRNA_21	4D:142764794-142764971	+	UAGCUUAUUCUACAUCCCAGU	21	MITE_1731		intergenic	
MITE_miRNA_22	5A:478013119-478013226	-	UCUGUGACAAGUAAUUCGAAACGG	24	DTT_Tmon_Icarus_BG607724-1	tae-miR1135	intron	TraesCS5A02G266500
MITE_miRNA_23	5A:552632192-552632284	-	UGCGGCACUUAUUUUGGGACG	21	DTT_Tdur_Hades_294D11-1	hvu-miR5049c	intergenic	
MITE_miRNA_24	5A:609867615-609867781	+	UUGUAGAGCUUUCAUUAUGGA	21	DTT_Atau_Thalos_AF338431-1		intron	TraesCS5A02G424400
MITE_miRNA_25	5B:272441139-272441265	+	AUCUGUAUGUAGUUUGUAGCGGAA	24	DTT_Hvul_Thalos_AF427791-1		intergenic	
MITE_miRNA_26	5D:6108105-6108490	-	UUUGGACAUCUGACAAGCUCU	21	MITE_1134		intergenic	
MITE_miRNA_27	5D:156814986-156815098	+	CUCCGUCCGGAAAUAUUUGUGGGA	24	DTT_Taes_Icarus_BJ263892-1		intergenic	
MITE_miRNA_28	6A:23496429-23496595	-	UUAGAGGUUUCAAUACGGACU	21	DTT_Taes_Thalos_BJ273584-1		intergenic	
MITE_miRNA_29	6B:89431495-89431654	+	CACCUAGUGGAAUCUCUAUAAAGA	24	DTT_Hvul_Thalos_BJ486760-1		intergenic	
MITE_miRNA_30	6D:273095753-273095831	+	UCGGUAACUAAAUAUGAGACU	21	MITE_1736		intergenic	
MITE_miRNA_31	6D:361524524-361524682	-	UUAGAGAUUUCAAUGUGGAUU	21	DTT_Null_Thalos_consensus-1		intron	TraesCS6D02G256100
MITE_miRNA_32	6D:461512362-461512720	-	AGAUGACCAAAUGAGCUGAAACUU	24	MITE_1134		promoter	TraesCS6D02G38090
MITE_miRNA_33	7A:516155427-516155555	-	AUGGACAAAAAGGGGUGUAUCUAG	24	DTT_Taes_Icarus_42j2-9		intergenic	
MITE_miRNA_34	7A:668530082-668530159	+	AUGACGAGUAAAUCAGAACGG	21	DTT_Tdur_Icarus_103H9-1		intergenic	
MITE_miRNA_35	7A:668530089-668530164	+	CUUCUGAUUUACUCGUCGUGG	21	DTT_Tdur_Icarus_103H9-1		intergenic	
MITE_miRNA_36	7B:34679825-34679952	+	UGUCGUAGAUUUGUCUAGAUA	21	DTT_Taes_Pan_42j2-6	hvu-miR6191	intergenic	
MITE_miRNA_37	7B:145795106-145795346	-	UCGGUAAACUAAUAUAAGAGC	21	DTT_Tdur_Athos_103H9-1		intergenic	
MITE_miRNA_38	7B:223173899-223174050	+	UGGCAAAUCUAGUUGGUGAGC	21	MITE_955		intron	TraesCS7B02G162500

**Fig. 1:**
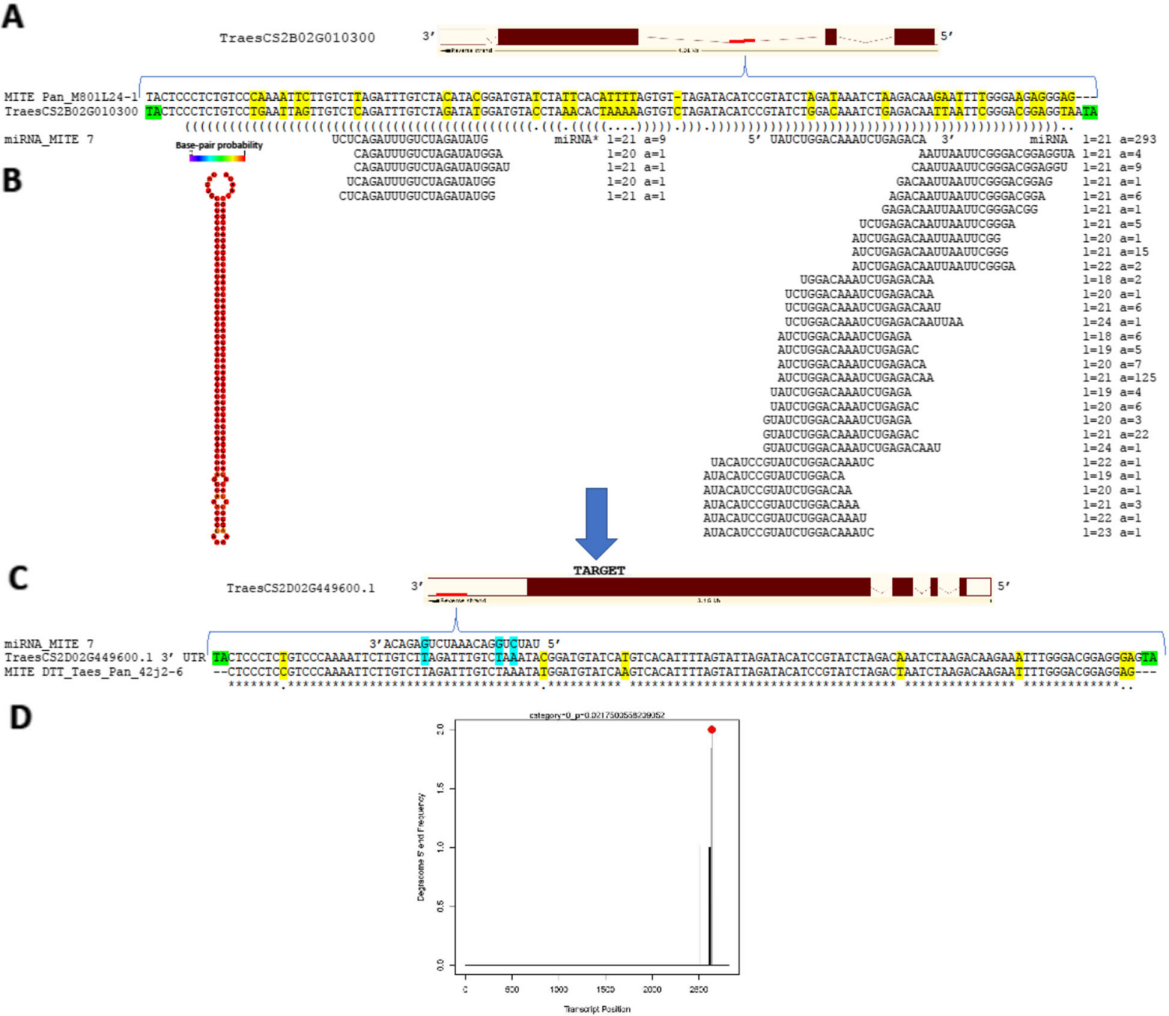
**A** Scheme of the production site of MITE-derived miRNA 7 (highlighted in red) located at the intron 2 of *TraesCS2B02G010300* gene. The DNA alignment sho ws the high sequence homology between the MITE-derived miRNA precursor, the most similar MITE identified (DTT_Hvul_ Pan_M801L24-1) and the mature MITE-derived miRNA 7. Highlighted in green is the Target Site Duplication (TSD) where the MITE was inserted and in yellow the mismatches between the intron 2 and MITE sequence. Also, mature miRNA and miRNA* as well as all small RNA reads mapped to the region as reported by Shortstack are shown below **B** Hairpin structure found in the production site of MITE-derived miRNA 7 in the intron 2 of the *TraesCS2B02G010300* gene. **C** Scheme of the *TraesCS2D02G449600.1* transcript showing a MITE-derived miRNA 7 (highlighted in red) target site at its 3’ UTR.The DNA alignment shows the high sequence homology between the target region, the most similar MITE (DTT_Taes_Pan_42j2-6) and the complementary position of the MITE-derived miRNA 7. Highlighted in green the TSD, in yellow the mismatches between the 3’ UTR, and the MITE and light blue the mismatches between the mature MITE-derived miRNA 7 and the complementary site in the 3’ UTR of *TraesCS2D02G449600.1*. **D** Signature plot of the cleavage site in the target transcript

As an example, Fig. 1A shows the high sequence homology in the alignment between the MITE-derived miRNA precursor sequence (given by ShortStack) located in the intron 2 of the gene *TraesCS2B02G010300*, the most similar MITE identified (DTT_Hvul_ Pan_M801L24-1), and the mature MITE-derived miRNA 7 (Table 2). The small RNA sequences that mapped against the region are also shown. Figure 1B shows the hairpin structure of the precursor sequence of MITE-derived miRNA 7. Finally, Fig. 1C shows the *TraesCS2D02G449600.1* transcript where a portion of the 3’ UTR corresponds to the cleavage target site of MITE-derived miRNA 7. Figure S5 in Additional file 6 shows the similarities between the predicted secondary structures of four examples of highly conserved miRNAs and four examples of MITE-derived miRNAs, which is an important feature for predicting miRNAs *loci*. Regarding their size, 28 of 38 (74%) of the MITE-derived miRNAs produce predominantly 21-nt sRNAs (Additional file 6, Fig. S1) indicating that, in wheat, MITE-derived miRNAs gave rise to 21-nt sRNAs as well as canonical miRNAs. Since these 21-nt MITE-derived sRNAs also presented hairpin structures typical of canonical miRNAs (see Additional file 3), we consider it is unlikely they were DCL3-dependent. On the other hand, ten MITE-derived miRNAs produced predominantly 24-nt sRNAs (Additional file 6, Fig. S1) (Table 2). The MITE-derived miRNAs were detected in all wheat chromosomes except for 2D and 7D. The genomic annotation indicated that 19 of those MITE-derived miRNAs were intergenic, 15 mapped inside introns, two in promoter regions and one (MITE-derived miRNA 13) in the promoter and the 3’ UTR of two different genes (Table 2).

### Identification of miRNAs and MITE-derived miRNA targets

**Fig. 2:**
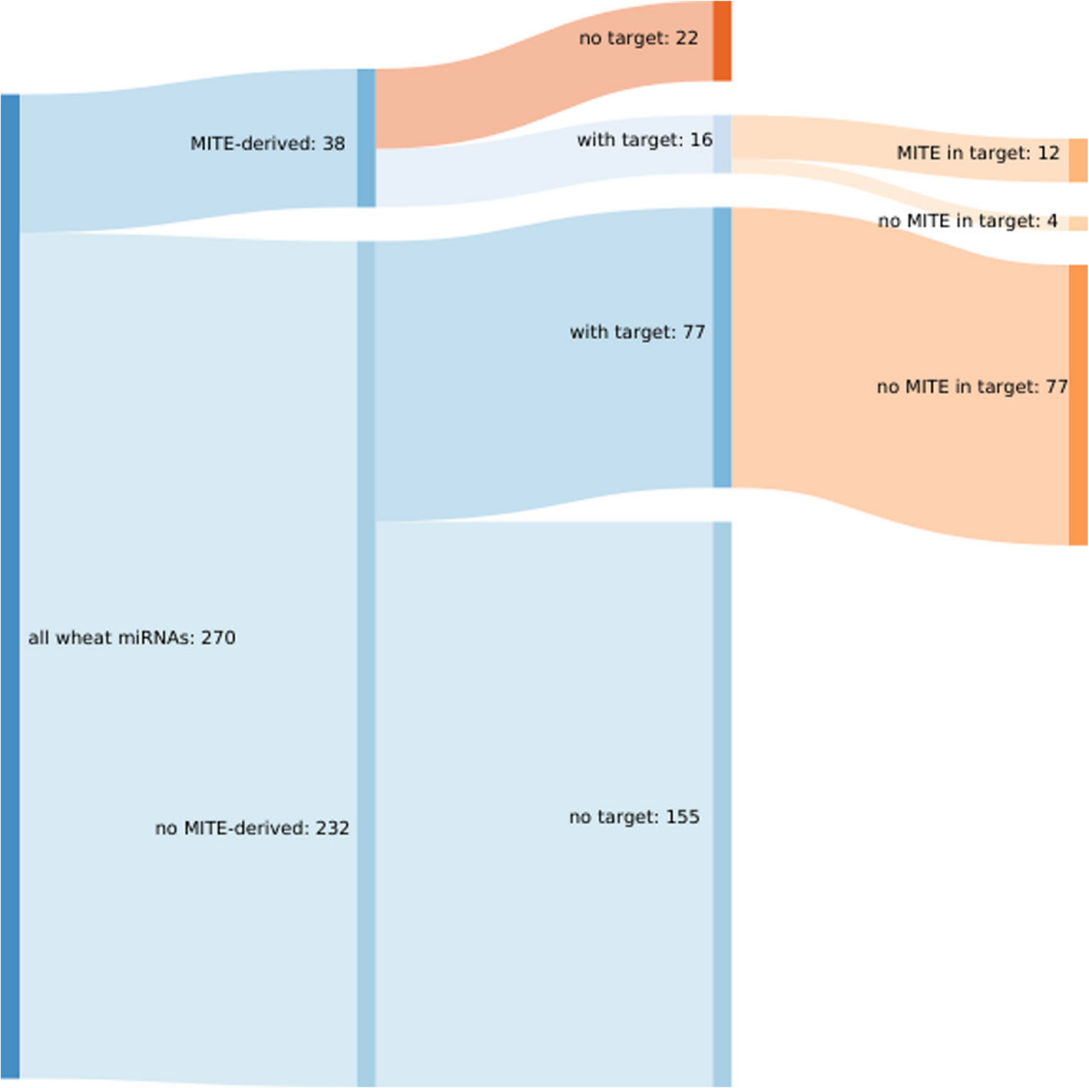
An overview of all wheat miRNAs and their targets showing how many of them are related to MITEs

To further explore the functional activity of the previously detected 270 miRNAs, target analysis was performed by sequentially using two programs that give complementary information. Cleaveland [22] is based on target degradation whereas psRNATarget [23] detects the homology between miRNA and their potential targets. Four previously described degradome libraries [17] and the 270 mature miRNAs sequences were used as input for Cleaveland, while the miRNA wheat cDNAs sequences were used in the psRNATarget pipeline. The results showed that 93 (34.4%) of the detected miRNAs had at least one confirmed target identified by both programs, producing in total 277 cleavage sites (from categories 0 to 3 and *P*-value <0.05 in Cleaveland plus positive cleavage in psRNATarget) for 227 different wheat transcripts. Additional file 7 shows all the miRNAs and their identified target transcripts. Additional file 8 shows all the cleavage site plots ordered by experiment, miRNA code name, and transcript name. Noticeably, when considering only the subgroup of the 38 MITE-derived miRNAs (Table 2), 16 (42.4%) of them had at least one target under the conditions mentioned above. These MITE-derived miRNAs targeted 36 cleavage sites in 34 different transcripts (Fig. 2; Additional file 7).

**Fig. 3:**
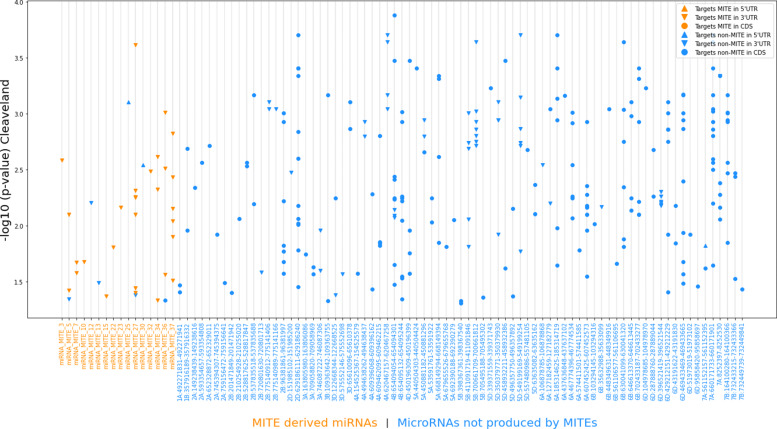
Wheat miRNAs and their targets. The X-axis has the miRNA names. Y-axis represents the *P*-value of the target sites according to Cleaveland software. MiRNA names in orange (left most of X-axis) have high homology with MITEs. Similarly, targets in orange have high homology with MITEs. Targets represented by ∘ are located in coding sequences, △ represents 5 ^′^UTR and ▽, 3 ^′^UTR

It is worth noting that miRNAs that are not derived from MITEs target both in 5’ and 3’ UTR regions as well as in coding regions (CDS), although these target regions do not show homology with MITEs (Fig. 3). Conversely, the MITE-derived miRNAs mainly had target regions with high homology with MITEs, and these regions were all located in the 3’ UTR regions of the transcripts. An example of this is shown in Fig. 1D, where the target site in the 3’ UTR of the *TraesCS1B02G479800.1* transcript and the sequence alignment between the target region, the most similar MITE (DTT_Taes_Pan_42j2-6), and the complementary position of the MITE-derived miRNA 7 promoting the cleavage of the 3’ UTR of the transcript are shown. Additional examples are listed in Table 3. However, seven MITE-derived miRNA target sites did not have high homology with MITEs at the cleavage site. In this case, four of them were located in 3’ UTRs, two in 5’ UTRs and one in a CDS (Table 3, Fig. 3).

**Table 3:** MITE-derived miRNAs and its targets transcripts with MITE insertions detected in wheat genome

miRNA	Targets		Cleaveland		psRNATarget	MITE in	MITE in
name	Trascript	Region	Category	*P*-value	Cleavage	production region	target region
miRNA_MITE_3	TraesCS5B02G218100.1	3’ UTR	0	0.003	+	MITE_1125	DTT_Hvul_Oleus_AF490468-1
miRNA_MITE_5	TraesCS2A02G281000.1	3’ UTR	1	0.008	+	DTT_Tmon_Icarus_BG607724-1	DTT_Hvul_Pan_M801L24-1
miRNA_MITE_5	TraesCS1A02G090300.1	3’ UTR	1	0.038	+	DTT_Tmon_Icarus_BG607724-1	DTT_Taes_Icarus_BQ281801-1
miRNA_MITE_7	TraesCS4B02G021400.2	3’ UTR	1	0.026	+	DTT_Hvul_Pan_M801L24-1	DTT_Taes_Pan_42j2-6
miRNA_MITE_7	TraesCS2D02G449600.1	3’ UTR	0	0.021	+	DTT_Hvul_Pan_M801L24-1	DTT_Taes_Pan_42j2-6
miRNA_MITE_10	TraesCS2D02G578900.1	3’ UTR	2	0.021	+	DTT_Bdis_BdisStowawayT_consensus-1	DTT_Bdis_BdisStowawayT_consensus-1
miRNA_MITE_15	TraesCS1B02G479800.1	3’ UTR	3	0.043	+	DTT_Taes_Athos_BJ282680-1	DTT_Taes_Athos_BJ282680-1
miRNA_MITE_22	TraesCS7A02G355700.1	3’ UTR	1	0.015	+	DTT_Tmon_Icarus_BG607724-1	DTT_Hvul_Pan_M801L24-1
miRNA_MITE_23	TraesCS3A02G333900.1	3’ UTR	0	0.007	+	DTT_Tdur_Hades_294D11-1	DTT_Taes_Hades_42j2-4
miRNA_MITE_27	TraesCS2B02G228200.1	3’ UTR	0	0.0	+	DTT_Taes_Icarus_BJ263892-1	DTT_Taes_Icarus_BJ306535-1
miRNA_MITE_27	TraesCS6B02G025200.2	3’ UTR	0	0.008	+	DTT_Taes_Icarus_BJ263892-1	DTT_Tdur_Icarus_294D11-3
miRNA_MITE_27	TraesCS6B02G069300.2	3’ UTR	1	0.006	+	DTT_Taes_Icarus_BJ263892-1	DTT_Tdur_Icarus_294D11-3
miRNA_MITE_27	TraesCS1B02G325200.1	3’ UTR	0	0.006	+	DTT_Taes_Icarus_BJ263892-1	DTT_Taes_Icarus_BJ306535-1
miRNA_MITE_27	TraesCS6B02G005300.1	3’ UTR	2	0.04	+	DTT_Taes_Icarus_BJ263892-1	DTT_Taes_Icarus_BQ605897-1
miRNA_MITE_27	TraesCS6B02G036100.1	3’ UTR	0	0.036	+	DTT_Taes_Icarus_BJ263892-1	DTT_Taes_Icarus_BE517313-1
miRNA_MITE_27	TraesCS2D02G558100.2	3’ UTR	1	0.005	+	DTT_Taes_Icarus_BJ263892-1	DTT_Hvul_Icarus_AV928891-1
miRNA_MITE_32	TraesCS3B02G129400.1	3’ UTR	1	0.003	+	MITE_1134	MITE_1134
miRNA_MITE_34	TraesCS5B02G444200.1	3’ UTR	0	0.002	+	DTT_Tdur_Icarus_103H9-1	MITE_334
miRNA_MITE_34	TraesCS4D02G124600.1	3’ UTR	0	0.005	+	DTT_Tdur_Icarus_103H9-1	MITE_394
miRNA_MITE_34	TraesCS1A02G090300.1	3’ UTR	1	0.046	+	DTT_Tdur_Icarus_103H9-1	DTT_Taes_Icarus_BQ281801-1
miRNA_MITE_36	TraesCS2A02G281000.3	3’ UTR	2	0.027	+	DTT_Taes_Pan_42j2-6	DTT_Hvul_Pan_M801L24-1
miRNA_MITE_36	TraesCS2A02G281000.2	3’ UTR	0	0.003	+	DTT_Taes_Pan_42j2-6	DTT_Hvul_Pan_M801L24-1
miRNA_MITE_36	TraesCS7B02G184300.8	3’ UTR	0	0.001	+	DTT_Taes_Pan_42j2-6	DTT_Hvul_Pan_M801L24-1
miRNA_MITE_37	TraesCS1D02G273500.1	3’ UTR	2	0.031	+	DTT_Tdur_Athos_103H9-1	DTT_Taes_Athos_42j2-5
miRNA_MITE_37	TraesCS3A02G274100.1	3’ UTR	0	0.013	+	DTT_Tdur_Athos_103H9-1	DTT_Tdur_Athos_103H9-1
miRNA_MITE_37	TraesCS6B02G168300.1	3’ UTR	0	0.007	+	DTT_Tdur_Athos_103H9-1	DTT_Taes_Athos_42j2-4
miRNA_MITE_37	TraesCS6A02G276700.1	3’ UTR	2	0.004	+	DTT_Tdur_Athos_103H9-1	DTT_Tmon_Athos_AF326781-1
miRNA_MITE_37	TraesCSU02G068000.1	3’ UTR	3	0.009	+	DTT_Tdur_Athos_103H9-1	DTT_Tdur_Athos_103H9-1
miRNA_MITE_37	TraesCS1B02G479800.1	3’ UTR	3	0.002	+	DTT_Tdur_Athos_103H9-1	DTT_Taes_Athos_BJ282680-1

Overall, our results support the existence of two fairly independent miRNA-based regulatory networks. In the first and more “conventional” one, miRNAs and their targets show no homology with MITEs. The target sites of this network can be found equally in coding regions as well as in the 5’ and 3’ UTR of the transcripts. Instead, in the second putative regulatory network, the sequences of the miRNA-producing region as well as those of the target regions of the miRNAs showed high homology with MITEs. The target sites of this network were aiming to the 3’ UTR ends of the transcripts.

Figure 3 shows all the miRNAs and their targets found in the wheat genome, grouped by unique miRNAs. Out of the 38 MITE-derived miRNAs, 16 (42%) had targets and 12 of them (75%) had also a MITE inserted in one of their targets. Noticeably, none of the non-MITE-derived miRNAs with detected targets, report a MITE insertion in their target regions.

### The MITE-derived miRNA regulatory network

For a MITE to produce a miRNA, it must be inserted into an active transcription site. Of the 38 MITE-derived miRNAs detected, 17 (45%) were found in known active transcription sites, gene promoters or introns, and the remaining 21 (55%) were located in intergenic regions, with no additional evidence of being in an active transcribed site (Table 2). We explored the idea that the incorporation of a target transcript into the MITE-derived miRNA regulatory network could depend on the evolutionary time of the MITE insertion in the target region. Taking the wheat A genome as a reference, the MITE-derived miRNA 5 (Table 2) is produced by the MITE Icarus_BG607724-1 inserted in the promoter of the gene *TraesCS2A02G175300*. The MITE-derived miRNA 5 targets the 3’ UTR of the transcript *TraesCS2A02G281000.1* where a MITE Pan_M801L24-1 is inserted (Table 3). The Pan_M801L24-1 insertion was also observed in the A genome of wheat relatives *T. urartu* (2n = 2x = 14, genome AA), *T.turgidum spp. diccocoides* (2n = 4x = 28, genome AABB) and domesticated *T. turgidum spp. durum* (2n = 4x = 28, genome AABB) but the insertion was absent in the B and D genome homeologous of *TraesCS2A02G281000* (*TraesCS2B02G298400*; *TraesCS2D02G279900* respectively), suggesting that the insertion of *TraesCS2A02G281000.1* transcript in the MITE-derived miRNA regulatory network occurred relatively early in the evolution of the A genome lineage (Additional file 6, Fig. S2). Instead, the MITE-derived miRNA 37 (Table 2), which is produced by Athos_103H9-1 in an intergenic region, targets the AthosAF326781-1 MITE inserted in the 3’ UTR of the *TraesCS6A02G276700.1* transcript. This insertion was observed in the A genome of the domesticated *T. turgidum spp. durum* but is absent in the wild relatives *T. urartu* and *T. turgidum spp. diccocoides* and also in the *TraesCS6A02G276700* homeologous in the B and D genomes (*TraesCS6B02G304200*; *TraesCS6D02G257000* respectively) (Additional file 6, Fig. S3). These results suggest that the incorporation of the *TraesCS6A02G276700.1* transcript into the MITE-derived miRNA_MITE_37 regulatory network occurred more recently in evolutionary time. Finally, another target of MITE-derived miRNA_MITE_37, is the *TraesCS3A02G274100.1* which has an Athos_103H9-1 insertion in the 3’ UTR. Such insertion was observed in *T. aestivum* A genome and not present neither in wild relatives *T. urartu* and *T. diccocoides*, nor in the domesticated *T. turgidum spp. durum*, or in the homeologous B and D genomes (*TraesCS3B02G307800*; *TraesCS3D02G273300*) (Additional file 6, Fig. S4) indicating that this transcript was incorporated into the regulatory network very recently in terms of evolutionary time.

### Expression profiles of MITE-derived miRNAs and their targets in different tissues

**Fig. 4:**
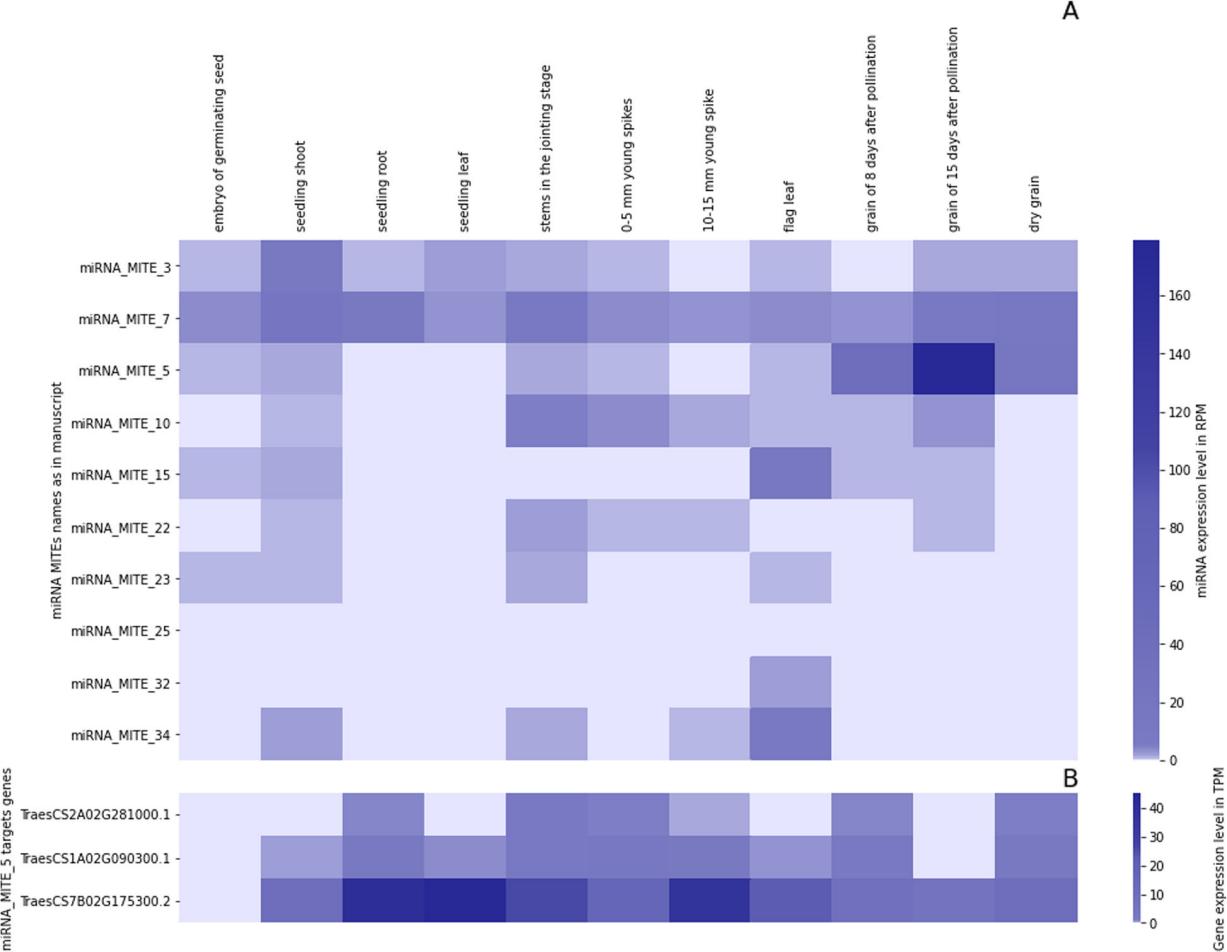
**A** Expression profiles of MITE-derived miRNA with target genes showing the insertion of a MITE in the miRNA recognition site in the 3’ UTR. The expression was evaluated in 11 different tissues, counts normalized to the total mapped reads in each library (RPM) **B** Expression profiles of target transcripts of miRNA_MITE_5 (TPM)

The expression level of MITE-derived miRNAs with validated targets in different tissues was analyzed by using Illumina-sequencing profiles from [17] (Fig. 4, Additional file 9). The miRNA_MITE_3 and miRNA_MITE_7 showed expression in all the tissues evaluated. On the other hand, miRNA_MITE_10 showed expression in all tissues except in seedling leaf. The miRNA_MITE_15 showed expression in most tissues except in seedling root, 0-5 mm young spikes and 10-15 mm young spike. Moreover, miRNA_MITE_22 showed expression in almost all tissues, except seedling leaf and grain of 8 days after pollination. miRNA_MITE_25 presented an expression similar to miRNA_MITE_22 with the exception that it is not expressed in flag leaf. The miRNA_MITE_32 is expressed in almost all tissues except 10-15 mm young spike and dry grain. On the other hand, miRNA_MITE_34 is expressed in most tissues except in embryo of germinating seed, seedling leaf and grain of 8 days after pollination. Interestingly, miRNA_MITE_5 is expressed in higher proportions in sRNAs libraries derived from grain tissues, especially in 8 and 15 days after pollination (Fig. 4A). Additionally, the miRNA_MITE_5 showed no expression in seedling root and seedling leaf tissues libraries. According to our search, the mature the miRNA_MITE_5 (UGAGACGGGUAAUUUGGAACGGAG) is not similar to any of those already described in wheat. In contrast, in a less stringent search using the miRBASE tool, we found that this miRNA is similar to tae-MIR5175 (BLAST score of 71 and an e-value of 1.5). Our findings indicates that miRNA_MITE_5 can target the 3’ UTRs of three transcripts, *TraesCS2A02G281000.1* (Dynamin-like family protein), *TraesCS7B02G175300.2* (Monodehydroasorbate reductase) and *TraesCS1A02G090300.1* (Anaphase-promoting complex subunit 7) (Additional file 7). By using public wheat expression data [24], we compared the expression level of these three target transcripts in different tissues. Remarkably, we found that two of them (*TraesCS7B02G175300.2* and *TraesCS1A02G090300.1*) are down-regulated in grain and reproductive stages (Fig. 4B). These results lead us to speculate that the high expression of the miRNA_MITE_5 during grain filling may be one of the causes of down-regulation of the two aforementioned transcripts.

## Discussion

### Characterization of the MITE-derived miRNAs in wheat

The association between miRNA and MITEs has been previously described in wheat [25, 26]. Still, to our knowledge, no genome-wide studies were conducted to decipher the connection between these two important genomics elements. Using a very stringent approach (all ShortStack miRNA discovery checkpoints passed) we identified 38 MITE-derived miRNAs throughout the entire wheat genome. However, when relaxing some parameters and taking the 2,081 sRNAs that were arrested in ShortStack N15 step (where the miRNA-star sequence was missing, Table 1) as putative miRNAs (for more detail, see Methods section), the number of miRNA derived from MITEs raised to 1,507 (72%) which would give a much more extensive and complex putative network.

Previously Yu et al. [25] found that a MITE inserted in the promoter of the spring *Vrn-A1a* allele would be responsible for the encoding of *TamiR1123*. Here, we have detected two new MITE-derived miRNAs that originated in promoter regions (Table 2) and both showed target transcripts (Table 3).

Intronic miRNAs or so-called “mirtrons” were first discovered in animals [27], and are being increasingly reported in plants. So far, mirtrons transcribed from the spliced out introns of the protein-coding genes have been reported in *Arabidopsis*, rice, cassava foxtail millet, and recently in wheat [26, 28–31]. Here, we detected 16 new MITE-derived miRNAs originated in intronic regions with evidence that four of them targets transcripts (Tables 2 and 3). These results reinforce the evidence for the presence of functional mirtrons within the wheat genome and their association with TEs. Regarding the size of the MITE-derived miRNAs, in rice Li et al. [2] detected that the miRNAs derived from TE (80% from MITEs) give rise to sRNA populations with different sizes in each *loci*. The authors suggest that the length distribution of sRNA products may reflect heterogeneity in the processing of the miRNA derived from TE and proposed that may be due to the transition of some TEs from the heterochromatic siRNAs pathway (which acts mainly via 24-nt siRNAs) to the hairpin siRNA or miRNA pathways (which act mainly via 21-nt sRNAs). Finally, the authors found that only 5.5% of the miRNAs derived from MITEs in rice were 21-nt long and 39.5% were 24-nt long. In contrast, in wheat, we found that 74% of the MITE-derived miRNAs were 21-nt long, while 26% were 24-nt long (Additional file 6, Fig. S1), suggesting that MITEs-derived miRNAs may have different origins and evolution.

### MITE-derived miRNA targets

In animals, miRNAs control the expression of target genes through binding to complementary target sites in mRNA targets [3]. In mammals, some miRNAs are similar to TEs and the presence of cognate elements in the 3’ UTR of protein-coding genes may confer susceptibility to miRNA regulation [32–36]

Otherwise, in plants, a few reports in *Arabidopsis* [37], rice [38], and more recently in wheat [26] evidenced the presence of miRNA complementary sites located at the 3’ UTR regions and not within the CDS. In this work, we present substantive evidence that MITE-derived miRNAs would target preferentially the 3’ UTRs of target genes, where a large number of MITEs are inserted (Table 3, Figs. 1C and 2).

The evolutionary separation of the diploid species *Aegilops* and *Triticum* occurred between 5.3 and 1.8 MYA, the creation of the allotetraploid species of *Triticum* between 1.8 and 0.01 MYA, and the creation and domestication of hexaploid (bread) wheat, by the incorporation of the *A. tauschii* genome and domesticated forms of tetraploid wheat occurred very recently in the last 10,000 years [39]. Several TE families underwent either proliferation or reduction during species diversification. Markers from MITEs showed polymorphic insertions (about 80%) among species and accessions of *Aegilops* and *Triticum* [40]. Interestingly, we found evidence of a regulatory network produced by MITE-derived miRNAs, on transcript targets with MITEs insertions early in evolutionary time, from *T. urartu* (5.3-1.8 MYA) to *T. aestivum* (Additional file 6, Fig. S2). We also presented evidence of the functionality of this network in more recent evolutionary periods from *T. durum* to *T. aestivum* (Additional File 6, Fig. S3) and even in very recent insertions present in *T. aestivum* (<10,000 years old) (Additional file 6, Fig. S4).

In general, our results lead us to propose that there are three crucial factors to define whether a given target transcript can be controlled by a miRNA derived from a MITE: a) the family to which the inserted MITE belongs; b) the precise place of insertion in the target gene (3’ UTR); and c) the rate of mutations of the target region. In contrast, the evolutionary time of insertion of a certain MITE into its target gene is not a crucial factor since we found old (5.3-1.8 MYA) and new (<10,000 years old) MITE elements already incorporated into the MITE-derived miRNAs regulatory network. An example of this is that MITE-derived miRNA 3 (Table 2) produced by the MITE 1125 detected by MITETracker [12], targets an element of the family Oleus (Table 3), suggesting that the variability between elements from different or the same family is important for the proper functioning of the regulatory network.

Previously Crescente et al. [12] and more recently Poretti et al. [26] proposed that genome-wide distribution of thousands of MITE copies and the frequent association of these with genes, combined with a functionally diversified regulatory network, might provide a selective advantage for frequent domestication of MITEs into miRNA genes relevant in the development and/or for adaptation to biotic stress. Here we extended these findings using a genome-wide approach to study the miRNA MITE regulatory network in wheat and unrevealed that this network is much more extensive and deep than it was foreseen in the past.

Regarding the co-expression studies of miRNAs and their target transcripts, in this work it was observed that a large part of the miRNAs derived from MITE presented expression in numerous wheat tissues and in some cases their expression was very abundant, as in the case of miRNA_MITE_5. In our study using public expression libraries we were able to establish at least a negative correlation between the expression of the miRNA and the target gene. However, deeper and more precise studies must be carried out in the future to establish accurately and reliably the expression associations between MITE-derived miRNAs and their target transcripts throughout the wheat genome.

## Concluding remarks

In this work, we identified 38 MITE-derived miRNAs out of a total of 270 miRNAs identified in the entire wheat genome. For several of these MITE-derived miRNAs, we reliably established the presence of at least one cleavage target transcript, which mostly also has a MITE inserted in the 3’ UTR region. These data revealed a regulatory network parallel to the classical miRNA network described so far. This work was carried out following stringent rules for miRNA and targets discovery. The data suggested that the scope of the MITE-derived miRNA regulatory network could be much broader and with deeper implications. Because MITEs are found in millions of copy numbers in the wheat genome and are mostly located in gene-rich regions, this regulatory network could have major impact in the wheat post-transcriptional control of gene expression.

## Methods

### MITEs database

A comprehensive wheat MITEs database was created using families retrieved with MITE Tracker [12] and from TREP database [13]. All MITEs sequences were merged into a single file and clustered using VSearch [14] (parameters –iddef 1 –id 0.8) to collapse redundant sequences. If one of the clusters obtained contained elements from TREP, a single of those elements was picked to represent each family, otherwise a random element was selected. Next, to annotate MITEs in the entire wheat genome, a genome-wide search was performed using BLAST [41]. The results were subjected to a set of filters using python scripts previously implemented [15] where elements shorter than 50 and larger than 1100 nucleotides were removed and a minimum distance between the start and end position of two different elements of five nucleotides is required to avoid duplicated hits. Also, a minimum percentage of identity between the query and the subject of 80% is required and the alignment length must be at least 80% the size of the query length. Ultimately, the filtered BLAST results file was converted to a GFF file.

### miRNAs assessment

To find putative miRNA production *loci*, we used two comprehensive sets of sRNA libraries. The first one corresponds to 11 libraries from different tissues of the hexaploid wheat cultivar Chinese Spring (*Triticum aestivum* L.) [17]. The second set is from four libraries from fully developed *T. aestivum* leaves were two of them came from plants infected with Mal de Río Cuarto virus (MRCV) and the remaining two were mock-infected controls [18]. Libraries from [17] were trimmed using Trim Galore [42] with the adapter 5’ TCGTATGCCGTCTTCTGCTTG 3’. The script used for trimming is available in the provided code repository. The two sRNA libraries and the wheat genome were used as input for ShortStack v3.8.5 software with default parameters [16, 43]. Using the alignments files from the two sets given by Shortstack, a second genome-wide identification step was done analyzing only intervals annotated with MITEs (using the –locifile parameter) using the MITE annotation obtained from the GFF file from the previous step. Secondary structure prediction plots were done using RNAfold web server [44].

### miRNAs production *loci*

Only *loci* annotated as valid miRNAs according to ShortStack criteria were kept and merged into a single file. In addition, all clusters with less than 10 reads were discarded. Duplicated elements were identified based on the miRNA sequence and the position of the sRNA cluster and removed to avoid redundancy.

To search for known mature miRNAs, a BLAST search was executed within the most common miRNA sequence of each cluster and mature miRNAs sequences from miRBase [19] and Plant sRNA gene server [20]. Results were filtered so that no more than four mismatches and gaps were allowed. Also, only alignments with a length of at least the matching mature miRNA length minus four were kept.

To annotate MITE-derived miRNAs we used ShortStack’s output which contains genomic sequences surrounding each sRNA cluster (between 60 and 900 nucleotides length). This procedure was used to find sequence homology between the production site of the miRNAs and the MITEs by using BLAST. Only results with at least 80% of identity were kept. Also a minimum alignment length of 80% of the length of the query was required.

Using the genomic annotation of [43], a GFF file containing introns and promoter’s regions (2000-nt upstream each gene) was created. Using these expanded genomic annotations, each miRNA production site was labelled according to the genomic coordinates that overlaps with it. If there was no match, the miRNA was labelled as intergenic.

### Cleavage of targets

To find putative miRNA target sites, we used two approaches that complemented each other. First, sliced targets of miRNAs from four degradome libraries from [17] were searched using the Cleaveland pipeline [22]. Only results with a *P*-value lower than 0.05 were kept. Second, transcript targets were identified using psRNATarget website with the Schema V2 (2017 release) scoring parameters [23]. The results from both approaches were merged by using the cleavage alignment positions in the transcripts reported by each program. Only those results simultaneously reported by both programs were considered. MITE insertions in target regions were identified using BLAST between the target genes sequences and the MITEs library. Only results with at least 80% identity and 80% query/alignment length were considered. Also, those hits which location included the exact miRNA cleavage genomic location were kept. Each region was labeled as 5’ UTR, 3’ UTR, or exon by using a python script that matches the position of the target sites in the transcripts and genomic annotations. Python scripts were orchestrated using several Jupyter notebooks [45]. Pandas [46] was used to read and transform miRNA production and target sites as well as genomic annotations. Sequences were read and manipulated using biopython [47]. All scripts are available in a Github repository (see Availability of data and materials).

### miRNAs expression profiles

The expression level of the miRNAs were obtained from ShortStack’s output using the sRNA libraries of [17]. These specific libraries were chosen because they represent expression levels of miRNAs in different age and high level tissues. The read counts were normalized in RPM using a python script available in the provided repository inside a Jupyter notebook. A heatmap plot was created using the Seaborn library [48]. The expression level of the target transcripts was obtained from the website wheat-expression.com [24].

## Supplementary Information

**Additional file 1** FASTA file containing 2002 unique MITEs used in this study.

**Additional file 2** GFF file containing 1.211.340 MITEs locations in the IWGSC wheat genome.

**Additional file 3** pre-miRNAs secondary structures predictions.

**Additional file 4** CSV file containing detailed information about the 270 valid miRNAs detected through ShortStack. Contains additional information about homology with MITEs and genomic annotations.

**Additional file 5** MITEs and pre-miRNAs sequences and alignments between them.

**Additional file 6** **Figure S1**: Length distribution of the sRNAs produced by the 38 MITE-derived miRNAs. **Figure S2**: Description of the MITE-derived miRNA 5 production site and an alignment of the target transcript *TraesCS2A02G281000.2* region considering the wheat relatives for the A genome. **Figure S3**: Description of the MITE-derived miRNA 36 production site and an alignment of the target transcript *TraesCS6A02G276700.1* region considering the wheat relatives for the A genome. **Figure S4**: Description of the MITE-derived miRNA 36 production site and an alignment of the target transcript *TraesCS3A02G274100.1* region considering the wheat relatives for the A genome. **Figure S5**: Hairpin plots of the secondary structures of 4 examples of highly conserved miRNAs and 4 examples of MITE-derived miRNAs.

**Additional file 7** CSV file containing detailed information about all targets detected by Cleaveland and psRNATarget. Contains additional information about homology with MITEs and genomic annotations.

**Additional file 8** Degradome plots of all miRNA and targets discovered.

**Additional file 9** MITE-derived miRNA expression profiles in different ages and high level tissues.

## Data Availability

All scripts, commands and software used are detailed in https://github.com/juancrescente/mirna_mite. Relevant data is provided as additional files.

## Abbreviations

GFF: Generic file format
miRNA: microRNA
MITE-derived miRNA: microRNA produced by a MITE-related region
MITE: Miniature Inverted-repeat Transposable Element
MYA: Million Years Ago
sRNA: Small RNA
TE: Transposable Element
UTR: untranslated region
