# Supplementary material for: Genome-wide identification of MITE-derived microRNAs and their targets in bread wheat
Source: BMC Genomics. 2022 Feb 22;23:154. doi: 10.1186/s12864-022-08364-4 (PMC8862332; doi:10.1186/s12864-022-08364-4)

MITE_miRNA_1

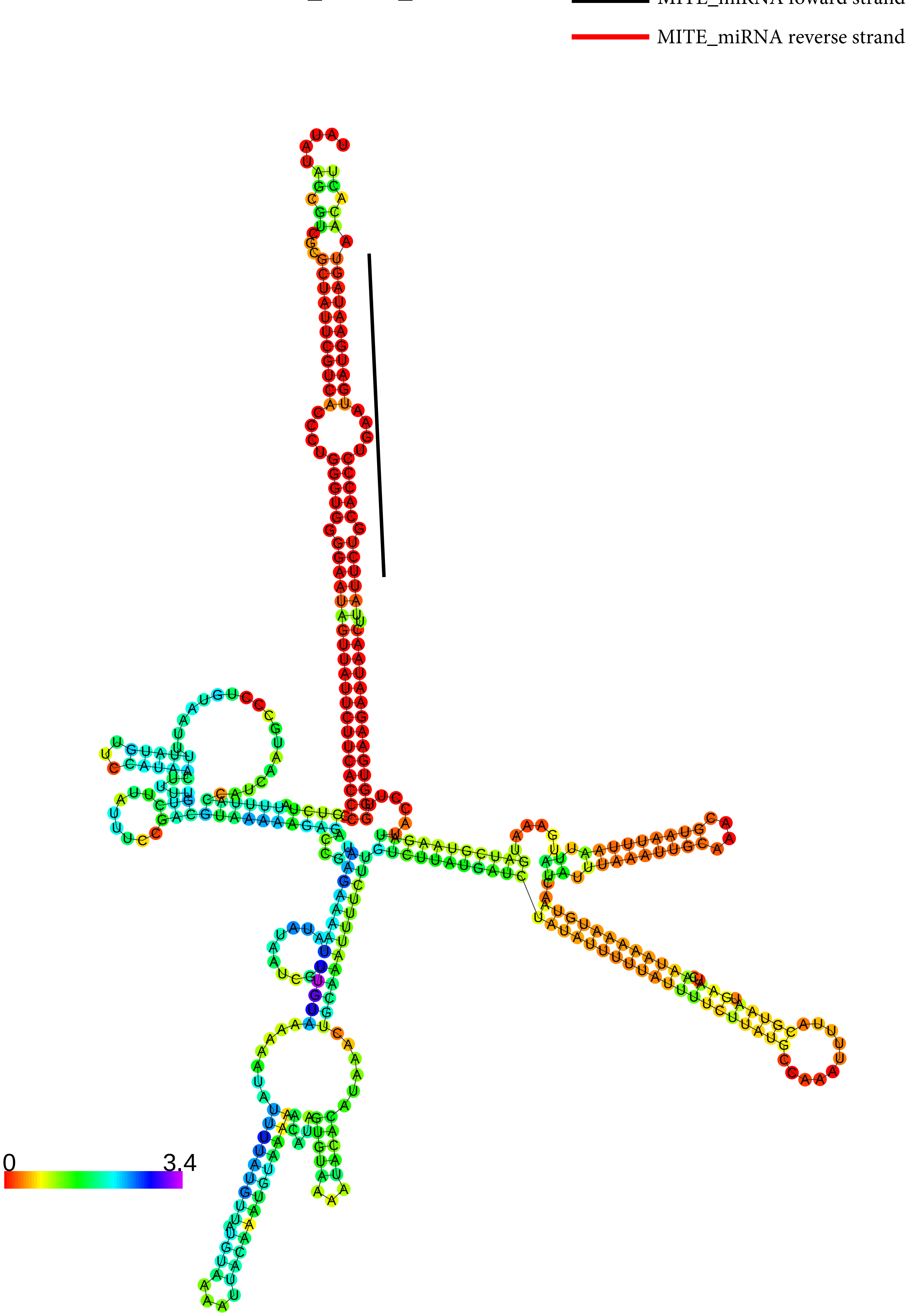

MITE_miRNA_2

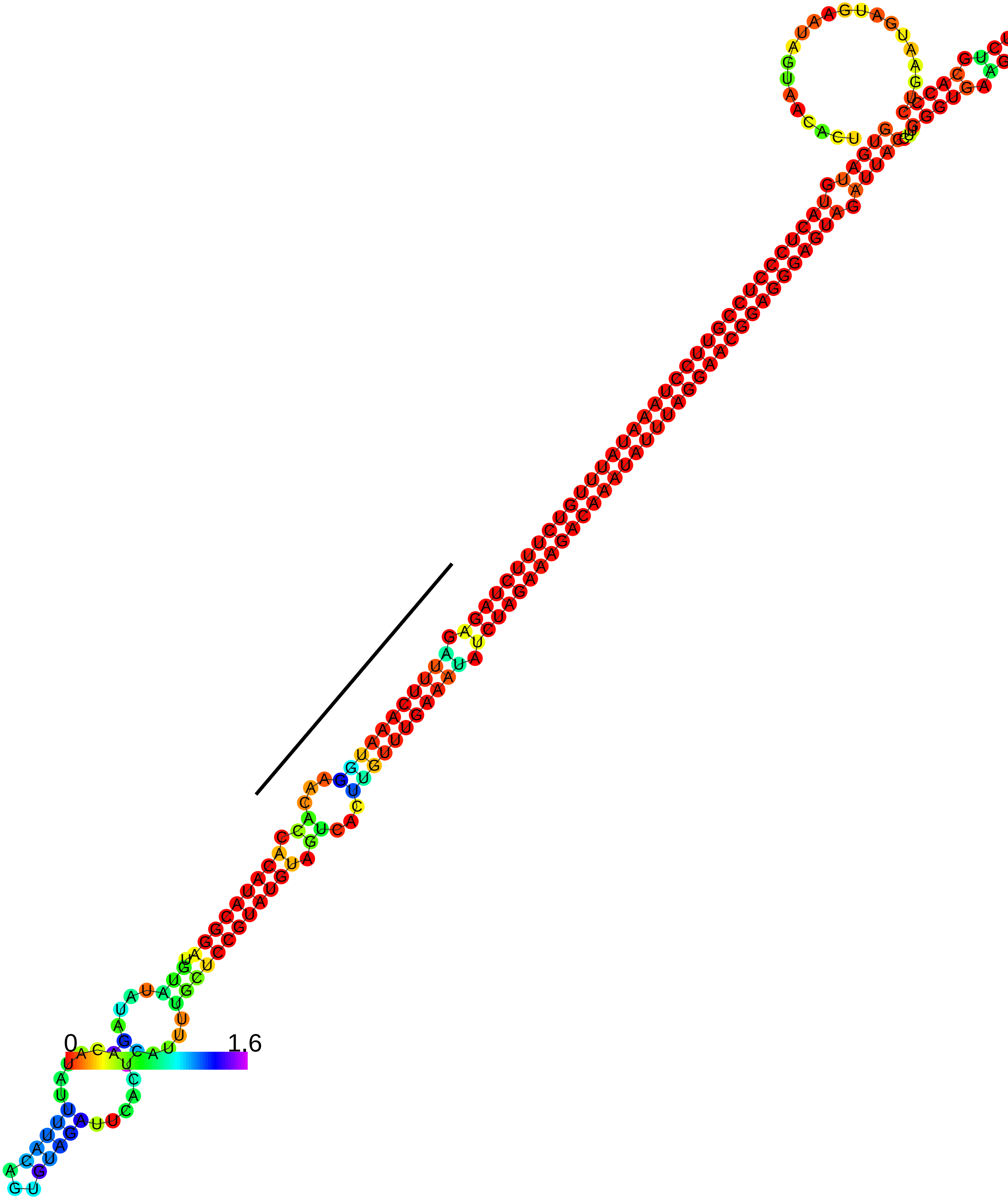

MITE_miRNA_3

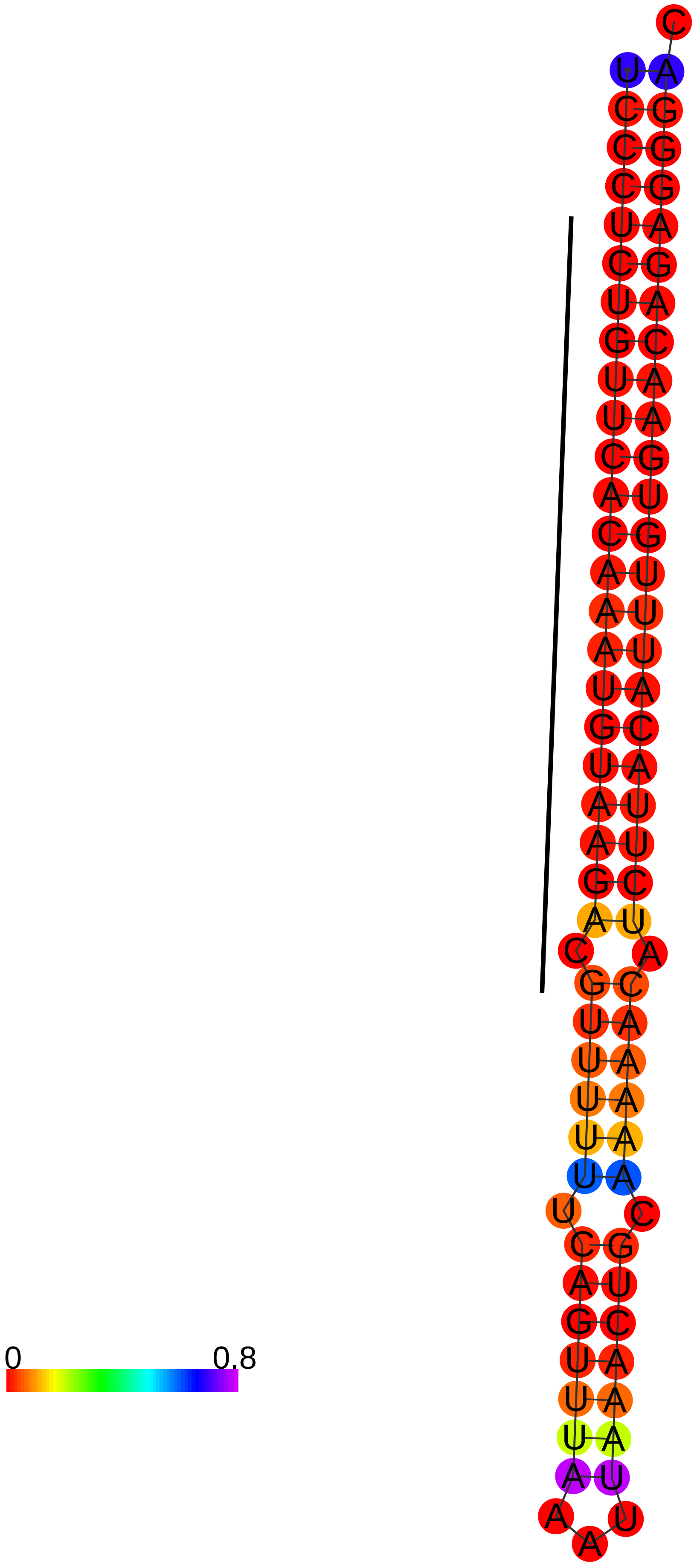

MITE_miRNA_4

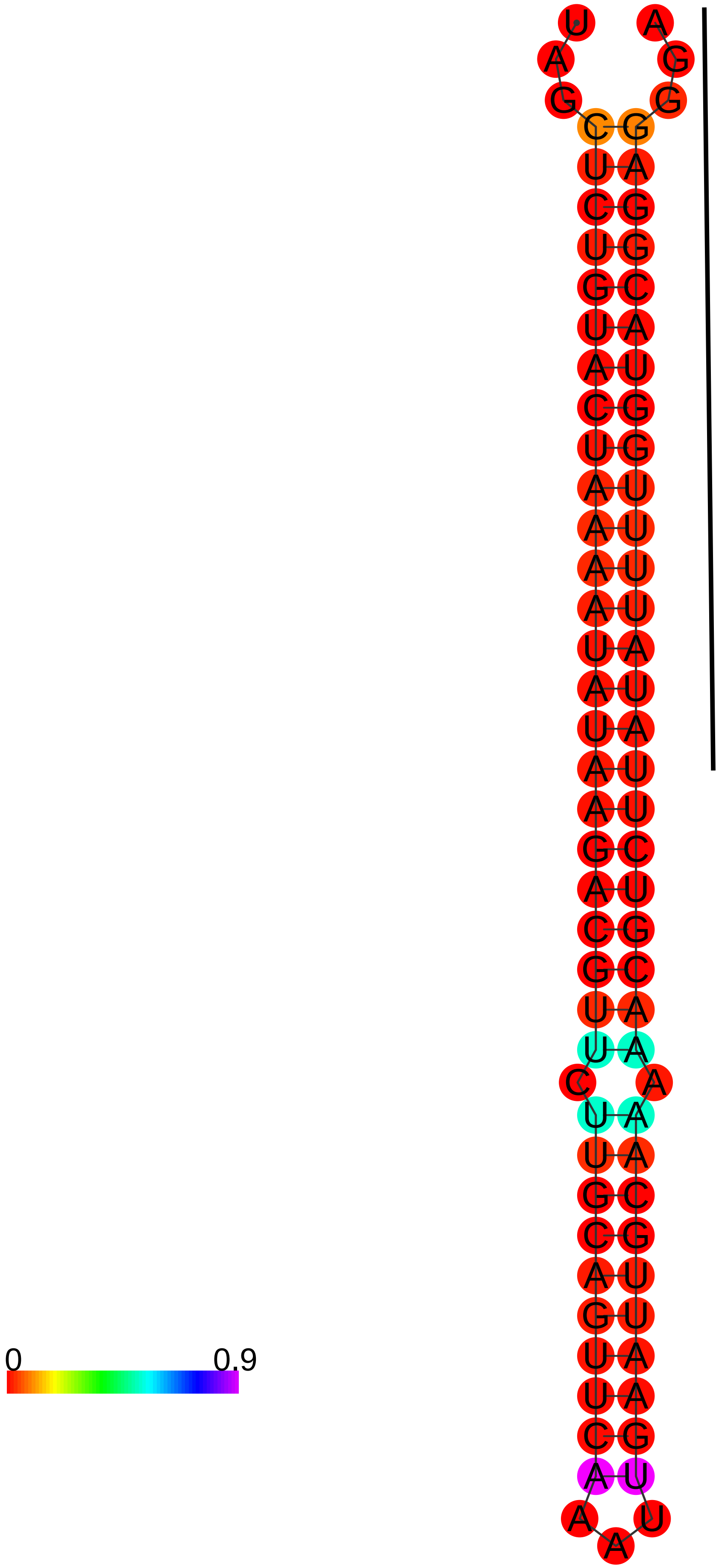

MITE_miRNA_5

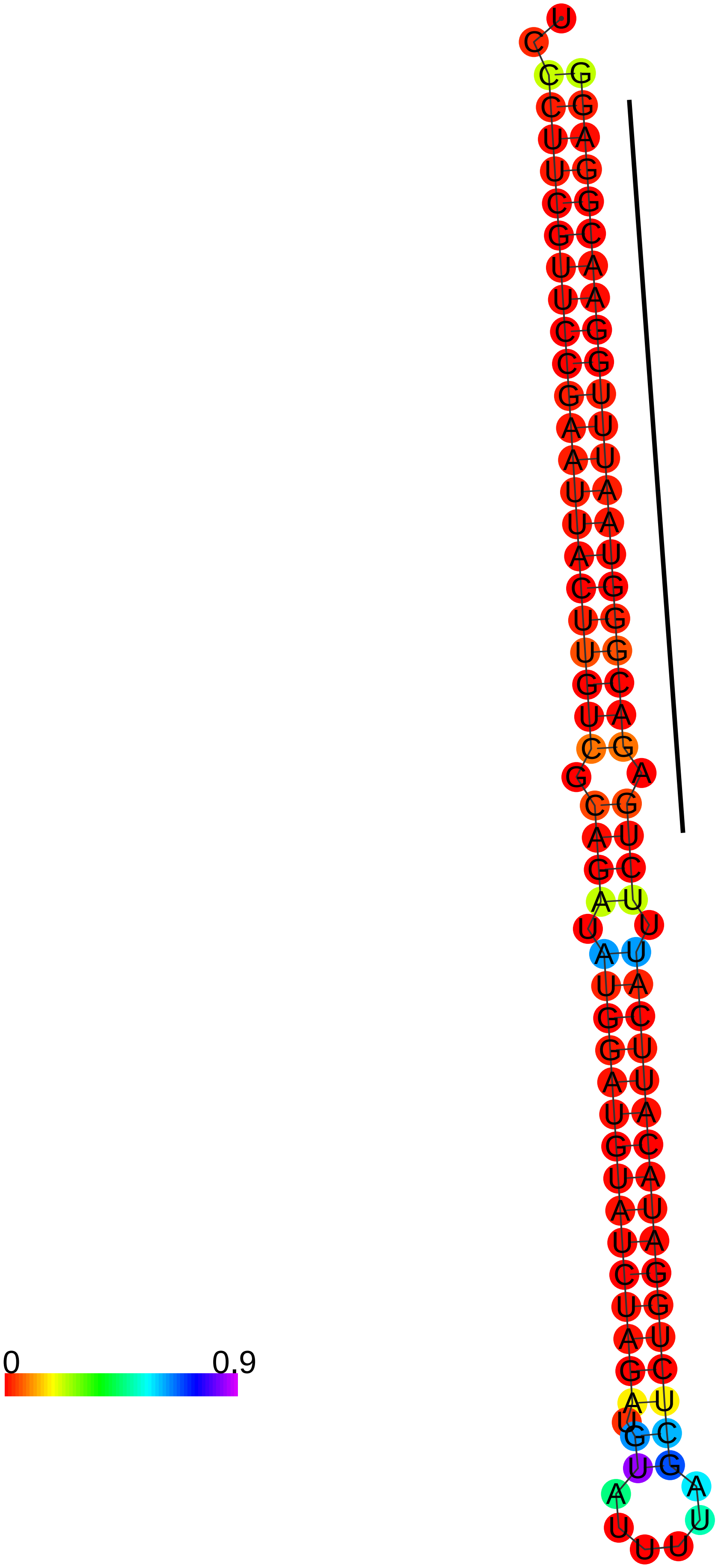

MITE_miRNA_6

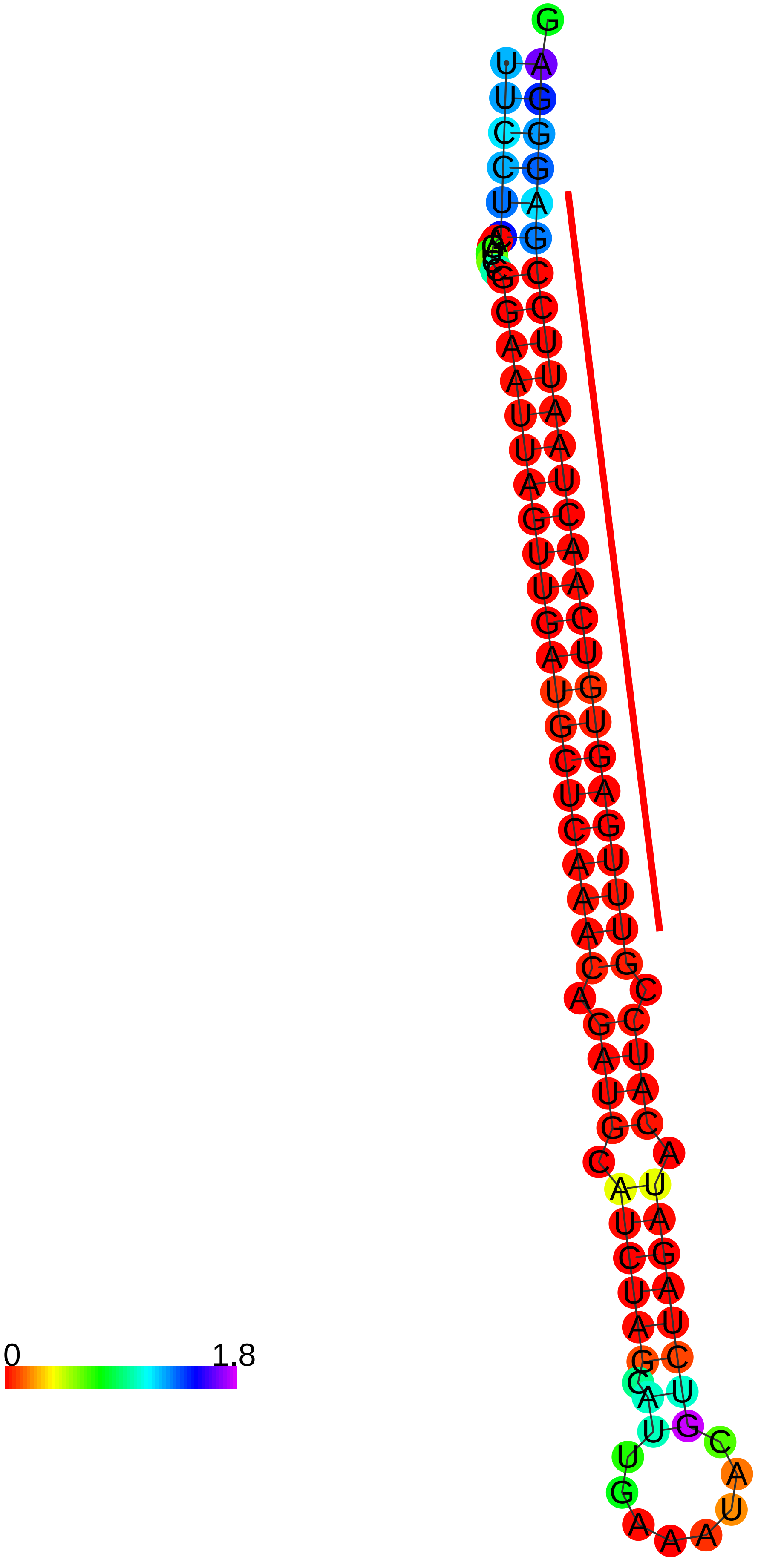

MITE_miRNA_7

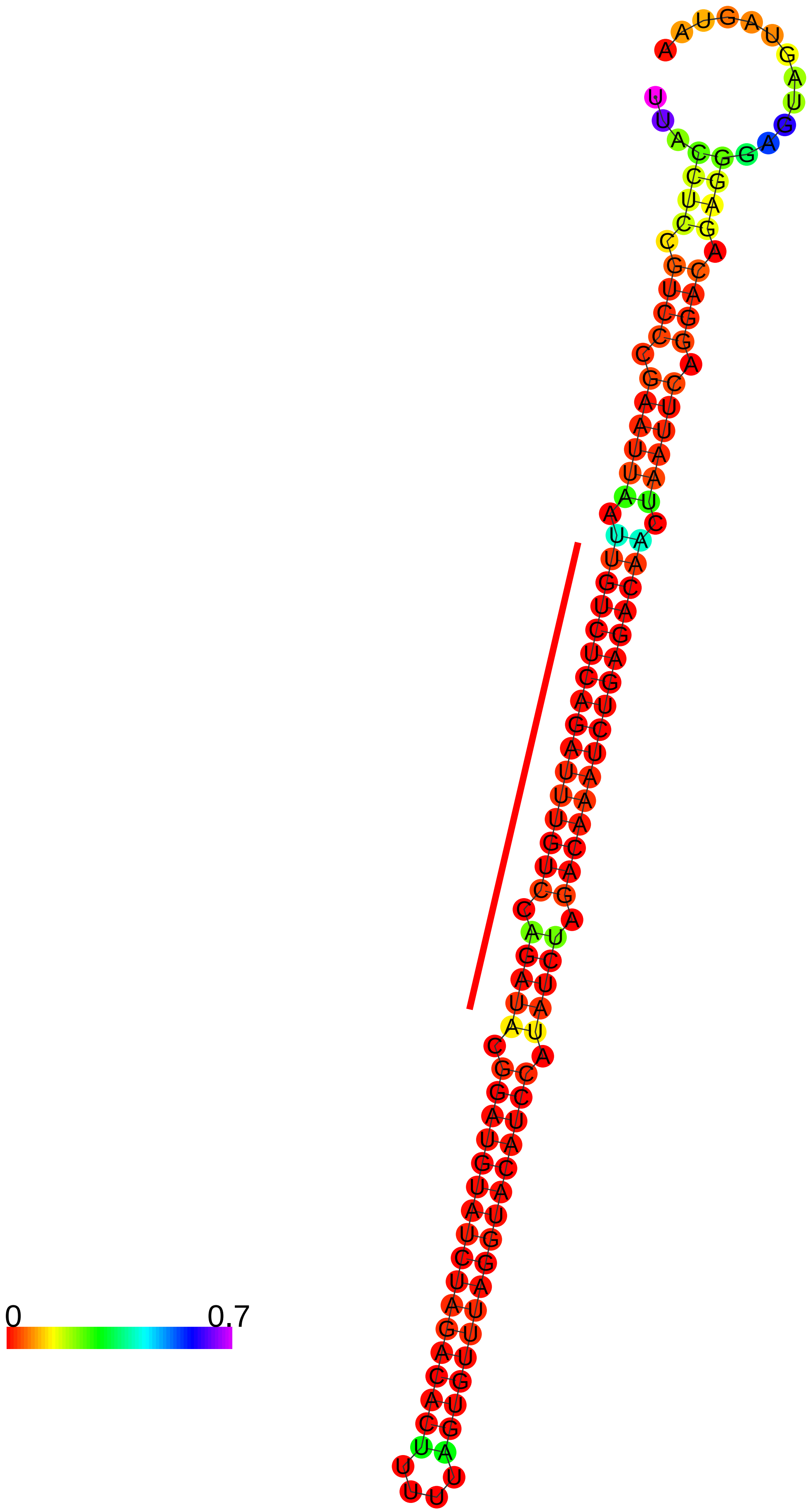

MITE_miRNA_8

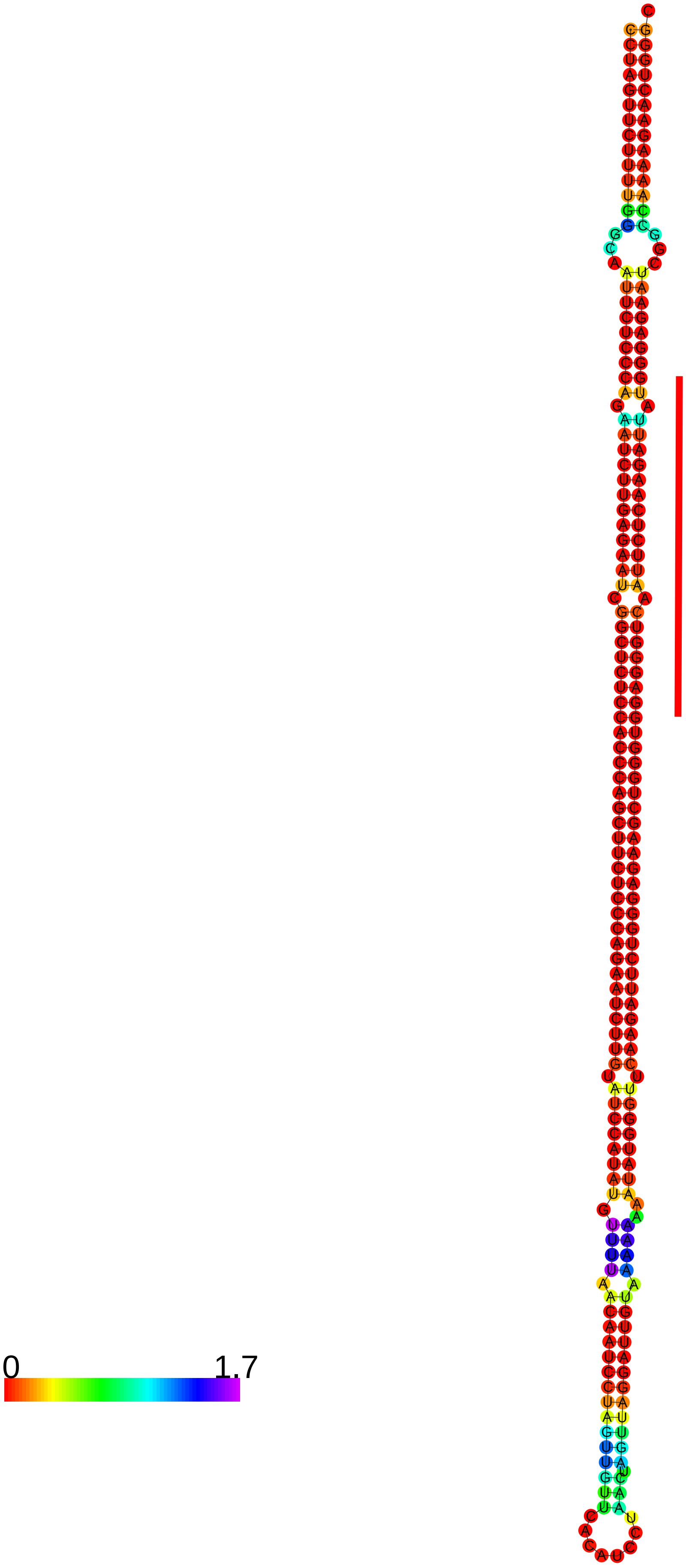

MITE_miRNA_9

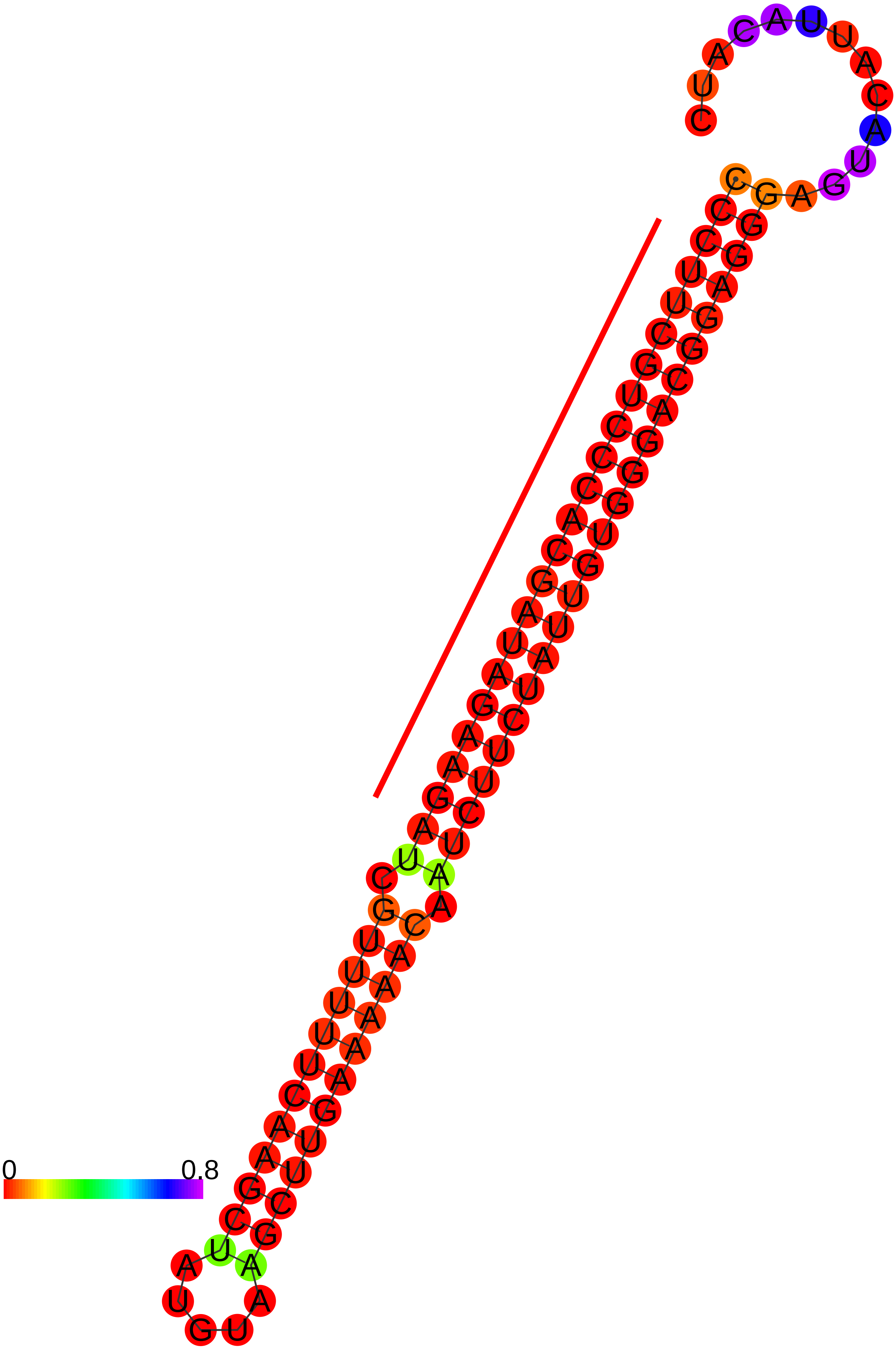

MITE_miRNA_10

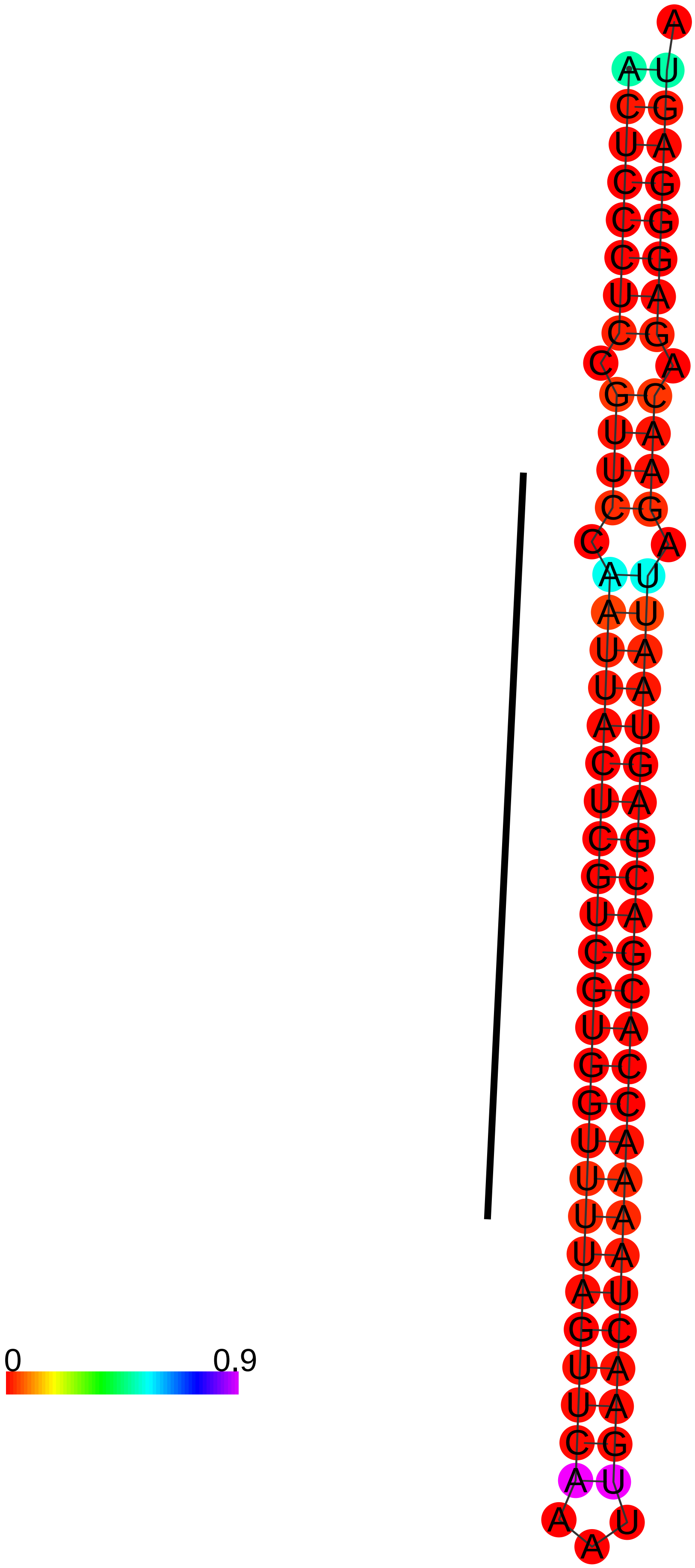

MITE_miRNA_11

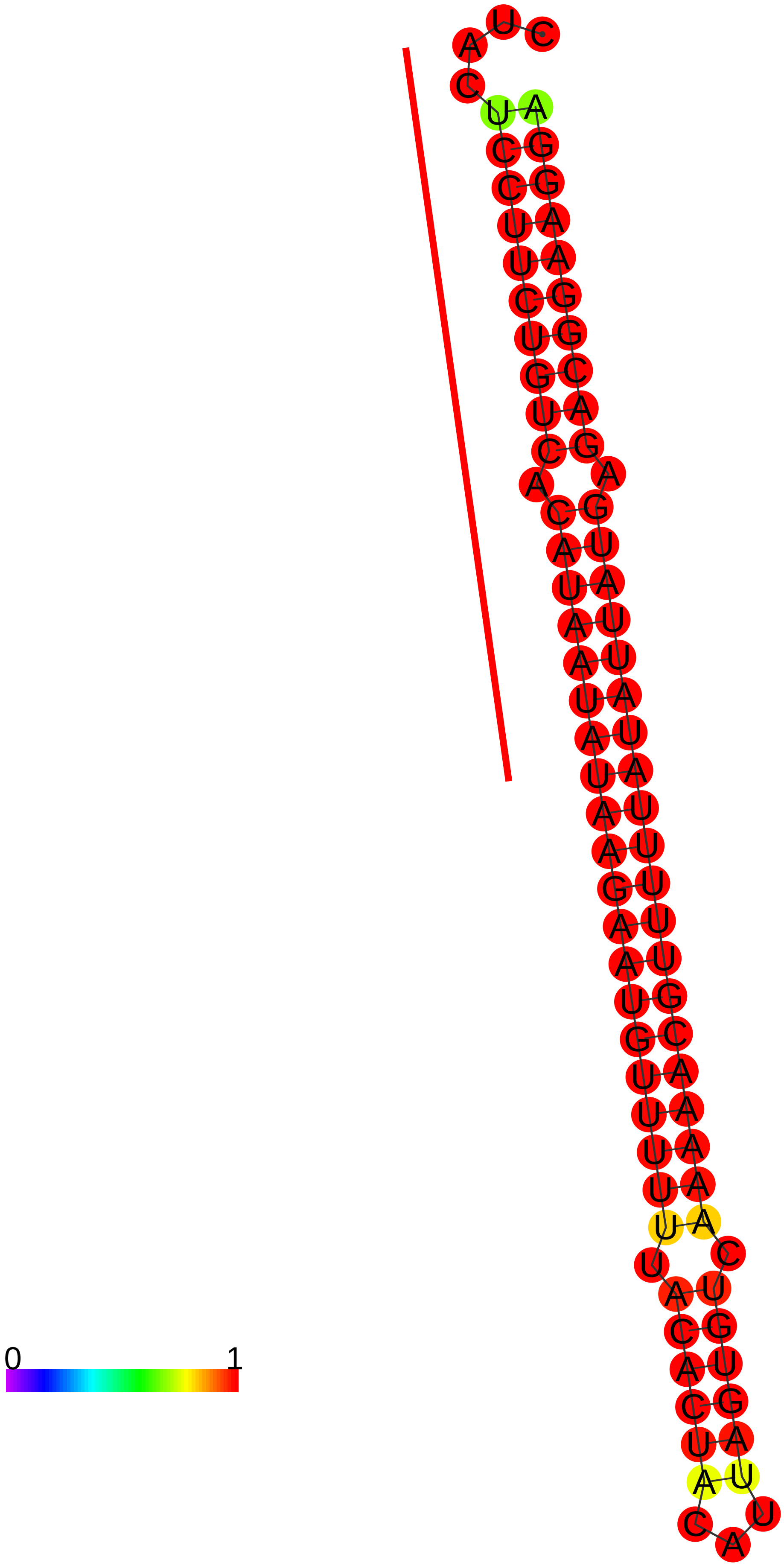

MITE_miRNA_12

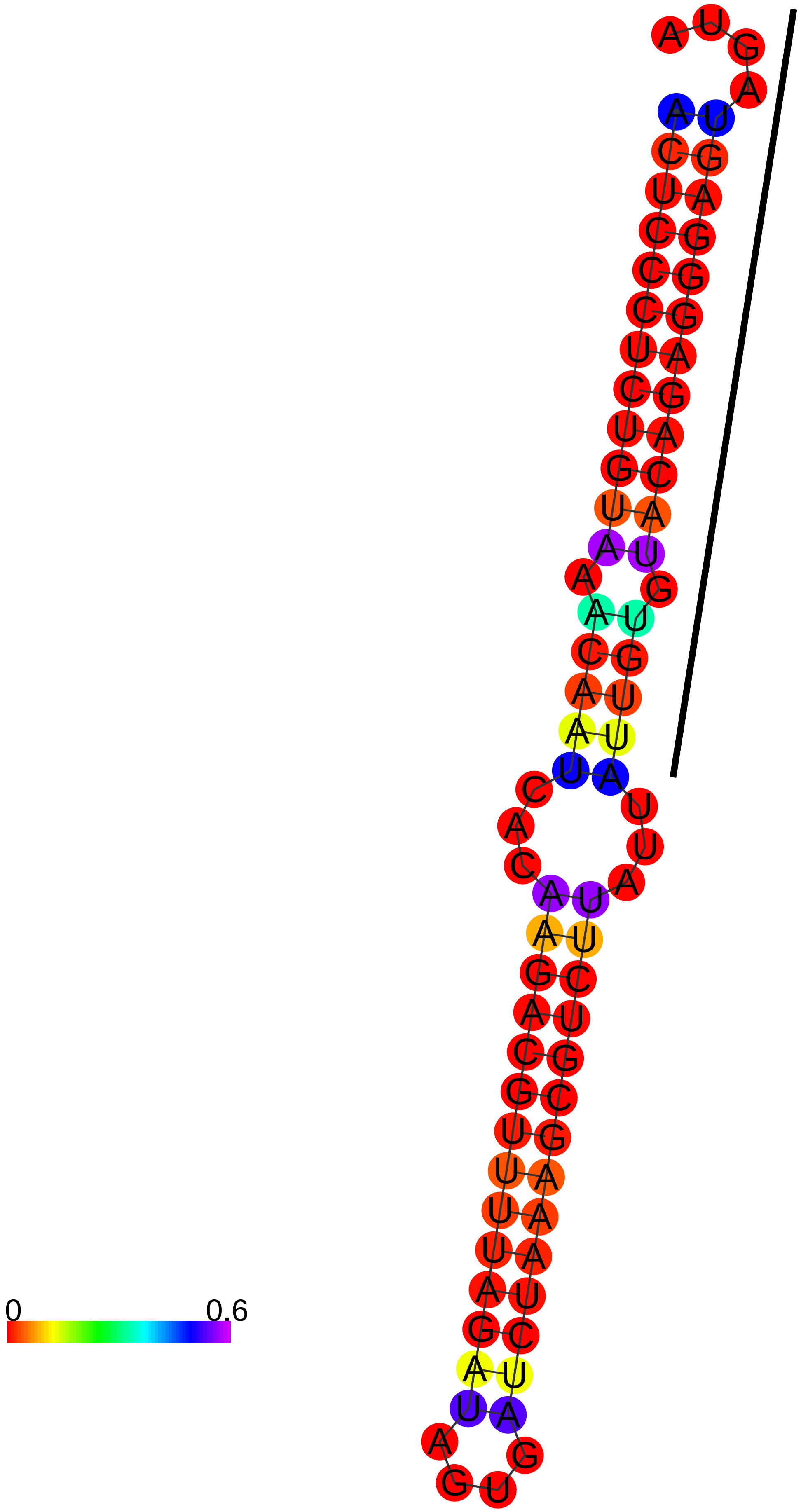

MITE_miRNA_13

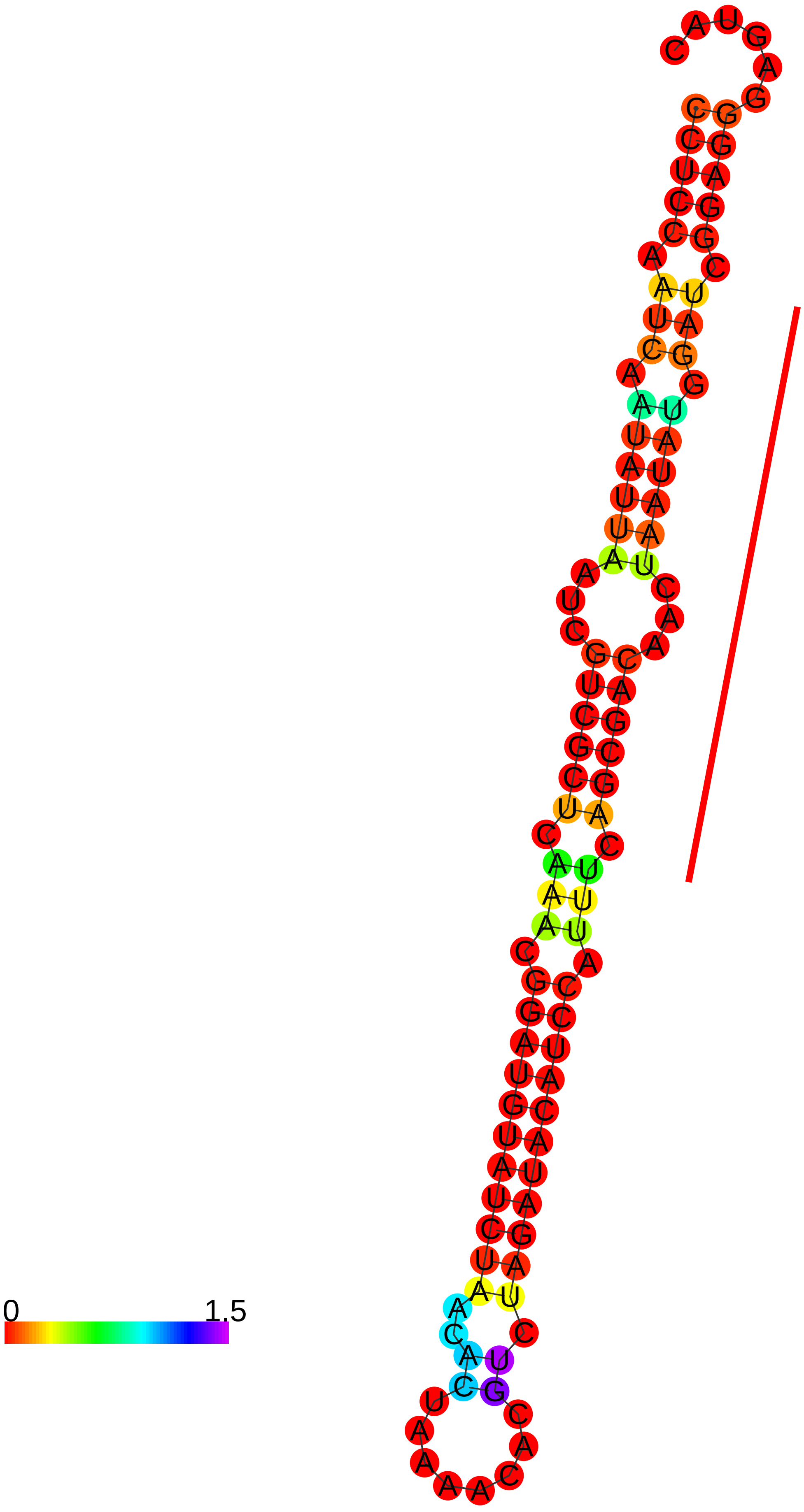

MITE_miRNA_14

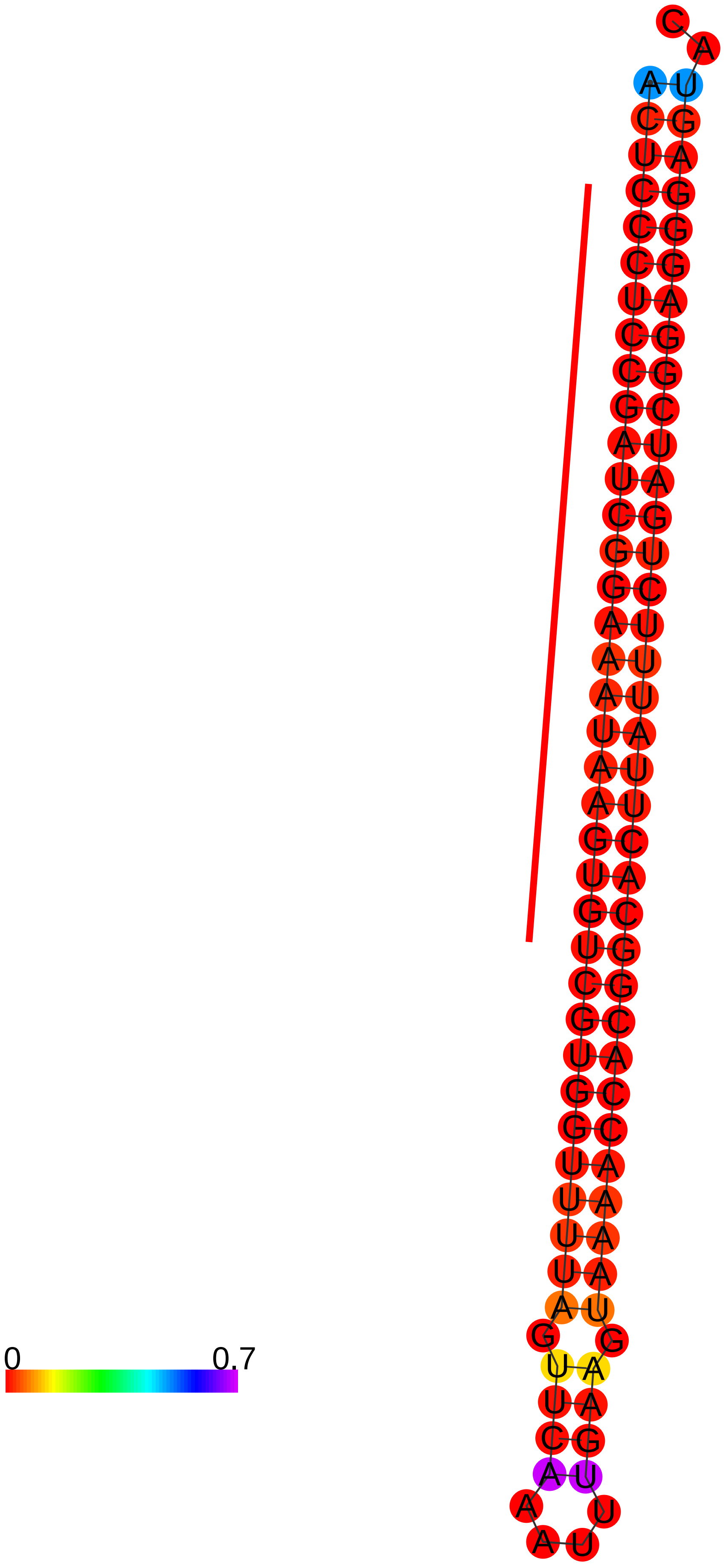

MITE_miRNA_15

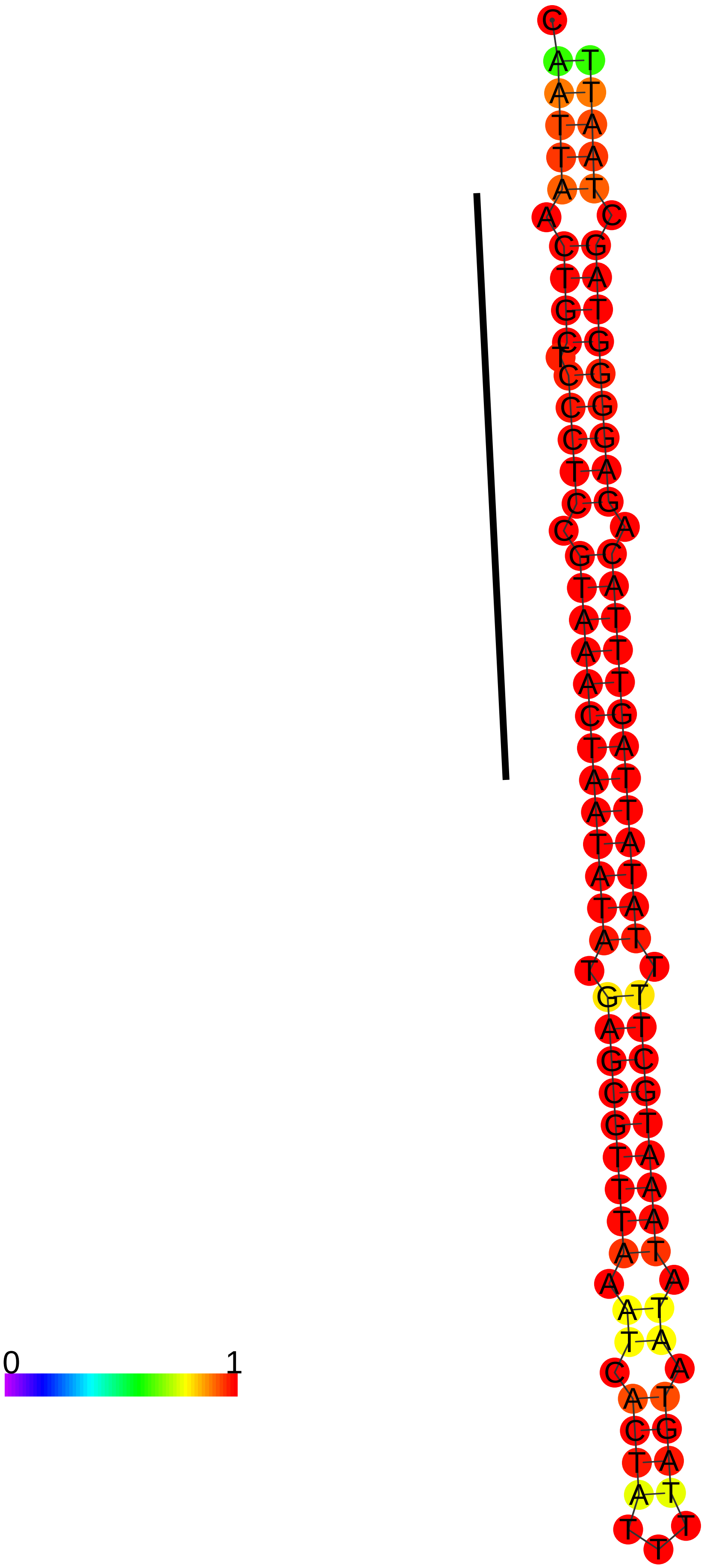

MITE_miRNA_16

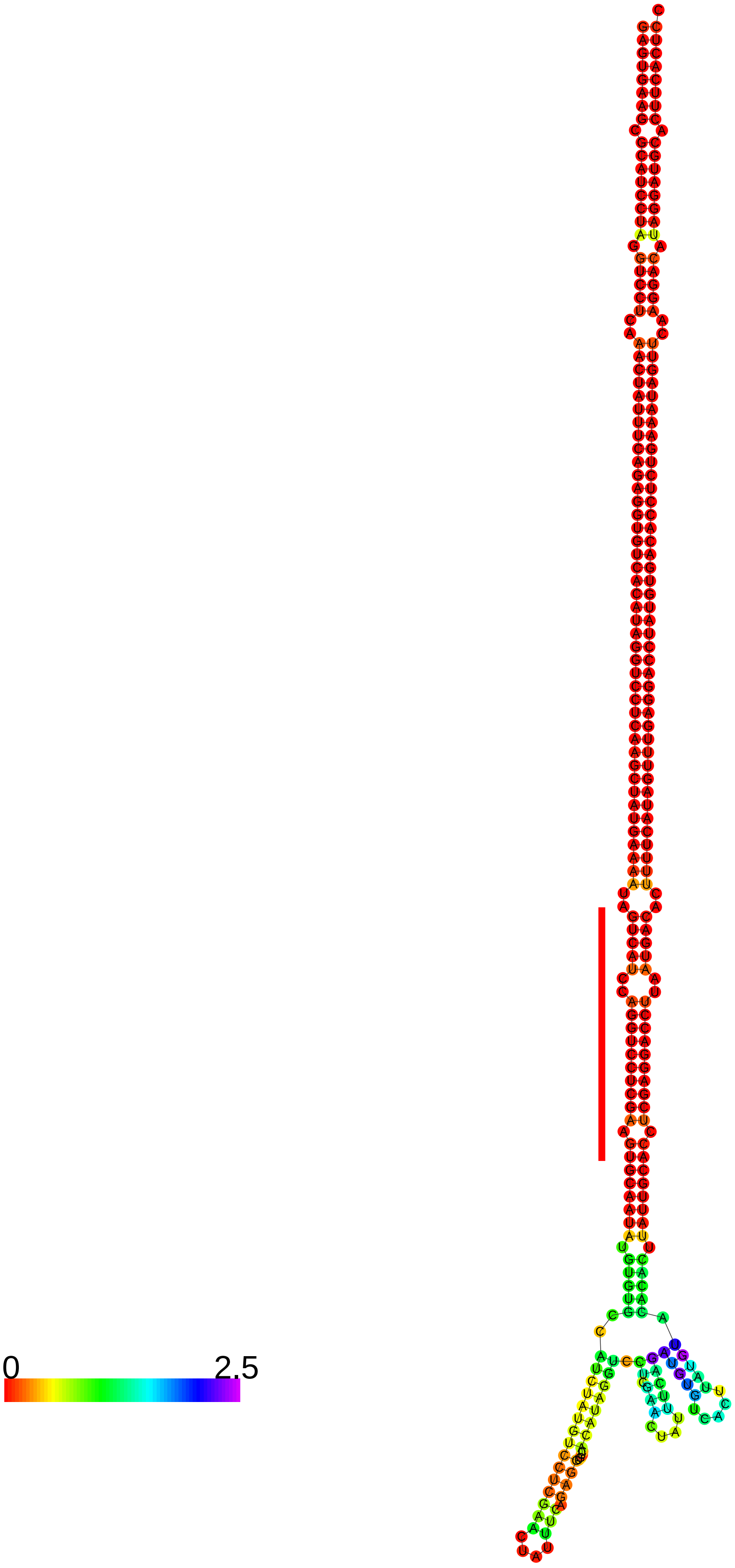

MITE_miRNA_17

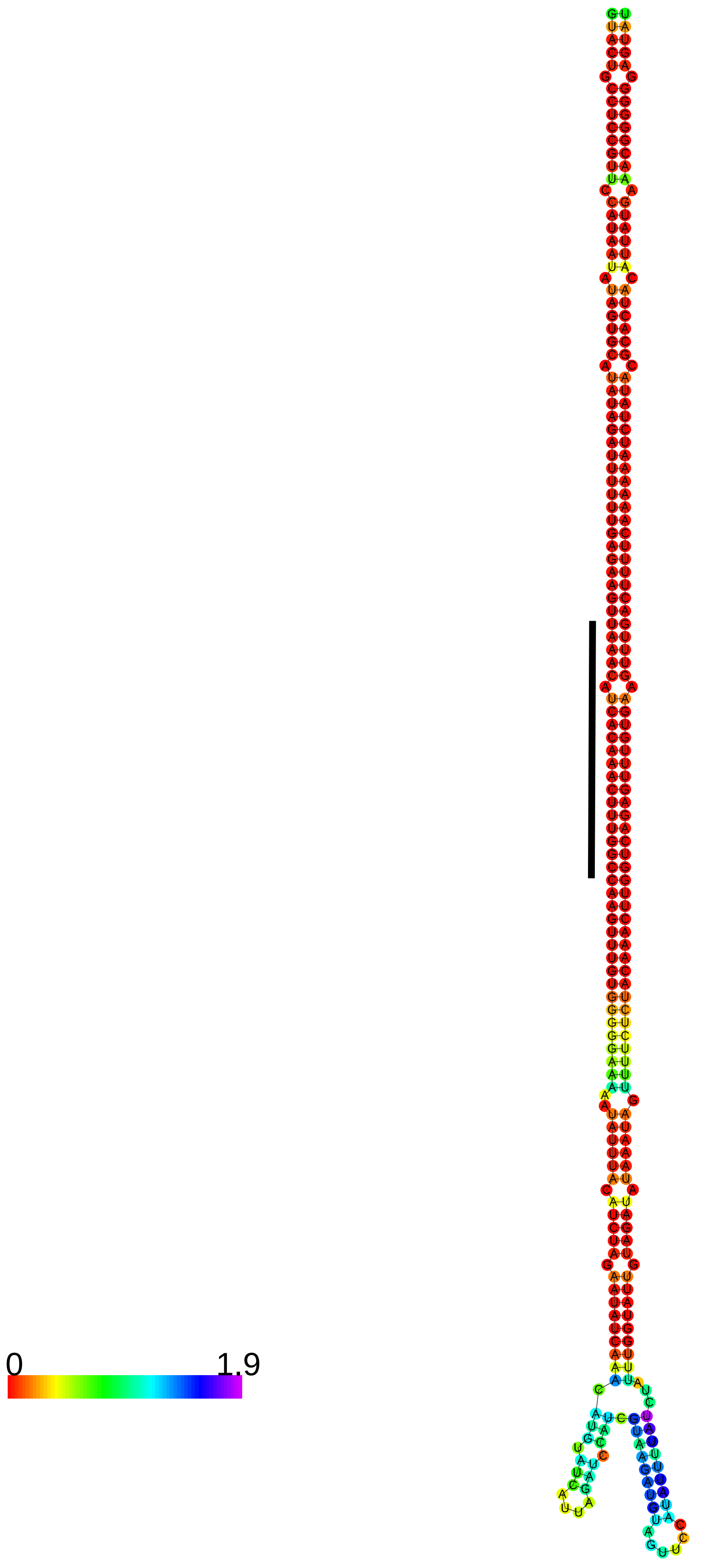

MITE_miRNA_18

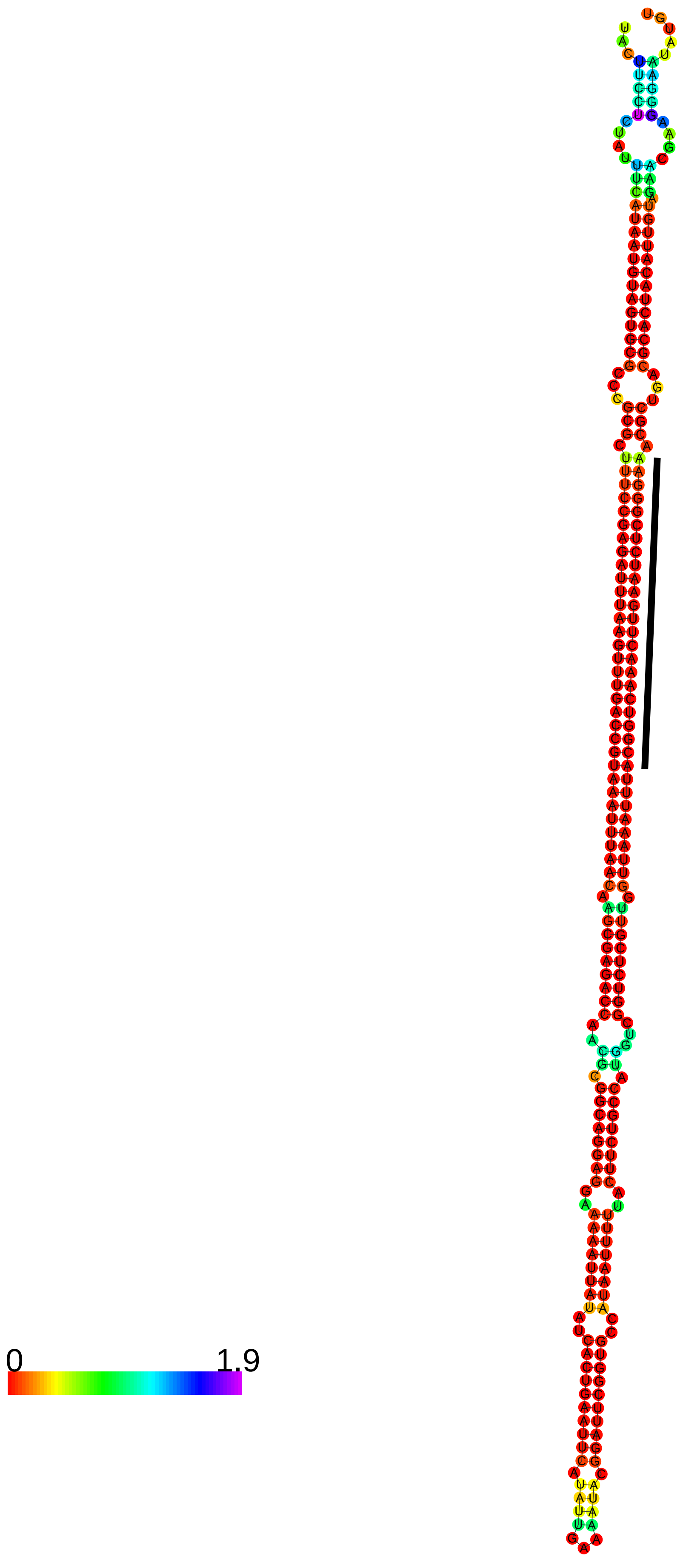

MITE_miRNA_19

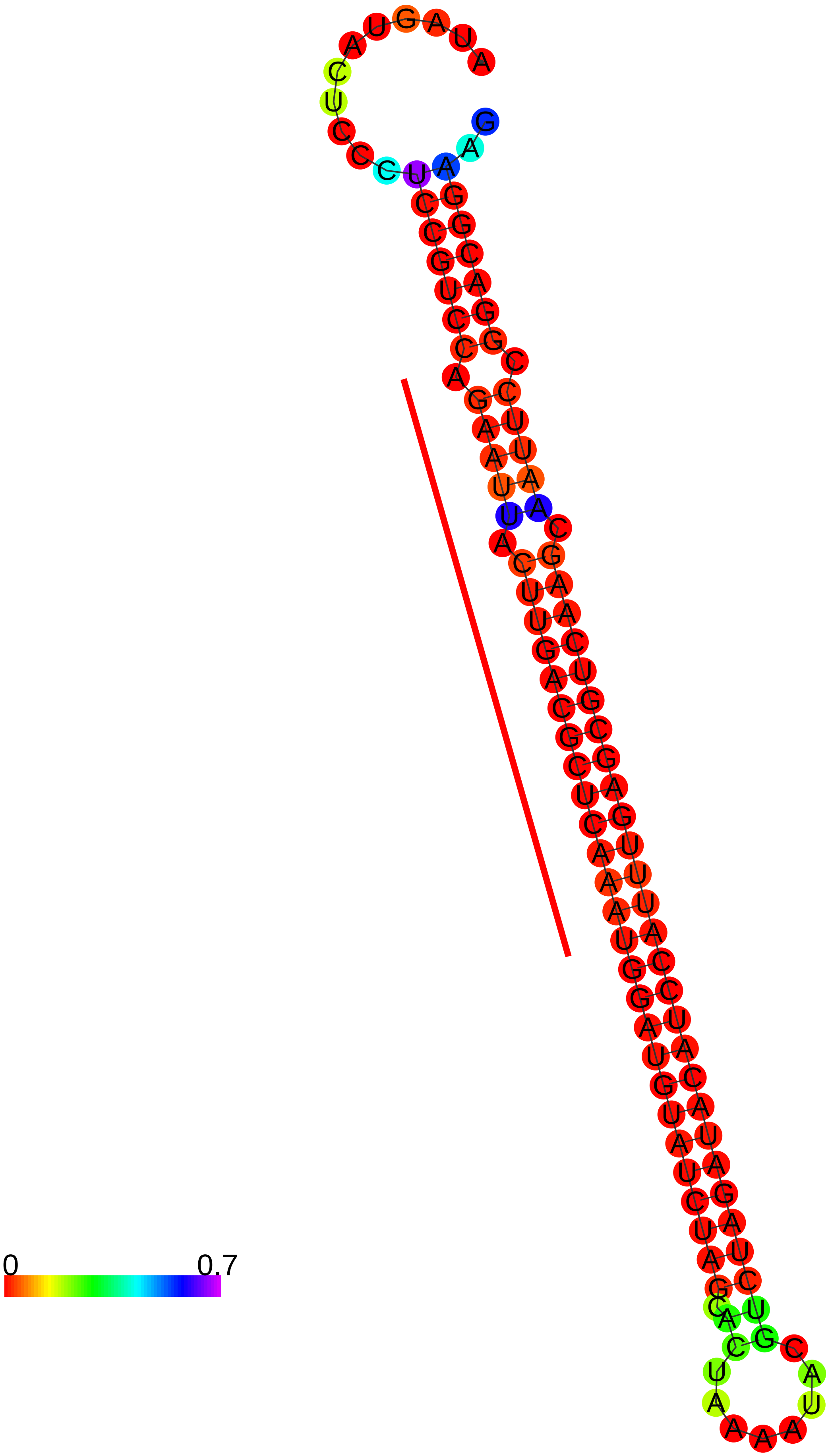

MITE_miRNA_20

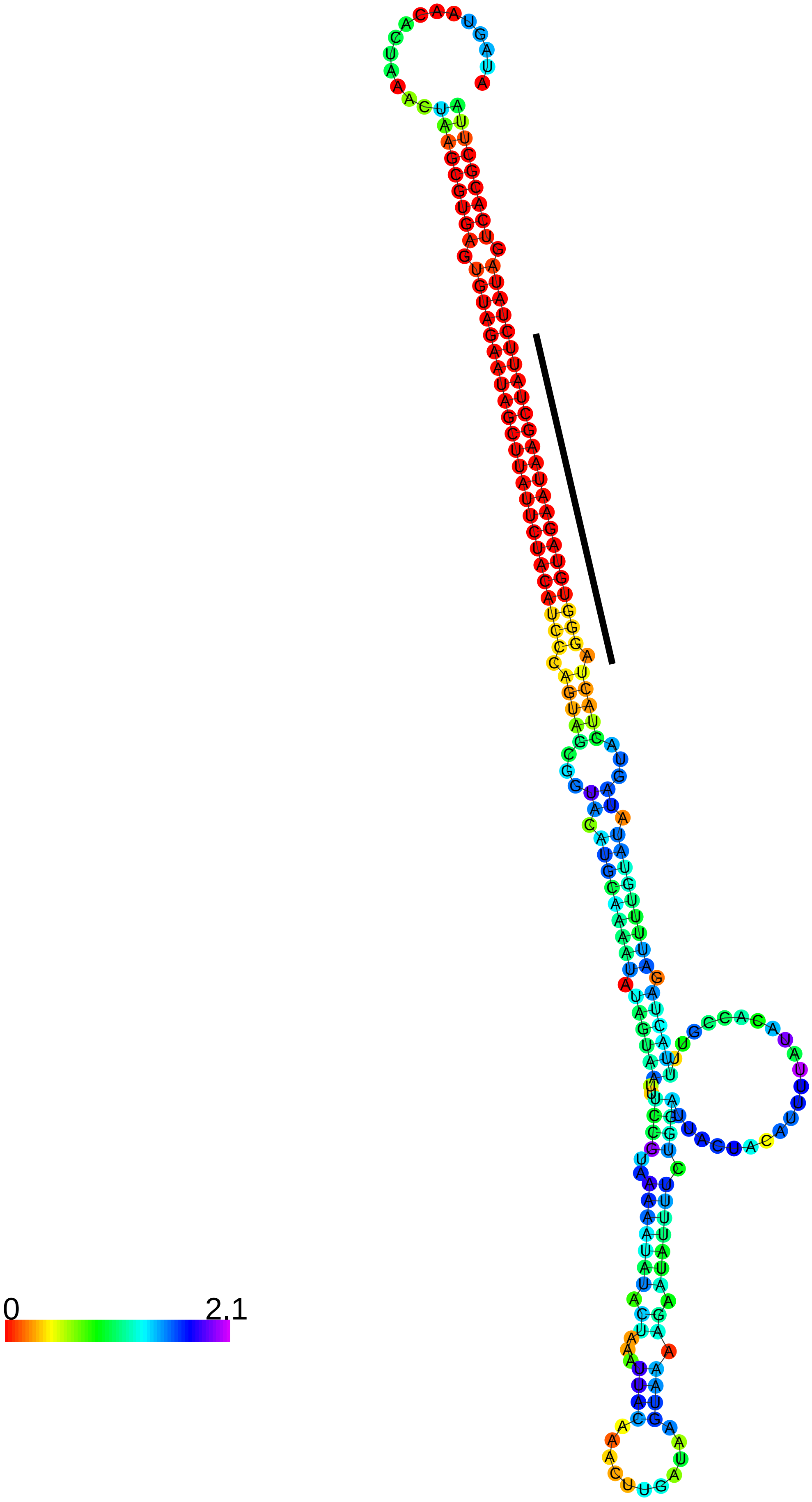

MITE_miRNA_21

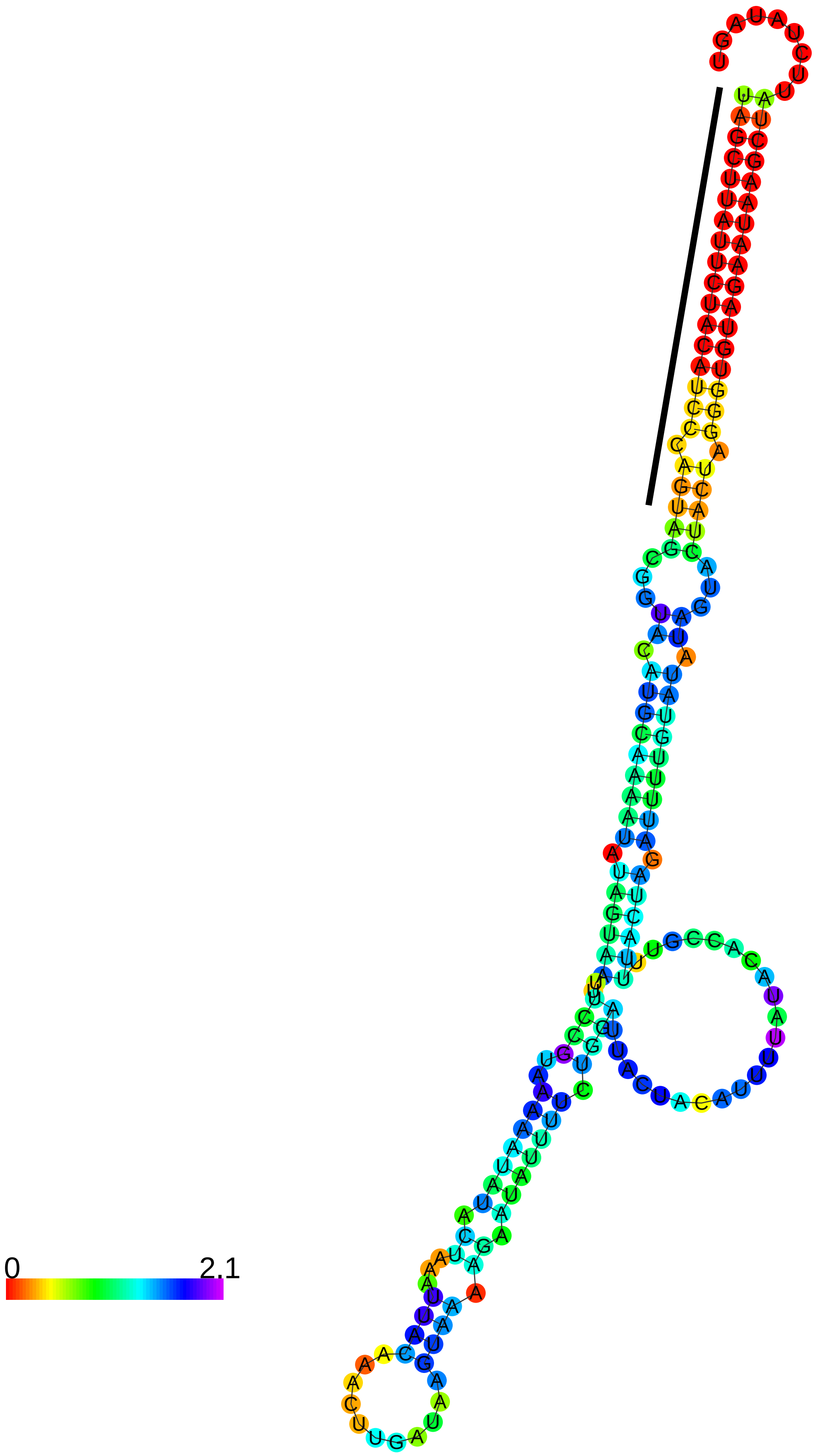

MITE_miRNA_22

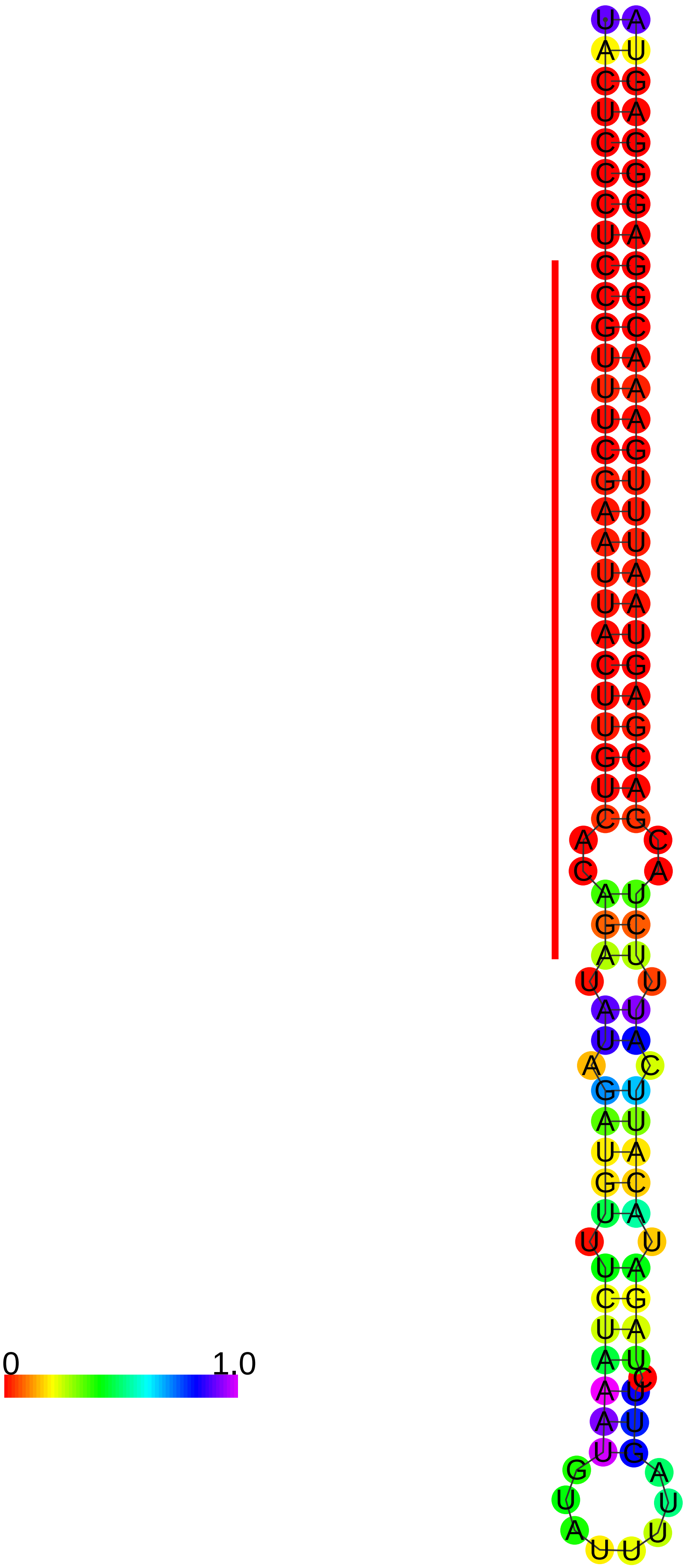

MITE_miRNA_23

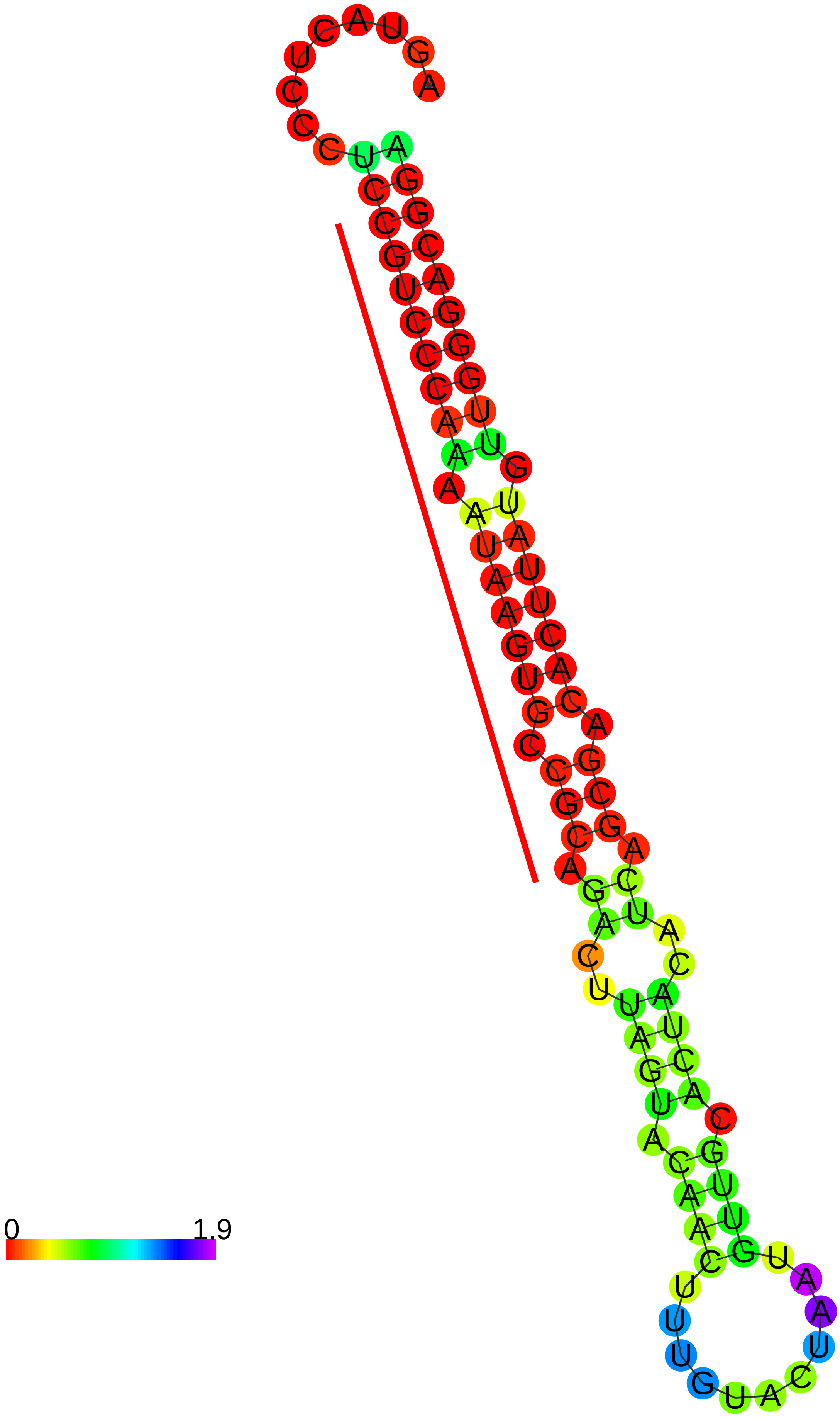

MITE_miRNA_24

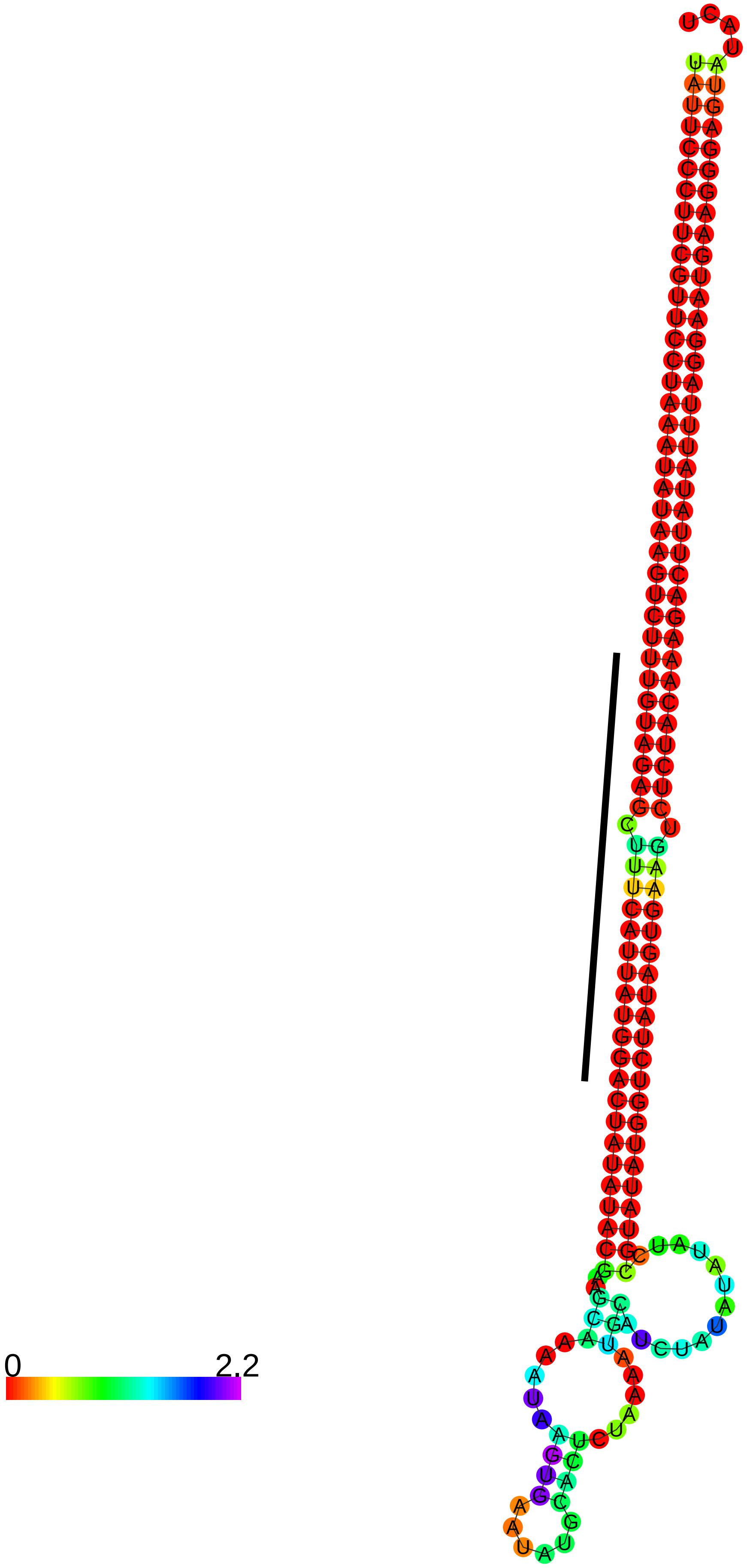

MITE_miRNA_25

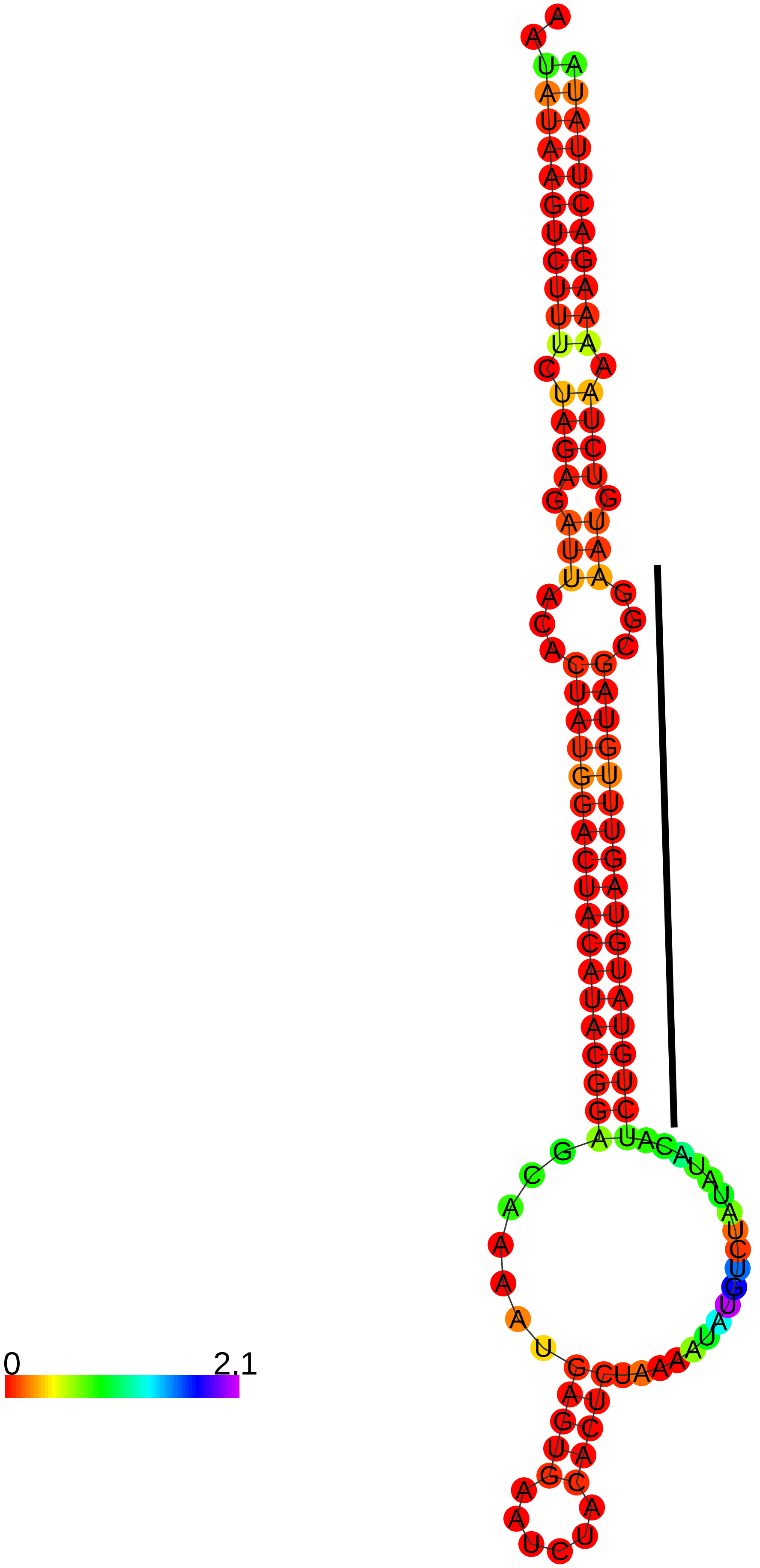

MITE_miRNA_26

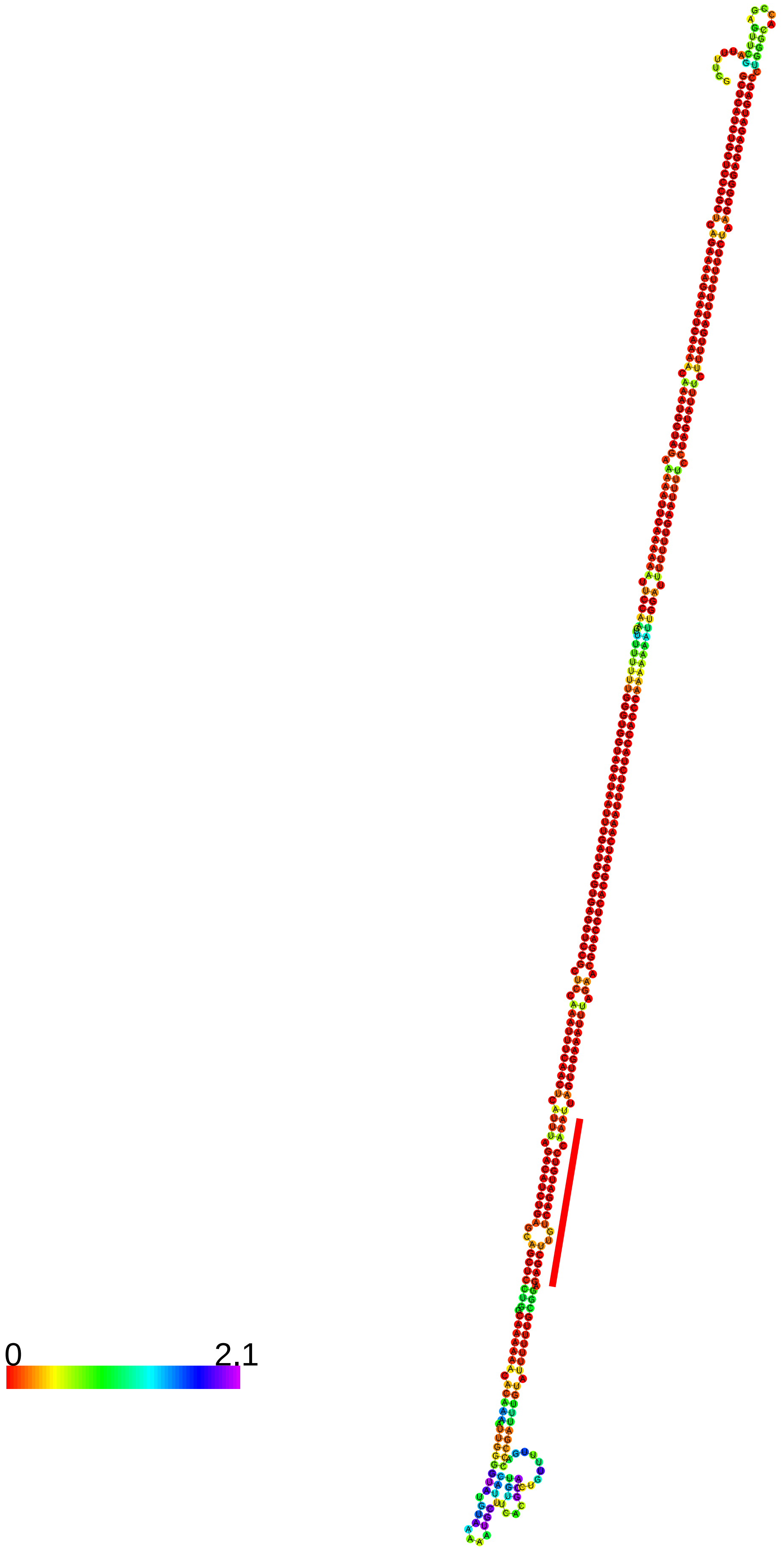

MITE_miRNA_27

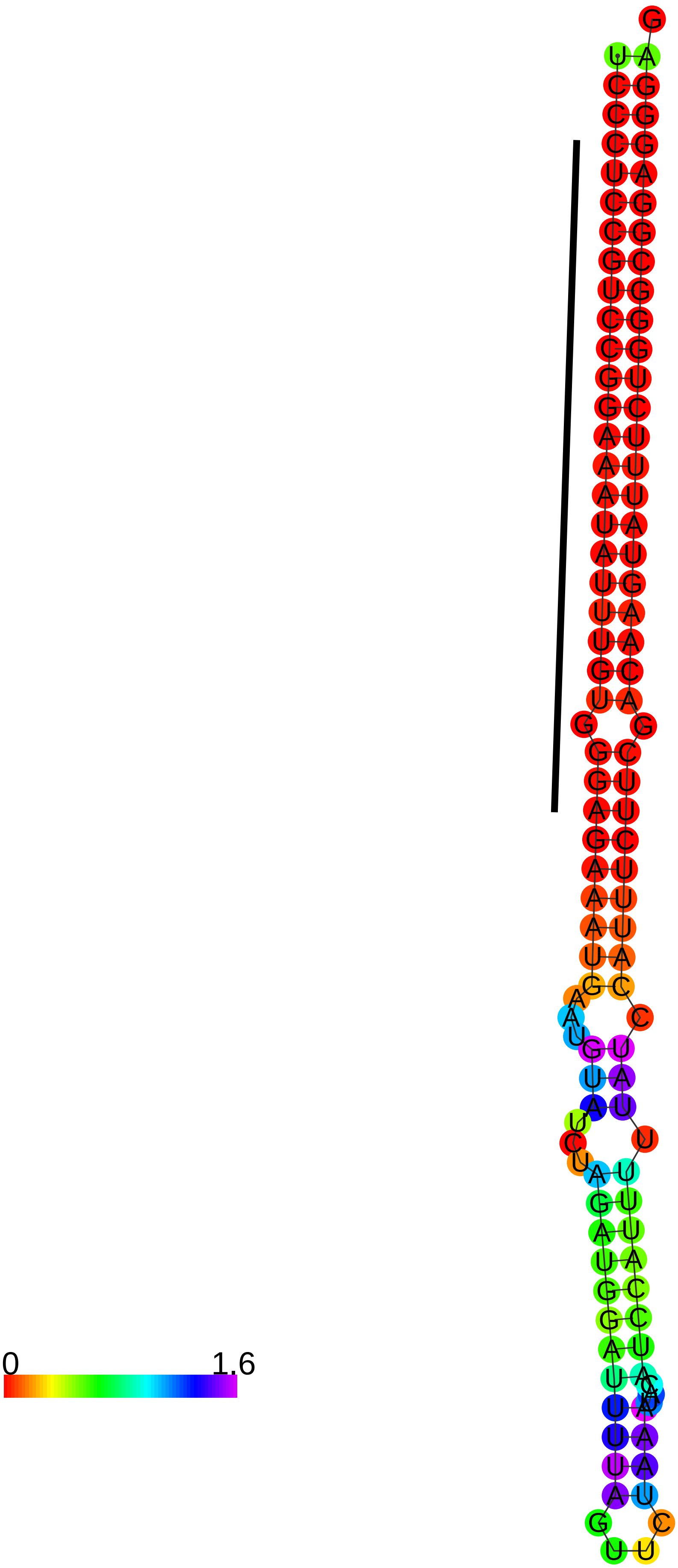

MITE_miRNA_28

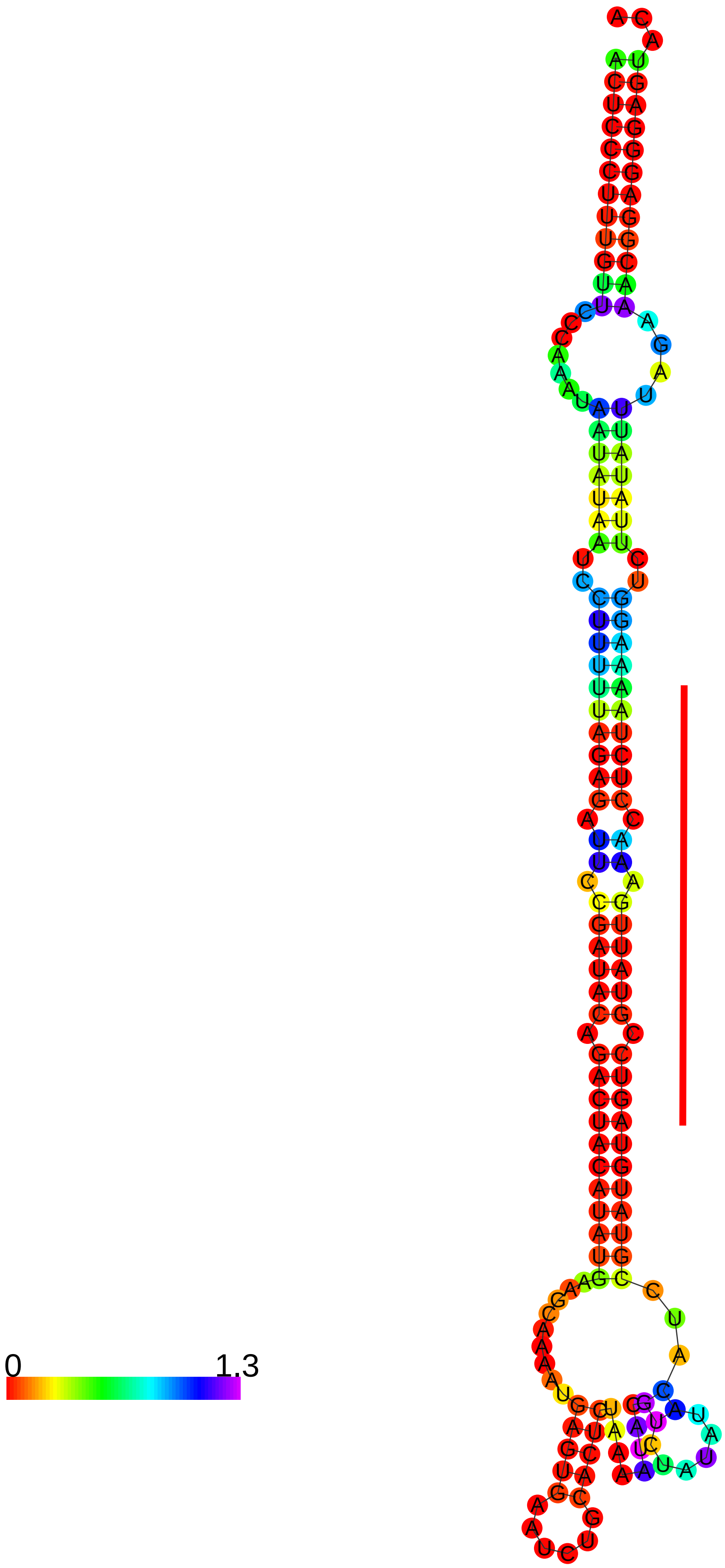

MITE_miRNA_29

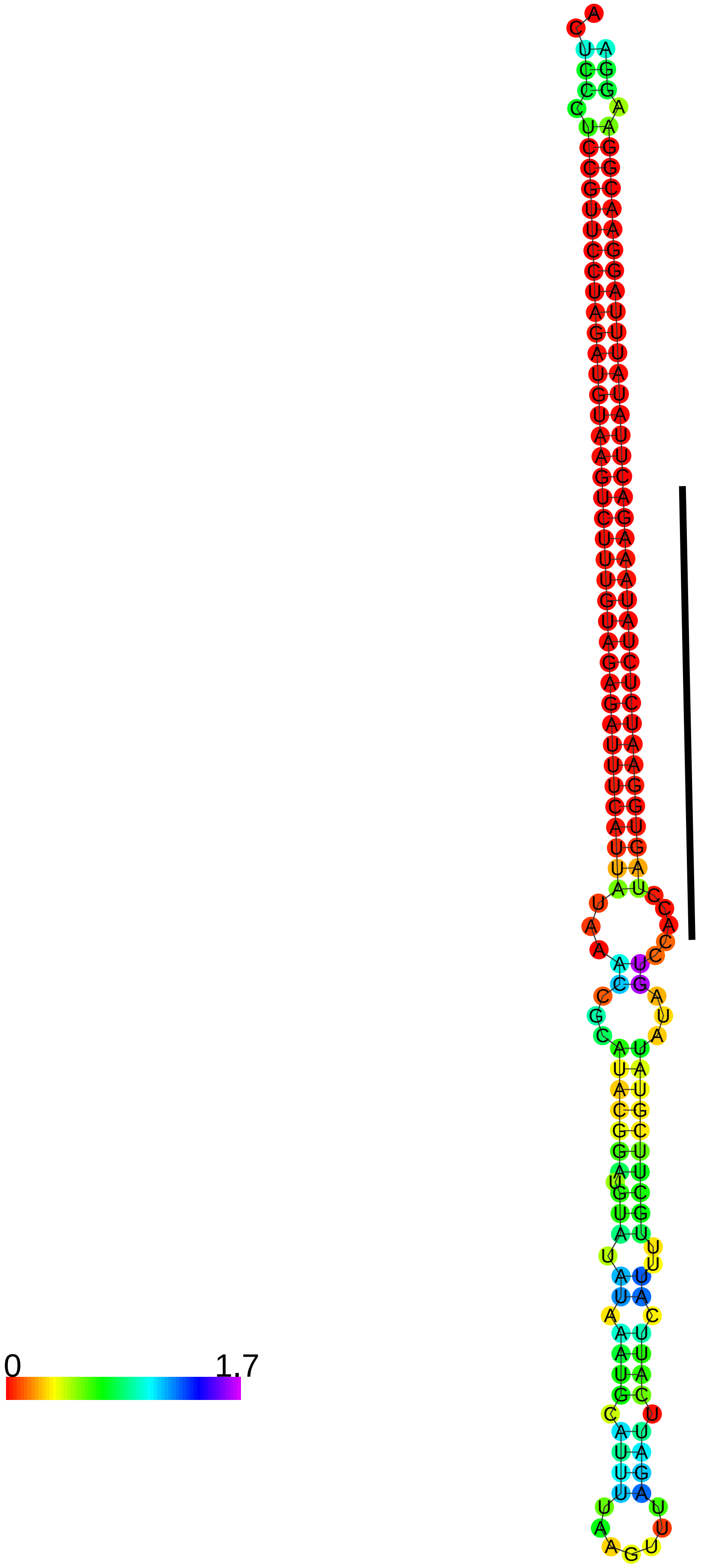

MITE_miRNA_30

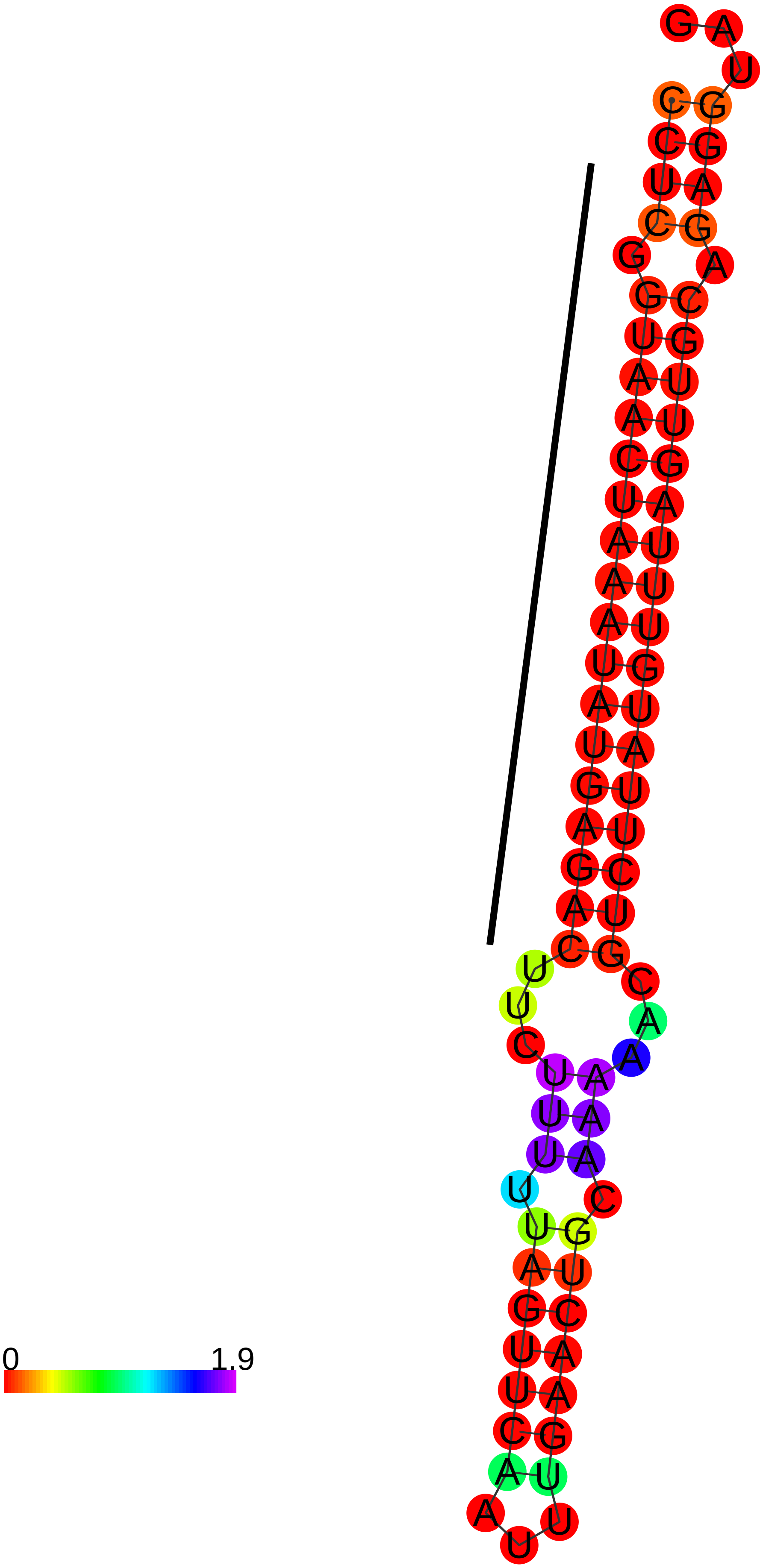

MITE_miRNA_31

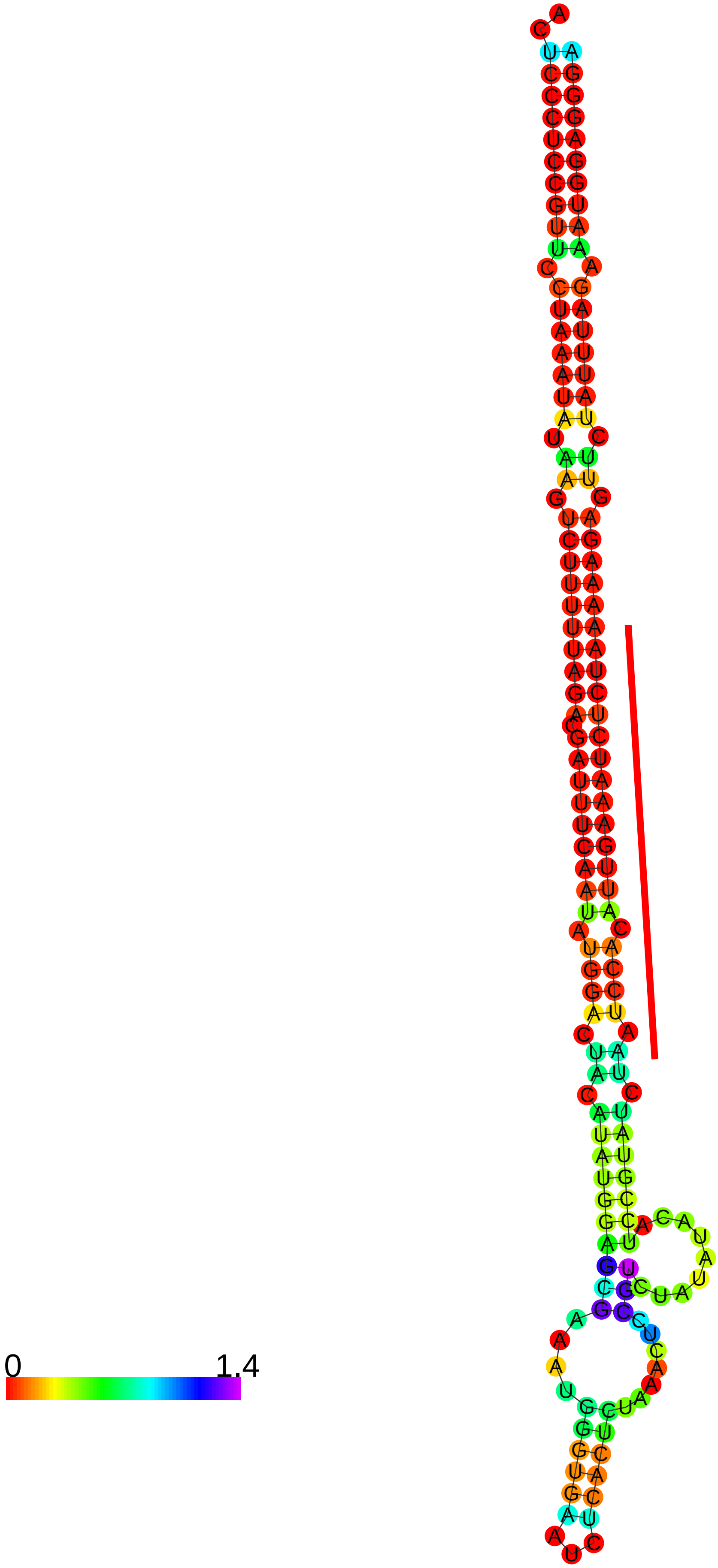

MITE_miRNA_32

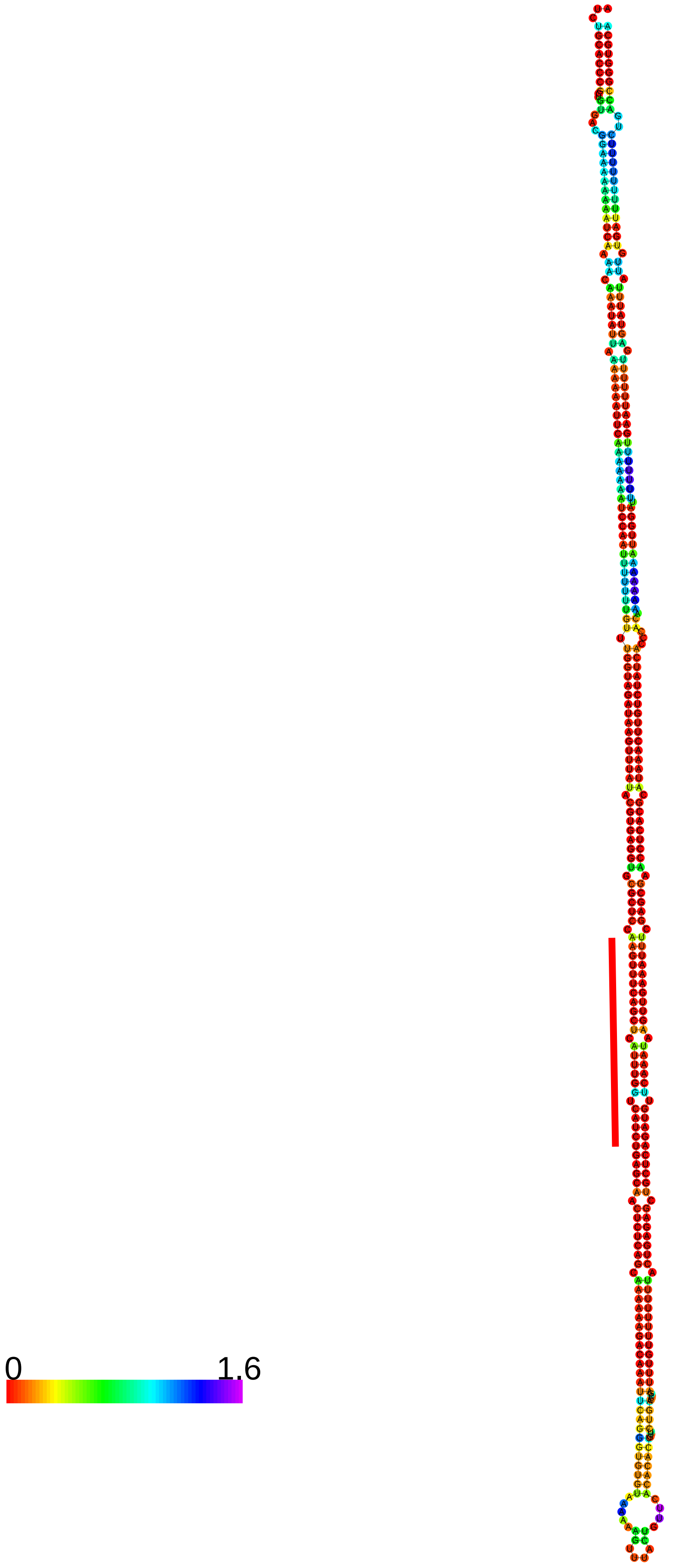

MITE_miRNA_33

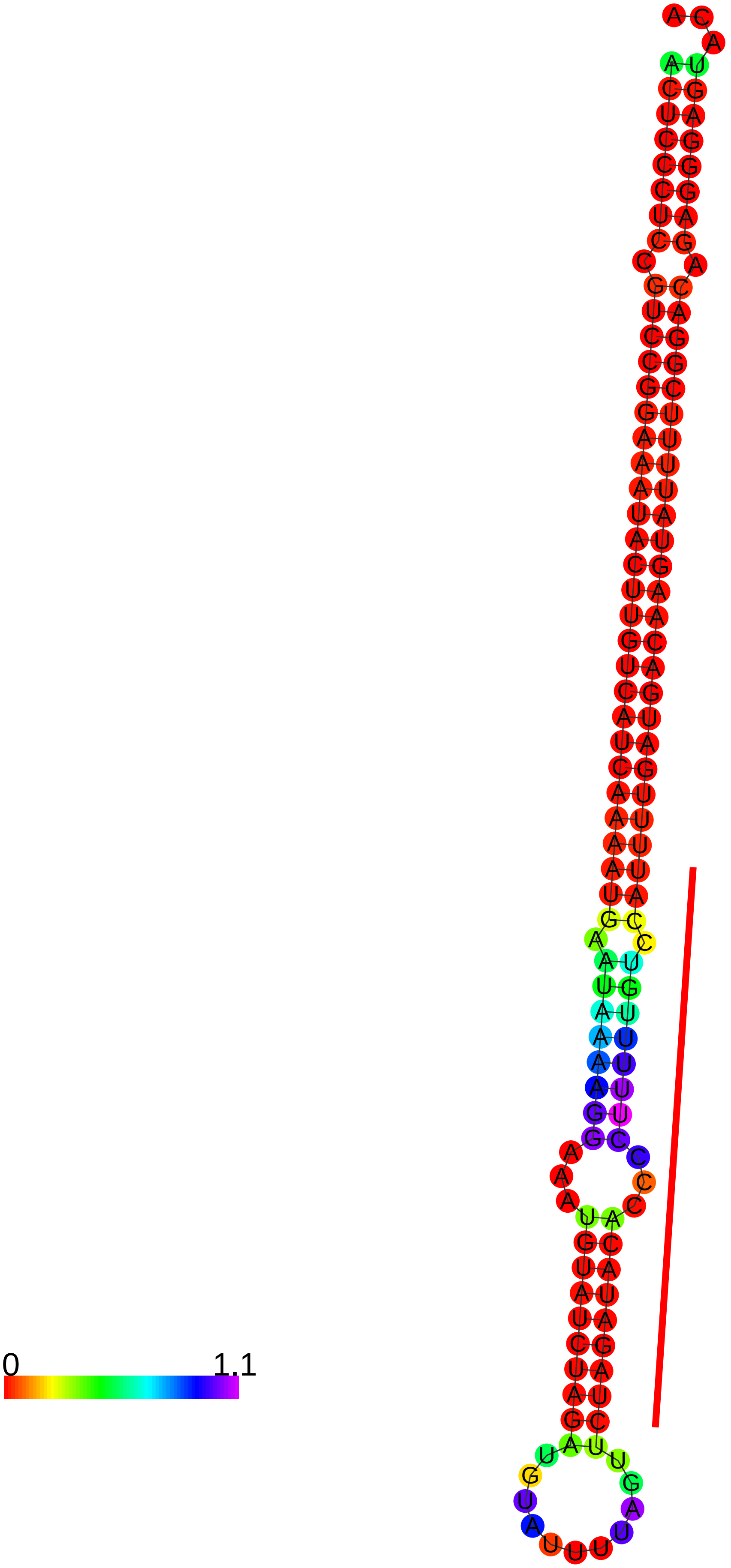

MITE_miRNA_34

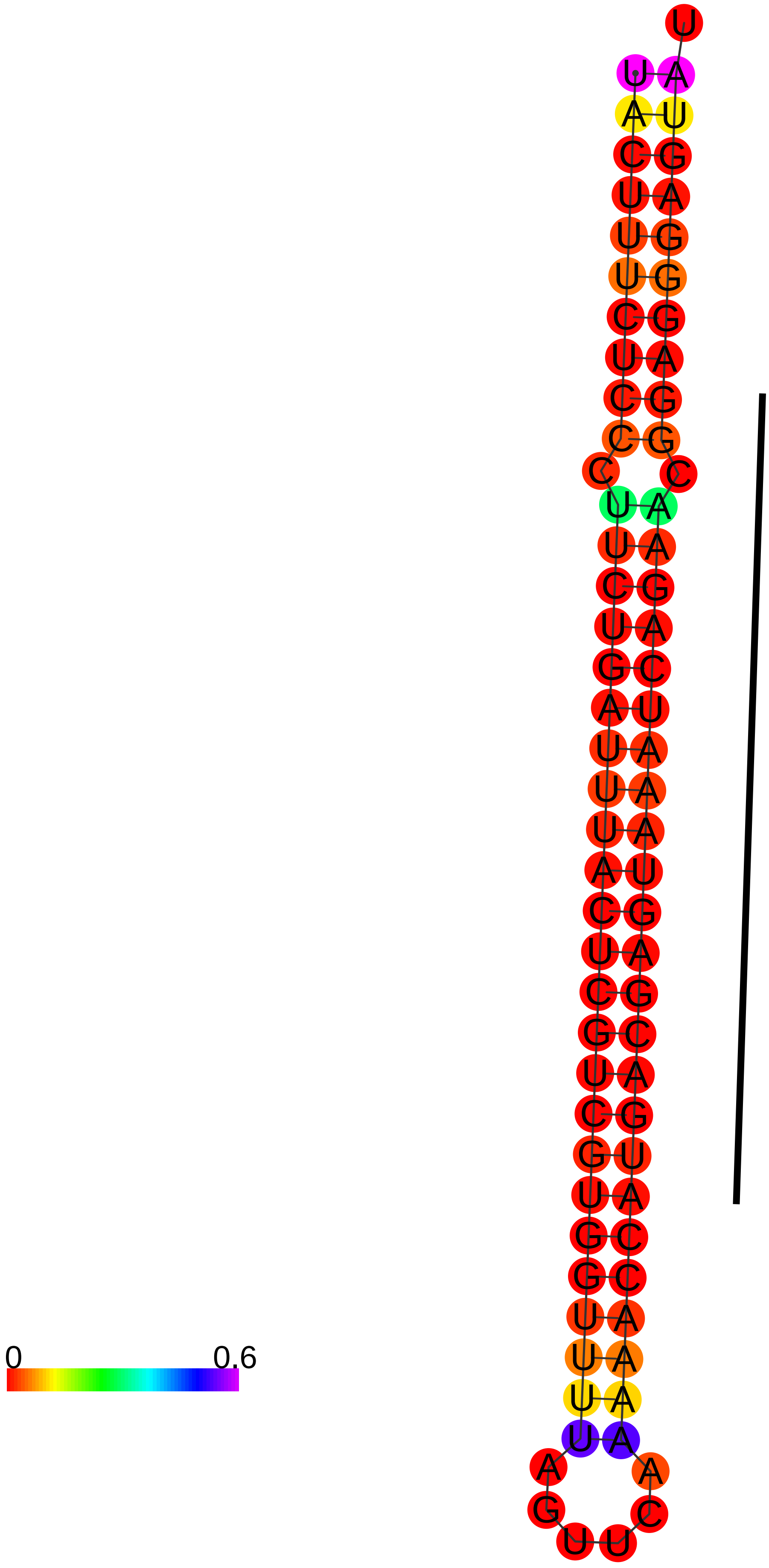

MITE_miRNA_35

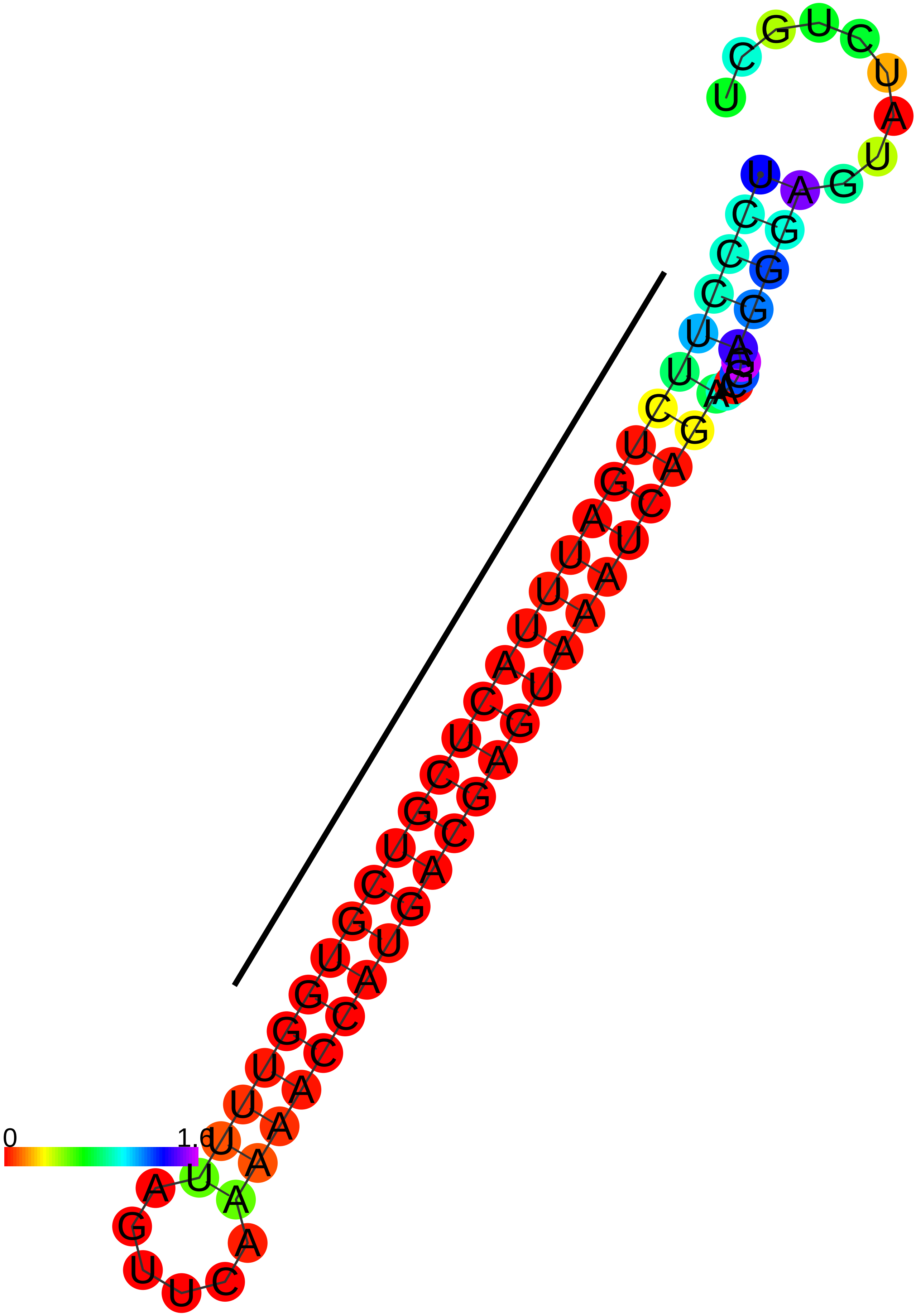

MITE_miRNA_36

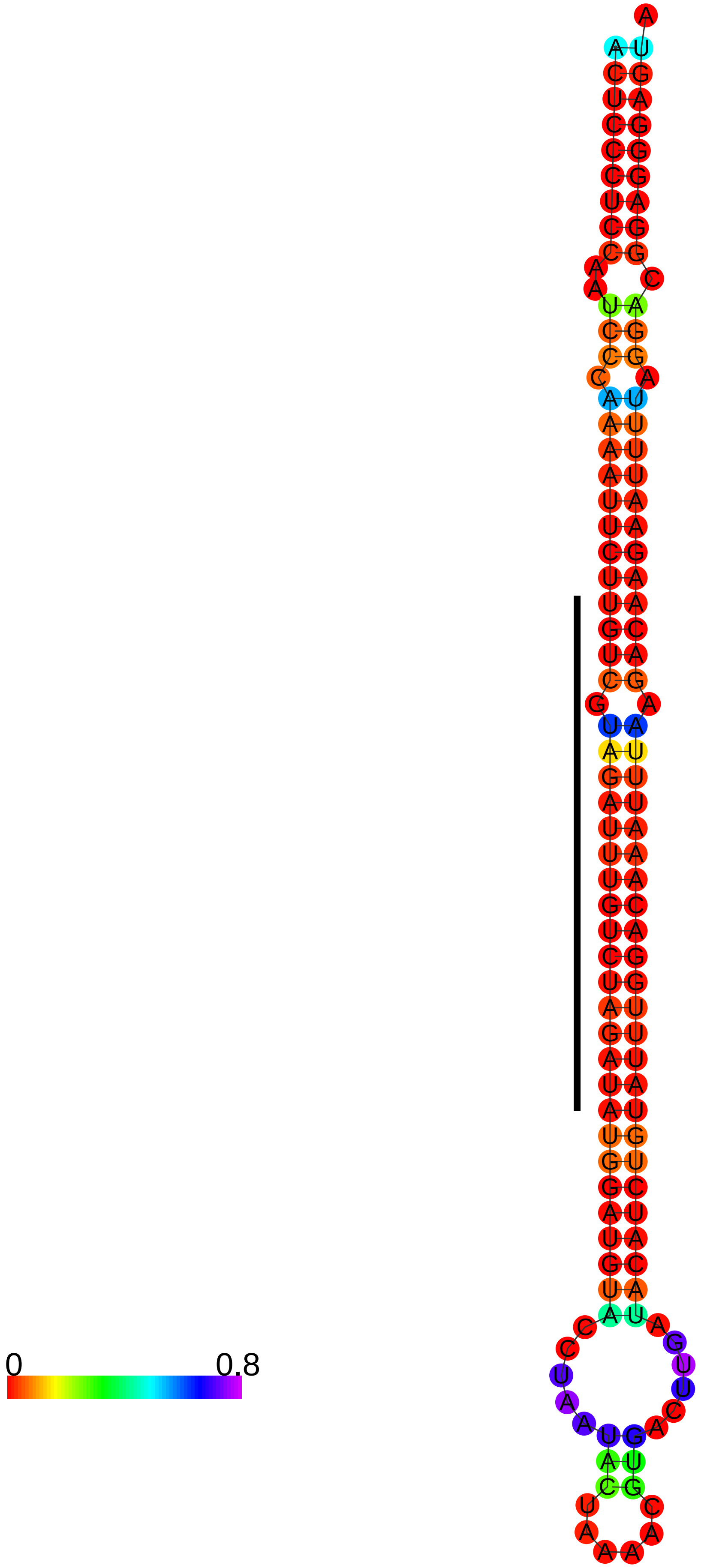

MITE_miRNA_37

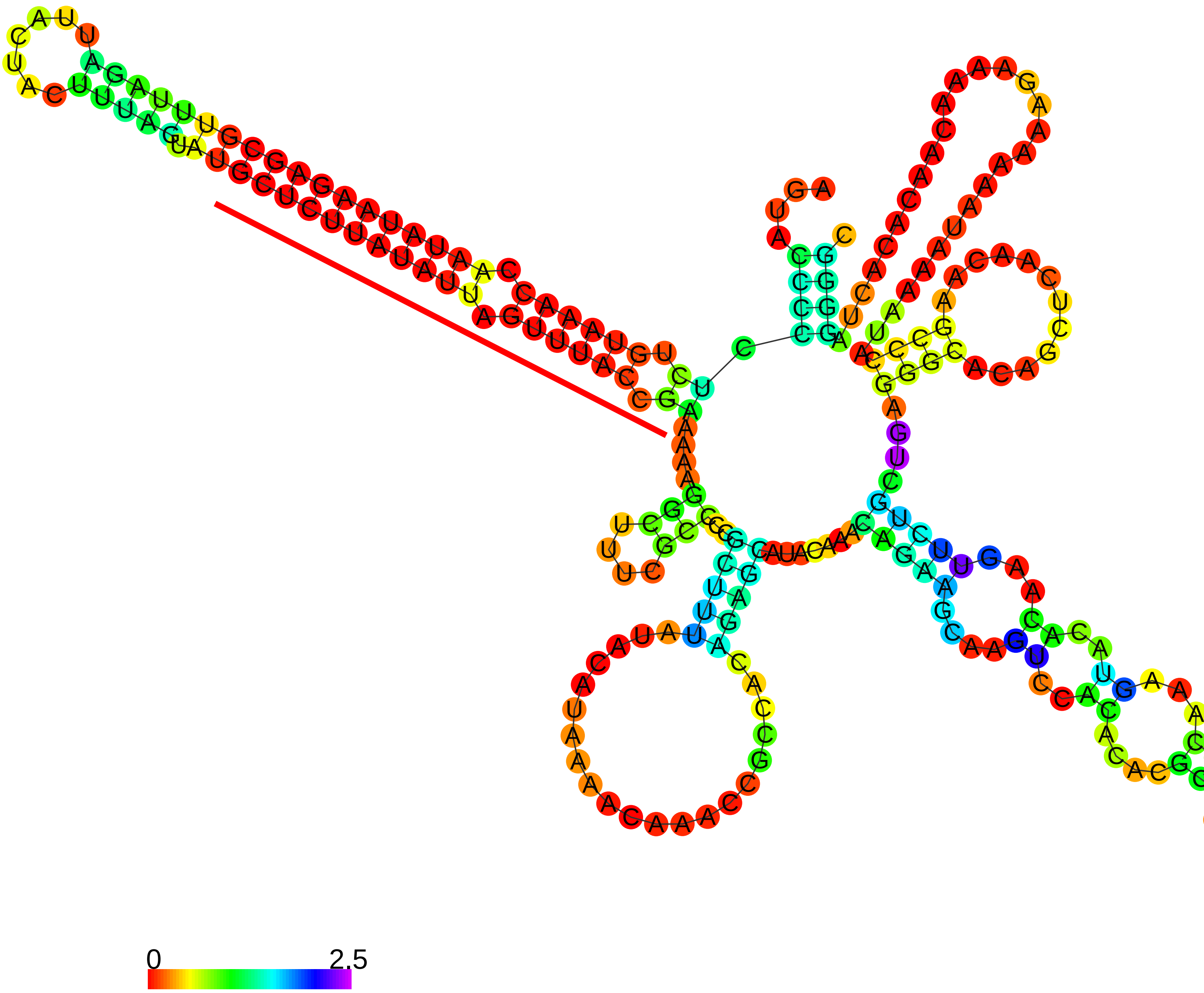

MITE_miRNA_38

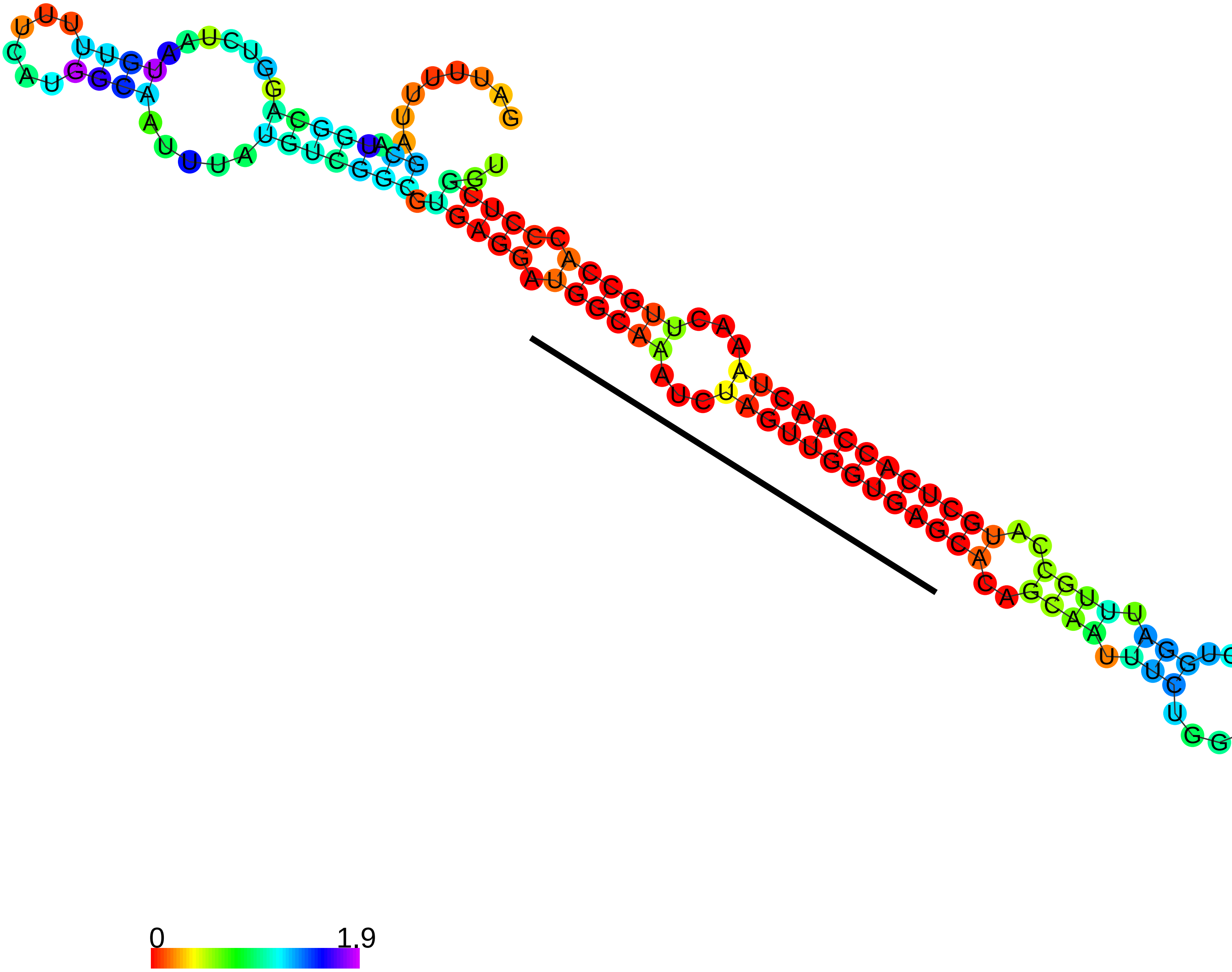

Supplement: Supplementary file 3 — Additional file 3 pre-miRNAs secondary structures predictions. [file 12864_2022_8364_MOESM3_ESM.pdf]
