# Supplementary material for: Genome-wide identification of MITE-derived microRNAs and their targets in bread wheat
Source: BMC Genomics. 2022 Feb 22;23:154. doi: 10.1186/s12864-022-08364-4 (PMC8862332; doi:10.1186/s12864-022-08364-4)
Supplement: Supplementary file 6 — Additional file 6 Figure S1: Length distribution of the sRNAs produced by the 38 MITE-derived miRNAs. Figure S2: Description of the MITE-derived miRNA 5 production site and an alignment of the target transcript TraesCS2A02G281000.2 region considering the wheat relatives for the A genome. Figure S3: Description of the MITE-derived miRNA 36 production site and an alignment of the target transcript TraesCS6A02G276700.1 region considering the wheat relatives for the A genome. Figure S4: Description of the MITE-derived miRNA 36 production site and an alignment of the target transcript TraesCS3A02G274100.1 region considering the wheat relatives for the A genome. Figure S5: Hairpin plots of the secondary structures of 4 examples of highly conserved miRNAs and 4 examples of MITE-derived miRNAs. [file 12864_2022_8364_MOESM6_ESM.pdf]

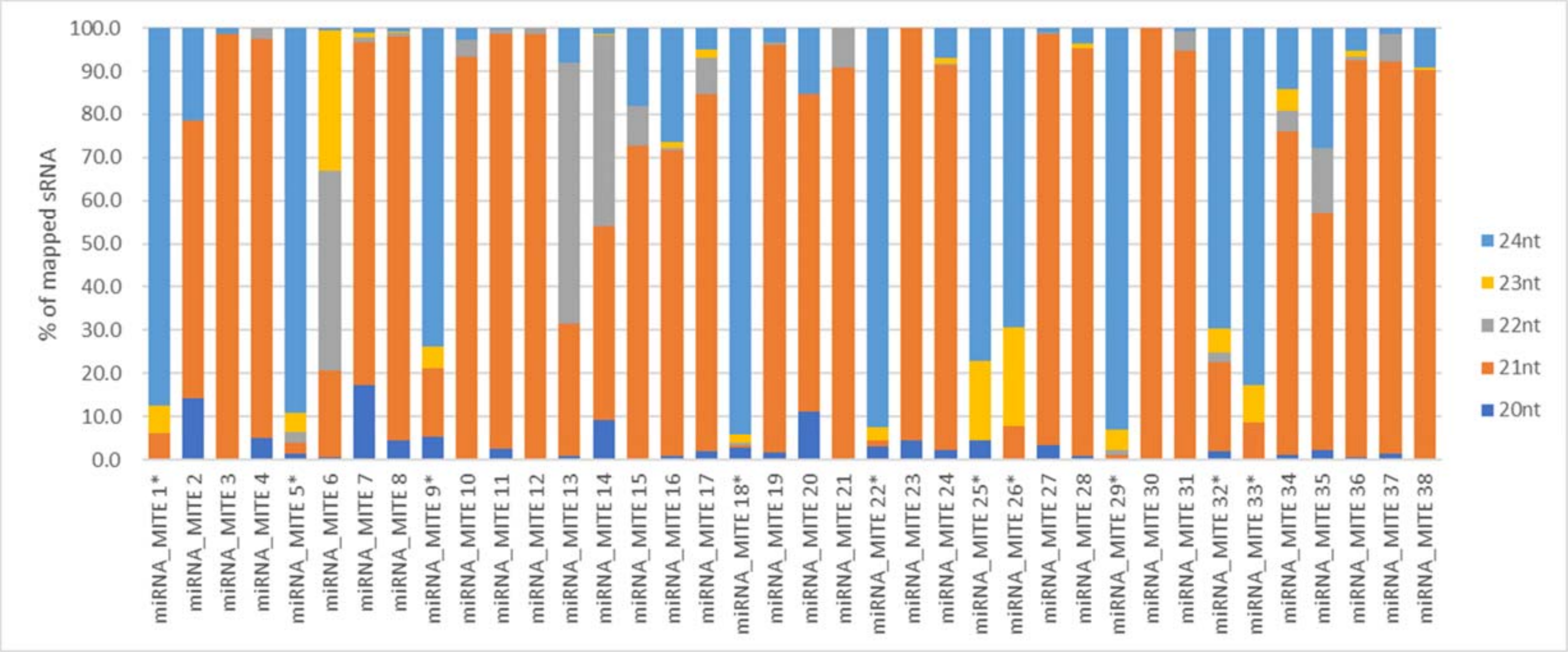

Figure S1: Length distribution of the sRNAs produced by the 38 miRNA_MITEs detected in this work. With * are highlighted the 10 miRNA_MITEs with a higher proportion of 24nt sRNA. The rest 28 miRNA_MITEs showed a higher proportion of 21nt sRNA

A)

miRNA_MITE 5 origin locus (TraesCS2A02G175300 promoter)

```
2A:133702671-133702770 ctcccttcgttccgaattacttgtcgcagatatggatgtatctagatgtattttagctctggatacattcatttcTGAGACGGGTAATTTGGAACGGAGg
                                                                                  miRNA_MITE 5  5′ TGAGACGGGTAATTTGGAACGGAG 3′
Icarus_BG607724-1      -tccctccgttccgaattacttgtcgcaggtatggatgtatctagatgtattttagttctagatacatccatttctgcgacgagtaatttggaacggaggga
                        *****.*********************.**************************.***.*******.******** ****.*****************
```

B)

Target transcript

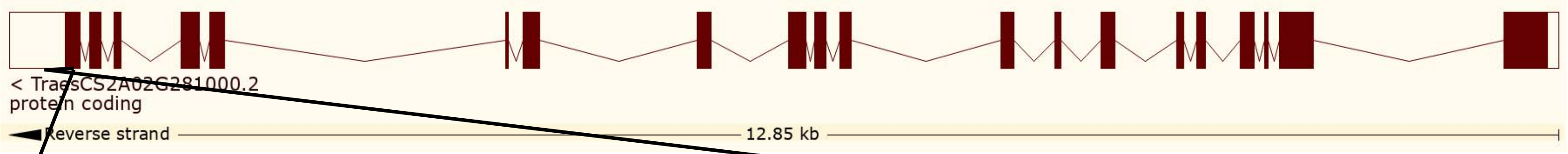

Exon 20 3′ UTR

```
TRITR3_27131       CTGGACTCAGCAATCGCAATCCTAGGGGTATCCTCCCTTAActtgtacagttgaaagaagcatcaaatgtacatgaacatgctcatgtggcatgttggtcggacccaggtgccaaaaaag
TRIDC2AG040650     CTGGACTCAGCAATCGCAATCCTAGGGGTATCCTCCCTTAActtgtacagttgaaagaagcatcaaatgtacatgaacatgctcatgtggcatgttggtcggacccaggtgccaaaaaag
TRITD2Av1G167480   CTGGACTCAGCAATCGCAATCCTAGGGGTATCCTCCCTTAActtgtacagttgaaagaagcatcaaatgtacatgaacatgctcatgtggcatgttggtcggacccaggtgccaaaaaag
TraesCS2A02G281000 CTGGACTCAGCAATCGCAATCCTAGGGGTATCCTCCCTTAActtgtacagttgaaagaagcatcaaatgtacatgaacatgctcatgtggcatgttggtcggacccaggtgccaaaaaag
TraesCS2B02G298400 CTGGACTCAGCAATCGCAATCCTAGGGGT-TCCTCCCTTAActtgtacagttgaaagaaacatcaaatgtacatgaacatgctcatgtggcatgttggtcggacccaggtgccaaaaaag
TraesCS2D02G279900 CTGGACTCAGCAATCGCAATCCTAGGGGTATCCTCCCTTAActtgtacagttgaaagaagcatcaaatgtacatgaacatgcttatgtggcatgttcgtcgga-ccaggtg-cgaaaaag
                   ***************************** *****************************.***********************.************ ****** ******* *.******

TRITR3_27131       ggcacctcttcatttggttttgatccaatcctcaatcattttggagcTACTCCCTCCGTTCCGAATTACTTGTCTTGGATTTGTCTAGATACTGATGTGTCTAGACTAATTTTAGTGCTA
TRIDC2AG040650     ggcacctcttcatttggttttgatccaatcctcaatcattttggagcTACTCCCTCCGTTCCGAATTACTTGTCTTGGATTTGTCTAGATACGGATGTATCTAGACTAATTTTAGTGCTA
TRITD2Av1G167480   ggcacttcttcatttggttttgatccaatcctcaatcattttggagcTACTCCCTCCGTTCCGAATTACTTGTCTTGGATTTGTCTAGATACGAATGTATCTAGACTAATTTTAGTGCTA
TraesCS2A02G281000 ggcacctcttcatttggttttgatccaatcctcaatcattttggagcTACTCCCTCCGTTCCGAATTACTTGTCTTGGATTTGTCTAGATACGGATGTA--TAGACTAATTTTAGTGCTA
Pan_M801L24-1                                                    GTACTCCCTCTGTCCCAAAATTCTTGTCTTAGATTTGTCTACATACGGATGTATCTATTCACATTTTAGTGTTA
miRNA_MITE 5                                                         3′ GAGGCAAGGTTTAATGGGCAGATG 5′
TraesCS2B02G298400 ggcacctcttcacttggttttgattcaatcctcaatcattttggagc--------------------------------------------------------------------------
TraesCS2D02G279900 ggcacctcttcacttggttttgatccaatcctcaaacattttgagc--------------------------------------------------------------------------
                   *****.******.***********.********** ****** ****

TRITR3_27131       GATACCTGTGTATCTAGACAAATCTAAGACGAAGTAATTCGGAACGGAGGGAGTActtgtttgagttttgagctgagcatccaaattttgttctacaaaggcagttctttgtaatcggaa
TRIDC2AG040650     GATACCTGTGTATCTAGACAAATCTAAGAC-AAGTAATTCGGAACGGAGGGAGTActtgtttgagttttgagctgagcatccaaattttgttctacaaaggcagttctttgtaatcggaa
TRITD2Av1G167480   GATACCTGTGTATCTAGACAAATCTAAGAC-AAGTAATTCGGAACGGAGGGAGTActtgtttgagttttgagctgagcatccaaattttgttctacaaaggcagttctttgtaatcggaa
TraesCS2A02G281000 GATACCTGTGTATCTAGACAAATCTAAGAC-AAGTAATTCGGAACGGAGGGAGTActtgtttgagttttgagctgagcatccaaattttgttctacaaaggcagttctttgtaatcggaa
Pan_M801L24-1      GATACATCCGTATCTAGATAAATCTAAGAC-AAGAATTTTGGGAA-GAGGGAGTAC
TraesCS2B02G298400 -----------------------------------------------------tacttgtttgagttttgagctgggcatccaaattttgttctacaaaggcatttctttgtaatcggaa
TraesCS2D02G279900 -----------------------------------------------------tacttgtttgagttttgagctgggcatccaaattttgttctacaaaggcatttctttgtaatcggaa
                                                                        **********************.************************** ****************
```

Figure S2: A) Description of the miRNA_MITE 5 origin *locus*. DNA sequence alignment of the TraesCS2A02G175300 gene promoter, the miRNA_MITE 5 and the MITE Icarus_BG607724-1. **B)** Visualization of the TraesCS2A02G281000.2 transcript (reverse strand), which is target of the miRNA_MITE 5 in the 3' UTR. Alignment of the transcript target region in *T. Urartu* (TRITR3_27131), *T. dicoccoides* A genome (TRIDC2AG040650), *T. durum* A genome (TRITD2Av1G167480) and the B (TraesCS2B02G298400) and D (TraesCS2D02G279900) homeologues in *T. aestivum*. The stop codon of the transcript is highlighted in red, in yellow is highlighted the region correspond to the insertion of MITE Pan_M801L24-1 and in green the TSD (TA). The complementary alignment of the miRNA_MITE 5 in the 3' UTR target region is also shown.

A)

miRNA_MITE 36 origin locus (intergenic)

```
7B:145795106:145795346:-1 gctctgtggcggtttgttttatgtataaagcggggcgaaagcctttTCGGTAAACTAATATAAGAGCat-------actaaagtagtaatctaaacgctcttatattggtttacagagg
miRNA_MITE 36                                                        5'  TCGGTAAACTAATATAAGAGC  3'
Athos_103H9-1           _ --------------------------------------------ccctctgtaaactaatataagagcgtttagatcactattttagtattctaaacgctcttatattagtttacggagg
                                                                    ...** ******************.*       ****    ***** ******************.******.****
```

B)

Target transcript

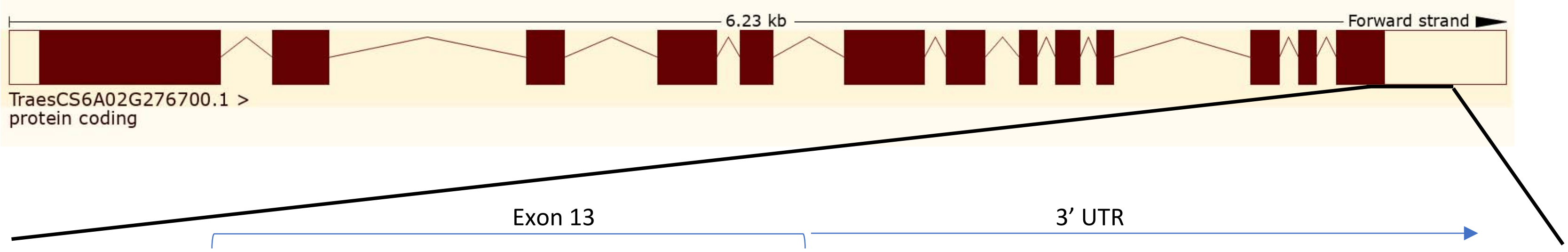


```
TRIUR3_19471       TGCCCCTGAGAAGAATAACGATTCGGAACCAGAATCCCATGACGAGTTGTGAttattagaatcggcttttgaacacccctgagagatactactc--aatatact-ccgagaagacagtt
TRIDC6AG042320     TGCCCCTGAGAAGAATAACGATTCGGAACCAGAATCCCATGACGAGTTGTGAttattagaatcggcttttgaacacccctgagagatactactc--aatatact-ccgagaagatagtt
TRITD6Av1G17400    TGCCCCTGAGAAGAATAACGATTCGGAACCAGAATCCCATGACGAGTTGTGAttattagaatcggcttttgaacacccctgagagatactactc--aatatact-ccgagaagatagtt
TraesCS6A02G276700 TGCCCCTGAGAAGAATAACGATTCGGAACCAGAATCCCATGACGAGTTGTGAttattagaatcggcttttgaacacccctgagagatactactc--aatatact-ccgagaagatagtt
TraesCS6B02G304200 TGCATCGGAAAAGACTAACGATTCGGAACCGGAATCCCATGACGAATTGTGAttattagaattggcttttgaacaccccgagagatactactc--aatatactcccgagaagatagtt
TraesCS6D02G257000 TGCCCCTGAGAAGAATAACGATTCGGAACCAGAATCCCATGACGAGTTGTGAttattagaatcggcttttgaacacccctgagagatactactcaaaatatact-ccgagaagatagct
                   ***  .* **.**** ***************.*************.****************.***************.**************  ******** *********.**.*

TRIUR3_19471       tgtcaccaccacagttttgttatggcggccgaaaagac----aggtttgtgtaggaattcacggcaggctagatgctatatgggcgagaagaatttgatgtcaatcgctgtatagcagt
TRIDC6AG042320     tgtcaccaccacagttttgttatggcggccgaaaagaca--gaggtttgtgtaggaattcacggcaggctagatgctatatgggcgagaagaatttgatgtcaatcgctgtatagcagt
TRITD6Av1G17400    tgtcaccaccacagttttgttatggcggccgaaaagaca--gaggtttgtgtaggaattcacggcaggctagatgctatatgggcgagaagaatttgatgtcaatcgctgtatagcagt
TraesCS6A02G276700 tgtcaccaccacagttttgttatggcggccgaaaagaca--gaggtttgtgtaggaattcacggcaggctagatgctatatgggcgagaagaatttgatgtcaatcgctgtatagcagt
TraesCS6B02G304200 tgtcaccaccacagttgtgtt-tggcggctgaaaagacagagaggtttgtgtaggaattcacggcaggctagatgctatatgggcgagaagaatttgatatcaatcgctgtatagcagt
TraesCS6D02G257000 cgtcaccaccacagttttgtt-tggcggccgaaaagacagagaggtttgtgtaggaattcacggcaggctagatgctatacgagcgagaagaatttgatattgatcgctgtatagcaat
                   .*************** **** *******.********    *************************************.*.***************.*..*************.*

TRIUR3_19471       attgtggtctctagggcatatgctgtcaagatagcgcctcagaaaggcttagttgtcaggactgagatgactttgtagctgcattgttgtact-gaaactcatttatgttttgataaca
TRIDC6AG042320     attgtggtctctagggcatatgctgtcaagatagcgcctcagaaaggcttagttgtcaggactgagatgactttgtagctgcattgttgtact-gaaactcatttatgttttgataaca
TRITD6Av1G17400    attgtggtctctagggcatatgctgtcaagatagcgcctcagaaaggcttagttgtcaggactgagatgactttgtagctgcattgttgtact-gaaactcatttatgttttgataaca
TraesCS6A02G276700 attgtggtctctagggcatatgctgtcaagatagcgcctcagaaaggcttagttgtcaggactgagatgactttgtagctgcattgttgtact-gaaactcatttatgttttgataaca
TraesCS6B02G304200 attgtggactctagggcatatgctgtcaagatagcgcctc-gaaaggcttagttgtcaggactgagaagactttgtagctgcattgttgtactggaaactcatttatattttgataaca
TraesCS6D02G257000 attgtggtctctagggcatatgctgtcgagatagcggctc-gaaaggcttagttgtcaggactgagaagactttgtagctgcattgttgtact-gaaactcatttatattttgataaca
                   ******* ******************.******** *** ************************* ************************ *************.**********

TRIUR3_19471       gc-----------------------------------------------------------------------------------tactatagagattttatgtctggtaggggtatcg
TRIDC6AG042320     gc-----------------------------------------------------------------------------------tactatagagattttatgtctggtaggggtatcg
TRITD6Av1G17400    gcTACTCCCTCTGTAAACTAATATAAGAGCATTTAGATCACTAACTTAGTAATCTAAACGCTCTTATATTAGTTTACGGAGGGAGTActatagagattttatgtctggtaggggtatcg
TraesCS6A02G276700 gcTACTCCCTCTGTAAACTAATATAAGAGCATTTAGATCACTAACTTAGTAATCTAAACGCTCTTATATTAGTTTACGGAGGGAGTActatagagattttatgtctggtaggggtatcg
Athos_AF326781-1        TCCCTCCGTAAACTAATATAAGAGCGTTTAGAATACTAAAGTAGTGATCTAAATGTTCTTATATTAGTTTACAGAGGGA
miRNA_MITE 36                                                           3' CGAGAATATAATCAAATGGCT 5'
TraesCS6B02G304200 gc-----------------------------------------------------------------------------------tactatagagattttatgtttggta------tcg
TraesCS6D02G257000 gc-----------------------------------------------------------------------------------tactatagagattttatgcctggtaggggtttca
                   **                                                                                   *****************..*****      **.
```

```
TRIUR3_19471       gtgtatctgtttgctctttcatactgtcaatcacgcagtgctg-ttaacttgcagttactataa
TRIDC6AG042320     gtgtatctgtttgctctttcatactgtcaatcacgcagtgctg-ttaacttgcagttactataa
TRITD6Av1G17400    gtgtatctgtttgctctttcatactgtcaatcacgcagtgctg-ttaacttgcagttactataa
TraesCS6A02G276700 gtgtatctgtttgctctttcatactgtcaatcacgcagtgctg-ttaacttgcagttactataa
TraesCS6B02G304200 atgtatctgtttgctctttcatactgtctatcacgcagtgccg-ctaacttgcagt-----taa
TraesCS6D02G257000 atgtatctgtttgctcttttatactgtctatcacgcagtgctgtttaacttgcagt-----taa
                   .******************.******** ************.* .***********     ***
```

Figure S3: A) Description of the miRNA_MITE 36 origin *locus*. DNA sequence alignment of the intergenic region in chromosome 7B (145795106-145795346), the miRNA_MITE 36 and the MITE Athos_103H9-1. **B)** Visualization of the TraesCS6A02G276700.1 transcript (foward strand), which is target of miRNA_MITE 36 in the 3’ UTR. Alignment of the transcript target region in *T. Urartu* (TRIUR3_19471), *T. dicoccoides* A genome (TRIDC6AG042320), *T. durum* A genome (TRITD6Av1G17400) and the B (TraesCS6B02G304200) and D (TraesCS6D02G257000) homeologues in *T. aestivum*. The stop codon of the transcript is highlighted in red, in yellow highlighted the region correspond to the insertion of MITE Athos_AF326781-1 and in green the TSD (TA). The complementary alignment of the miRNA_MITE 36 in the 3’ UTR target region is also shown.

A)

miRNA_MITE 36 origin locus (intergenic)

```
7B:145795106:145795346:-1 gctctgtggcggtttgttttatgtataaagcggggcgaaagccttttTCGGTAAACTAATATAAGAGCat-------actaaagtagtaatctaaacgctcttatattggtttacagagg
miRNA_MITE 36                                                       5’   TCGGTAAACTAATATAAGAGC  3’
Athos_103H9-1           _ --------------------------------------------ccctctgtaaactaatataagagcgtttagatcactattttagtattctaaacgctcttatattagtttacggagg
                                                                      ...** ******************.*       ****    ***** *****************.******.****
```

B)

Target transcript

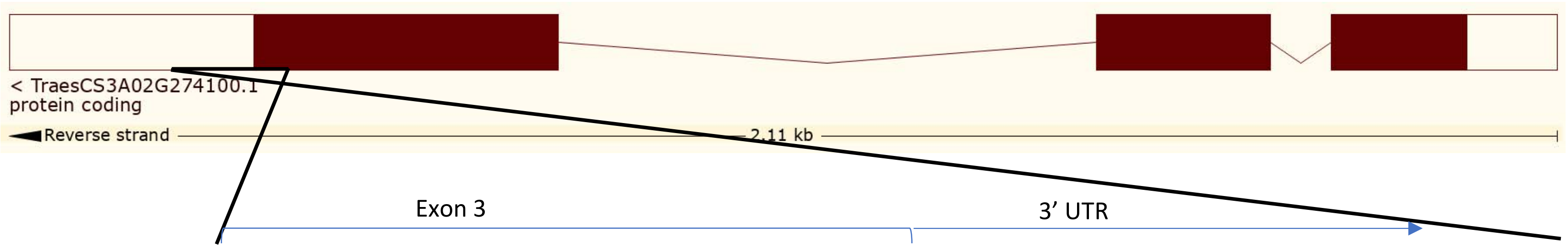


```
TRIUR3_21627       AAGTTCAGGGCGTCCATGCTGAGCTTCGACTTCGGCCGGGCCCCGGTGGTGGTCGCGTGAcgcgtgcatgg--------cgtcaagaggggtatgatgccatggtgtcacgtgtatg
TRIDC3AG040720     AAGTTCAGGGCGTCCATGCTGAGCTTCGACTTCGGCCGGGCCCCGGTGGTGGTCGCGTGAcgcgtgcatgg--------cgtcaagaggggtatgatgccatggtgtcacgtgtatg
TRITD3Av1G181850   AAGTTCAGGGCGTCCATGCTGAGCTTCGACTTCGGCCGGGCCCCGGTGGTGGTCGCGTGAcgcgtgcatgg--------cgtcaagaggggtatgatgccatggtgtcacgtgtatg
TraesCS3A02G274100 AAGTTCAGGGCGTCCATGCTGAGCTTCGACTTCGGCCGGGCCCCGGTGGTGGTCGCGTGAcgcgtgcatgg--------cgtcaagaggggtatgatgccatggtgtcacgtgtatg
TraesCS3B02G307800 AAGTATAGGGCGTCCATGCTGAGCTTCGACTTCGGCCGGGCCCCGGTGGTGGTTGCGTGAtgcgtgcatgg----cggacaacaggaggagtatgatgccatggtgtcacgtgtatg
TraesCS3D02G273300 AAGTTCAGGGCGTCCATGCTGAGCTTCGACTTCGGCCGGGCCCCGGTGGTGGTTGCGTGAtgcgtgcatggcagacagacaacaagagaaatatgatgccatggtgtcactgtcacg
                   ****  .*********************************************.******.**********        *. **.***...*****************   .*.*

TRIUR3_21627       tgcccatgttattatagaccgtatgccgtatgccatgccgggcactgtagaaggccatagtagta----------------------------------------------------
TRIDC3AG040720     tgcccatgttattatagaccgtatgccgtatgccatgccgggcactgtagaaggccatagtagta----------------------------------------------------
TRITD3Av1G181850   tgcccatgttattatagaccgtatgccgtatgccatgccgggcactgtagaaggccatagtagta----------------------------------------------------
TraesCS3A02G274100 tgcccatgttattatagaccgtatgccgtatgccatgccgggcactgtagaaggccatagtagTACTTCCTCCGTAAACTAATATAAGAGTGTTTAGATTACTAAAATAGTGATCTA
Athos_103H9-1                                                                         CCCTCTGTAAACTAATATAAGAGCGTTTAGATCACTATTTTAGTATTCTA
TraesCS3B02G307800 tacccatgtt----------atagaccgtatgccatgccgggcactgtagtaggctagagtagta----------------------------------------------------
TraesCS3D02G273300 t-------------------gtatgccgtatgccatgccgggcgctctagtaggc--tagtagca----------------------------------------------------
                   *                   .** .*****************.** *** ****   ***** *

TRIUR3_21627       -------------------------------cttgttagtagagtgtgtaccggggattgtaggttgtgcatagtc
TRIDC3AG040720     -------------------------------cttgttagtagagtgtgtaccggggattgtaggttgtgcgtagtc
TRITD3Av1G181850   -------------------------------cttgttagtagagtgtgtaccggggattgtaggttgtgcgtagtc
TraesCS3A02G274100 AACGCTCTTATATTAGTTTACAGAGGGAGTActtgttagtagagtgtgtaccggggattgtaggttgtgcgtagtc
Athos_103H9-1      AACGCTCTTATATTAGTTTACGGAGGG
miRNA_MITE 36      3’ CGAGAATATAATCAAATGGCT 5’
TraesCS3B02G307800 -------------------------------------agtagagtgtgtaacggggattgtaggttgtacgtagtc
TraesCS3D02G273300 -------------------------------cttgttagtagagtgtgtaacggggattgtaggttgtgcgtagtc
                                                     ************* *****************.*.*****
```

Figure S4: A) Description of the miRNA_MITE 36 origin *locus*. DNA sequence alignment of the intergenic region in chromosome 7B (145795106-145795346), the miRNA_MITE 36 and the MITE Athos_103H9-1. **B)** Visualization of the TraesCS3A02G274100.1 transcript (reverse strand), which is the target of miRNA_MITE 36 in the 3' UTR. Alignment of the transcript target region in *T. Urartu* (TRIUR3_21627), *T. dicoccoides* A genome (TRIDC3AG040720), *T. durum* A genome (TRITD3Av1G181850) and the B (TraesCS3B02G307800) and D (TraesCS3D02G273300) homeologues in *T. aestivum*. The stop codon of the transcript is highlighted in red, in yellow highlighted the region correspond to the insertion of MITE Athos_103H9-1 and in green the TSD (TA). The complementary alignment of the miRNA_MITE 36 in the target region is also shown.

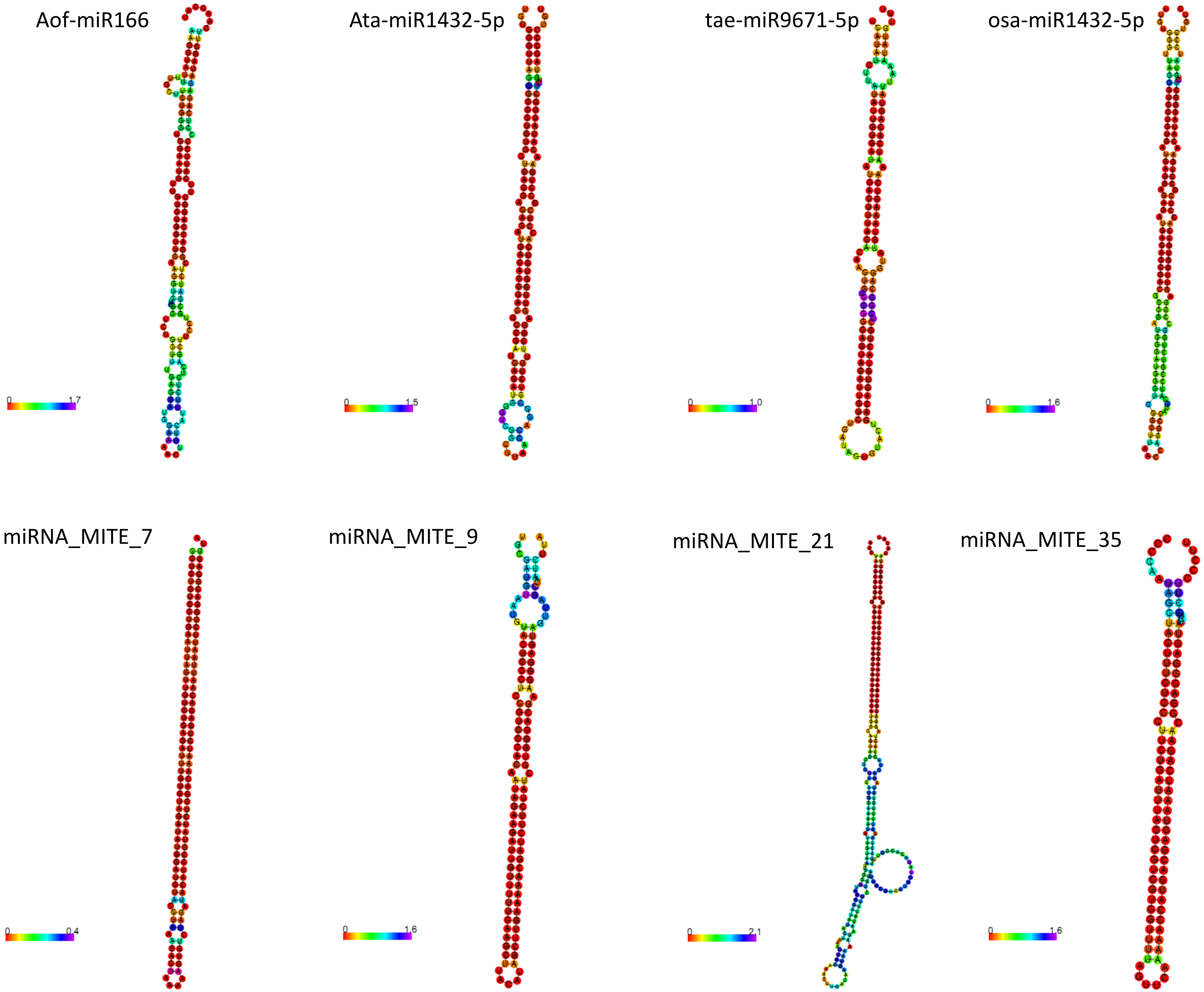

Aof-miR166
0
1.7
Ata-miR1432-5p
0
1.5
tae-miR9671-5p
0
1.0
osa-miR1432-5p
0
1.6
miRNA_MITE_7
0
0.4
miRNA_MITE_9
0
1.6
miRNA_MITE_21
0
2.1
miRNA_MITE_35
0
1.6

Figure S5: Hairpin plots of the secondary structures of 4 examples of highly conserved miRNAs and four examples of MITE-derived miRNAs.
