# Supplementary material for: Genome-wide identification of MITE-derived microRNAs and their targets in bread wheat
Source: BMC Genomics. 2022 Feb 22;23:154. doi: 10.1186/s12864-022-08364-4 (PMC8862332; doi:10.1186/s12864-022-08364-4)

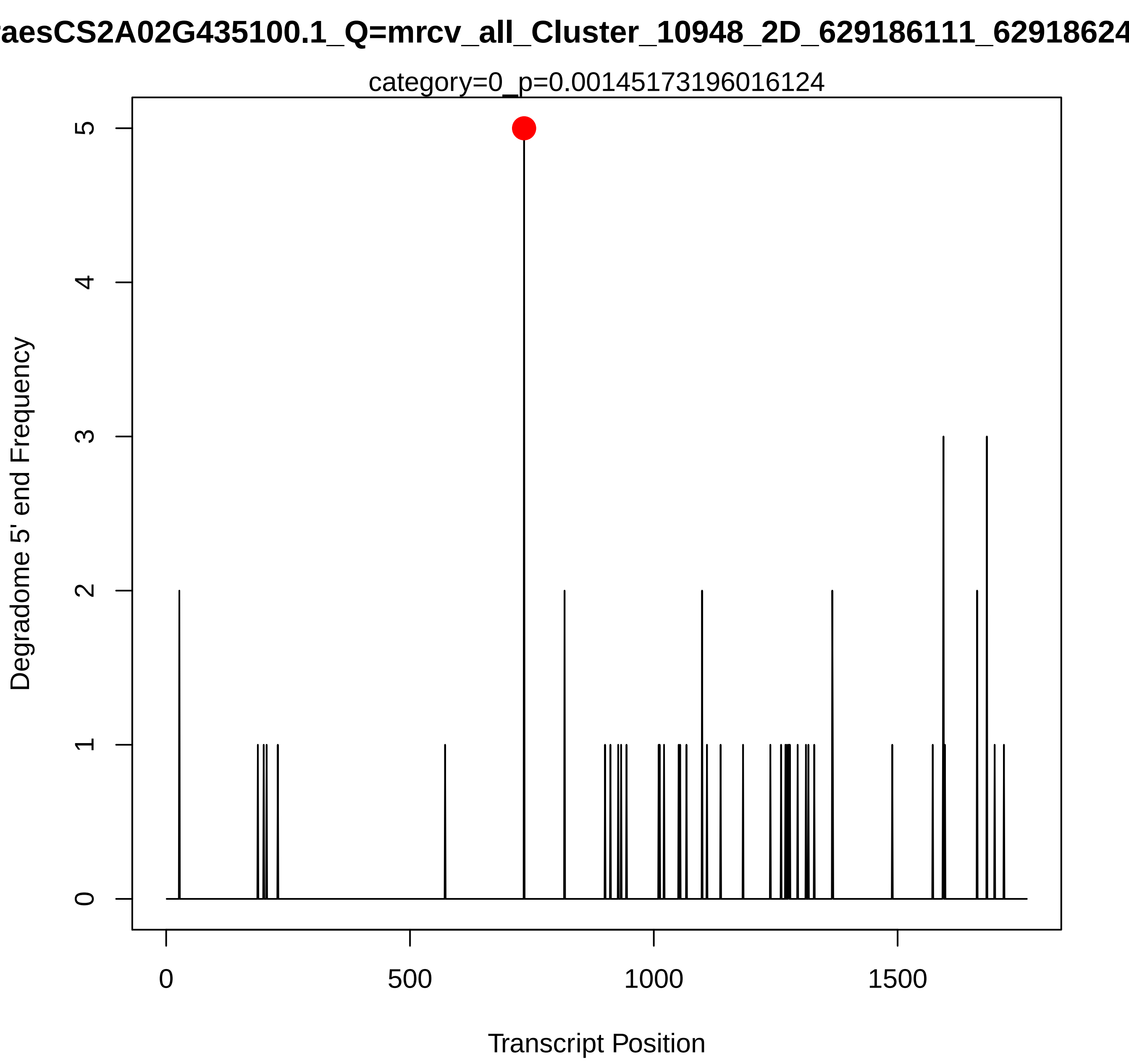

aesCS2A02G435100.1_Q=mrcv_all_Cluster_10948_2D_629186111_62918624
category=0_p=0.00145173196016124
Degradome 5' end Frequency
Transcript Position
0
1
2
3
4
5
0
500
1000
1500

[illegible]aesCS2D02G435200.1_Q=mrcv_all_Cluster_10948_2D_629186111_62918624[illegible]

category=3_p=0.00859209823651419

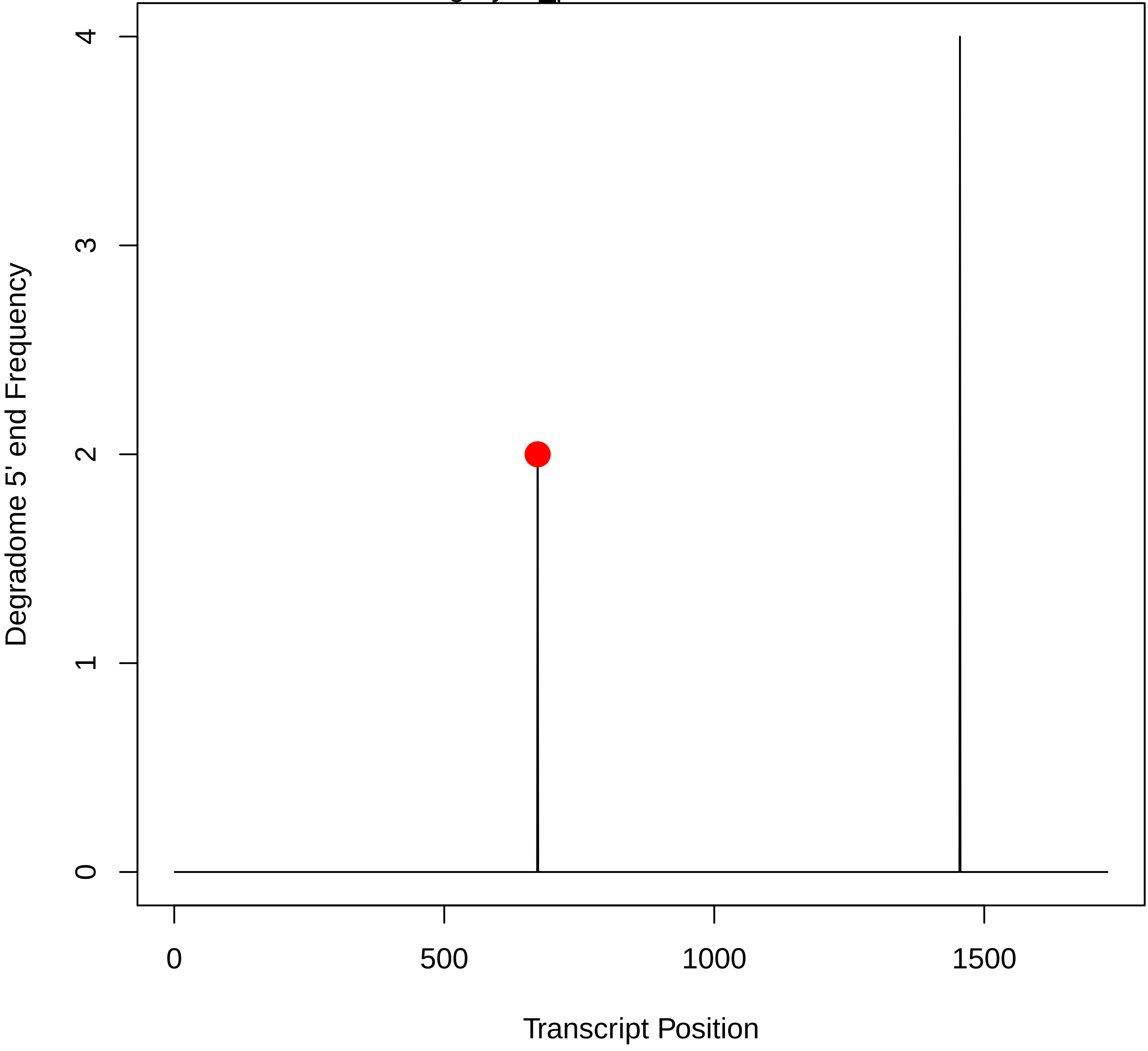

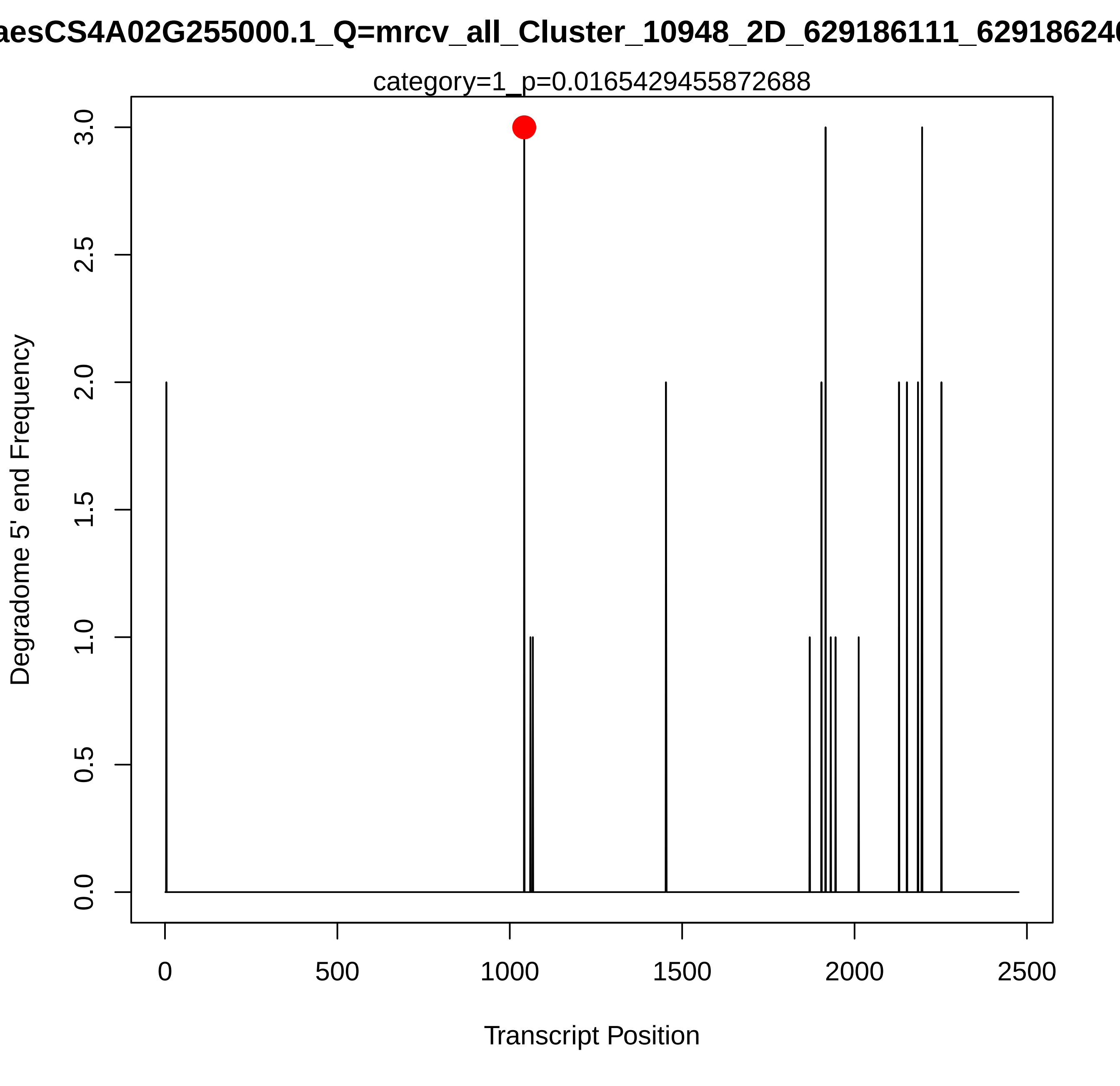

aesCS4A02G255000.1_Q=mrcv_all_Cluster_10948_2D_629186111_62918624
category=1_p=0.0165429455872688
Degradome 5' end Frequency
0.0
0.5
1.0
1.5
2.0
2.5
3.0
0
500
1000
1500
2000
2500
Transcript Position

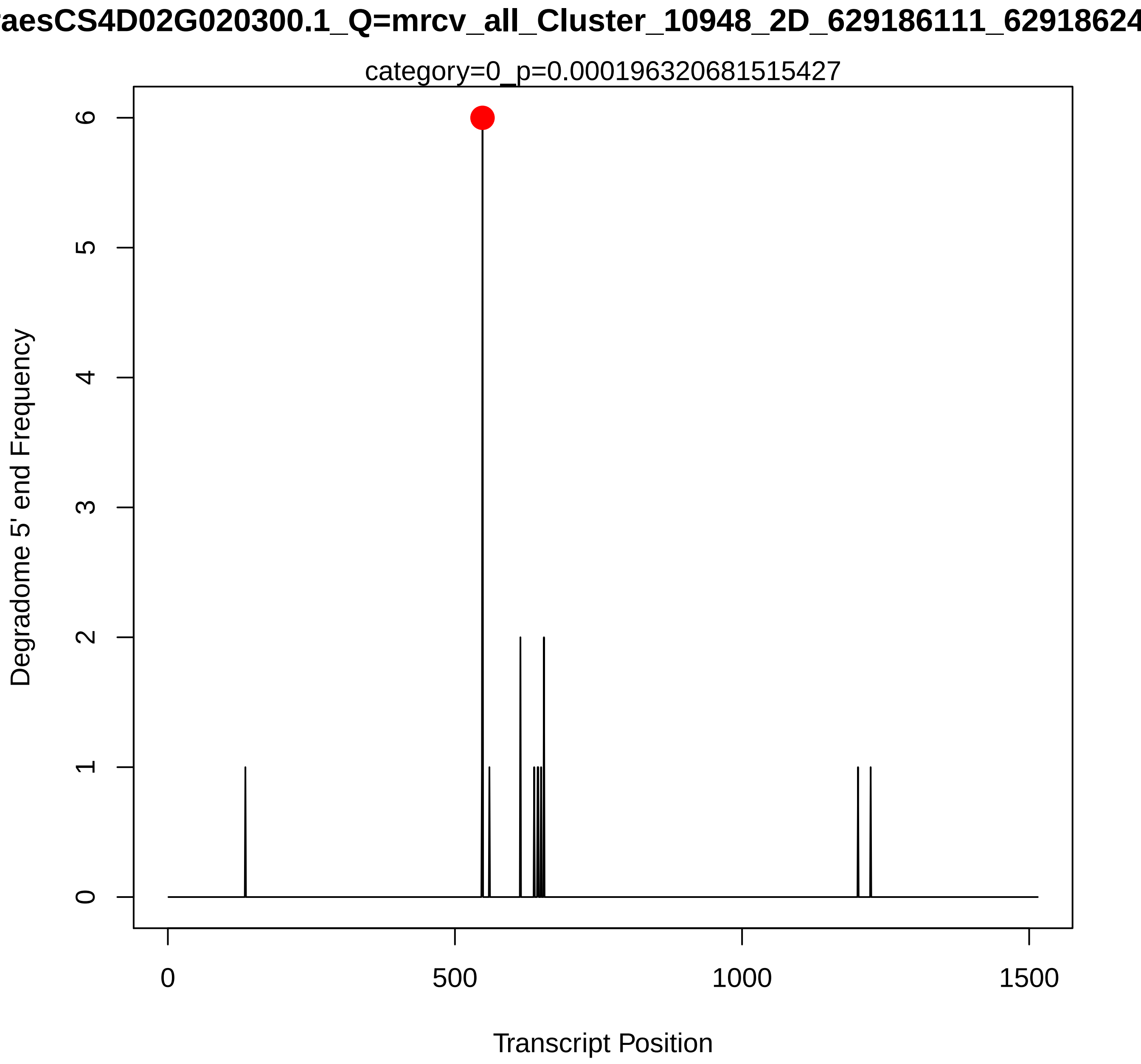
aesCS4D02G020300.1_Q=mrcv_all_Cluster_10948_2D_629186111_62918624
category=0_p=0.000196320681515427
Degradome 5' end Frequency
Transcript Position
0
1
2
3
4
5
6
0
500
1000
1500

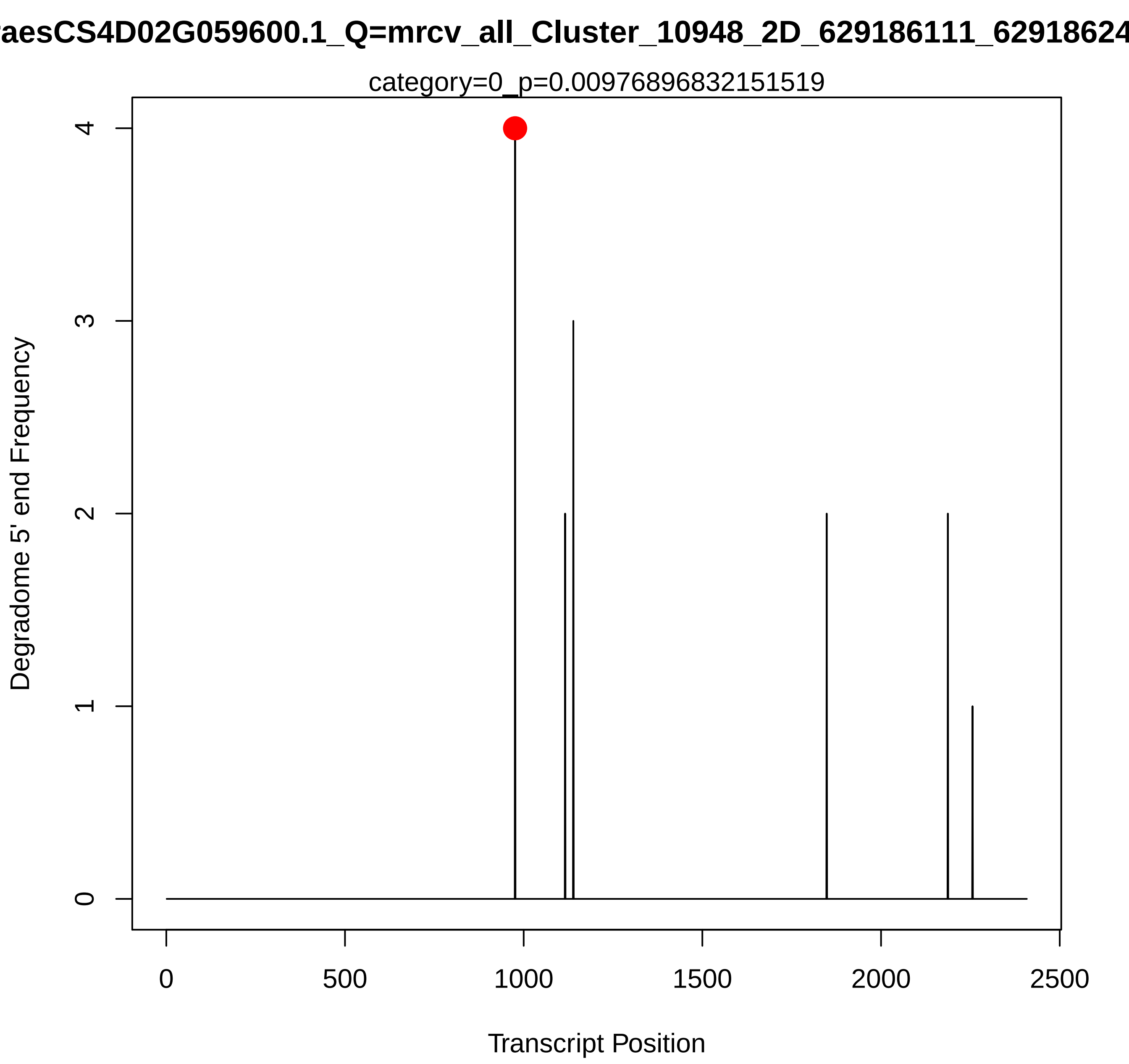
aesCS4D02G059600.1_Q=mrcv_all_Cluster_10948_2D_629186111_62918624
category=0_p=0.00976896832151519
Degradome 5' end Frequency
Transcript Position
0
1
2
3
4
0
500
1000
1500
2000
2500

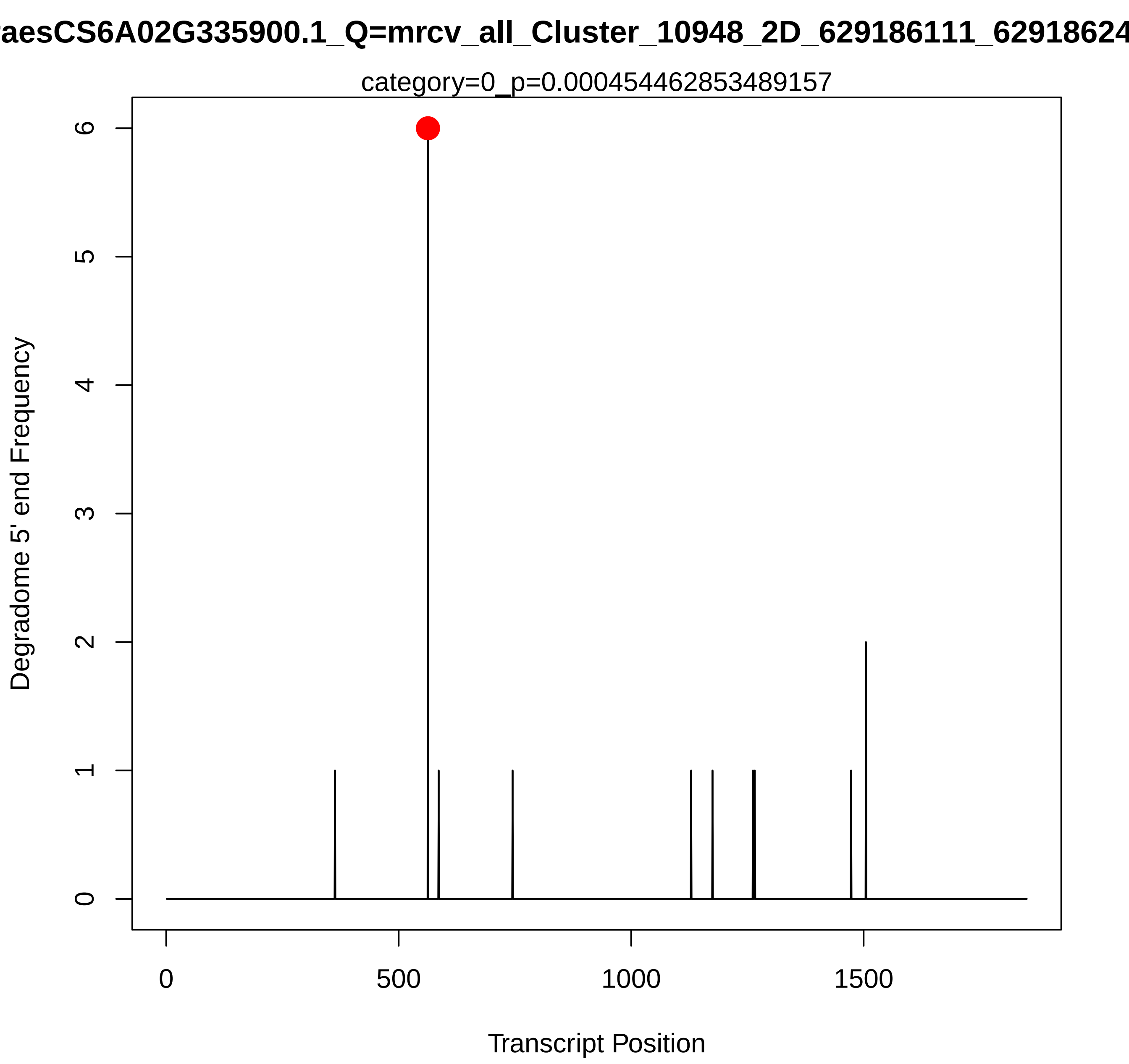
aesCS6A02G335900.1_Q=mrcv_all_Cluster_10948_2D_629186111_62918624
category=0_p=0.000454462853489157
Degradome 5' end Frequency
0
1
2
3
4
5
6
0
500
1000
1500
Transcript Position

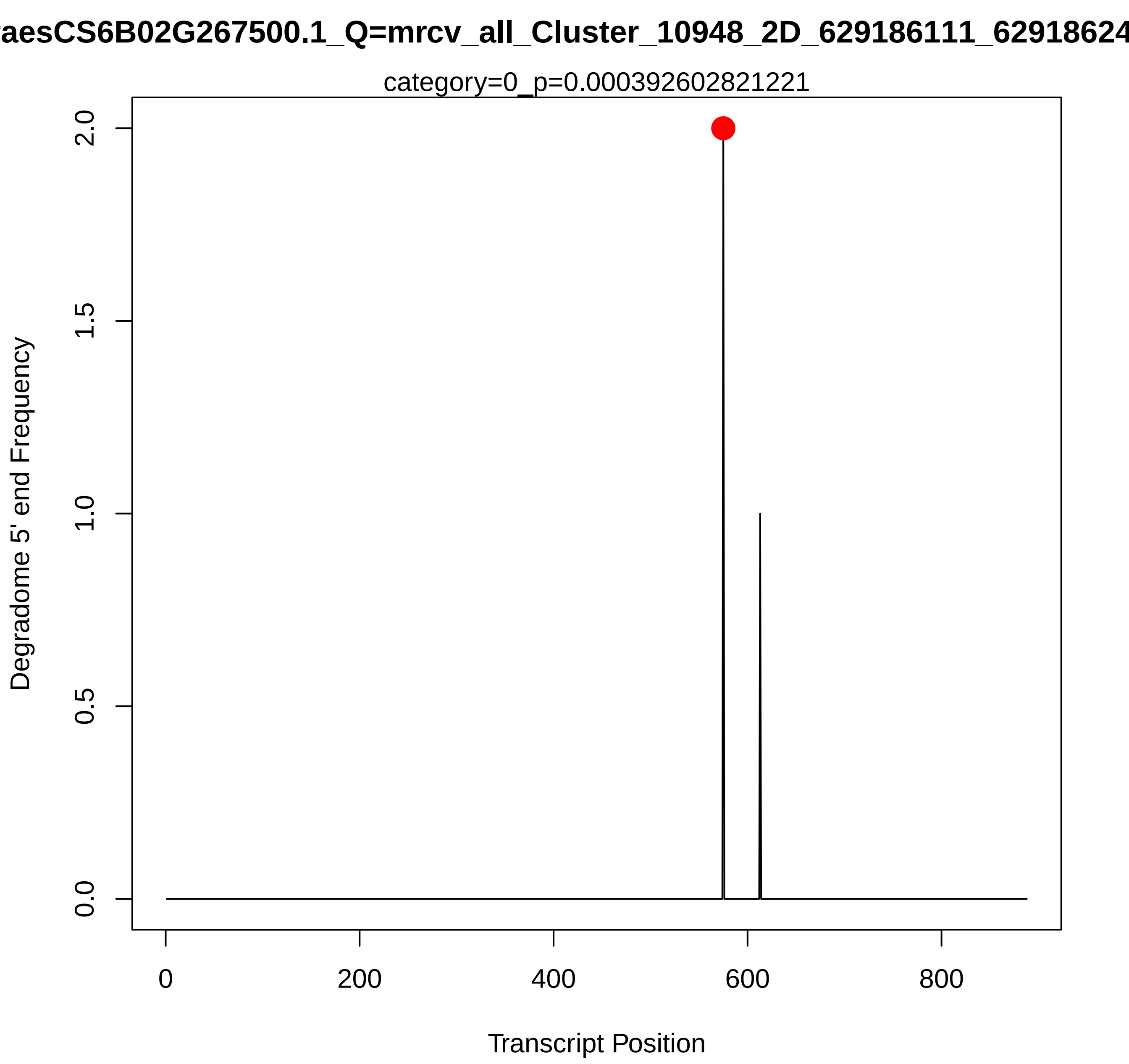
aesCS6B02G267500.1_Q=mrcv_all_Cluster_10948_2D_629186111_62918624
category=0_p=0.000392602821221
Degradome 5' end Frequency
0.0
0.5
1.0
1.5
2.0
0
200
400
600
800
Transcript Position

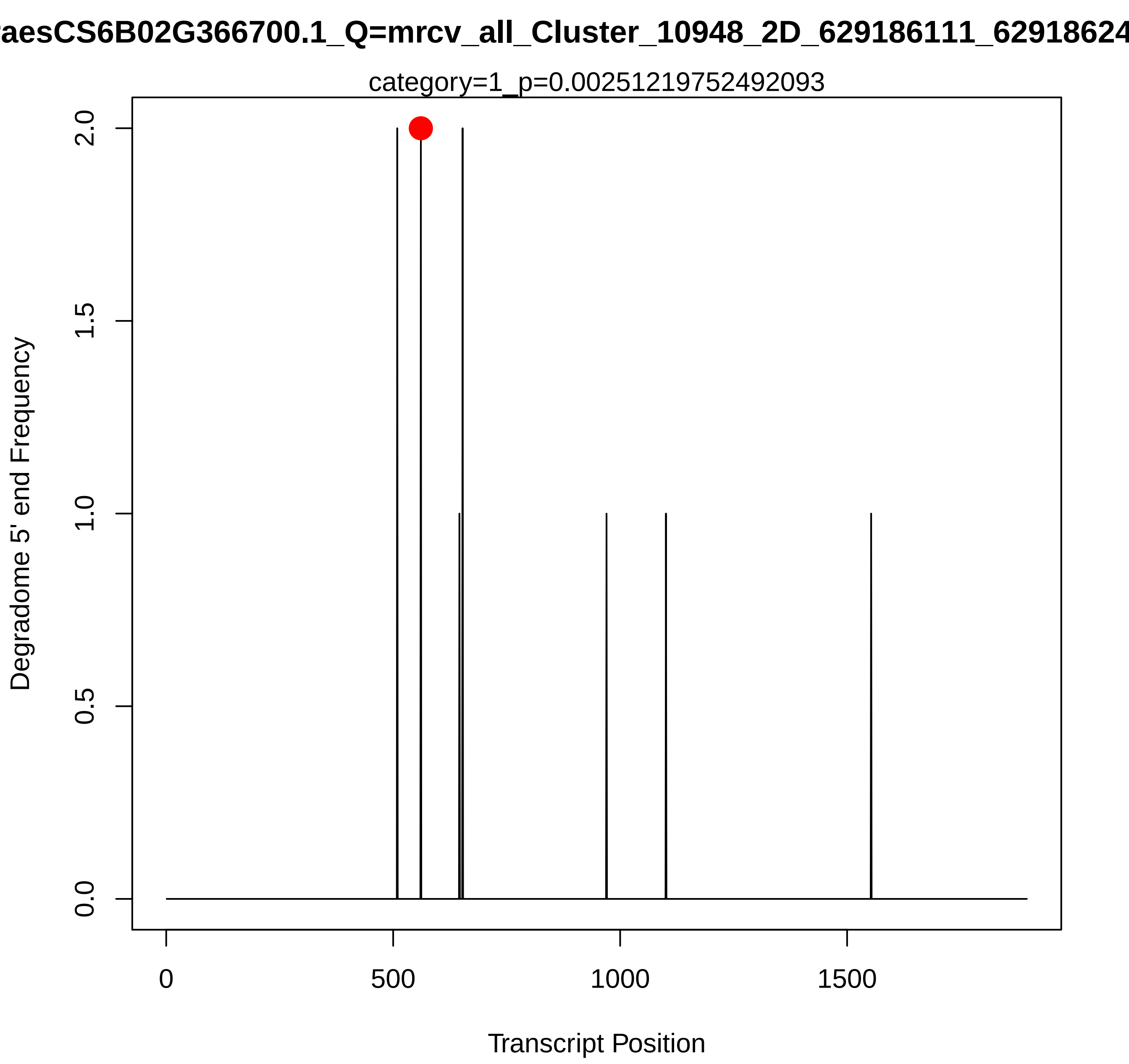

aesCS6B02G366700.1_Q=mrcv_all_Cluster_10948_2D_629186111_62918624
category=1_p=0.00251219752492093
Degradome 5' end Frequency
0.0
0.5
1.0
1.5
2.0
0
500
1000
1500
Transcript Position

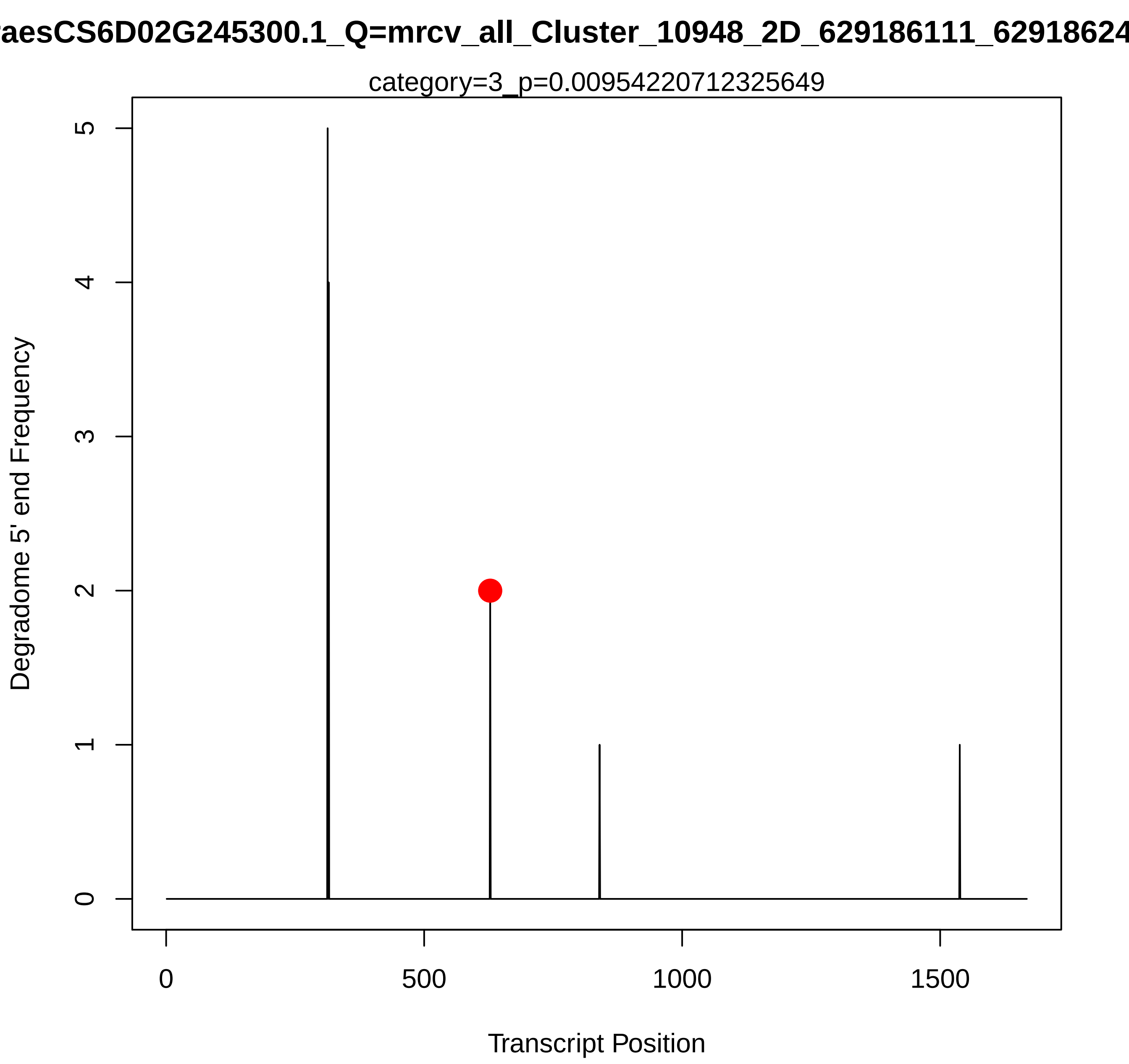

aesCS6D02G245300.1_Q=mrcv_all_Cluster_10948_2D_629186111_6291862
category=3_p=0.00954220712325649
Degradome 5' end Frequency
Transcript Position
0
1
2
3
4
5
0
500
1000
1500

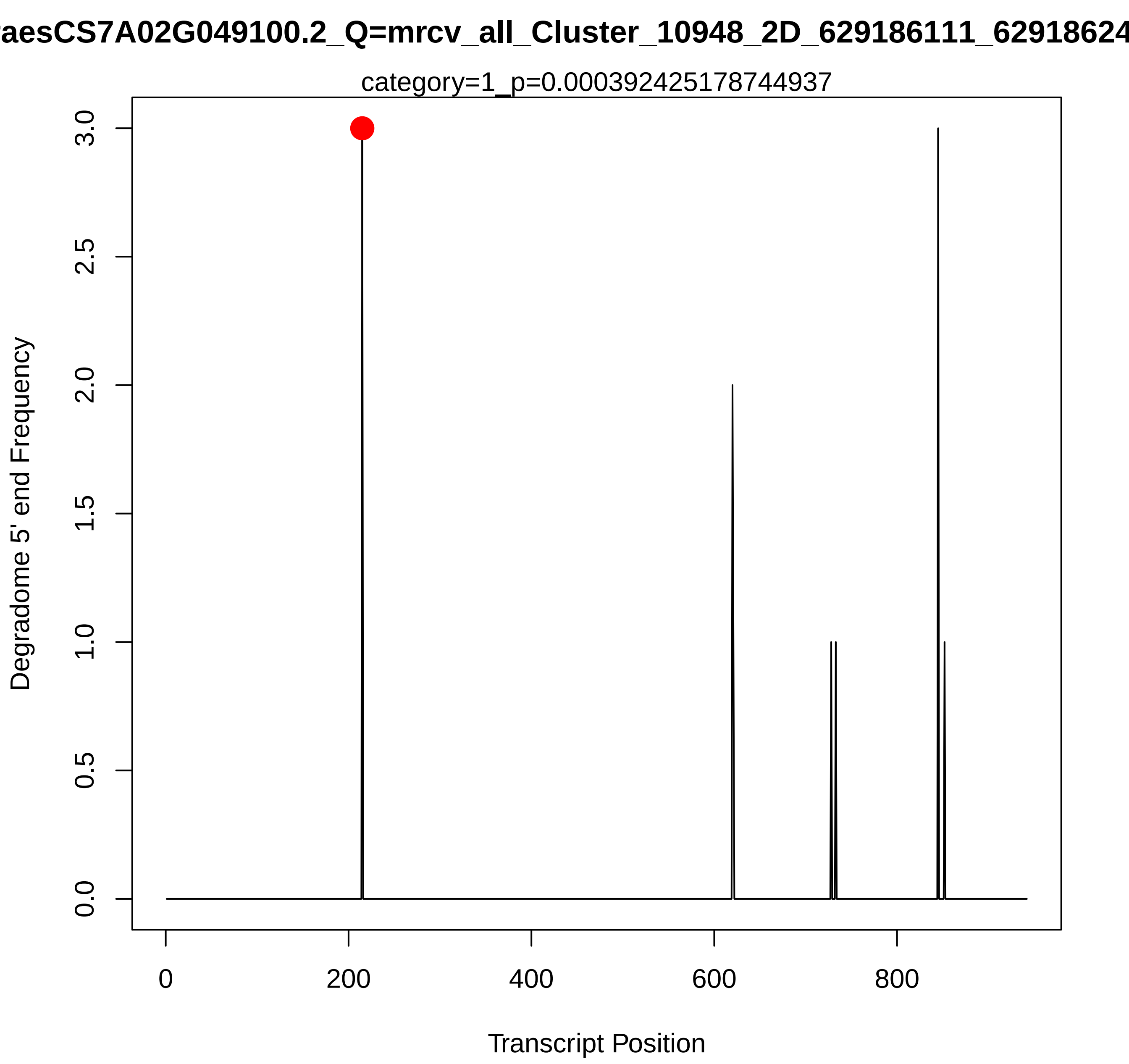
aesCS7A02G049100.2_Q=mrcv_all_Cluster_10948_2D_629186111_6291862[illegible]
category=1_p=0.000392425178744937
Degradome 5' end Frequency
Transcript Position
0.0
0.5
1.0
1.5
2.0
2.5
3.0
0
200
400
600
800

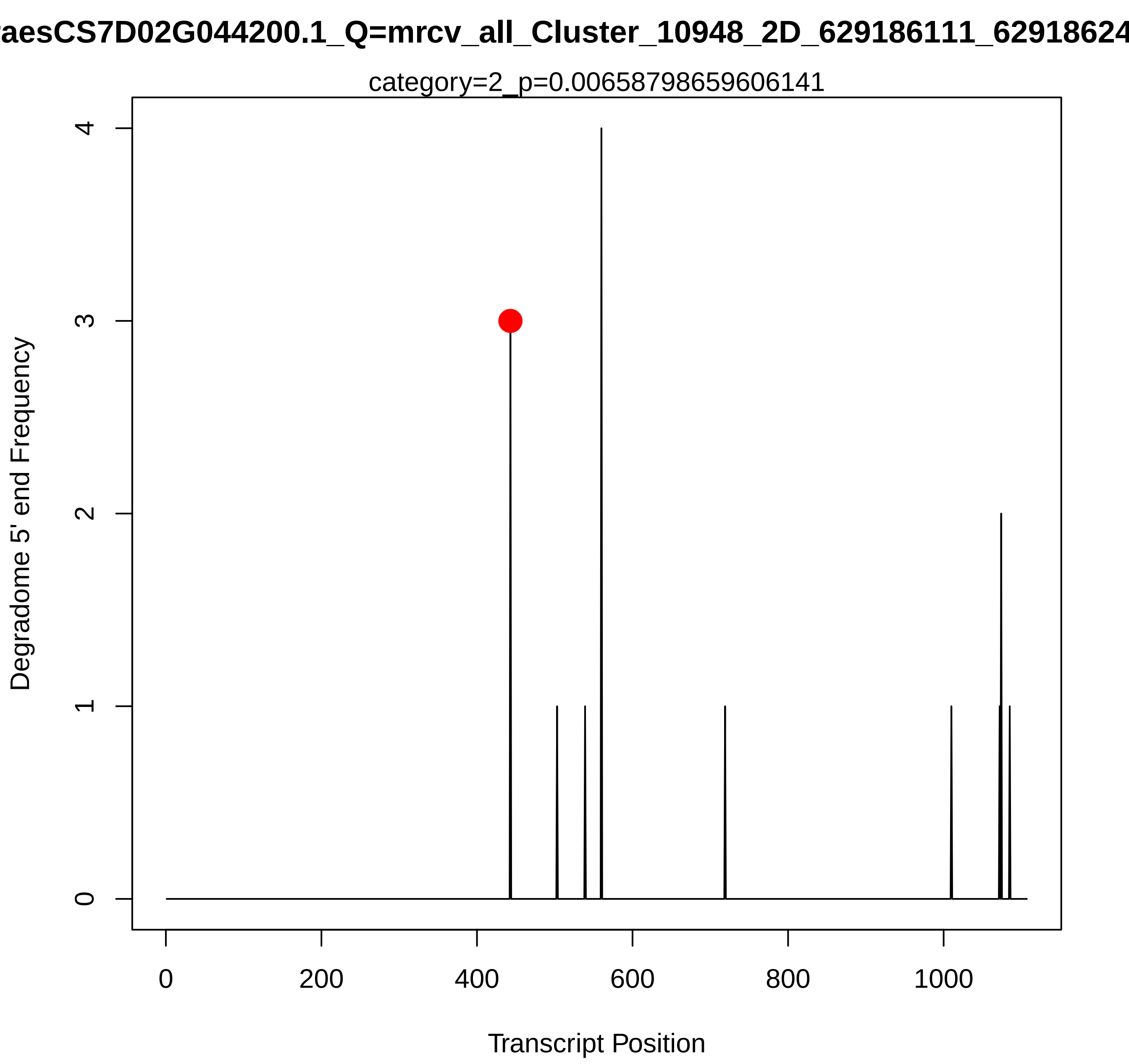

aesCS7D02G044200.1_Q=mrcv_all_Cluster_10948_2D_629186111_62918624
category=2_p=0.00658798659606141
Degradome 5' end Frequency
0
1
2
3
4
0
200
400
600
800
1000
Transcript Position

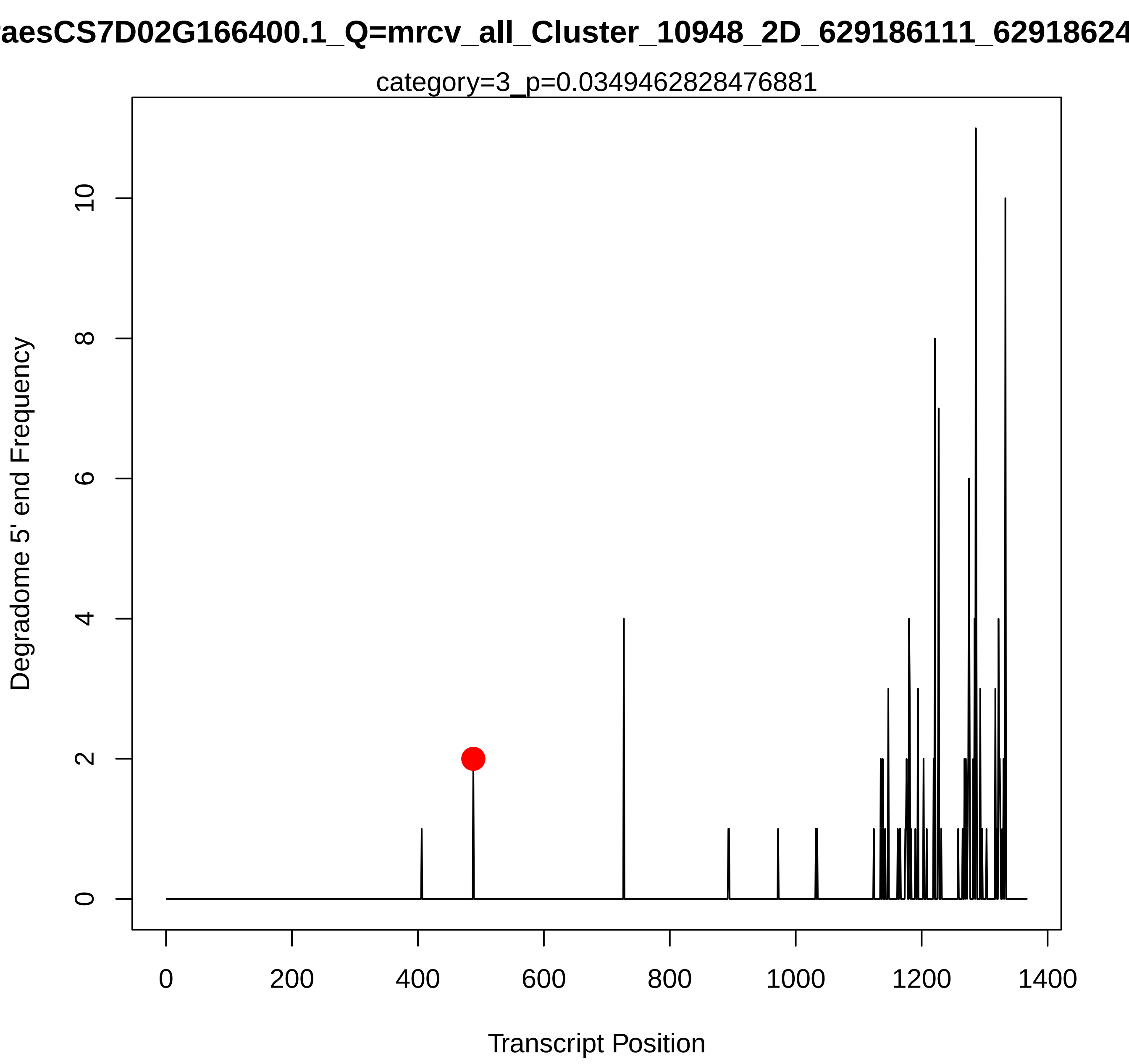
TraesCS7D02G166400.1_Q=mrcv_all_Cluster_10948_2D_629186111_629186245
category=3_p=0.0349462828476881
Degradome 5' end Frequency
Transcript Position
0
2
4
6
8
10
0
200
400
600
800
1000
1200
1400

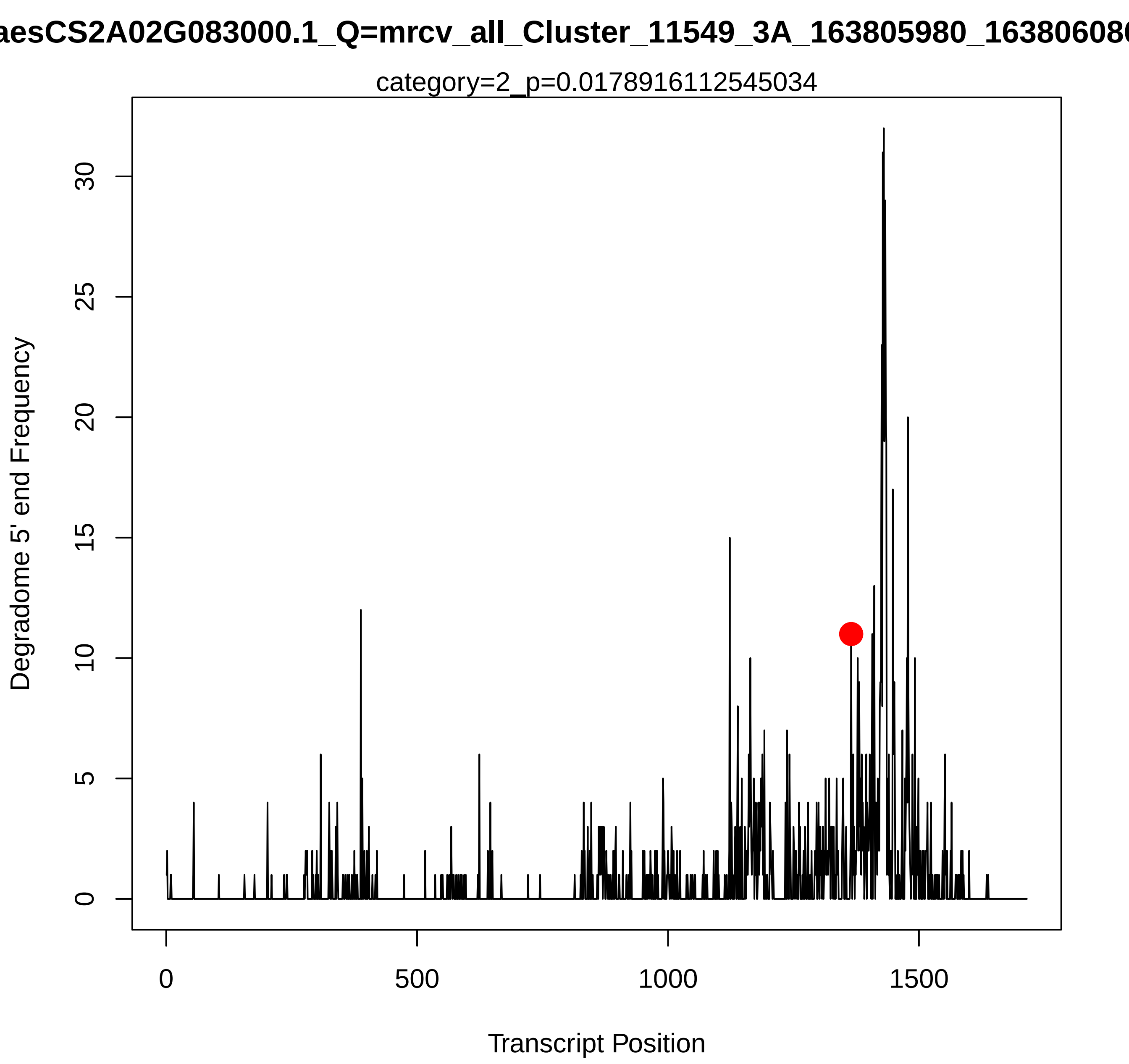

aesCS2A02G083000.1_Q=mrcv_all_Cluster_11549_3A_163805980_16380608
category=2_p=0.0178916112545034
Degradome 5' end Frequency
0
5
10
15
20
25
30
0
500
1000
1500
Transcript Position

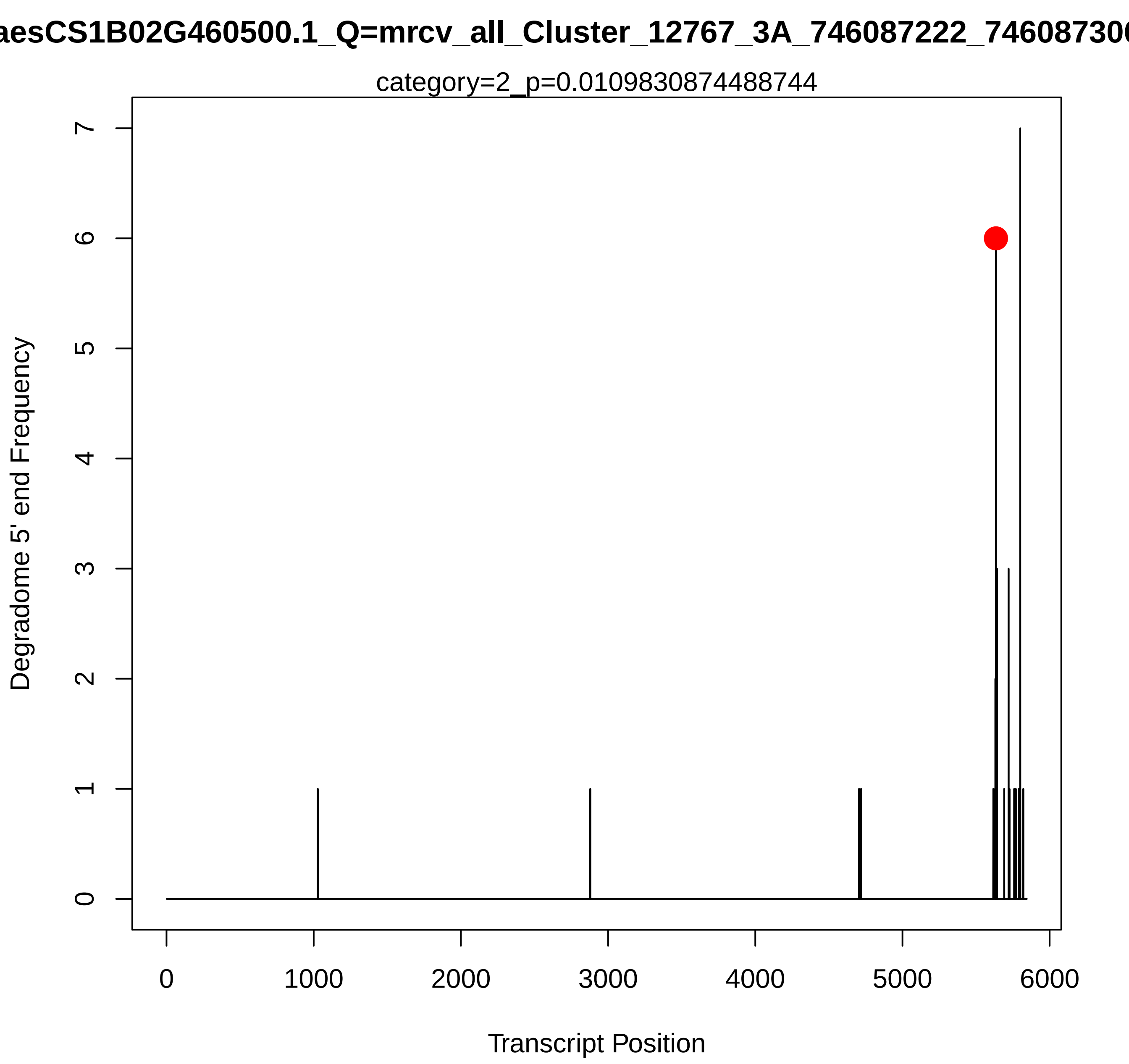
aesCS1B02G460500.1_Q=mrcv_all_Cluster_12767_3A_746087222_74608730
category=2_p=0.0109830874488744
Degradome 5' end Frequency
0
1
2
3
4
5
6
7
0
1000
2000
3000
4000
5000
6000
Transcript Position

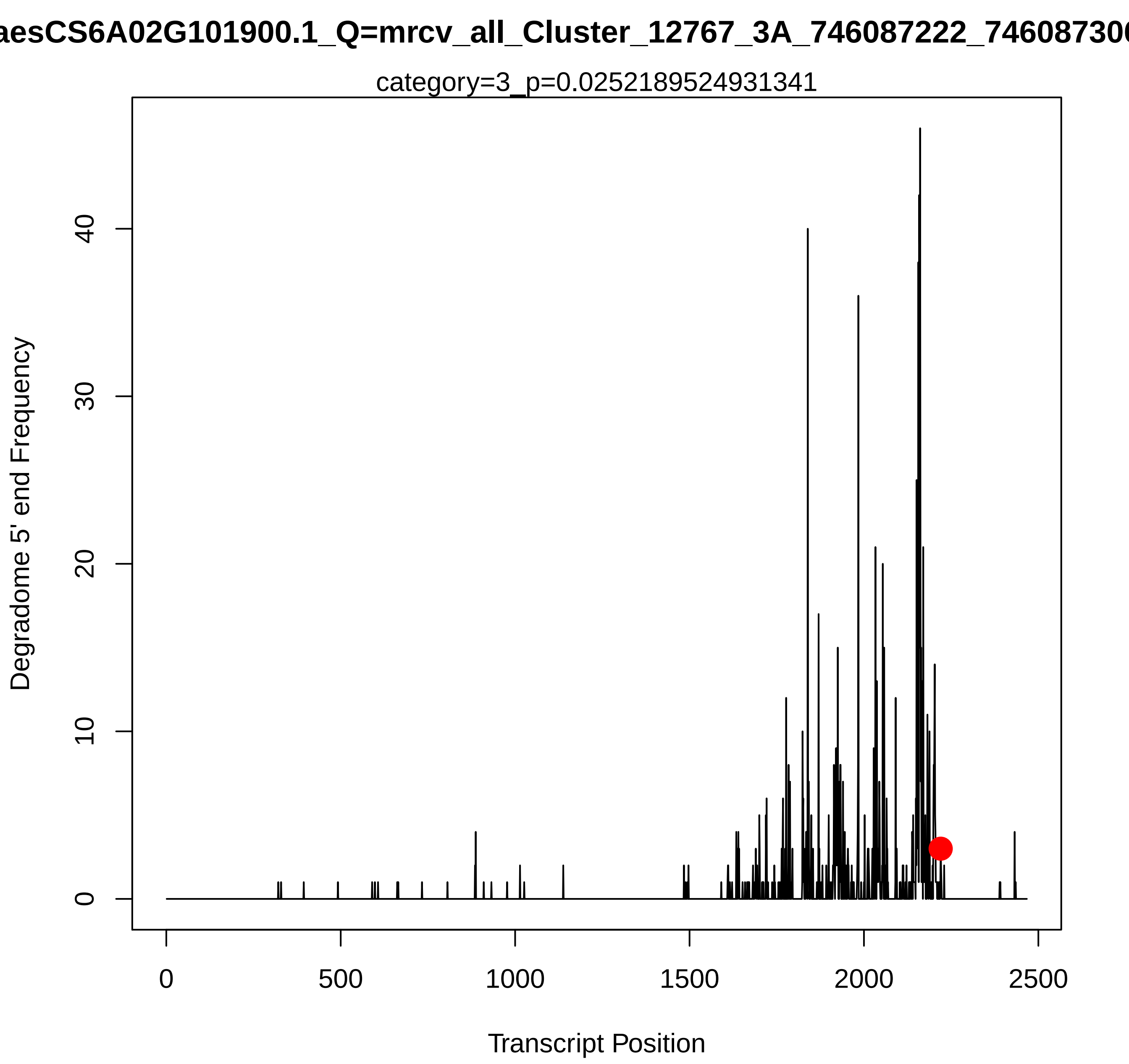
aesCS6A02G101900.1_Q=mrcv_all_Cluster_12767_3A_746087222_74608730
category=3_p=0.0252189524931341
Degradome 5' end Frequency
0
10
20
30
40
0
500
1000
1500
2000
2500
Transcript Position

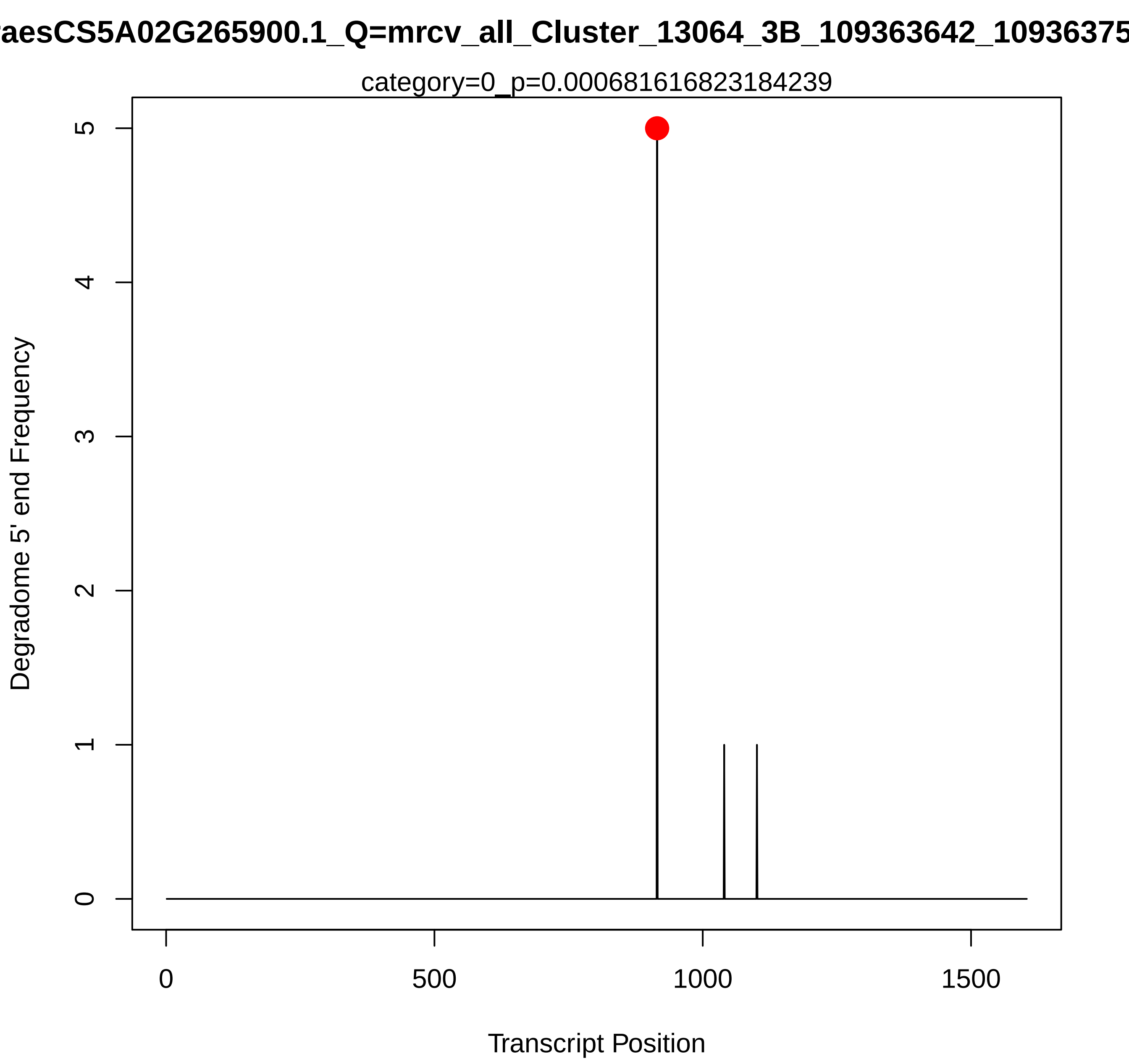
aesCS5A02G265900.1_Q=mrcv_all_Cluster_13064_3B_109363642_10936375
category=0_p=0.000681616823184239
Degradome 5' end Frequency
0
1
2
3
4
5
0
500
1000
1500
Transcript Position

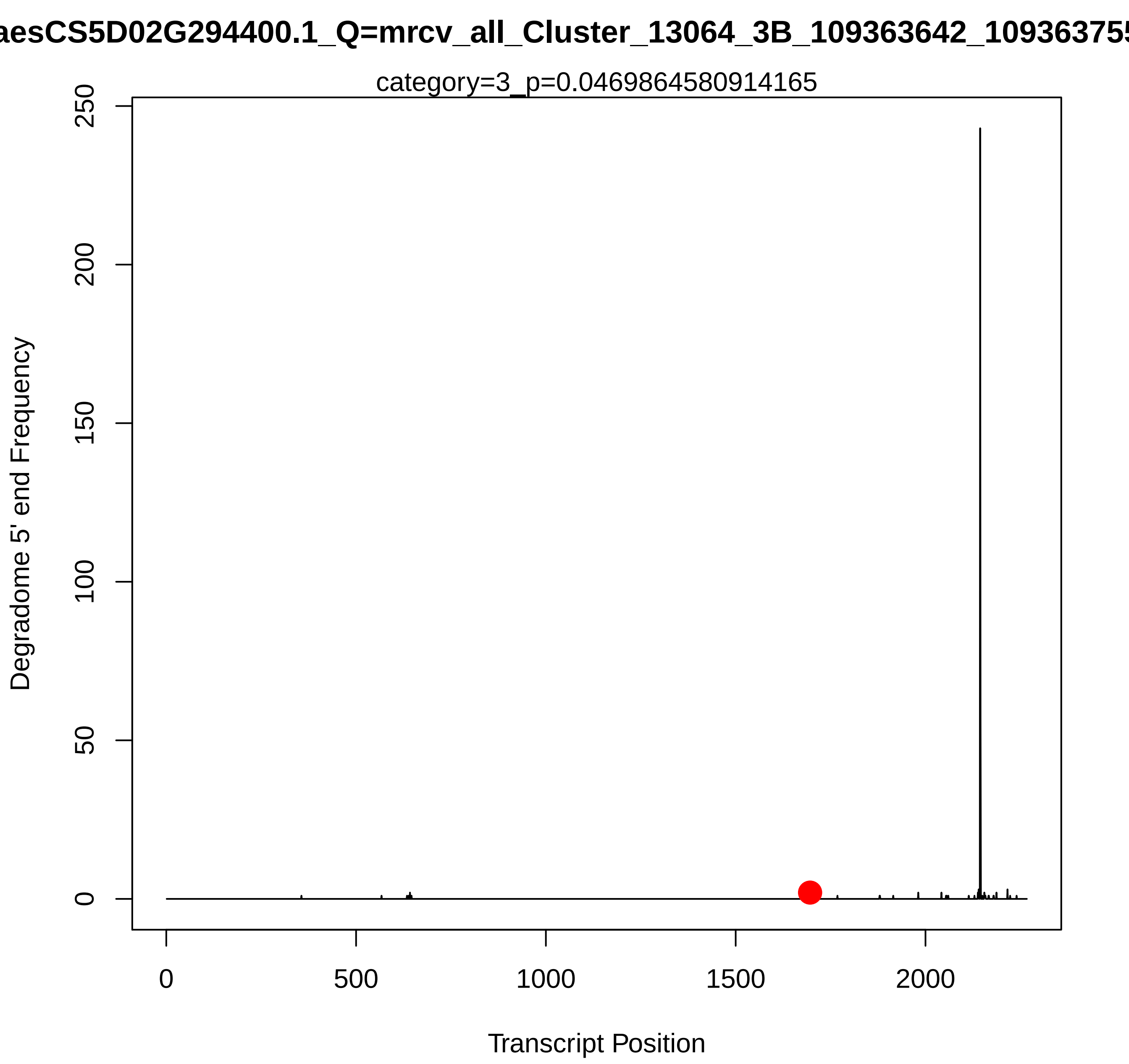
aesCS5D02G294400.1_Q=mrcv_all_Cluster_13064_3B_109363642_10936375
category=3_p=0.0469864580914165
Degradome 5' end Frequency
0
50
100
150
200
250
0
500
1000
1500
2000
Transcript Position

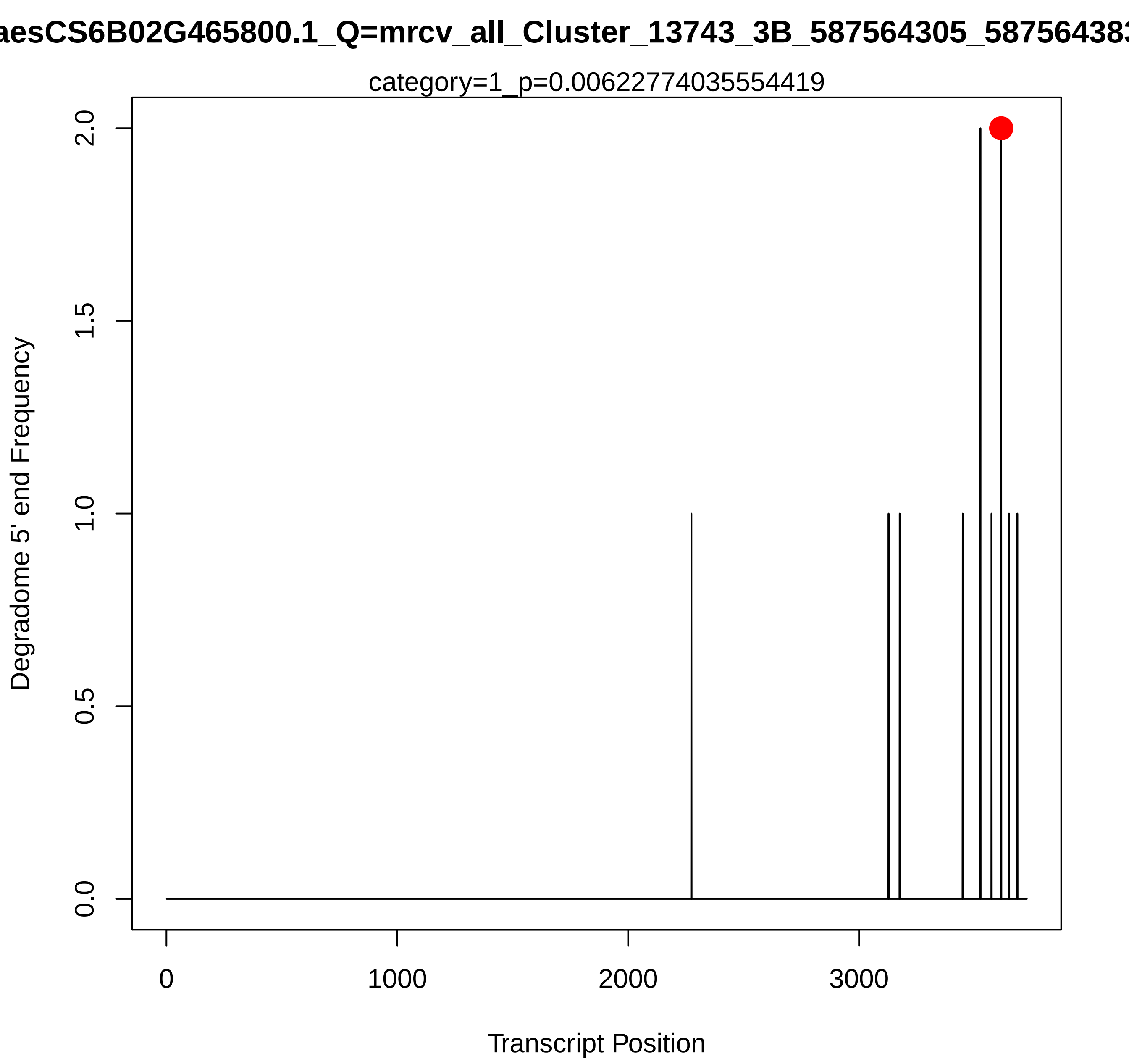

aesCS6B02G465800.1_Q=mrcv_all_Cluster_13743_3B_587564305_58756438
category=1_p=0.00622774035554419
Degradome 5' end Frequency
Transcript Position
0.0
0.5
1.0
1.5
2.0
0
1000
2000
3000

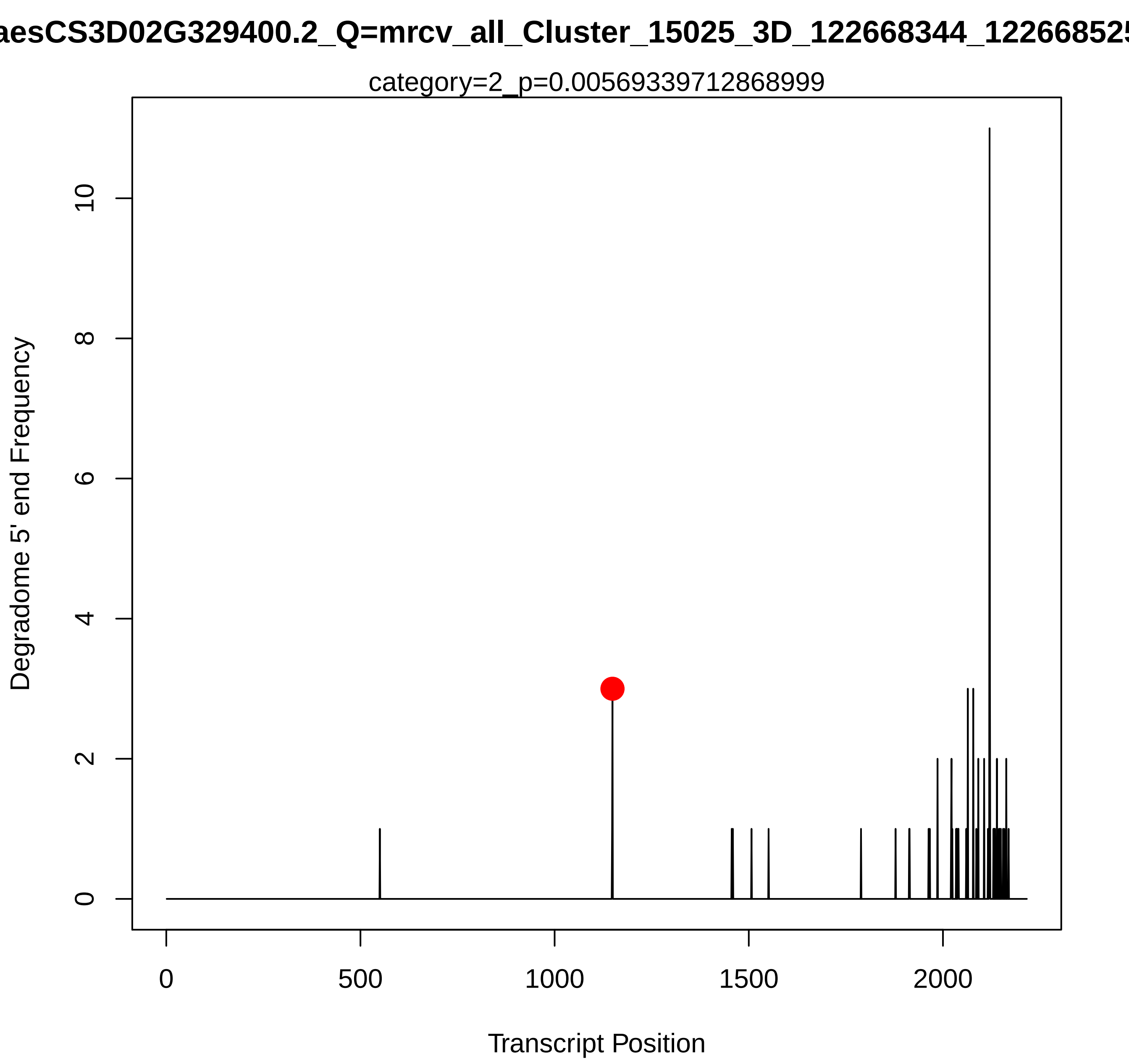
aesCS3D02G329400.2_Q=mrcv_all_Cluster_15025_3D_122668344_12266852
category=2_p=0.00569339712868999
Degradome 5' end Frequency
0
2
4
6
8
10
0
500
1000
1500
2000
Transcript Position

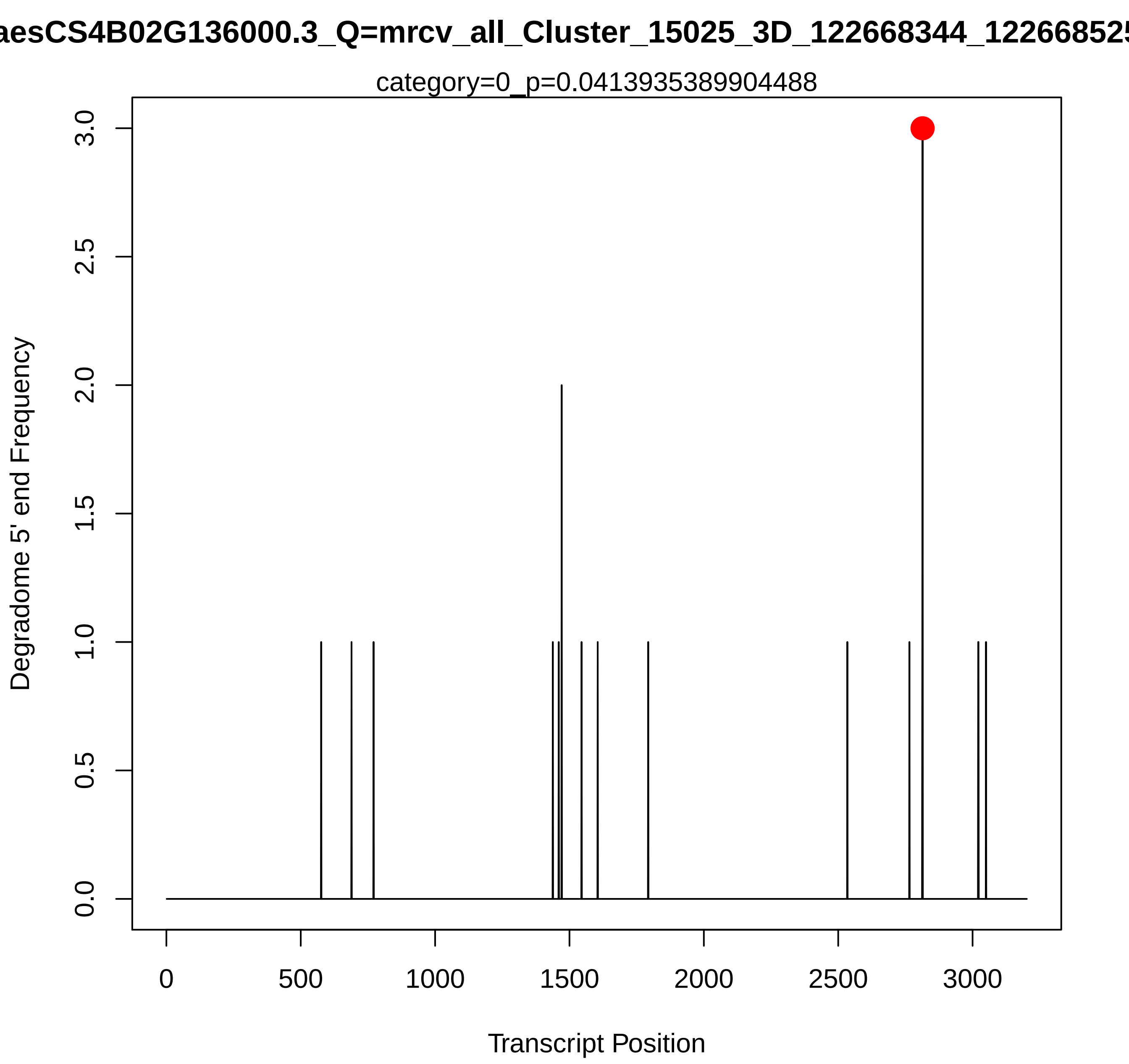

aesCS4B02G136000.3_Q=mrcv_all_Cluster_15025_3D_122668344_12266852
category=0_p=0.0413935389904488
Degradome 5' end Frequency
0.0
0.5
1.0
1.5
2.0
2.5
3.0
0
500
1000
1500
2000
2500
3000
Transcript Position

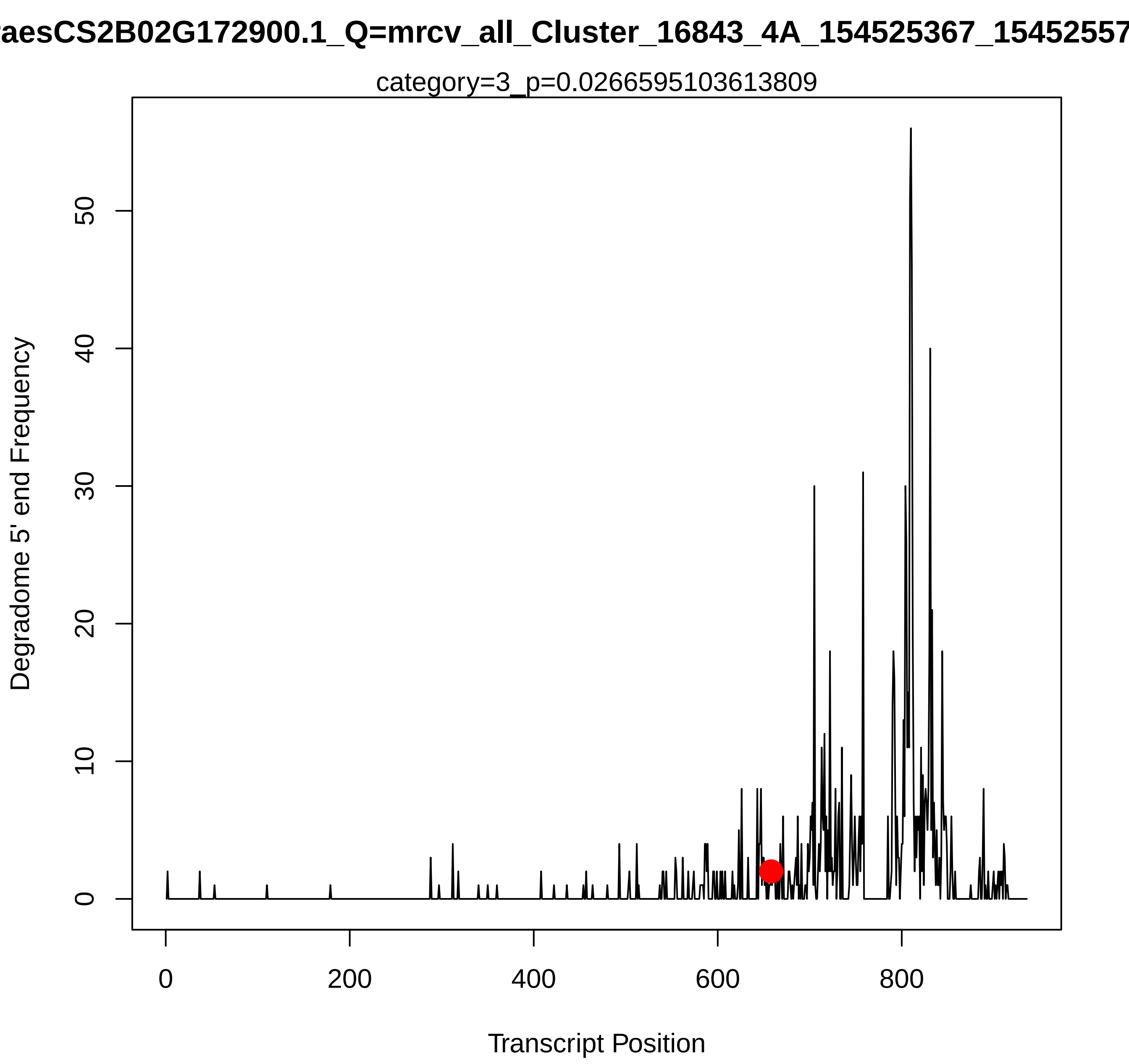

aesCS2B02G172900.1_Q=mrcv_all_Cluster_16843_4A_154525367_15452557
category=3_p=0.0266595103613809
Degradome 5' end Frequency
0
10
20
30
40
50
0
200
400
600
800
Transcript Position

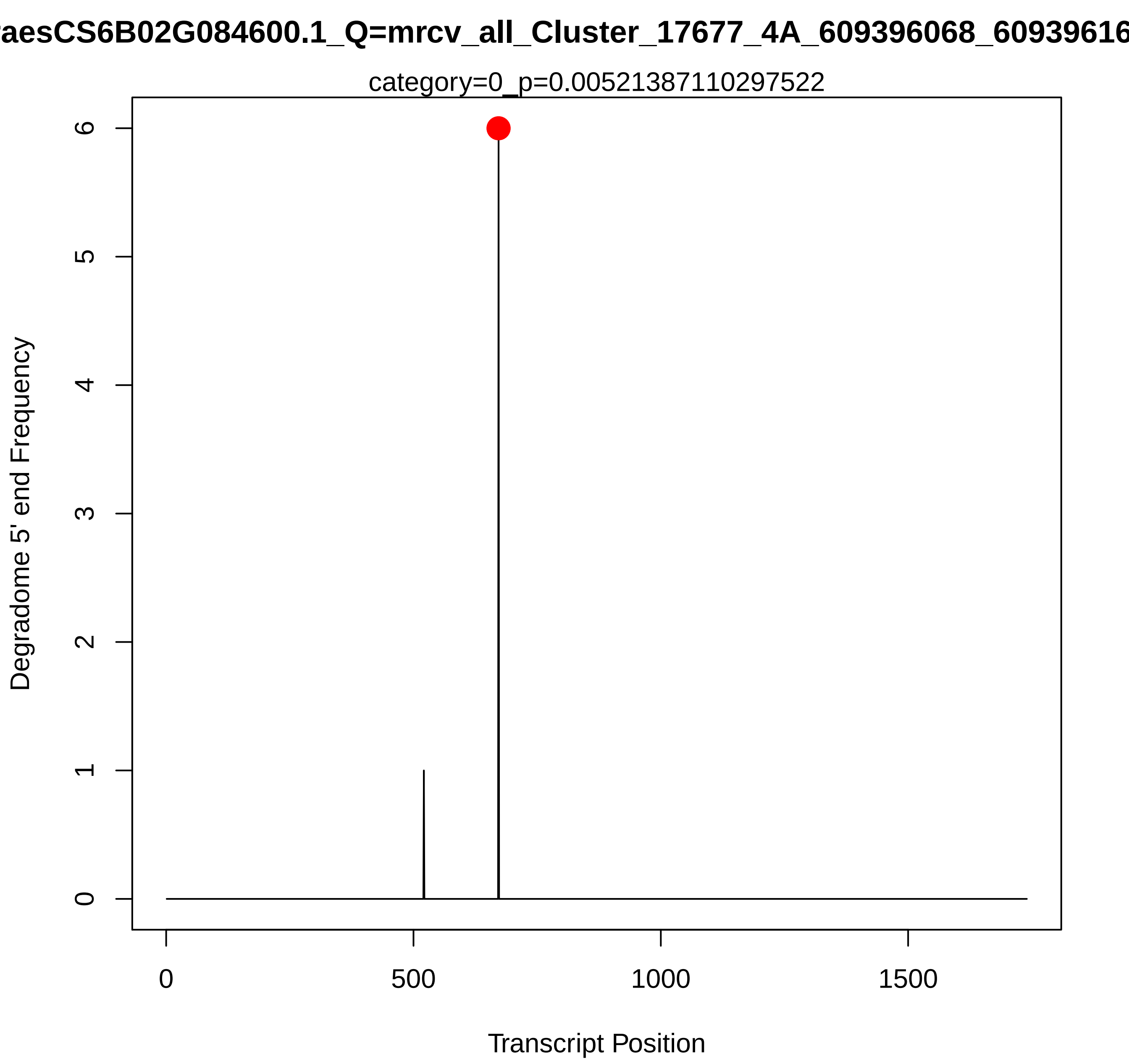
aesCS6B02G084600.1_Q=mrcv_all_Cluster_17677_4A_609396068_60939616
category=0_p=0.00521387110297522
Degradome 5' end Frequency
0
1
2
3
4
5
6
0
500
1000
1500
Transcript Position

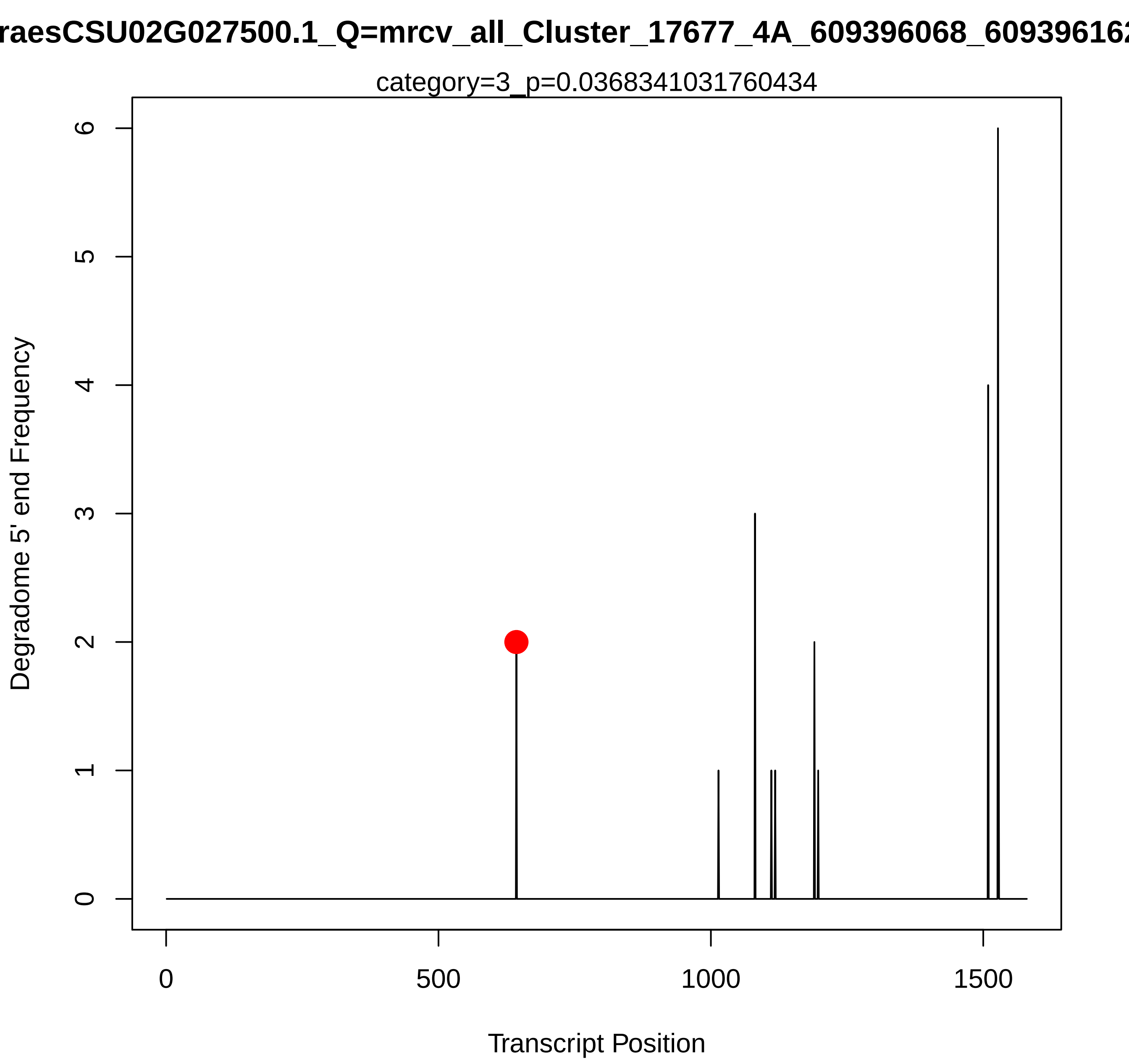
raesCSU02G027500.1_Q=mrcv_all_Cluster_17677_4A_609396068_60939616
category=3_p=0.0368341031760434
Degradome 5' end Frequency
Transcript Position
0
1
2
3
4
5
6
0
500
1000
1500

aesCS5B02G250800.1_Q=mrcv_all_Cluster_19641_4B_654094054_65409430

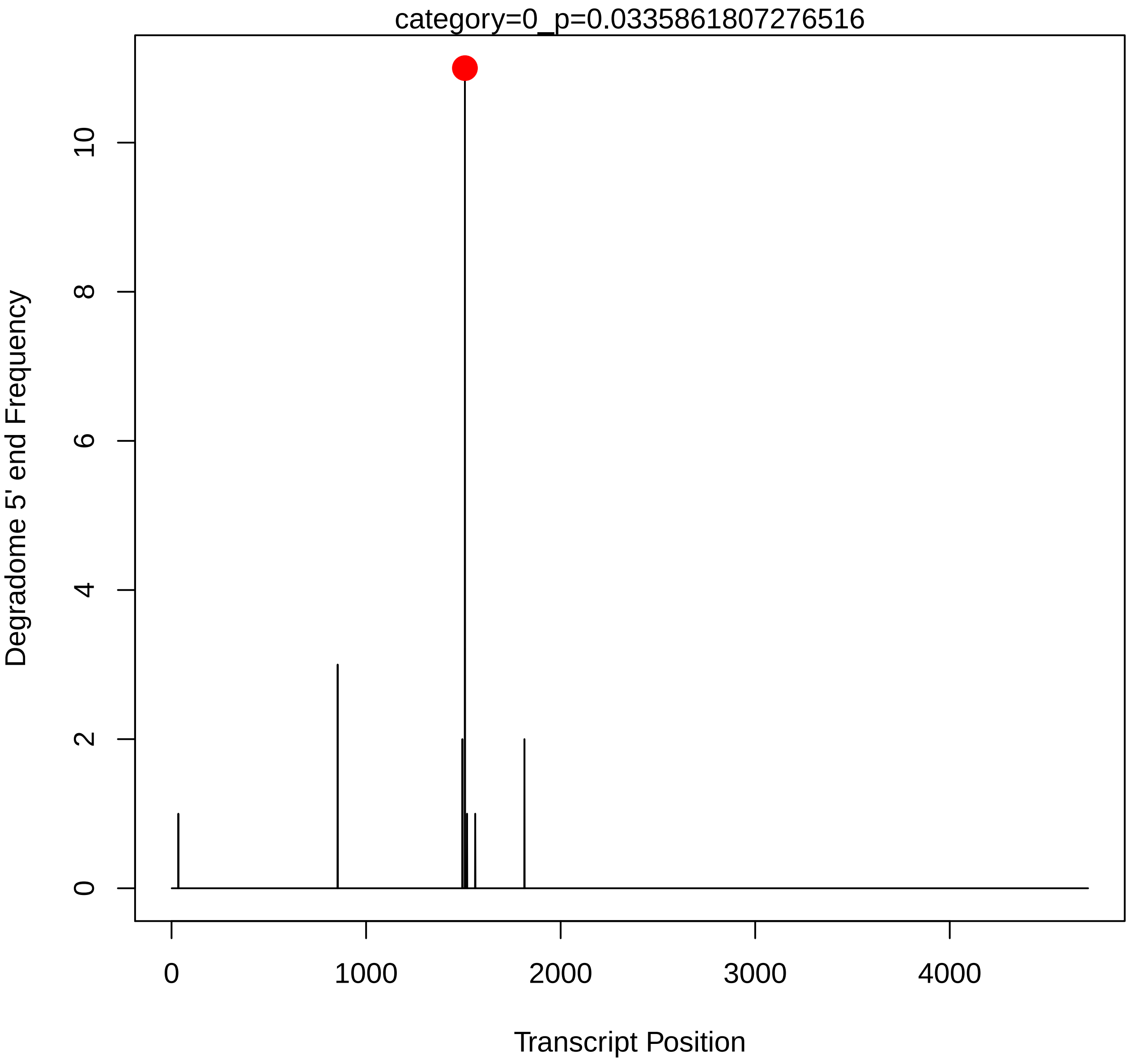

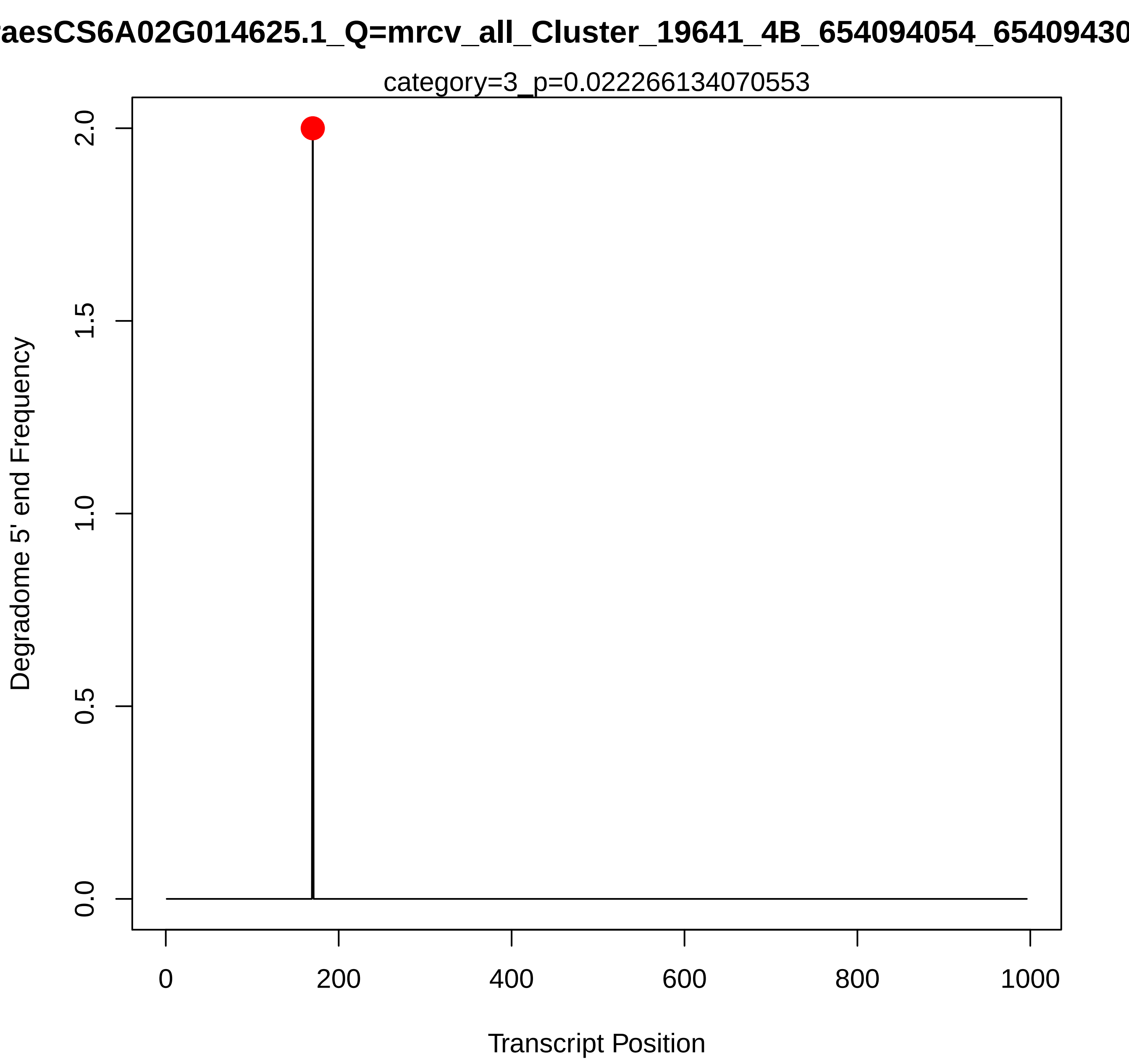

aesCS6A02G014625.1_Q=mrcv_all_Cluster_19641_4B_654094054_65409430
category=3_p=0.022266134070553
Degradome 5' end Frequency
0.0
0.5
1.0
1.5
2.0
0
200
400
600
800
1000
Transcript Position

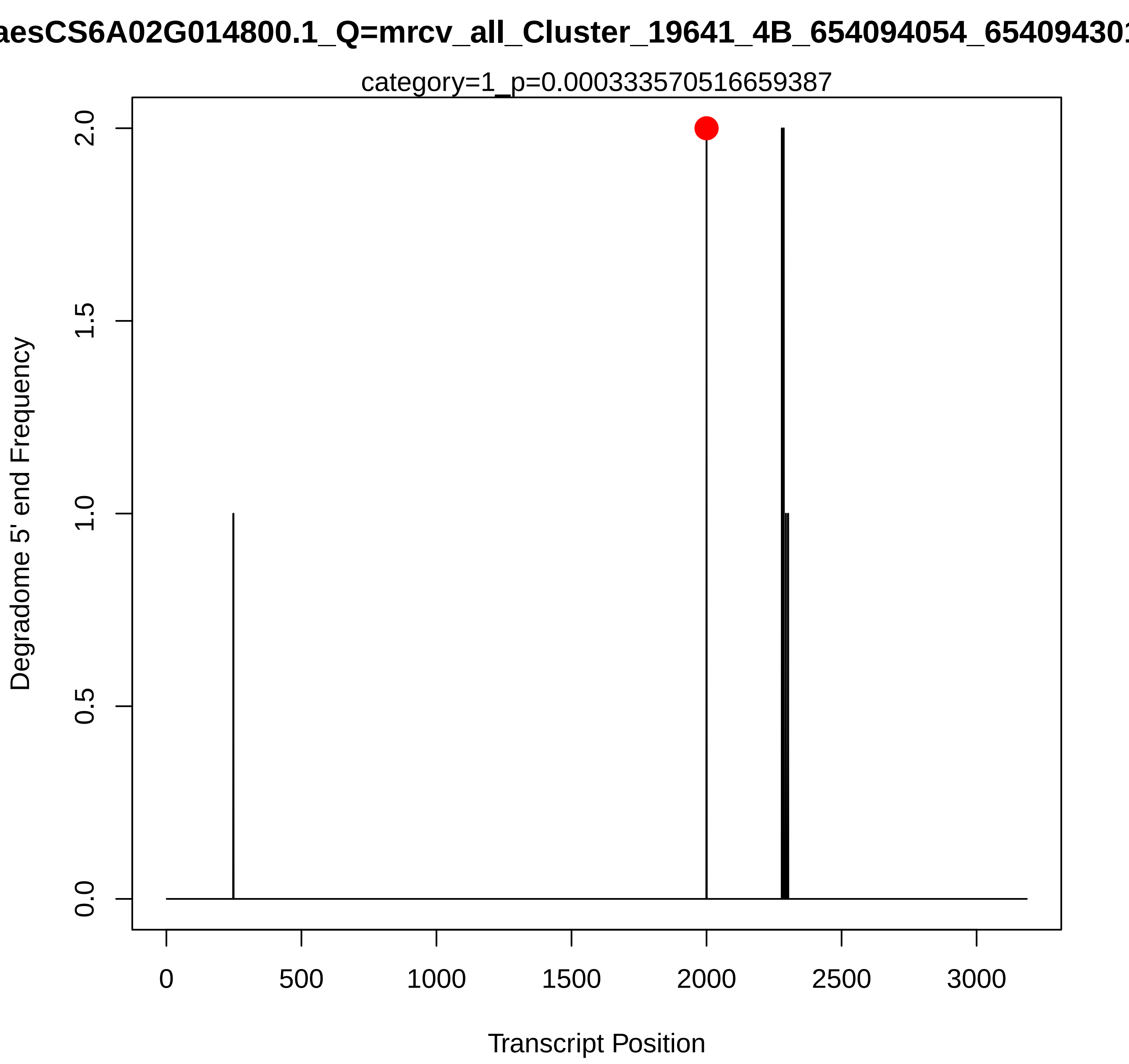
aesCS6A02G014800.1_Q=mrcv_all_Cluster_19641_4B_654094054_65409430
category=1_p=0.000333570516659387
Degradome 5' end Frequency
0.0
0.5
1.0
1.5
2.0
0
500
1000
1500
2000
2500
3000
Transcript Position

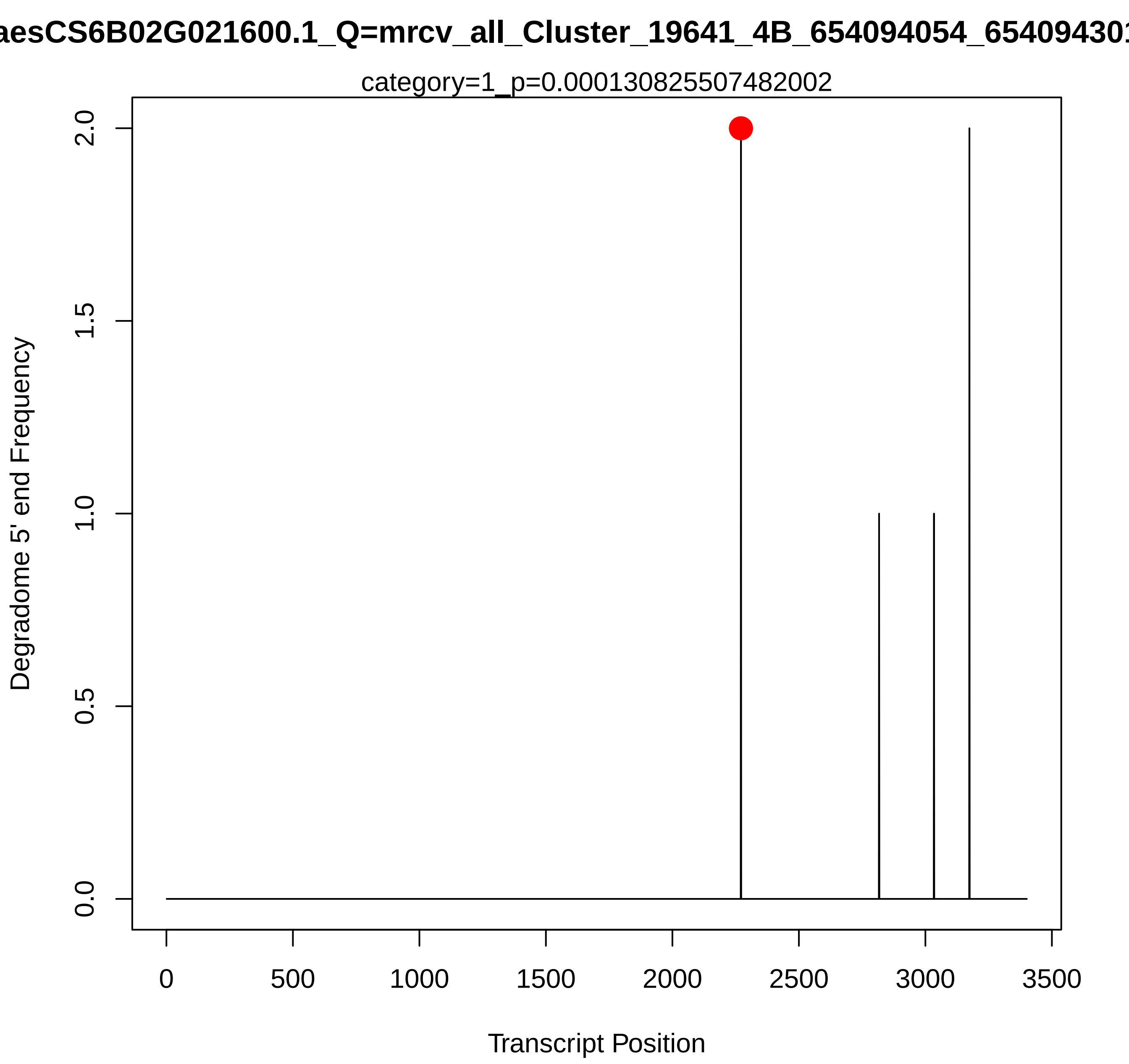
aesCS6B02G021600.1_Q=mrcv_all_Cluster_19641_4B_654094054_65409430
category=1_p=0.000130825507482002
Degradome 5' end Frequency
0.0
0.5
1.0
1.5
2.0
0
500
1000
1500
2000
2500
3000
3500
Transcript Position

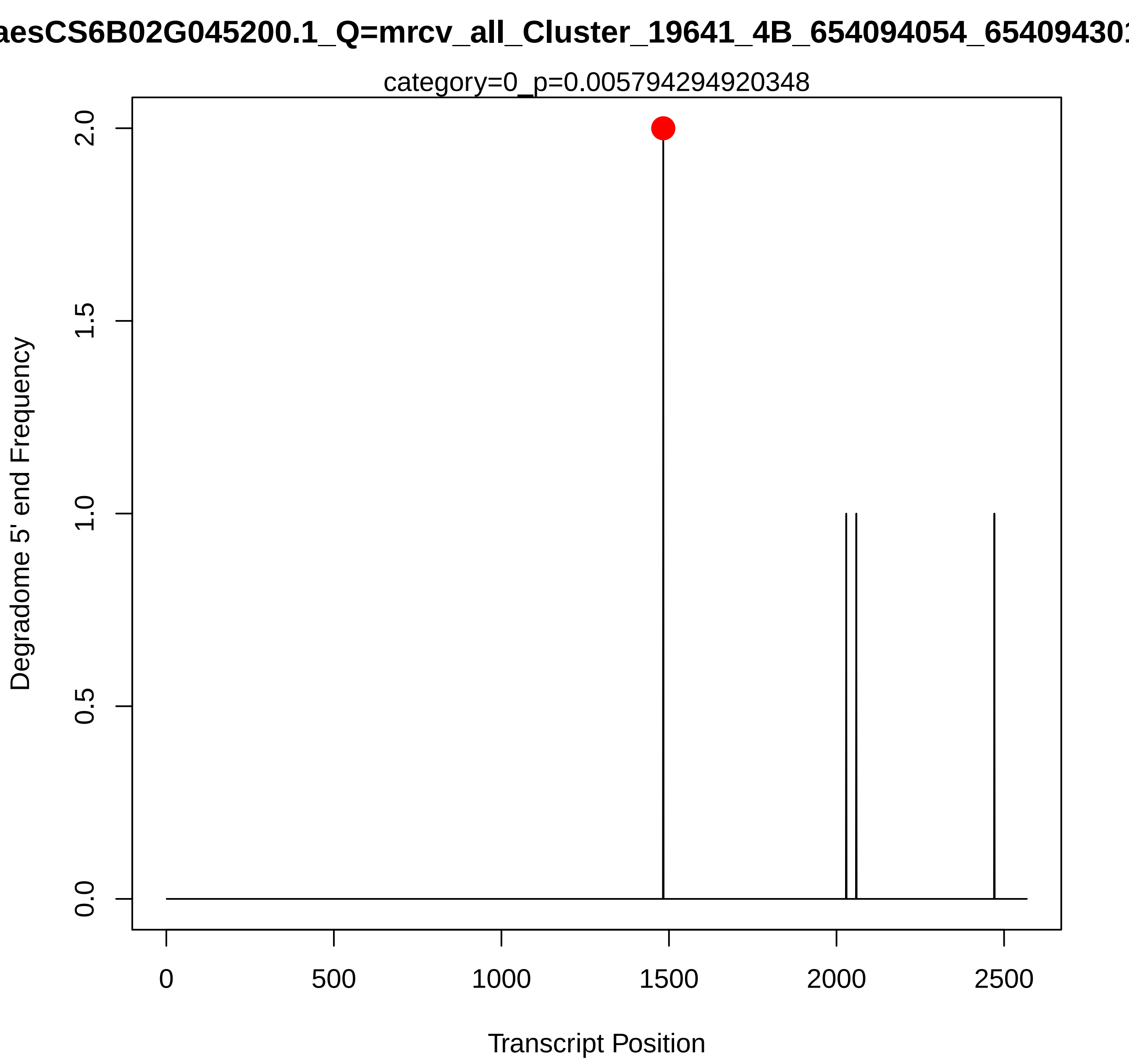

aesCS6B02G045200.1_Q=mrcv_all_Cluster_19641_4B_654094054_65409430
category=0_p=0.005794294920348
Degradome 5' end Frequency
Transcript Position
0.0
0.5
1.0
1.5
2.0
0
500
1000
1500
2000
2500

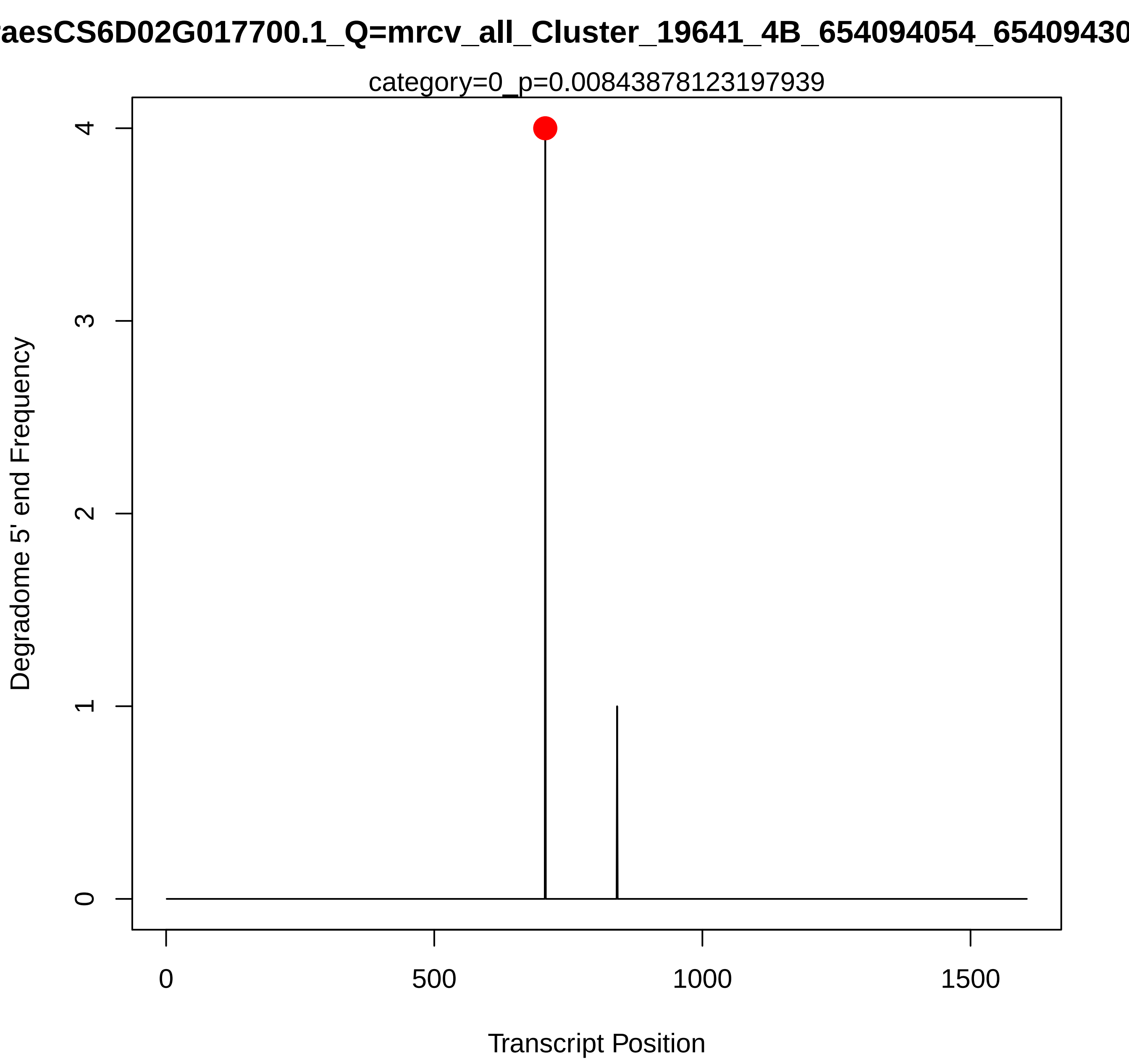

aesCS6D02G017700.1_Q=mrcv_all_Cluster_19641_4B_654094054_65409430
category=0_p=0.00843878123197939
Degradome 5' end Frequency
Transcript Position
0
1
2
3
4
0
500
1000
1500

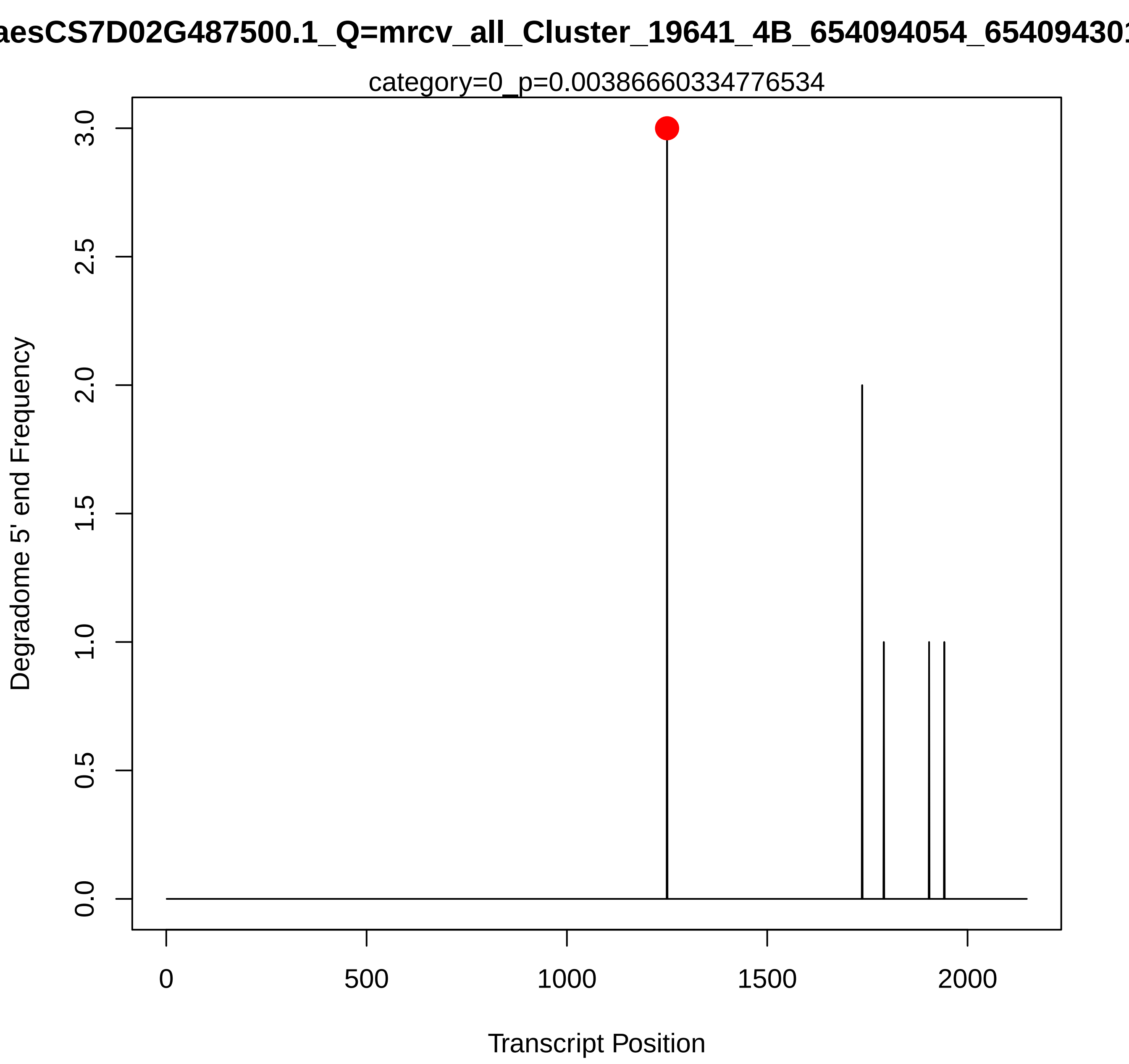
aesCS7D02G487500.1_Q=mrcv_all_Cluster_19641_4B_654094054_65409430
category=0_p=0.00386660334776534
Degradome 5' end Frequency
Transcript Position
0.0
0.5
1.0
1.5
2.0
2.5
3.0
0
500
1000
1500
2000

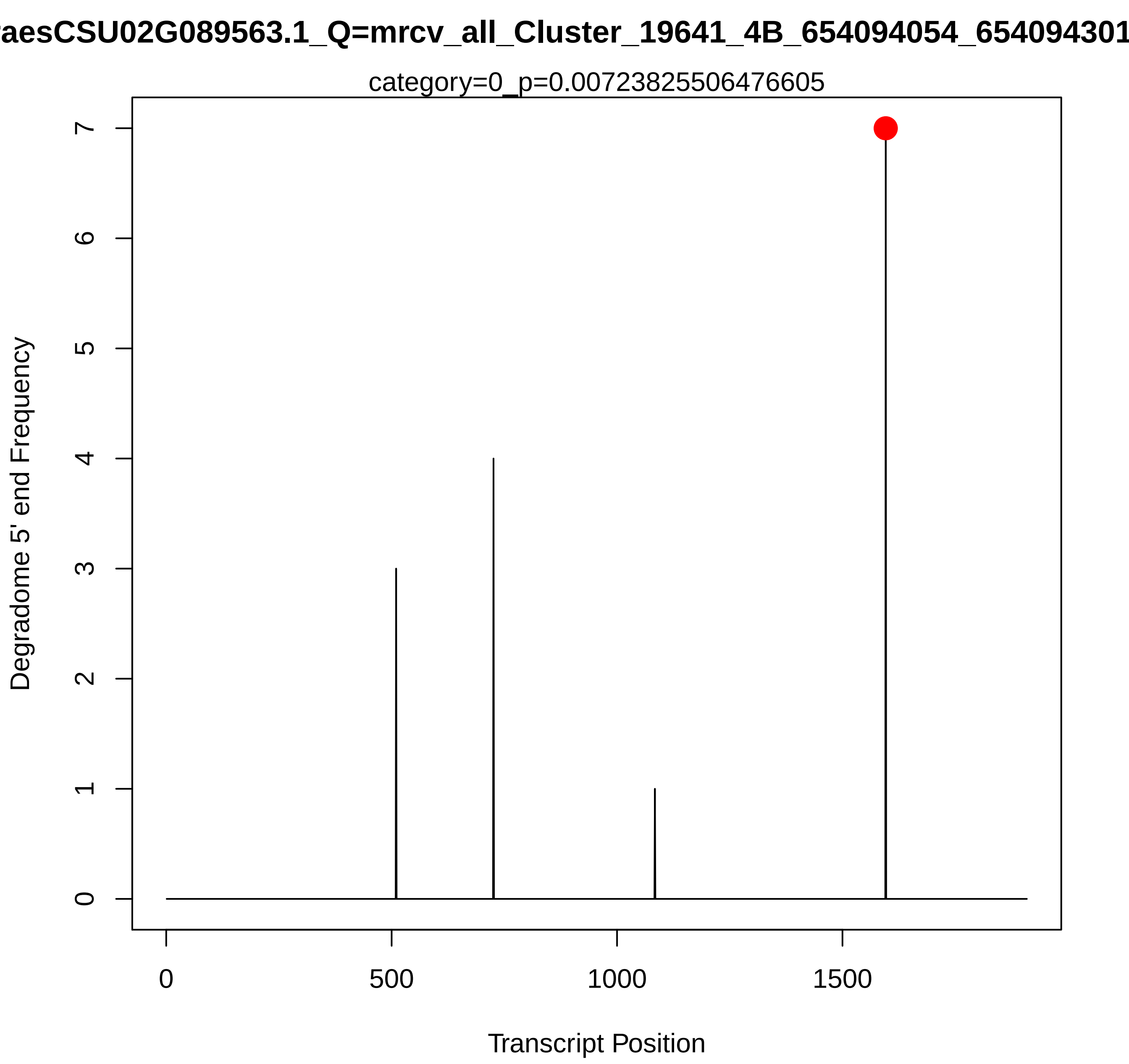

aesCSU02G089563.1_Q=mrcv_all_Cluster_19641_4B_654094054_654094301
category=0_p=0.00723825506476605
Degradome 5' end Frequency
0
1
2
3
4
5
6
7
0
500
1000
1500
Transcript Position

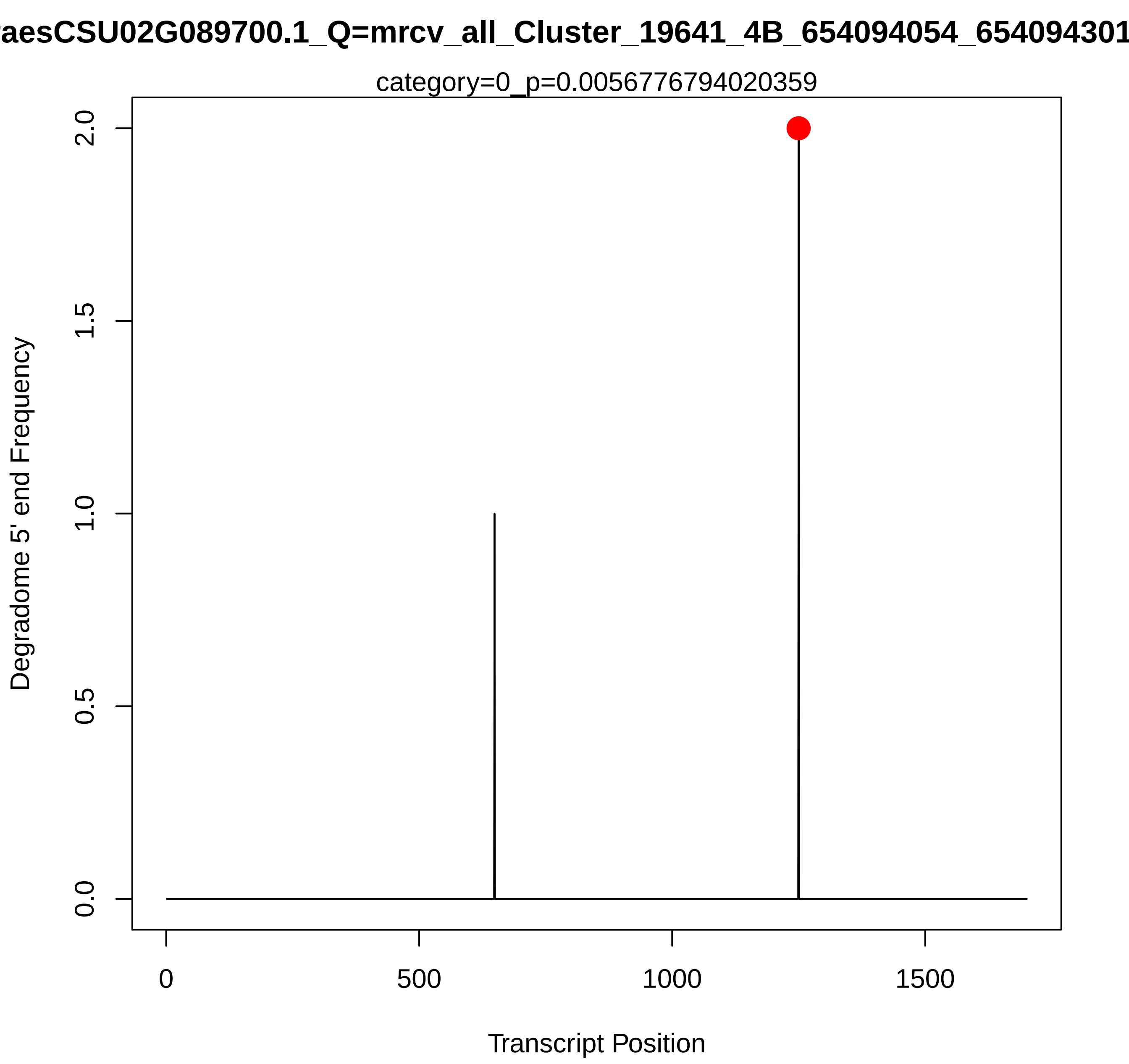

aesCSU02G089700.1_Q=mrcv_all_Cluster_19641_4B_654094054_654094301
category=0_p=0.0056776794020359
Degradome 5' end Frequency
0.0
0.5
1.0
1.5
2.0
0
500
1000
1500
Transcript Position

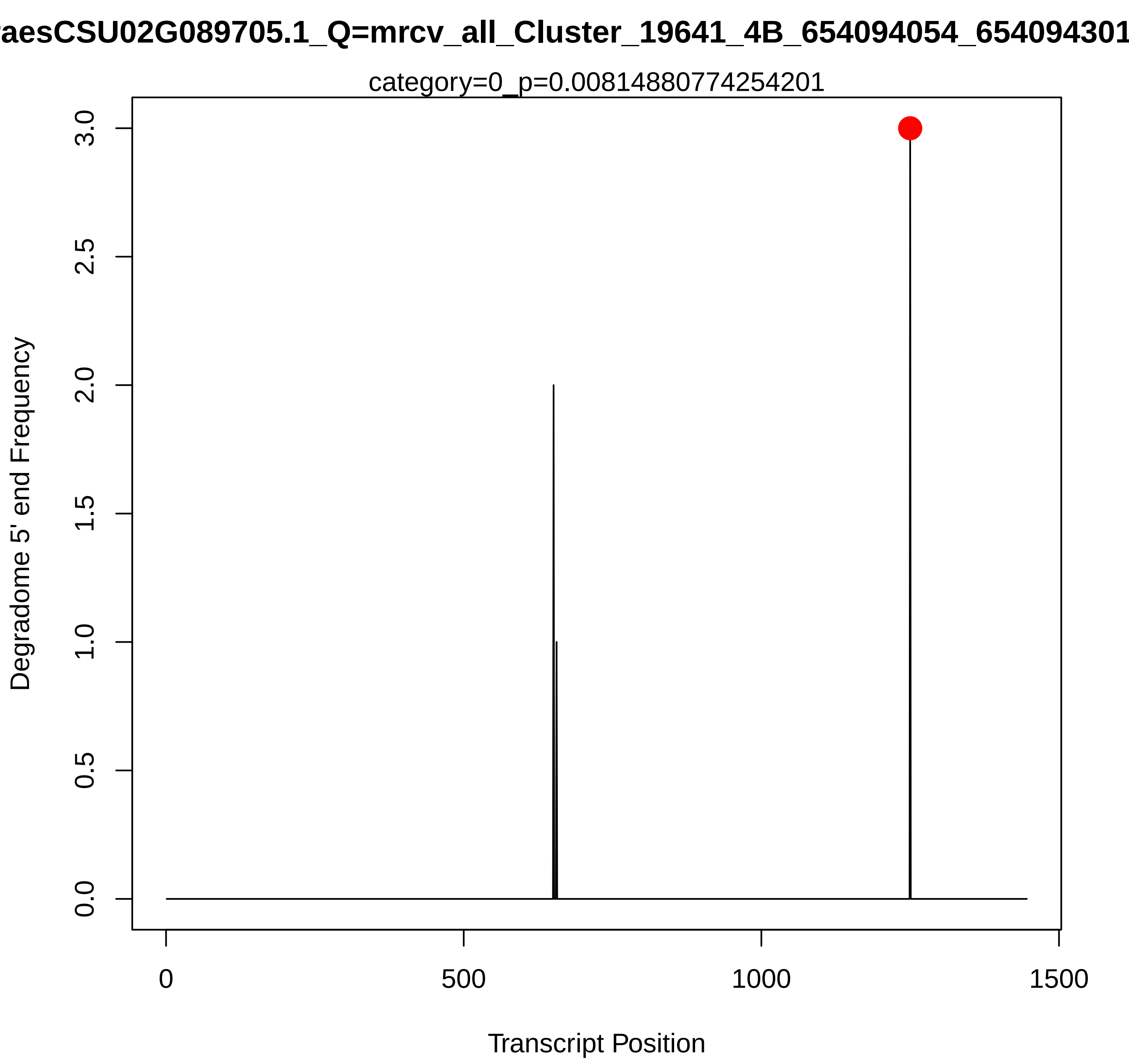

aesCSU02G089705.1_Q=mrcv_all_Cluster_19641_4B_654094054_654094301
category=0_p=0.00814880774254201
Degradome 5' end Frequency
0.0
0.5
1.0
1.5
2.0
2.5
3.0
Transcript Position
0
500
1000
1500

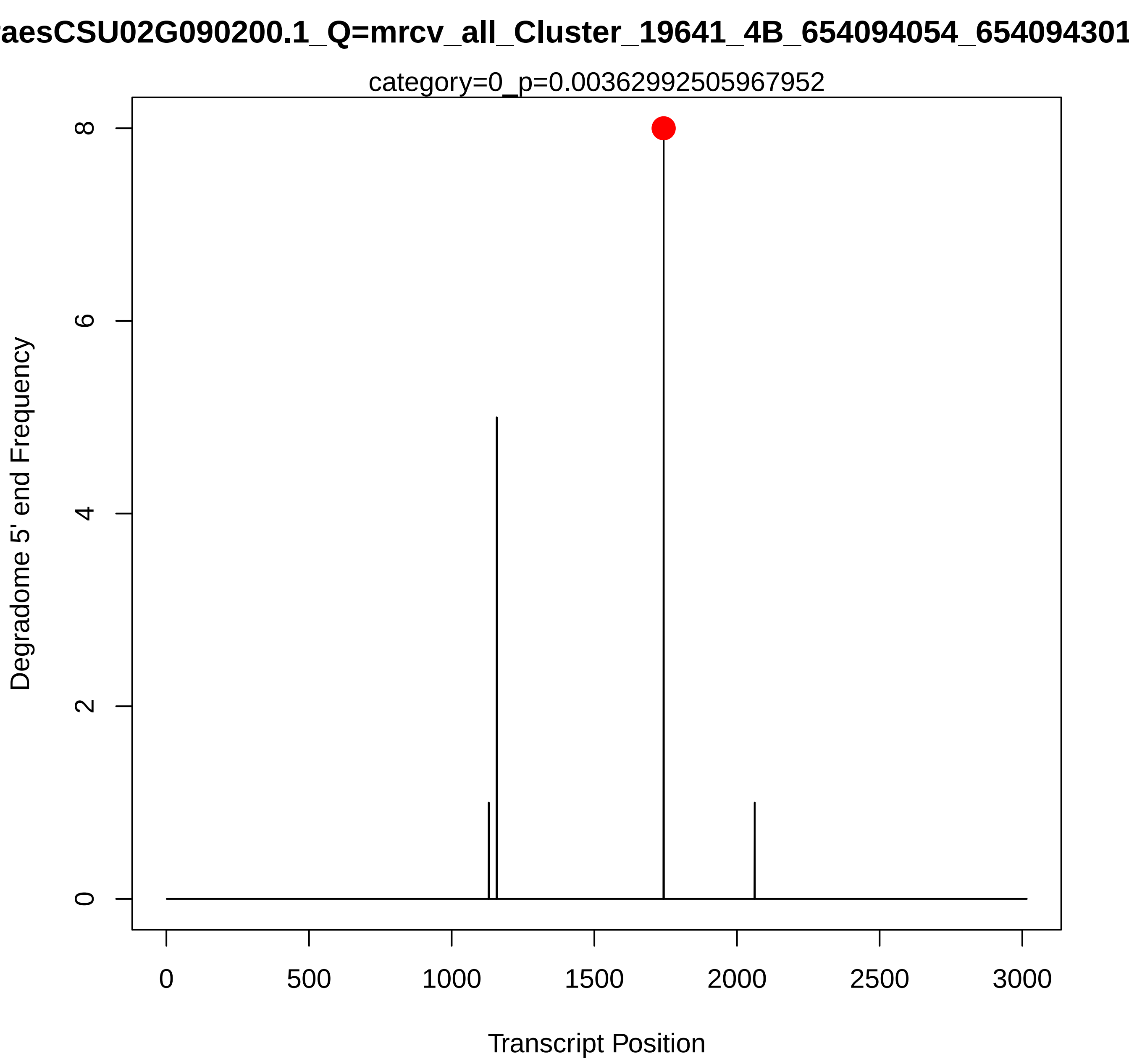

aesCSU02G090200.1_Q=mrcv_all_Cluster_19641_4B_654094054_654094301
category=0_p=0.00362992505967952
Degradome 5' end Frequency
0
2
4
6
8
0
500
1000
1500
2000
2500
3000
Transcript Position

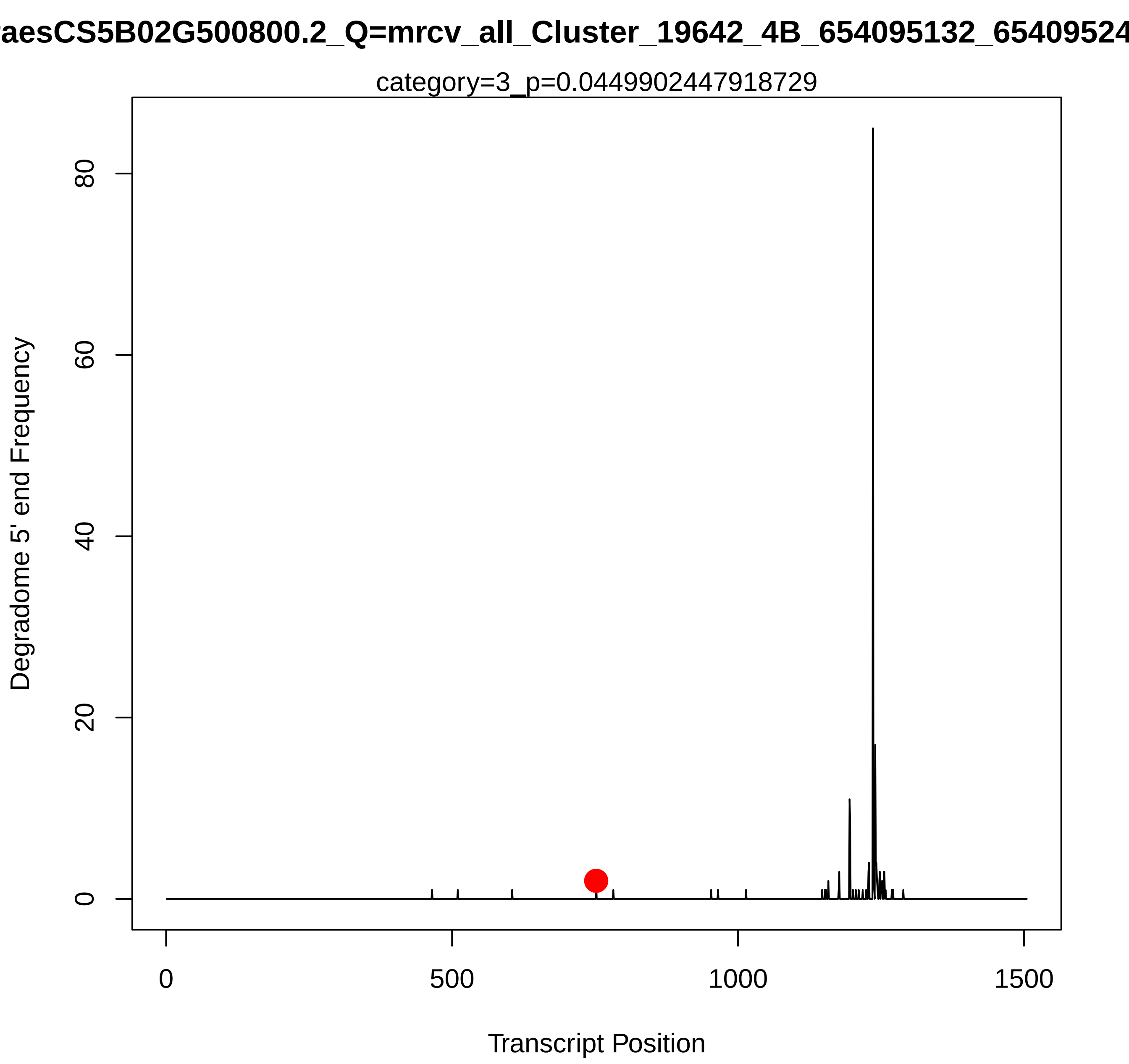
aesCS5B02G500800.2_Q=mrcv_all_Cluster_19642_4B_654095132_65409524
category=3_p=0.0449902447918729
Degradome 5' end Frequency
0
20
40
60
80
0
500
1000
1500
Transcript Position

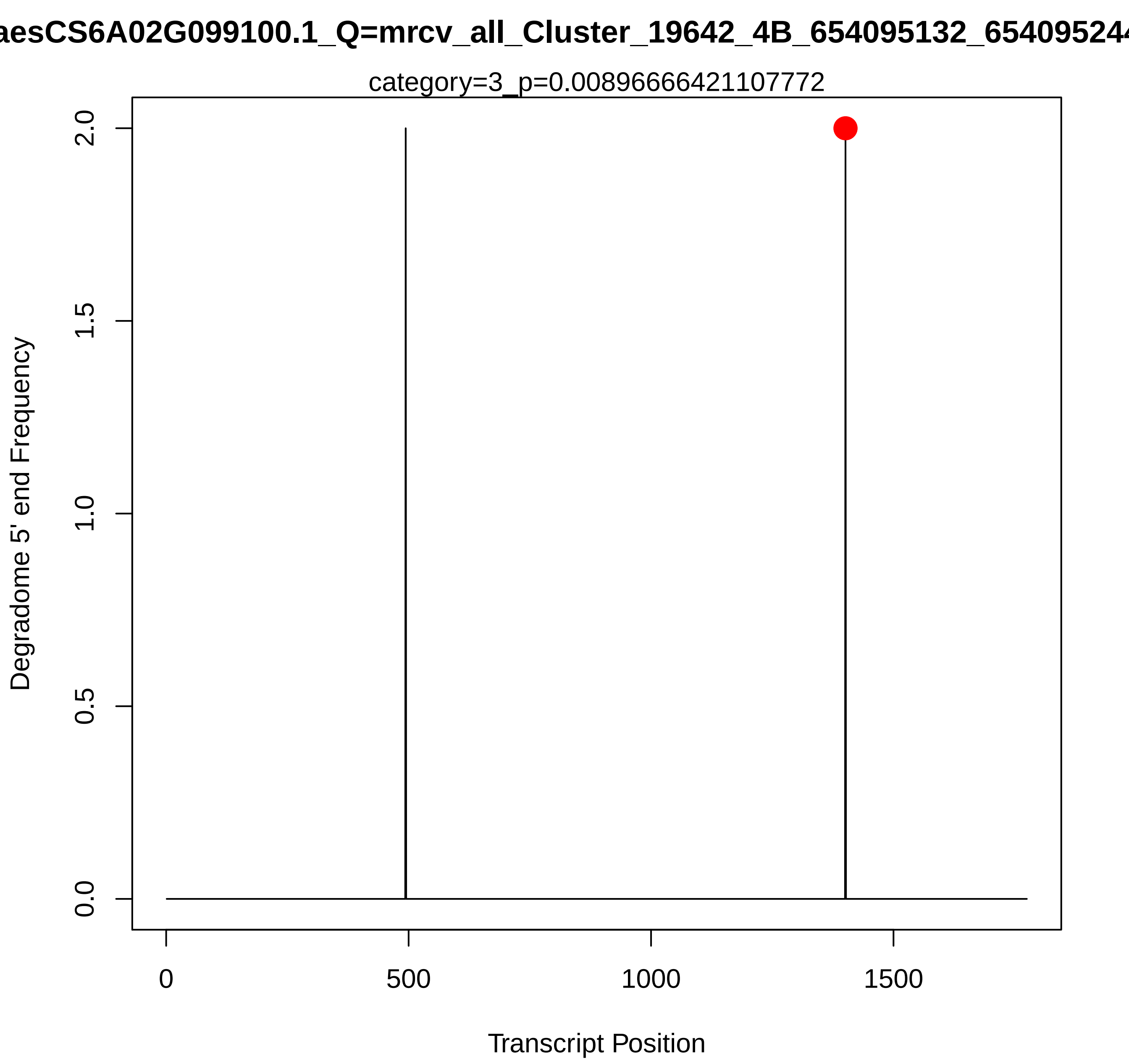
aesCS6A02G099100.1_Q=mrcv_all_Cluster_19642_4B_654095132_65409524
category=3_p=0.00896666421107772
Degradome 5' end Frequency
0.0
0.5
1.0
1.5
2.0
0
500
1000
1500
Transcript Position

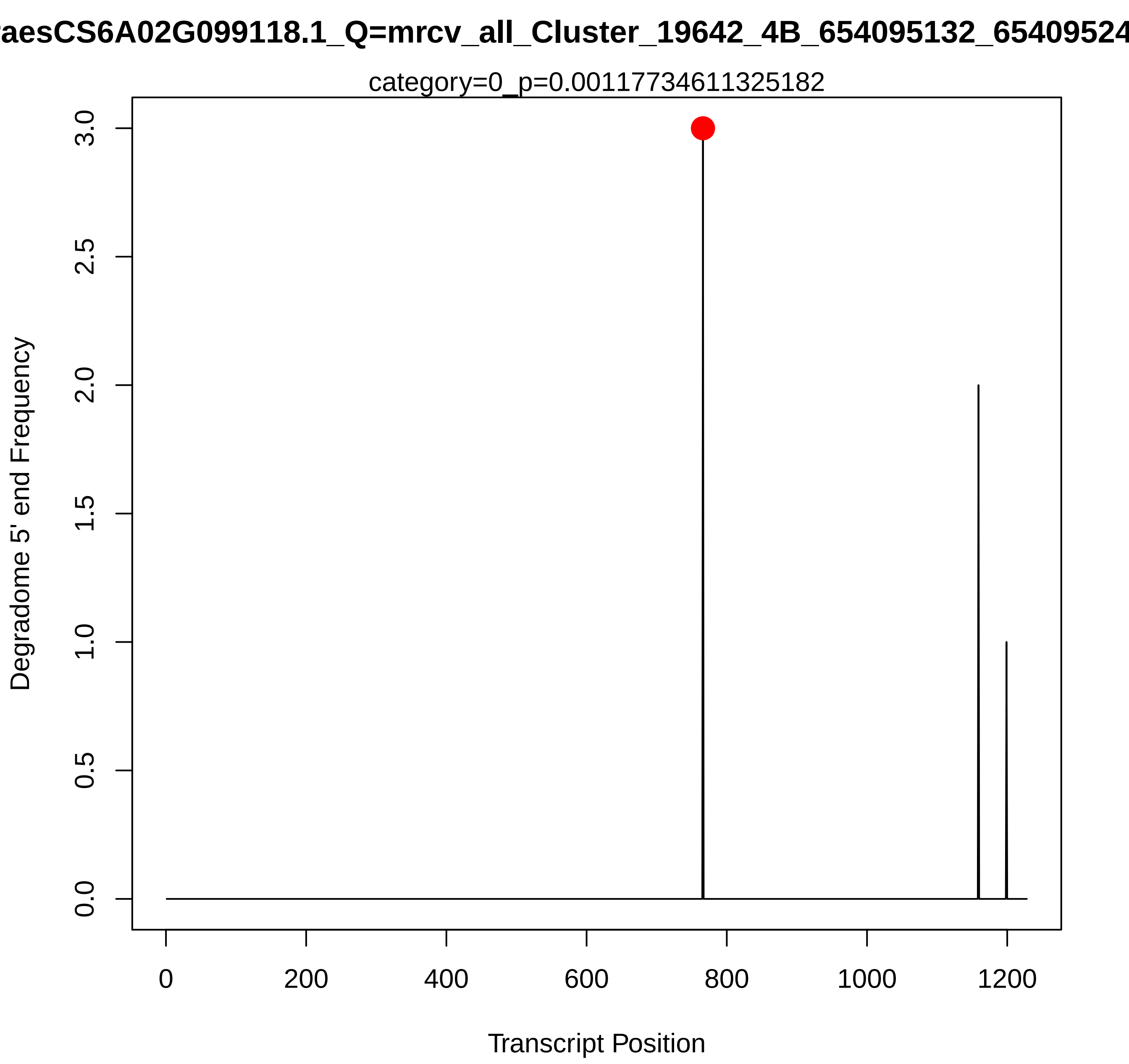

aesCS6A02G099118.1_Q=mrcv_all_Cluster_19642_4B_654095132_65409524
category=0_p=0.00117734611325182
Degradome 5' end Frequency
0.0
0.5
1.0
1.5
2.0
2.5
3.0
0
200
400
600
800
1000
1200
Transcript Position

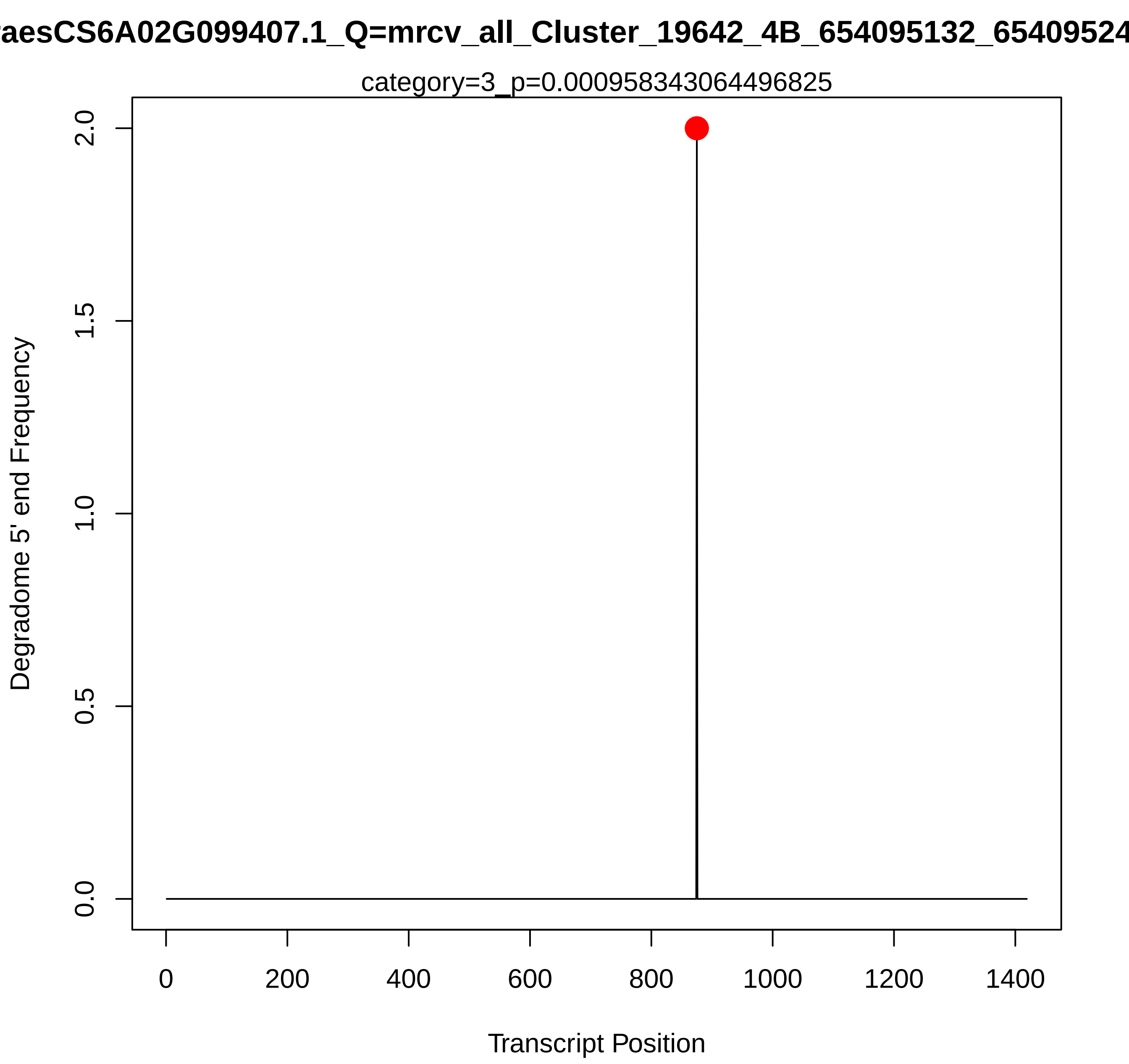
aesCS6A02G099407.1_Q=mrcv_all_Cluster_19642_4B_654095132_65409524
category=3_p=0.000958343064496825
Degradome 5' end Frequency
0.0
0.5
1.0
1.5
2.0
0
200
400
600
800
1000
1200
1400
Transcript Position

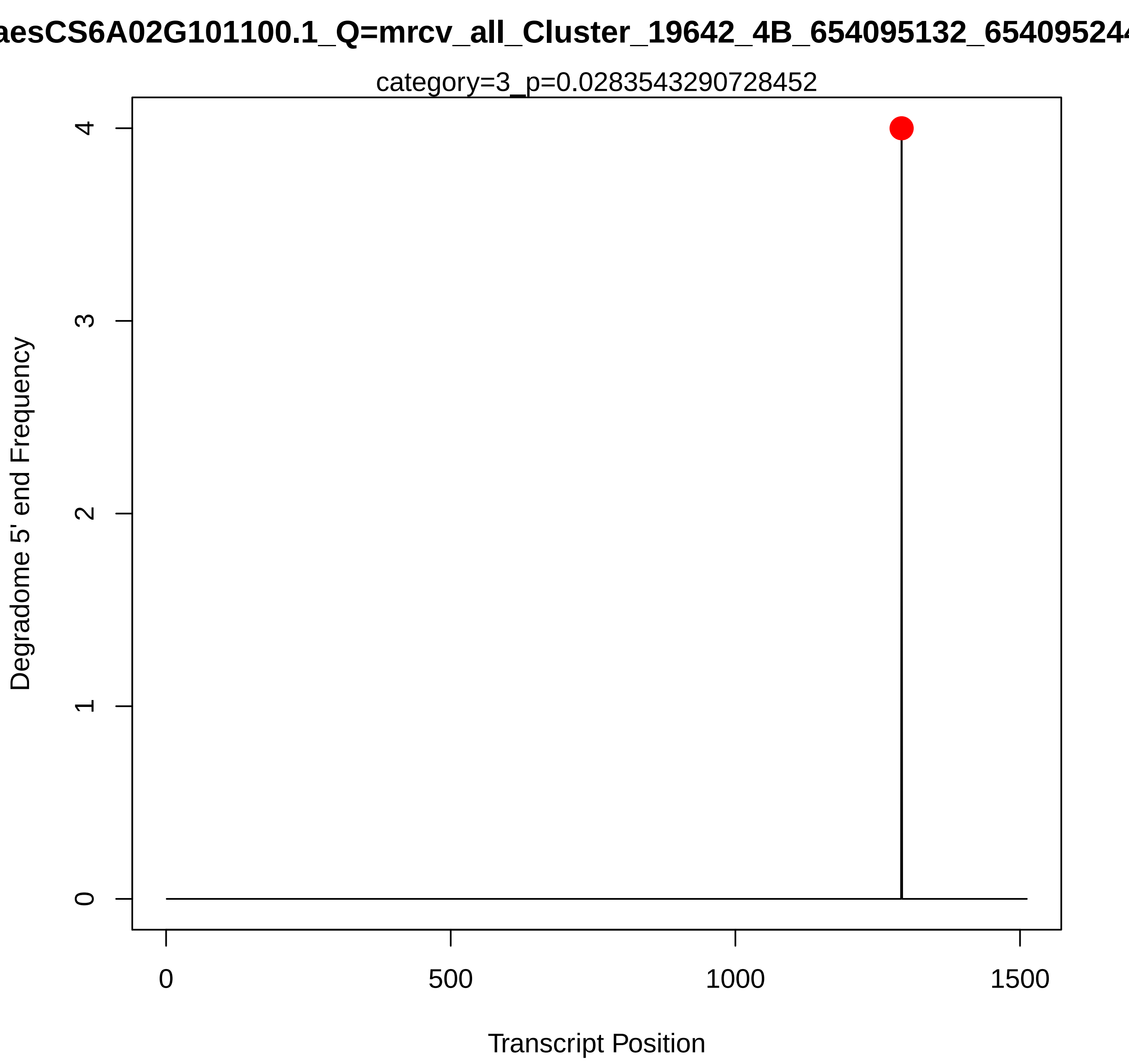

aesCS6A02G101100.1_Q=mrcv_all_Cluster_19642_4B_654095132_65409524
category=3_p=0.0283543290728452
Degradome 5' end Frequency
0
1
2
3
4
0
500
1000
1500
Transcript Position

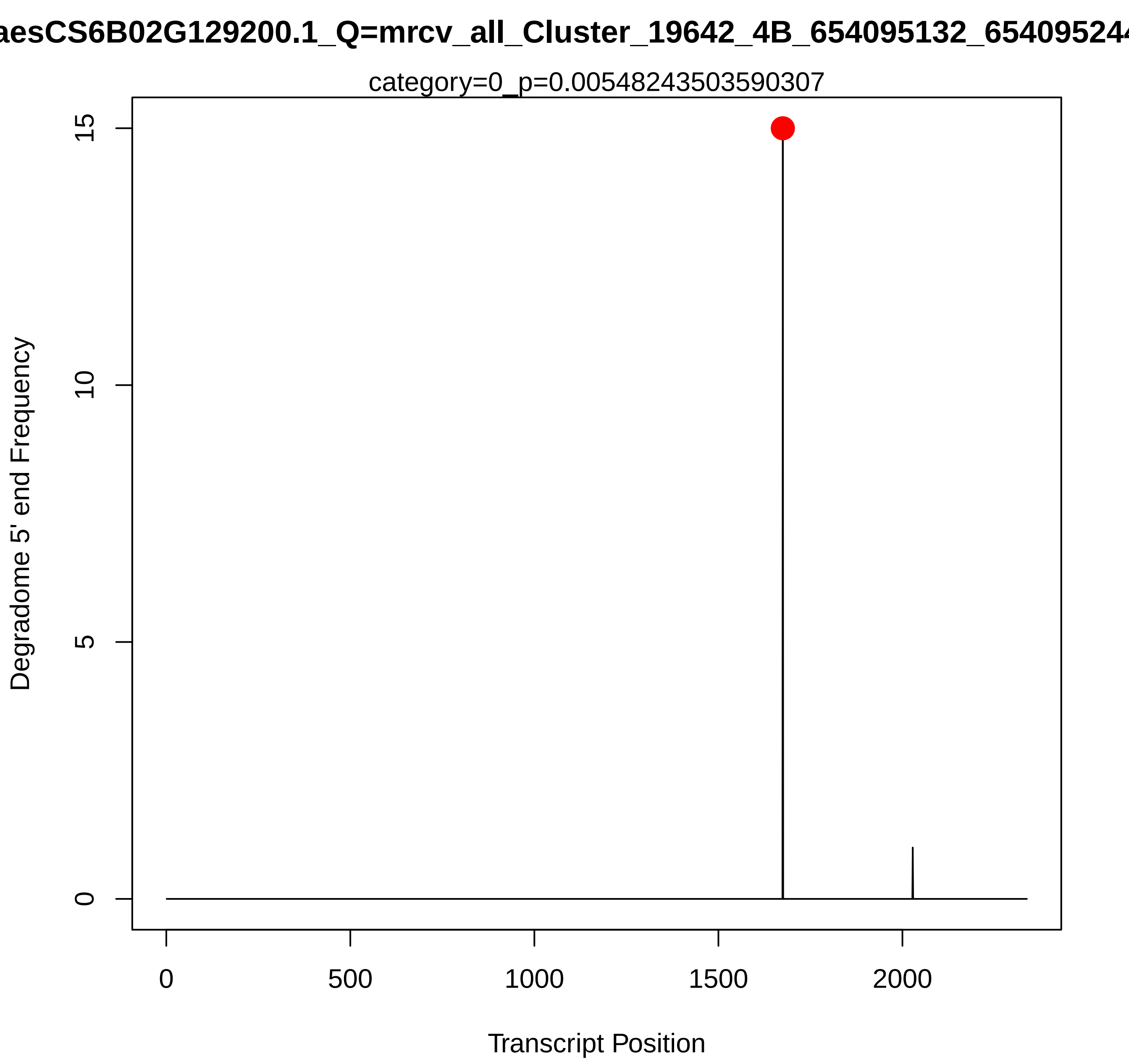
aesCS6B02G129200.1_Q=mrcv_all_Cluster_19642_4B_654095132_65409524
category=0_p=0.00548243503590307
Degradome 5' end Frequency
0
5
10
15
0
500
1000
1500
2000
Transcript Position

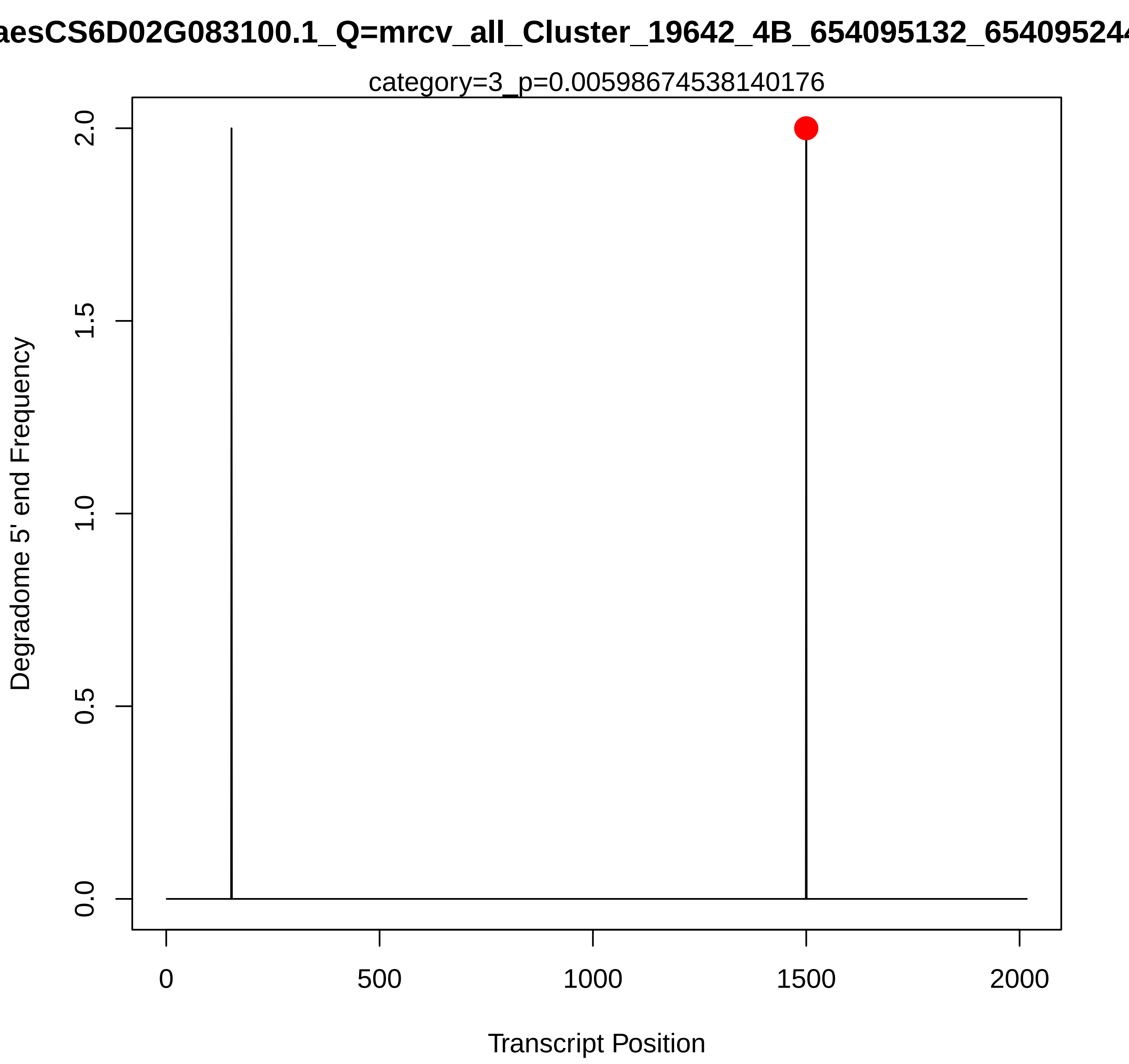
aesCS6D02G083100.1_Q=mrcv_all_Cluster_19642_4B_654095132_65409524
category=3_p=0.00598674538140176
Degradome 5' end Frequency
0.0
0.5
1.0
1.5
2.0
0
500
1000
1500
2000
Transcript Position

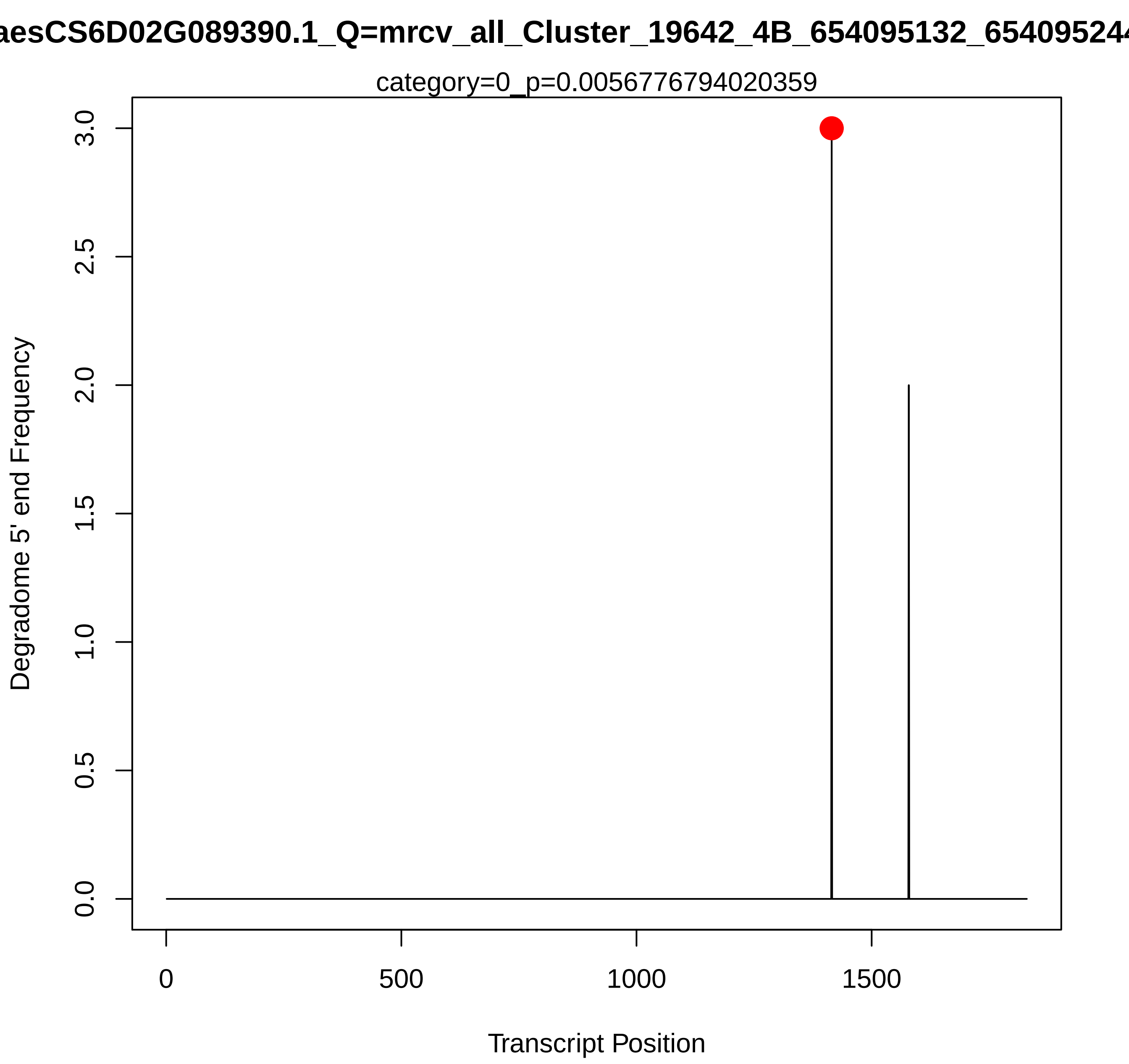
aesCS6D02G089390.1_Q=mrcv_all_Cluster_19642_4B_654095132_65409524
category=0_p=0.0056776794020359
Degradome 5' end Frequency
0.0
0.5
1.0
1.5
2.0
2.5
3.0
0
500
1000
1500
Transcript Position

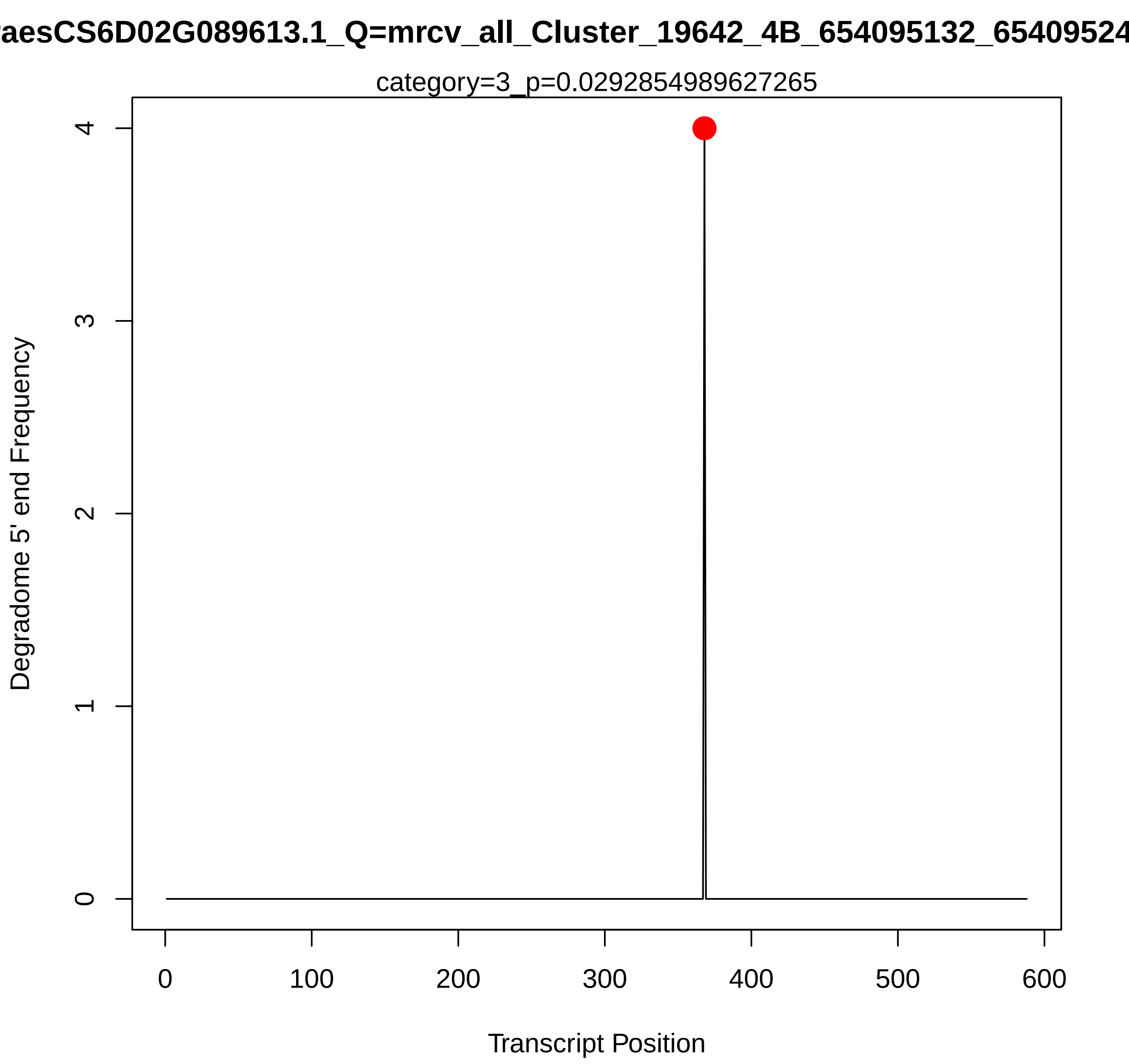
aesCS6D02G089613.1_Q=mrcv_all_Cluster_19642_4B_654095132_65409524
category=3_p=0.0292854989627265
Degradome 5' end Frequency
0
1
2
3
4
0
100
200
300
400
500
600
Transcript Position

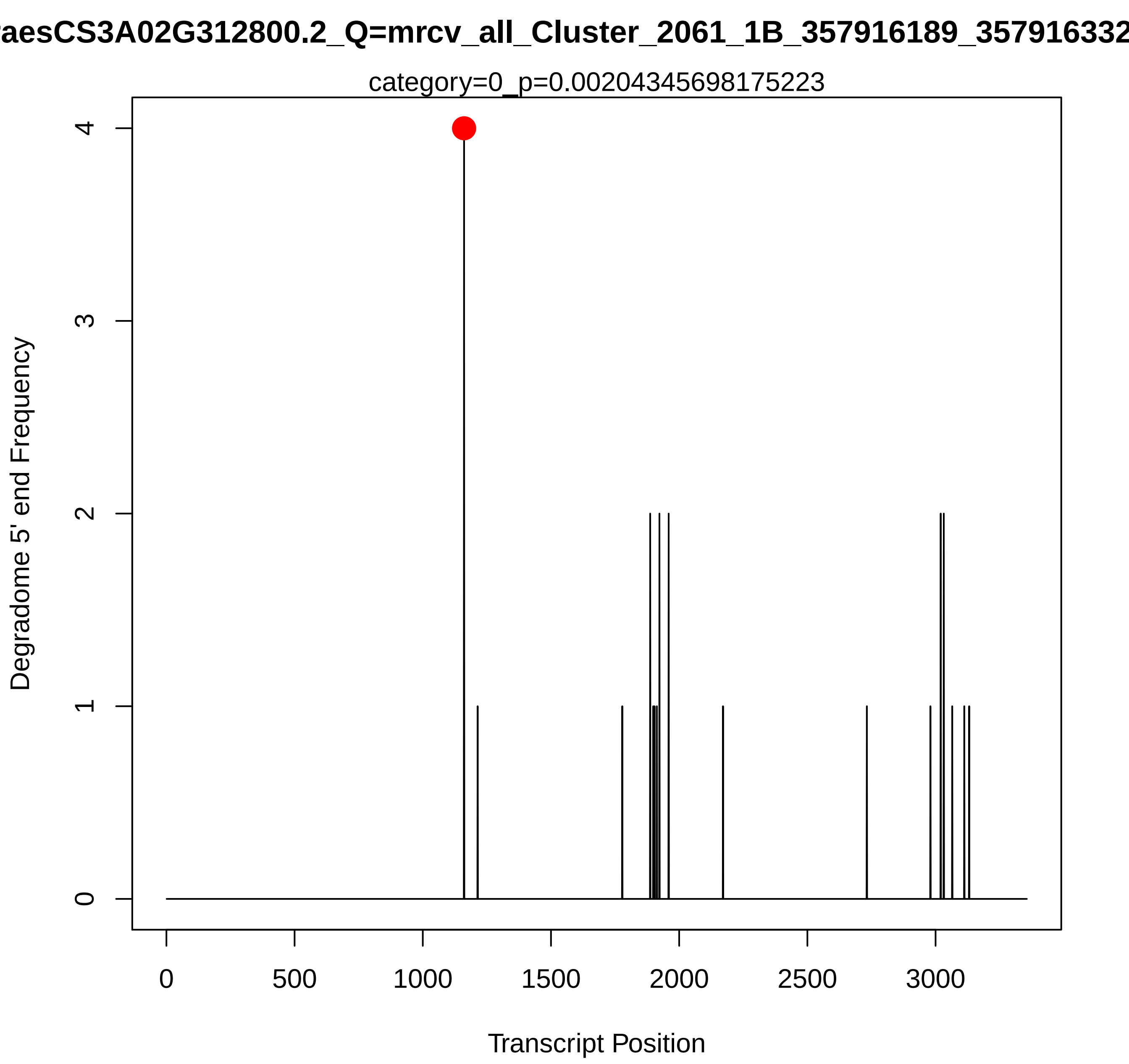
aesCS3A02G312800.2_Q=mrcv_all_Cluster_2061_1B_357916189_35791633
category=0_p=0.00204345698175223
Degradome 5' end Frequency
0
1
2
3
4
0
500
1000
1500
2000
2500
3000
Transcript Position

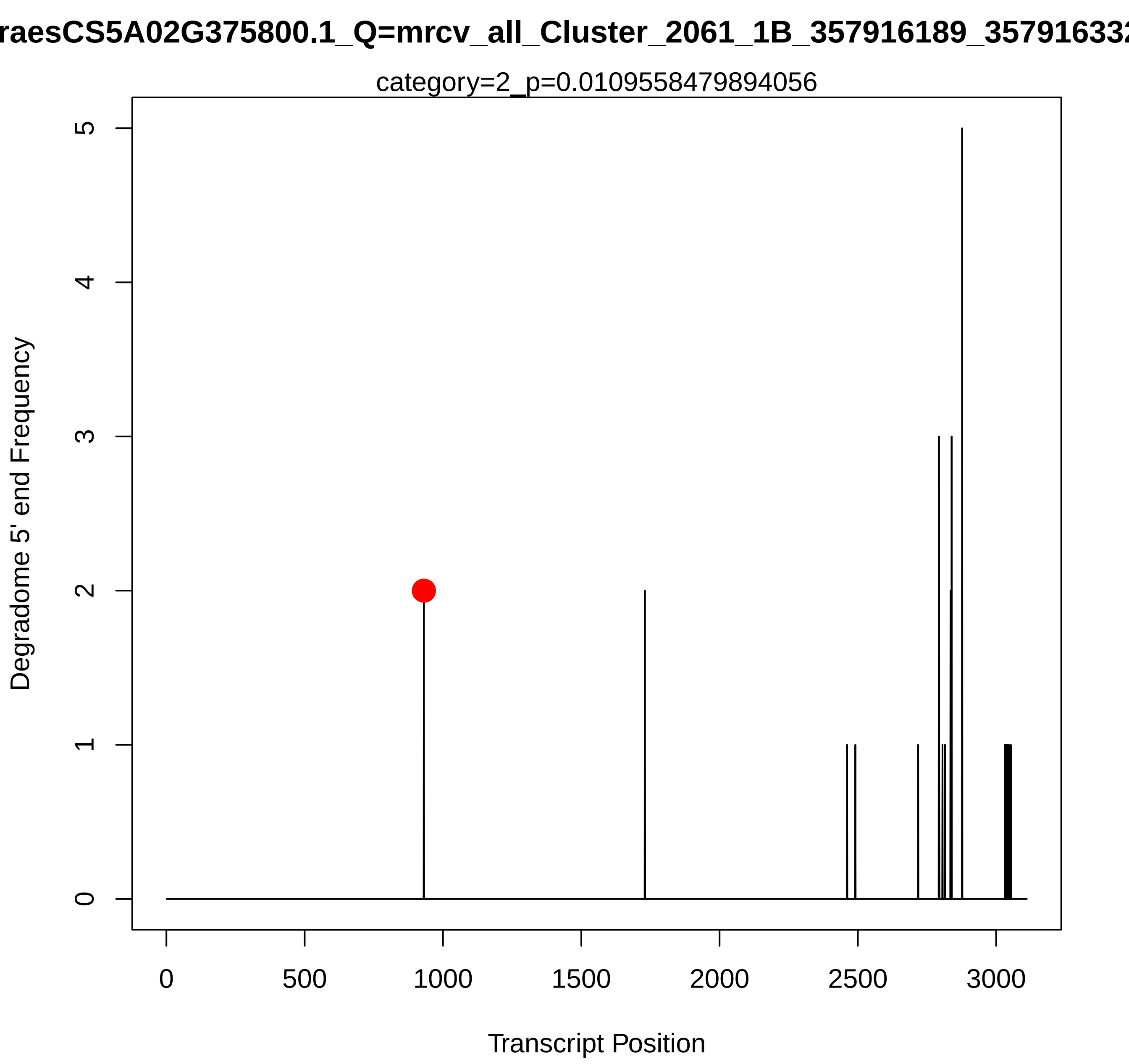
raesCS5A02G375800.1_Q=mrcv_all_Cluster_2061_1B_357916189_35791633
category=2_p=0.0109558479894056
Degradome 5' end Frequency
0
1
2
3
4
5
0
500
1000
1500
2000
2500
3000
Transcript Position

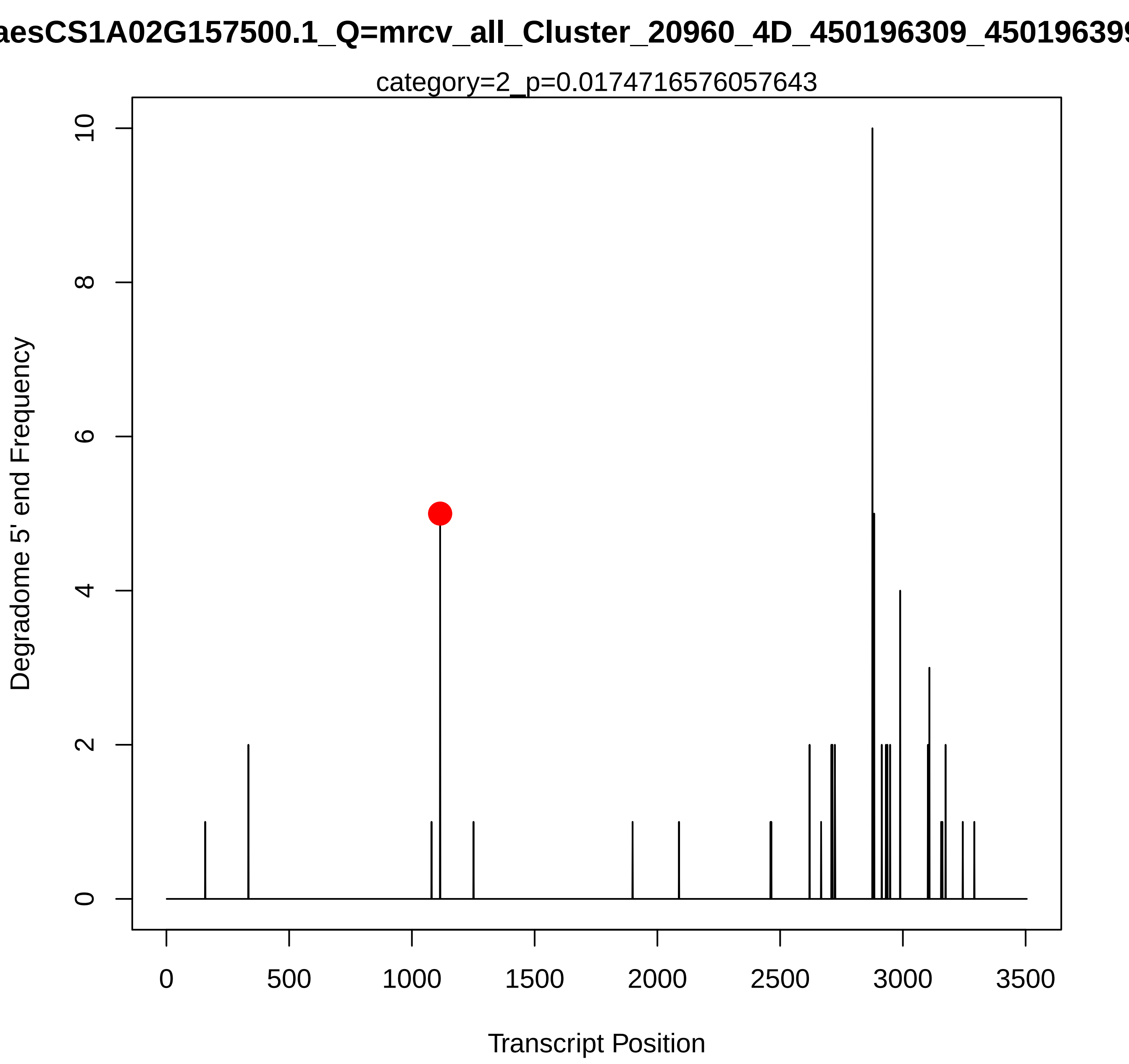
aesCS1A02G157500.1_Q=mrcv_all_Cluster_20960_4D_450196309_45019639
category=2_p=0.0174716576057643
Degradome 5' end Frequency
0
2
4
6
8
10
0
500
1000
1500
2000
2500
3000
3500
Transcript Position

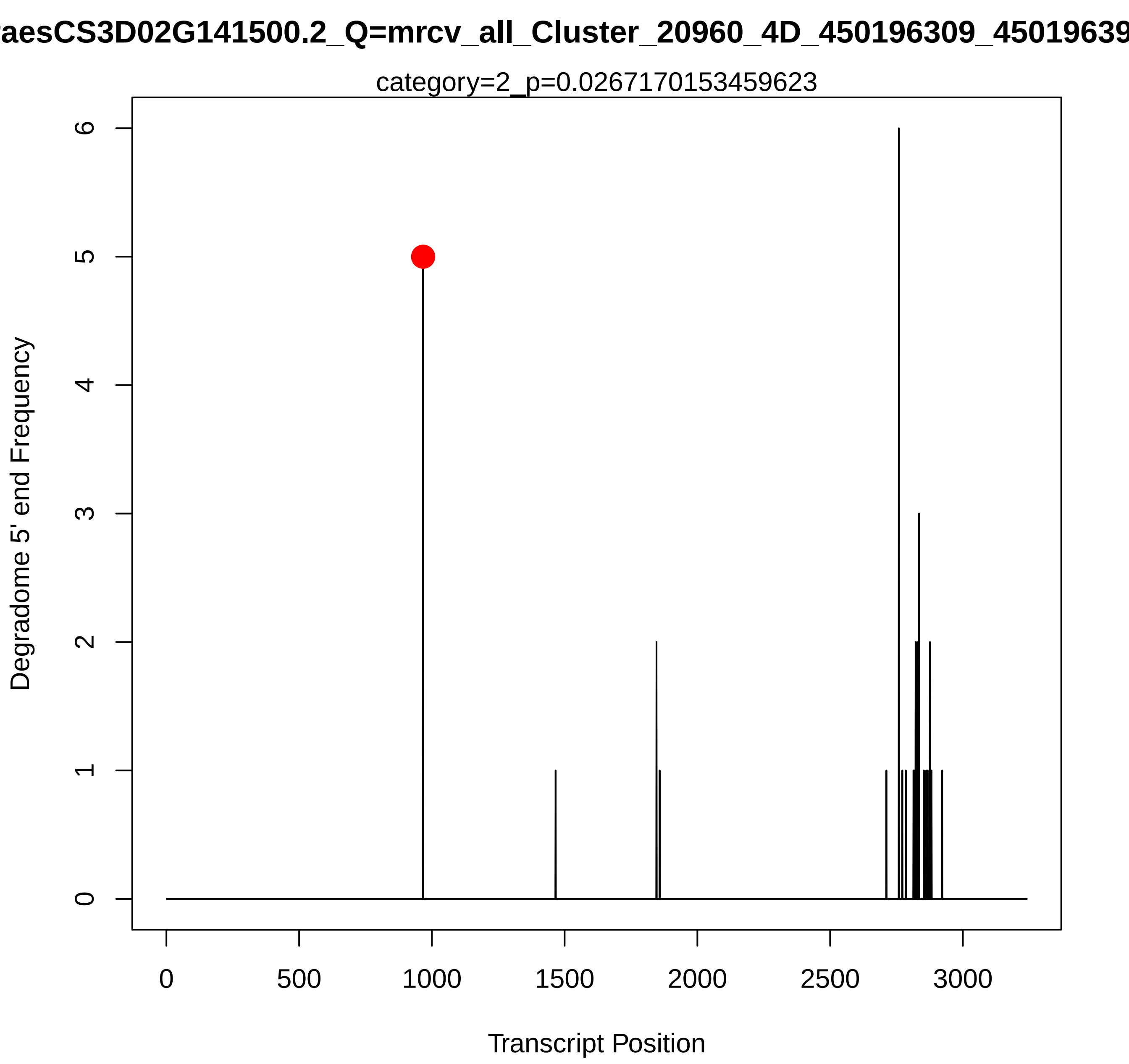

aesCS3D02G141500.2_Q=mrcv_all_Cluster_20960_4D_450196309_45019639
category=2_p=0.0267170153459623
Degradome 5' end Frequency
0
1
2
3
4
5
6
0
500
1000
1500
2000
2500
3000
Transcript Position

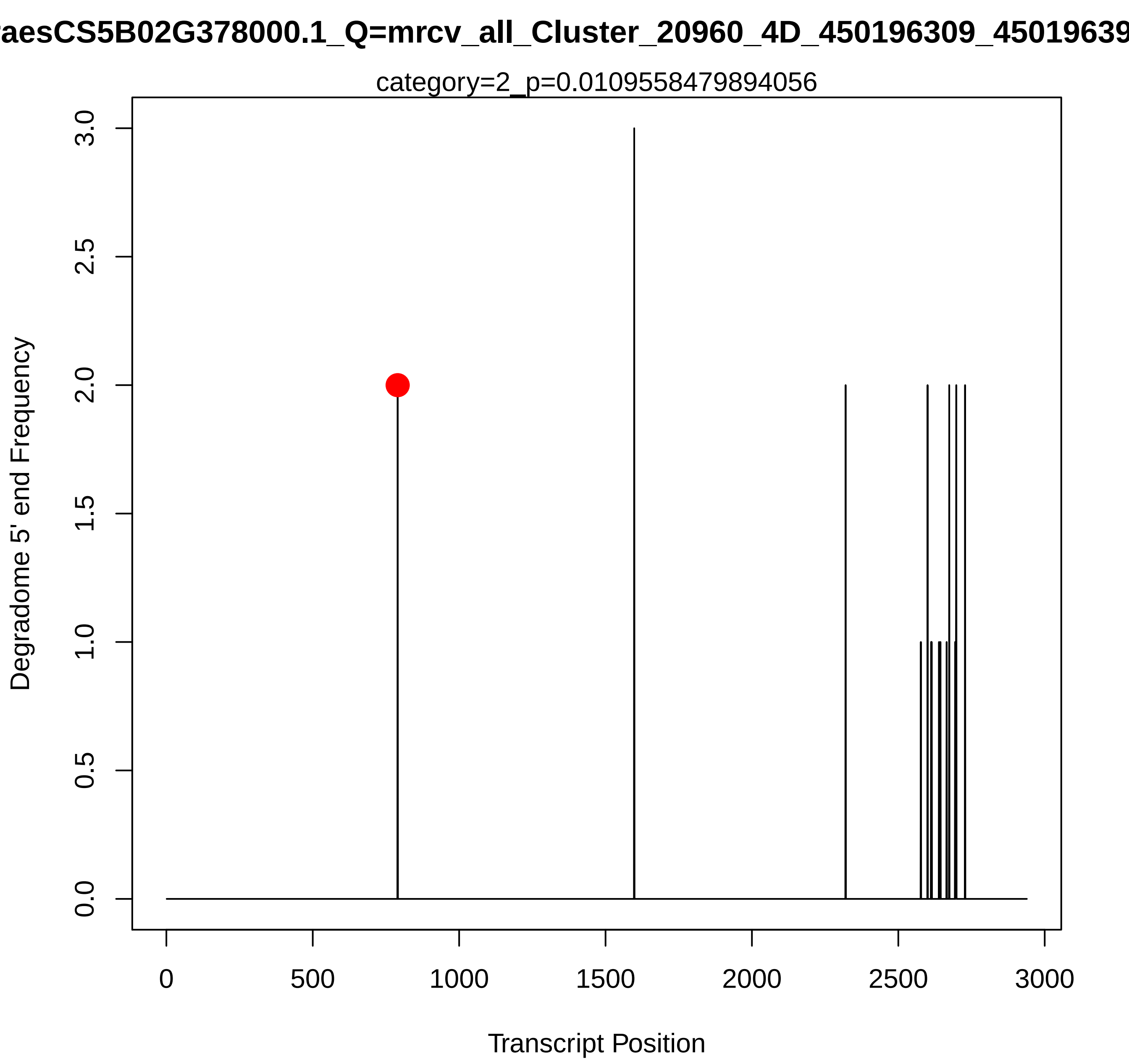
aesCS5B02G378000.1_Q=mrcv_all_Cluster_20960_4D_450196309_45019639
category=2_p=0.0109558479894056
Degradome 5' end Frequency
Transcript Position
0.0
0.5
1.0
1.5
2.0
2.5
3.0
0
500
1000
1500
2000
2500
3000

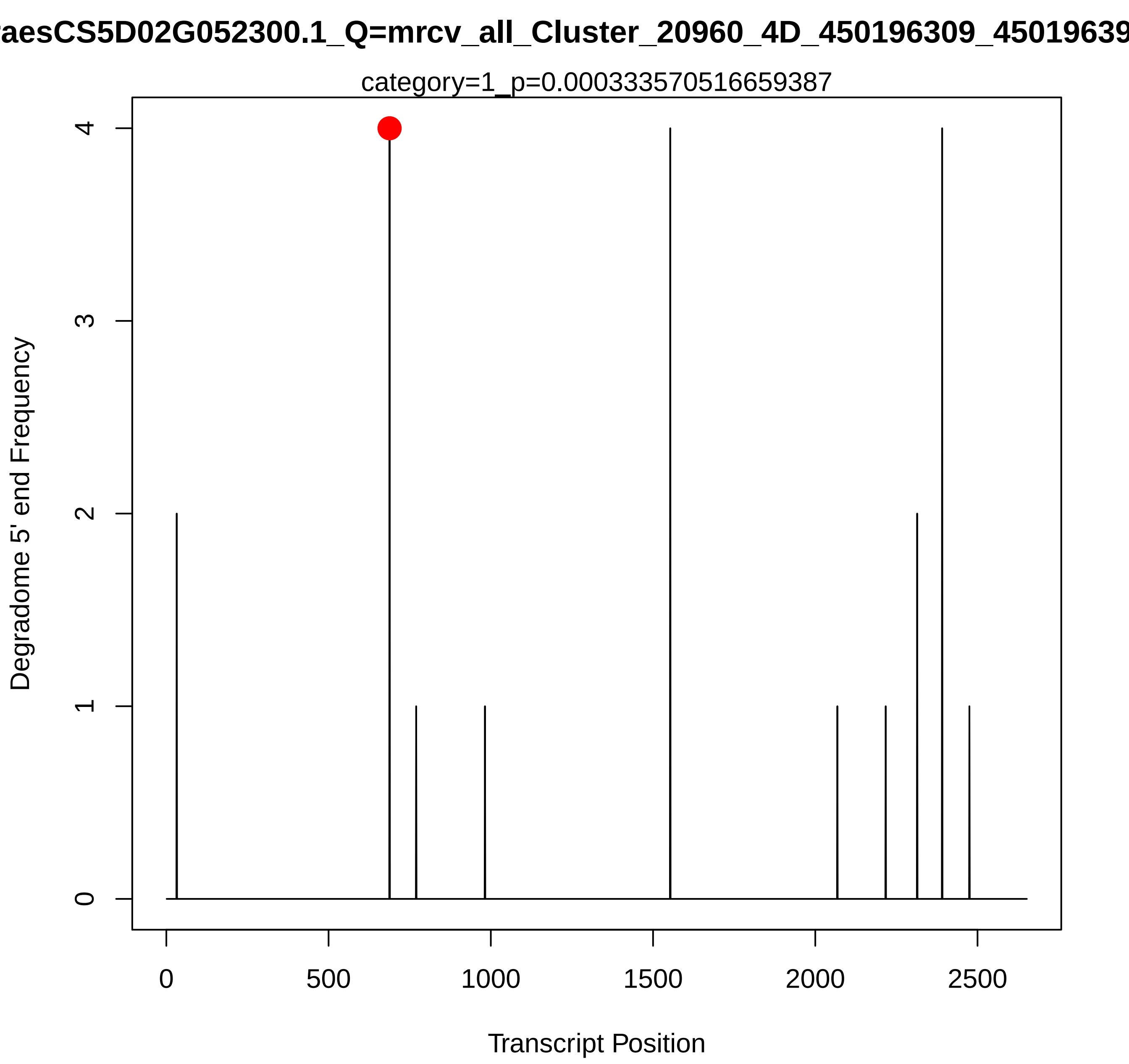

aesCS5D02G052300.1_Q=mrcv_all_Cluster_20960_4D_450196309_45019639
category=1_p=0.000333570516659387
Degradome 5' end Frequency
0
1
2
3
4
0
500
1000
1500
2000
2500
Transcript Position

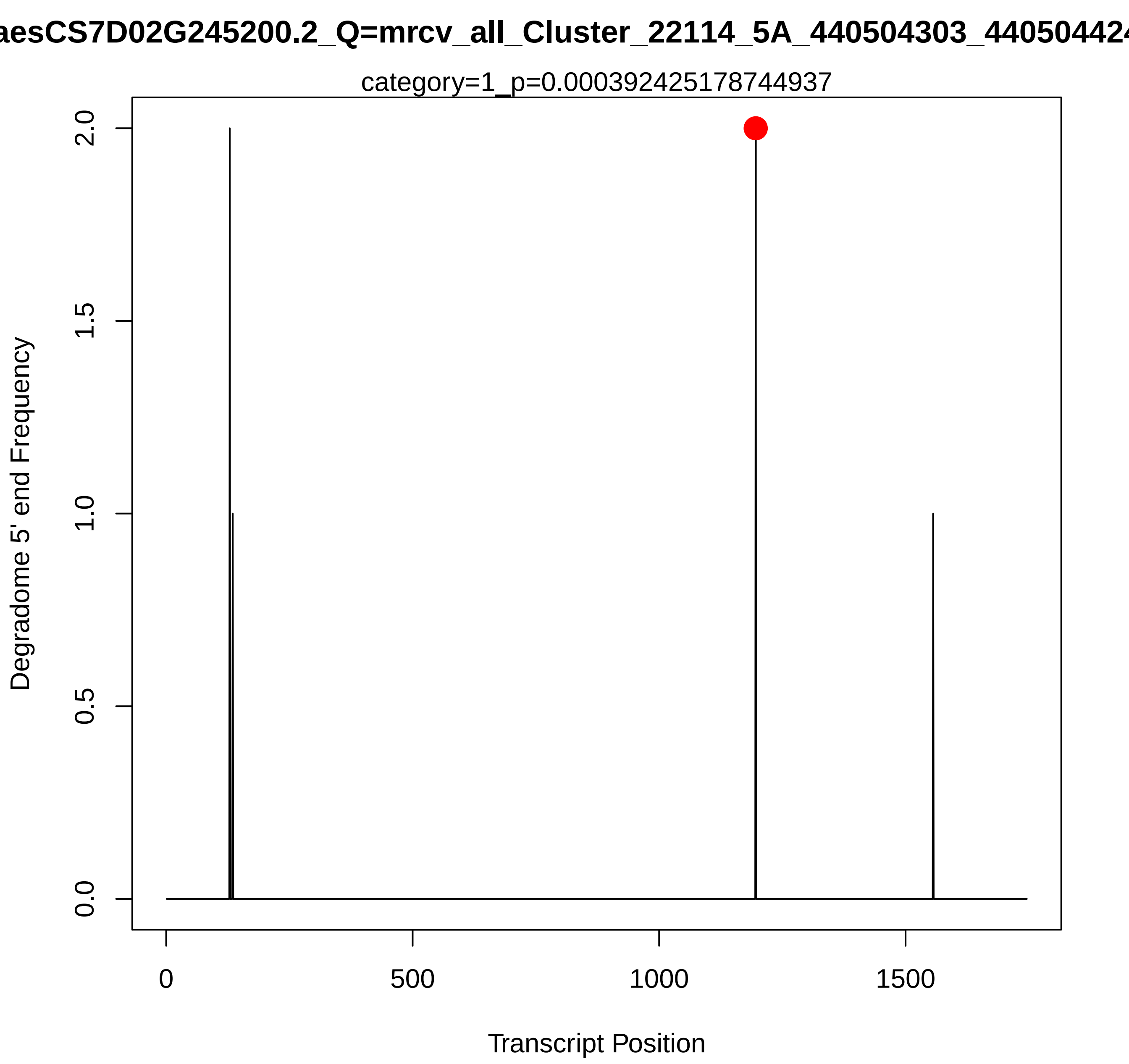
aesCS7D02G245200.2_Q=mrcv_all_Cluster_22114_5A_440504303_44050442
category=1_p=0.000392425178744937
Degradome 5' end Frequency
Transcript Position
0.0
0.5
1.0
1.5
2.0
0
500
1000
1500

aesCS1A02G157500.1_Q=mrcv_all_Cluster_22138_5A_450881182_450881290

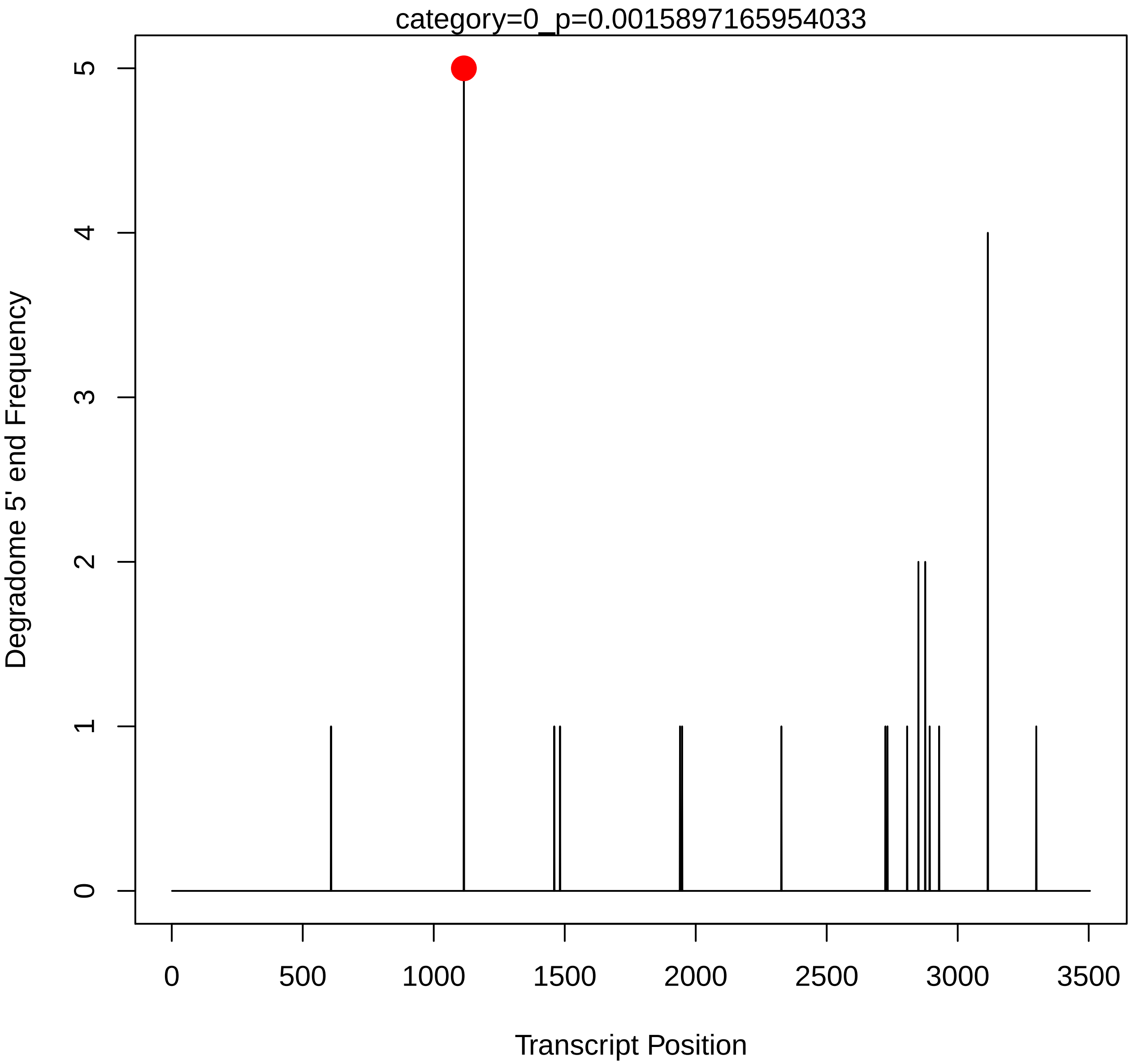

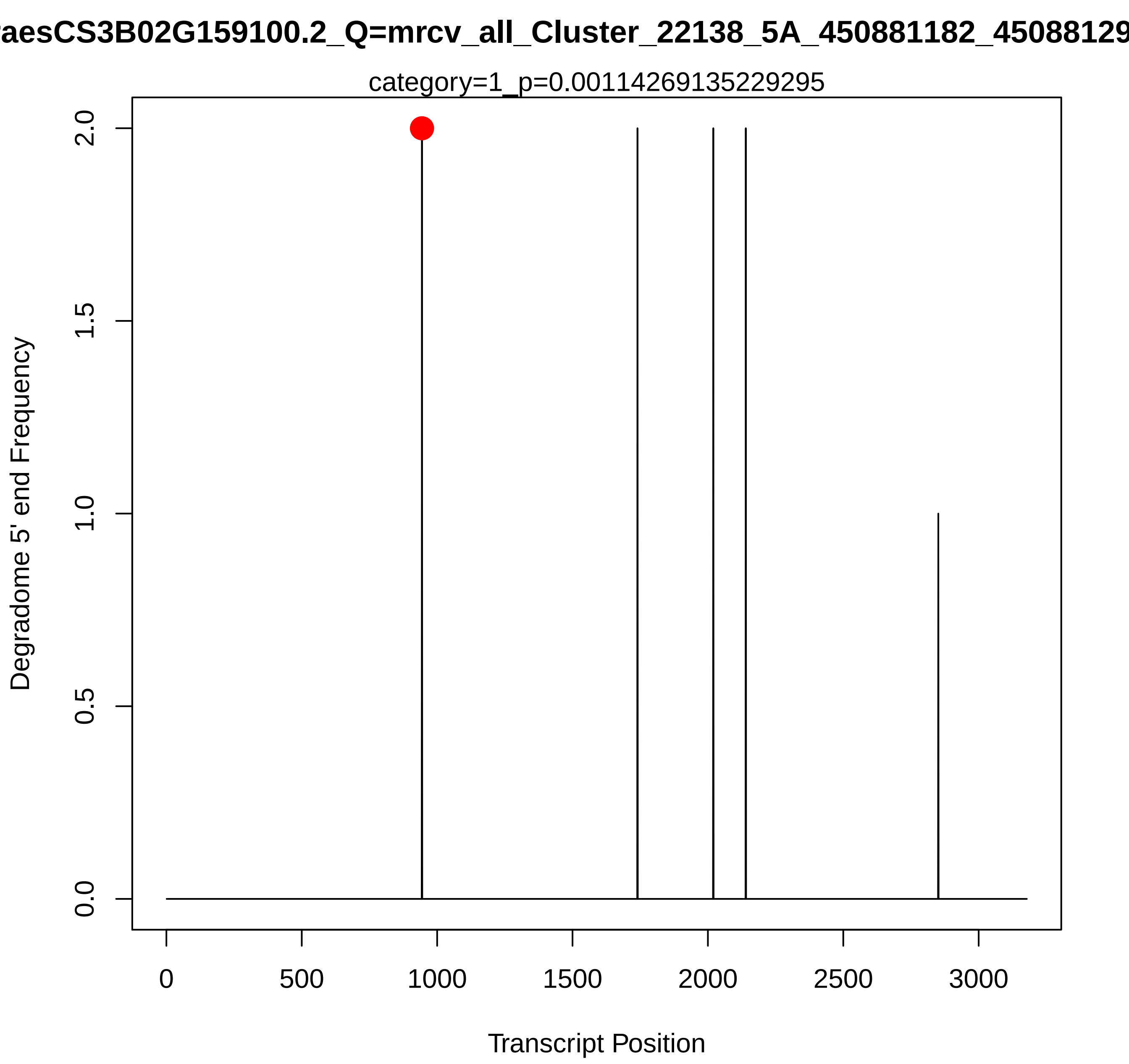

aesCS3B02G159100.2_Q=mrcv_all_Cluster_22138_5A_450881182_45088129
category=1_p=0.00114269135229295
Degradome 5' end Frequency
0.0
0.5
1.0
1.5
2.0
0
500
1000
1500
2000
2500
3000
Transcript Position

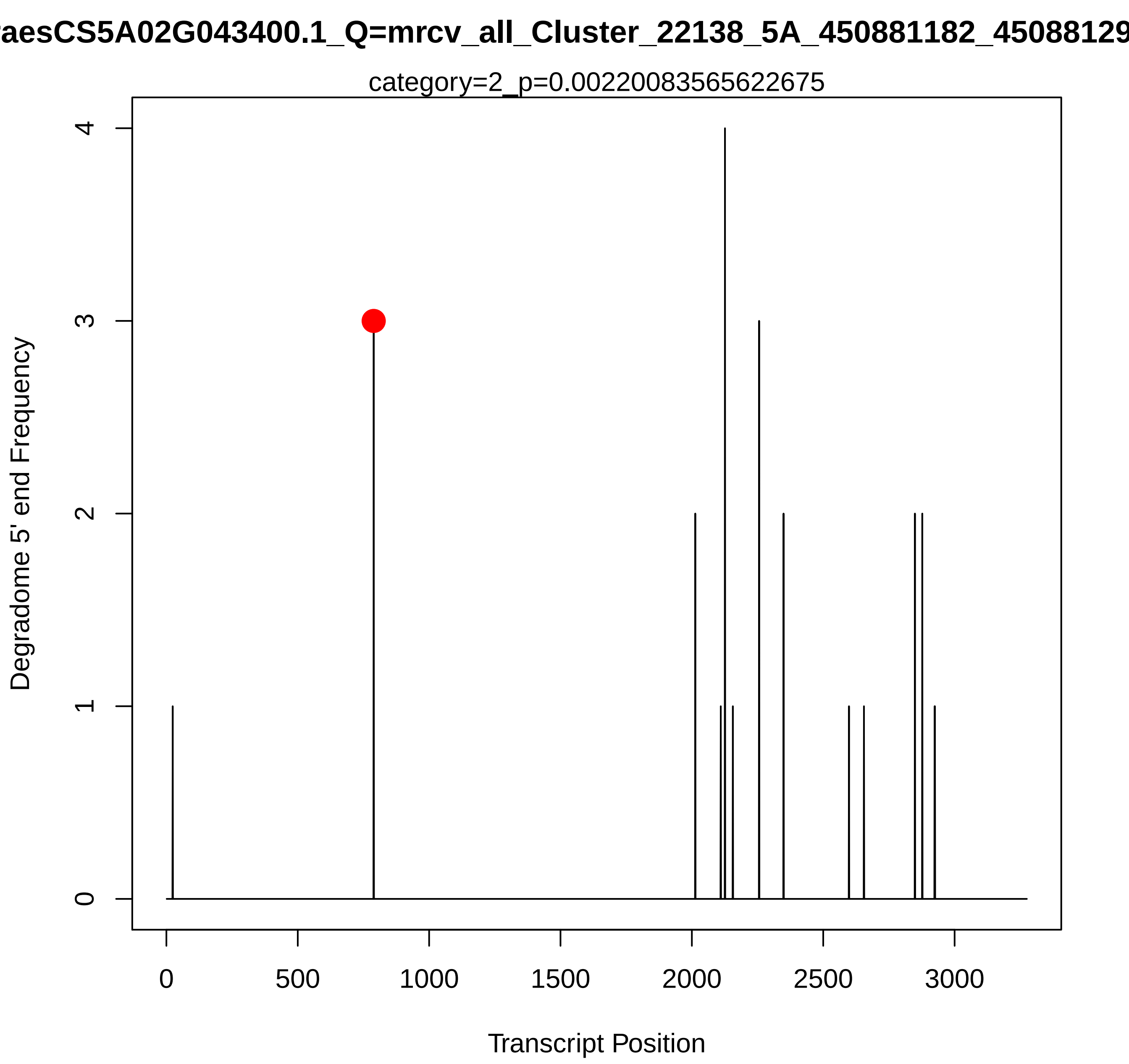

aesCS5A02G043400.1_Q=mrcv_all_Cluster_22138_5A_450881182_45088129
category=2_p=0.00220083565622675
Degradome 5' end Frequency
0
1
2
3
4
0
500
1000
1500
2000
2500
3000
Transcript Position

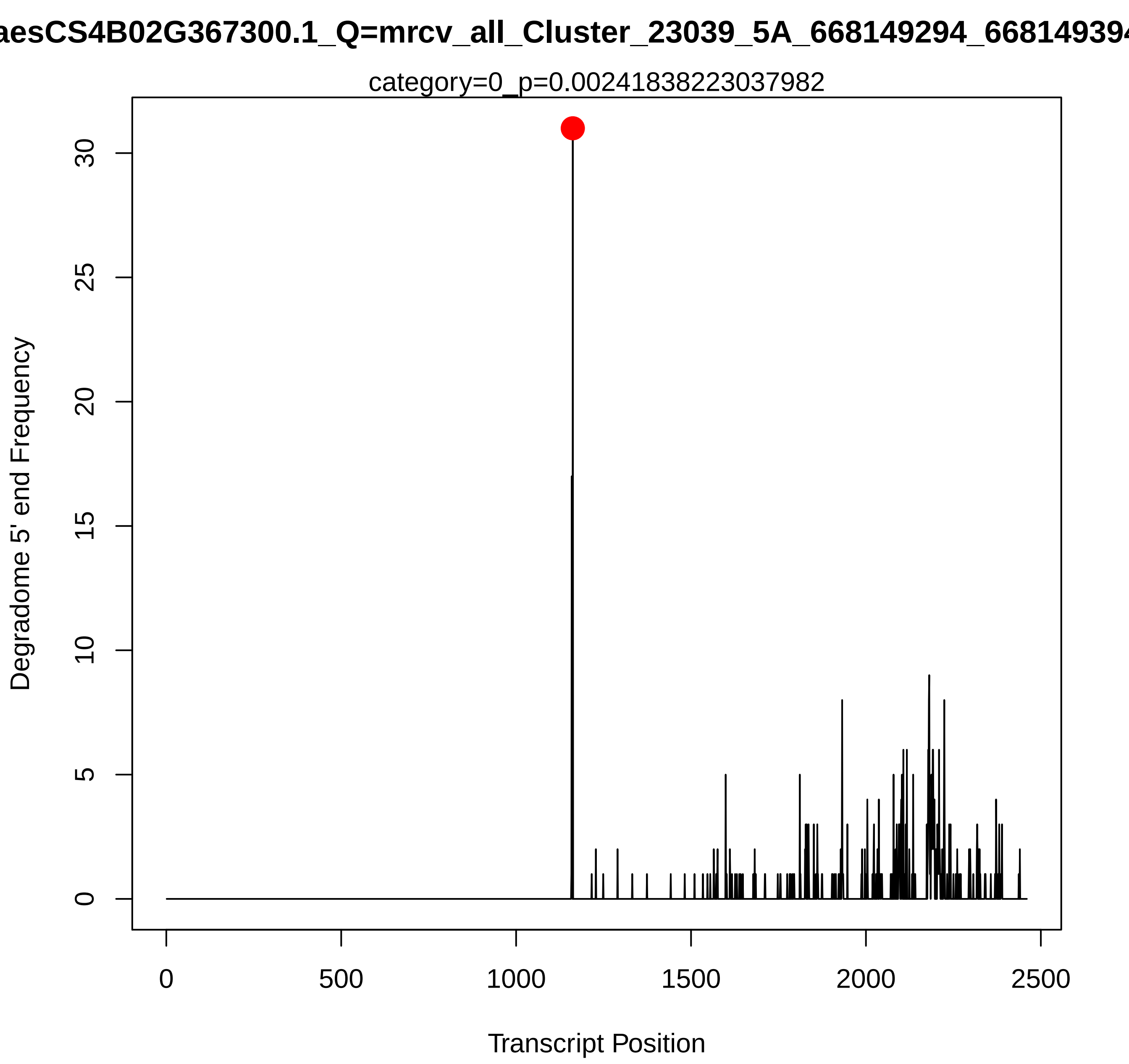
aesCS4B02G367300.1_Q=mrcv_all_Cluster_23039_5A_668149294_66814939
category=0_p=0.00241838223037982
Degradome 5' end Frequency
Transcript Position
0
5
10
15
20
25
30
0
500
1000
1500
2000
2500

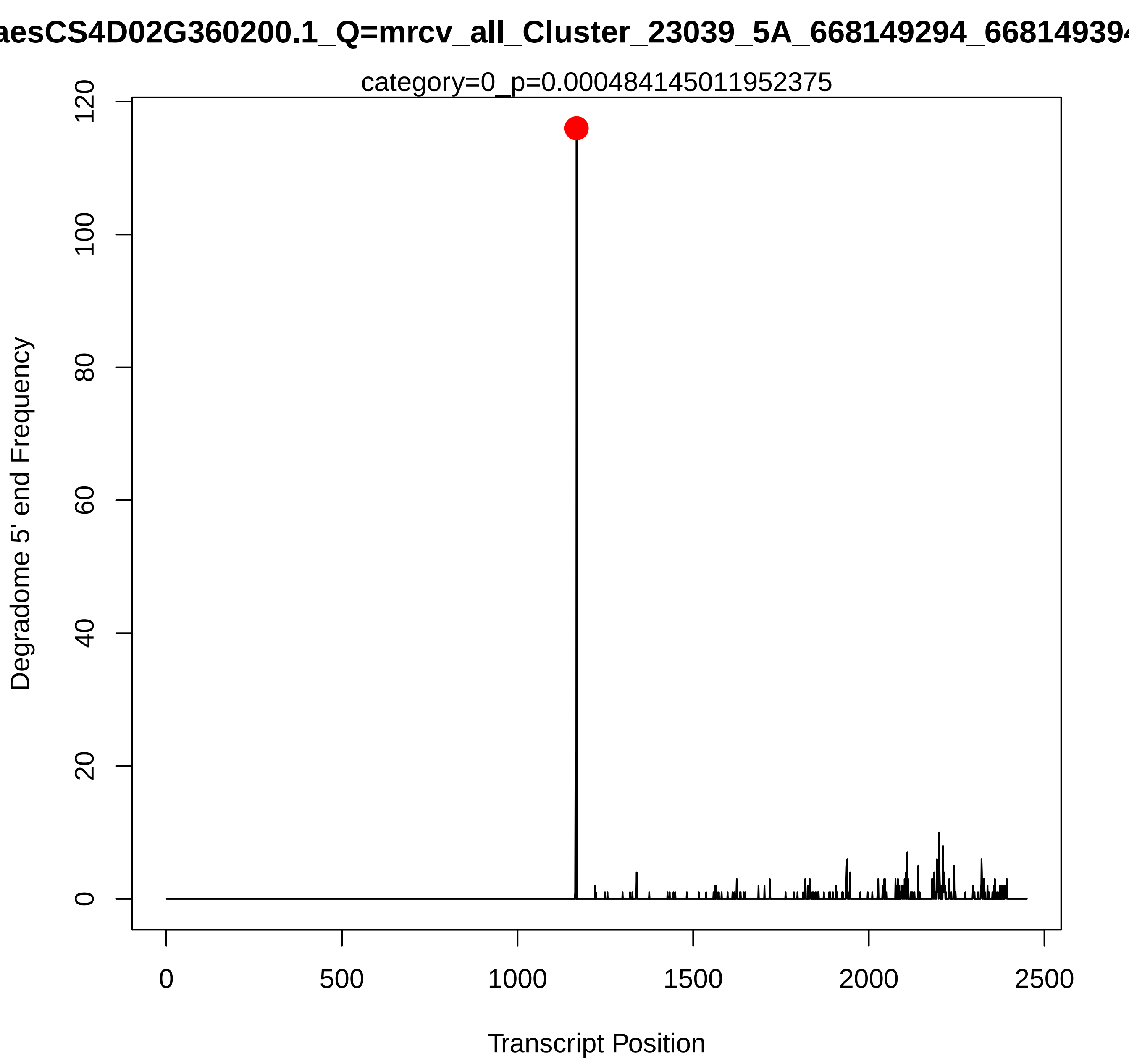

aesCS4D02G360200.1_Q=mrcv_all_Cluster_23039_5A_668149294_66814939
category=0_p=0.000484145011952375
Degradome 5' end Frequency
Transcript Position
0
20
40
60
80
100
120
0
500
1000
1500
2000
2500

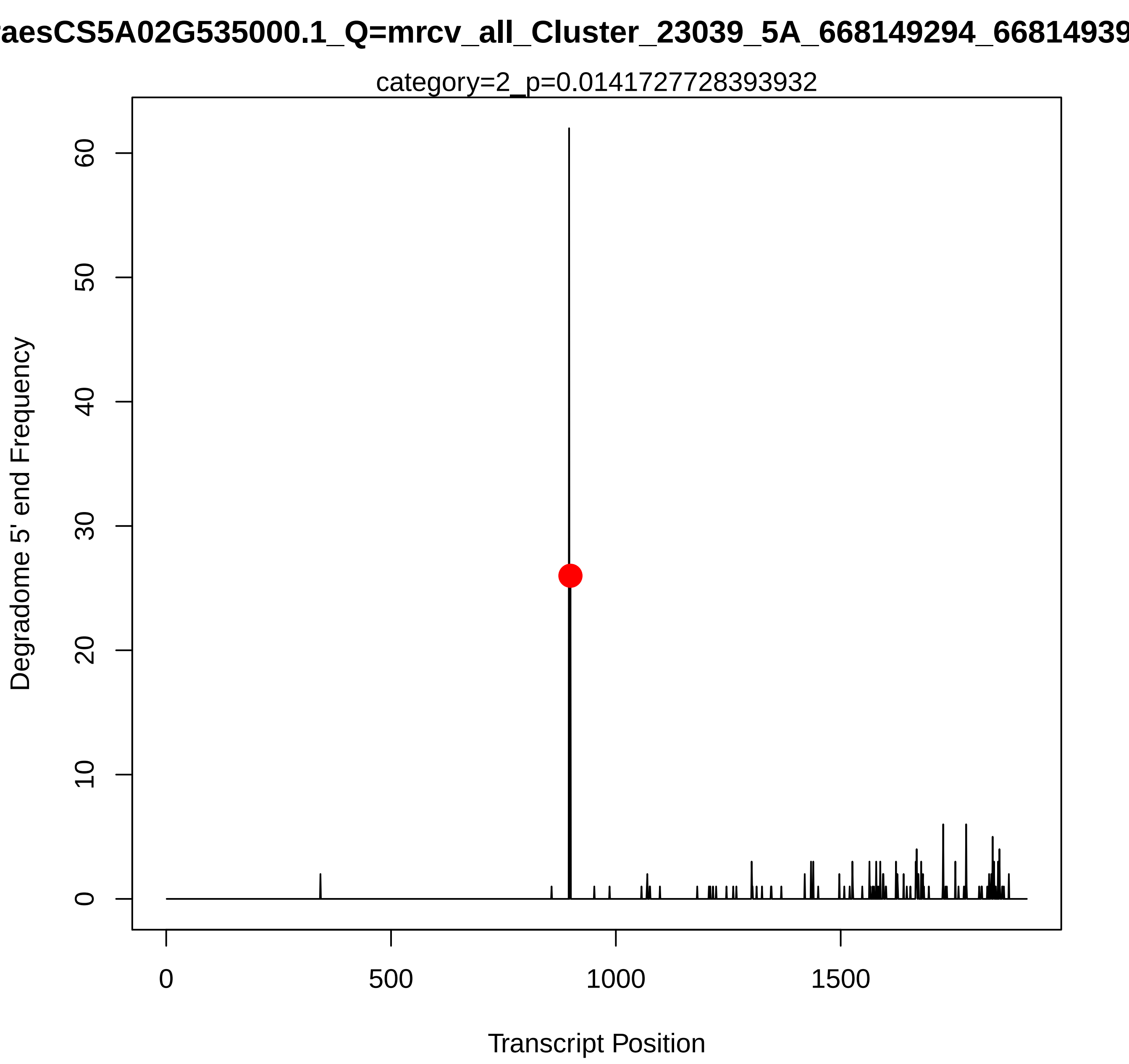

aesCS5A02G535000.1_Q=mrcv_all_Cluster_23039_5A_668149294_66814939
category=2_p=0.0141727728393932
Degradome 5' end Frequency
0
10
20
30
40
50
60
0
500
1000
1500
Transcript Position

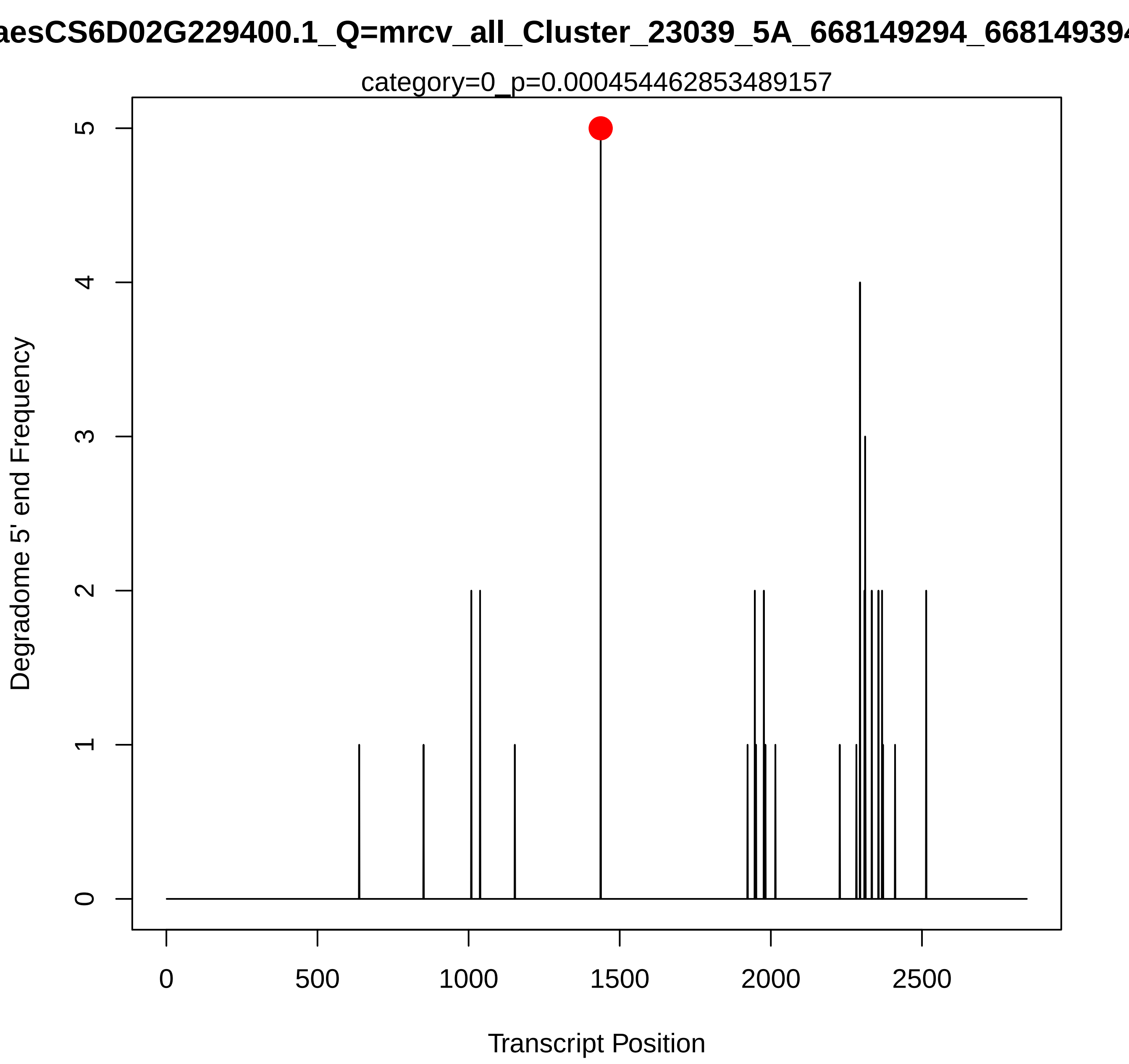
aesCS6D02G229400.1_Q=mrcv_all_Cluster_23039_5A_668149294_66814939
category=0_p=0.000454462853489157
Degradome 5' end Frequency
0
1
2
3
4
5
0
500
1000
1500
2000
2500
Transcript Position

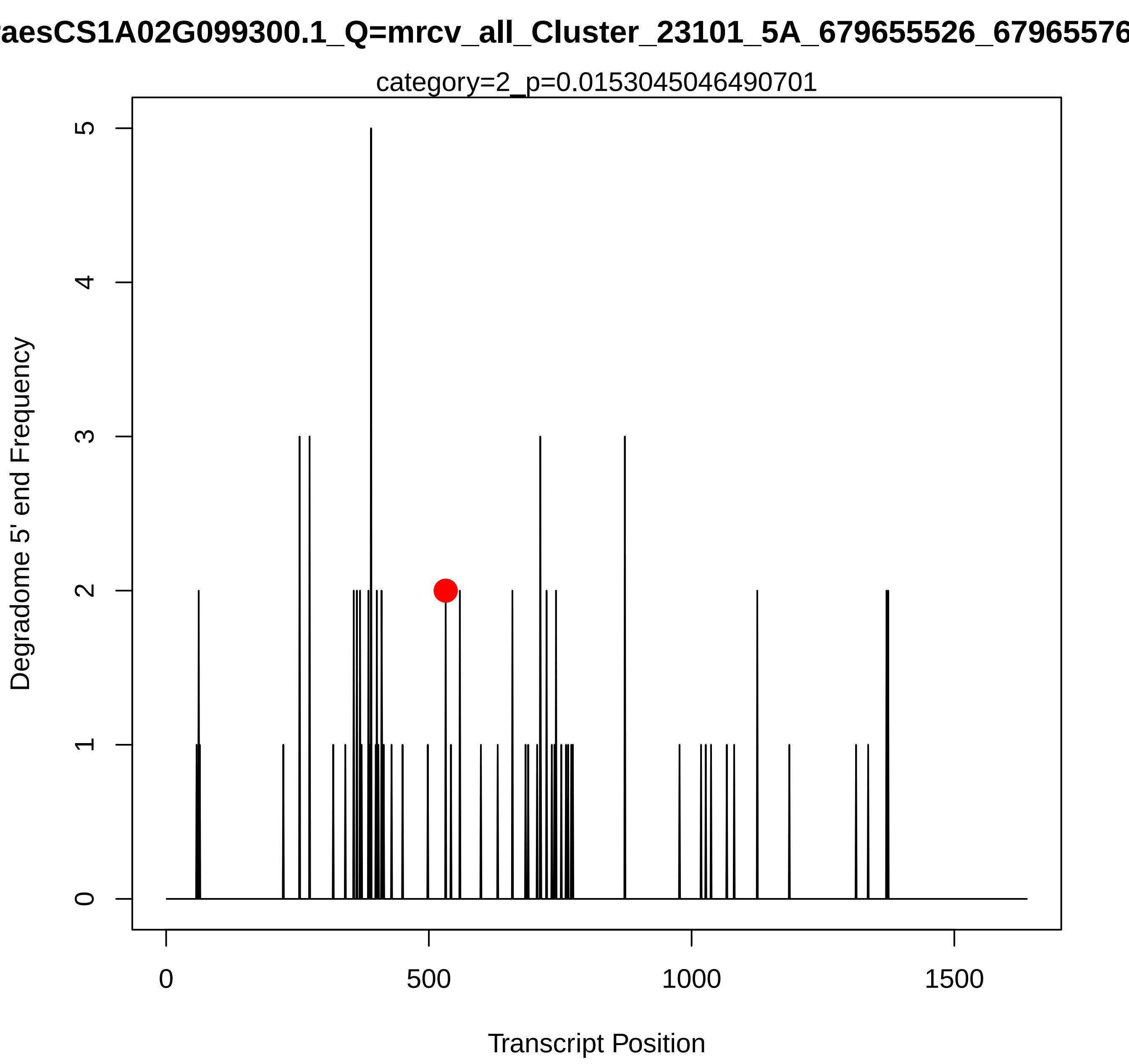
aesCS1A02G099300.1_Q=mrcv_all_Cluster_23101_5A_679655526_67965576
category=2_p=0.0153045046490701
Degradome 5' end Frequency
0
1
2
3
4
5
0
500
1000
1500
Transcript Position

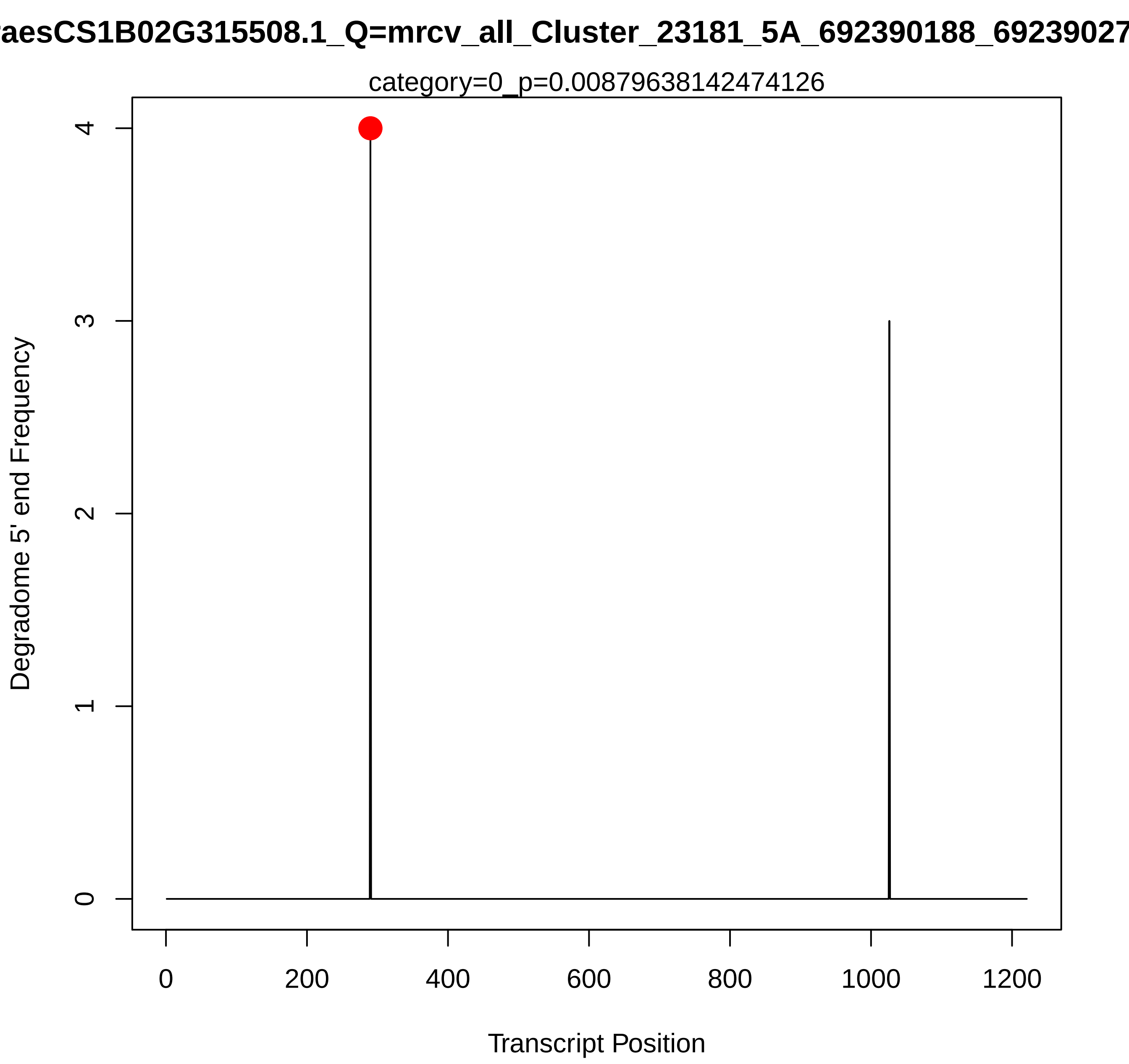
aesCS1B02G315508.1_Q=mrcv_all_Cluster_23181_5A_692390188_69239027
category=0_p=0.00879638142474126
Degradome 5' end Frequency
0
1
2
3
4
0
200
400
600
800
1000
1200
Transcript Position

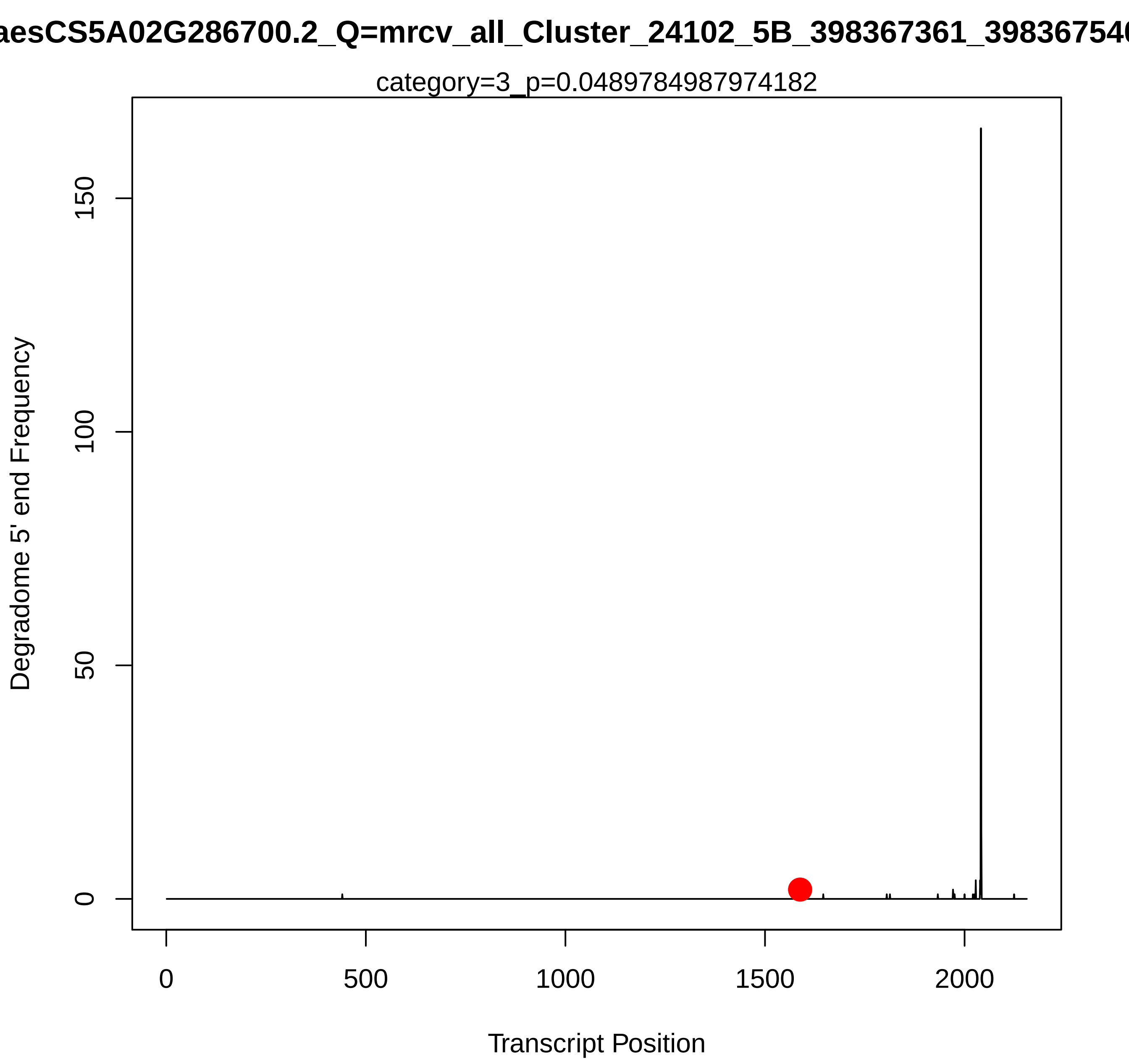
aesCS5A02G286700.2_Q=mrcv_all_Cluster_24102_5B_398367361_39836754
category=3_p=0.0489784987974182
Degradome 5' end Frequency
0
50
100
150
0
500
1000
1500
2000
Transcript Position

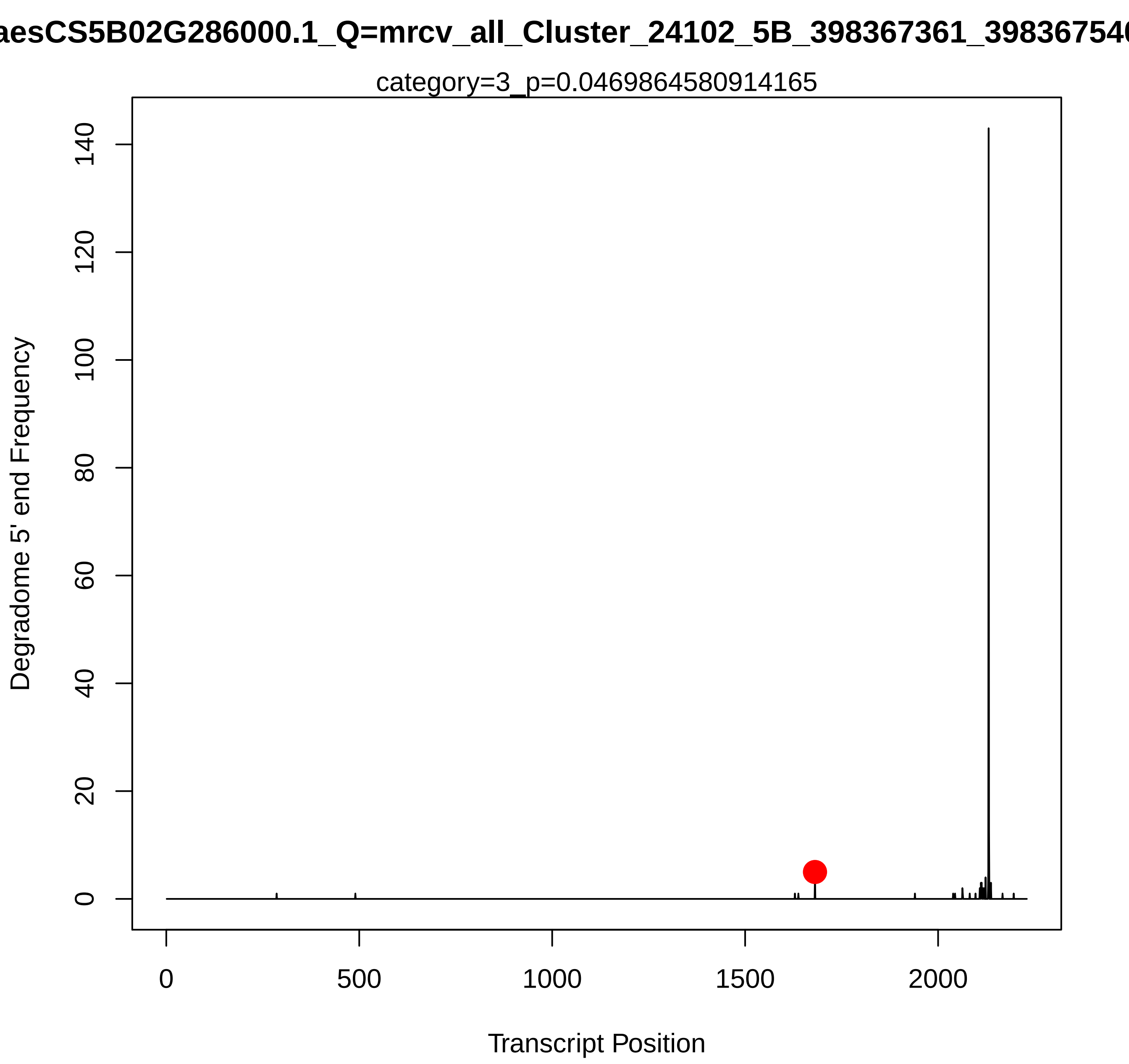
aesCS5B02G286000.1_Q=mrcv_all_Cluster_24102_5B_398367361_39836754
category=3_p=0.0469864580914165
Degradome 5' end Frequency
0
20
40
60
80
100
120
140
0
500
1000
1500
2000
Transcript Position

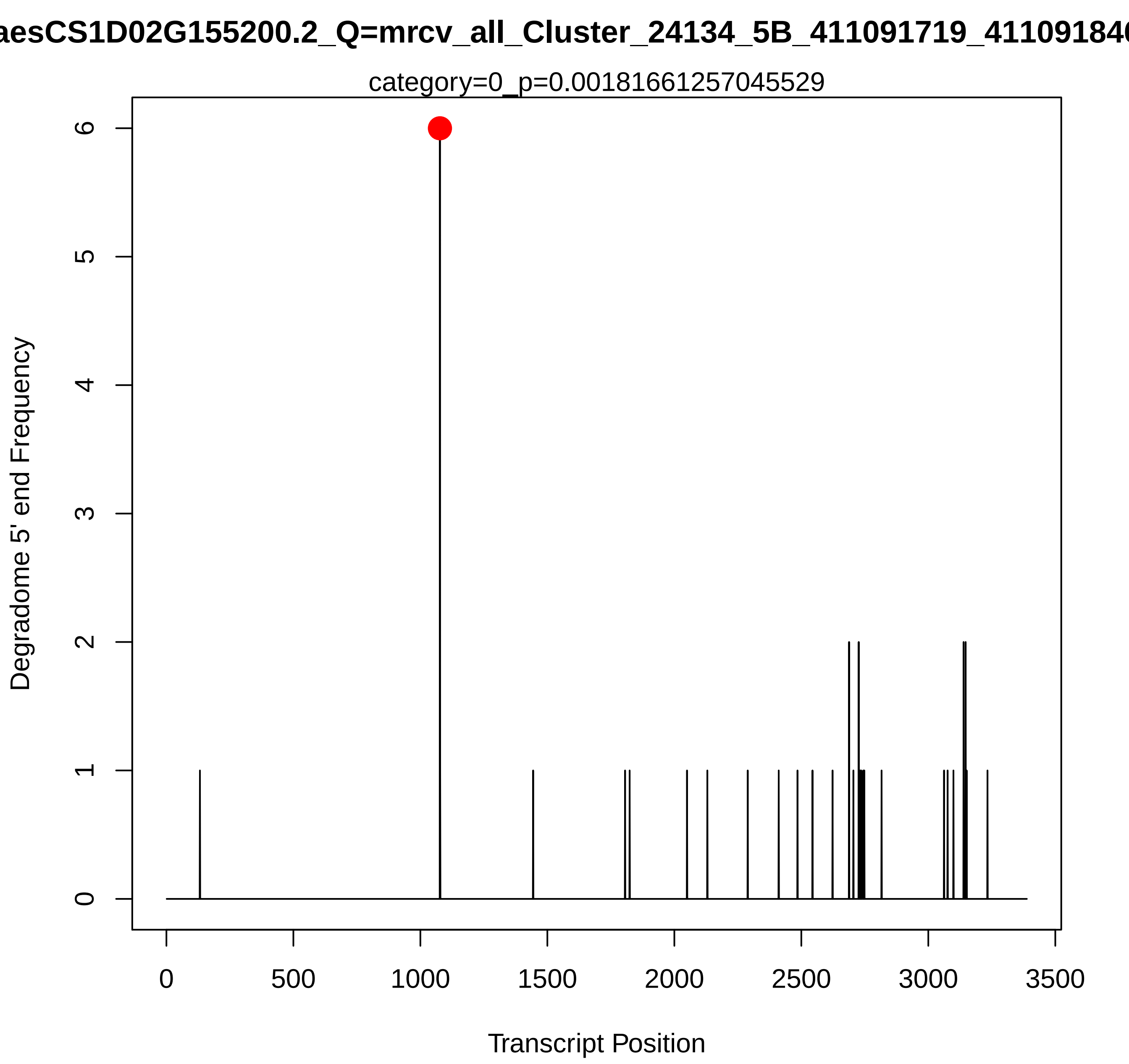
aesCS1D02G155200.2_Q=mrcv_all_Cluster_24134_5B_411091719_41109184
category=0_p=0.00181661257045529
Degradome 5' end Frequency
0
1
2
3
4
5
6
0
500
1000
1500
2000
2500
3000
3500
Transcript Position

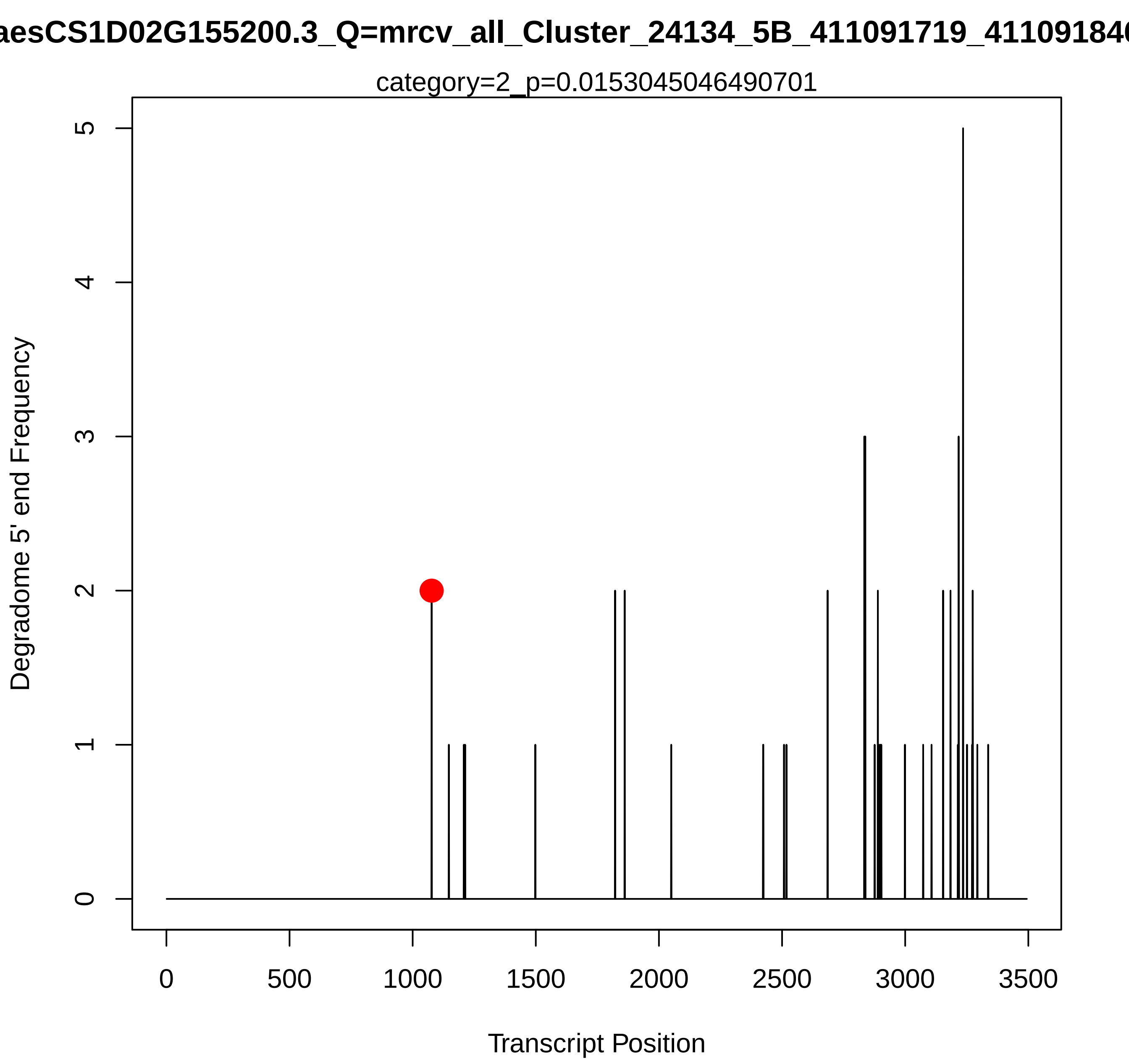
aesCS1D02G155200.3_Q=mrcv_all_Cluster_24134_5B_411091719_41109184
category=2_p=0.0153045046490701
Degradome 5' end Frequency
0
1
2
3
4
5
0
500
1000
1500
2000
2500
3000
3500
Transcript Position

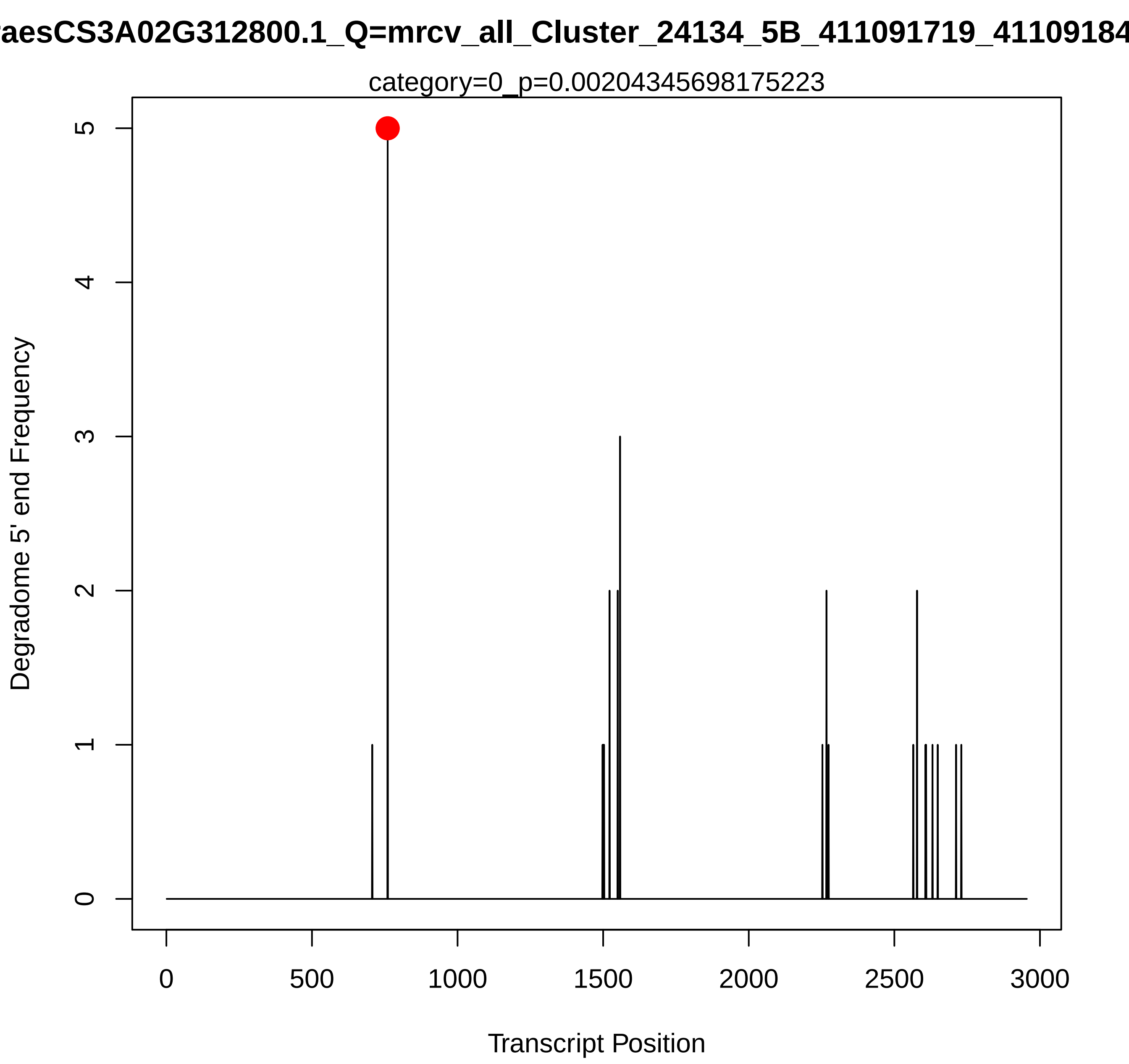
TraesCS3A02G312800.1_Q=mrcv_all_Cluster_24134_5B_411091719_41109184
category=0_p=0.00204345698175223
Degradome 5' end Frequency
Transcript Position
0
1
2
3
4
5
0
500
1000
1500
2000
2500
3000

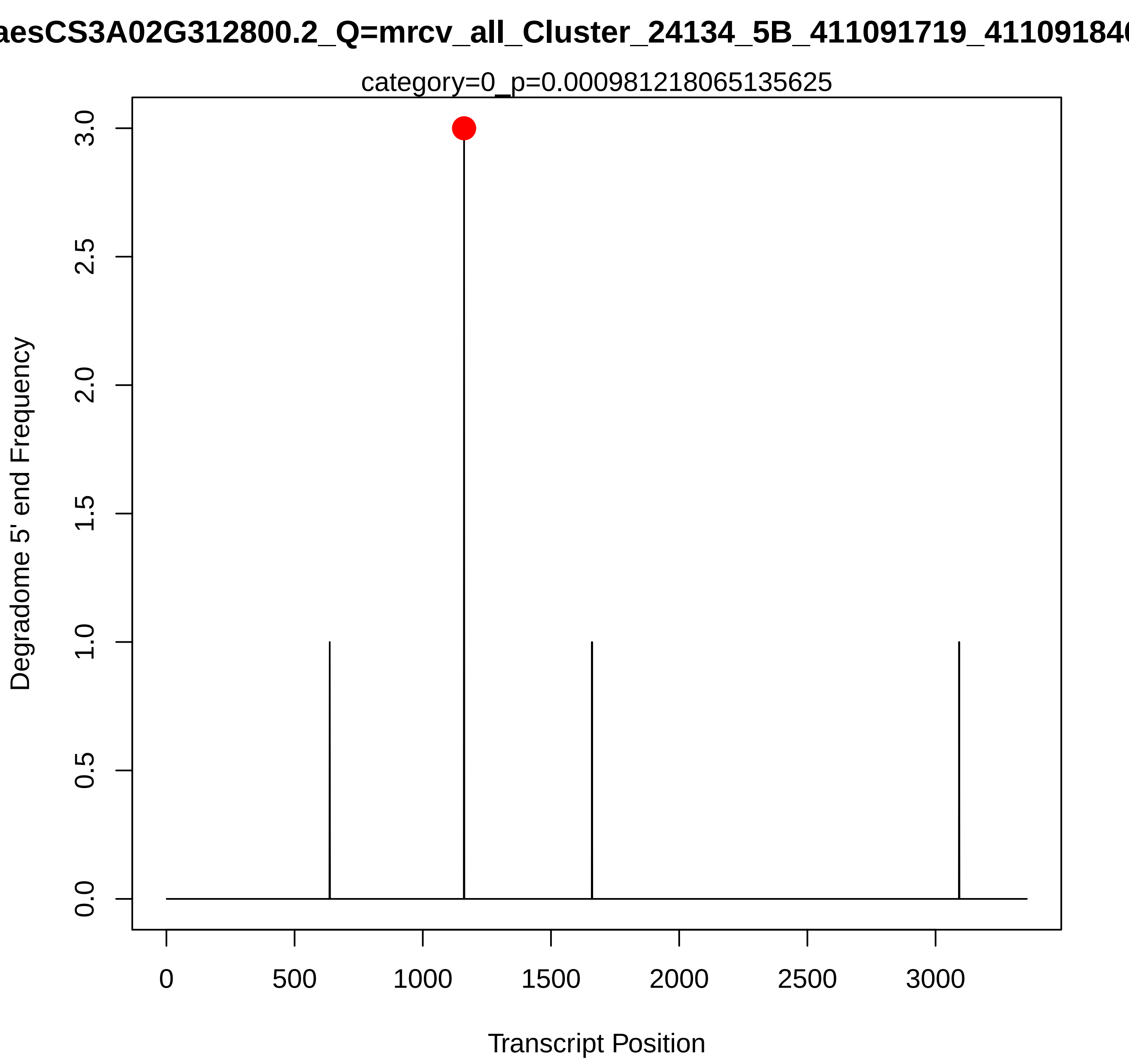

aesCS3A02G312800.2_Q=mrcv_all_Cluster_24134_5B_411091719_41109184
category=0_p=0.000981218065135625
Degradome 5' end Frequency
Transcript Position
0.0
0.5
1.0
1.5
2.0
2.5
3.0
0
500
1000
1500
2000
2500
3000

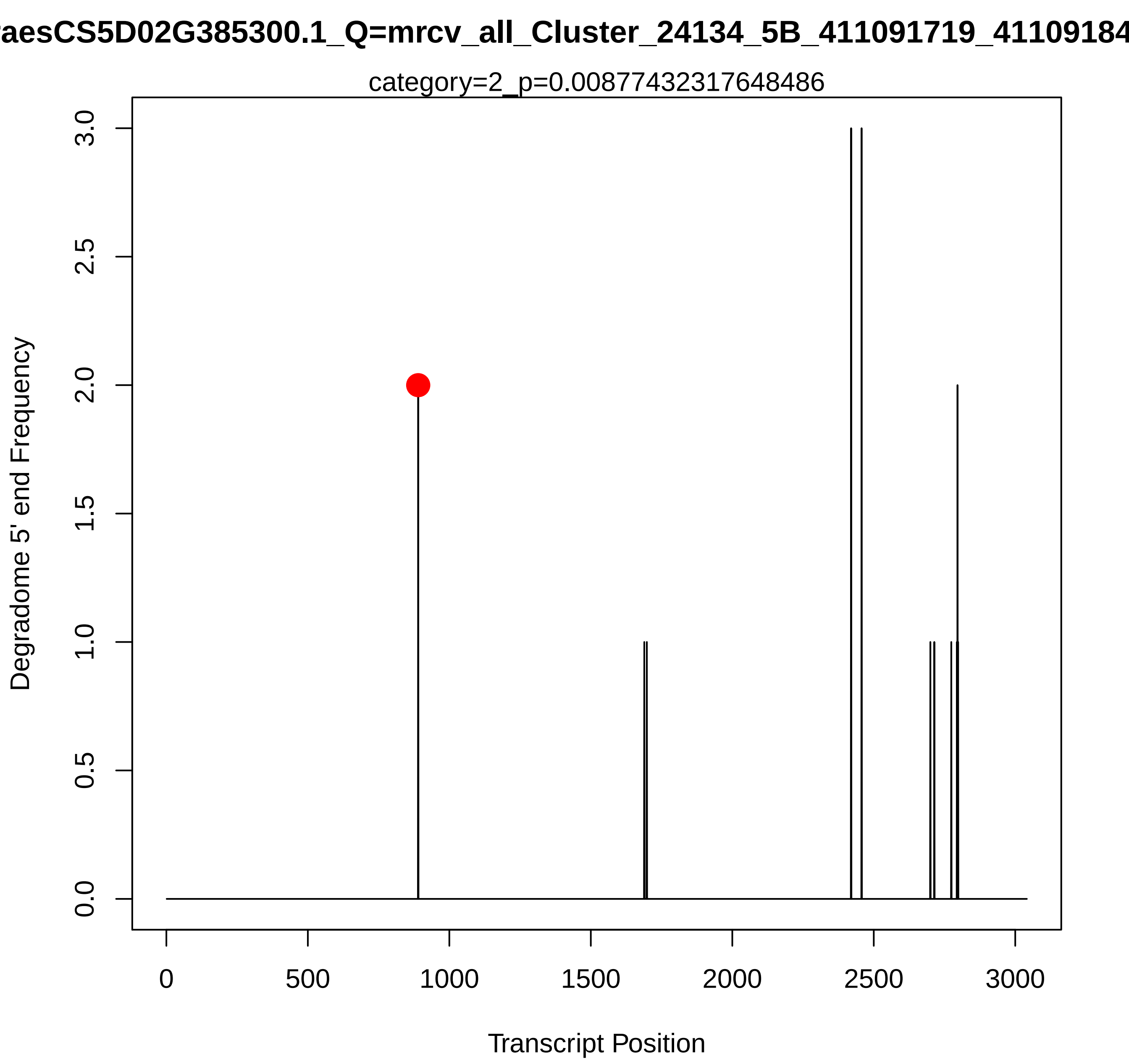
aesCS5D02G385300.1_Q=mrcv_all_Cluster_24134_5B_411091719_4110918
category=2_p=0.00877432317648486
Degradome 5' end Frequency
0.0
0.5
1.0
1.5
2.0
2.5
3.0
0
500
1000
1500
2000
2500
3000
Transcript Position

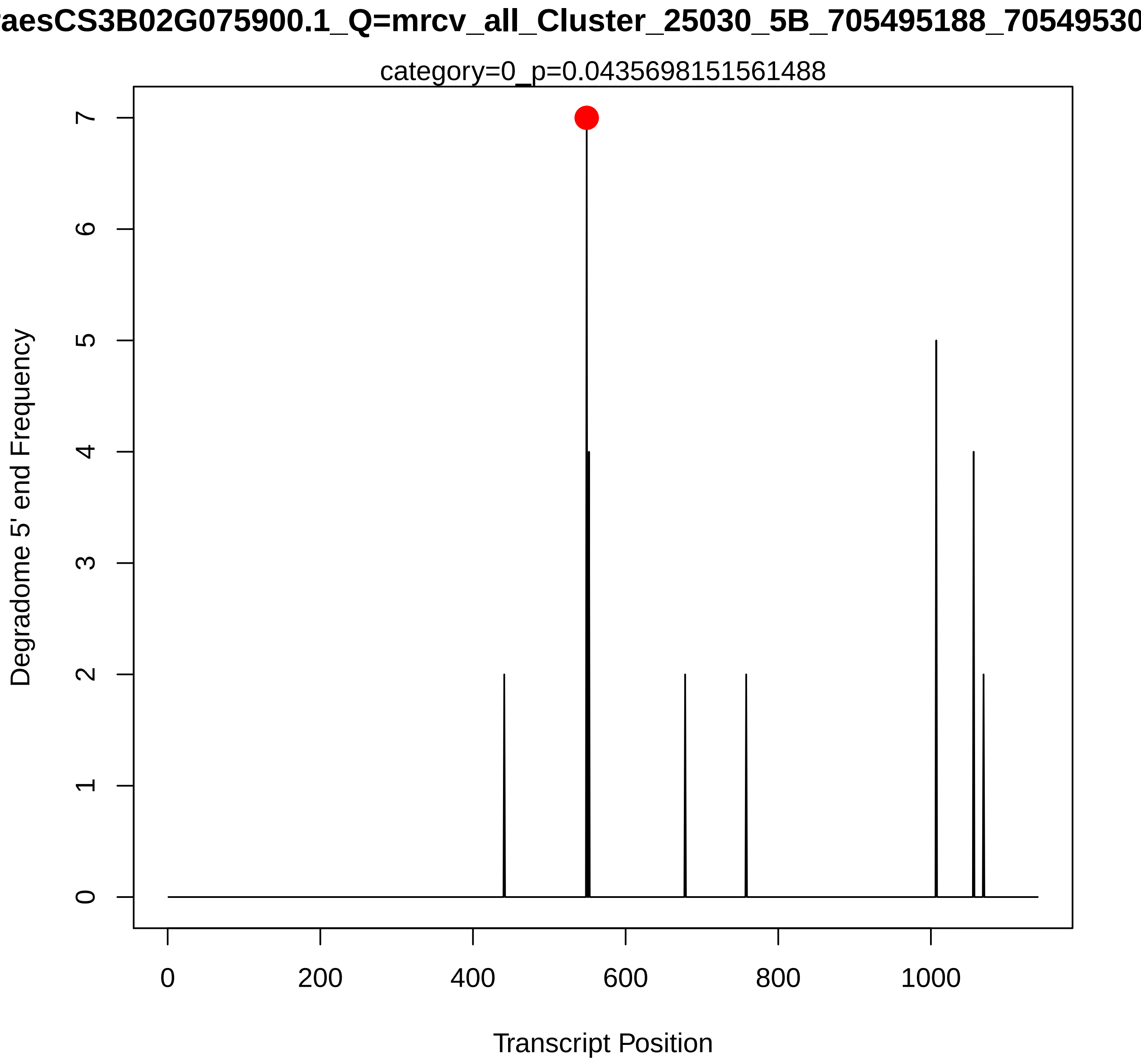

aesCS3B02G075900.1_Q=mrcv_all_Cluster_25030_5B_705495188_70549530
category=0_p=0.0435698151561488
Degradome 5' end Frequency
Transcript Position
0
1
2
3
4
5
6
7
0
200
400
600
800
1000

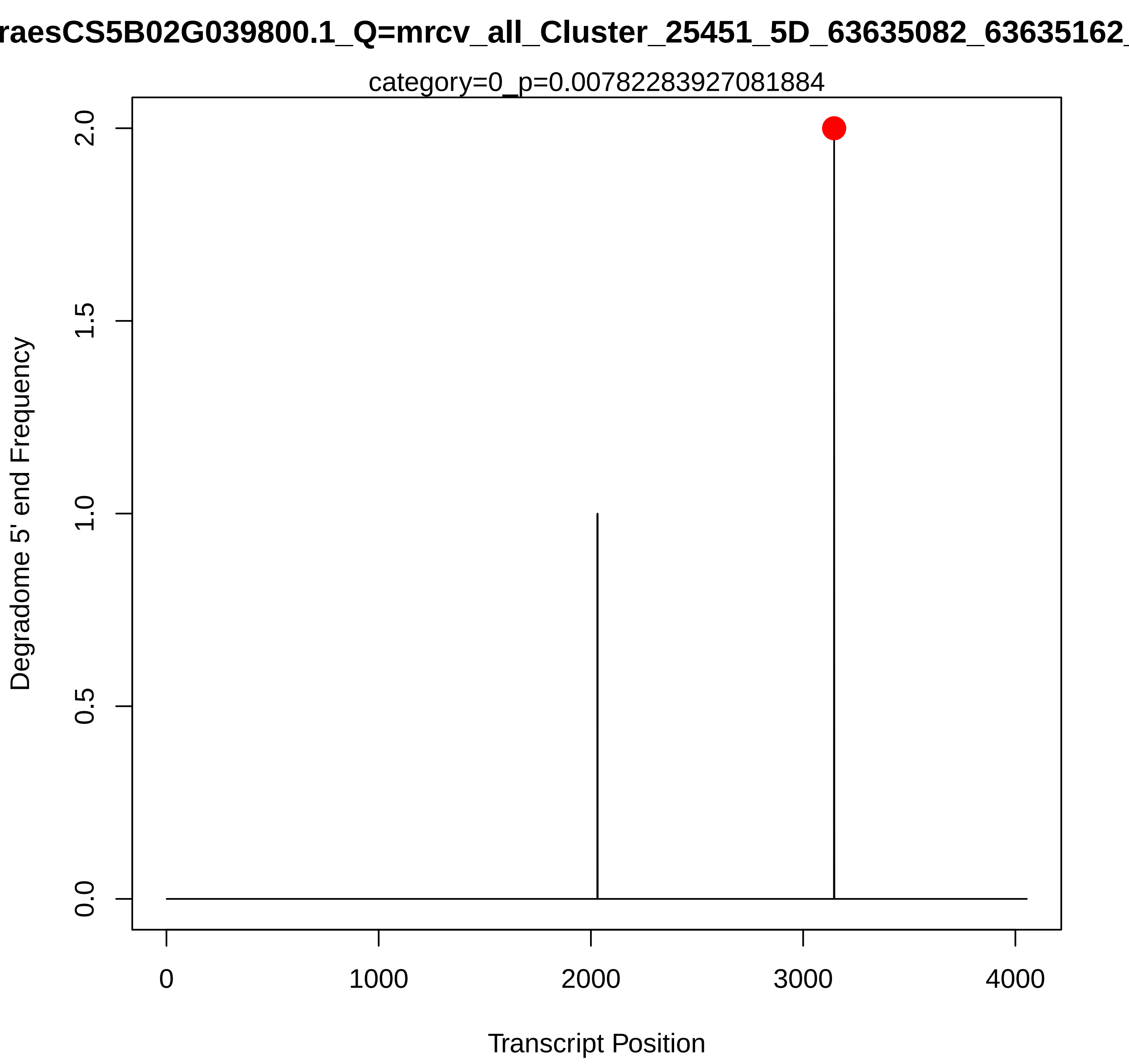

raesCS5B02G039800.1_Q=mrcv_all_Cluster_25451_5D_63635082_63635162_
category=0_p=0.00782283927081884
Degradome 5' end Frequency
0.0
0.5
1.0
1.5
2.0
0
1000
2000
3000
4000
Transcript Position

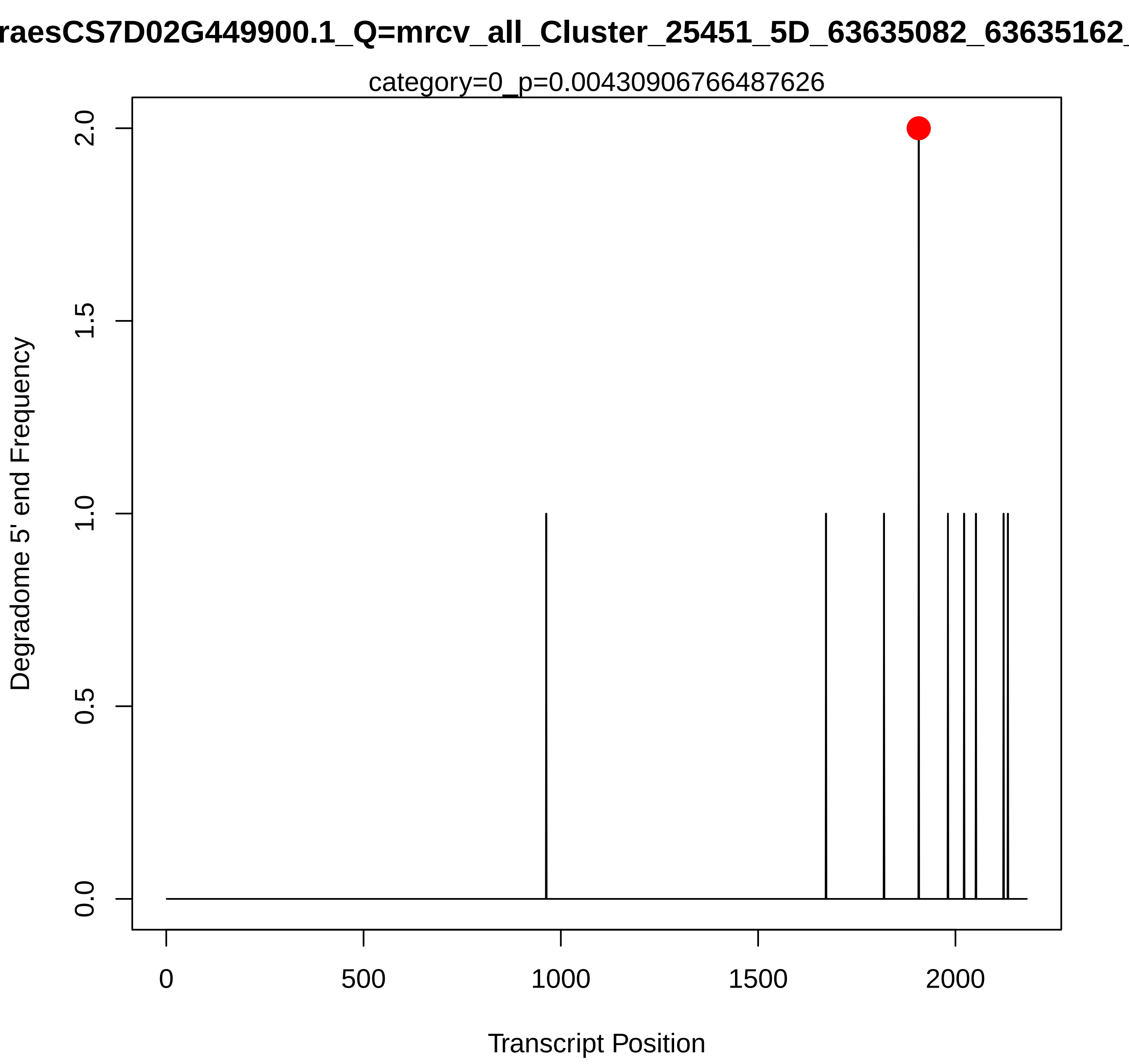
raesCS7D02G449900.1_Q=mrcv_all_Cluster_25451_5D_63635082_63635162_
category=0_p=0.00430906766487626
Degradome 5' end Frequency
0.0
0.5
1.0
1.5
2.0
0
500
1000
1500
2000
Transcript Position

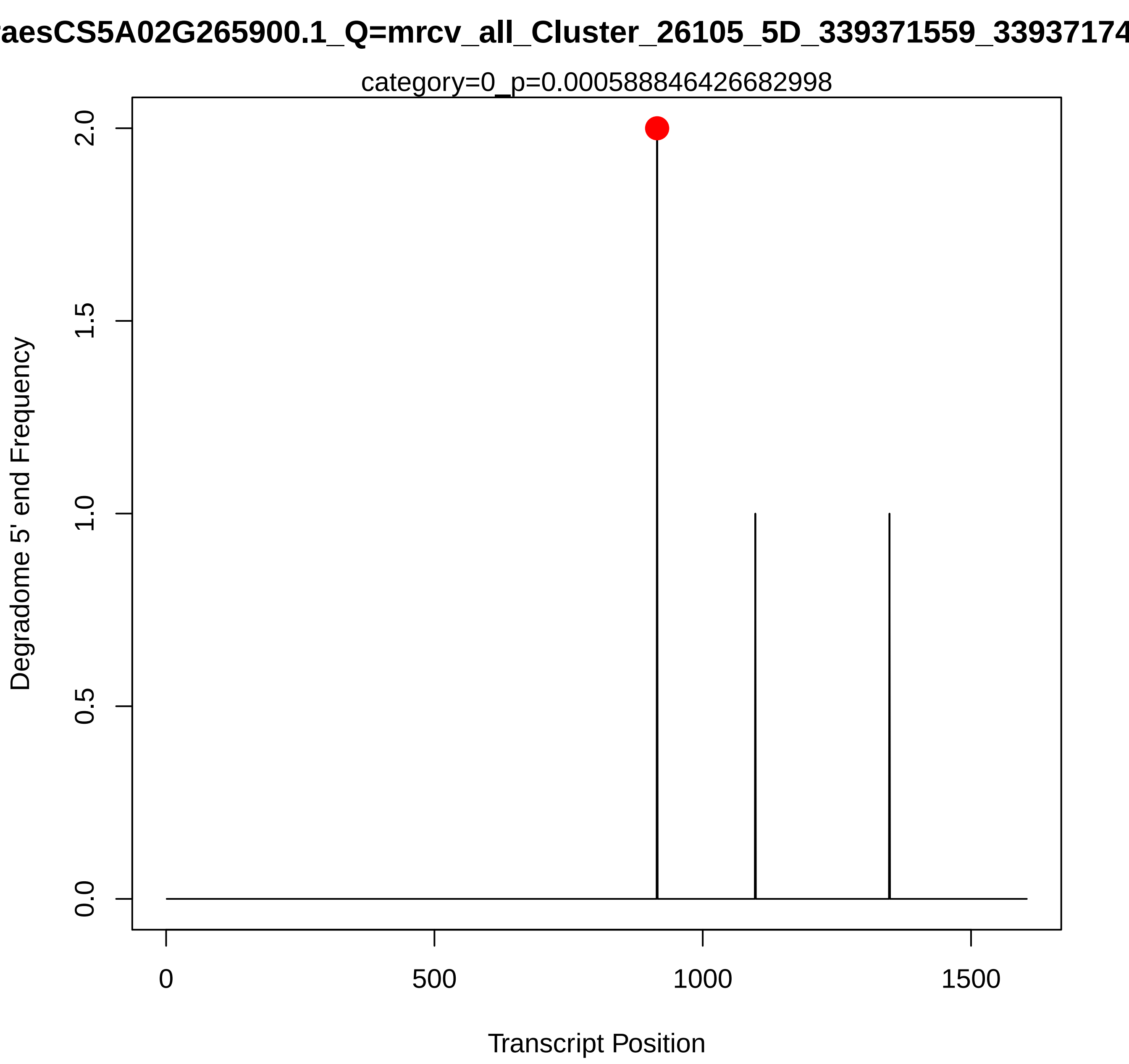
aesCS5A02G265900.1_Q=mrcv_all_Cluster_26105_5D_339371559_33937174
category=0_p=0.000588846426682998
Degradome 5' end Frequency
0.0
0.5
1.0
1.5
2.0
0
500
1000
1500
Transcript Position

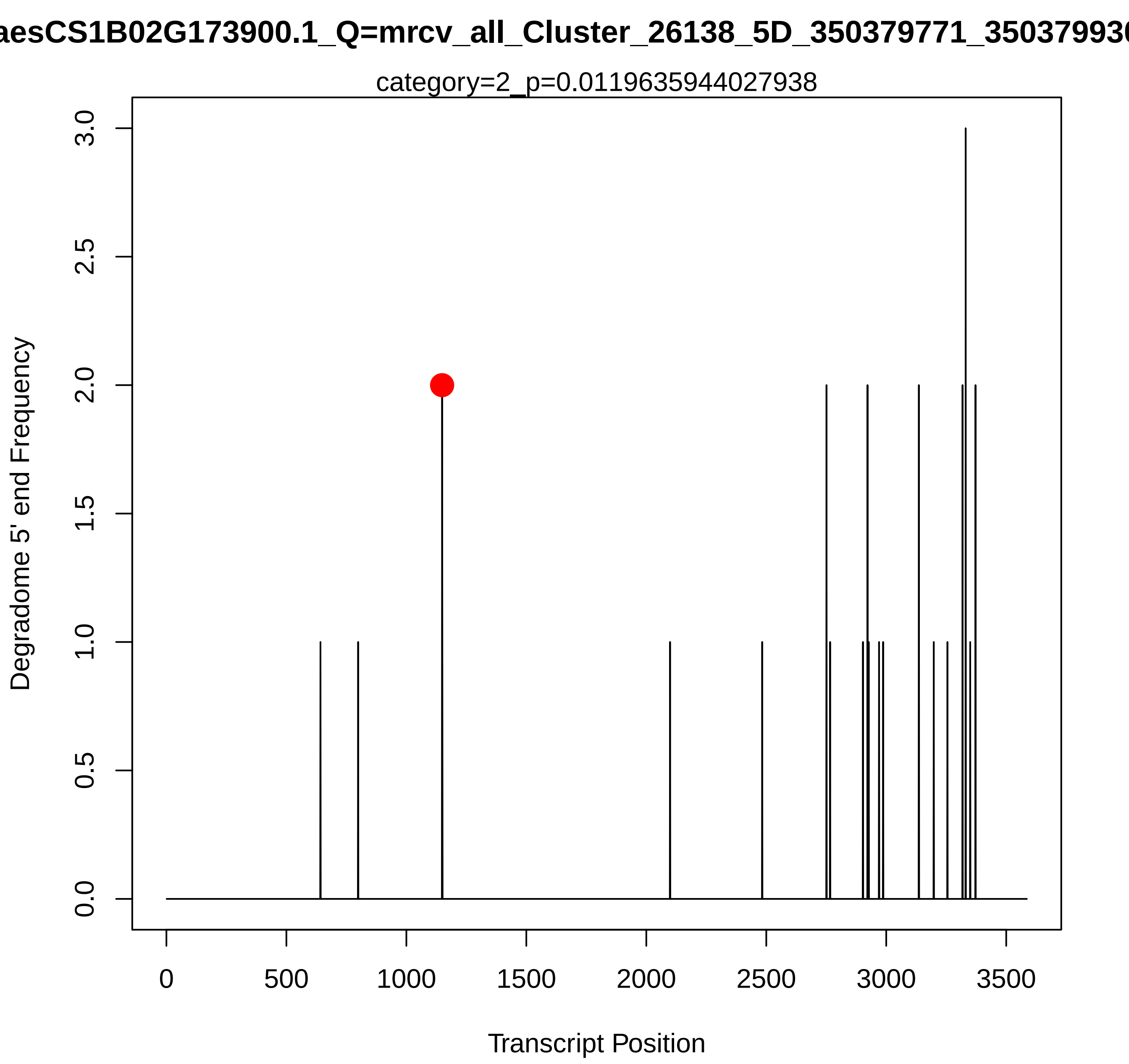
aesCS1B02G173900.1_Q=mrcv_all_Cluster_26138_5D_350379771_35037993
category=2_p=0.0119635944027938
Degradome 5' end Frequency
0.0
0.5
1.0
1.5
2.0
2.5
3.0
0
500
1000
1500
2000
2500
3000
3500
Transcript Position

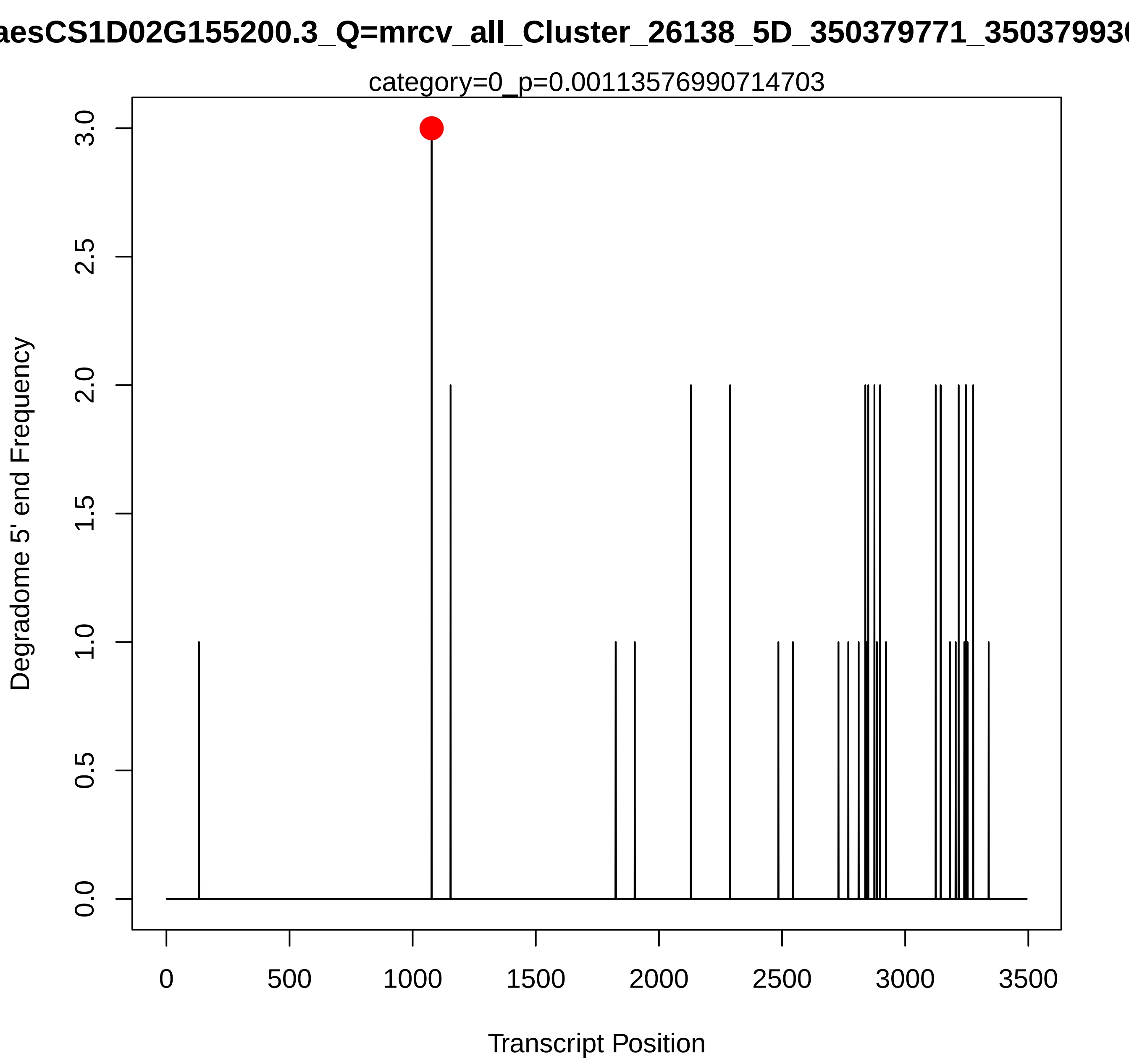
aesCS1D02G155200.3_Q=mrcv_all_Cluster_26138_5D_350379771_35037993
category=0_p=0.00113576990714703
Degradome 5' end Frequency
0.0
0.5
1.0
1.5
2.0
2.5
3.0
0
500
1000
1500
2000
2500
3000
3500
Transcript Position

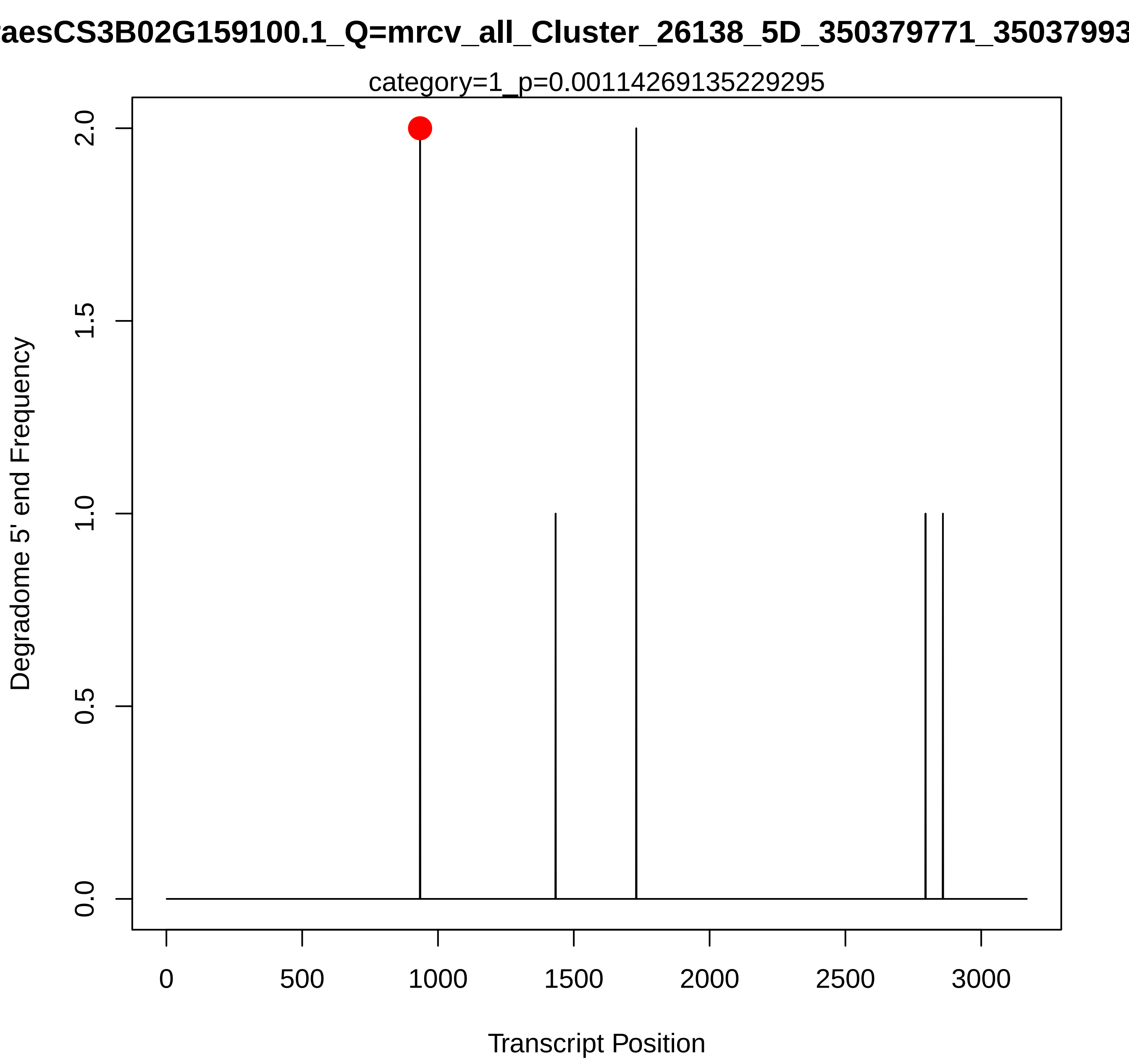
aesCS3B02G159100.1_Q=mrcv_all_Cluster_26138_5D_350379771_35037993
category=1_p=0.00114269135229295
Degradome 5' end Frequency
0.0
0.5
1.0
1.5
2.0
0
500
1000
1500
2000
2500
3000
Transcript Position

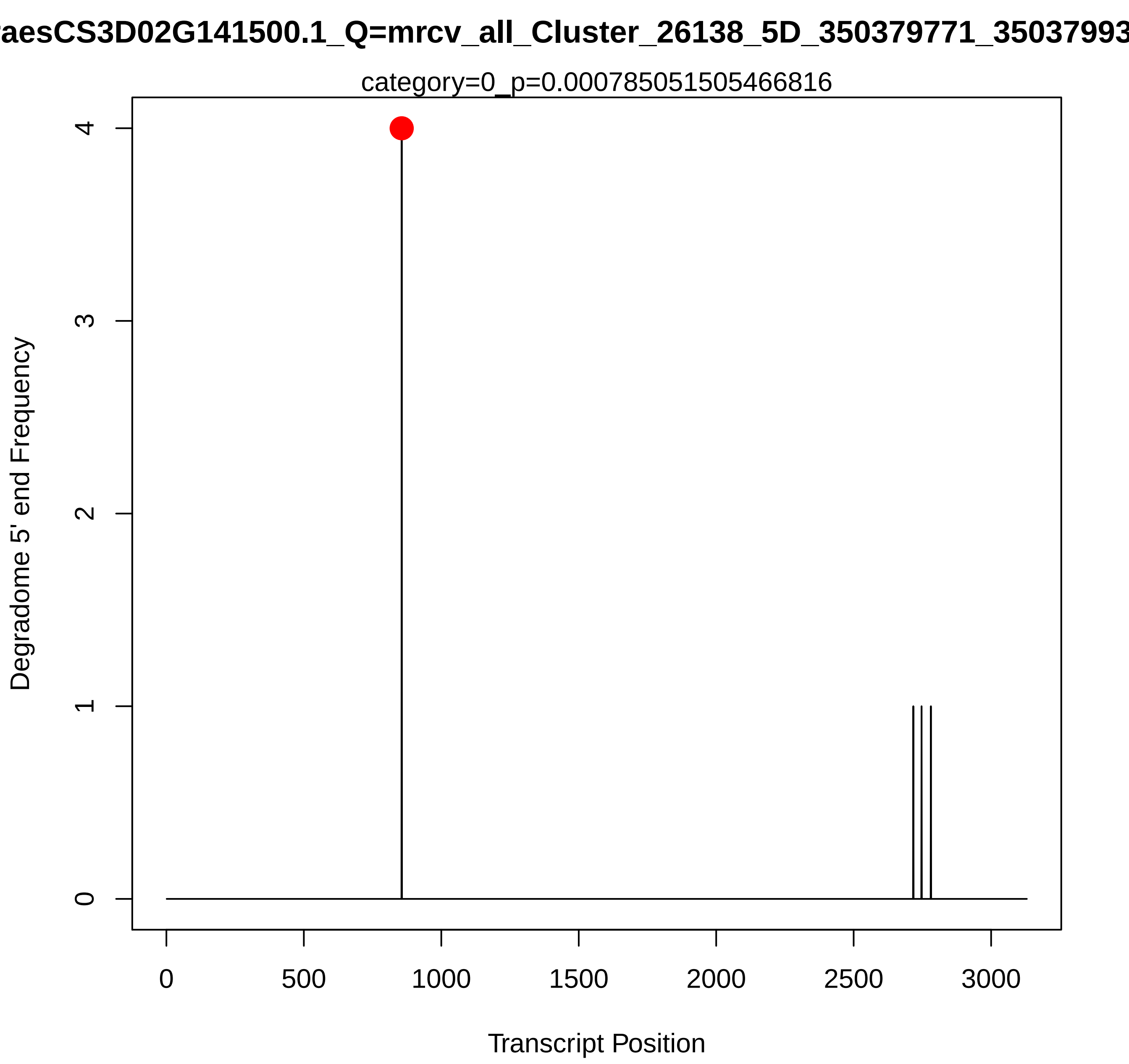
aesCS3D02G141500.1_Q=mrcv_all_Cluster_26138_5D_350379771_3503799
category=0_p=0.000785051505466816
Degradome 5' end Frequency
0
1
2
3
4
0
500
1000
1500
2000
2500
3000
Transcript Position

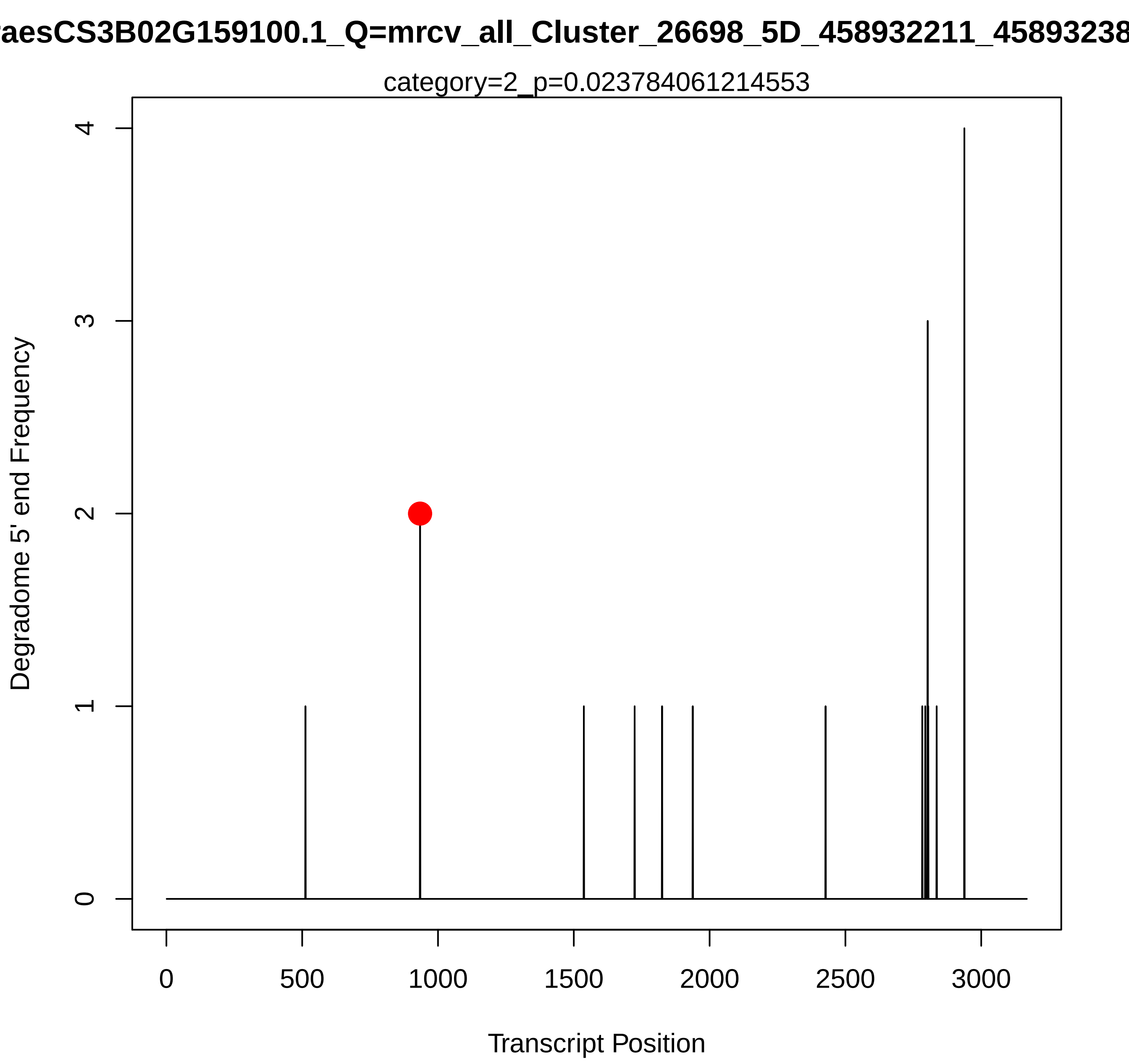

aesCS3B02G159100.1_Q=mrcv_all_Cluster_26698_5D_458932211_4589323
category=2_p=0.023784061214553
Degradome 5' end Frequency
0
1
2
3
4
0
500
1000
1500
2000
2500
3000
Transcript Position

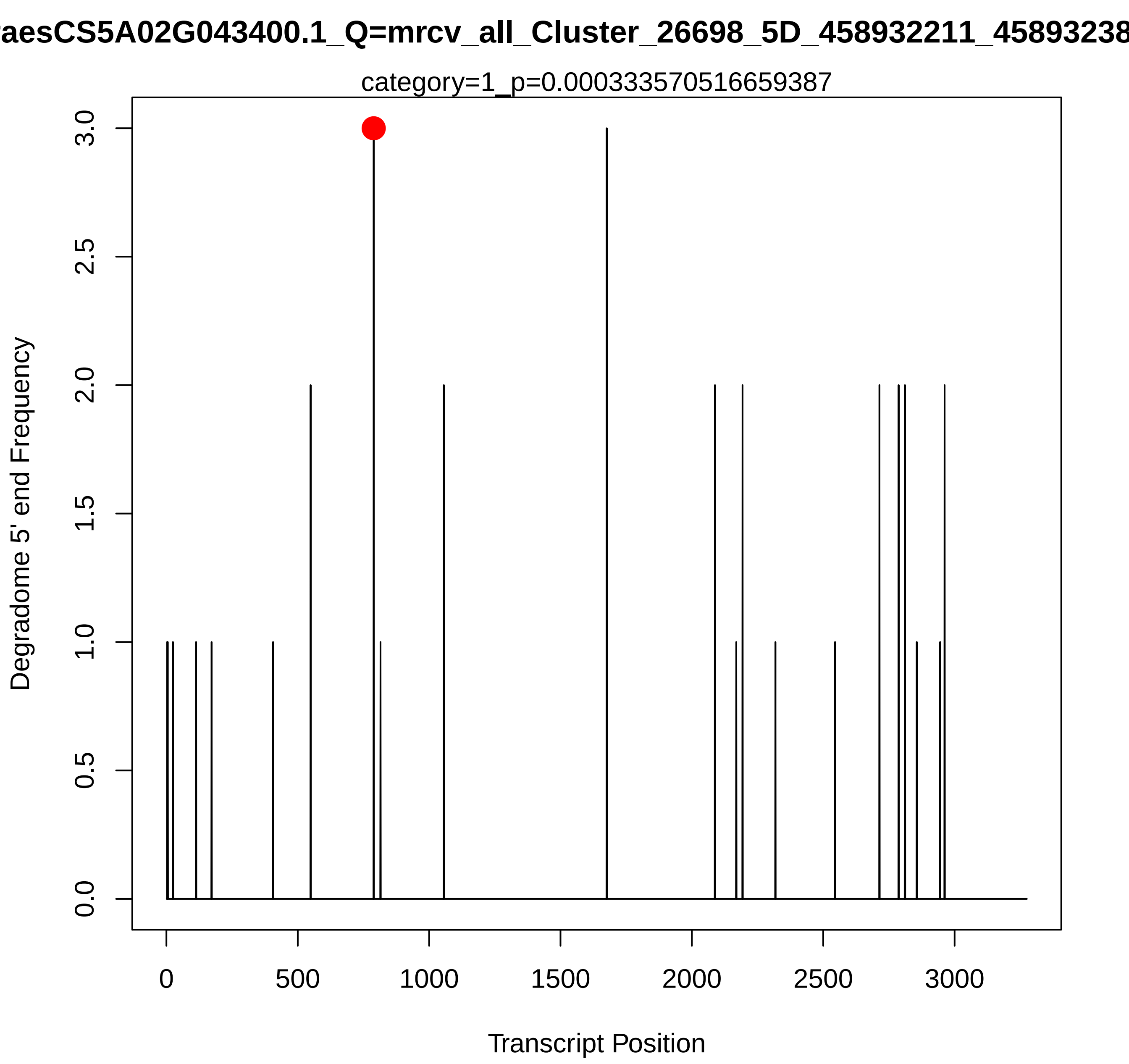

aesCS5A02G043400.1_Q=mrcv_all_Cluster_26698_5D_458932211_45893238
category=1_p=0.000333570516659387
Degradome 5' end Frequency
0.0
0.5
1.0
1.5
2.0
2.5
3.0
0
500
1000
1500
2000
2500
3000
Transcript Position

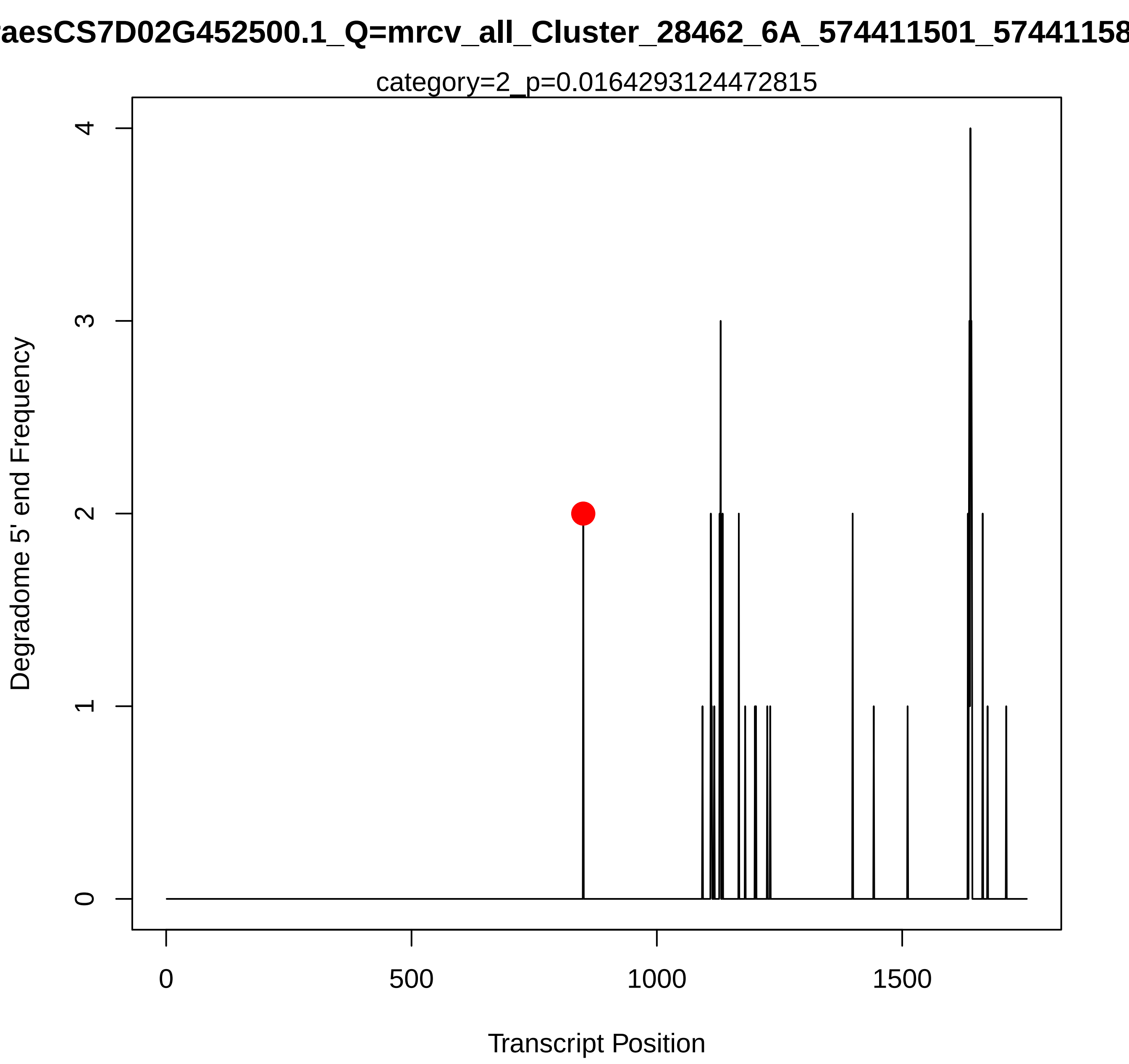

aesCS7D02G452500.1_Q=mrcv_all_Cluster_28462_6A_574411501_57441158
category=2_p=0.0164293124472815
Degradome 5' end Frequency
Transcript Position
0
1
2
3
4
0
500
1000
1500

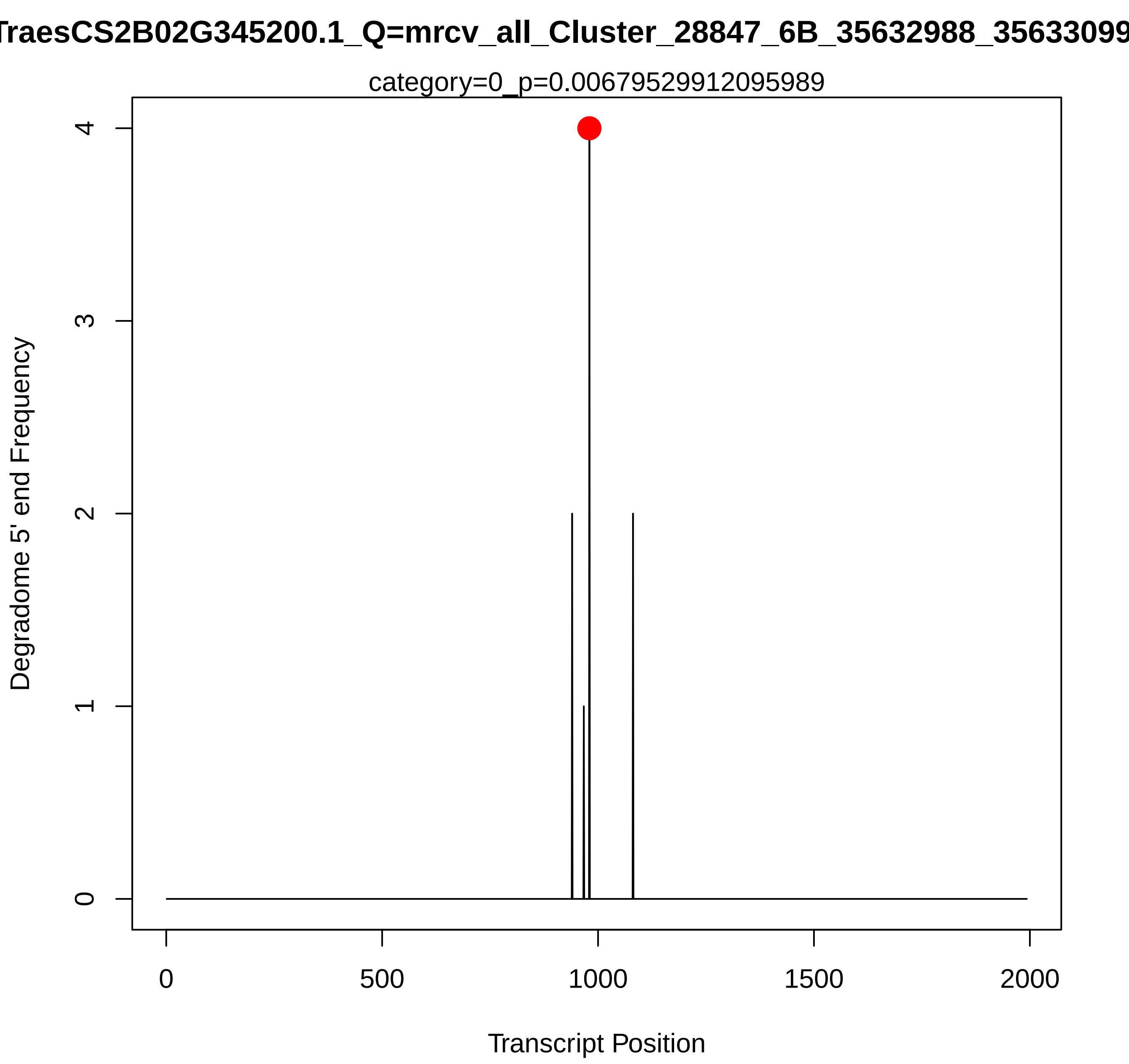

TraesCS2B02G345200.1_Q=mrcv_all_Cluster_28847_6B_35632988_35633099
category=0_p=0.00679529912095989
Degradome 5' end Frequency
0
1
2
3
4
0
500
1000
1500
2000
Transcript Position

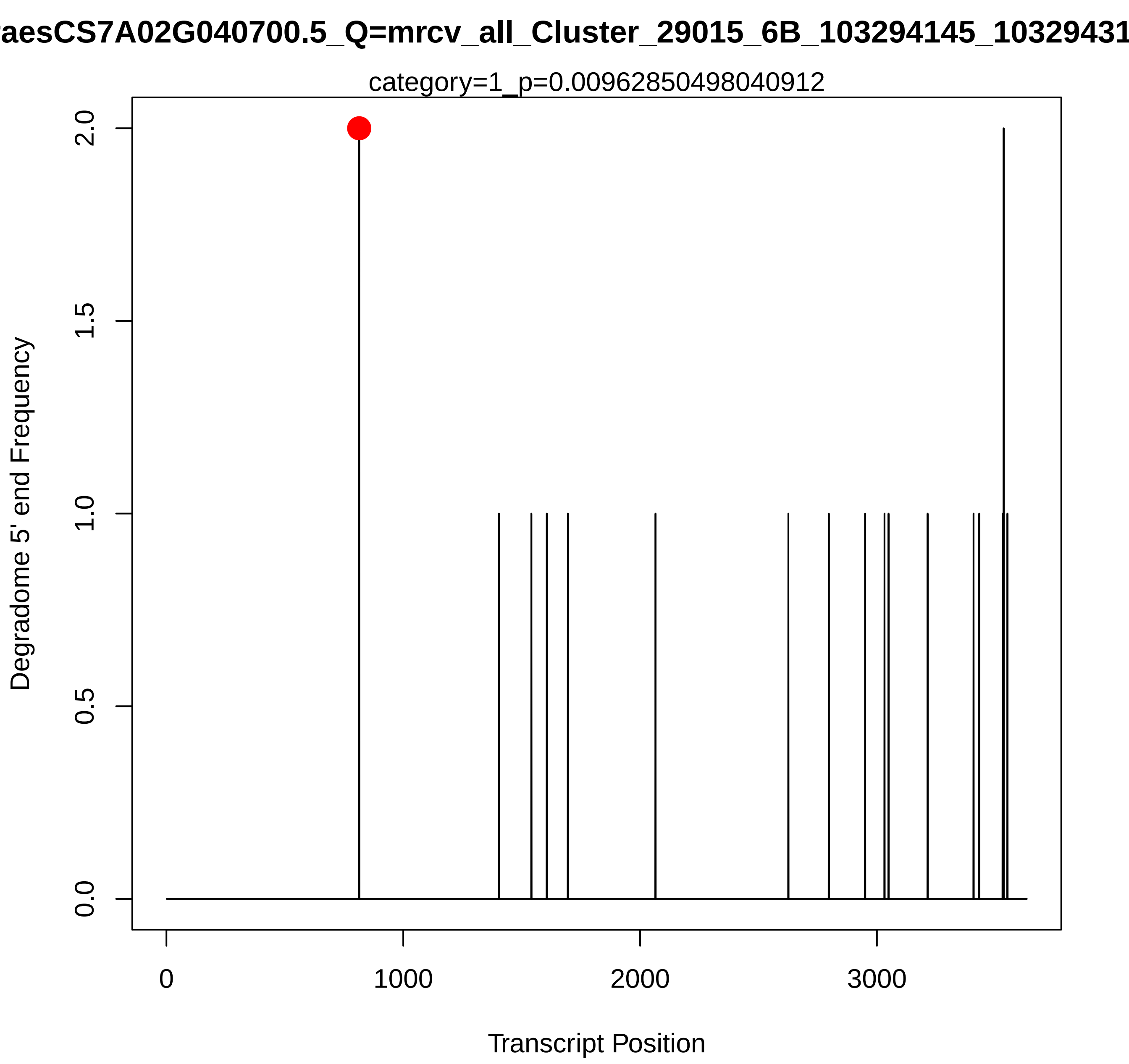
aesCS7A02G040700.5_Q=mrcv_all_Cluster_29015_6B_103294145_10329431
category=1_p=0.00962850498040912
Degradome 5' end Frequency
0.0
0.5
1.0
1.5
2.0
0
1000
2000
3000
Transcript Position

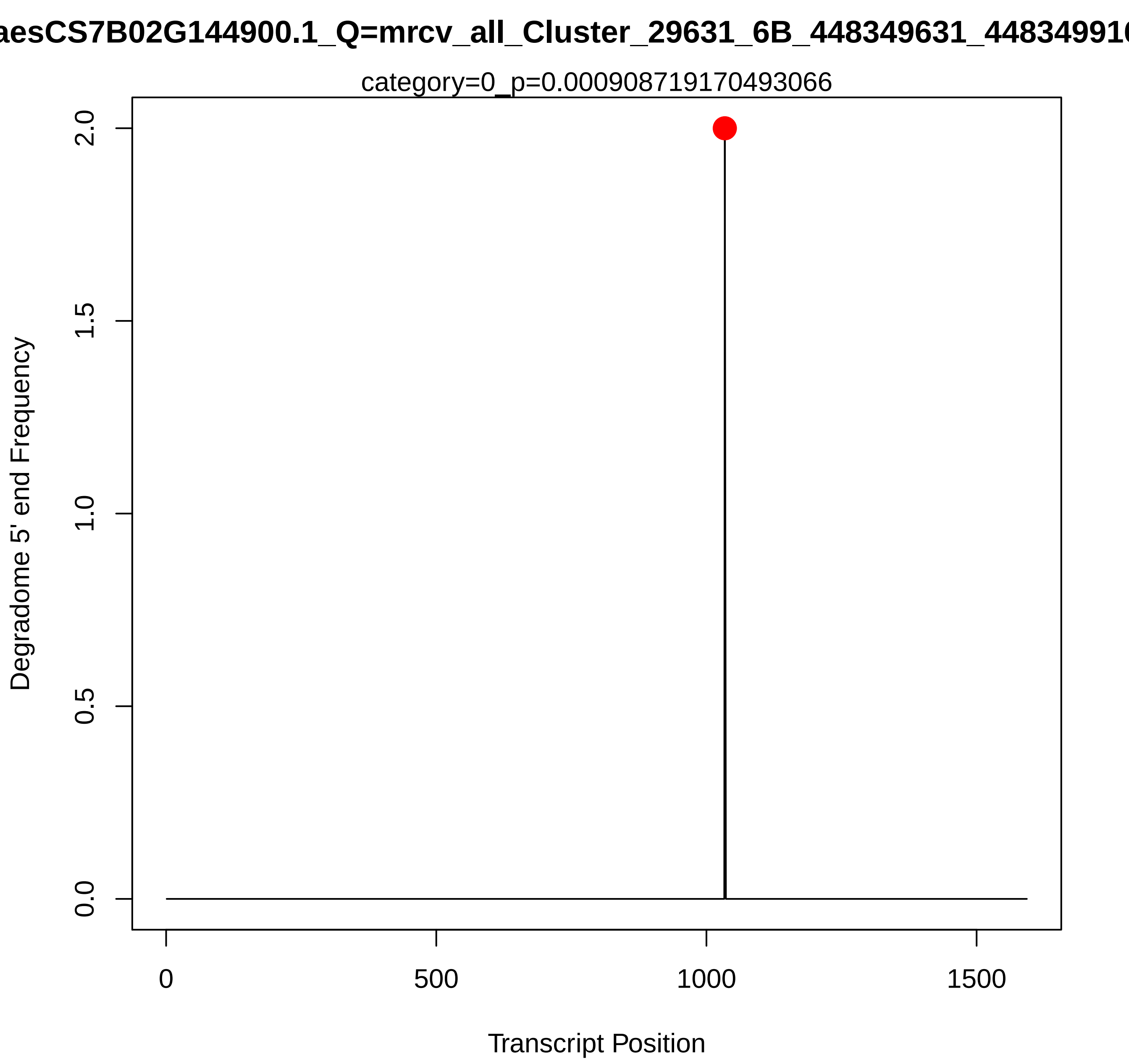

aesCS7B02G144900.1_Q=mrcv_all_Cluster_29631_6B_448349631_44834991
category=0_p=0.000908719170493066
Degradome 5' end Frequency
0.0
0.5
1.0
1.5
2.0
0
500
1000
1500
Transcript Position

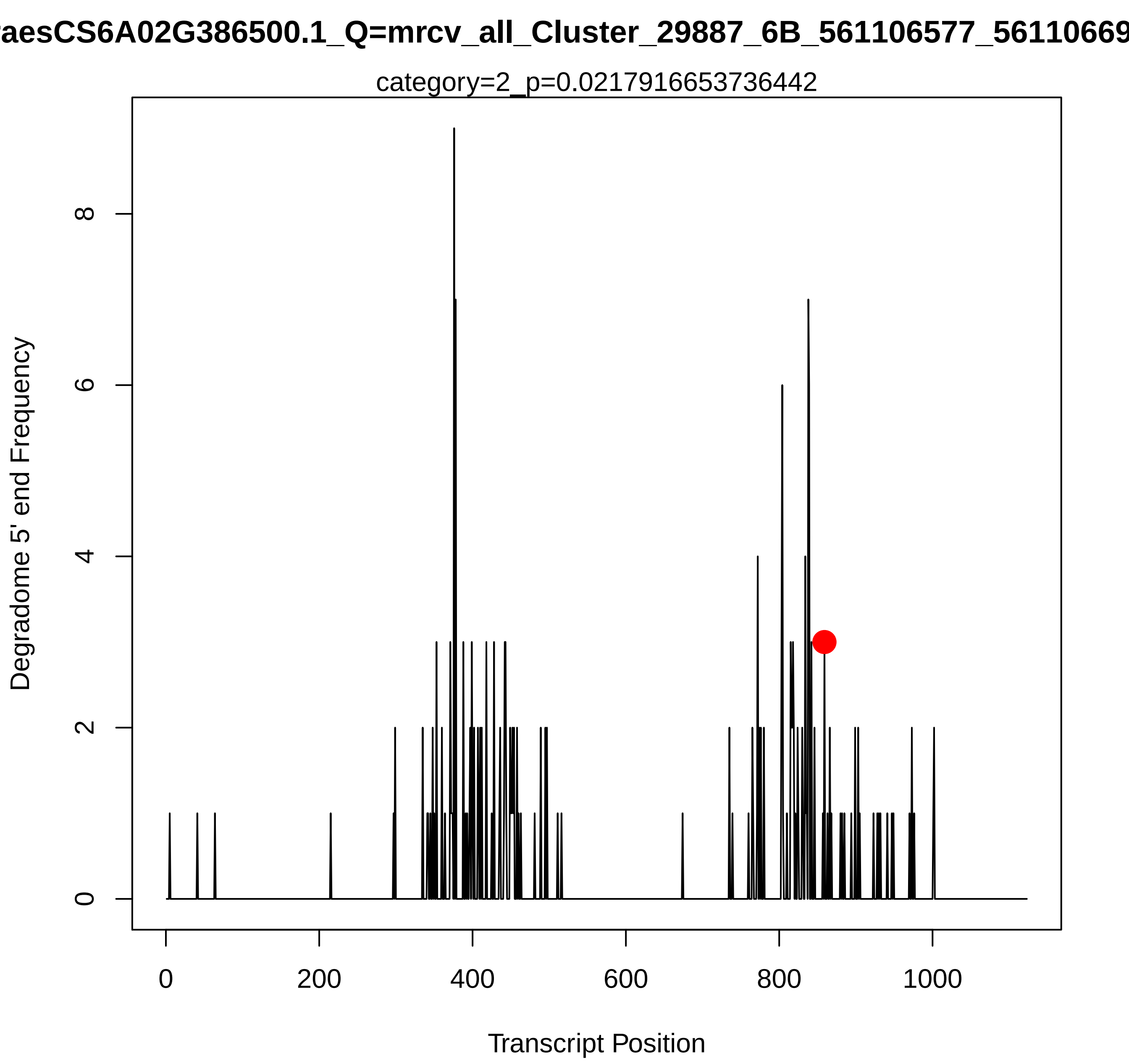

aesCS6A02G386500.1_Q=mrcv_all_Cluster_29887_6B_561106577_56110669
category=2_p=0.0217916653736442
Degradome 5' end Frequency
0
2
4
6
8
0
200
400
600
800
1000
Transcript Position

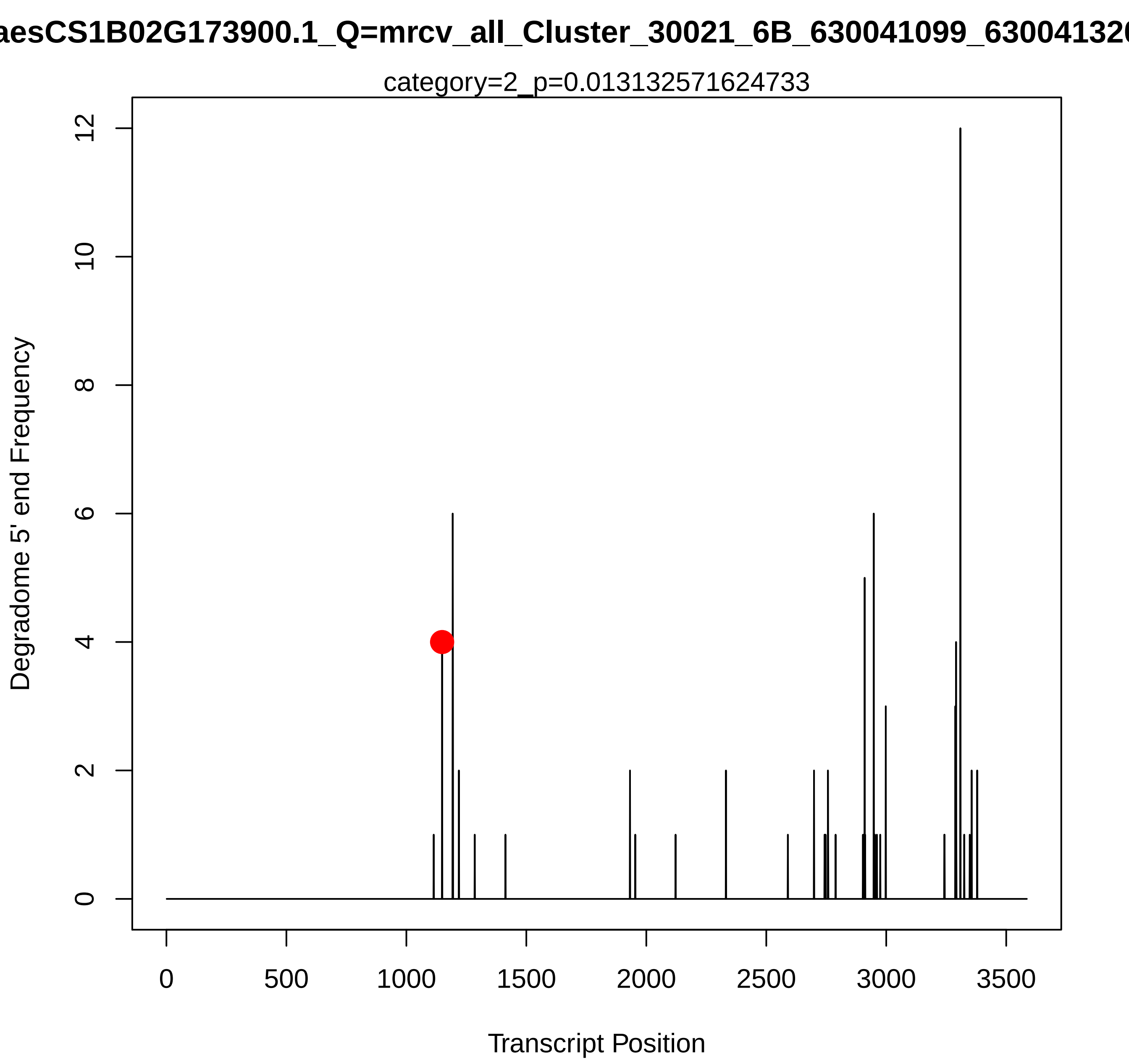
aesCS1B02G173900.1_Q=mrcv_all_Cluster_30021_6B_630041099_63004132
category=2_p=0.013132571624733
Degradome 5' end Frequency
Transcript Position
0
2
4
6
8
10
12
0
500
1000
1500
2000
2500
3000
3500

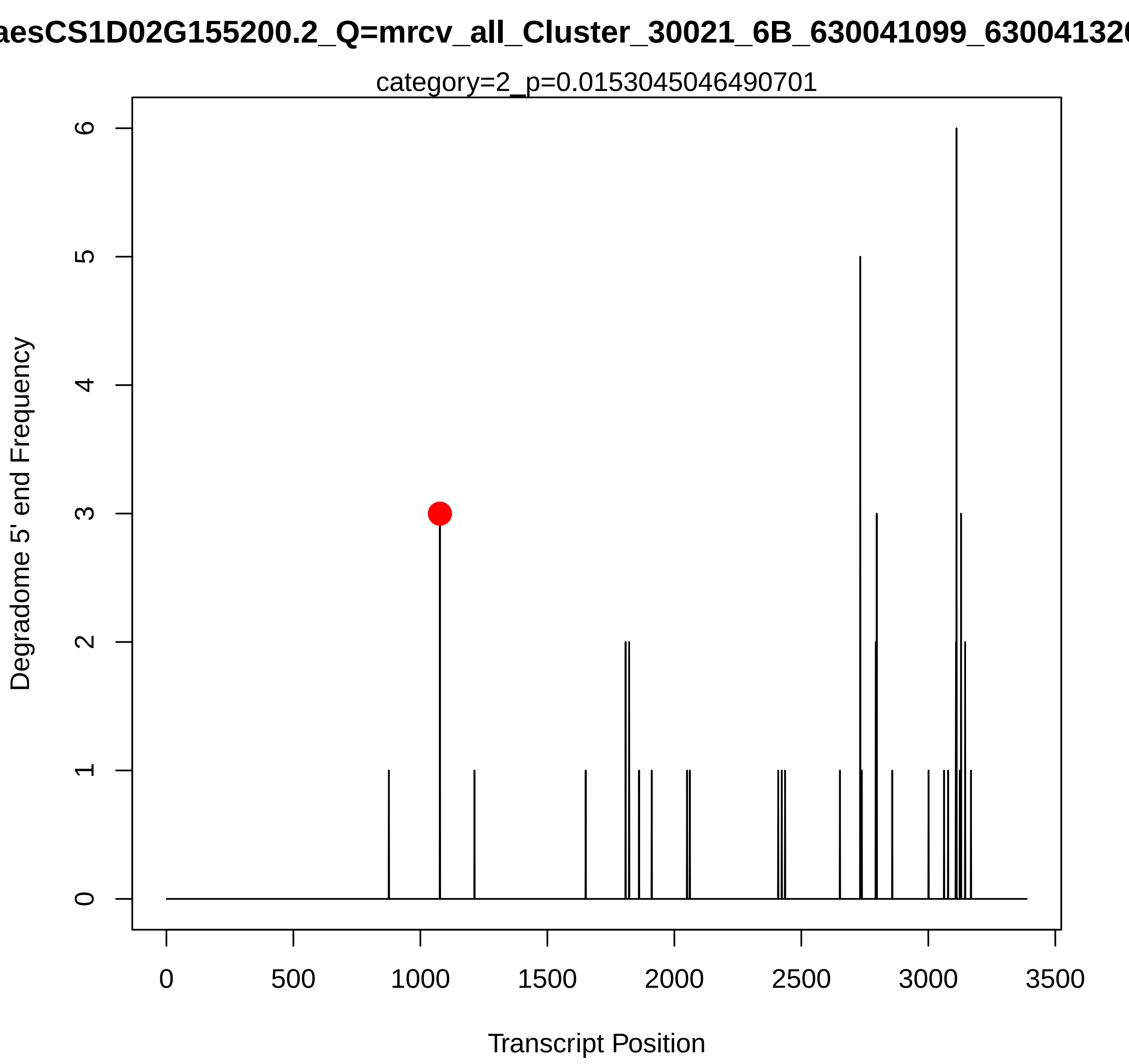

aesCS1D02G155200.2_Q=mrcv_all_Cluster_30021_6B_630041099_63004132
category=2_p=0.0153045046490701
Degradome 5' end Frequency
0
1
2
3
4
5
6
0
500
1000
1500
2000
2500
3000
3500
Transcript Position

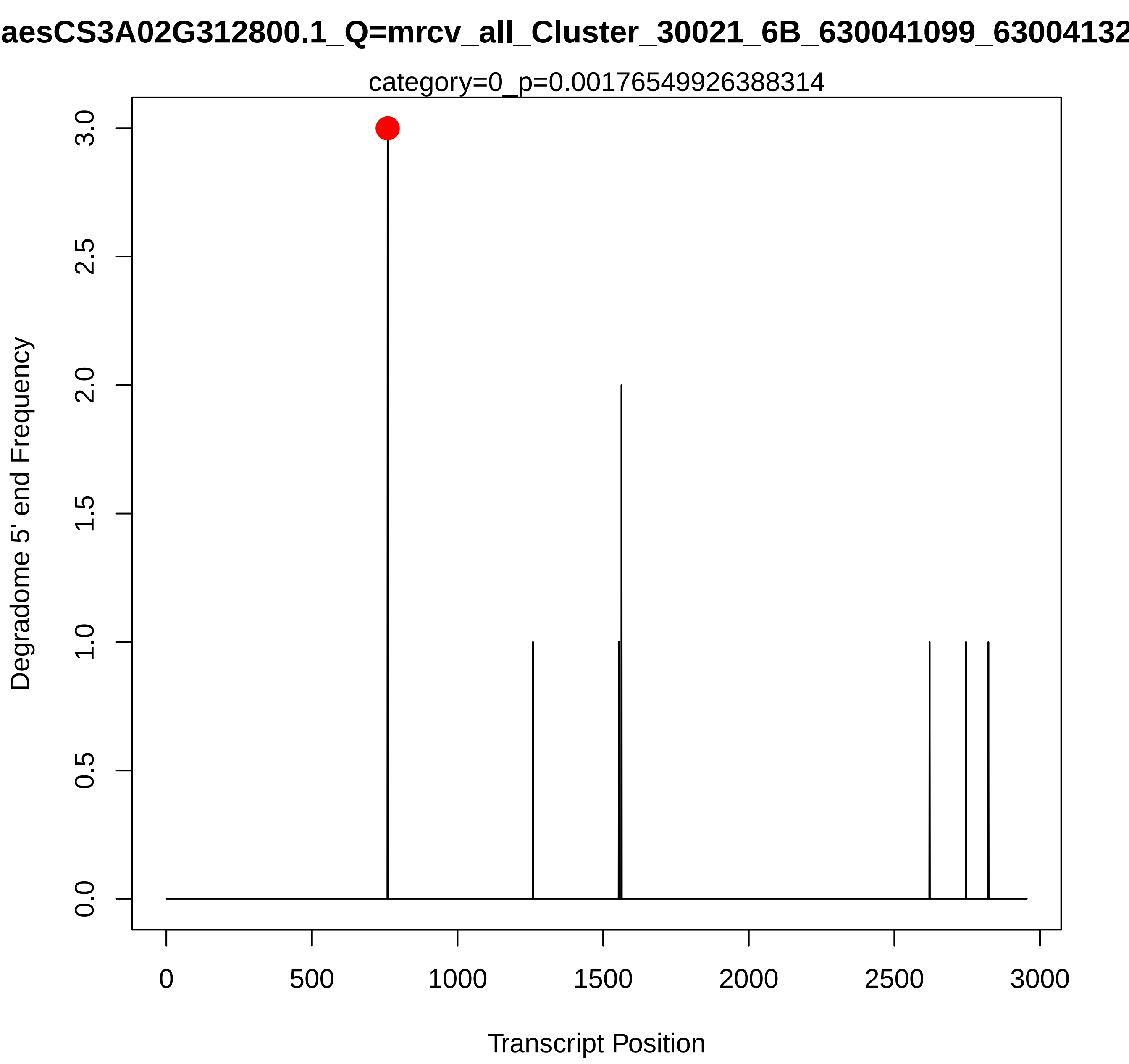

aesCS3A02G312800.1_Q=mrcv_all_Cluster_30021_6B_630041099_6300413
category=0_p=0.00176549926388314
Degradome 5' end Frequency
0.0
0.5
1.0
1.5
2.0
2.5
3.0
0
500
1000
1500
2000
2500
3000
Transcript Position

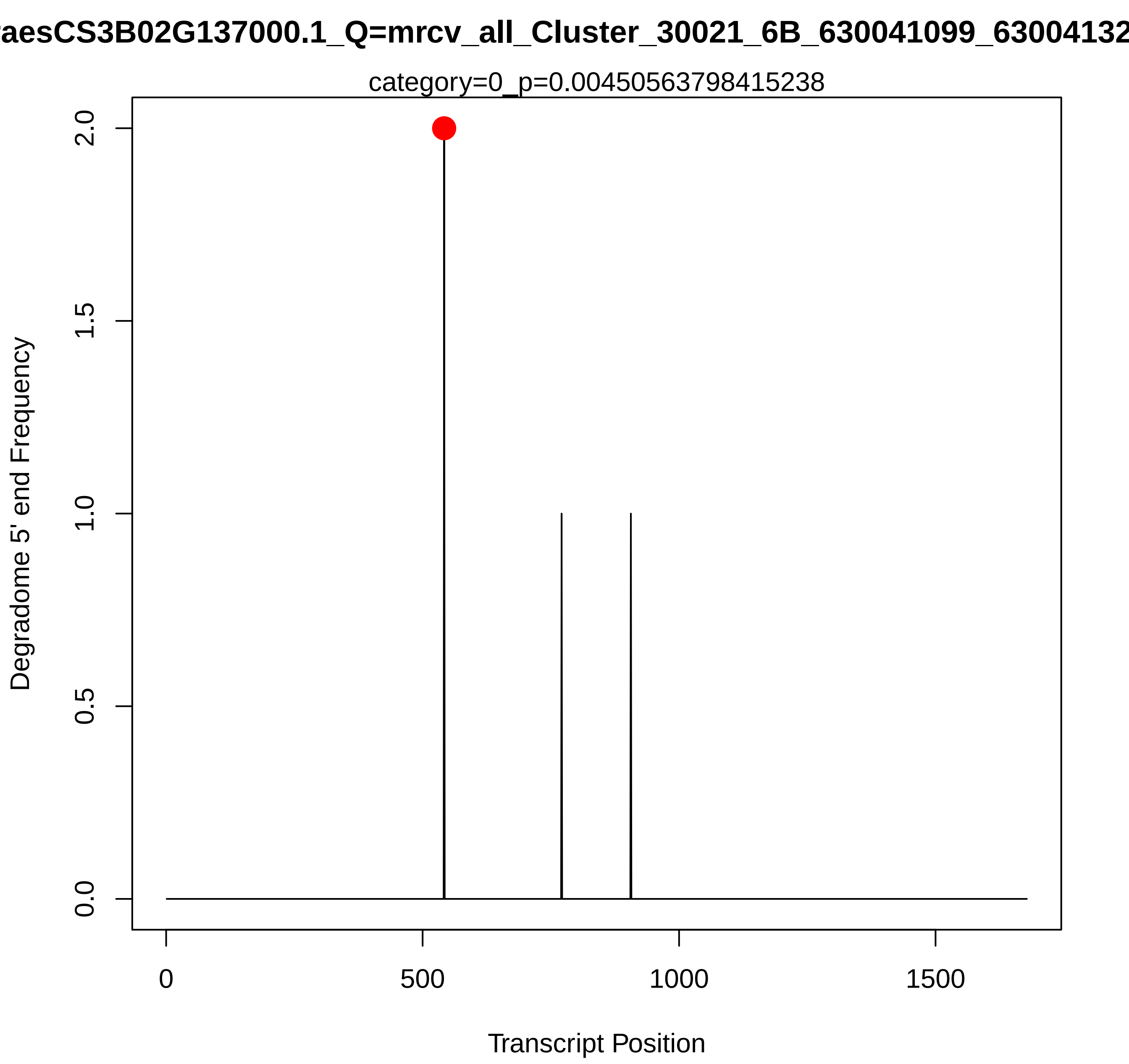
aesCS3B02G137000.1_Q=mrcv_all_Cluster_30021_6B_630041099_6300413
category=0_p=0.00450563798415238
Degradome 5' end Frequency
0.0
0.5
1.0
1.5
2.0
0
500
1000
1500
Transcript Position

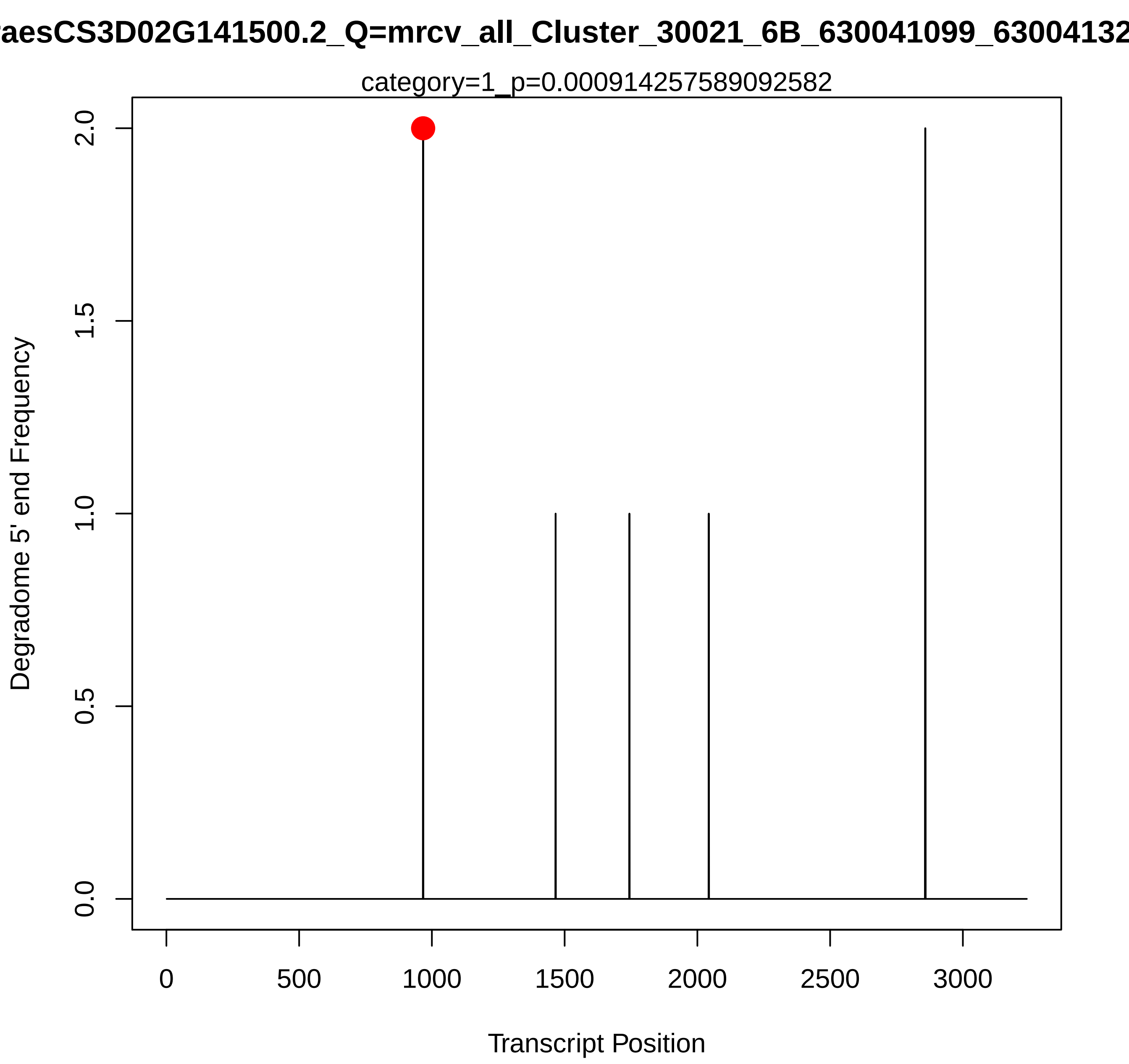

TraesCS3D02G141500.2_Q=mrcv_all_Cluster_30021_6B_630041099_63004132
category=1_p=0.000914257589092582
Degradome 5' end Frequency
0.0
0.5
1.0
1.5
2.0
0
500
1000
1500
2000
2500
3000
Transcript Position

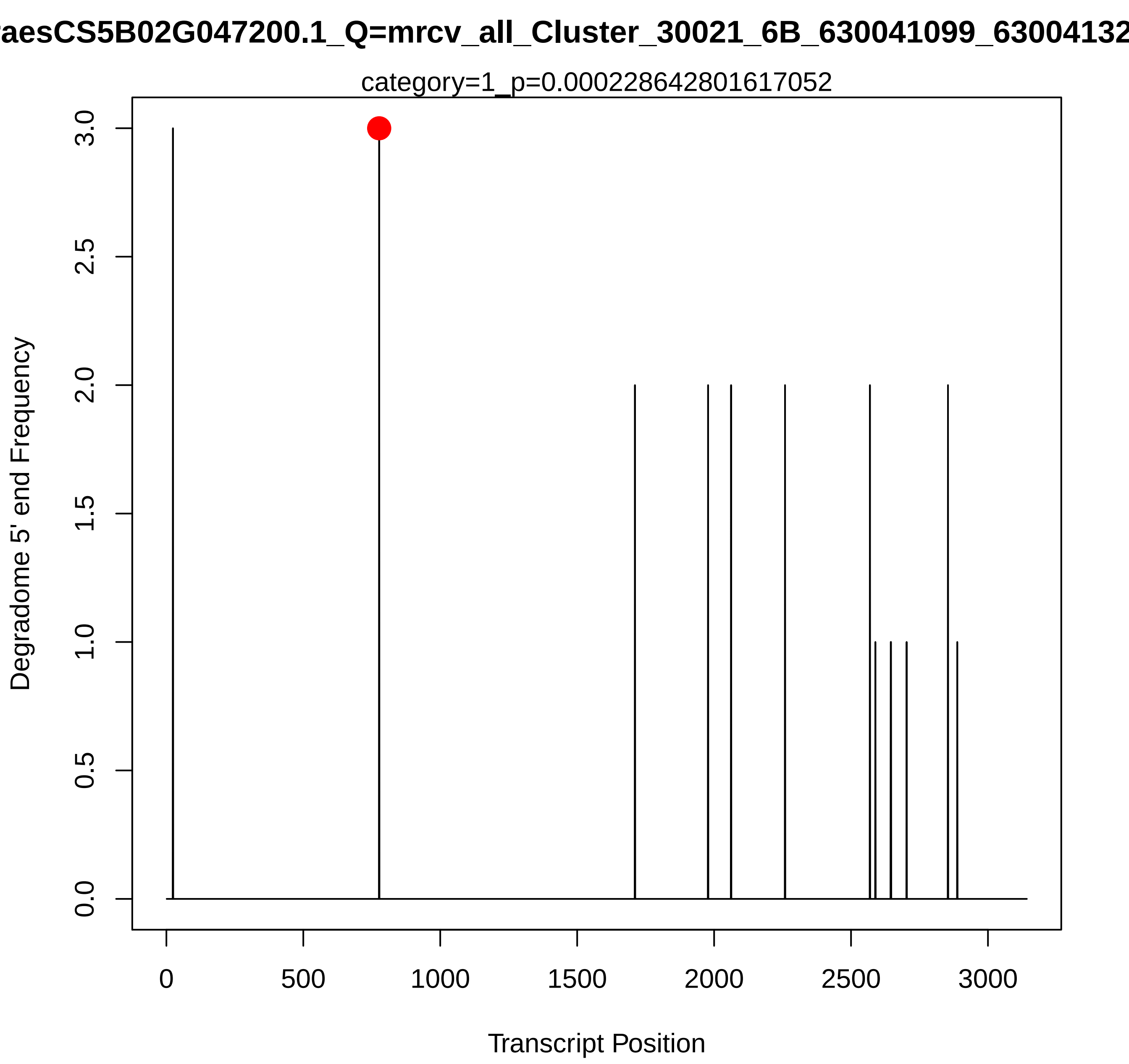

aesCS5B02G047200.1_Q=mrcv_all_Cluster_30021_6B_630041099_63004132
category=1_p=0.000228642801617052
Degradome 5' end Frequency
0.0
0.5
1.0
1.5
2.0
2.5
3.0
0
500
1000
1500
2000
2500
3000
Transcript Position

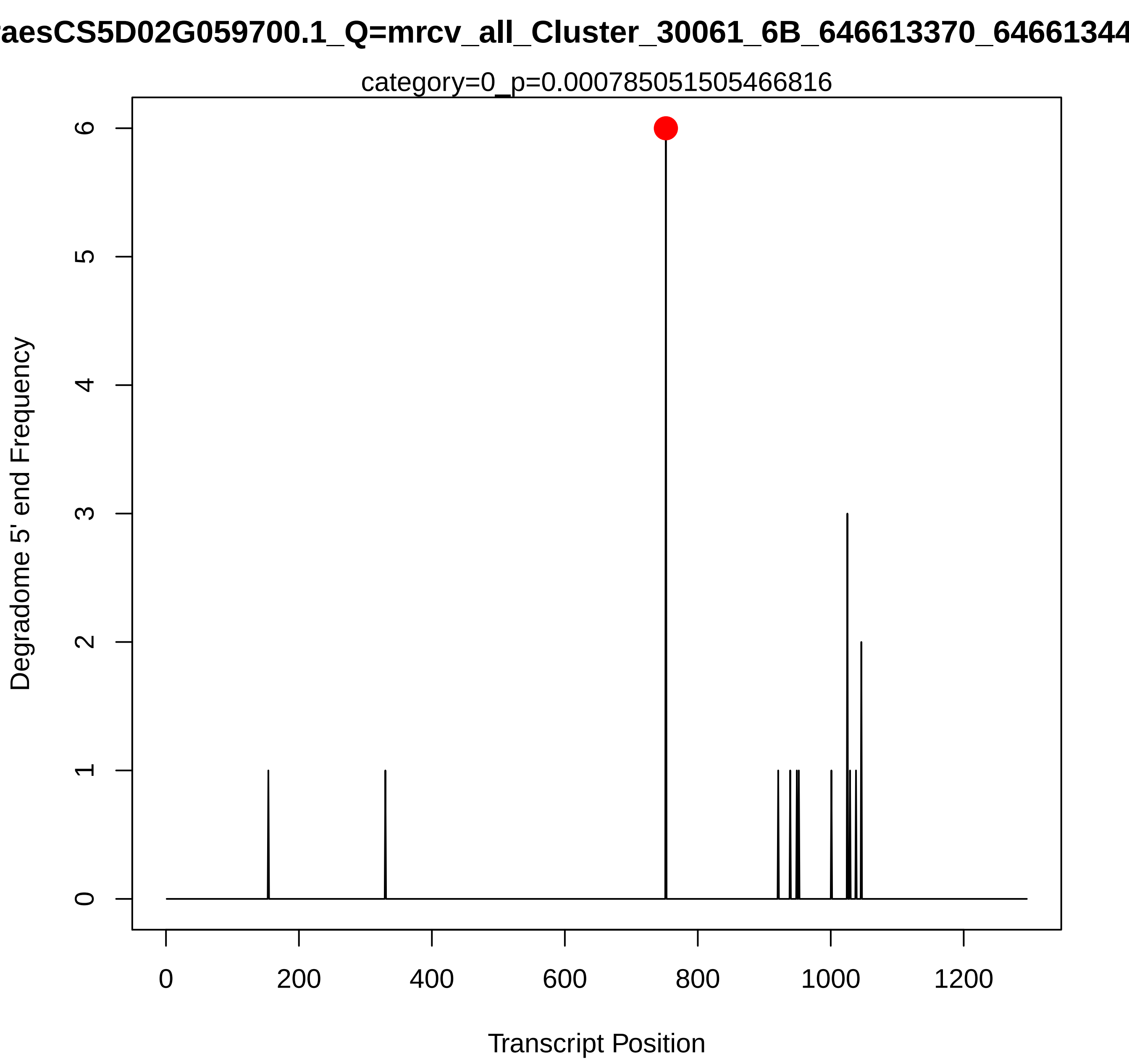
aesCS5D02G059700.1_Q=mrcv_all_Cluster_30061_6B_646613370_64661344
category=0_p=0.000785051505466816
Degradome 5' end Frequency
0
1
2
3
4
5
6
0
200
400
600
800
1000
1200
Transcript Position

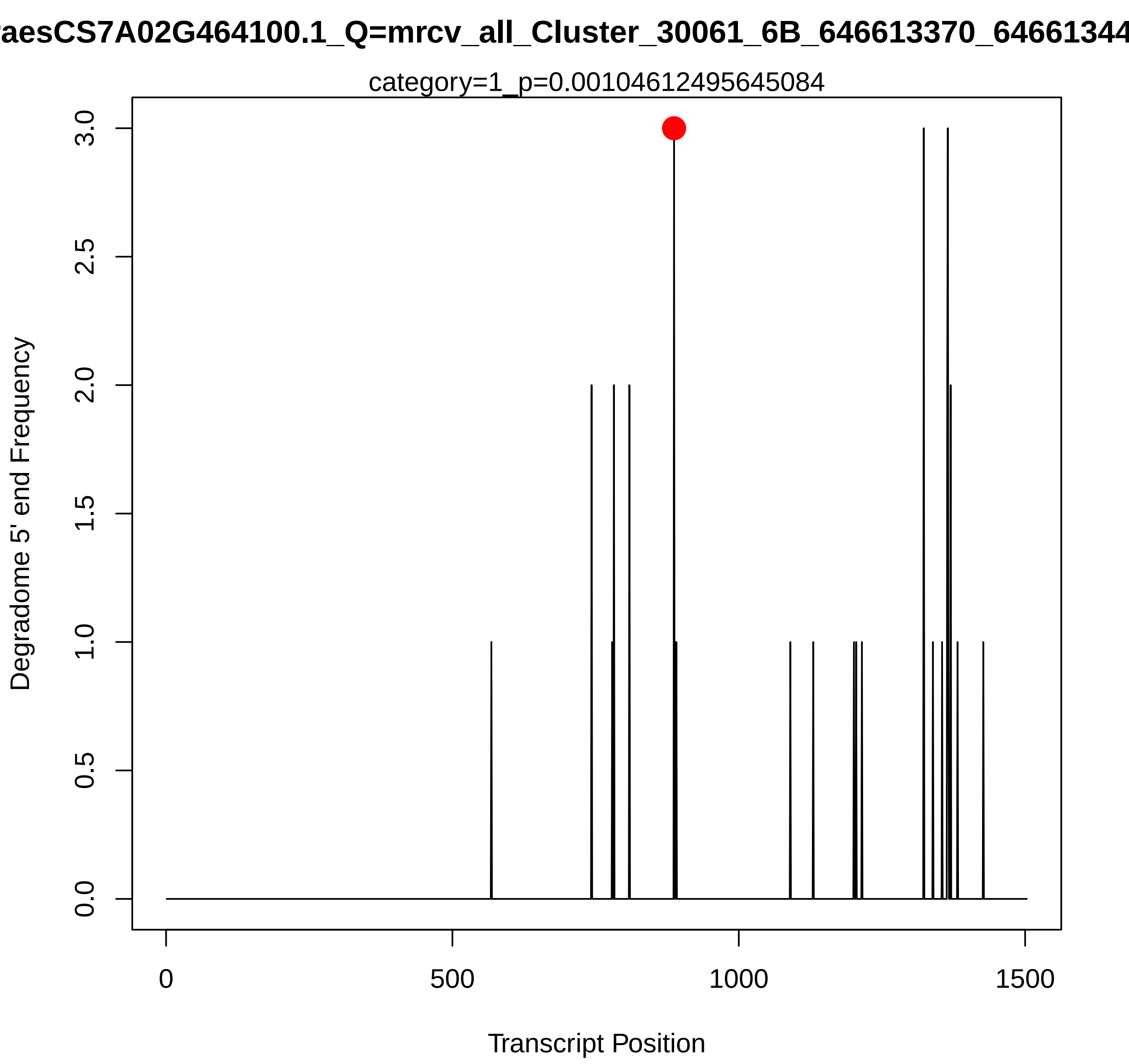

aesCS7A02G464100.1_Q=mrcv_all_Cluster_30061_6B_646613370_64661344
category=1_p=0.00104612495645084
Degradome 5' end Frequency
0.0
0.5
1.0
1.5
2.0
2.5
3.0
0
500
1000
1500
Transcript Position

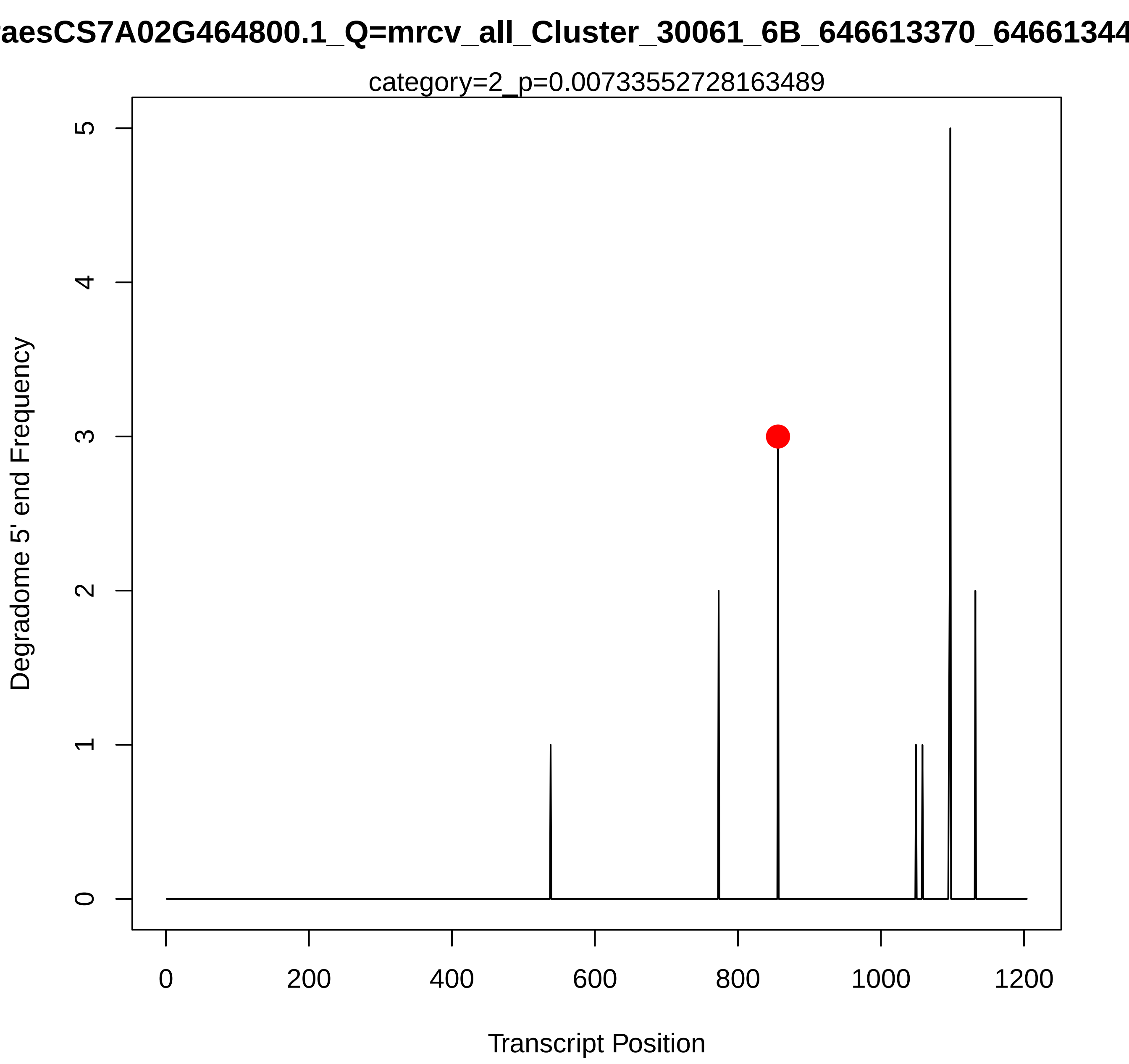
aesCS7A02G464800.1_Q=mrcv_all_Cluster_30061_6B_646613370_64661344
category=2_p=0.00733552728163489
Degradome 5' end Frequency
Transcript Position
0
1
2
3
4
5
0
200
400
600
800
1000
1200

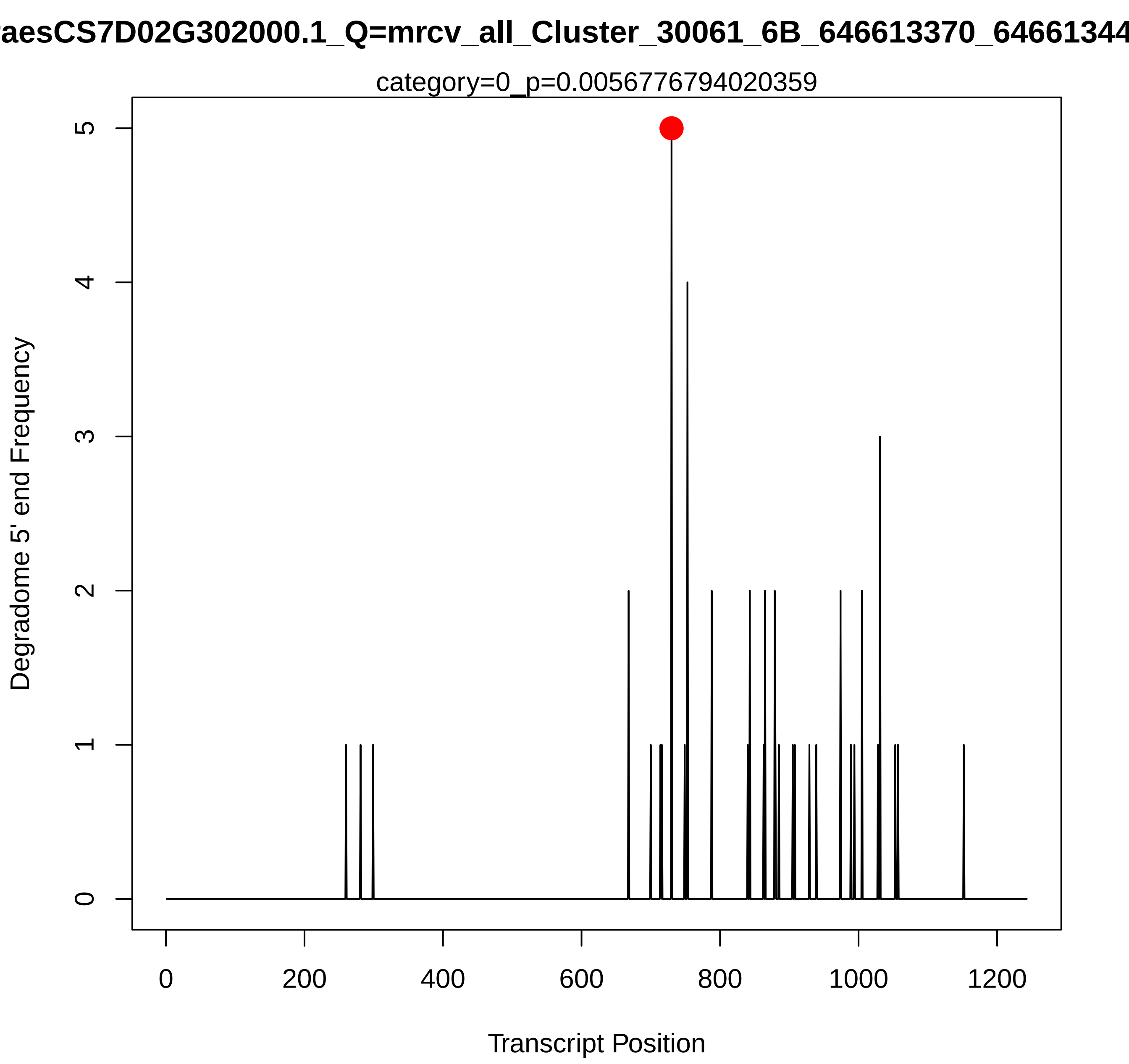

aesCS7D02G302000.1_Q=mrcv_all_Cluster_30061_6B_646613370_64661344
category=0_p=0.0056776794020359
Degradome 5' end Frequency
0
1
2
3
4
5
0
200
400
600
800
1000
1200
Transcript Position

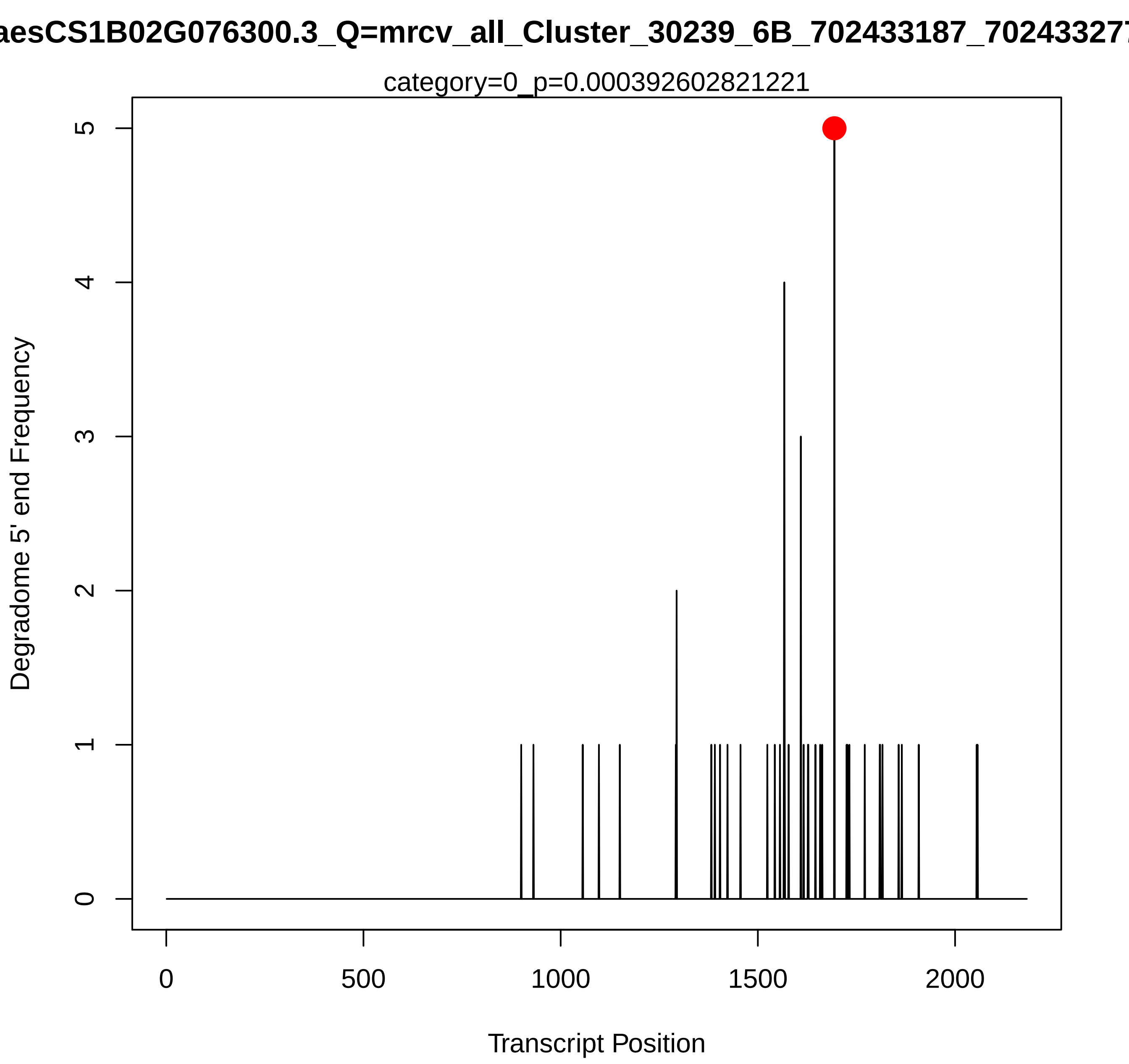

aesCS1B02G076300.3_Q=mrcv_all_Cluster_30239_6B_702433187_70243327
category=0_p=0.000392602821221
Degradome 5' end Frequency
0
1
2
3
4
5
0
500
1000
1500
2000
Transcript Position

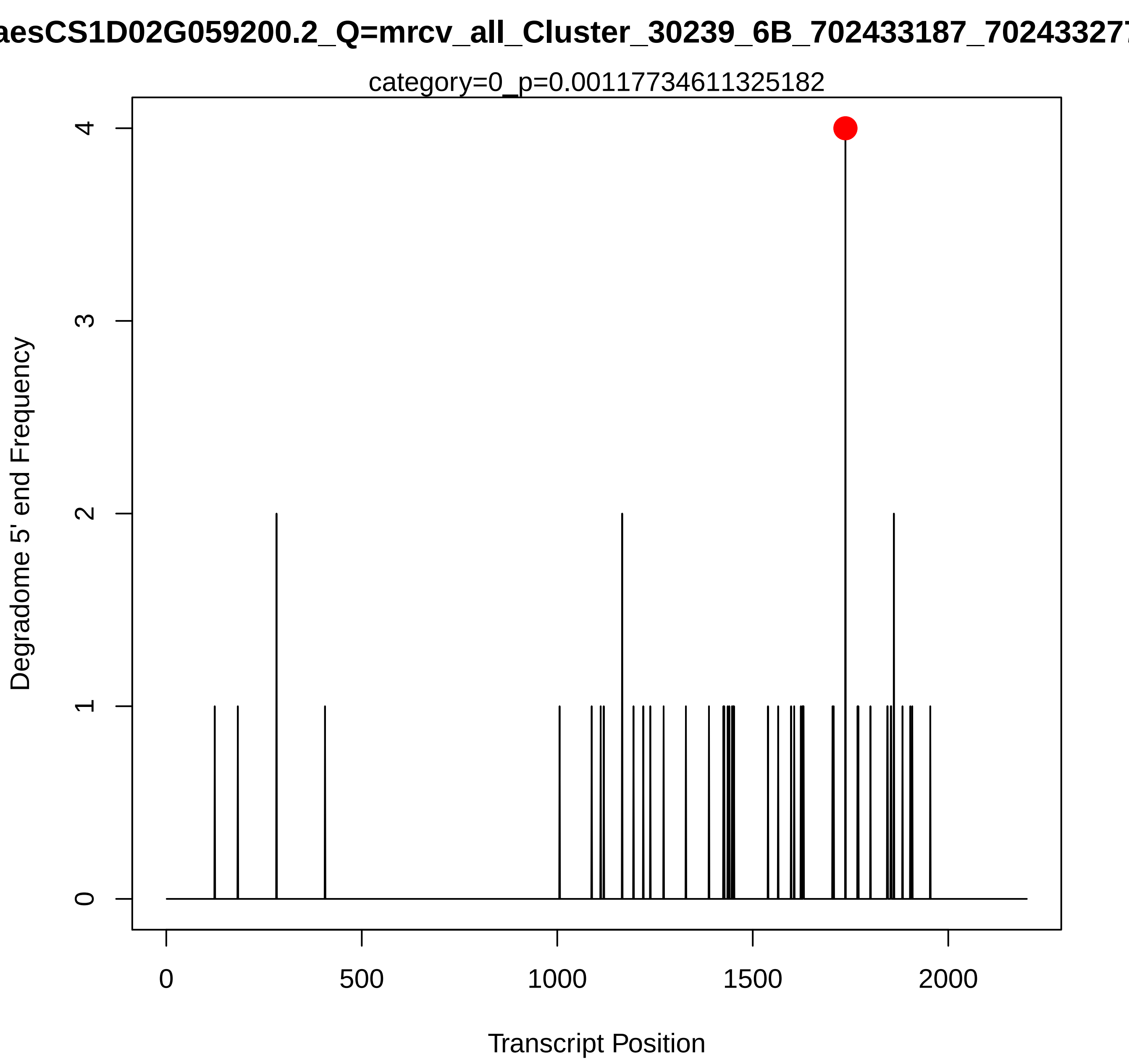

aesCS1D02G059200.2_Q=mrcv_all_Cluster_30239_6B_702433187_70243327
category=0_p=0.00117734611325182
Degradome 5' end Frequency
Transcript Position
0
1
2
3
4
0
500
1000
1500
2000

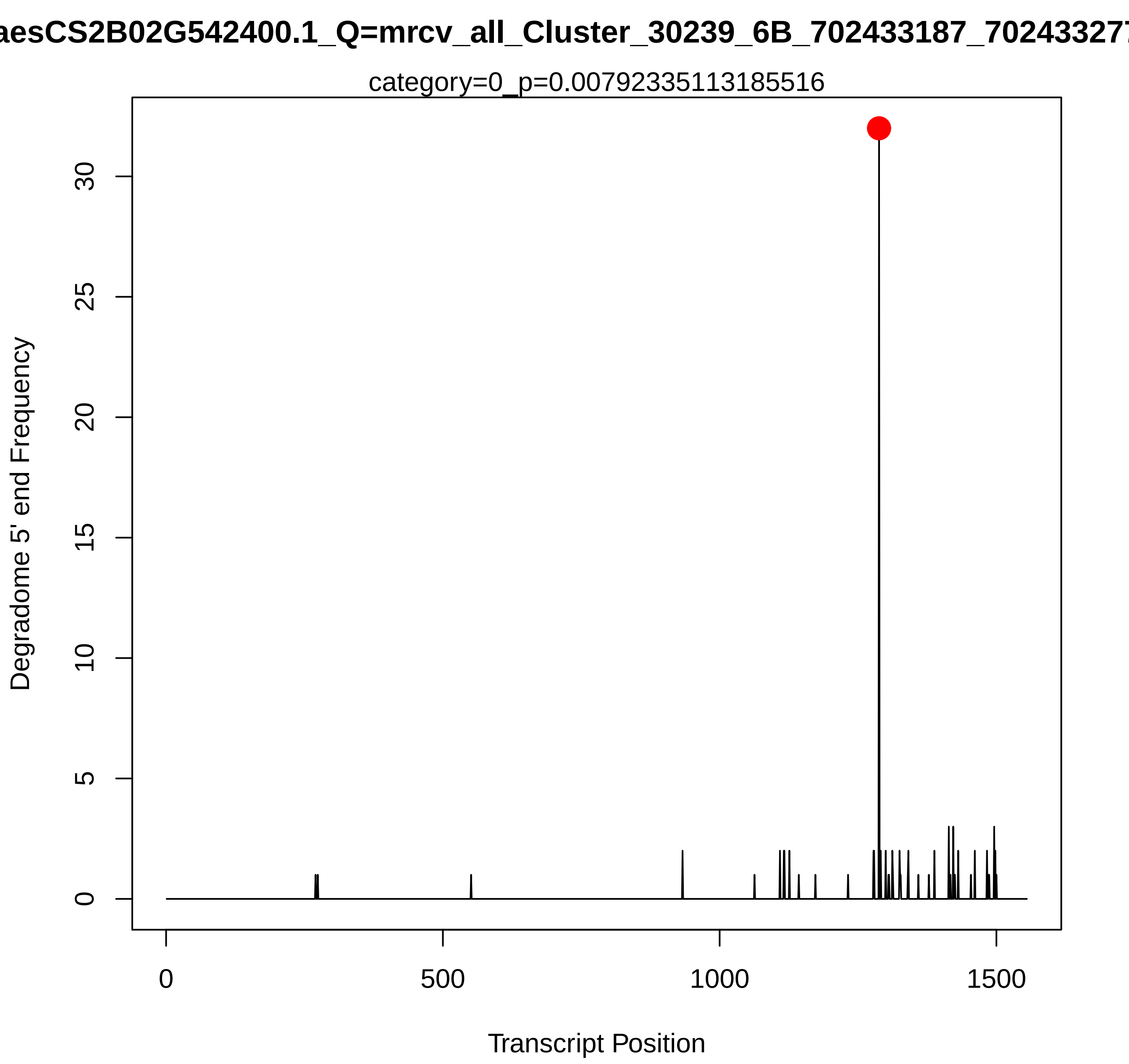

aesCS2B02G542400.1_Q=mrcv_all_Cluster_30239_6B_702433187_70243327
category=0_p=0.00792335113185516
Degradome 5' end Frequency
Transcript Position
0
5
10
15
20
25
30
0
500
1000
1500

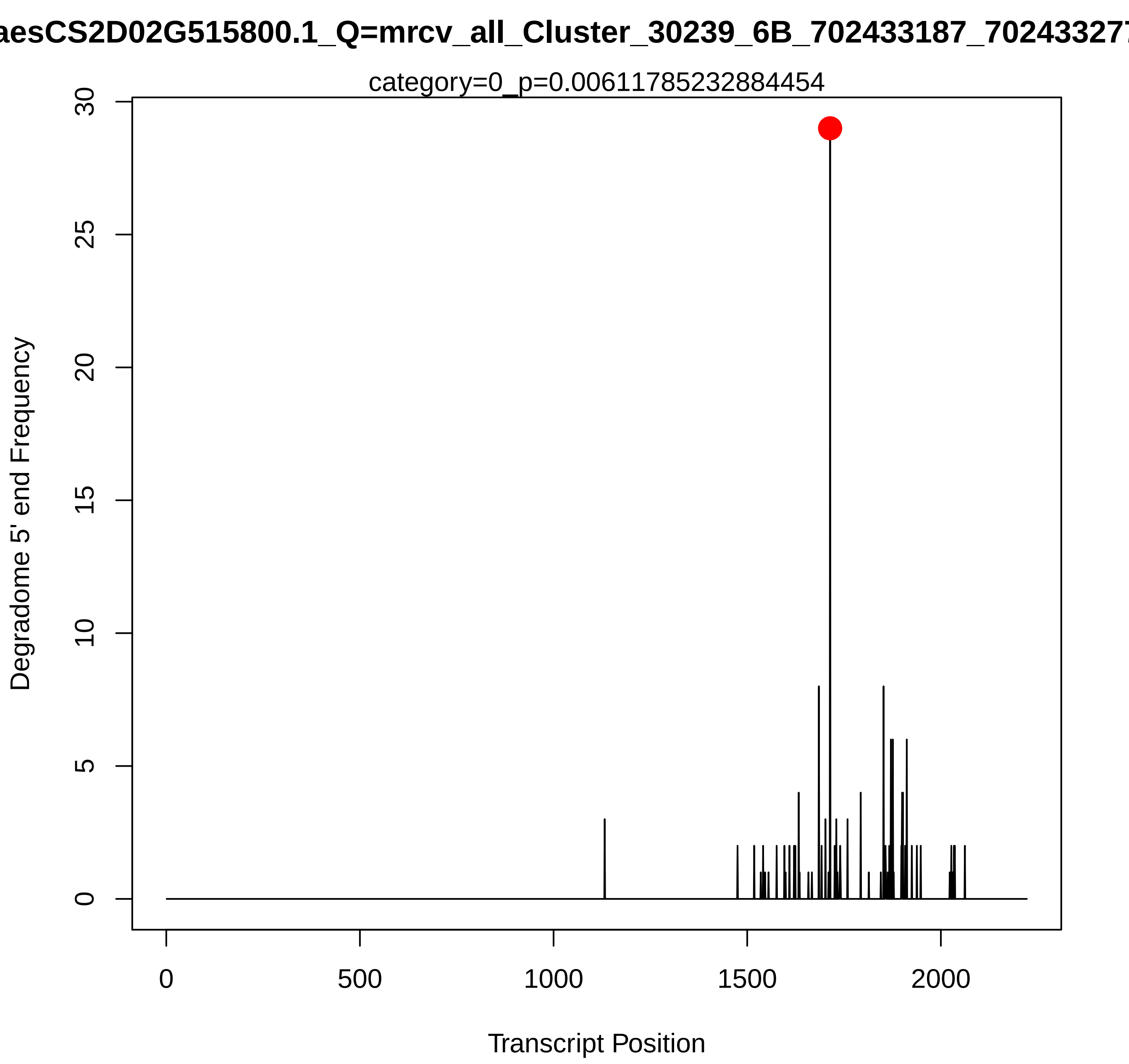
aesCS2D02G515800.1_Q=mrcv_all_Cluster_30239_6B_702433187_70243327
category=0_p=0.00611785232884454
Degradome 5' end Frequency
Transcript Position
0
5
10
15
20
25
30
0
500
1000
1500
2000

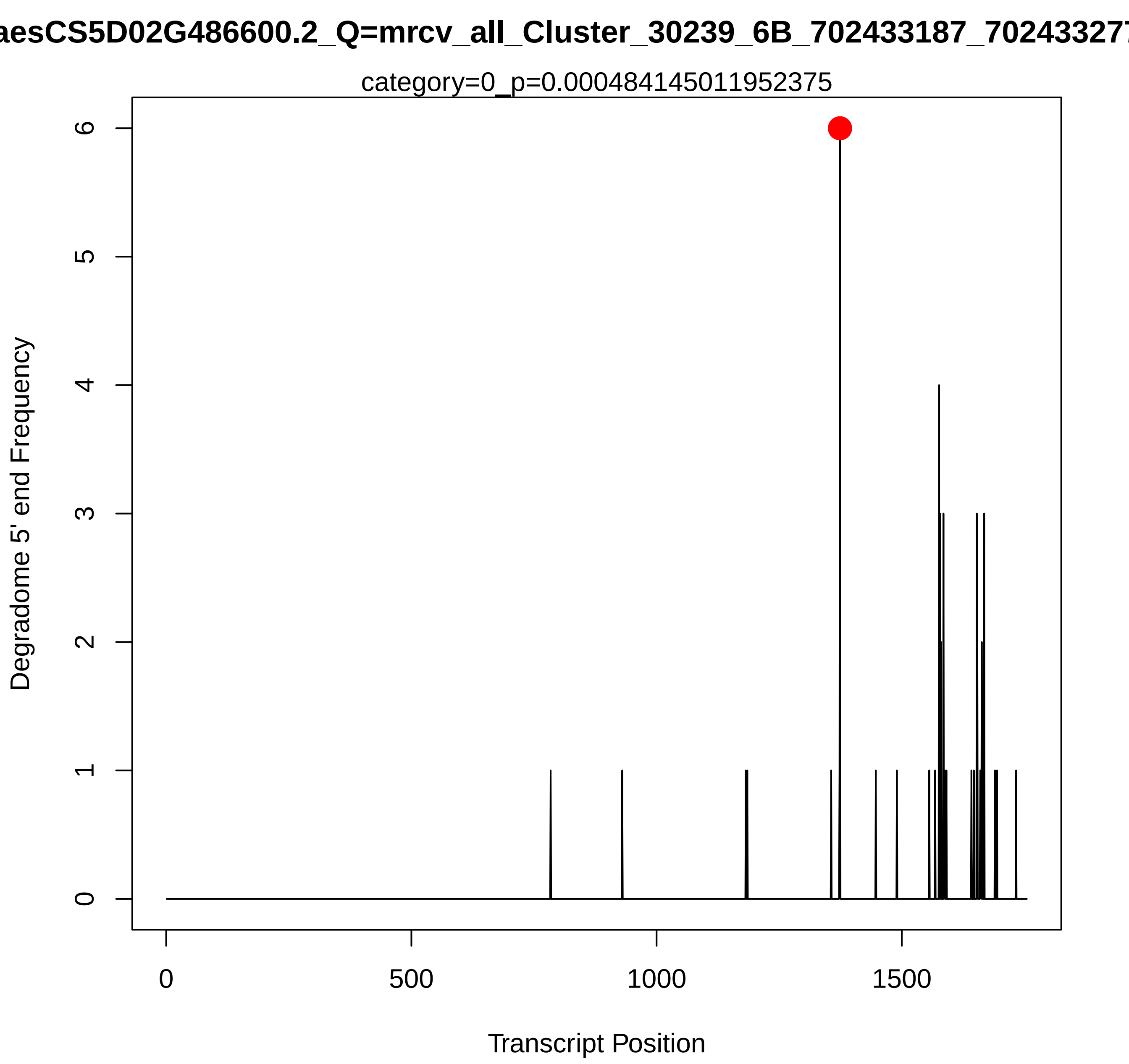
aesCS5D02G486600.2_Q=mrcv_all_Cluster_30239_6B_702433187_70243327
category=0_p=0.000484145011952375
Degradome 5' end Frequency
Transcript Position
0
1
2
3
4
5
6
0
500
1000
1500

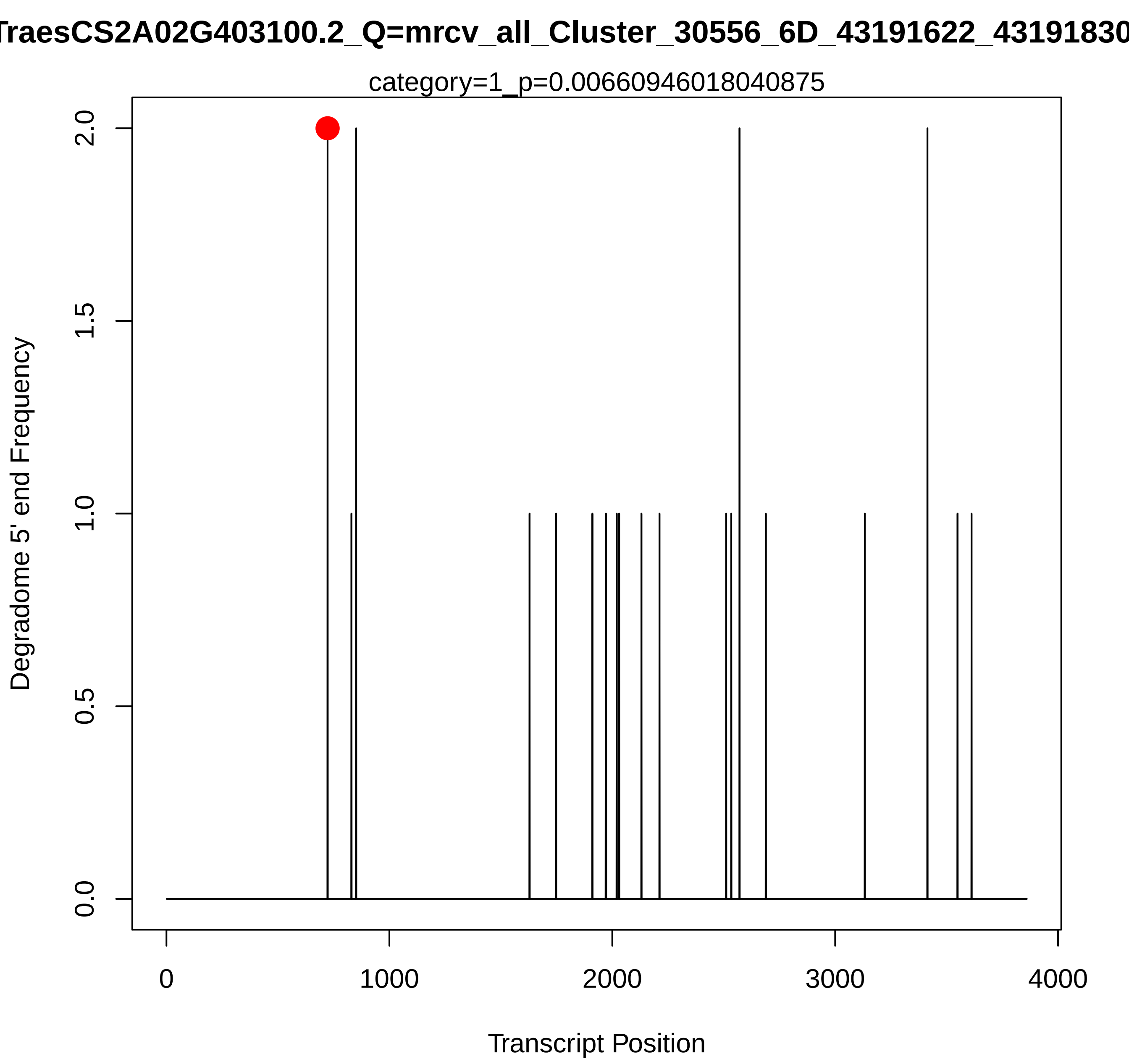
TraesCS2A02G403100.2_Q=mrcv_all_Cluster_30556_6D_43191622_43191830
category=1_p=0.00660946018040875
Degradome 5' end Frequency
Transcript Position
0.0
0.5
1.0
1.5
2.0
0
1000
2000
3000
4000

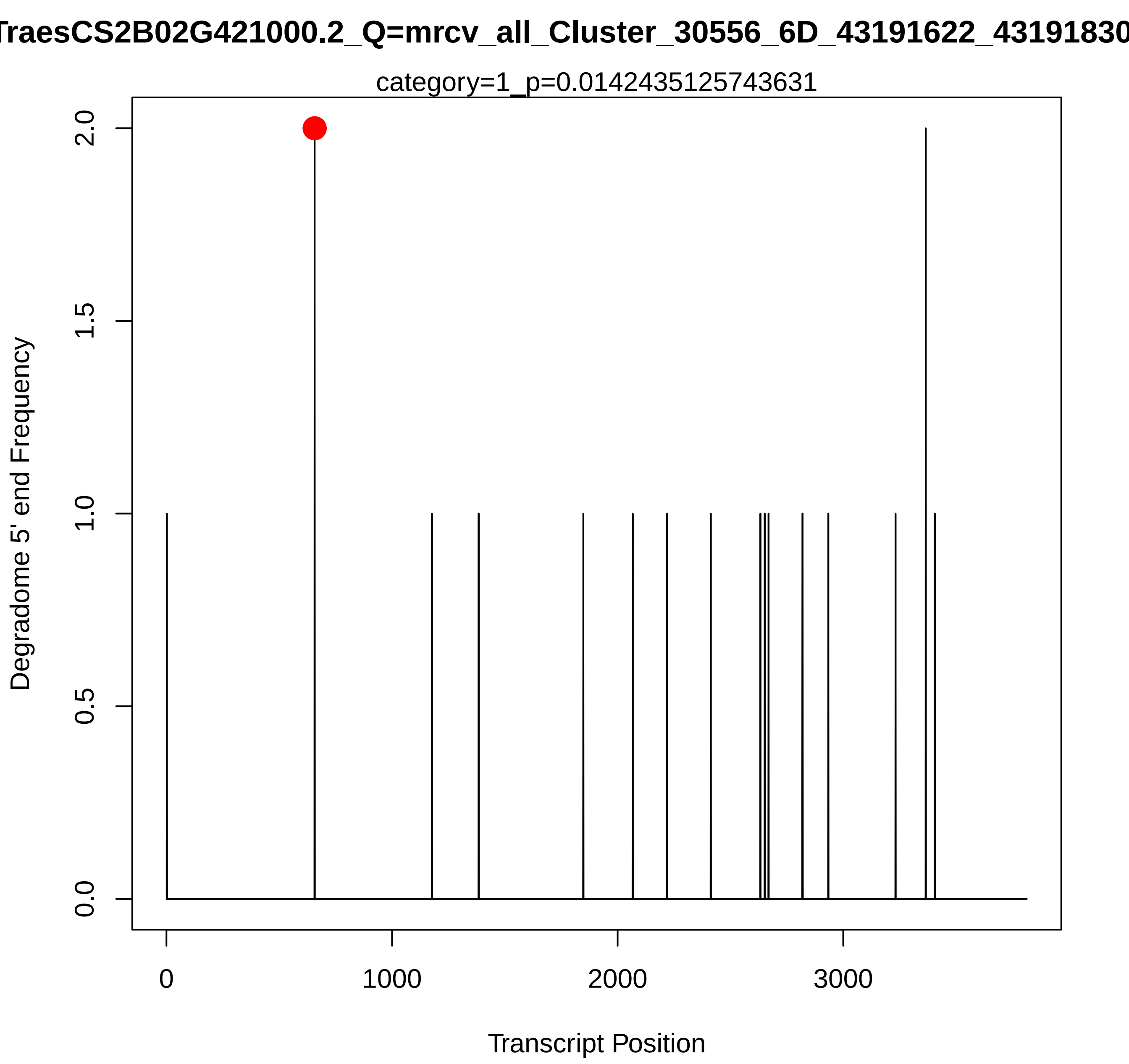

TraesCS2B02G421000.2_Q=mrcv_all_Cluster_30556_6D_43191622_43191830
category=1_p=0.0142435125743631
Degradome 5' end Frequency
0.0
0.5
1.0
1.5
2.0
0
1000
2000
3000
Transcript Position

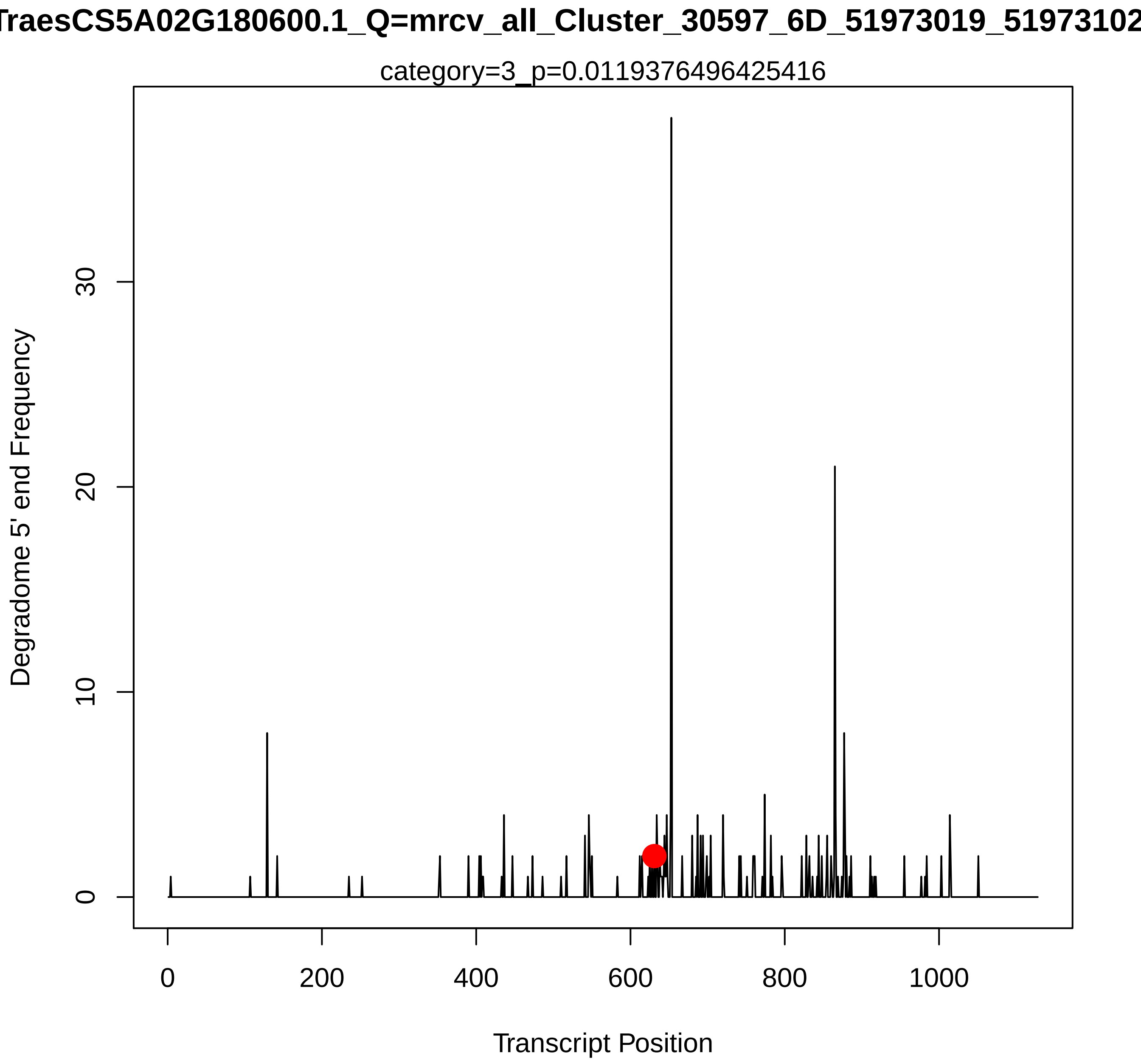
TraesCS5A02G180600.1_Q=mrcv_all_Cluster_30597_6D_51973019_51973102
category=3_p=0.0119376496425416
Degradome 5' end Frequency
Transcript Position
0
10
20
30
0
200
400
600
800
1000

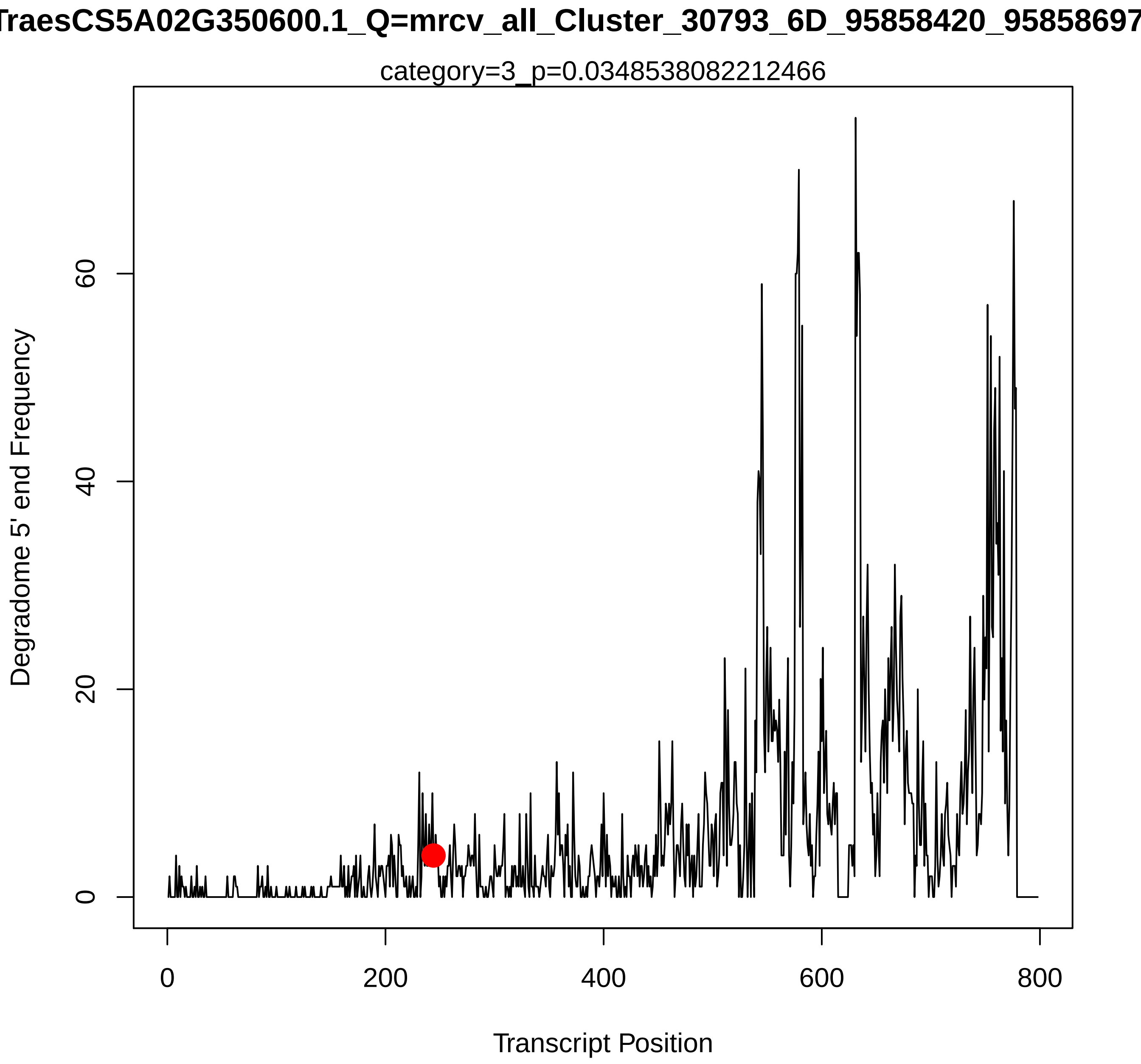
TraesCS5A02G350600.1_Q=mrcv_all_Cluster_30793_6D_95858420_95858697
category=3_p=0.0348538082212466
Degradome 5' end Frequency
0
20
40
60
0
200
400
600
800
Transcript Position

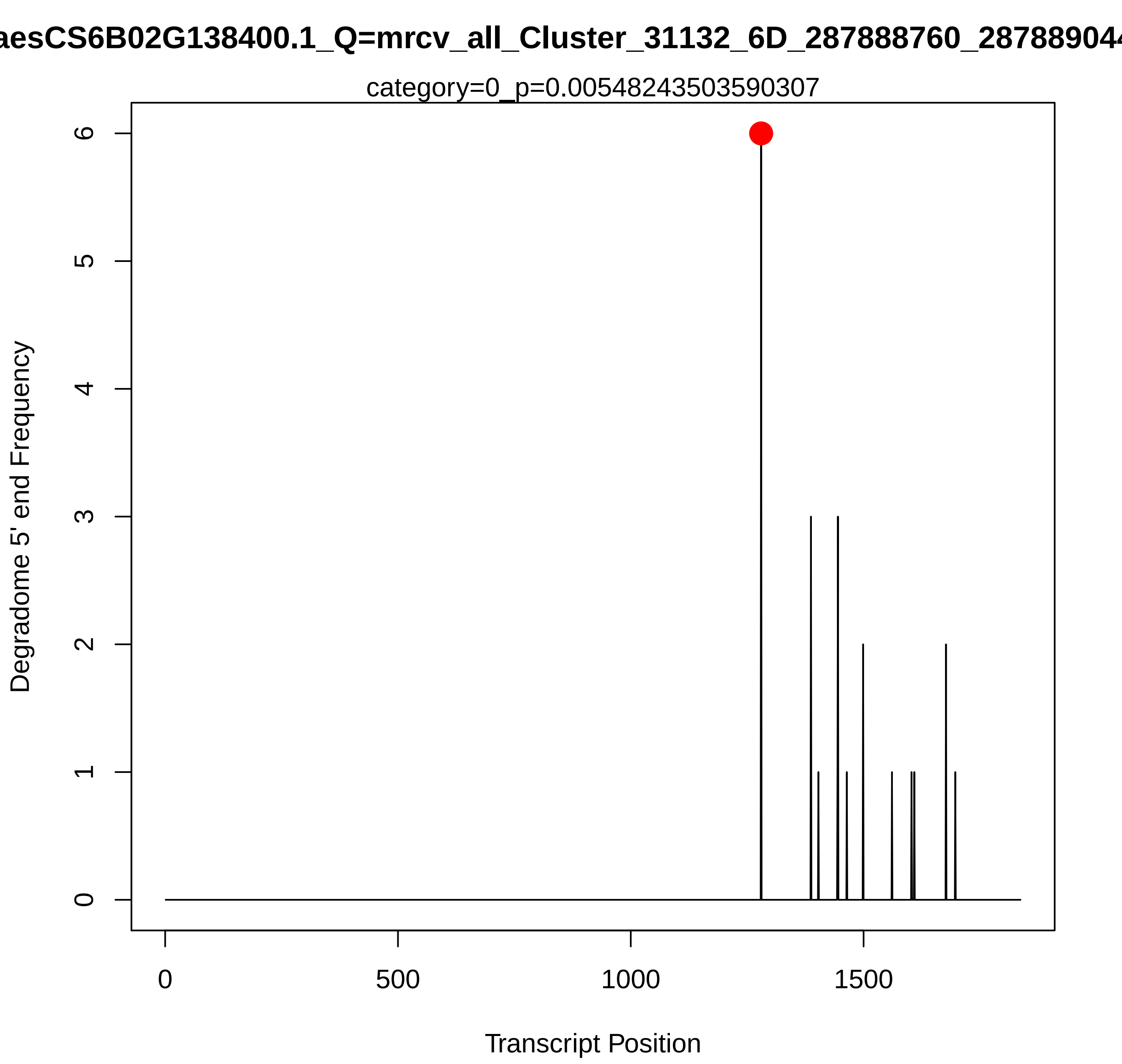
aesCS6B02G138400.1_Q=mrcv_all_Cluster_31132_6D_287888760_28788904
category=0_p=0.00548243503590307
Degradome 5' end Frequency
0
1
2
3
4
5
6
0
500
1000
1500
Transcript Position

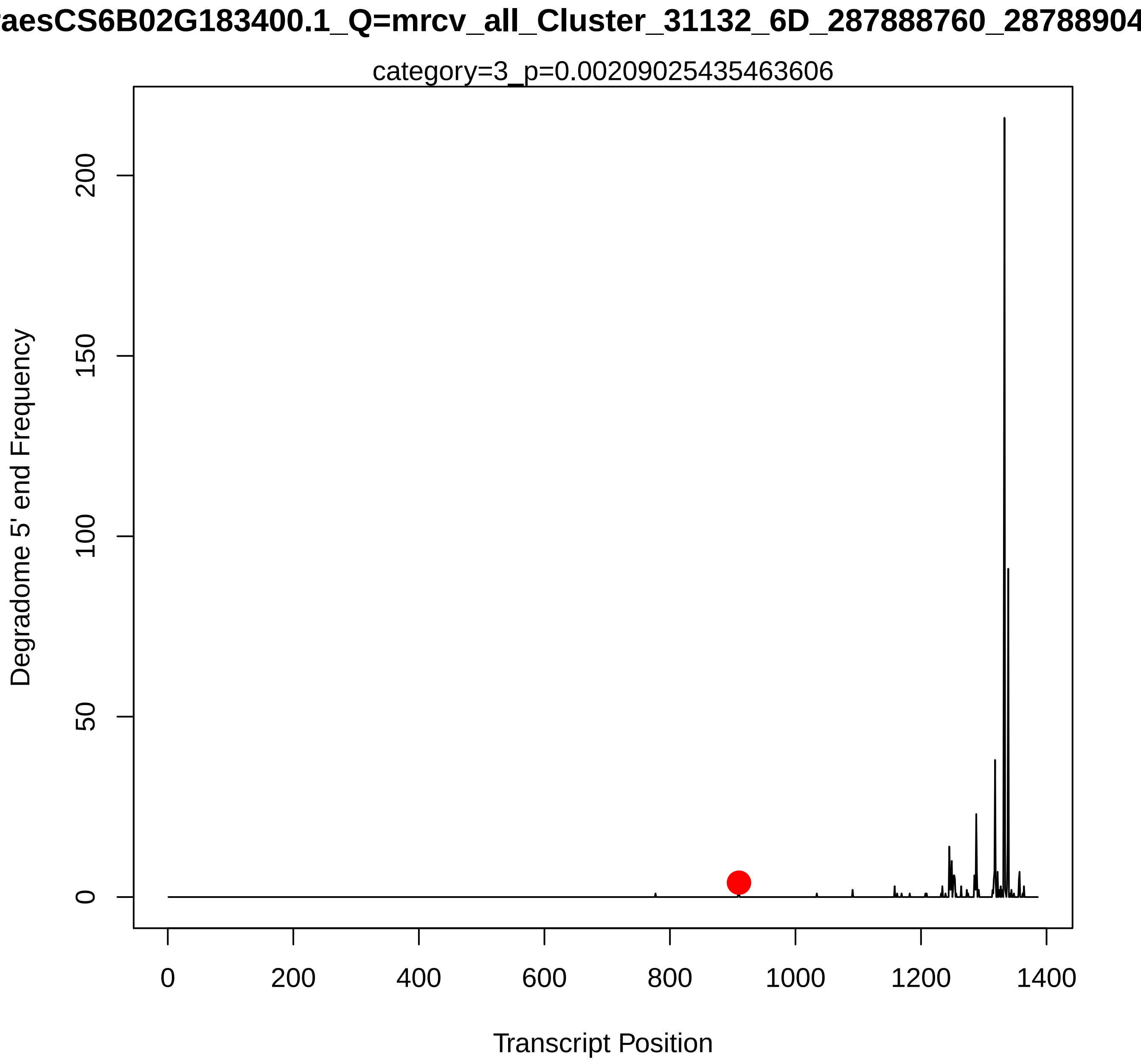
aesCS6B02G183400.1_Q=mrcv_all_Cluster_31132_6D_287888760_28788904
category=3_p=0.00209025435463606
Degradome 5' end Frequency
0
50
100
150
200
0
200
400
600
800
1000
1200
1400
Transcript Position

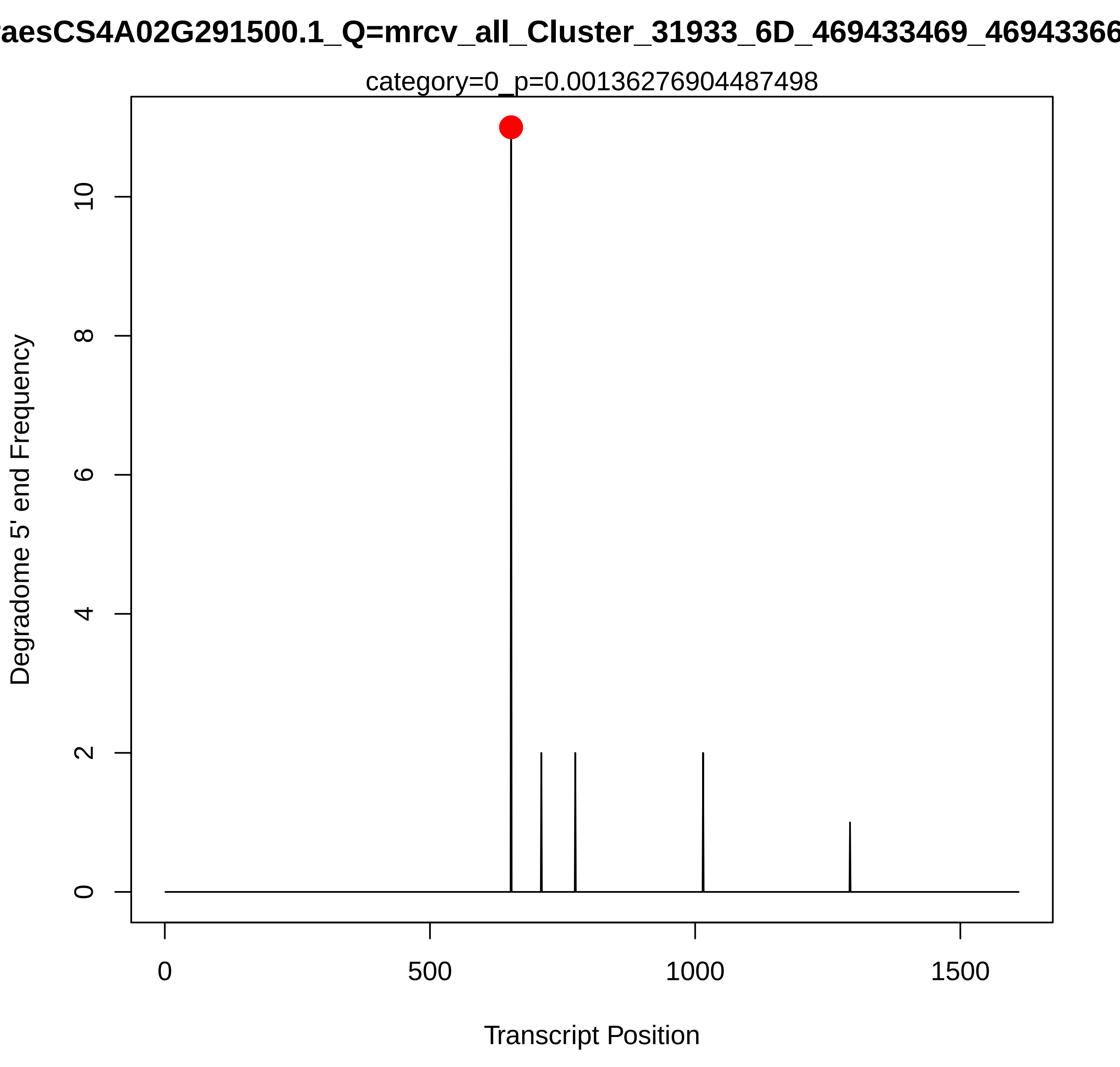

aesCS4A02G291500.1_Q=mrcv_all_Cluster_31933_6D_469433469_4694336
category=0_p=0.00136276904487498
Degradome 5' end Frequency
0
2
4
6
8
10
0
500
1000
1500
Transcript Position

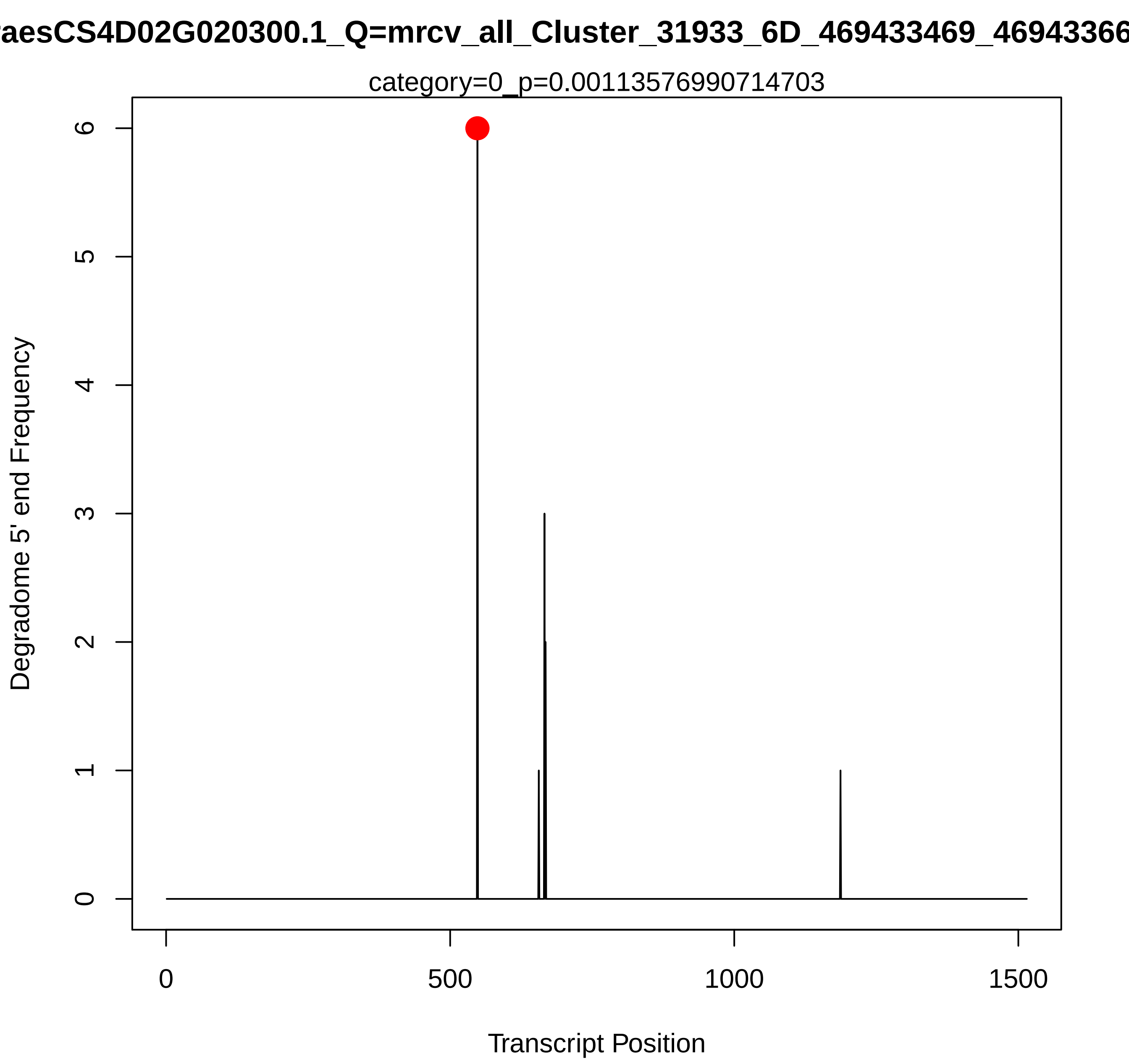
aesCS4D02G020300.1_Q=mrcv_all_Cluster_31933_6D_469433469_46943366
category=0_p=0.00113576990714703
Degradome 5' end Frequency
0
1
2
3
4
5
6
0
500
1000
1500
Transcript Position

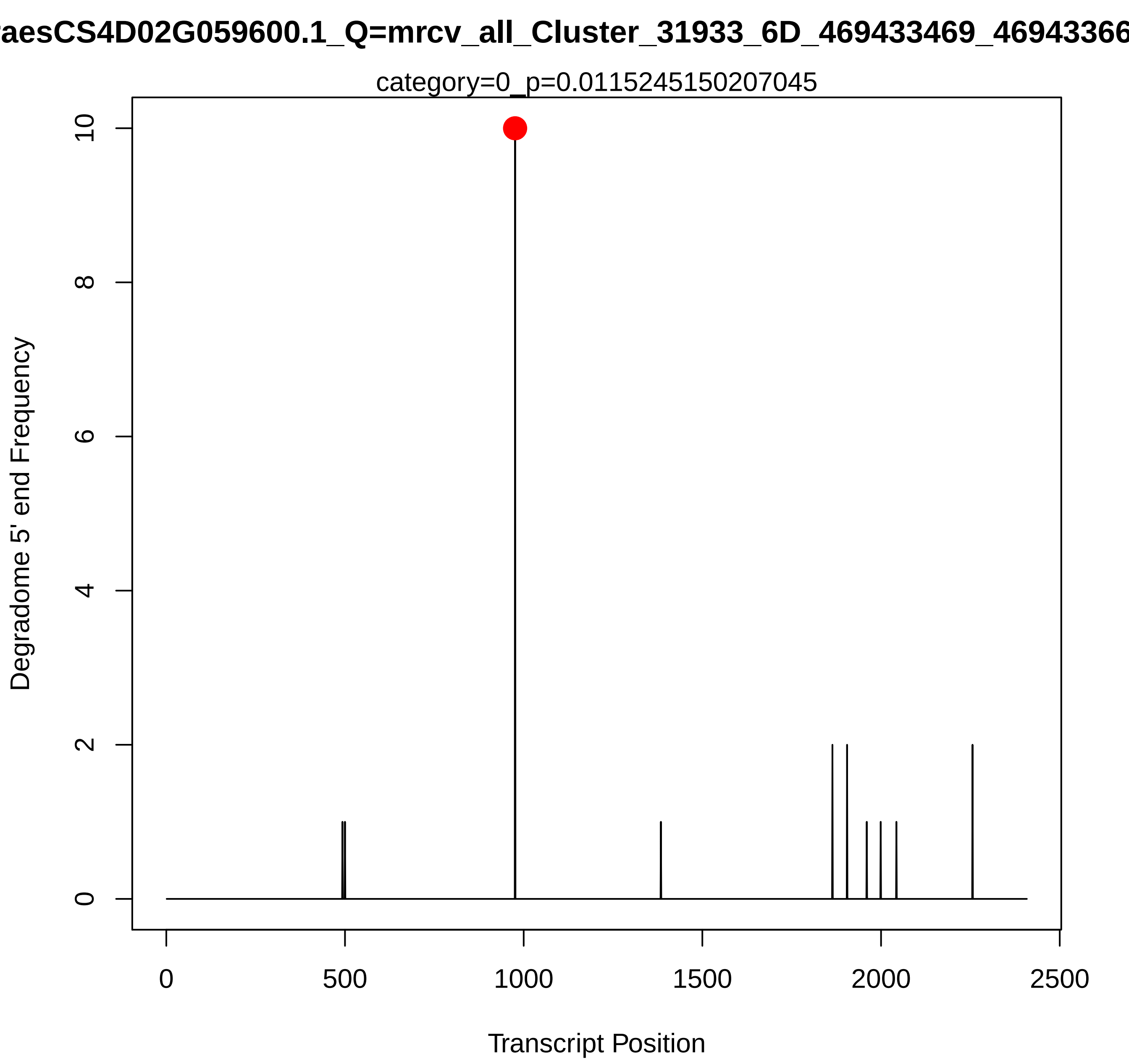
aesCS4D02G059600.1_Q=mrcv_all_Cluster_31933_6D_469433469_46943366
category=0_p=0.0115245150207045
Degradome 5' end Frequency
Transcript Position
0
2
4
6
8
10
0
500
1000
1500
2000
2500

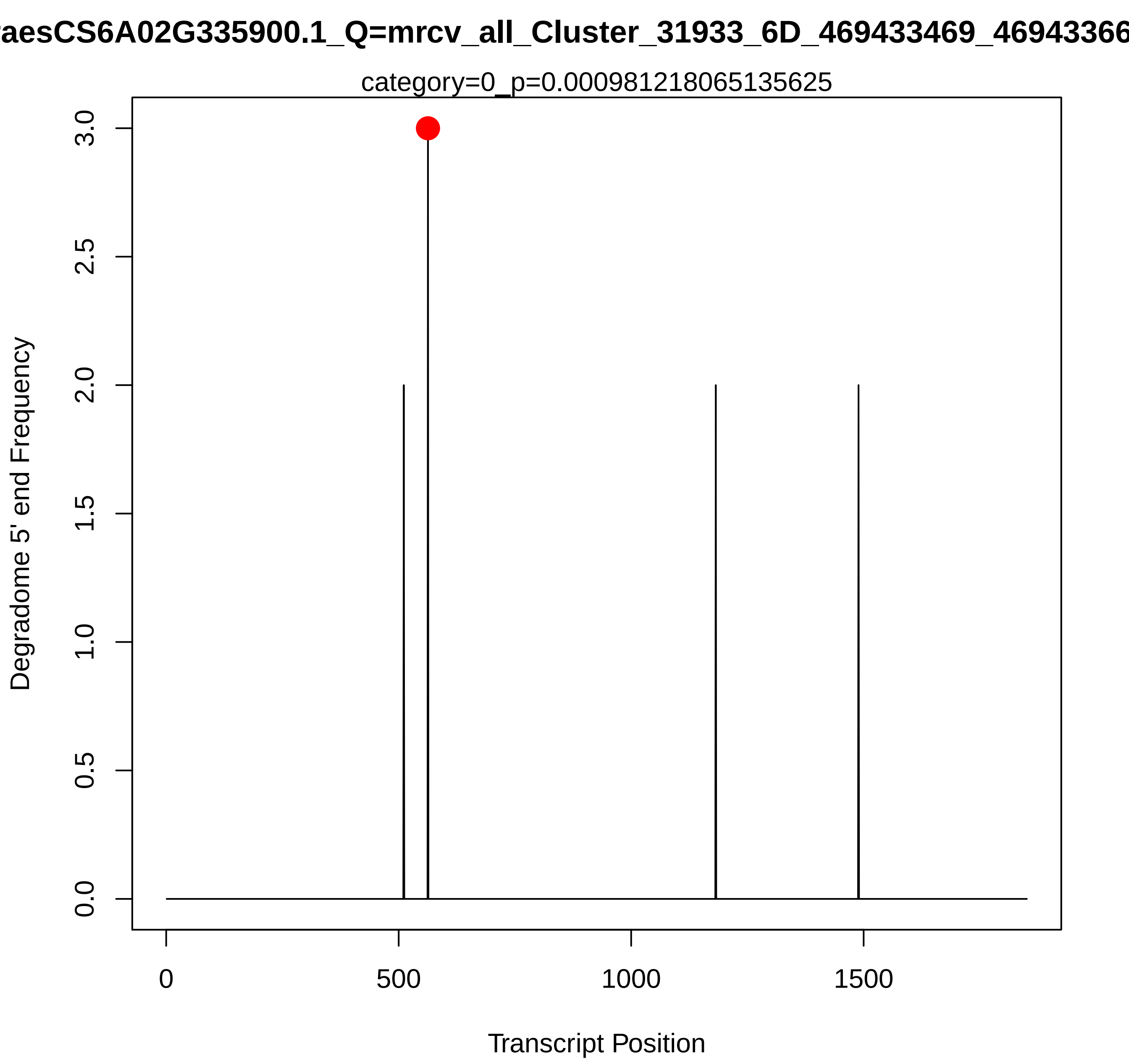

aesCS6A02G335900.1_Q=mrcv_all_Cluster_31933_6D_469433469_4694336
category=0_p=0.000981218065135625
Degradome 5' end Frequency
0.0
0.5
1.0
1.5
2.0
2.5
3.0
0
500
1000
1500
Transcript Position

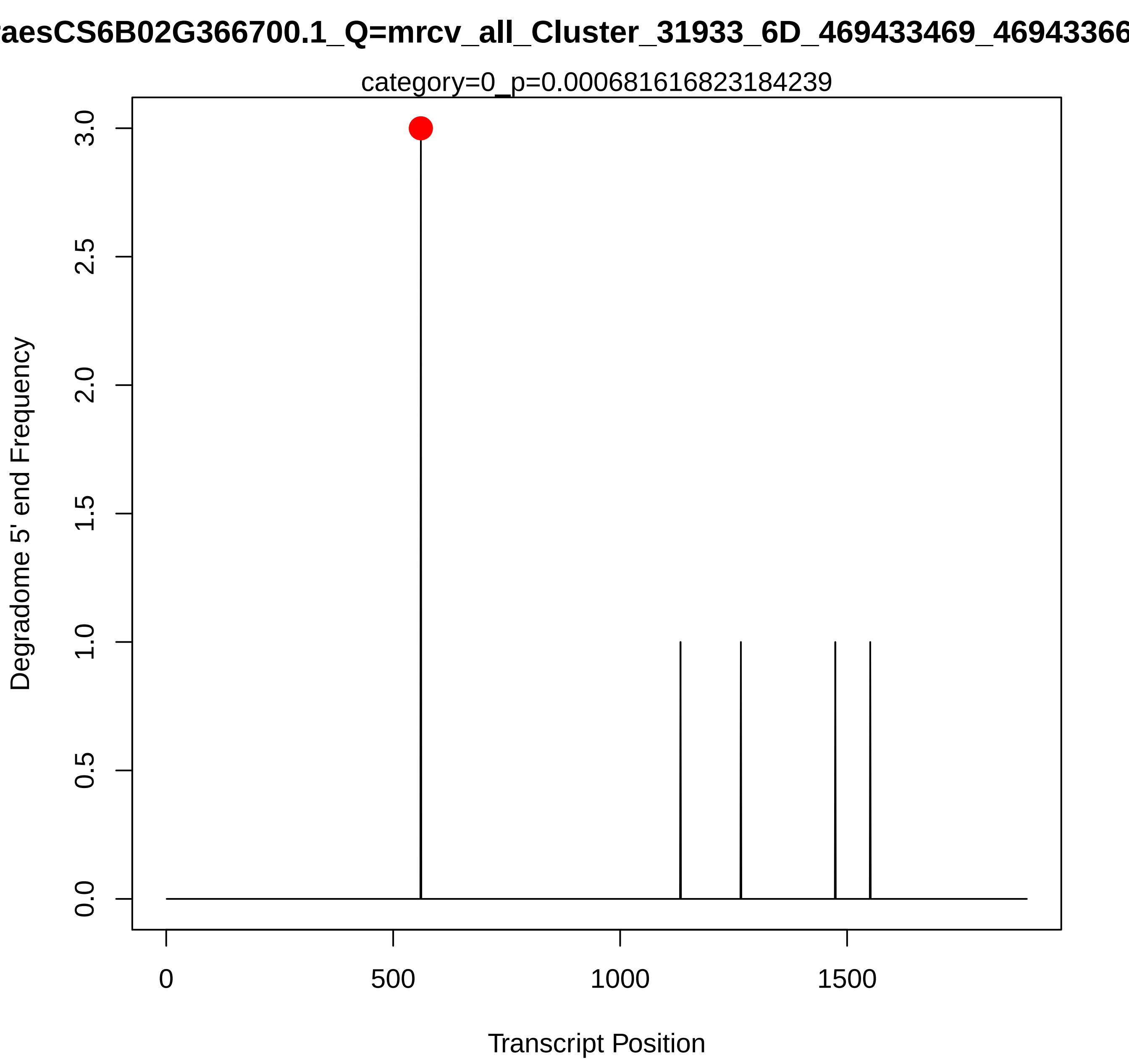
aesCS6B02G366700.1_Q=mrcv_all_Cluster_31933_6D_469433469_46943366
category=0_p=0.000681616823184239
Degradome 5' end Frequency
0.0
0.5
1.0
1.5
2.0
2.5
3.0
0
500
1000
1500
Transcript Position

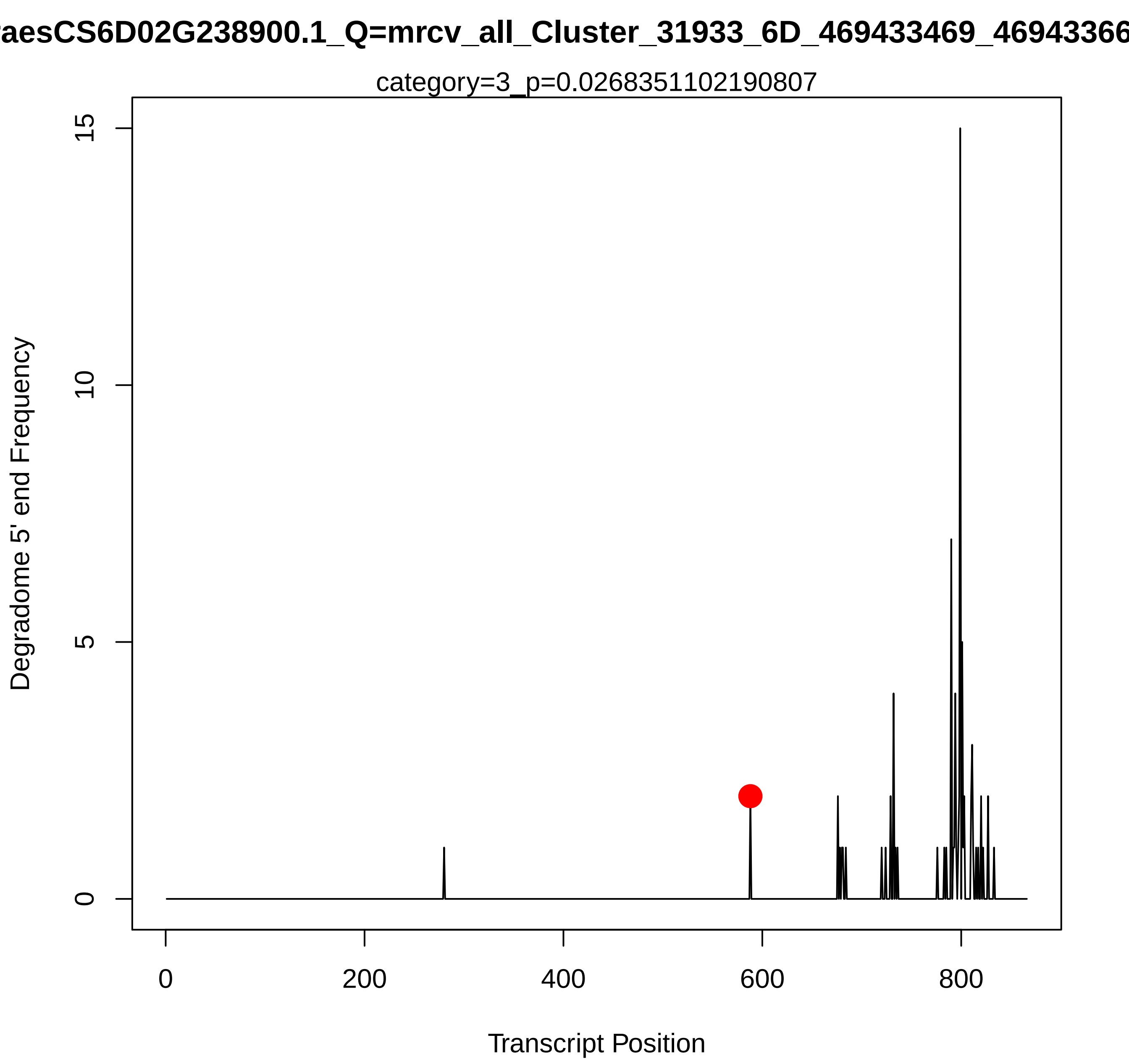

aesCS6D02G238900.1_Q=mrcv_all_Cluster_31933_6D_469433469_4694336
category=3_p=0.0268351102190807
Degradome 5' end Frequency
0
5
10
15
0
200
400
600
800
Transcript Position

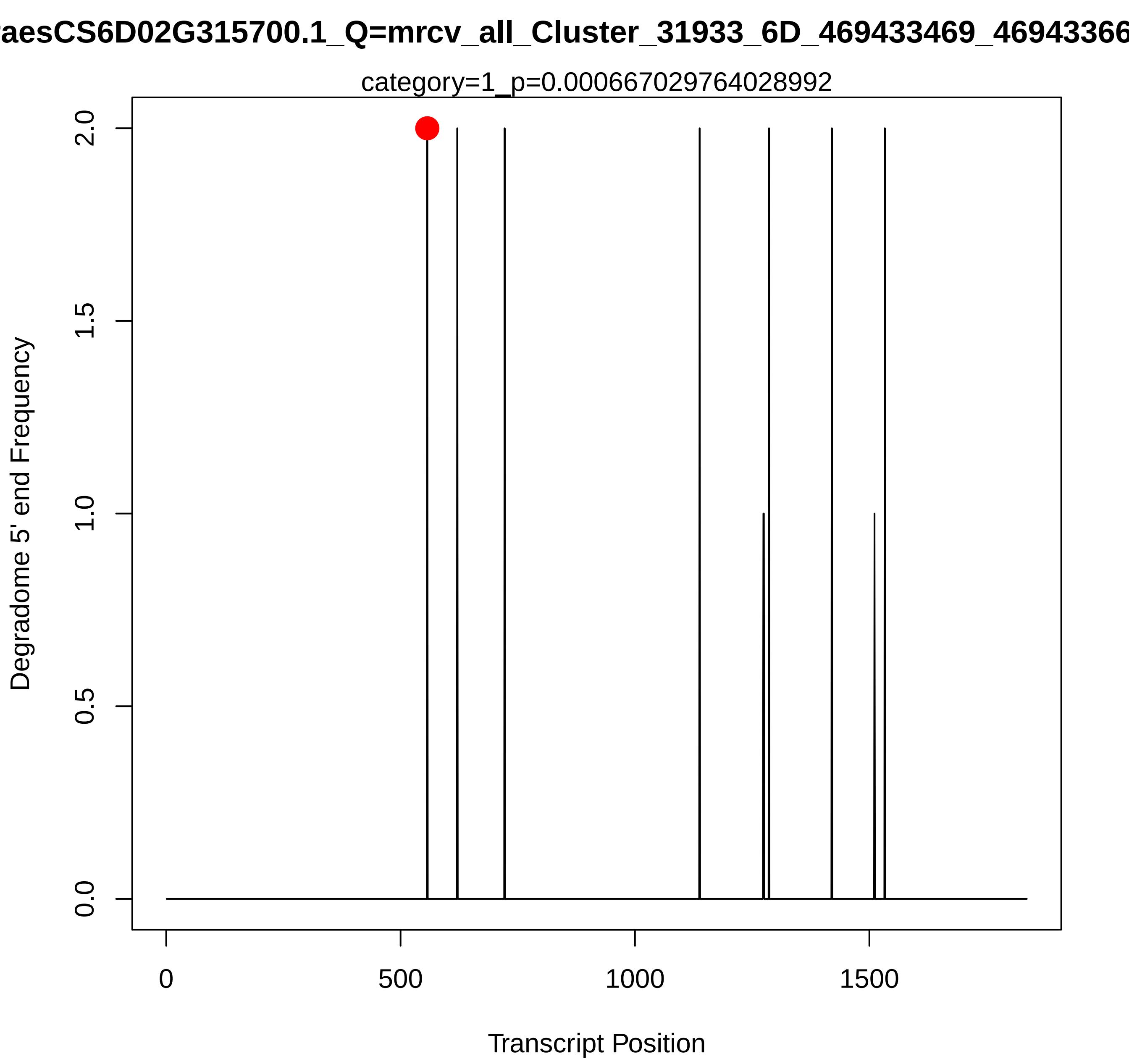
aesCS6D02G315700.1_Q=mrcv_all_Cluster_31933_6D_469433469_46943366
category=1_p=0.000667029764028992
Degradome 5' end Frequency
0.0
0.5
1.0
1.5
2.0
0
500
1000
1500
Transcript Position

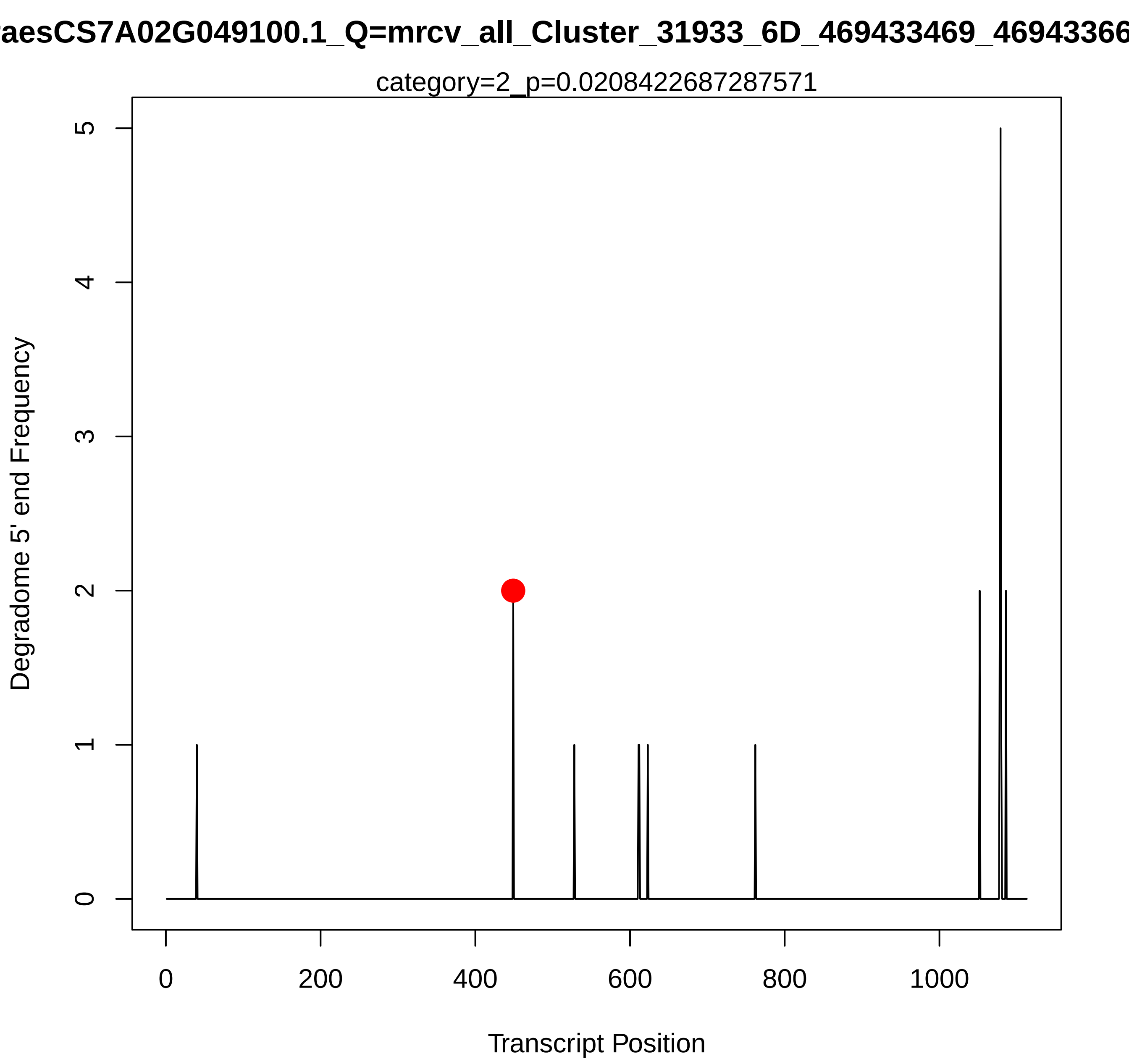

aesCS7A02G049100.1_Q=mrcv_all_Cluster_31933_6D_469433469_4694336
category=2_p=0.0208422687287571
Degradome 5' end Frequency
0
1
2
3
4
5
0
200
400
600
800
1000
Transcript Position

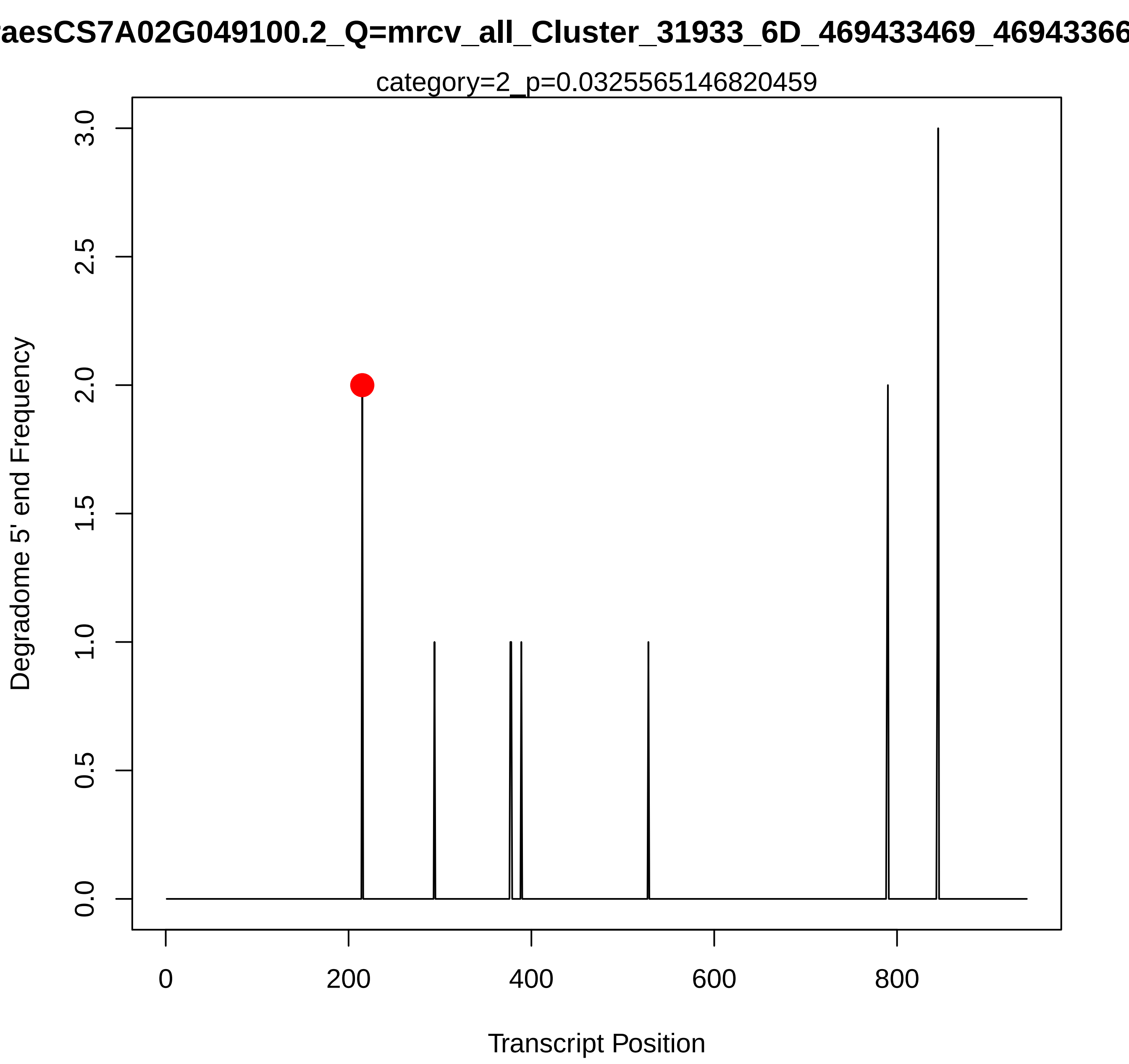

aesCS7A02G049100.2_Q=mrcv_all_Cluster_31933_6D_469433469_46943366
category=2_p=0.0325565146820459
Degradome 5' end Frequency
0.0
0.5
1.0
1.5
2.0
2.5
3.0
0
200
400
600
800
Transcript Position

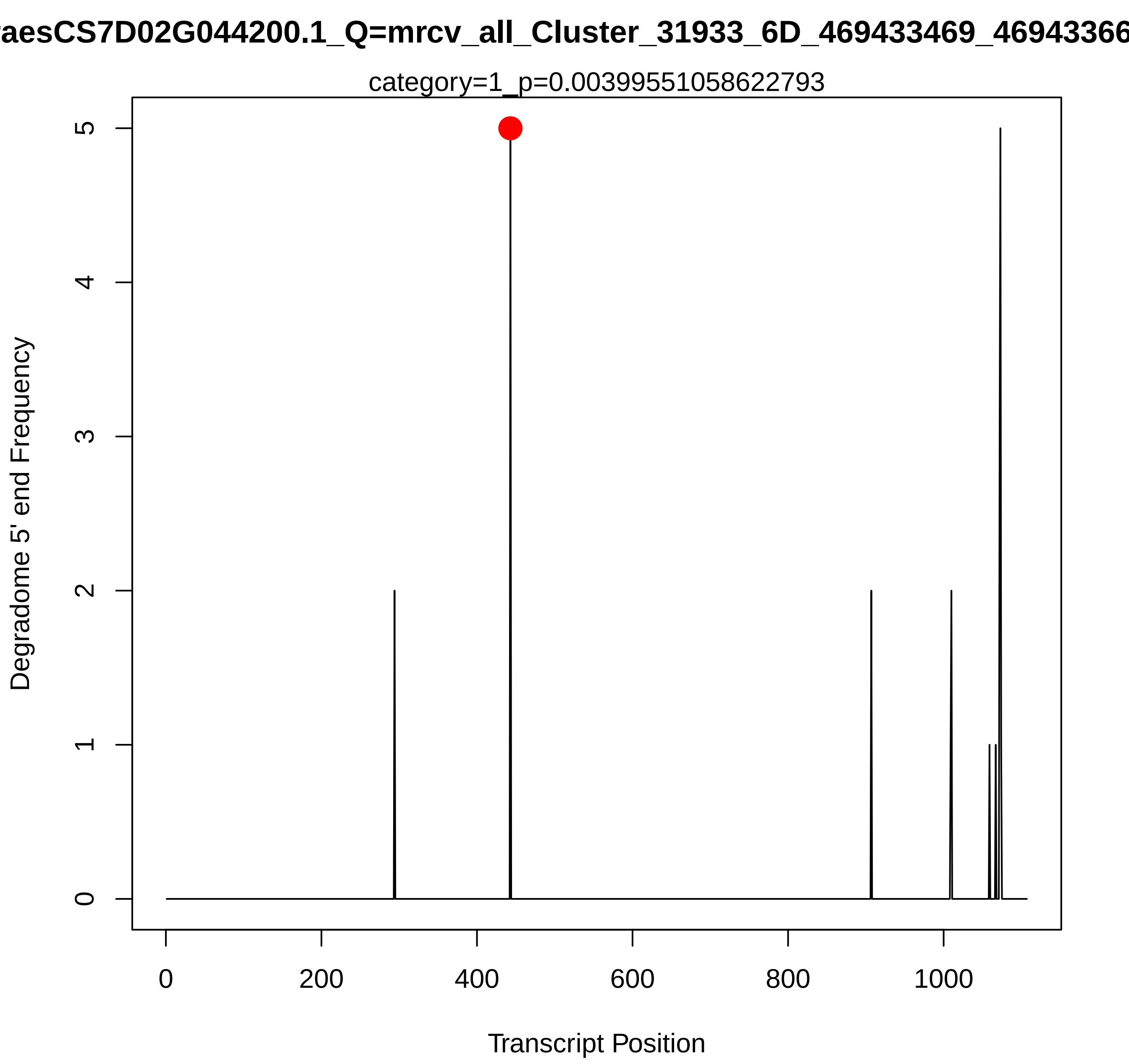

aesCS7D02G044200.1_Q=mrcv_all_Cluster_31933_6D_469433469_46943366
category=1_p=0.00399551058622793
Degradome 5' end Frequency
Transcript Position
0
1
2
3
4
5
0
200
400
600
800
1000

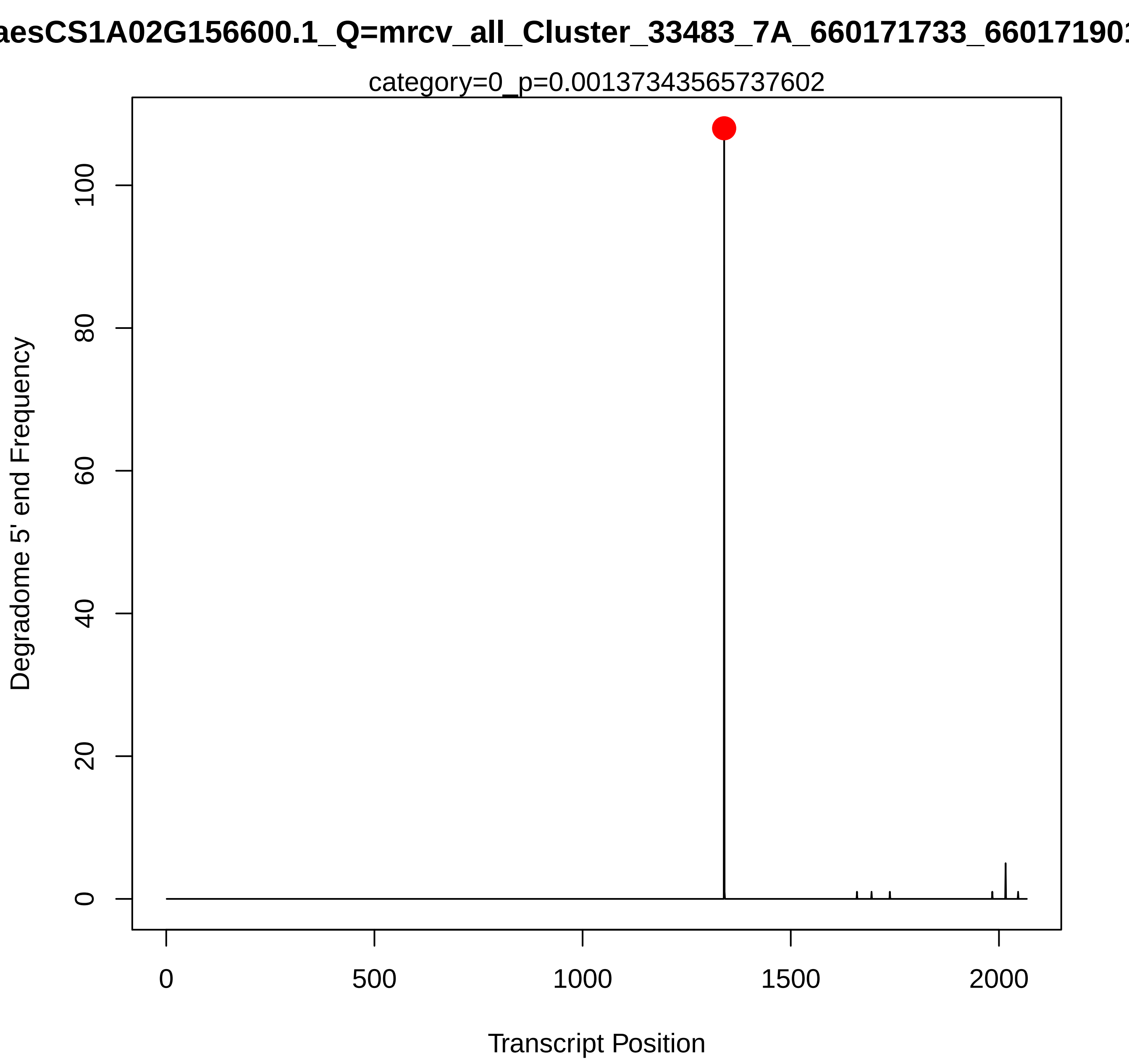
aesCS1A02G156600.1_Q=mrcv_all_Cluster_33483_7A_660171733_66017190
category=0_p=0.00137343565737602
Degradome 5' end Frequency
0
20
40
60
80
100
0
500
1000
1500
2000
Transcript Position

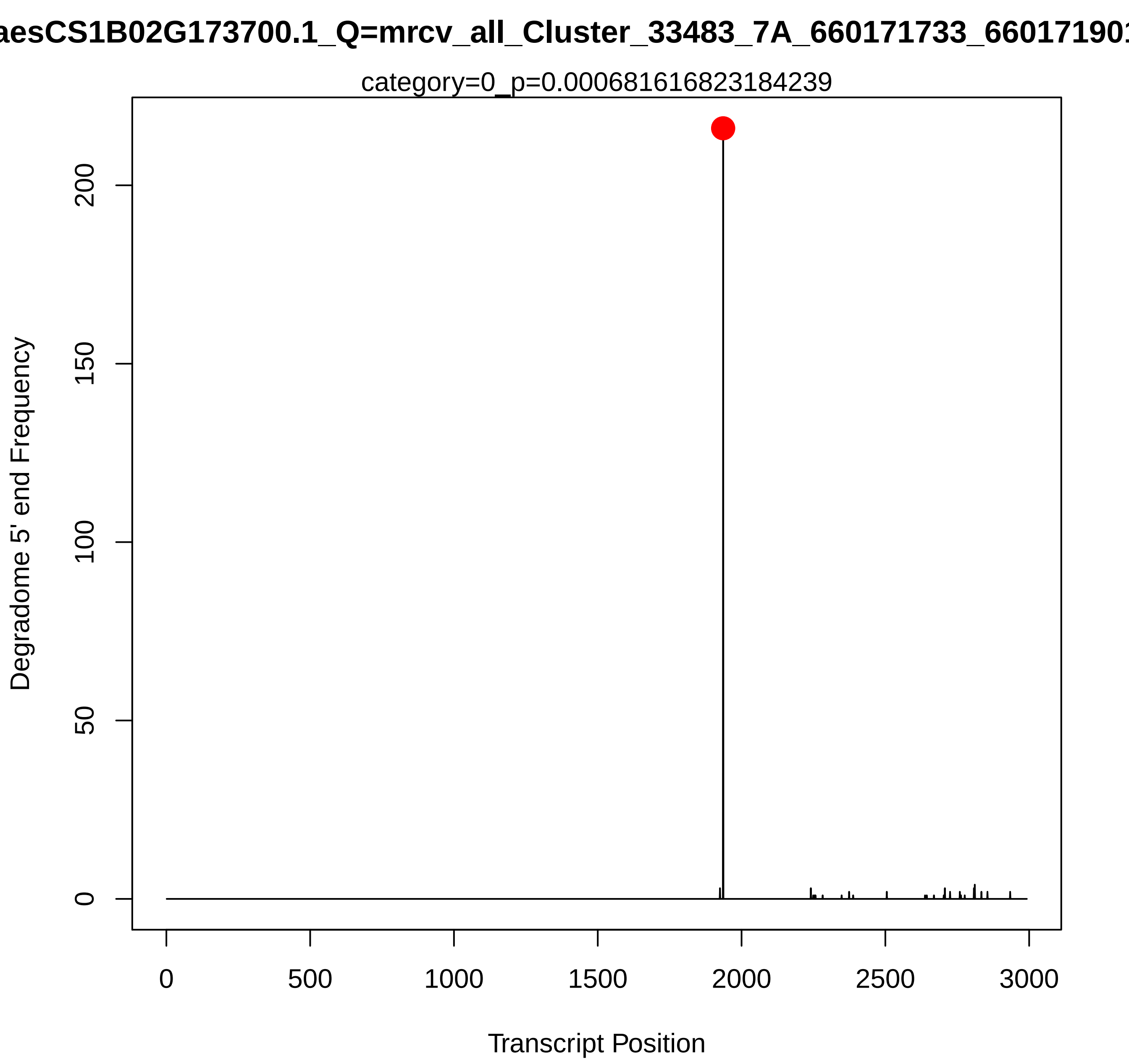
aesCS1B02G173700.1_Q=mrcv_all_Cluster_33483_7A_660171733_66017190
category=0_p=0.000681616823184239
Degradome 5' end Frequency
0
50
100
150
200
0
500
1000
1500
2000
2500
3000
Transcript Position

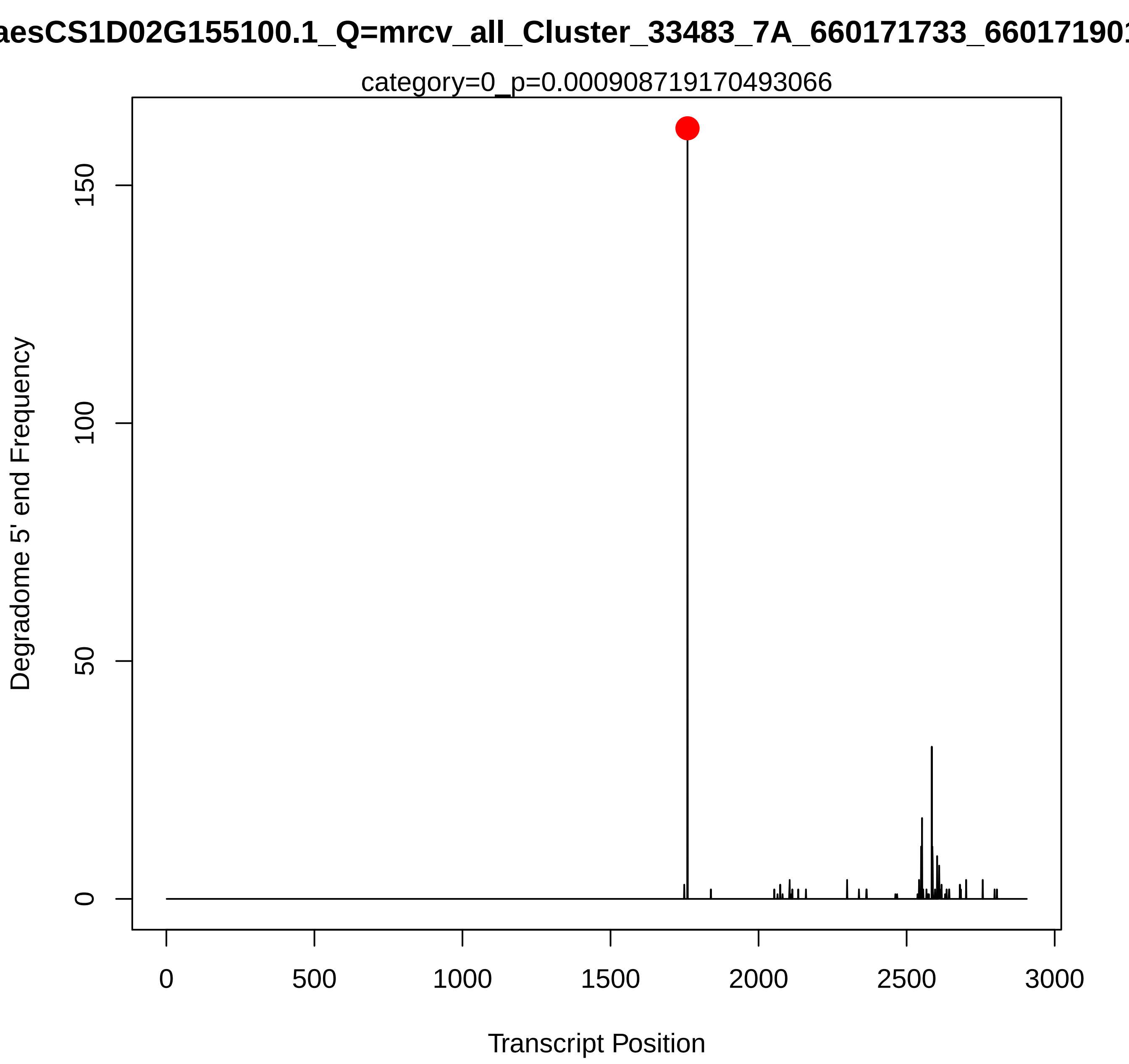
aesCS1D02G155100.1_Q=mrcv_all_Cluster_33483_7A_660171733_66017190
category=0_p=0.000908719170493066
Degradome 5' end Frequency
0
50
100
150
0
500
1000
1500
2000
2500
3000
Transcript Position

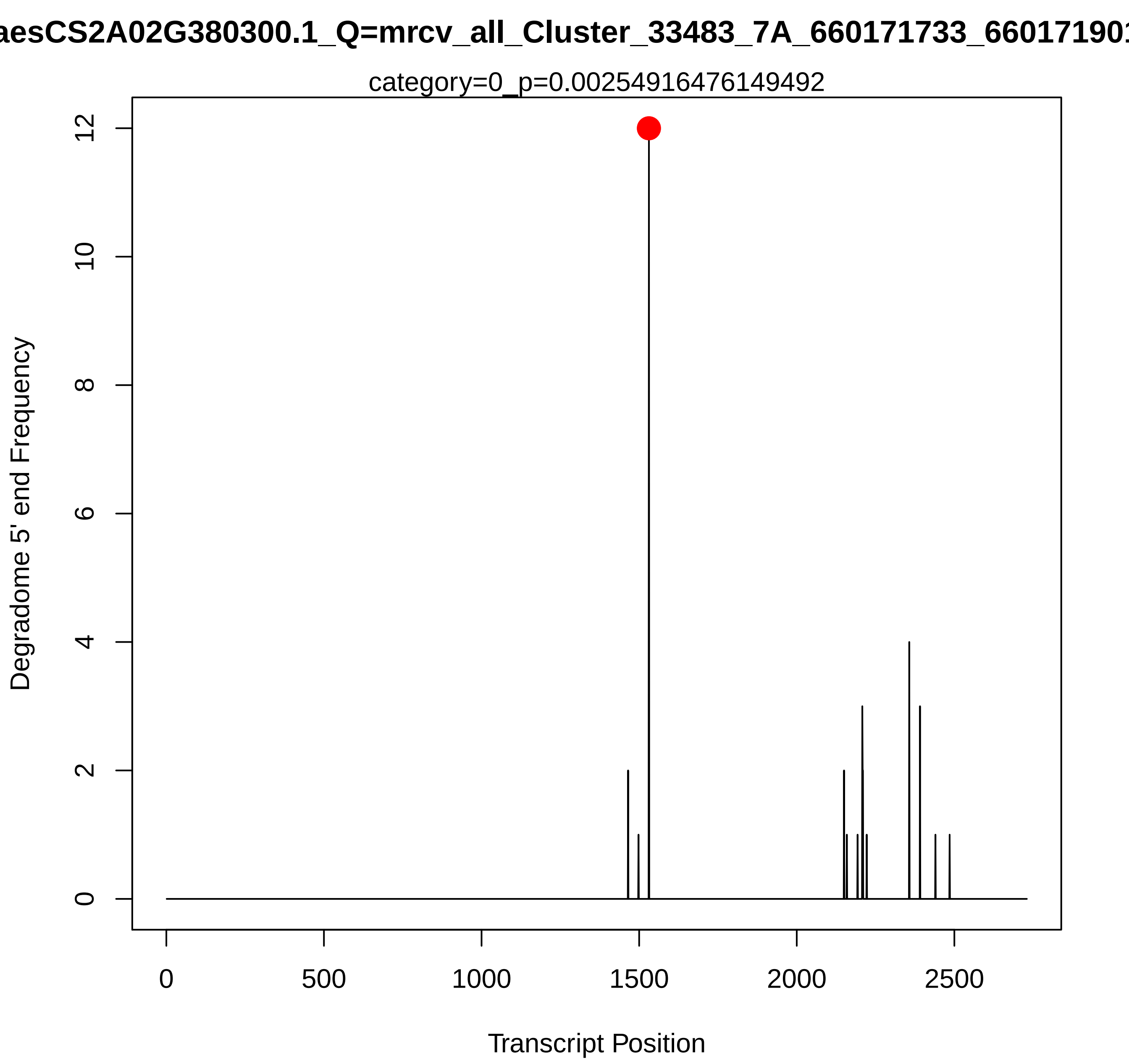
aesCS2A02G380300.1_Q=mrcv_all_Cluster_33483_7A_660171733_66017190
category=0_p=0.00254916476149492
Degradome 5' end Frequency
Transcript Position
0
2
4
6
8
10
12
0
500
1000
1500
2000
2500

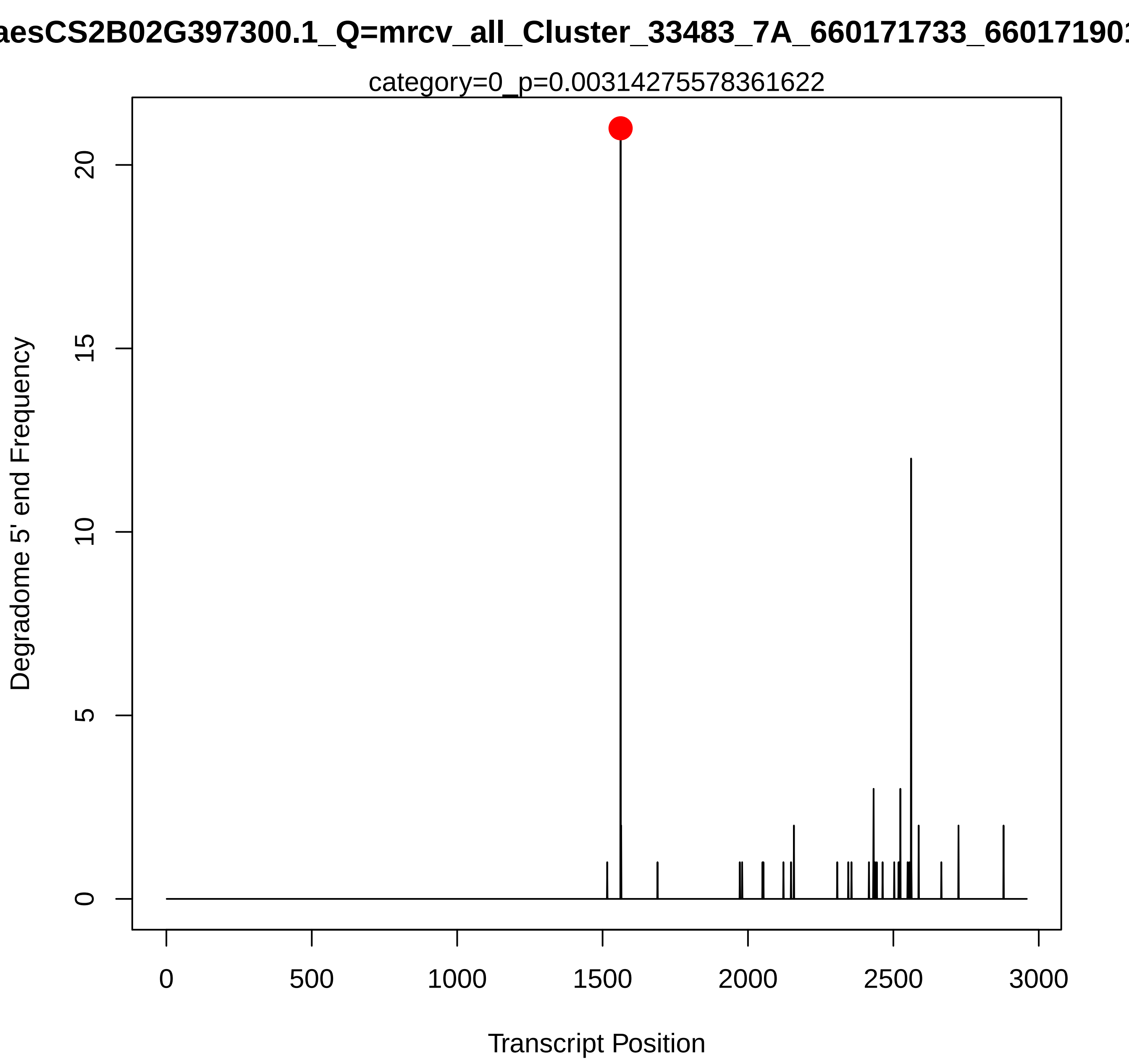
aesCS2B02G397300.1_Q=mrcv_all_Cluster_33483_7A_660171733_66017190
category=0_p=0.00314275578361622
Degradome 5' end Frequency
0
5
10
15
20
0
500
1000
1500
2000
2500
3000
Transcript Position

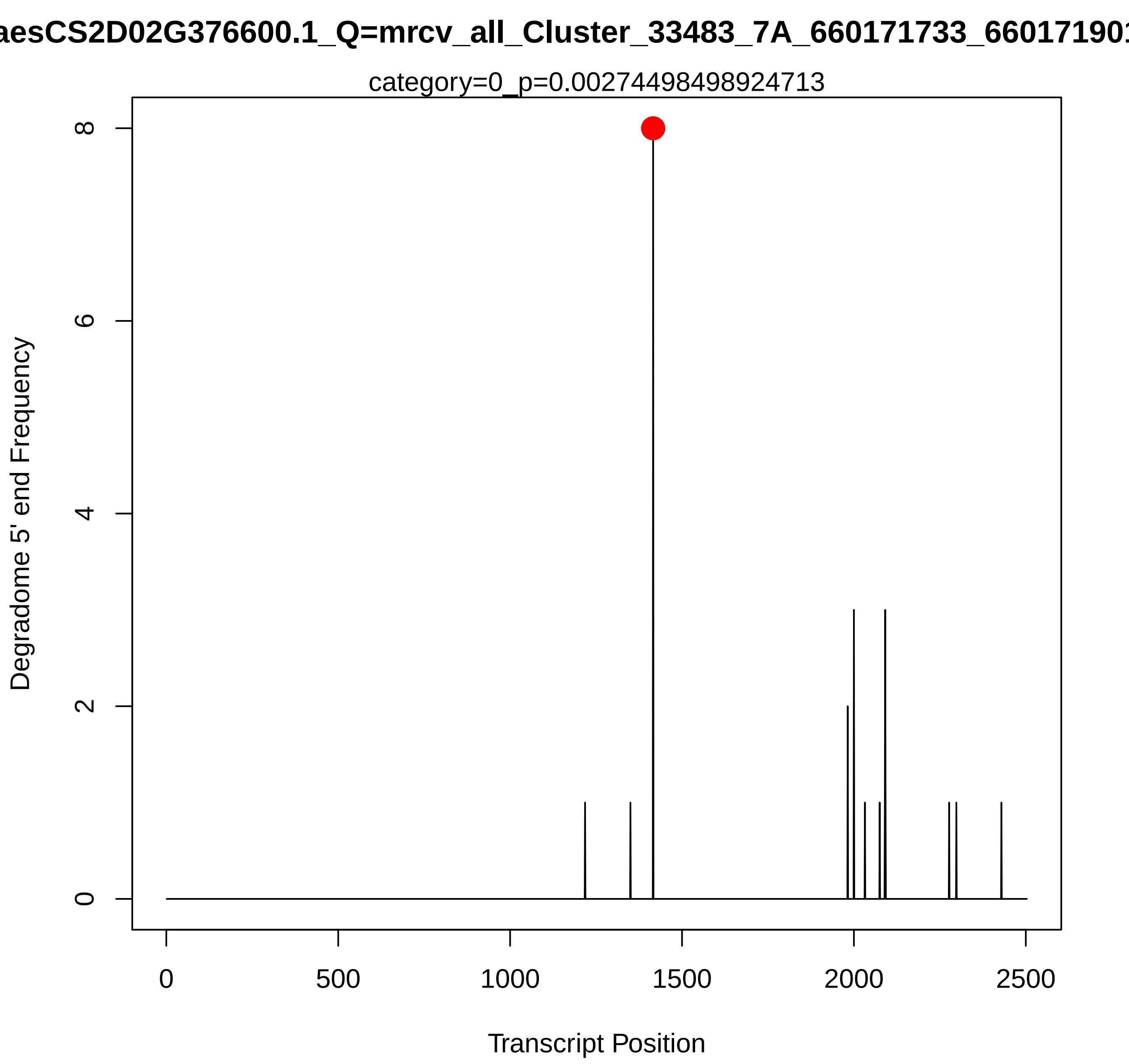

aesCS2D02G376600.1_Q=mrcv_all_Cluster_33483_7A_660171733_66017190
category=0_p=0.00274498498924713
Degradome 5' end Frequency
0
2
4
6
8
0
500
1000
1500
2000
2500
Transcript Position

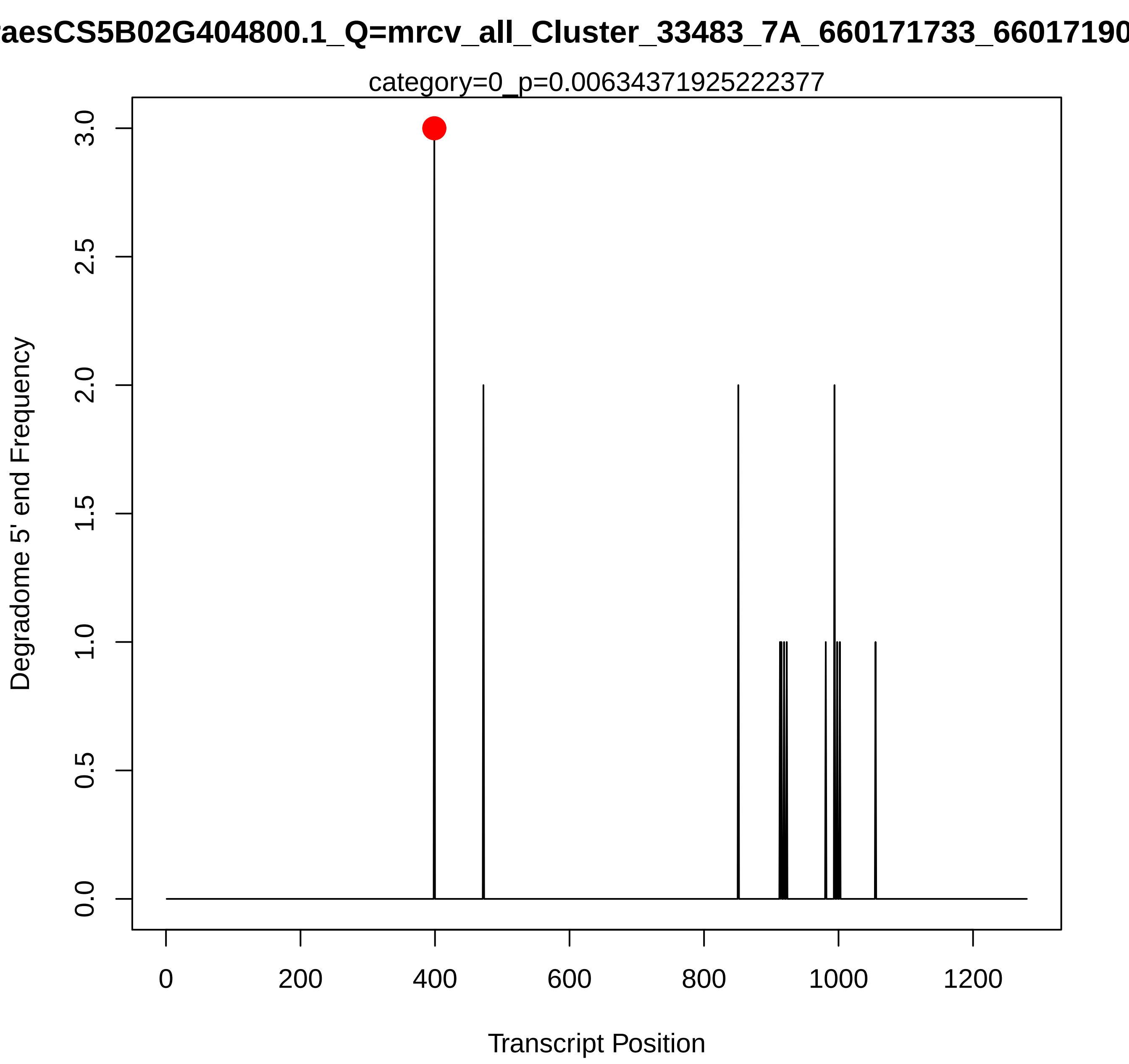

aesCS5B02G404800.1_Q=mrcv_all_Cluster_33483_7A_660171733_66017190
category=0_p=0.00634371925222377
Degradome 5' end Frequency
3.0
2.5
2.0
1.5
1.0
0.5
0.0
0
200
400
600
800
1000
1200
Transcript Position

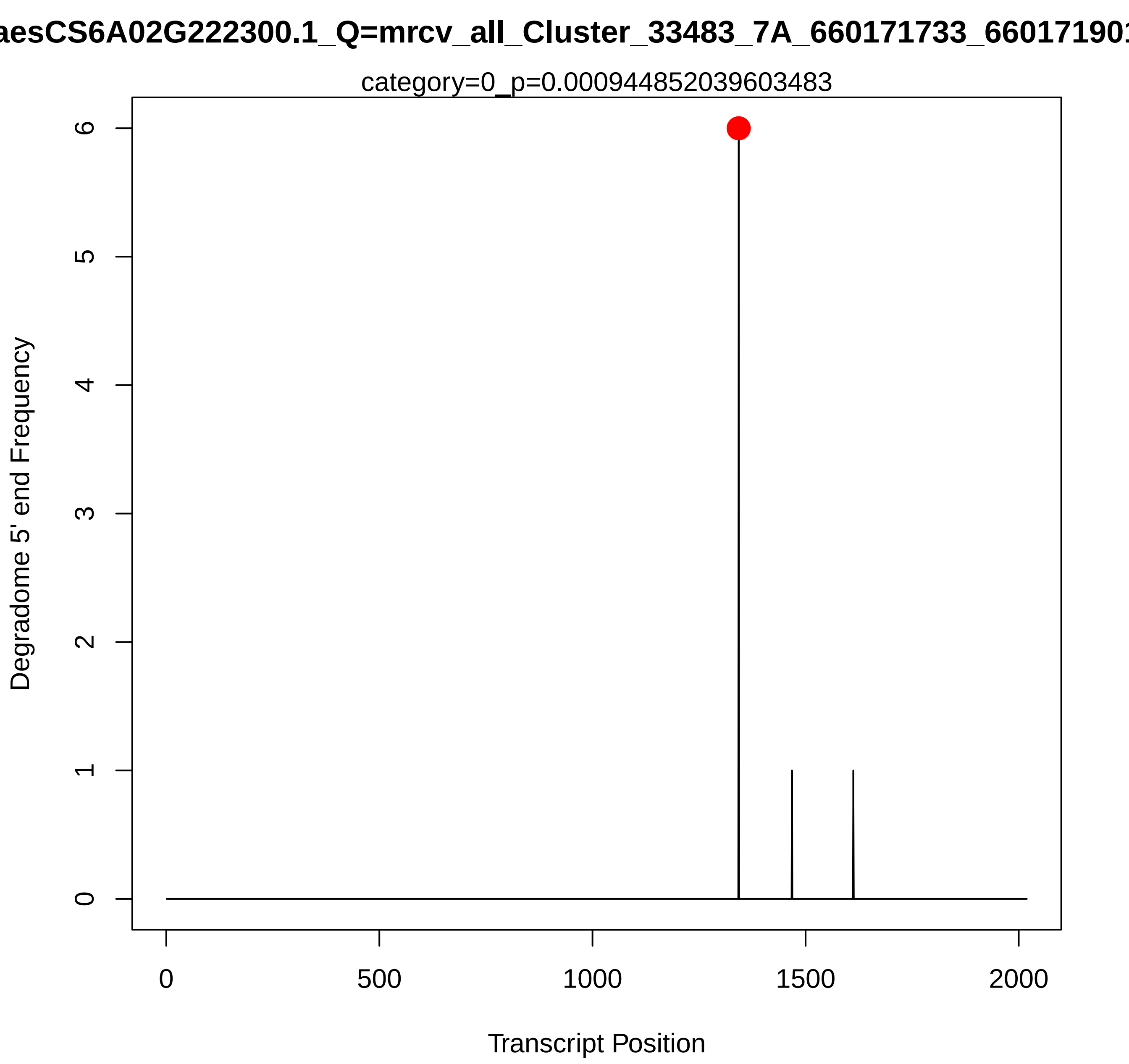
aesCS6A02G222300.1_Q=mrcv_all_Cluster_33483_7A_660171733_66017190
category=0_p=0.000944852039603483
Degradome 5' end Frequency
0
1
2
3
4
5
6
Transcript Position
0
500
1000
1500
2000

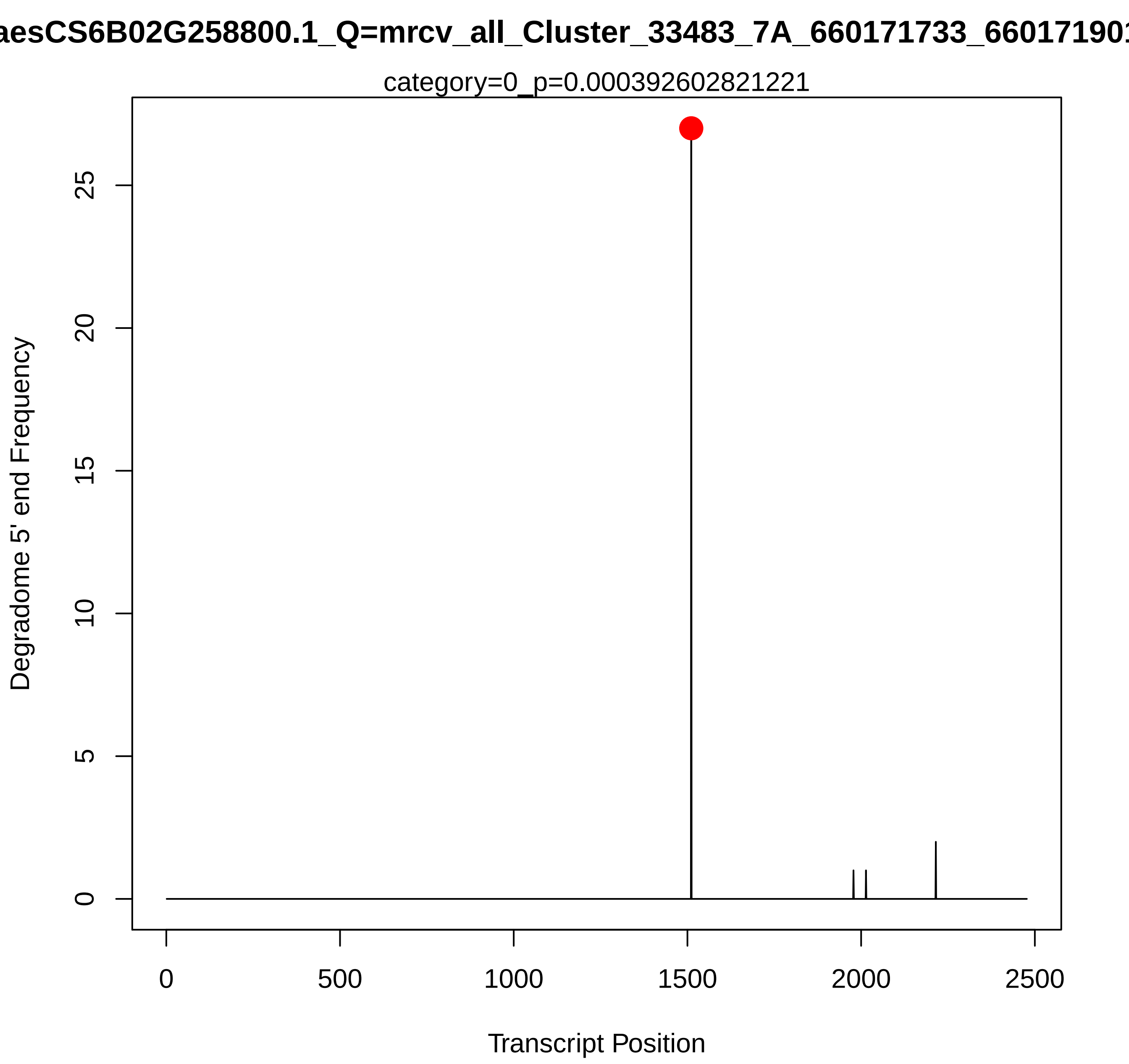
aesCS6B02G258800.1_Q=mrcv_all_Cluster_33483_7A_660171733_66017190
category=0_p=0.000392602821221
Degradome 5' end Frequency
0
5
10
15
20
25
0
500
1000
1500
2000
2500
Transcript Position

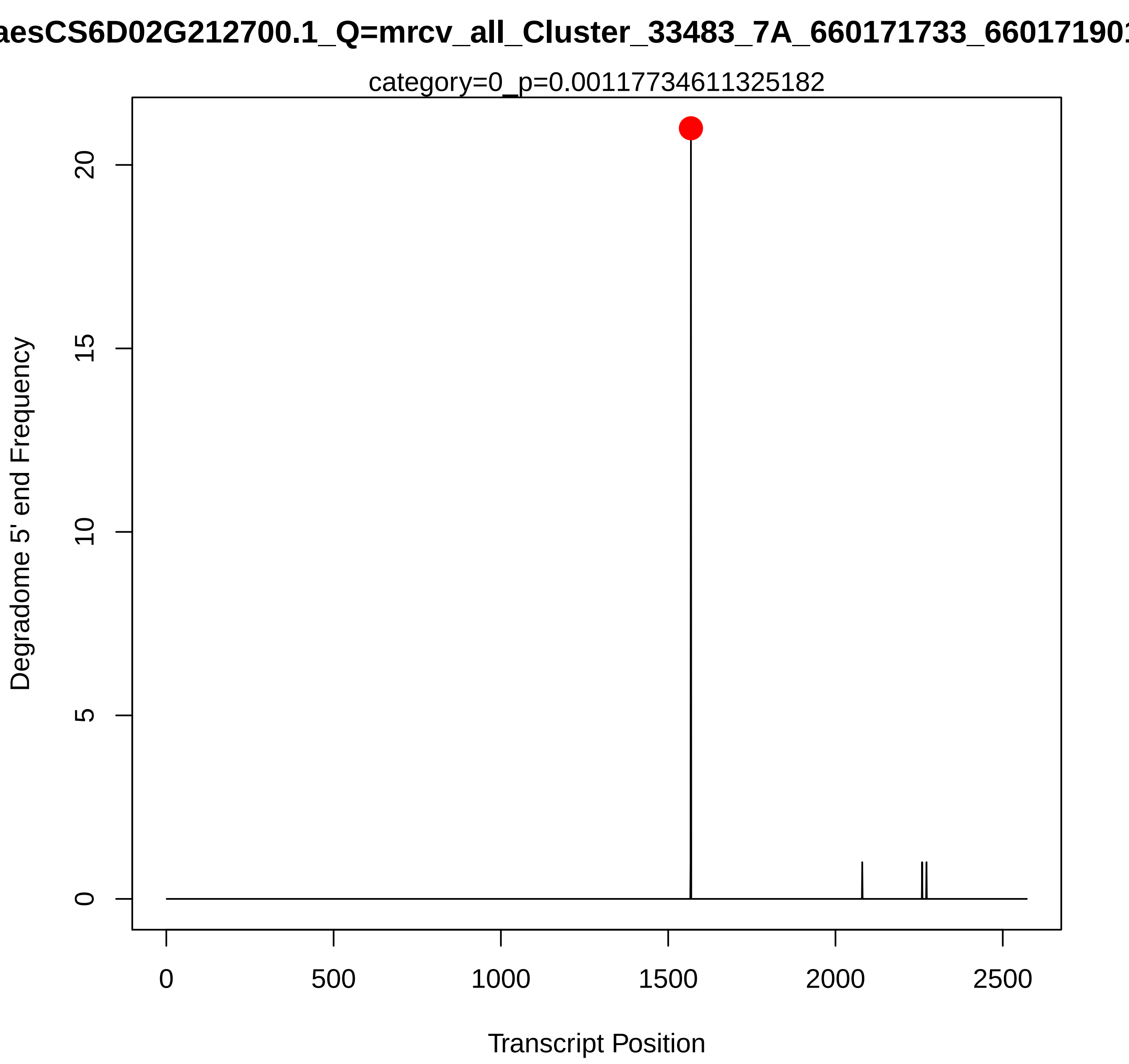

aesCS6D02G212700.1_Q=mrcv_all_Cluster_33483_7A_660171733_66017190
category=0_p=0.00117734611325182
Degradome 5' end Frequency
0
5
10
15
20
0
500
1000
1500
2000
2500
Transcript Position

aesCS7A02G446900.1_Q=mrcv_all_Cluster_33483_7A_660171733_66017190

category=2_p=0.0225798370395063

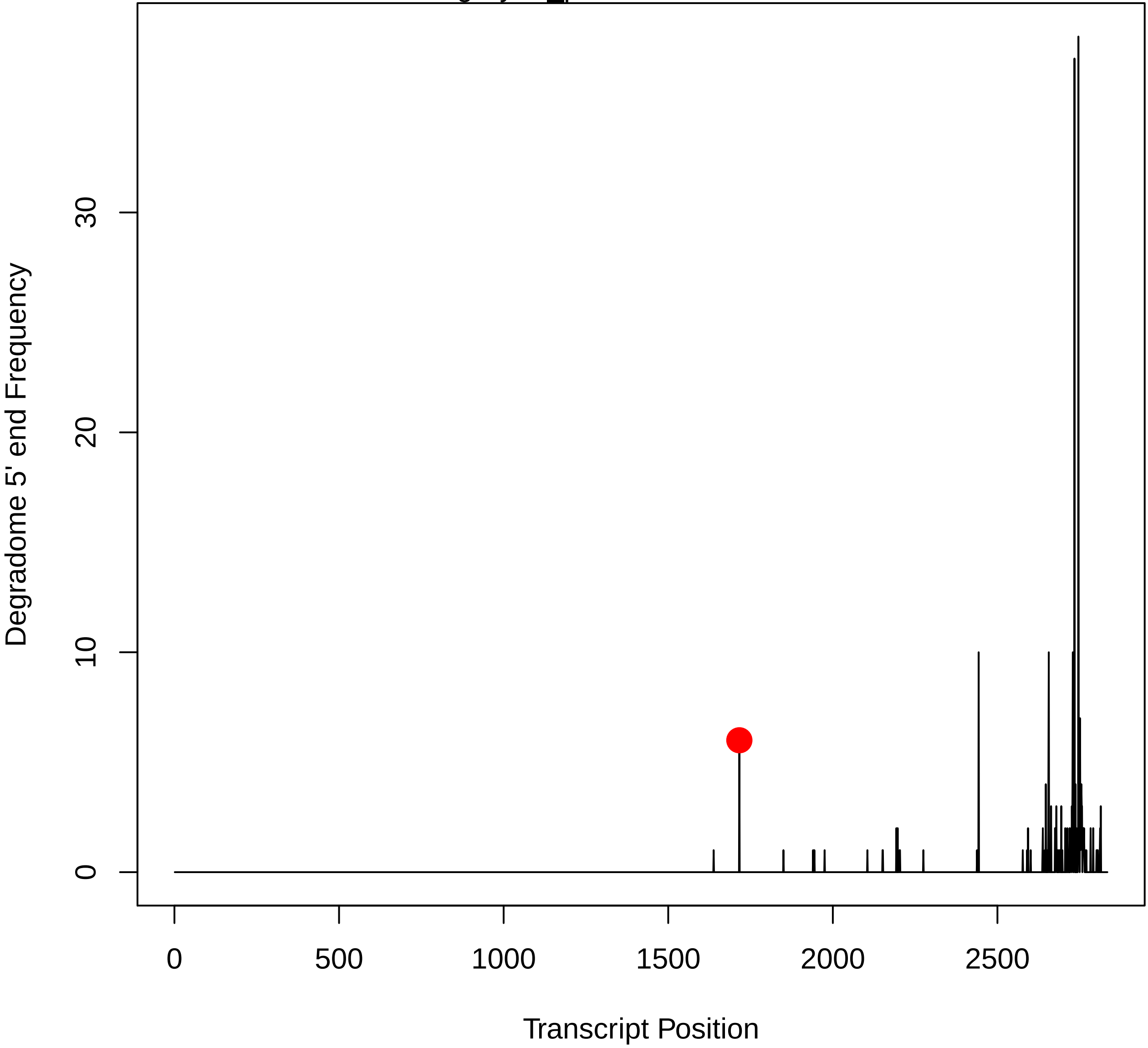

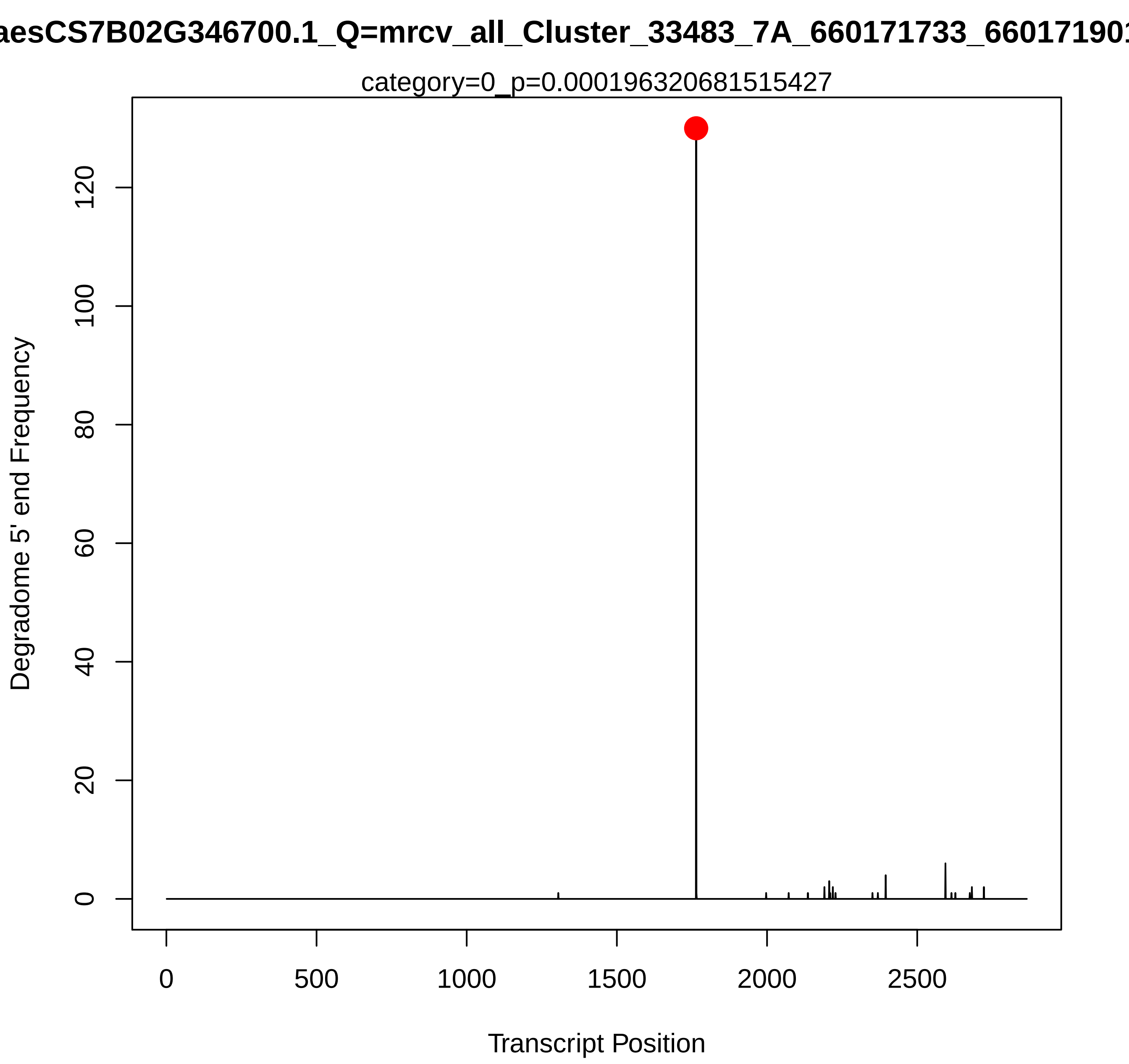
aesCS7B02G346700.1_Q=mrcv_all_Cluster_33483_7A_660171733_66017190
category=0_p=0.000196320681515427
Degradome 5' end Frequency
0
20
40
60
80
100
120
0
500
1000
1500
2000
2500
Transcript Position

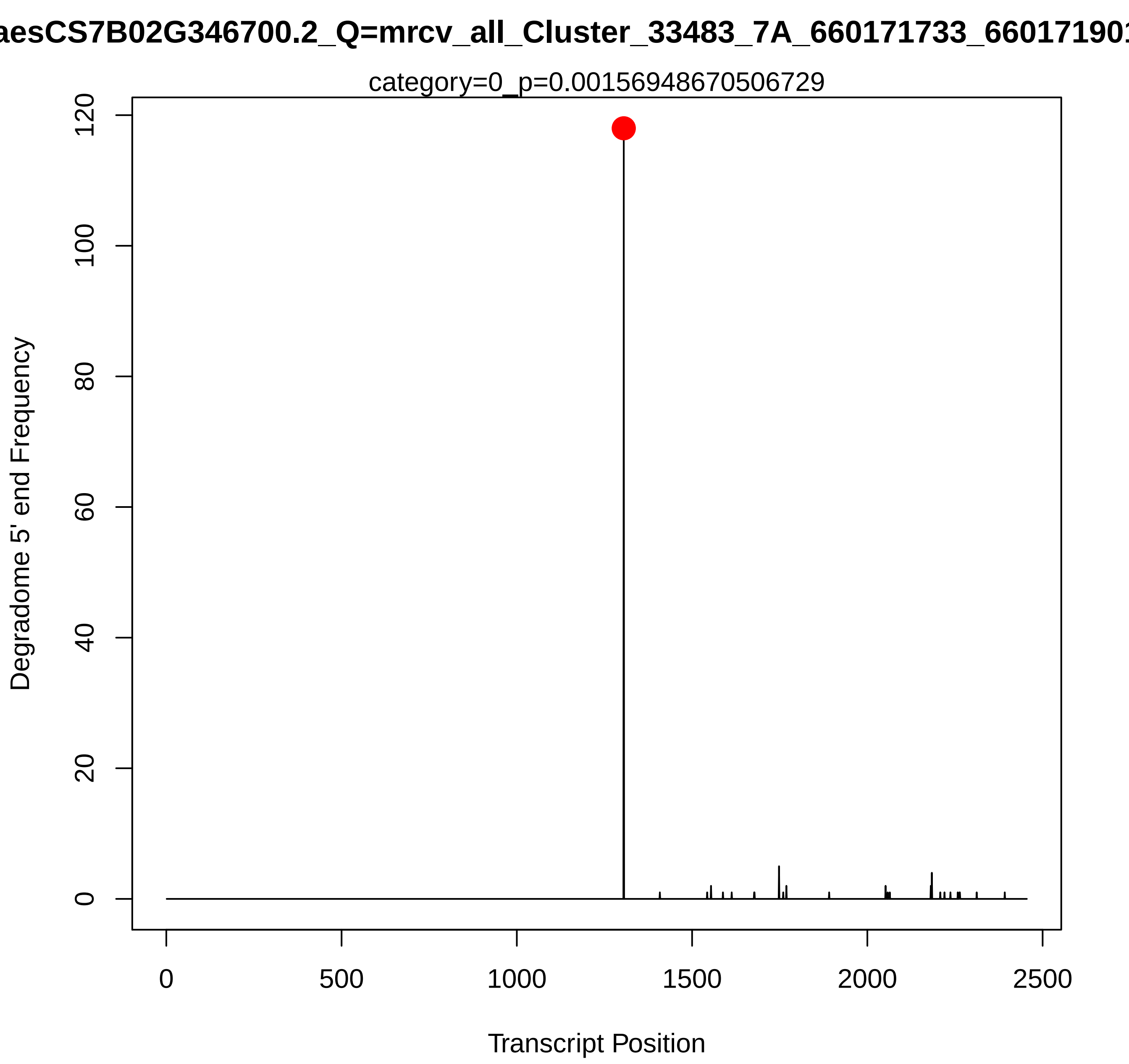
aesCS7B02G346700.2_Q=mrcv_all_Cluster_33483_7A_660171733_66017190
category=0_p=0.00156948670506729
Degradome 5' end Frequency
0
20
40
60
80
100
120
0
500
1000
1500
2000
2500
Transcript Position

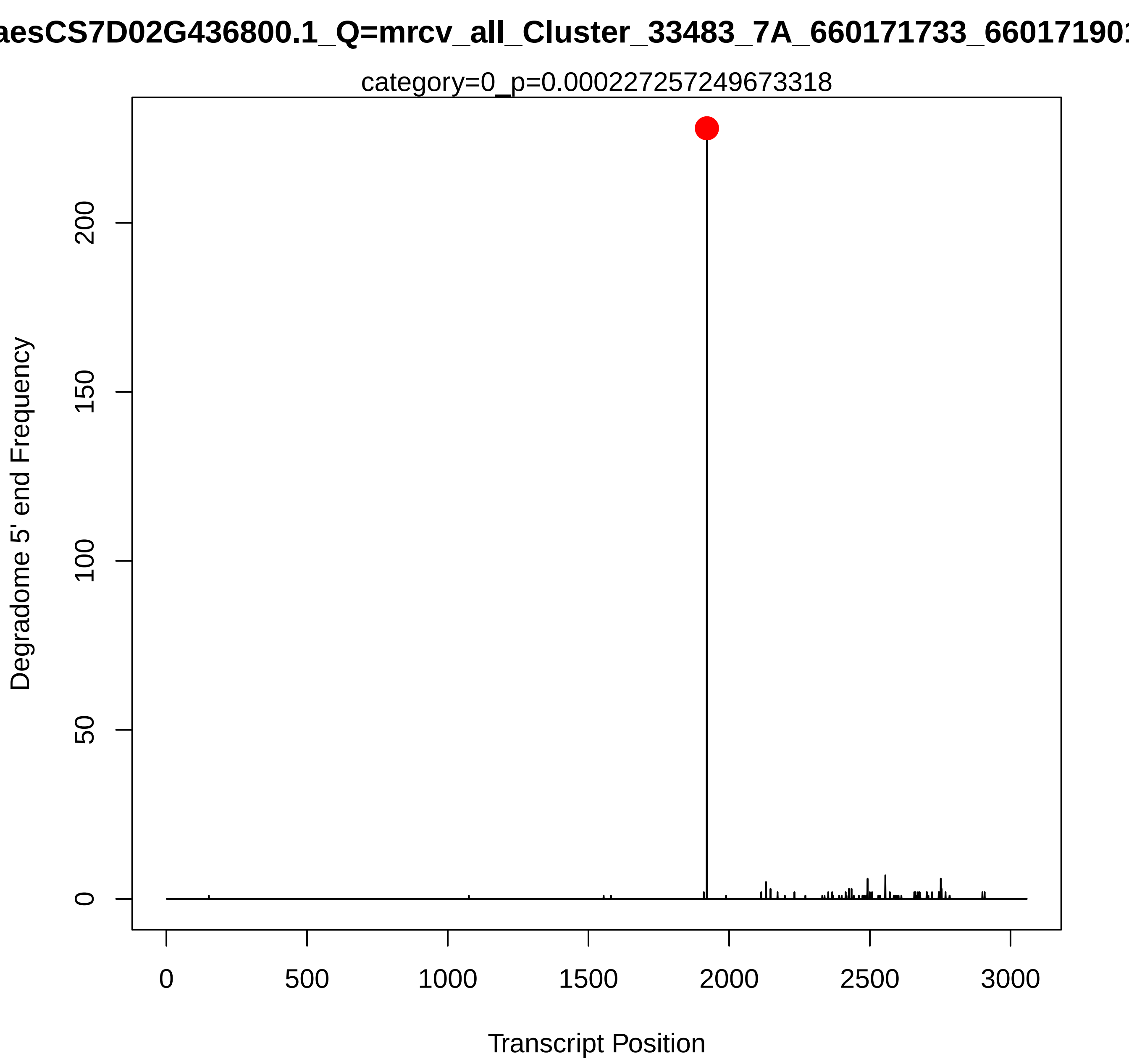
aesCS7D02G436800.1_Q=mrcv_all_Cluster_33483_7A_660171733_66017190
category=0_p=0.000227257249673318
Degradome 5' end Frequency
0
50
100
150
200
0
500
1000
1500
2000
2500
3000
Transcript Position

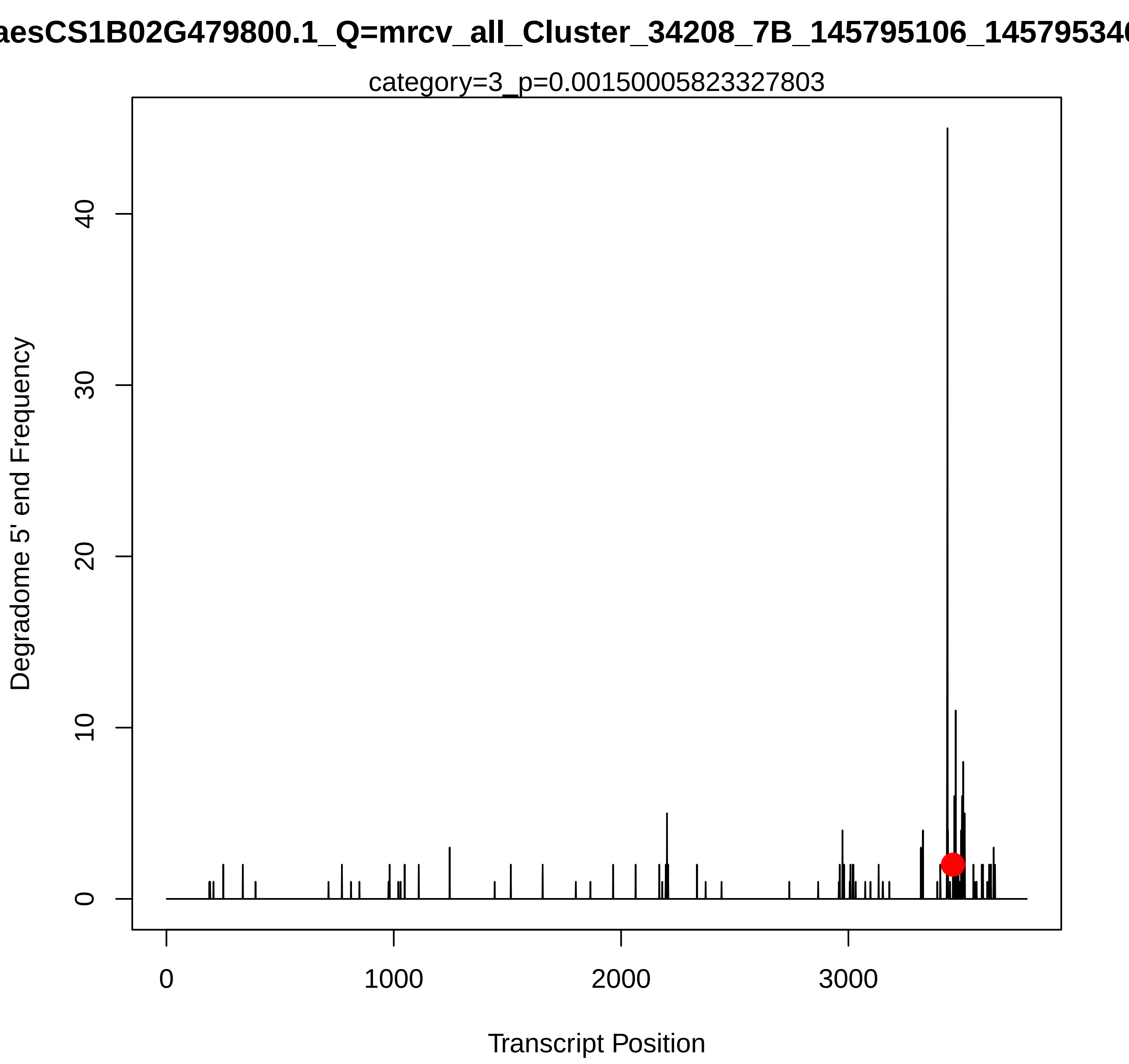
aesCS1B02G479800.1_Q=mrcv_all_Cluster_34208_7B_145795106_14579534
category=3_p=0.00150005823327803
Degradome 5' end Frequency
0
10
20
30
40
0
1000
2000
3000
Transcript Position

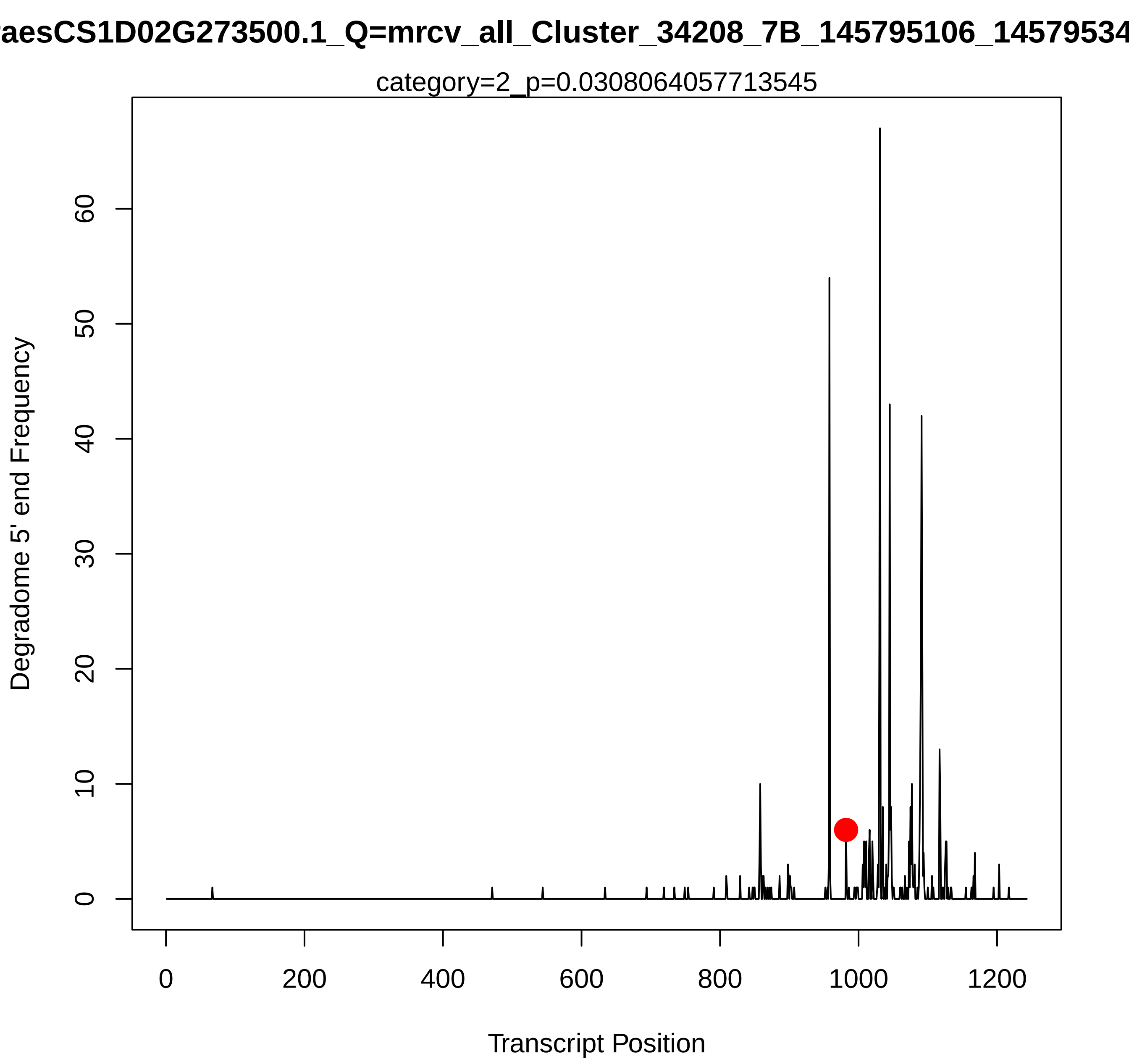

aesCS1D02G273500.1_Q=mrcv_all_Cluster_34208_7B_145795106_14579534
category=2_p=0.0308064057713545
Degradome 5' end Frequency
0
10
20
30
40
50
60
0
200
400
600
800
1000
1200
Transcript Position

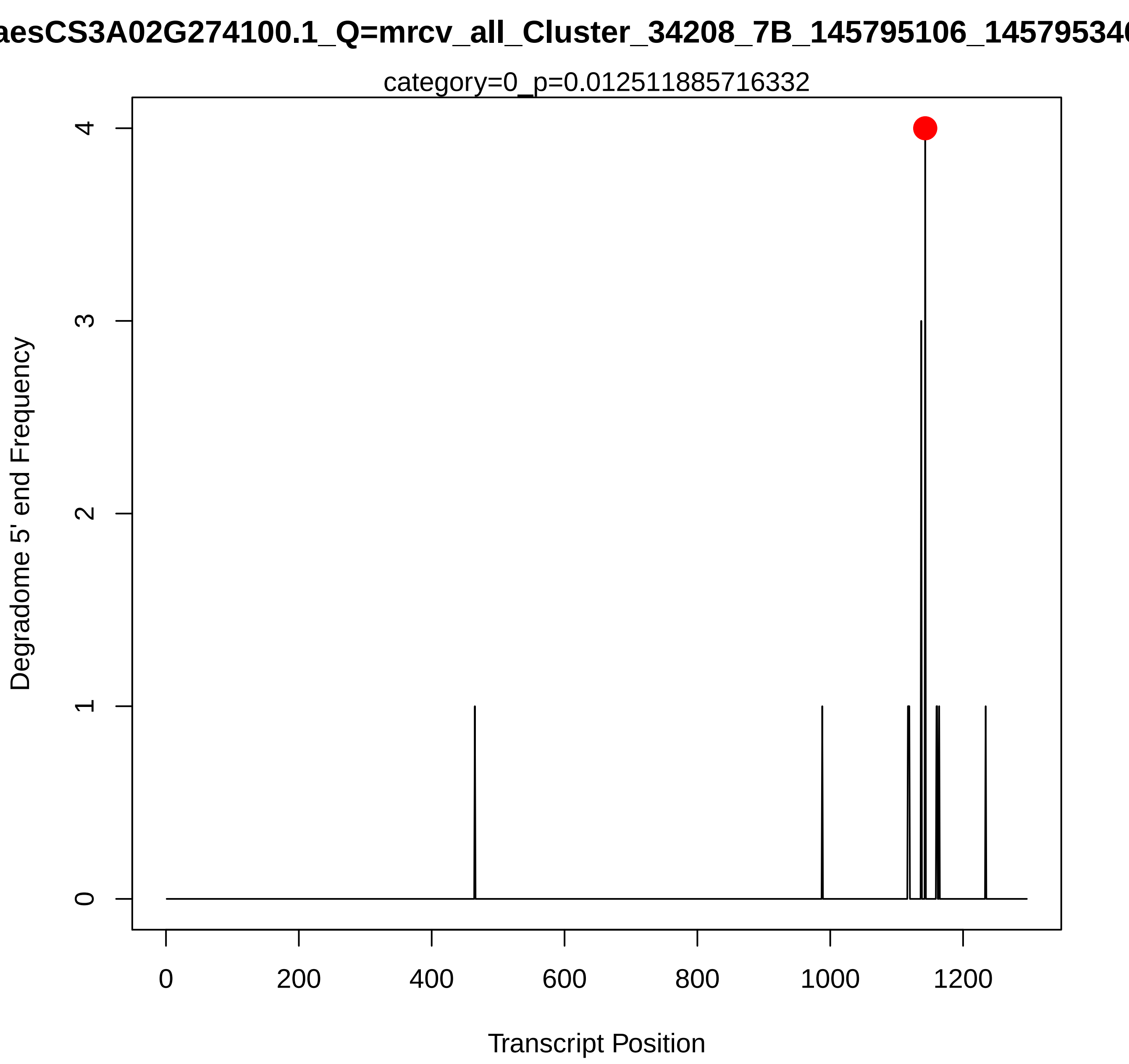

aesCS3A02G274100.1_Q=mrcv_all_Cluster_34208_7B_145795106_14579534
category=0_p=0.012511885716332
Degradome 5' end Frequency
0
1
2
3
4
0
200
400
600
800
1000
1200
Transcript Position

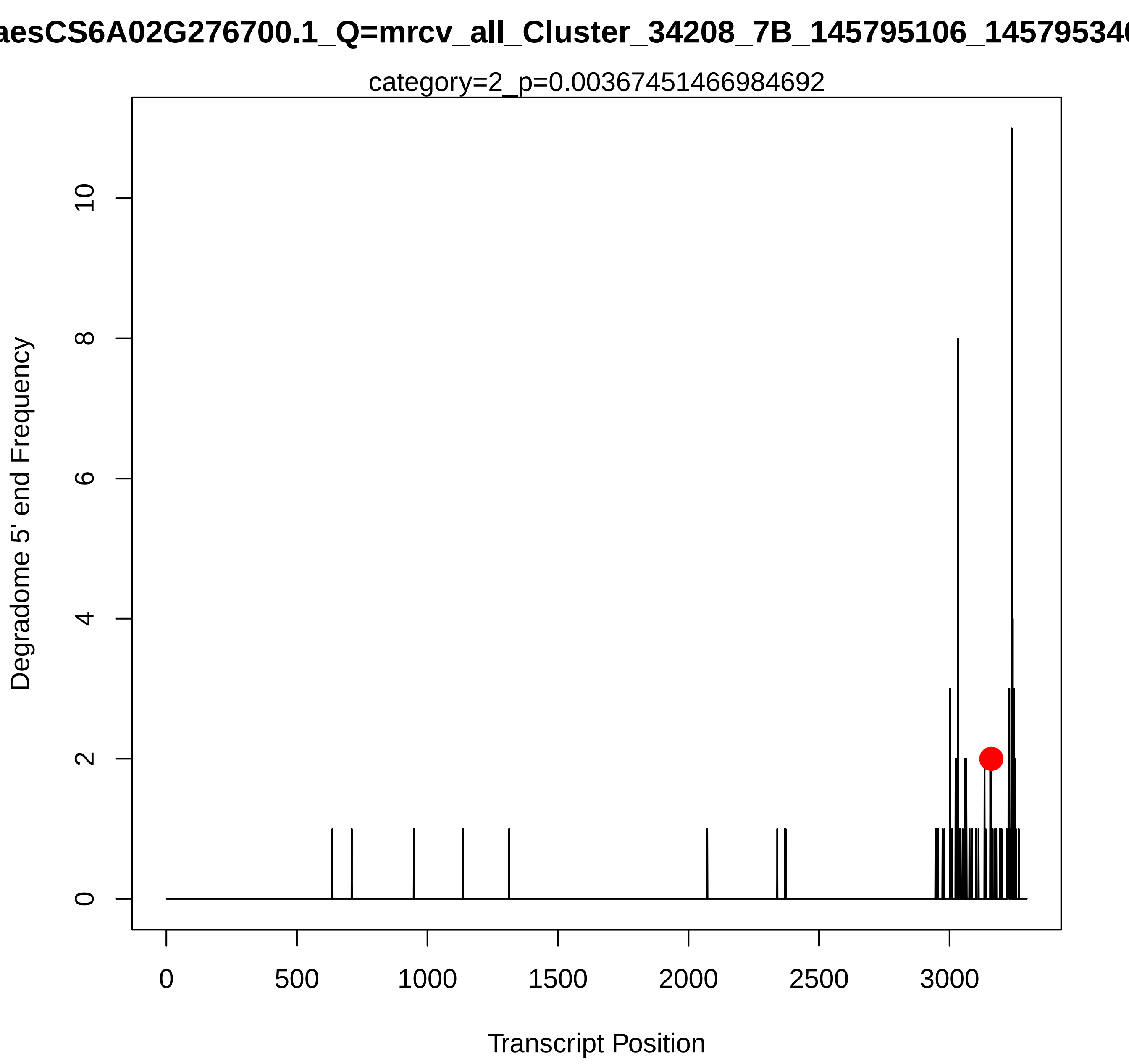
aesCS6A02G276700.1_Q=mrcv_all_Cluster_34208_7B_145795106_14579534
category=2_p=0.00367451466984692
Degradome 5' end Frequency
0
2
4
6
8
10
0
500
1000
1500
2000
2500
3000
Transcript Position

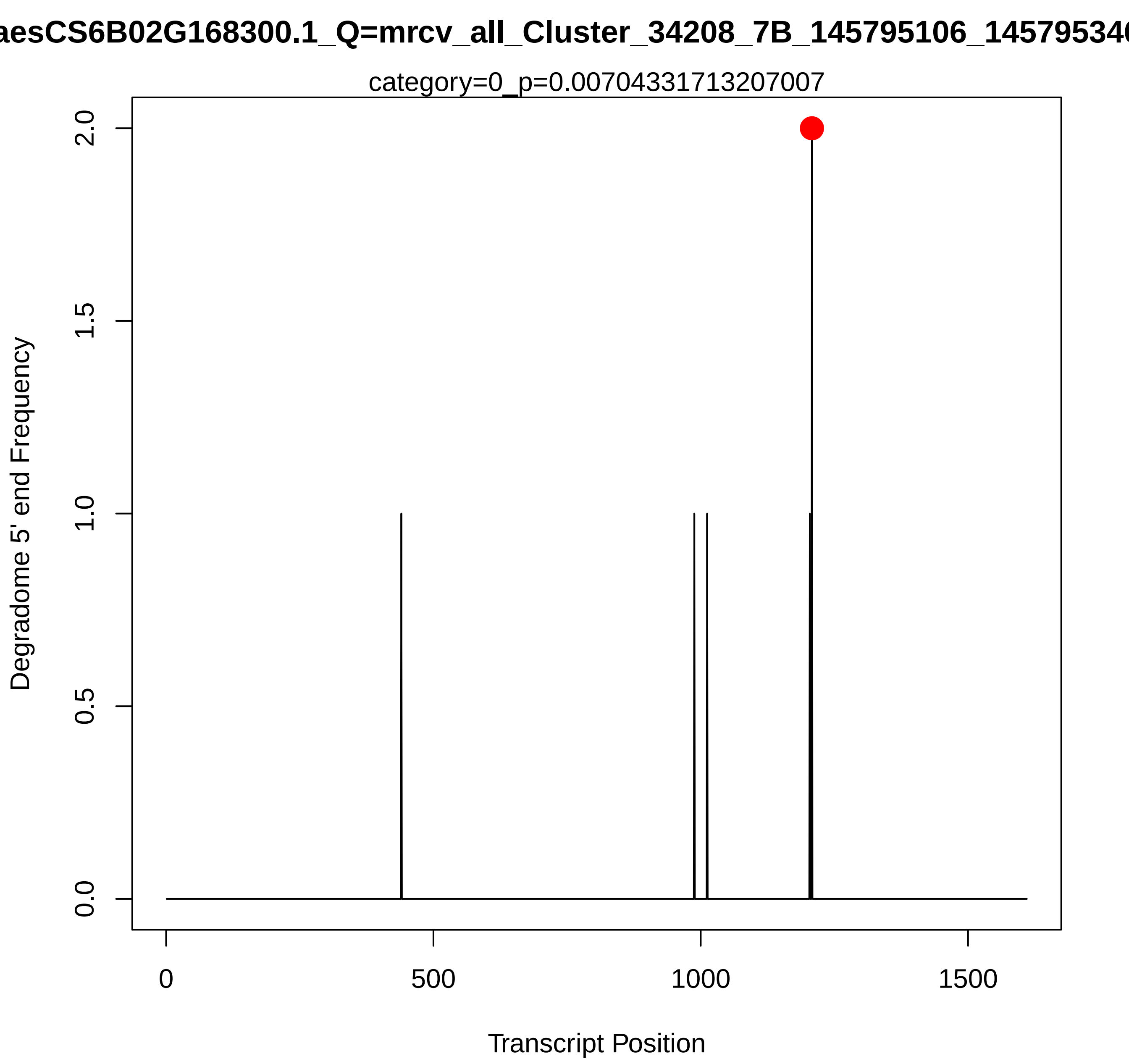

aesCS6B02G168300.1_Q=mrcv_all_Cluster_34208_7B_145795106_14579534
category=0_p=0.00704331713207007
Degradome 5' end Frequency
0.0
0.5
1.0
1.5
2.0
0
500
1000
1500
Transcript Position

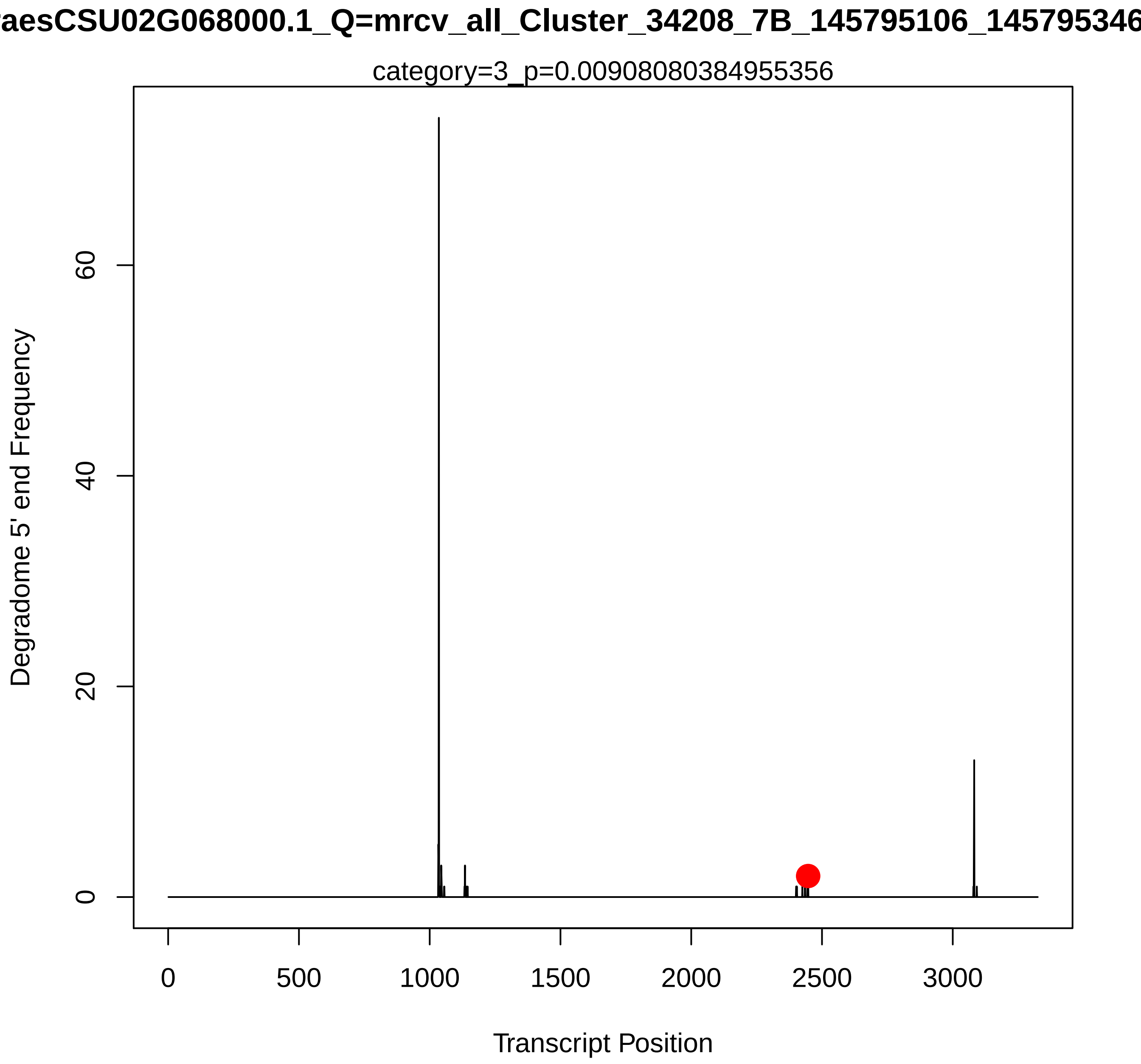

aesCSU02G068000.1_Q=mrcv_all_Cluster_34208_7B_145795106_14579534
category=3_p=0.00908080384955356
Degradome 5' end Frequency
0
20
40
60
0
500
1000
1500
2000
2500
3000
Transcript Position

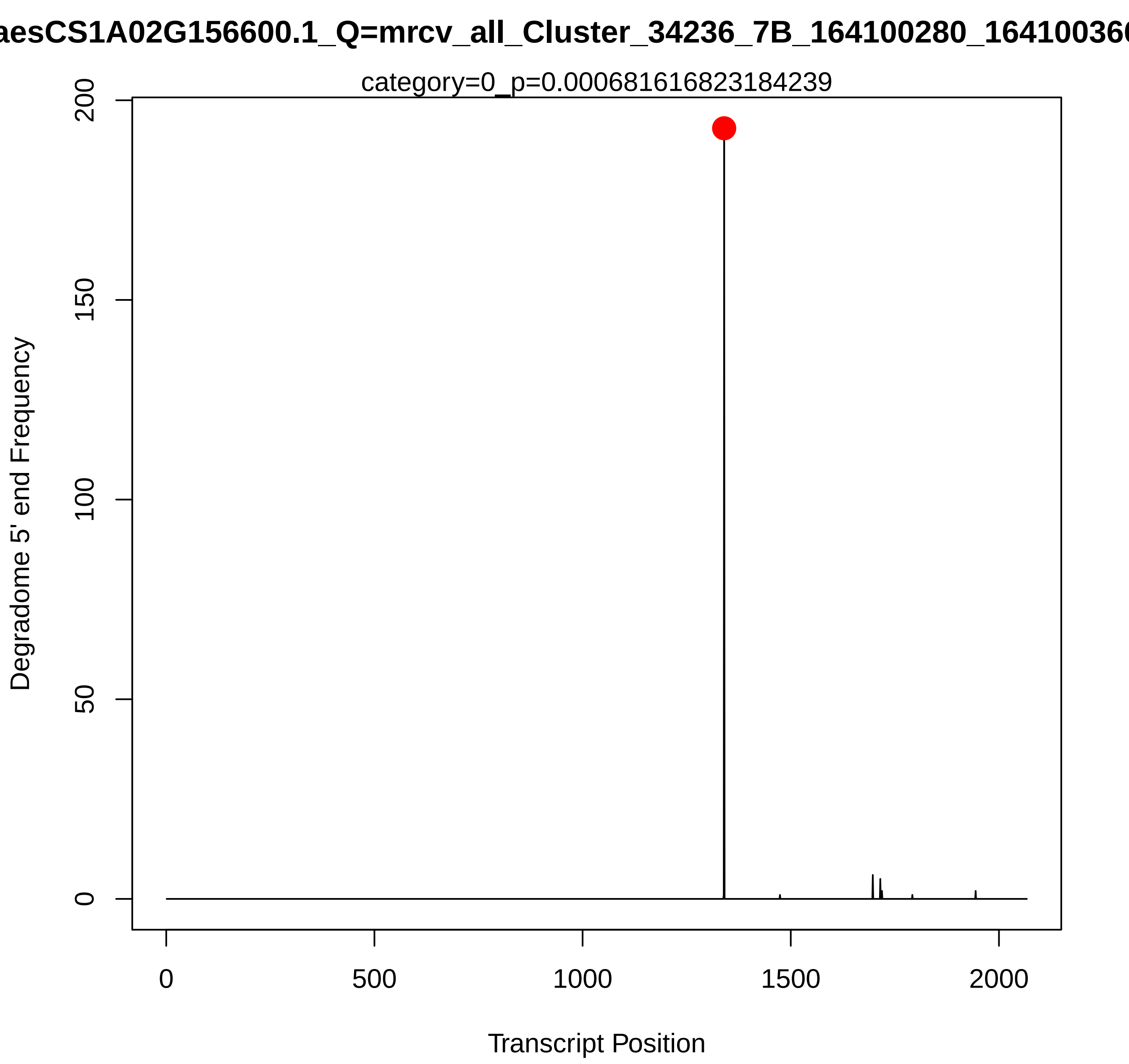
aesCS1A02G156600.1_Q=mrcv_all_Cluster_34236_7B_164100280_16410036
category=0_p=0.000681616823184239
Degradome 5' end Frequency
0
50
100
150
200
0
500
1000
1500
2000
Transcript Position

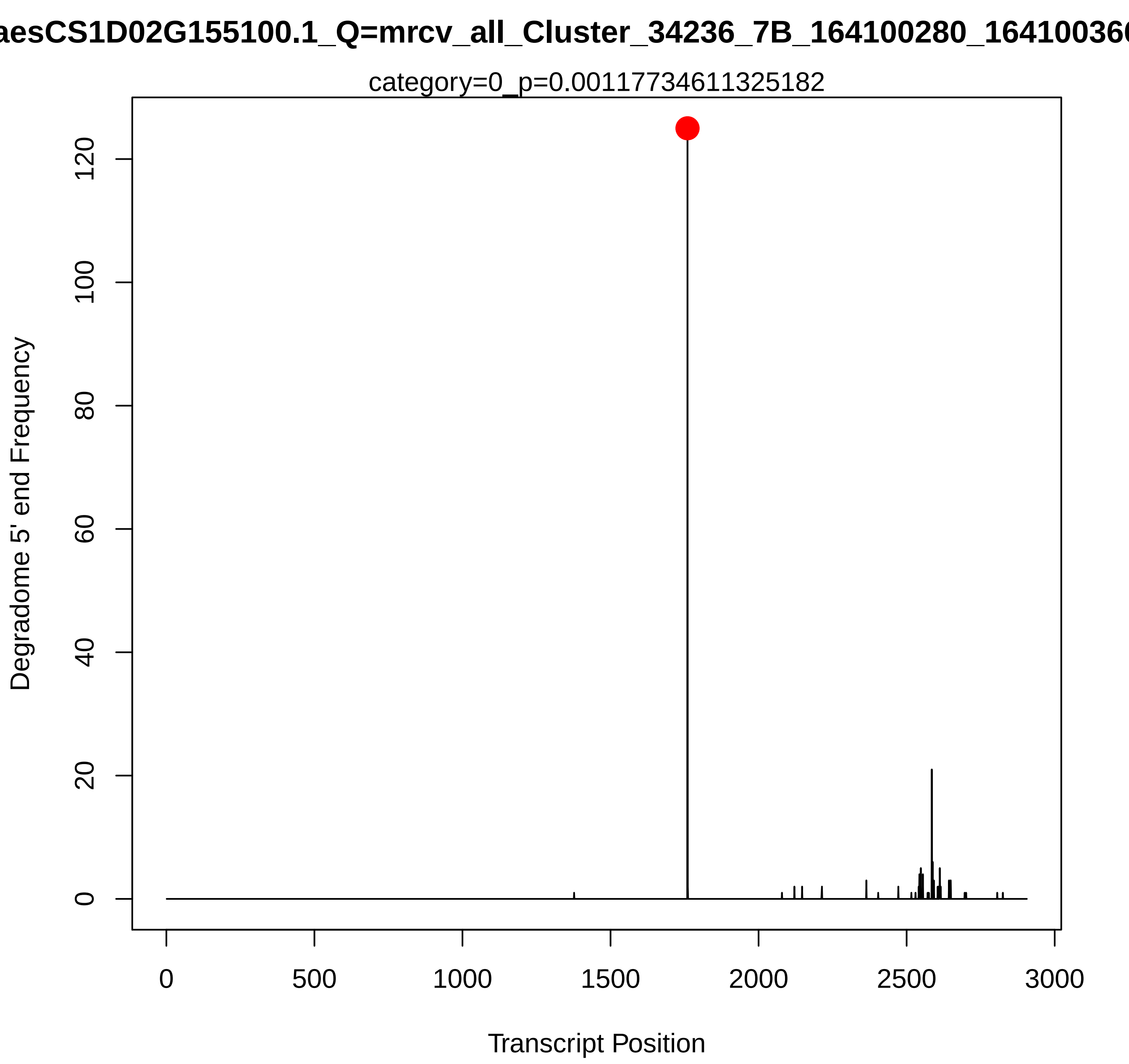
aesCS1D02G155100.1_Q=mrcv_all_Cluster_34236_7B_164100280_16410036
category=0_p=0.00117734611325182
Degradome 5' end Frequency
0
20
40
60
80
100
120
0
500
1000
1500
2000
2500
3000
Transcript Position

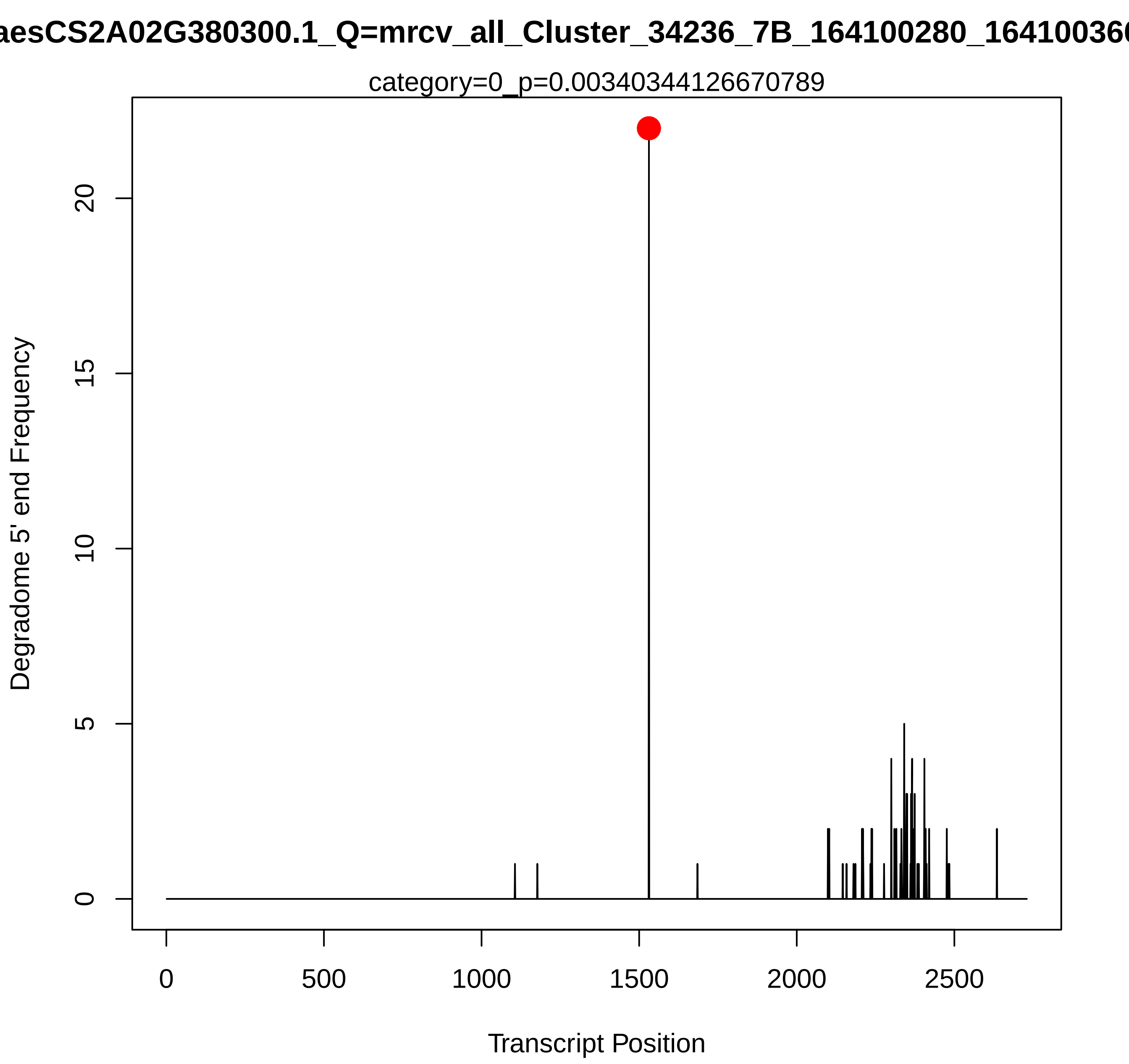

aesCS2A02G380300.1_Q=mrcv_all_Cluster_34236_7B_164100280_16410036
category=0_p=0.00340344126670789
Degradome 5' end Frequency
0
5
10
15
20
0
500
1000
1500
2000
2500
Transcript Position

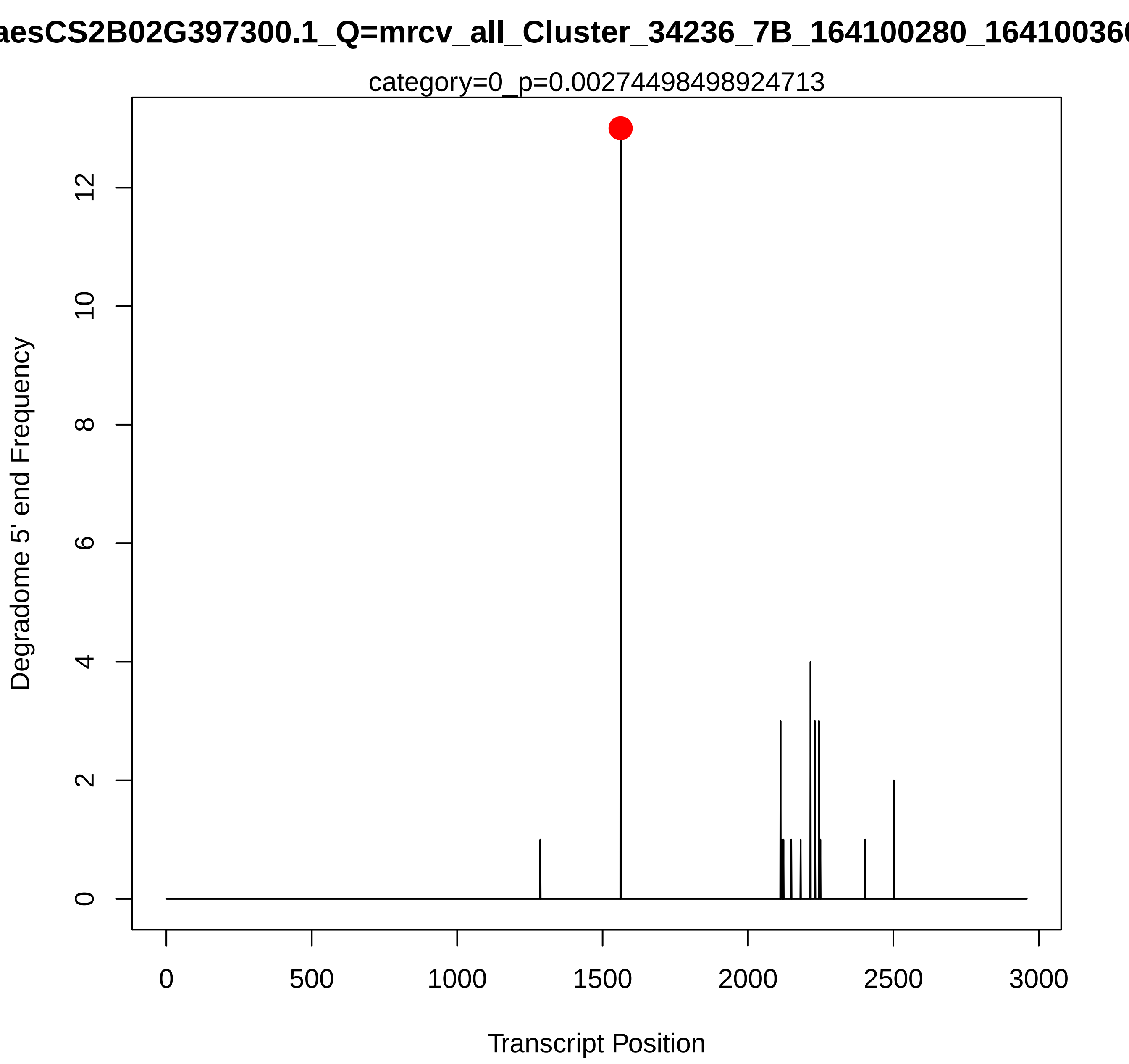

aesCS2B02G397300.1_Q=mrcv_all_Cluster_34236_7B_164100280_16410036
category=0_p=0.00274498498924713
Degradome 5' end Frequency
0
2
4
6
8
10
12
Transcript Position
0
500
1000
1500
2000
2500
3000

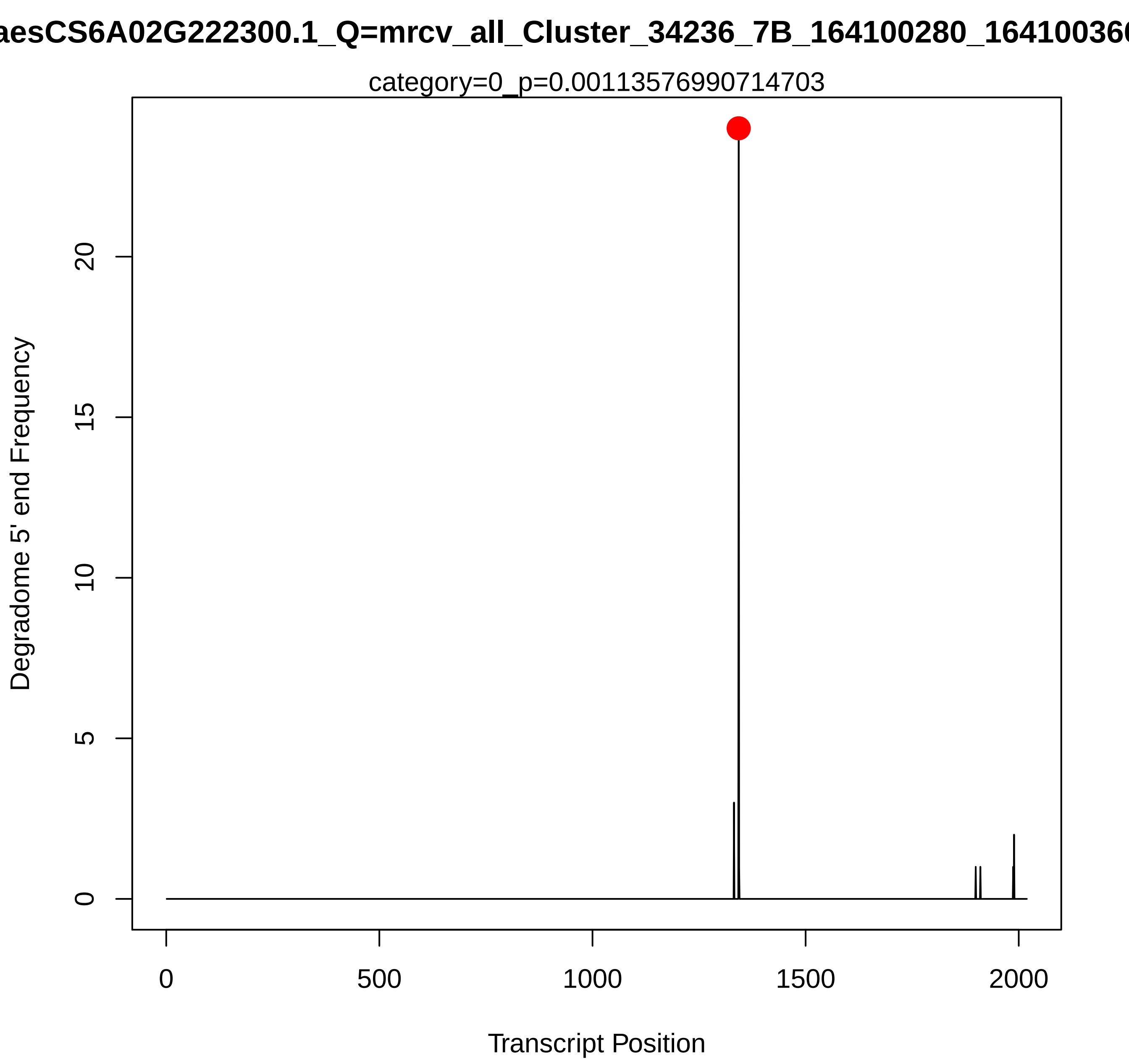

aesCS6A02G222300.1_Q=mrcv_all_Cluster_34236_7B_164100280_16410036
category=0_p=0.00113576990714703
Degradome 5' end Frequency
0
5
10
15
20
0
500
1000
1500
2000
Transcript Position

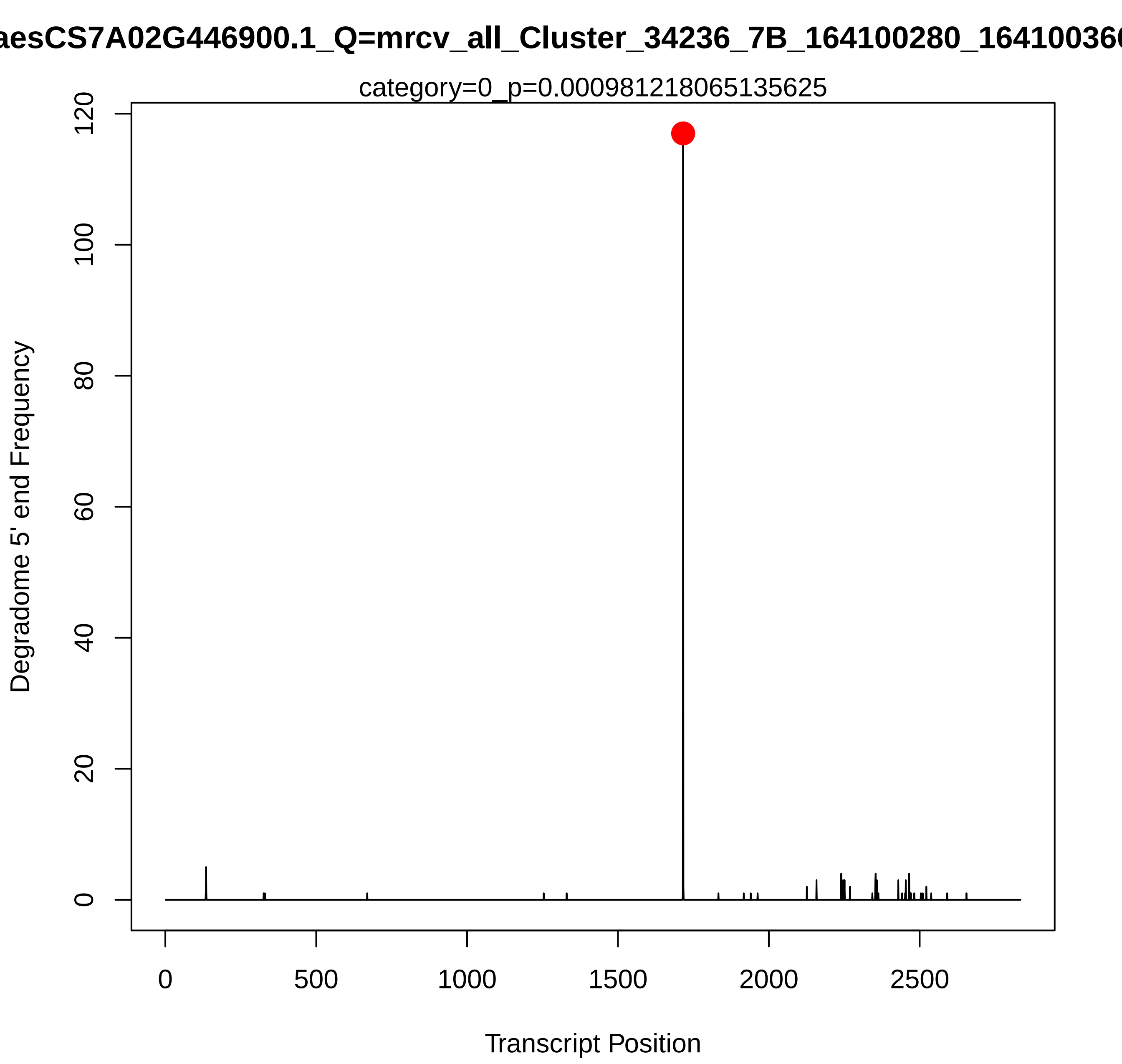
aesCS7A02G446900.1_Q=mrcv_all_Cluster_34236_7B_164100280_16410036
category=0_p=0.000981218065135625
Degradome 5' end Frequency
0
20
40
60
80
100
120
0
500
1000
1500
2000
2500
Transcript Position

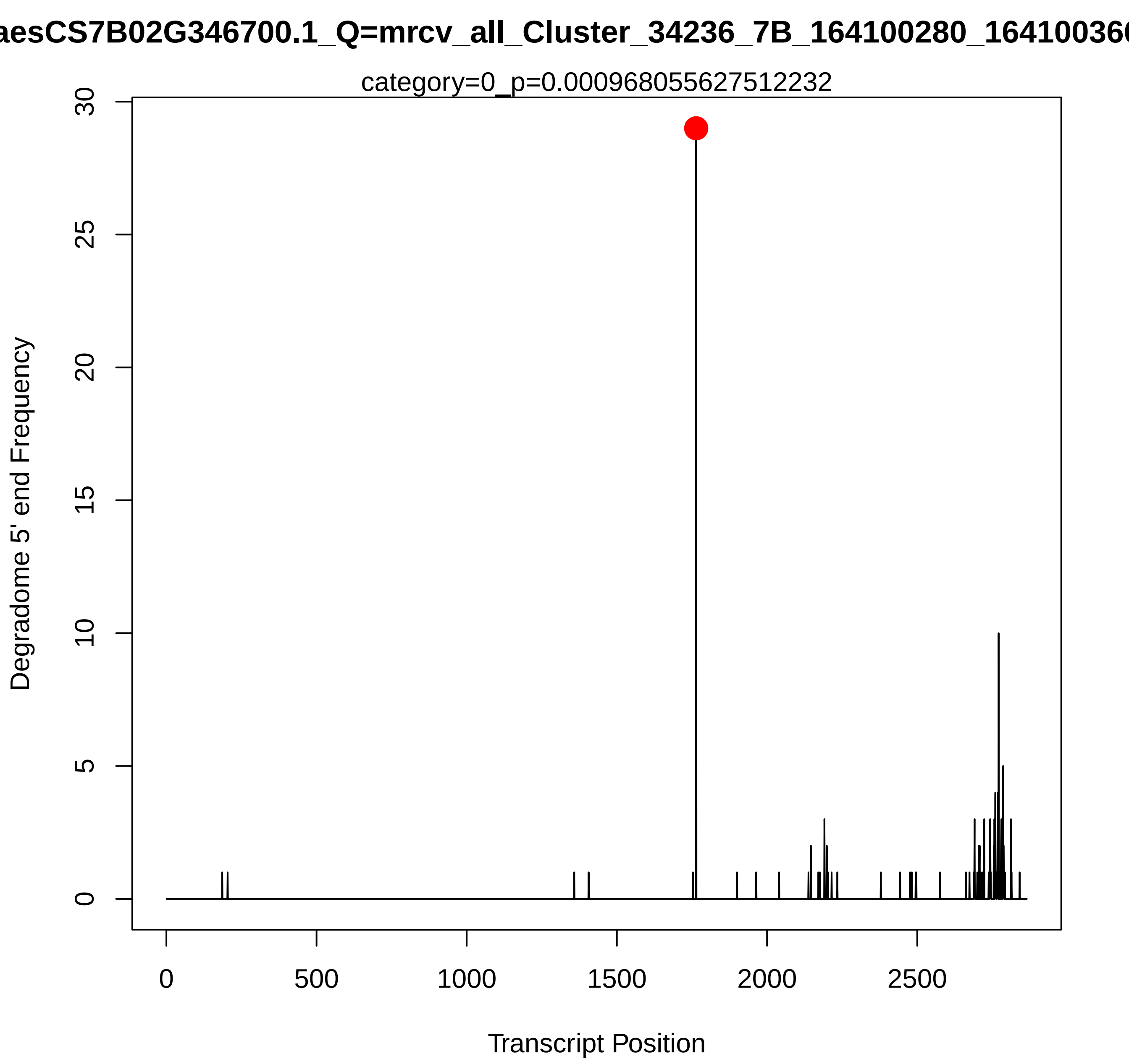
aesCS7B02G346700.1_Q=mrcv_all_Cluster_34236_7B_164100280_16410036
category=0_p=0.000968055627512232
Degradome 5' end Frequency
Transcript Position
0
5
10
15
20
25
30
0
500
1000
1500
2000
2500

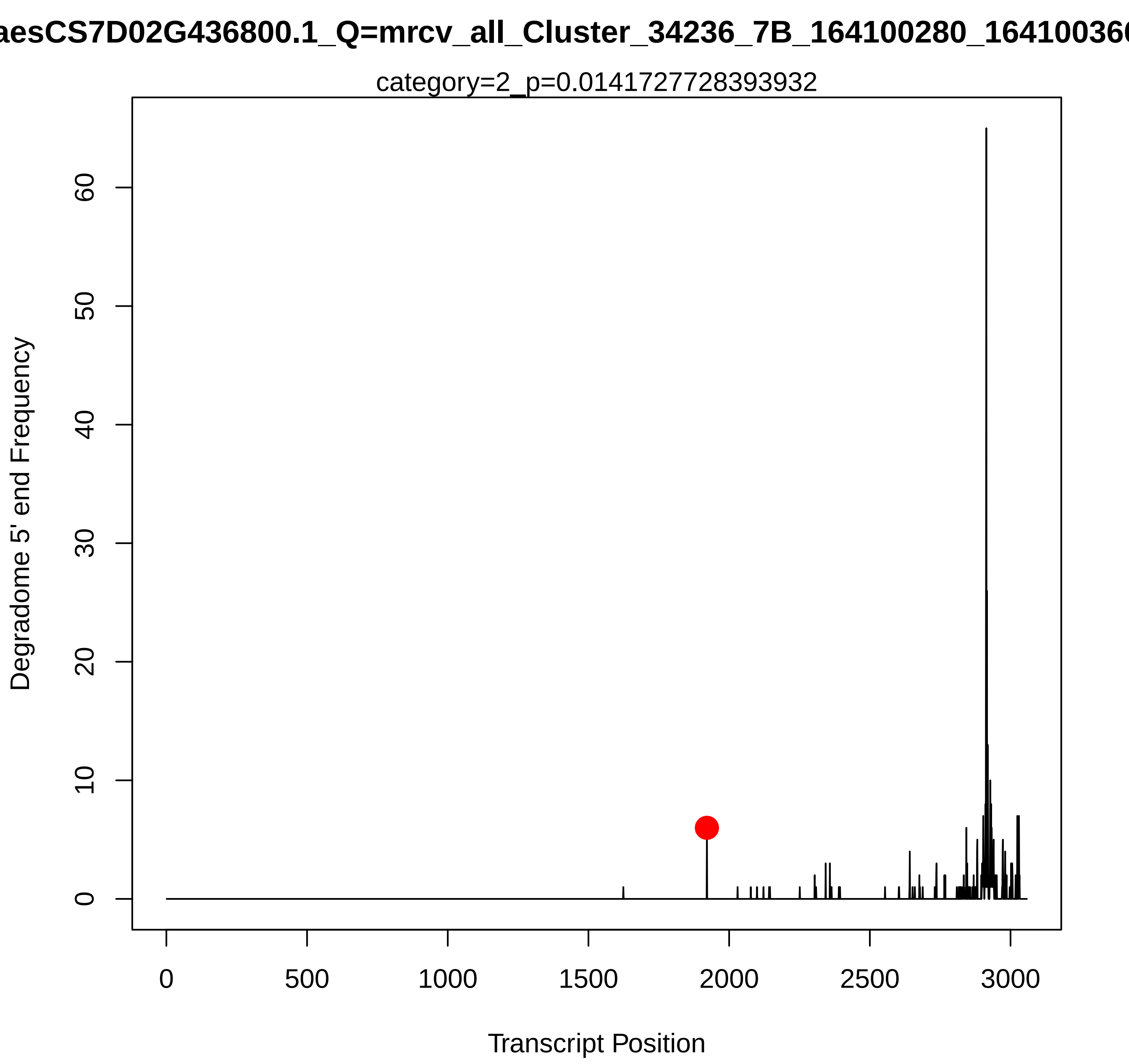

aesCS7D02G436800.1_Q=mrcv_all_Cluster_34236_7B_164100280_16410036
category=2_p=0.0141727728393932
Degradome 5' end Frequency
0
10
20
30
40
50
60
0
500
1000
1500
2000
2500
3000
Transcript Position

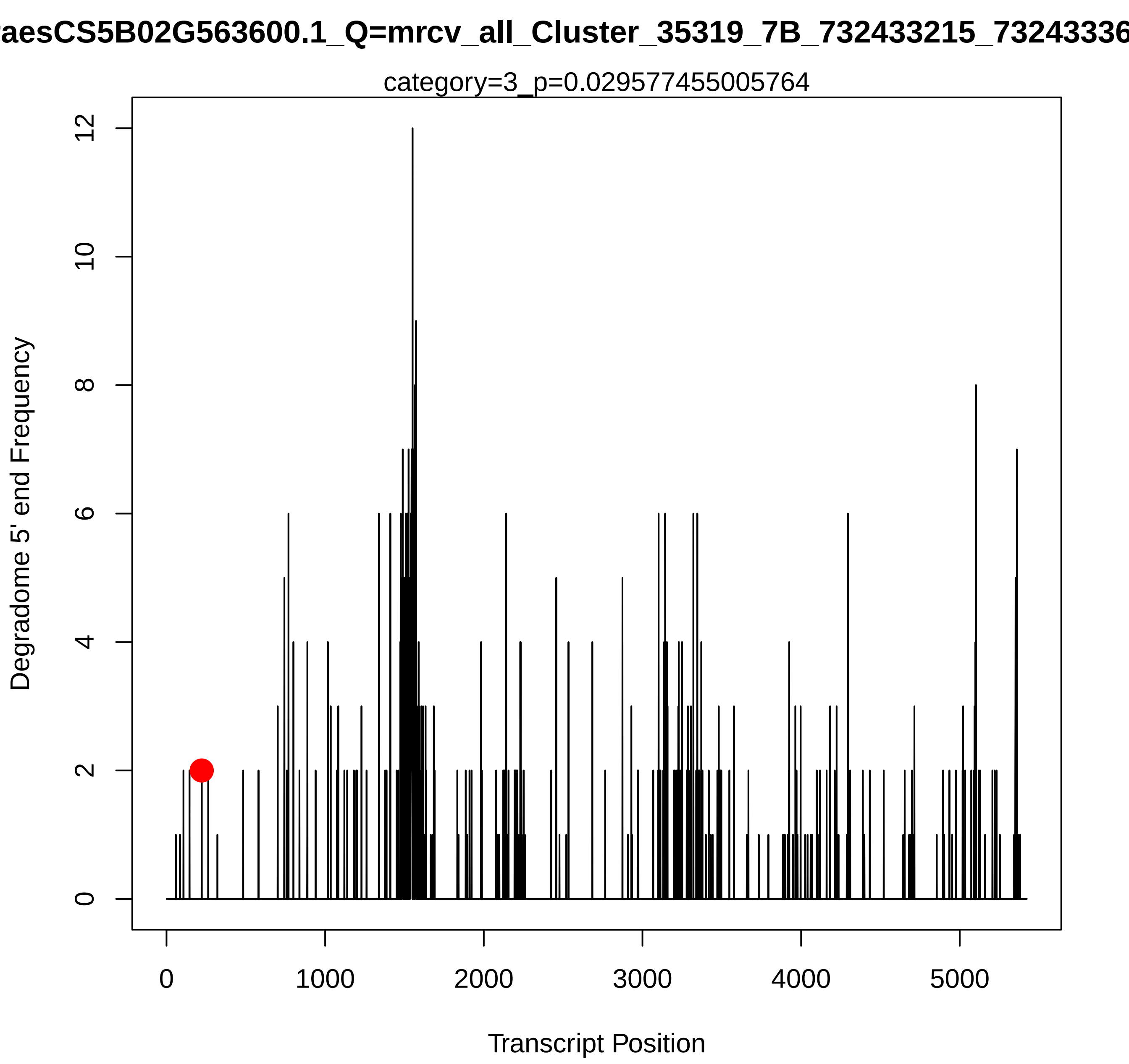
aesCS5B02G563600.1_Q=mrcv_all_Cluster_35319_7B_732433215_73243336
category=3_p=0.029577455005764
Degradome 5' end Frequency
Transcript Position
0
2
4
6
8
10
12
0
1000
2000
3000
4000
5000

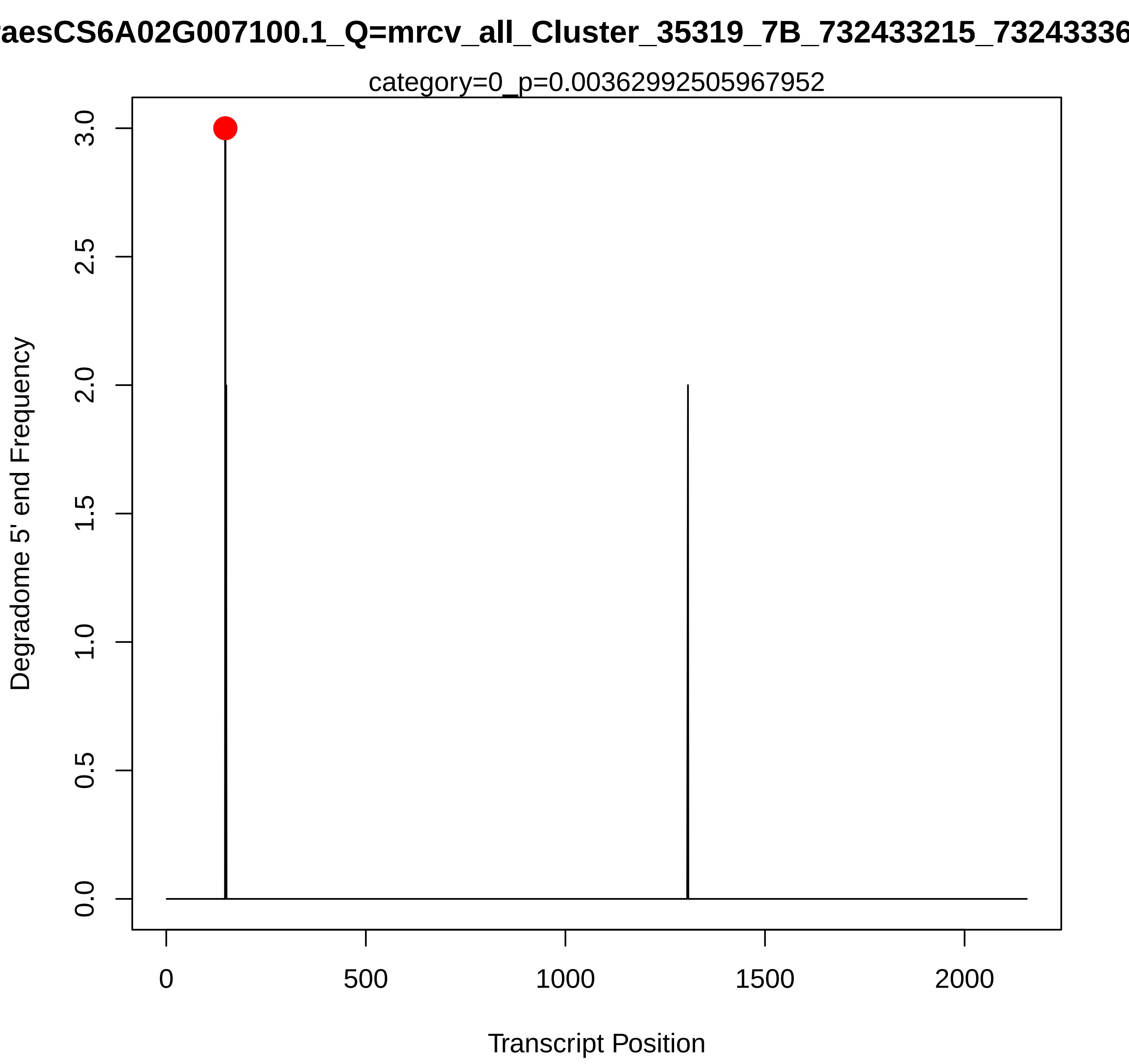

aesCS6A02G007100.1_Q=mrcv_all_Cluster_35319_7B_732433215_73243336
category=0_p=0.00362992505967952
Degradome 5' end Frequency
0.0
0.5
1.0
1.5
2.0
2.5
3.0
0
500
1000
1500
2000
Transcript Position

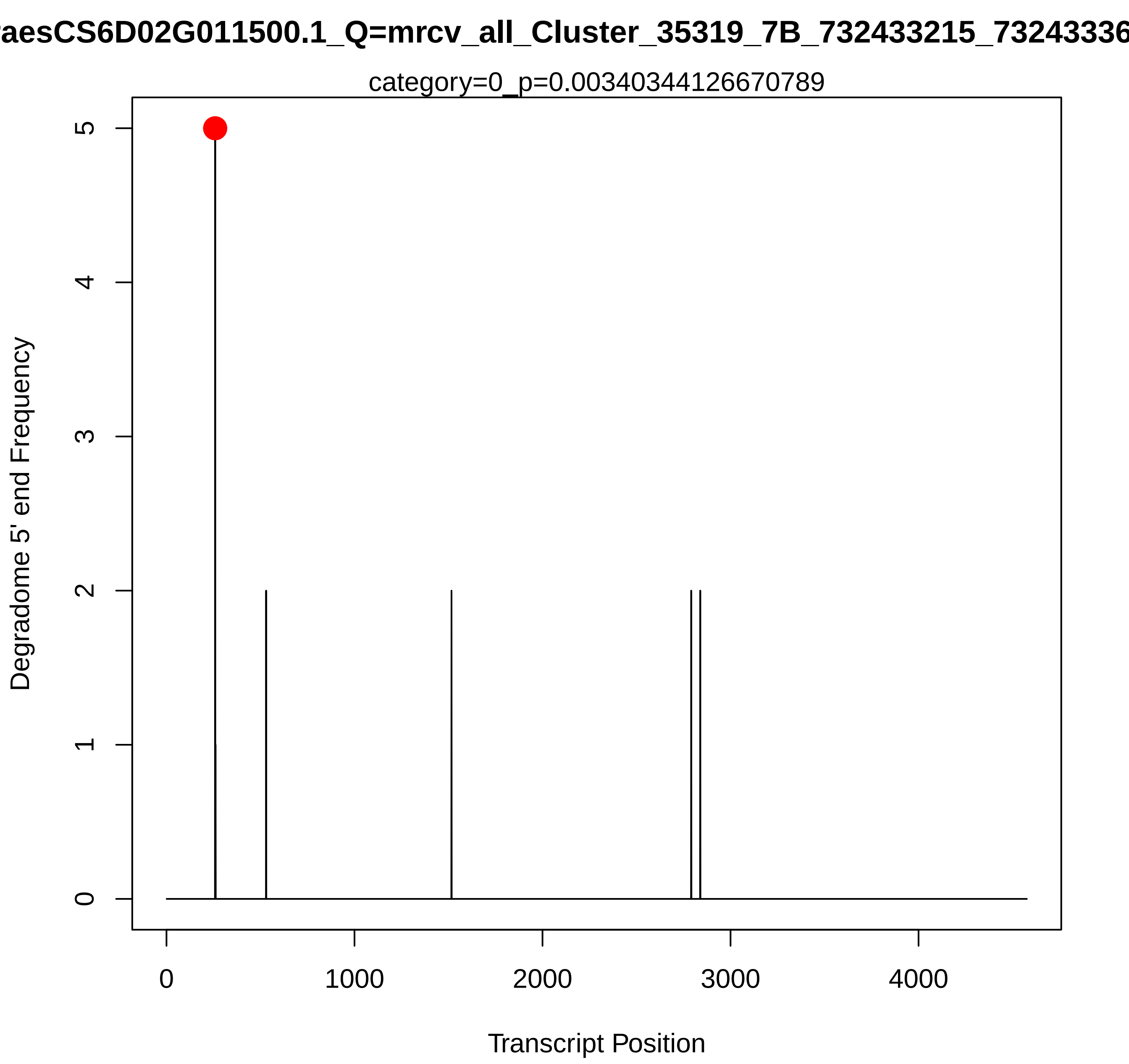
TraesCS6D02G011500.1_Q=mrcv_all_Cluster_35319_7B_732433215_73243336
category=0_p=0.00340344126670789
Degradome 5' end Frequency
Transcript Position
0
1
2
3
4
5
0
1000
2000
3000
4000

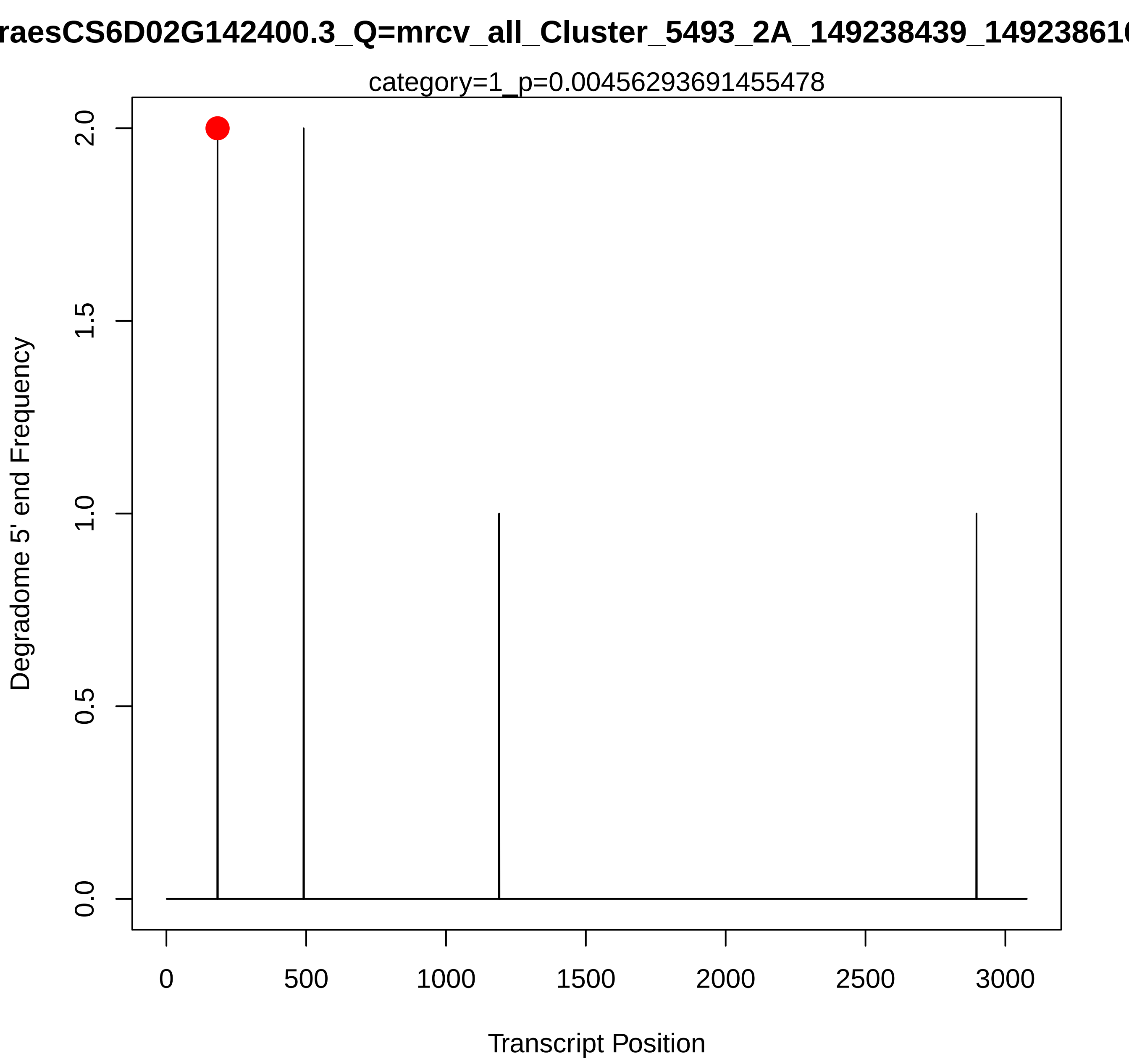
raesCS6D02G142400.3_Q=mrcv_all_Cluster_5493_2A_149238439_14923861
category=1_p=0.00456293691455478
Degradome 5' end Frequency
0.0
0.5
1.0
1.5
2.0
0
500
1000
1500
2000
2500
3000
Transcript Position

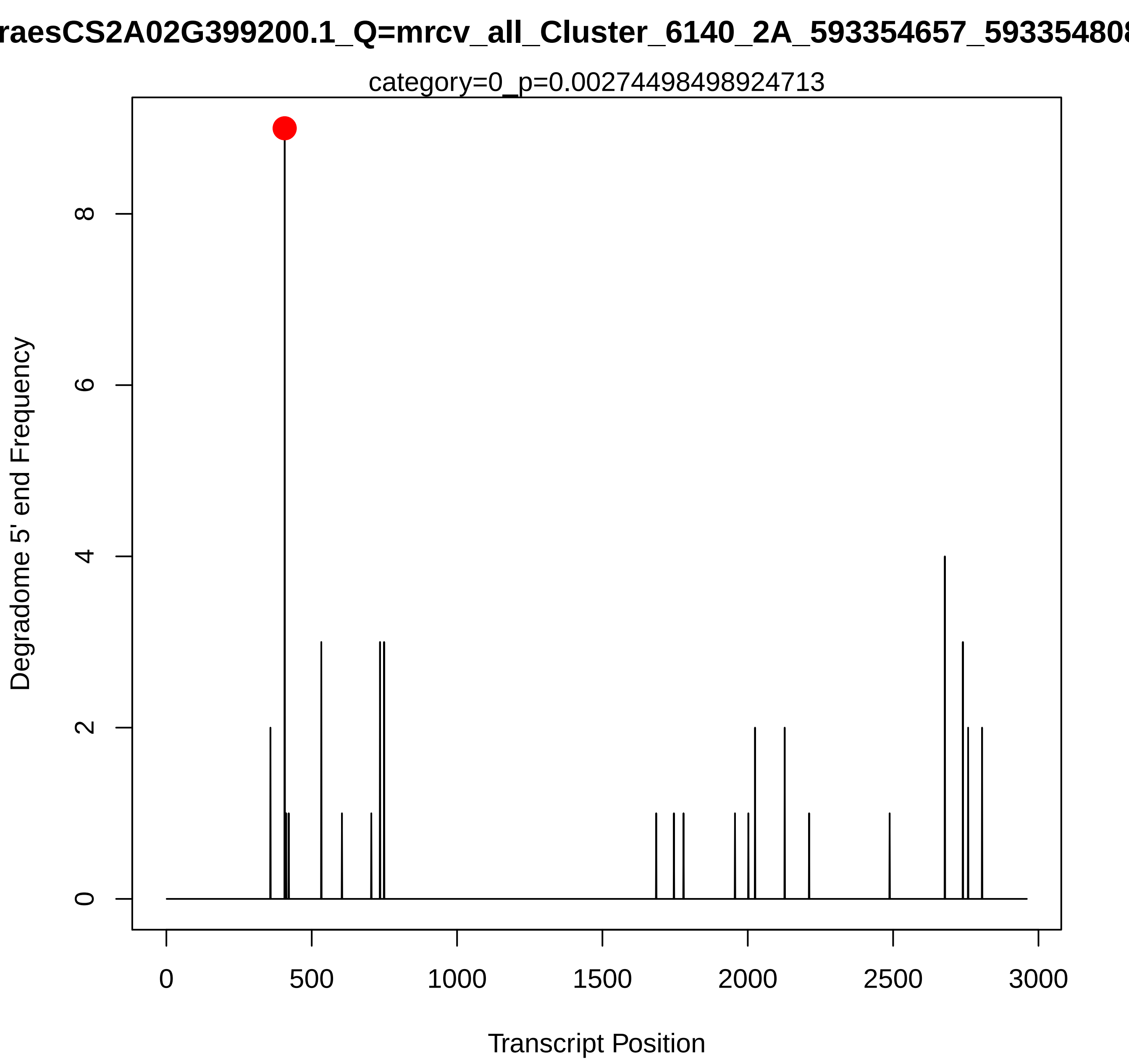

raesCS2A02G399200.1_Q=mrcv_all_Cluster_6140_2A_593354657_59335480
category=0_p=0.00274498498924713
Degradome 5' end Frequency
0
2
4
6
8
0
500
1000
1500
2000
2500
3000
Transcript Position

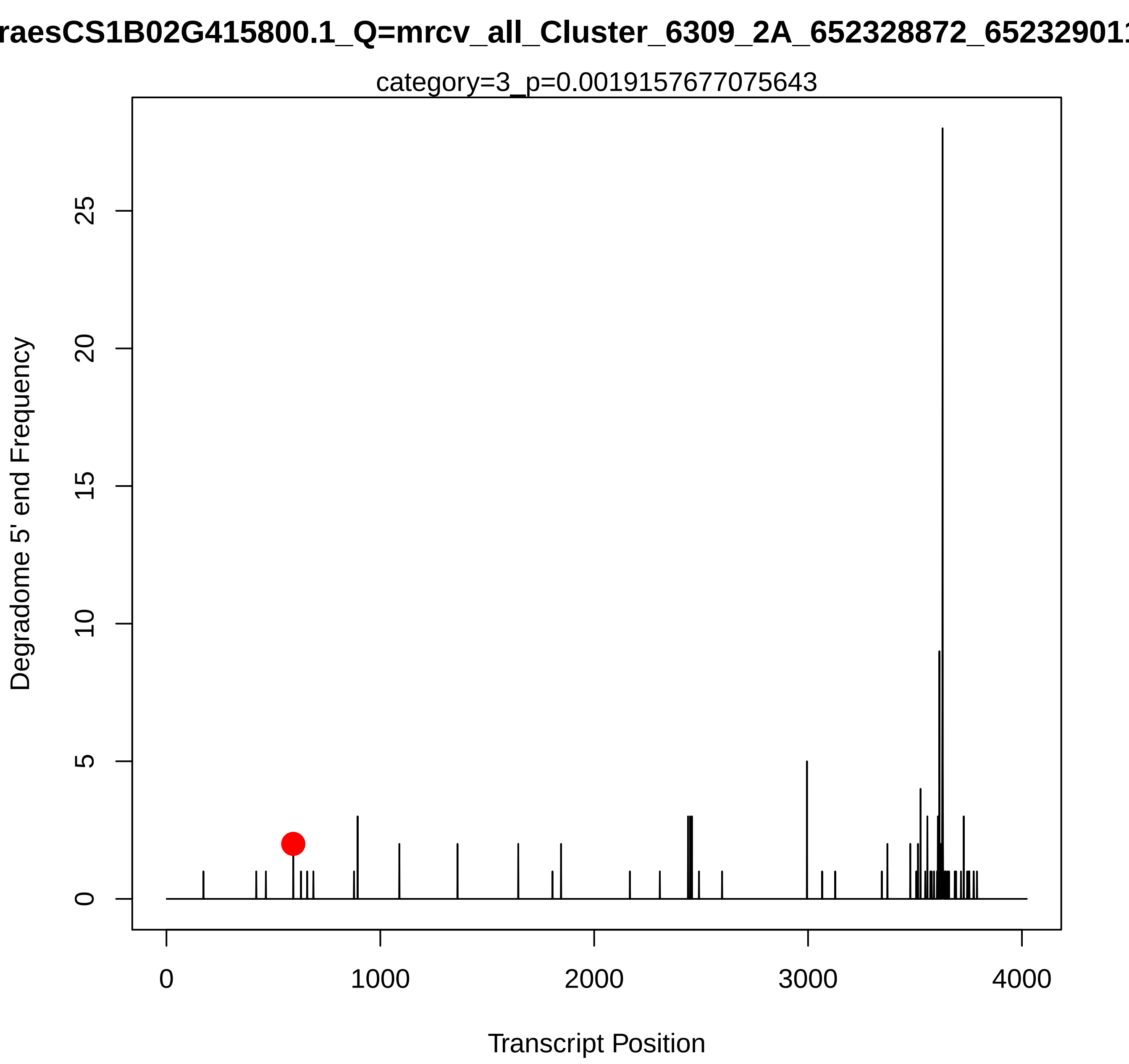

raesCS1B02G415800.1_Q=mrcv_all_Cluster_6309_2A_652328872_65232901
category=3_p=0.0019157677075643
Degradome 5' end Frequency
0
5
10
15
20
25
0
1000
2000
3000
4000
Transcript Position

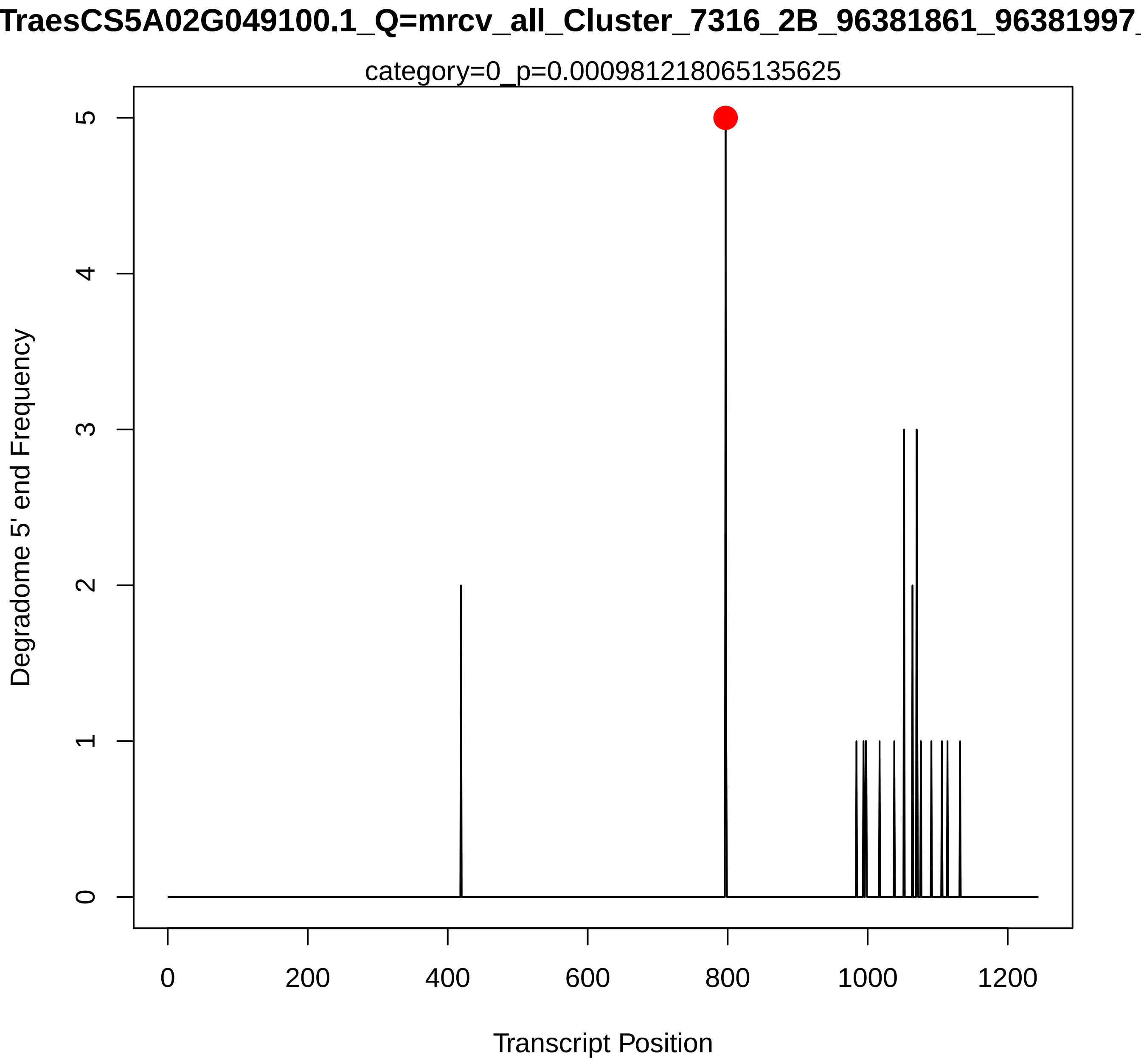

TraesCS5A02G049100.1_Q=mrcv_all_Cluster_7316_2B_96381861_96381997_
category=0_p=0.000981218065135625
Degradome 5' end Frequency
0
1
2
3
4
5
0
200
400
600
800
1000
1200
Transcript Position

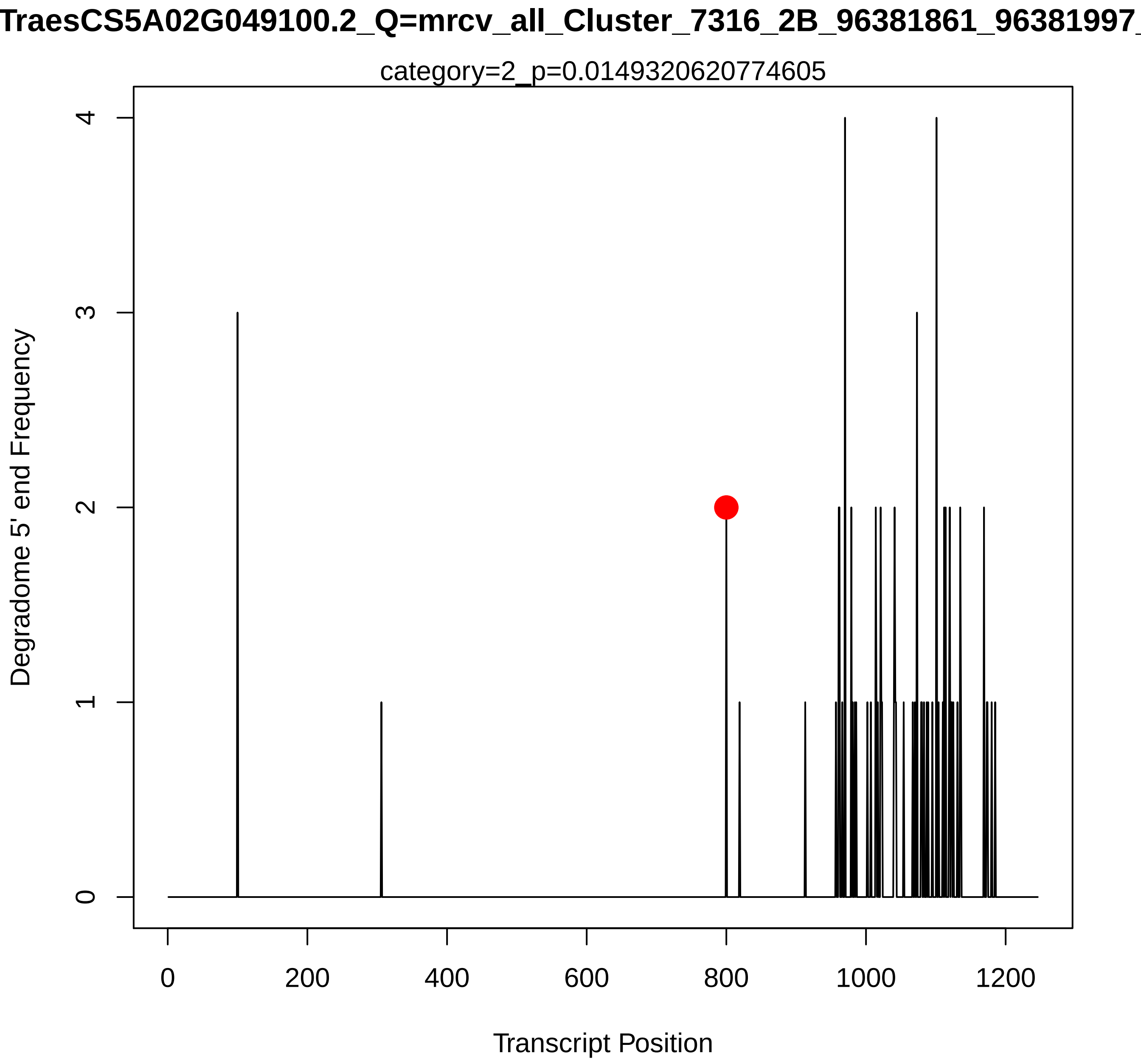
TraesCS5A02G049100.2_Q=mrcv_all_Cluster_7316_2B_96381861_96381997_
category=2_p=0.0149320620774605
Degradome 5' end Frequency
Transcript Position
0
1
2
3
4
0
200
400
600
800
1000
1200

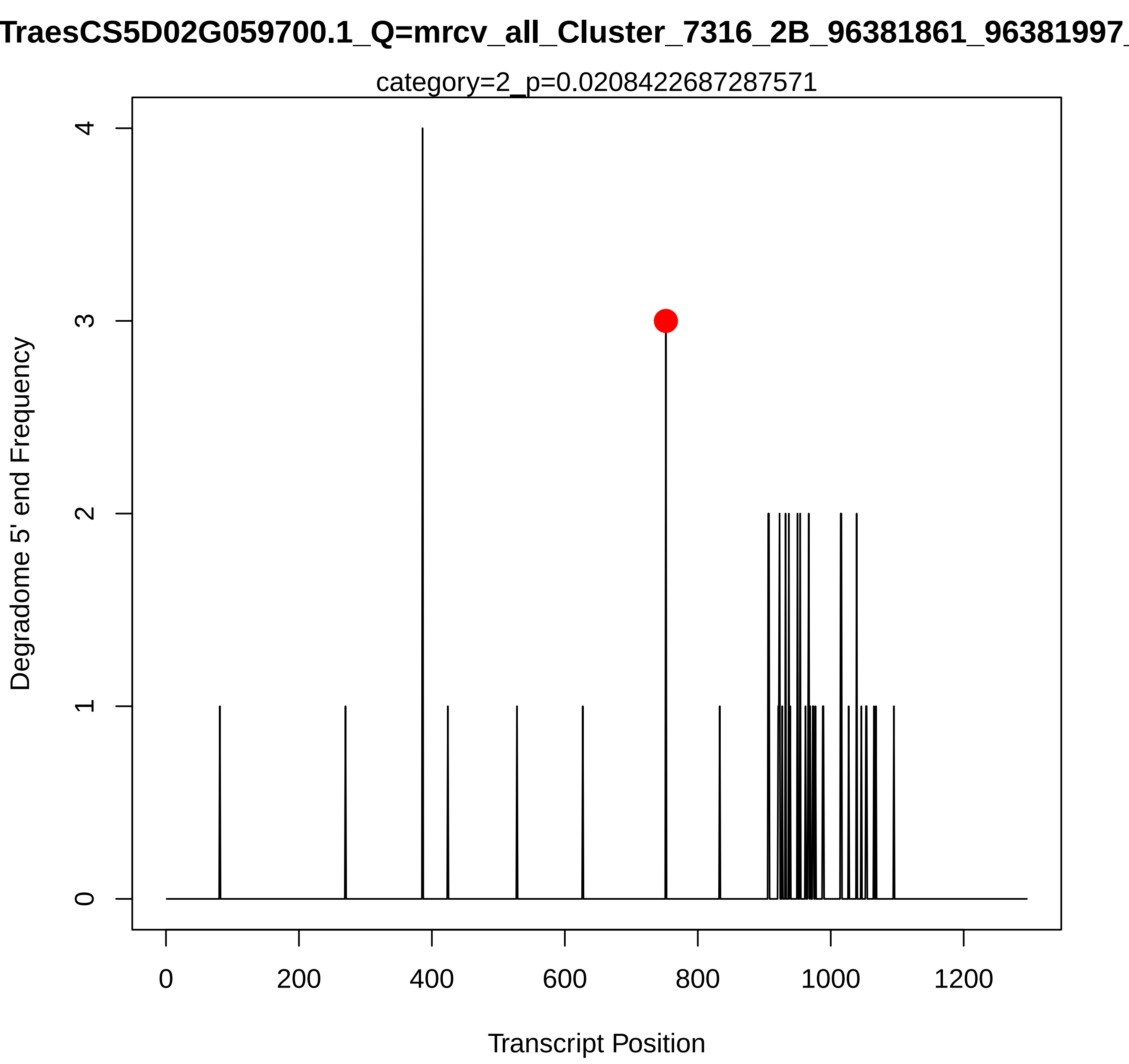

TraesCS5D02G059700.1_Q=mrcv_all_Cluster_7316_2B_96381861_96381997_
category=2_p=0.0208422687287571
Degradome 5' end Frequency
0
1
2
3
4
0
200
400
600
800
1000
1200
Transcript Position

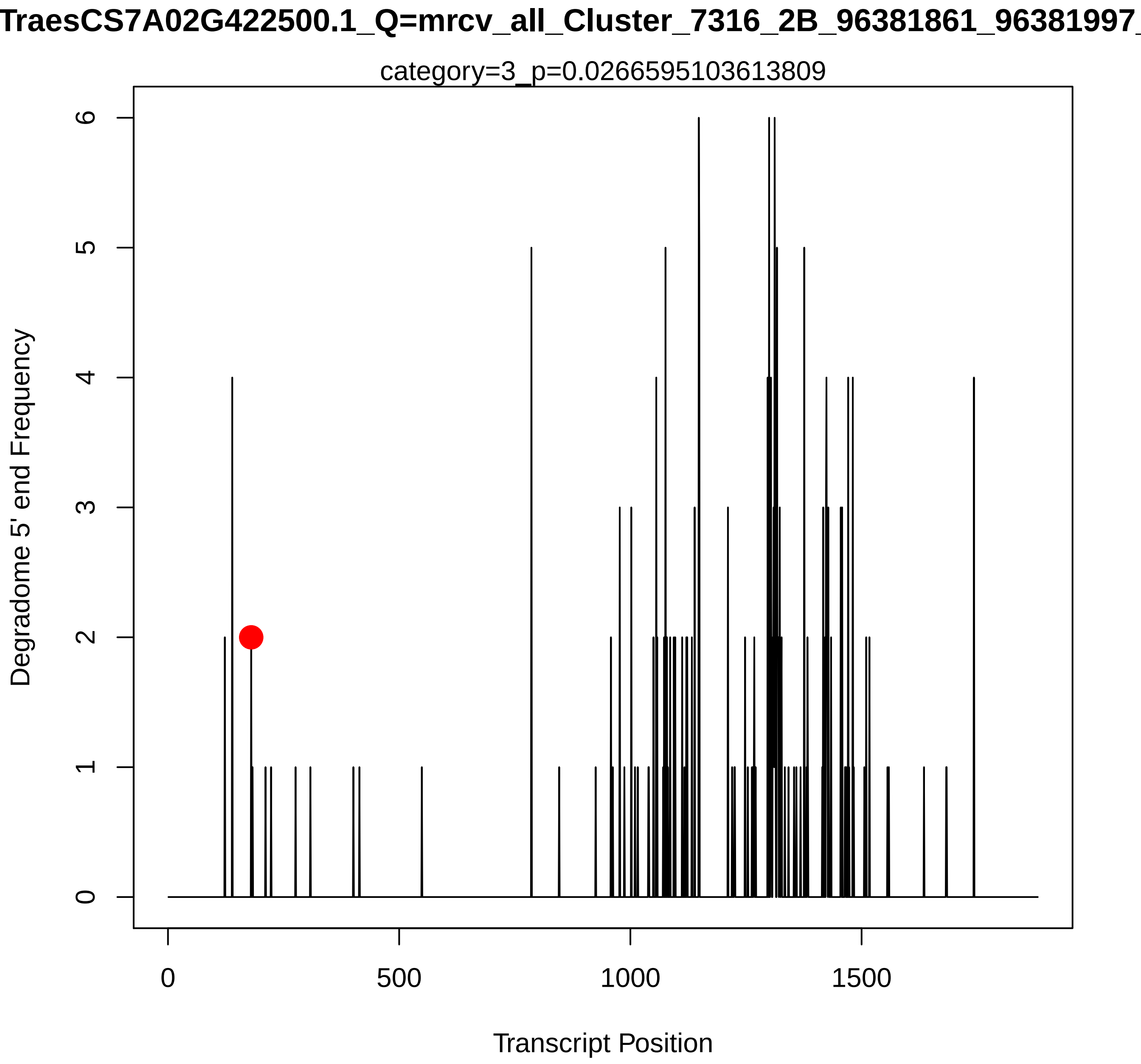

TraesCS7A02G422500.1_Q=mrcv_all_Cluster_7316_2B_96381861_96381997_
category=3_p=0.0266595103613809
Degradome 5' end Frequency
0
1
2
3
4
5
6
0
500
1000
1500
Transcript Position

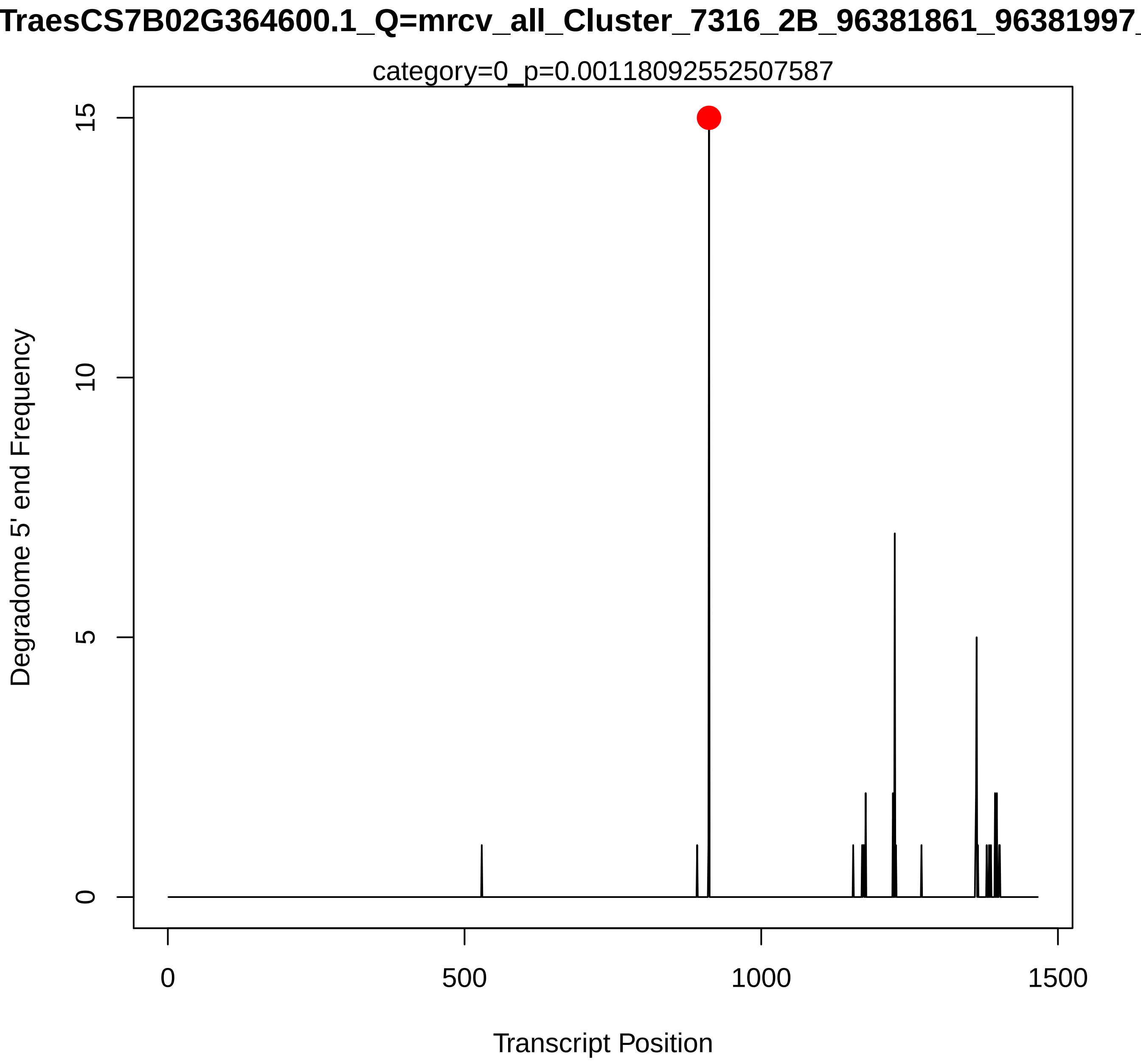
TraesCS7B02G364600.1_Q=mrcv_all_Cluster_7316_2B_96381861_96381997_
category=0_p=0.00118092552507587
Degradome 5' end Frequency
0
5
10
15
0
500
1000
1500
Transcript Position

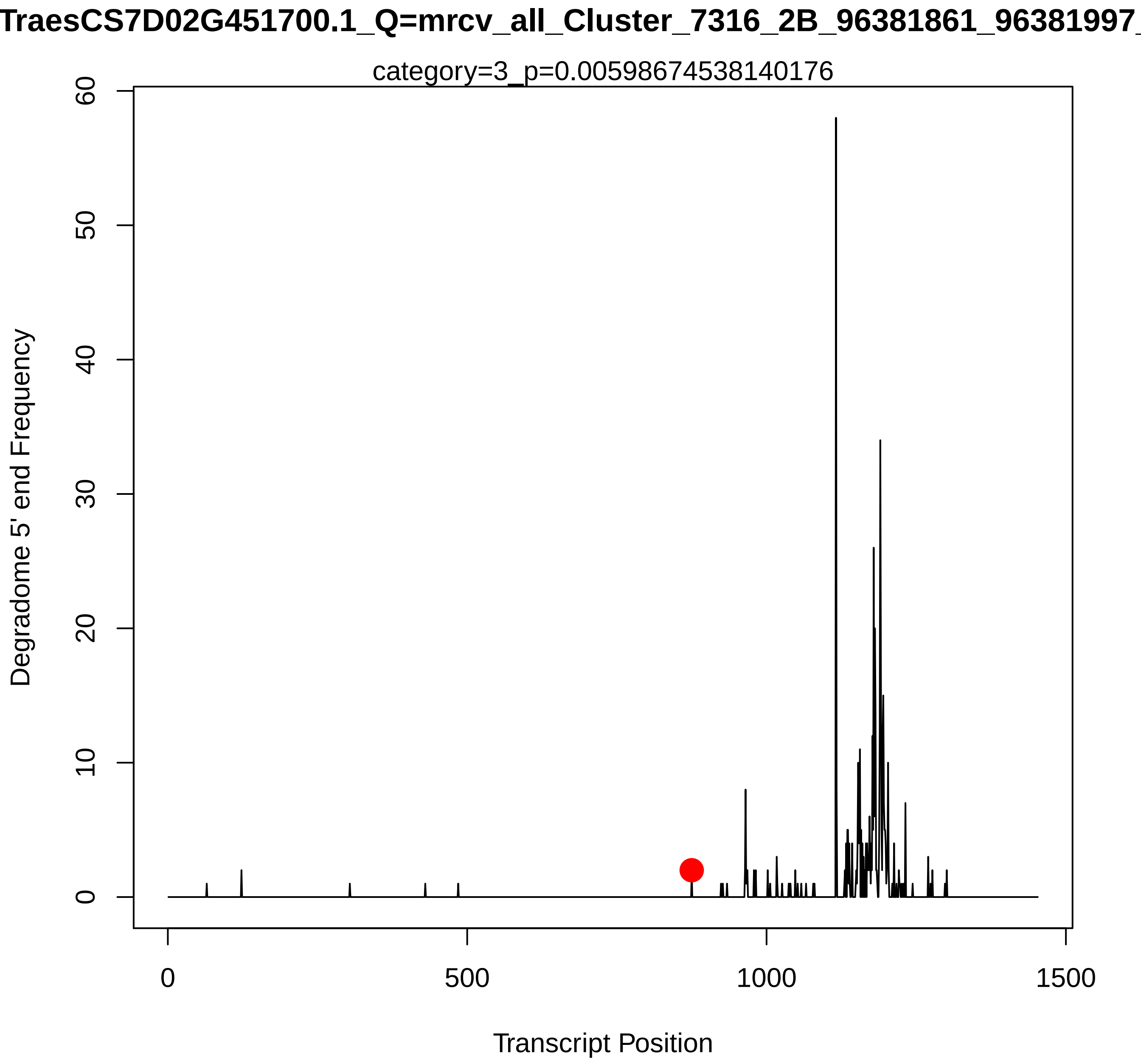
TraesCS7D02G451700.1_Q=mrcv_all_Cluster_7316_2B_96381861_96381997_
category=3_p=0.00598674538140176
Degradome 5' end Frequency
Transcript Position
0
10
20
30
40
50
60
0
500
1000
1500

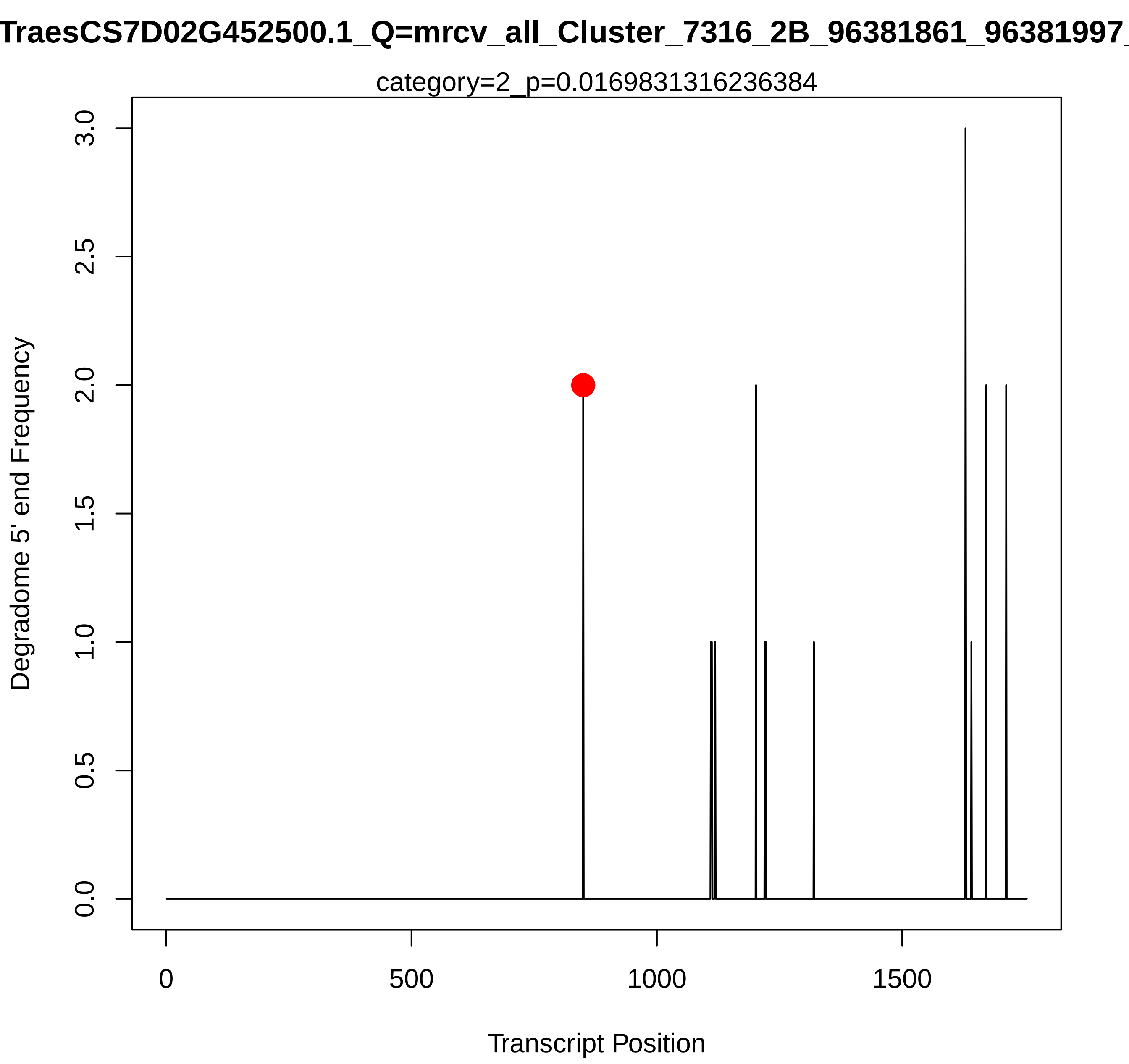
TraesCS7D02G452500.1_Q=mrcv_all_Cluster_7316_2B_96381861_96381997_
category=2_p=0.0169831316236384
Degradome 5' end Frequency
0.0
0.5
1.0
1.5
2.0
2.5
3.0
0
500
1000
1500
Transcript Position

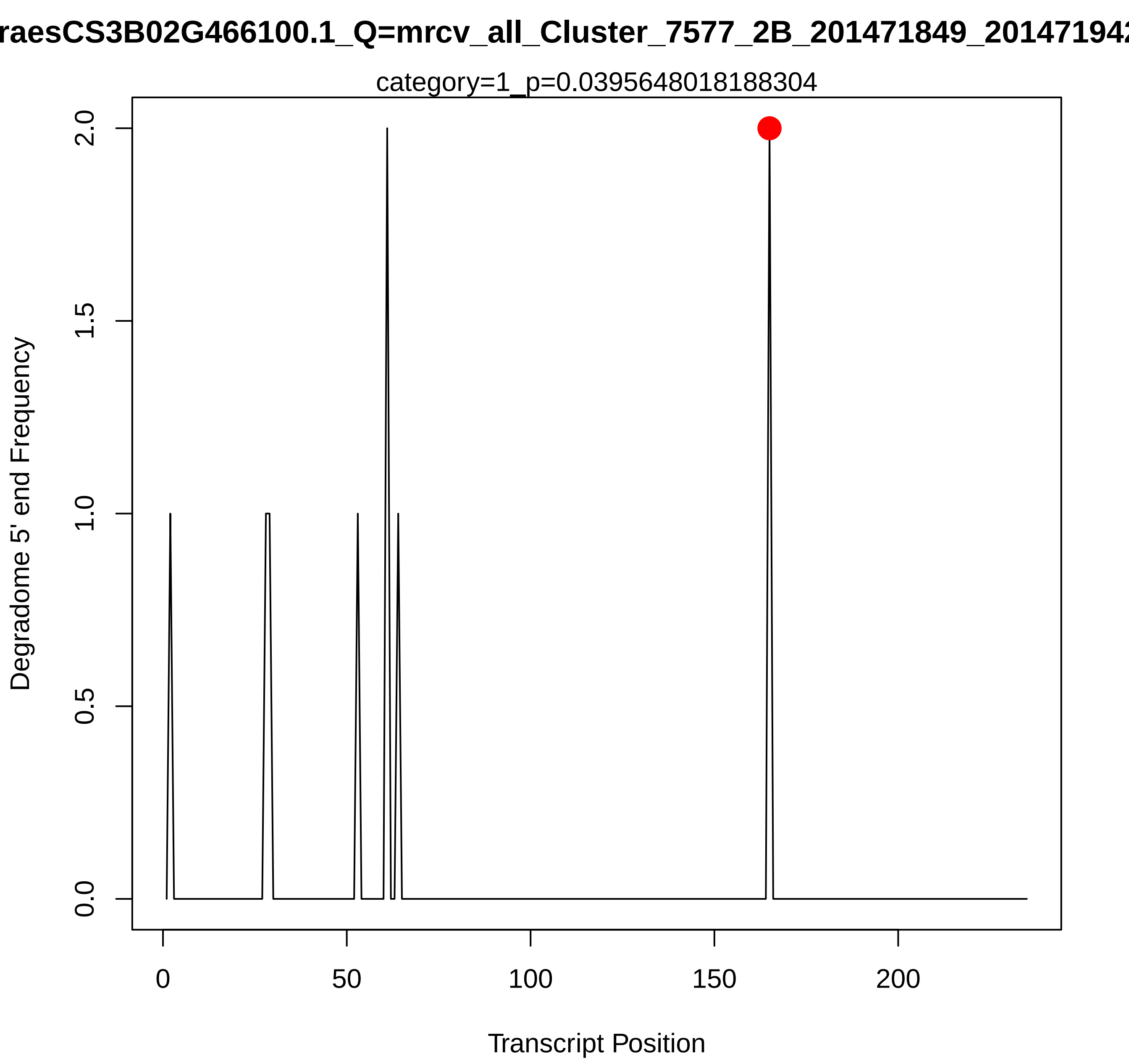
raesCS3B02G466100.1_Q=mrcv_all_Cluster_7577_2B_201471849_20147194
category=1_p=0.0395648018188304
Degradome 5' end Frequency
0.0
0.5
1.0
1.5
2.0
0
50
100
150
200
Transcript Position

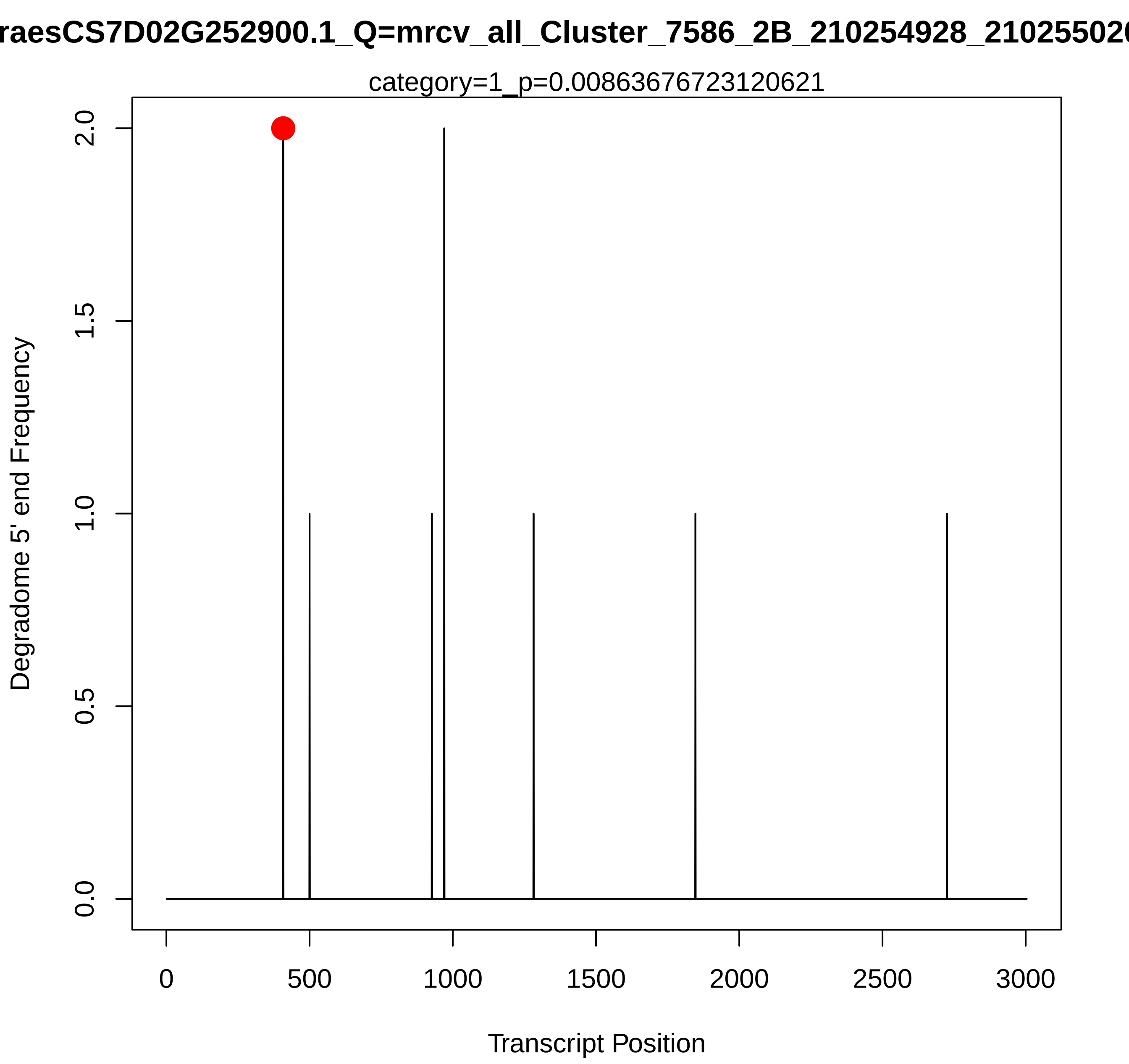
raesCS7D02G252900.1_Q=mrcv_all_Cluster_7586_2B_210254928_21025502
category=1_p=0.00863676723120621
Degradome 5' end Frequency
0.0
0.5
1.0
1.5
2.0
0
500
1000
1500
2000
2500
3000
Transcript Position

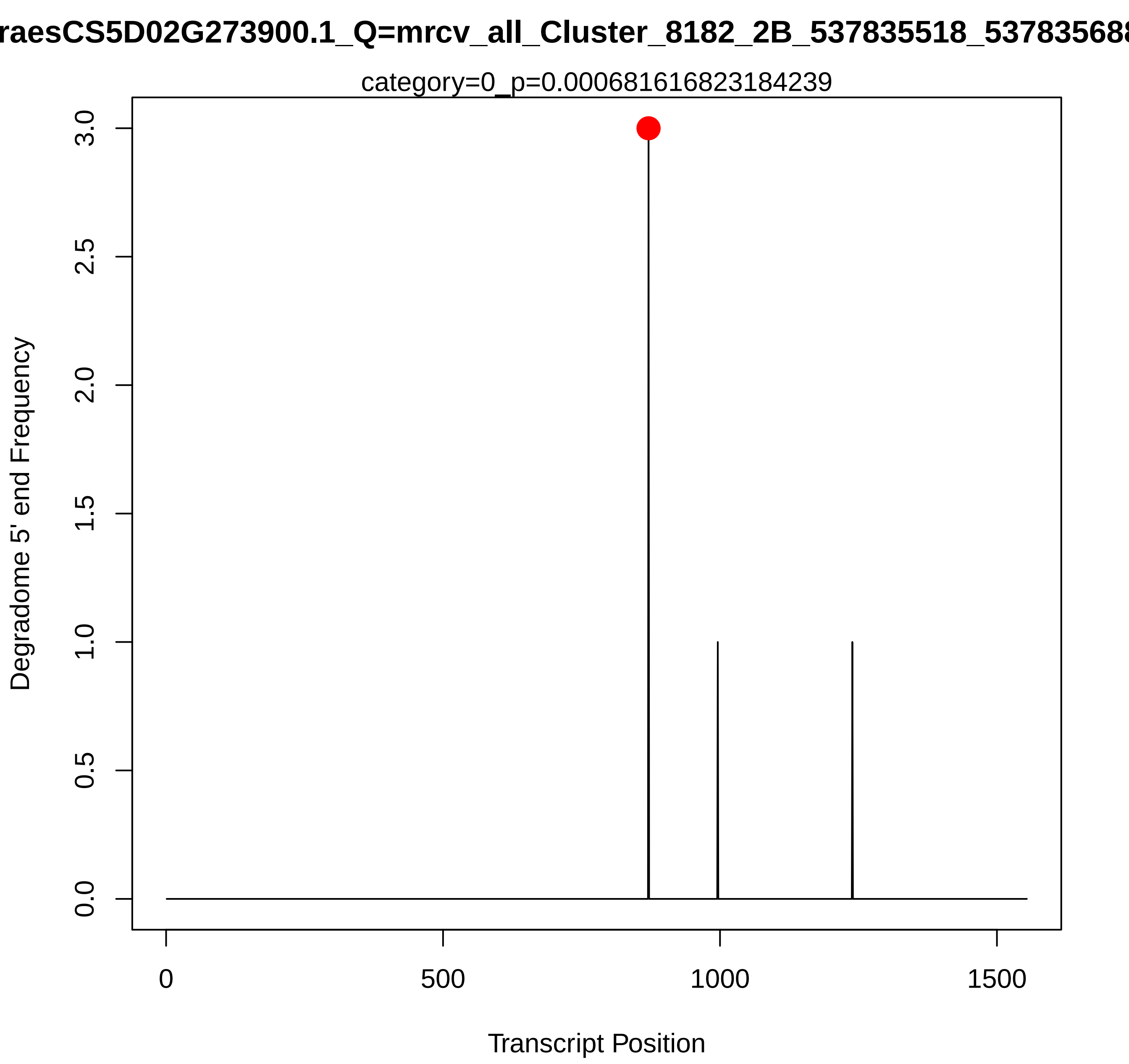
raesCS5D02G273900.1_Q=mrcv_all_Cluster_8182_2B_537835518_53783568
category=0_p=0.000681616823184239
Degradome 5' end Frequency
0.0
0.5
1.0
1.5
2.0
2.5
3.0
0
500
1000
1500
Transcript Position

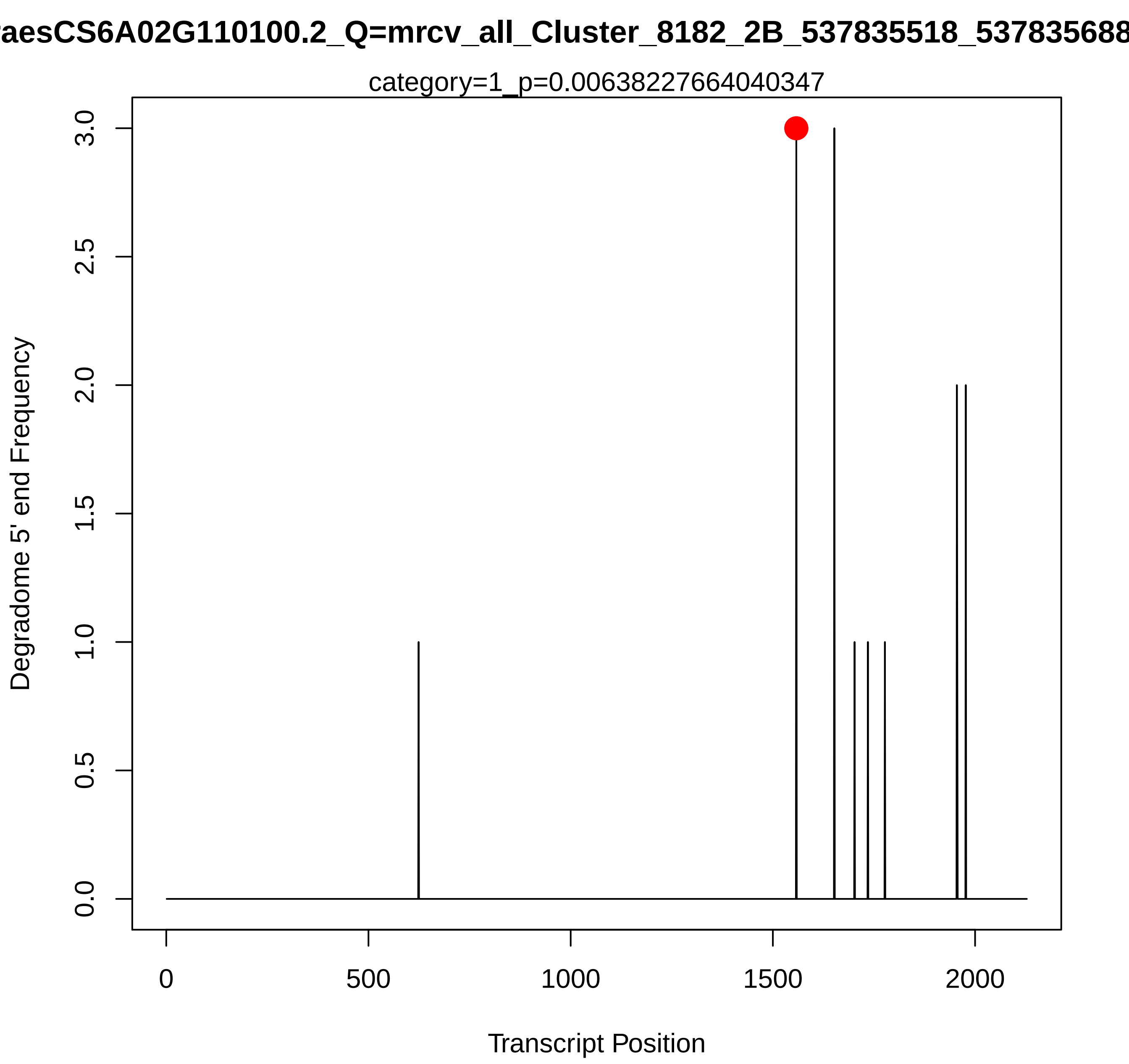

aesCS6A02G110100.2_Q=mrcv_all_Cluster_8182_2B_537835518_537835688
category=1_p=0.00638227664040347
Degradome 5' end Frequency
Transcript Position
0.0
0.5
1.0
1.5
2.0
2.5
3.0
0
500
1000
1500
2000

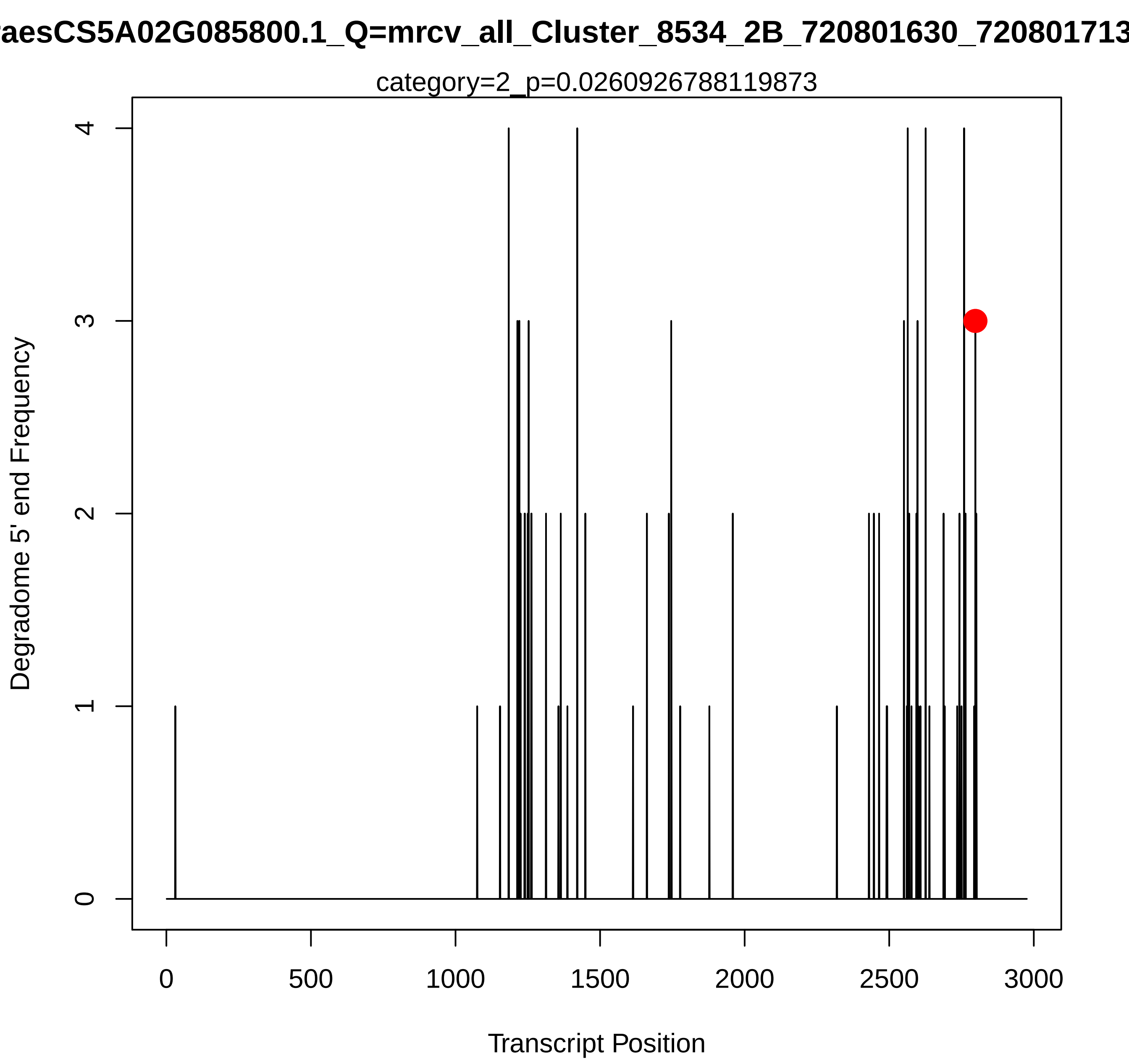
aesCS5A02G085800.1_Q=mrcv_all_Cluster_8534_2B_720801630_72080171
category=2_p=0.0260926788119873
Degradome 5' end Frequency
0
1
2
3
4
0
500
1000
1500
2000
2500
3000
Transcript Position

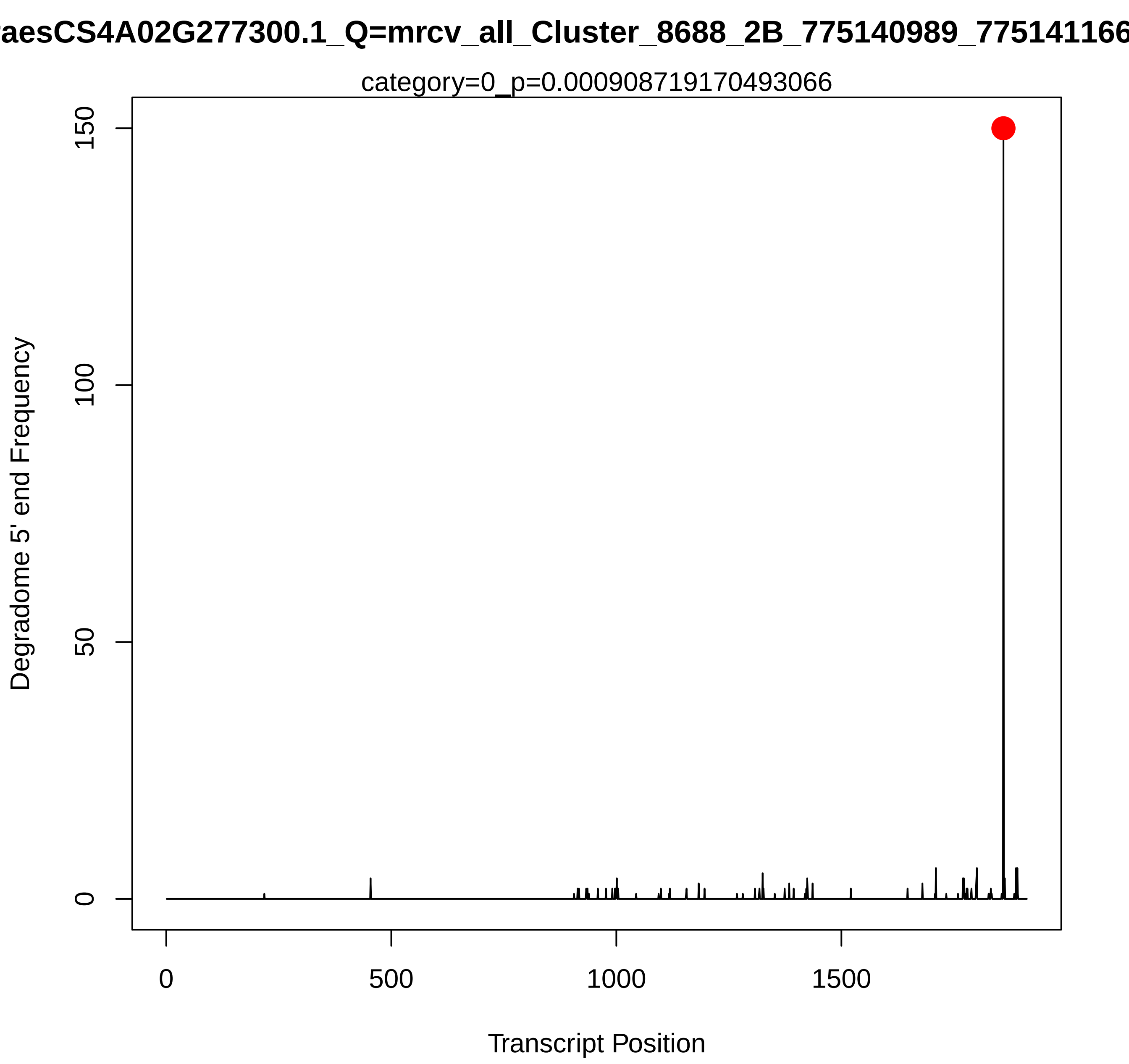

aesCS4A02G277300.1_Q=mrcv_all_Cluster_8688_2B_775140989_775141166
category=0_p=0.000908719170493066
Degradome 5' end Frequency
150
100
50
0
0
500
1000
1500
Transcript Position

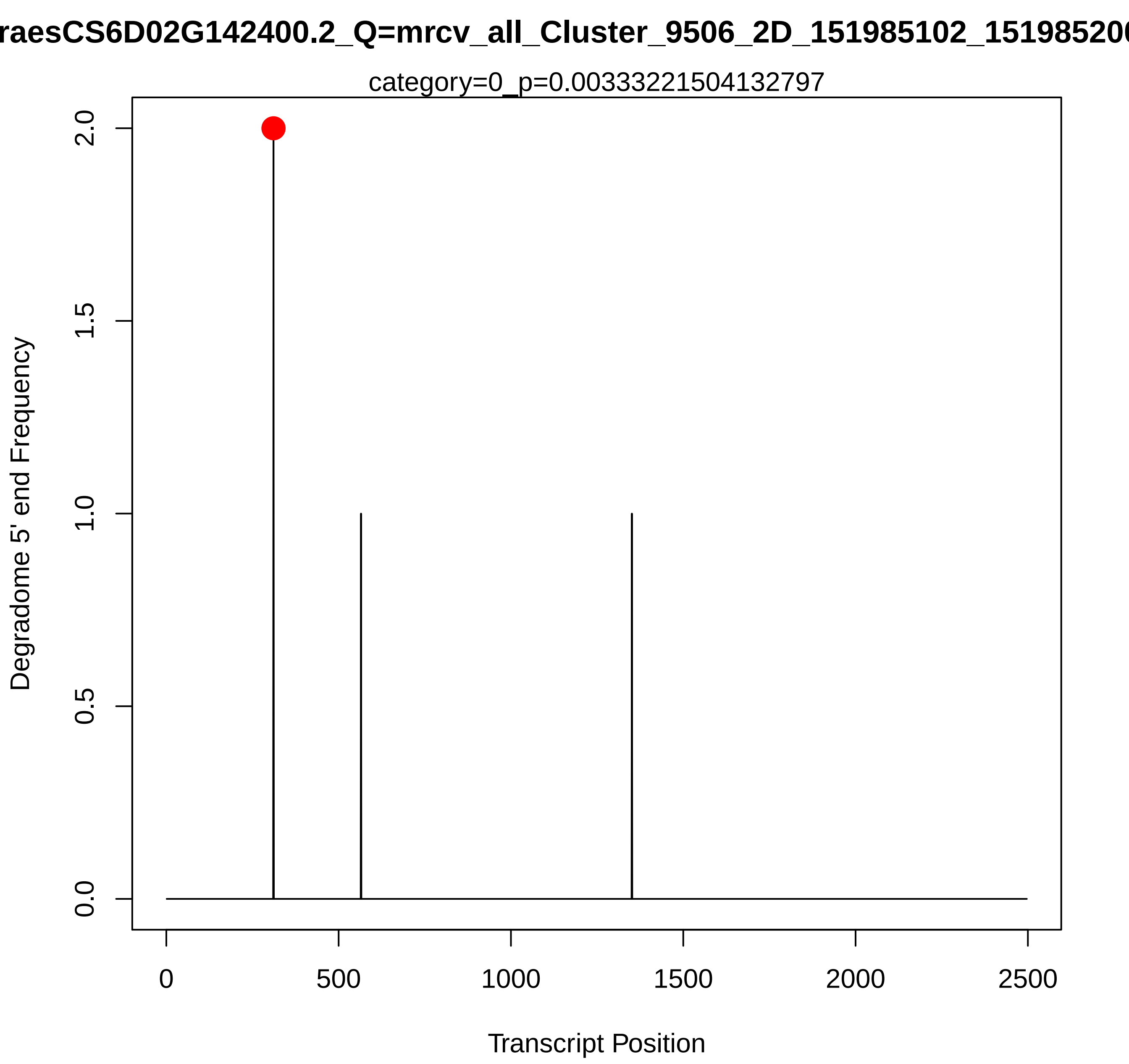

raesCS6D02G142400.2_Q=mrcv_all_Cluster_9506_2D_151985102_15198520
category=0_p=0.00333221504132797
Degradome 5' end Frequency
0.0
0.5
1.0
1.5
2.0
0
500
1000
1500
2000
2500
Transcript Position

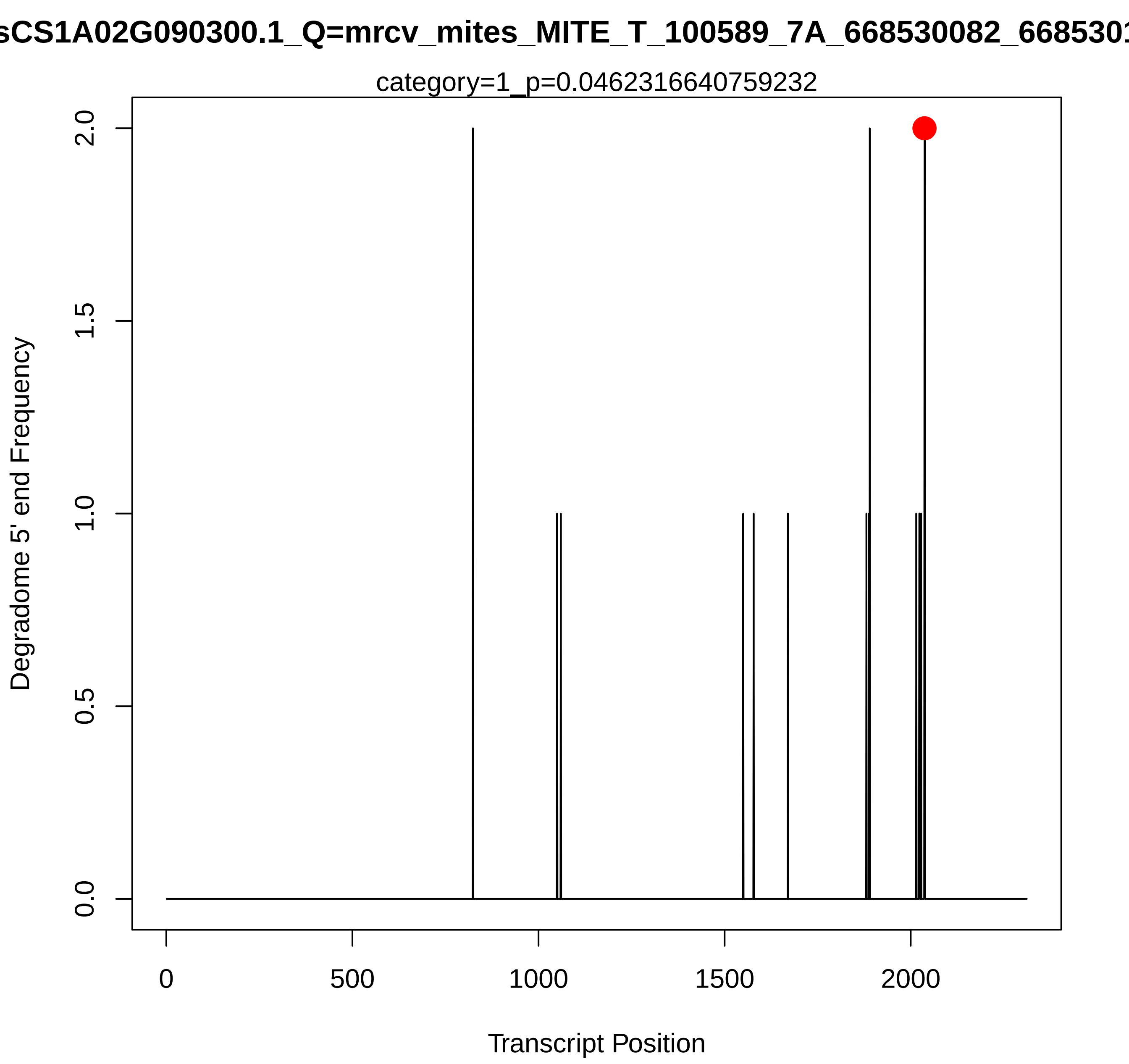

sCS1A02G090300.1_Q=mrcv_mites_MITE_T_100589_7A_668530082_6685301
category=1_p=0.0462316640759232
Degradome 5' end Frequency
0.0
0.5
1.0
1.5
2.0
0
500
1000
1500
2000
Transcript Position

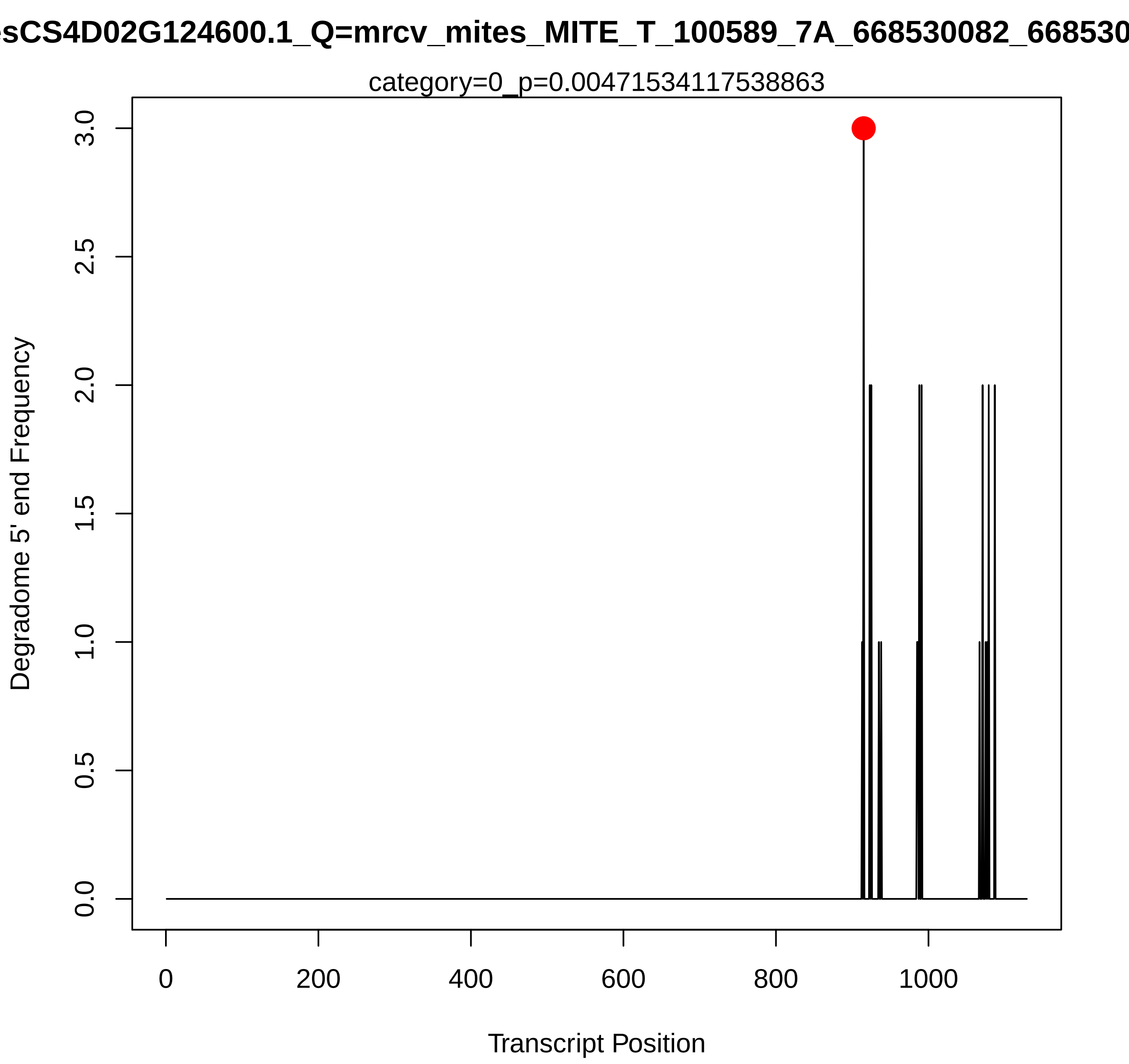
esCS4D02G124600.1_Q=mrcv_mites_MITE_T_100589_7A_668530082_668530
category=0_p=0.00471534117538863
Degradome 5' end Frequency
Transcript Position
0.0
0.5
1.0
1.5
2.0
2.5
3.0
0
200
400
600
800
1000

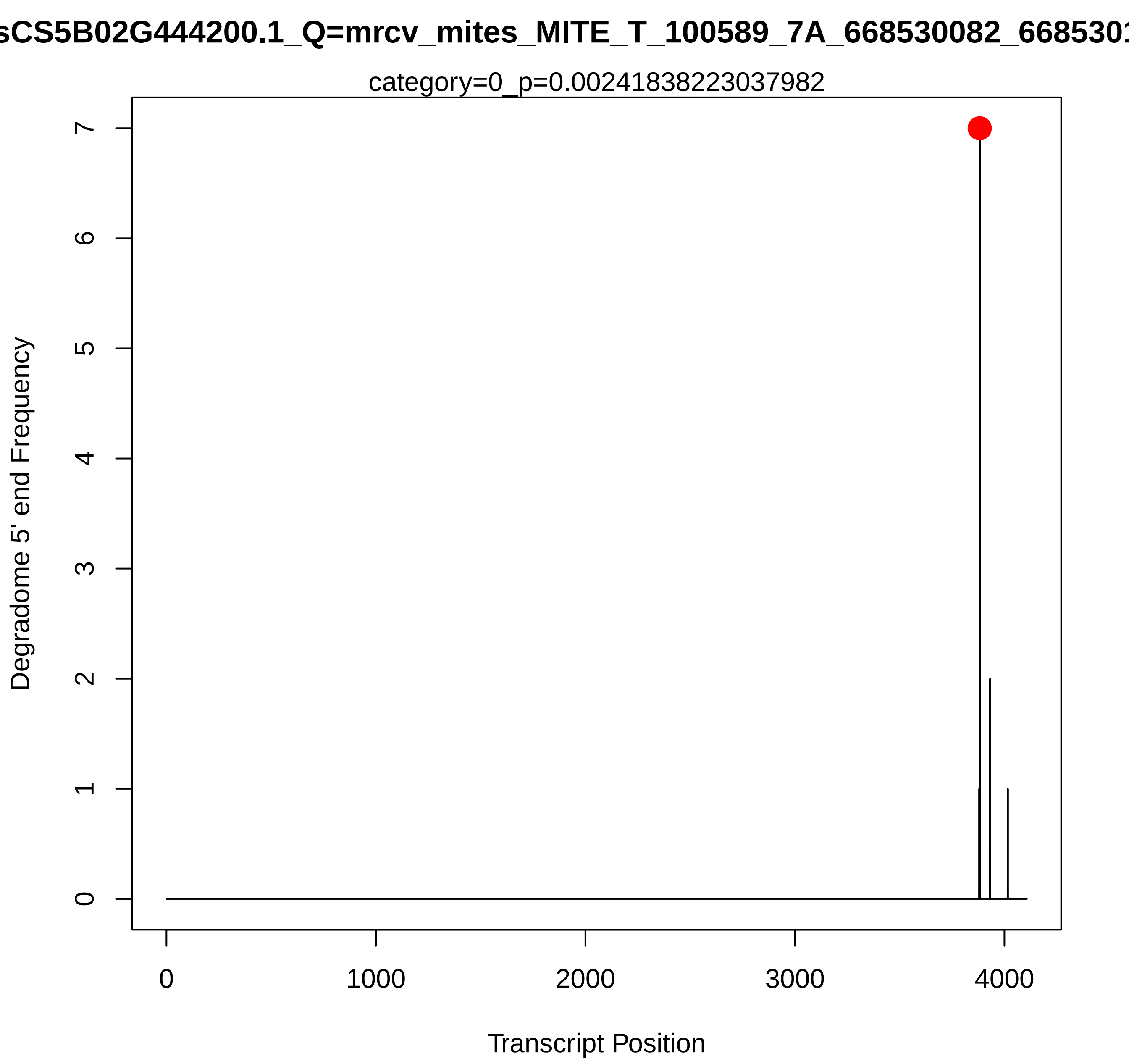
sCS5B02G444200.1_Q=mrcv_mites_MITE_T_100589_7A_668530082_66853O1
category=0_p=0.00241838223037982
Degradome 5' end Frequency
0
1
2
3
4
5
6
7
0
1000
2000
3000
4000
Transcript Position

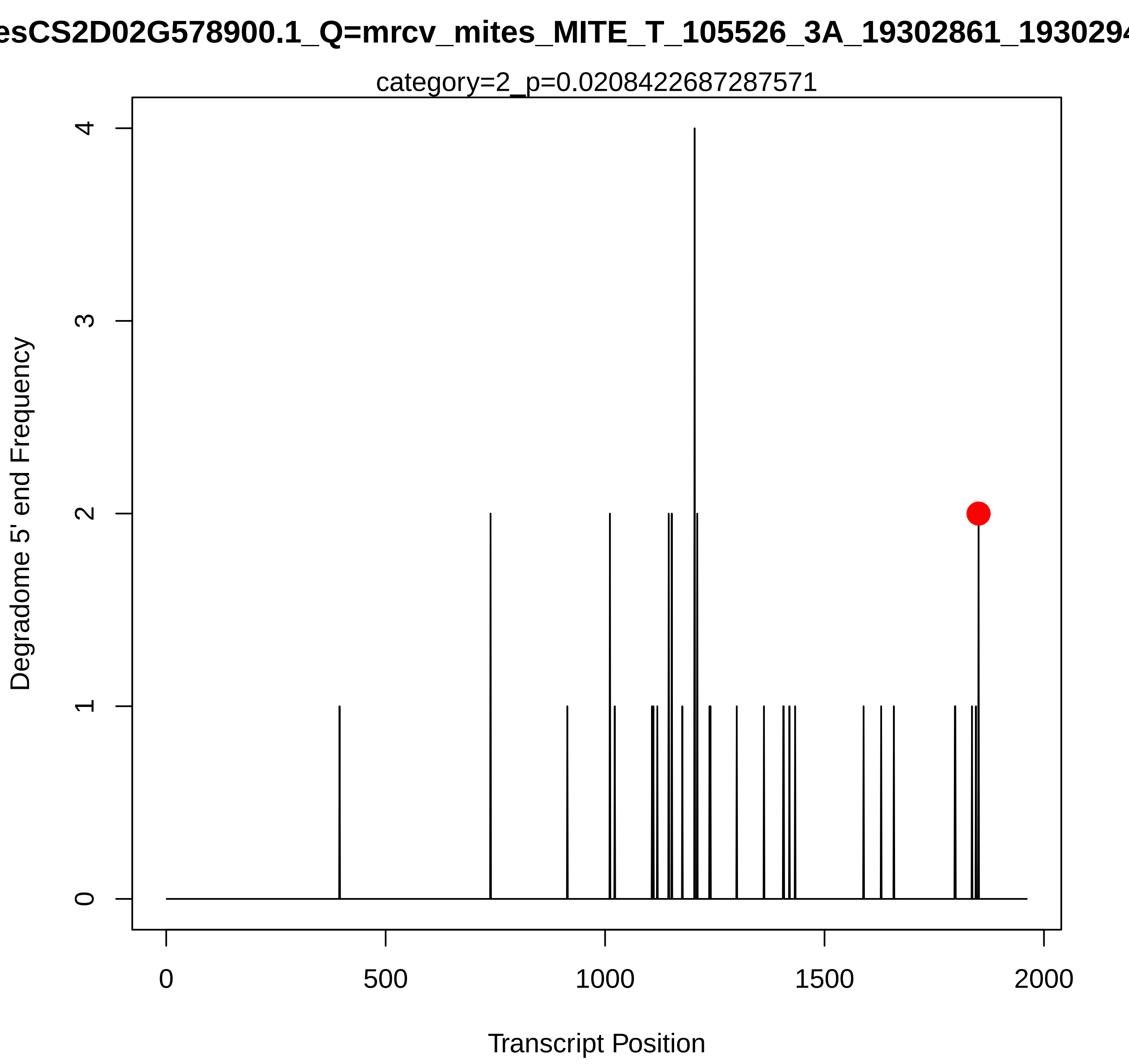

esCS2D02G578900.1_Q=mrcv_mites_MITE_T_105526_3A_19302861_1930294
category=2_p=0.0208422687287571
Degradome 5' end Frequency
0
1
2
3
4
0
500
1000
1500
2000
Transcript Position

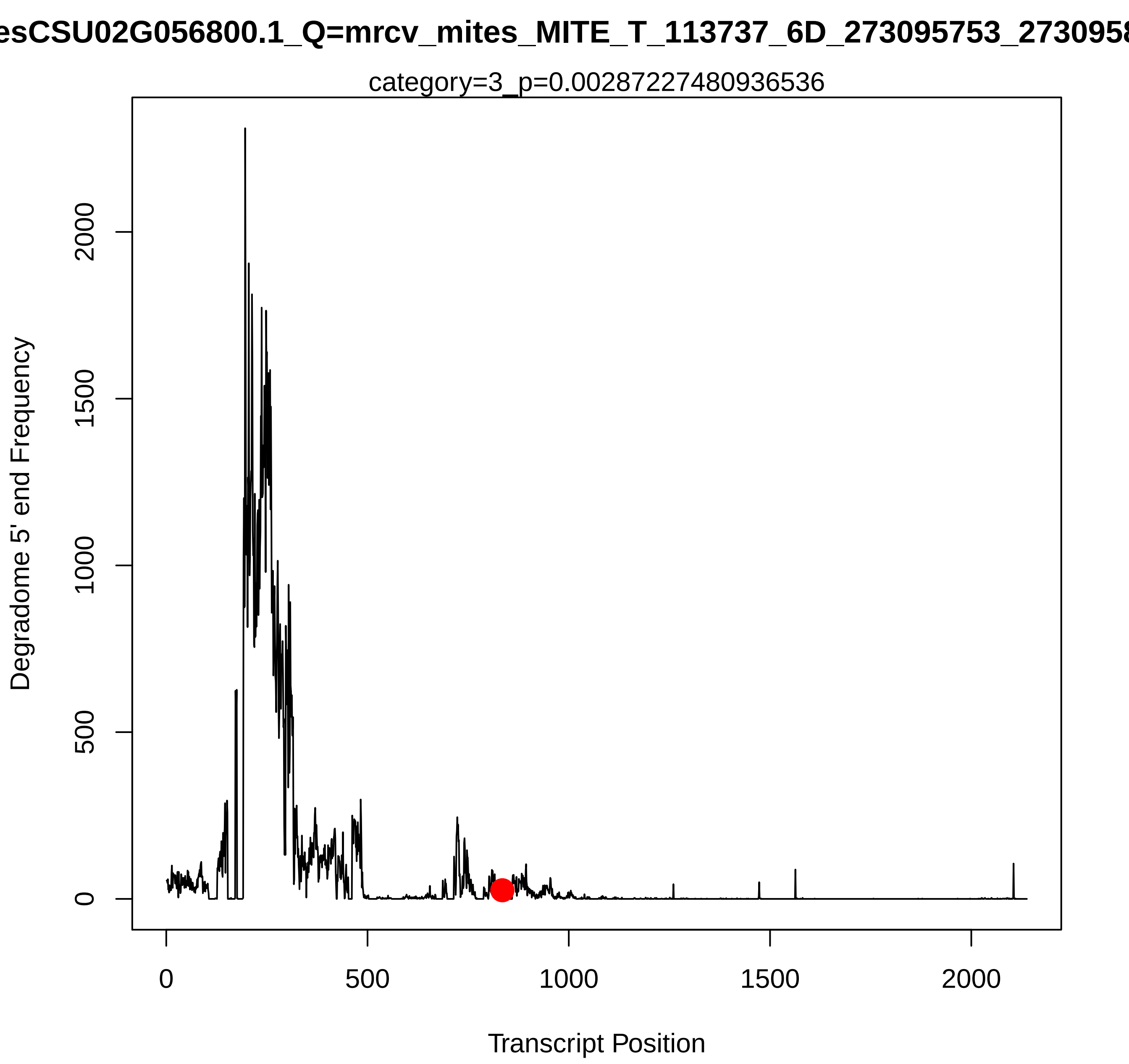
esCSU02G056800.1_Q=mrcv_mites_MITE_T_113737_6D_273095753_2730958
category=3_p=0.00287227480936536
Degradome 5' end Frequency
0
500
1000
1500
2000
Transcript Position
0
500
1000
1500
2000

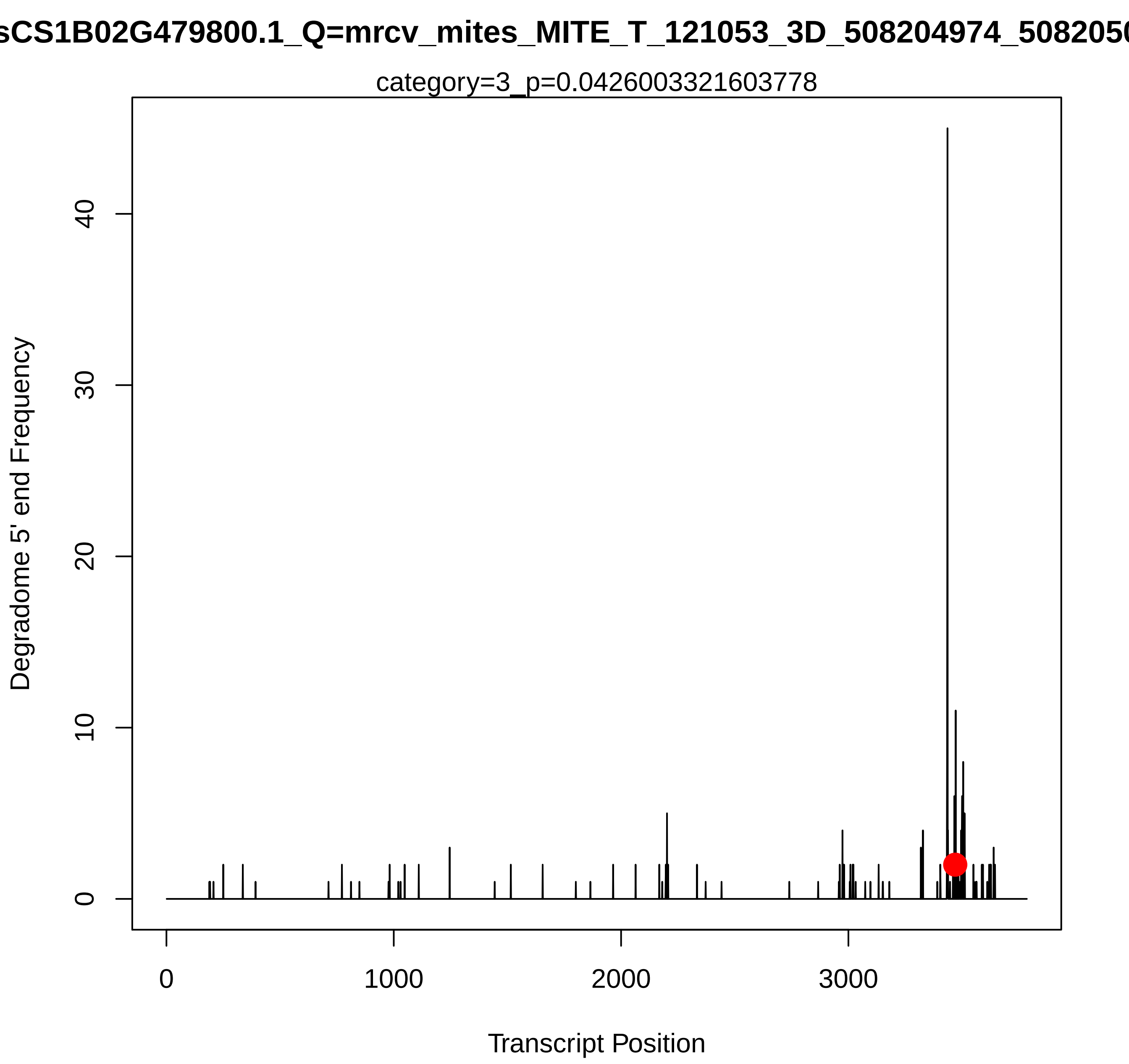
sCS1B02G479800.1_Q=mrcv_mites_MITE_T_121053_3D_508204974_508205
category=3_p=0.0426003321603778
Degradome 5' end Frequency
0
10
20
30
40
0
1000
2000
3000
Transcript Position

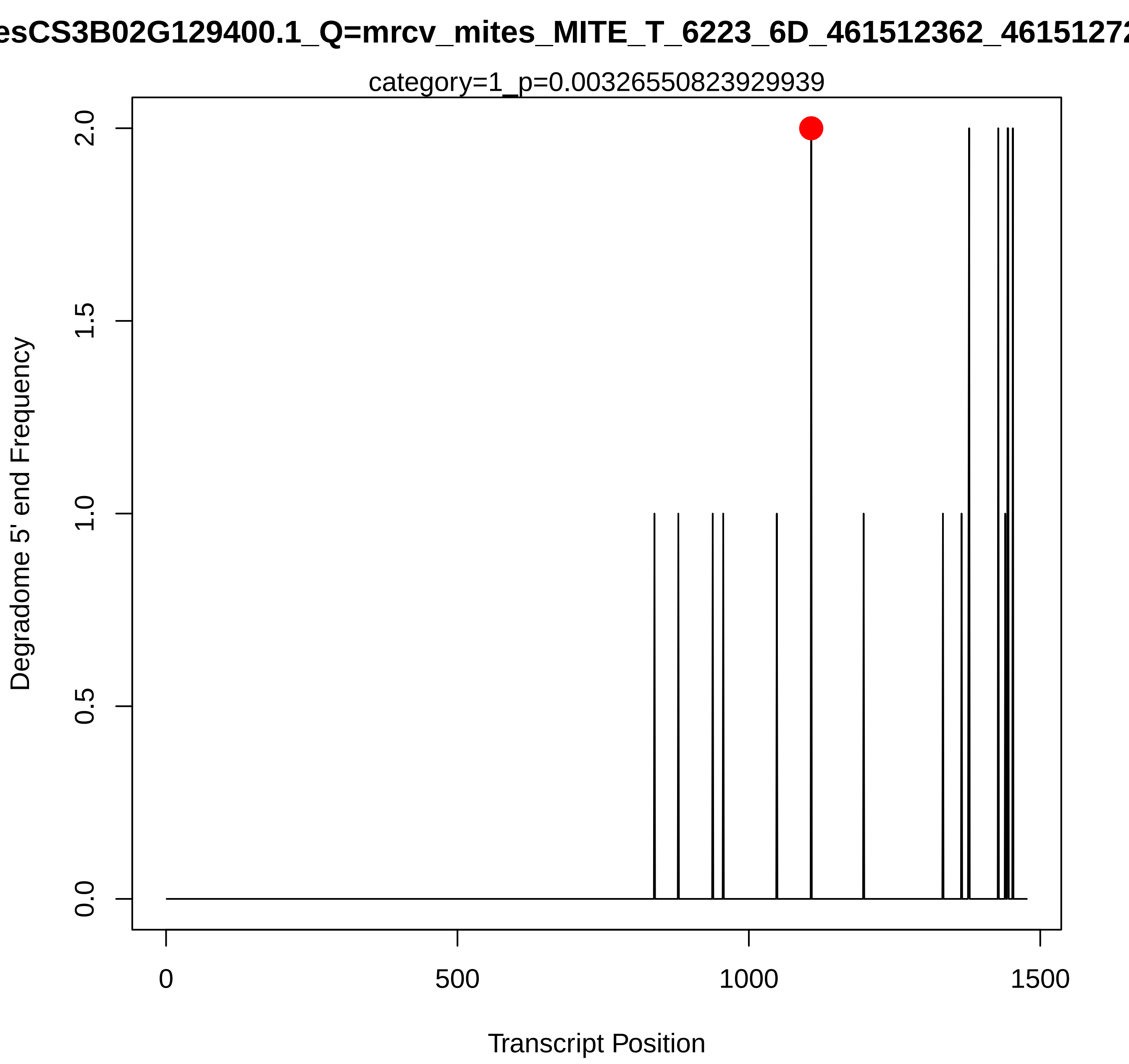
esCS3B02G129400.1_Q=mrcv_mites_MITE_T_6223_6D_461512362_46151272
category=1_p=0.00326550823929939
Degradome 5' end Frequency
0.0
0.5
1.0
1.5
2.0
0
500
1000
1500
Transcript Position

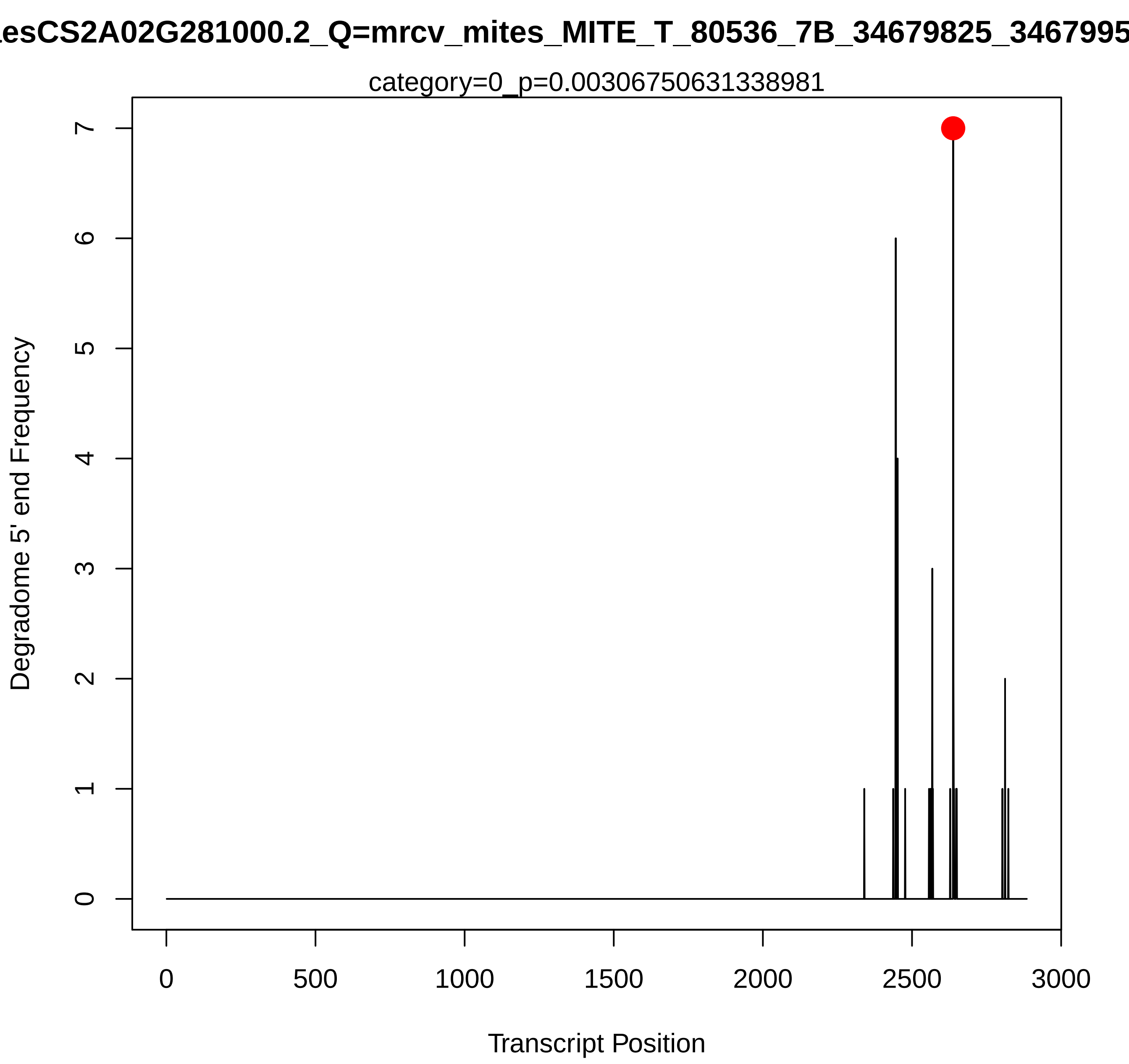

esCS2A02G281000.2_Q=mrcv_mites_MITE_T_80536_7B_34679825_3467995
category=0_p=0.00306750631338981
Degradome 5' end Frequency
0
1
2
3
4
5
6
7
0
500
1000
1500
2000
2500
3000
Transcript Position

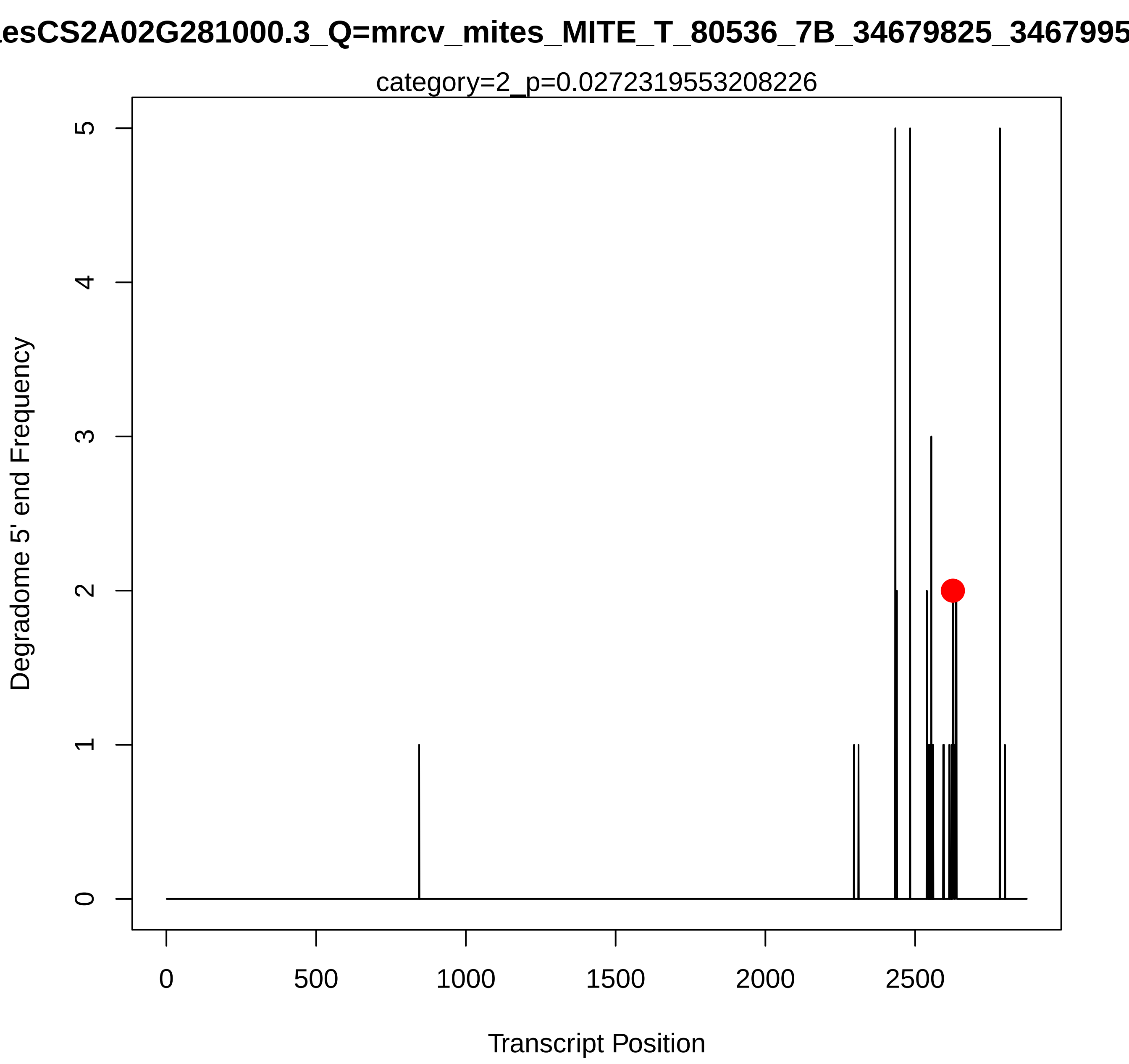

TraesCS2A02G281000.3_Q=mrcv_mites_MITE_T_80536_7B_34679825_3467995
category=2_p=0.0272319553208226
Degradome 5' end Frequency
0
1
2
3
4
5
0
500
1000
1500
2000
2500
Transcript Position

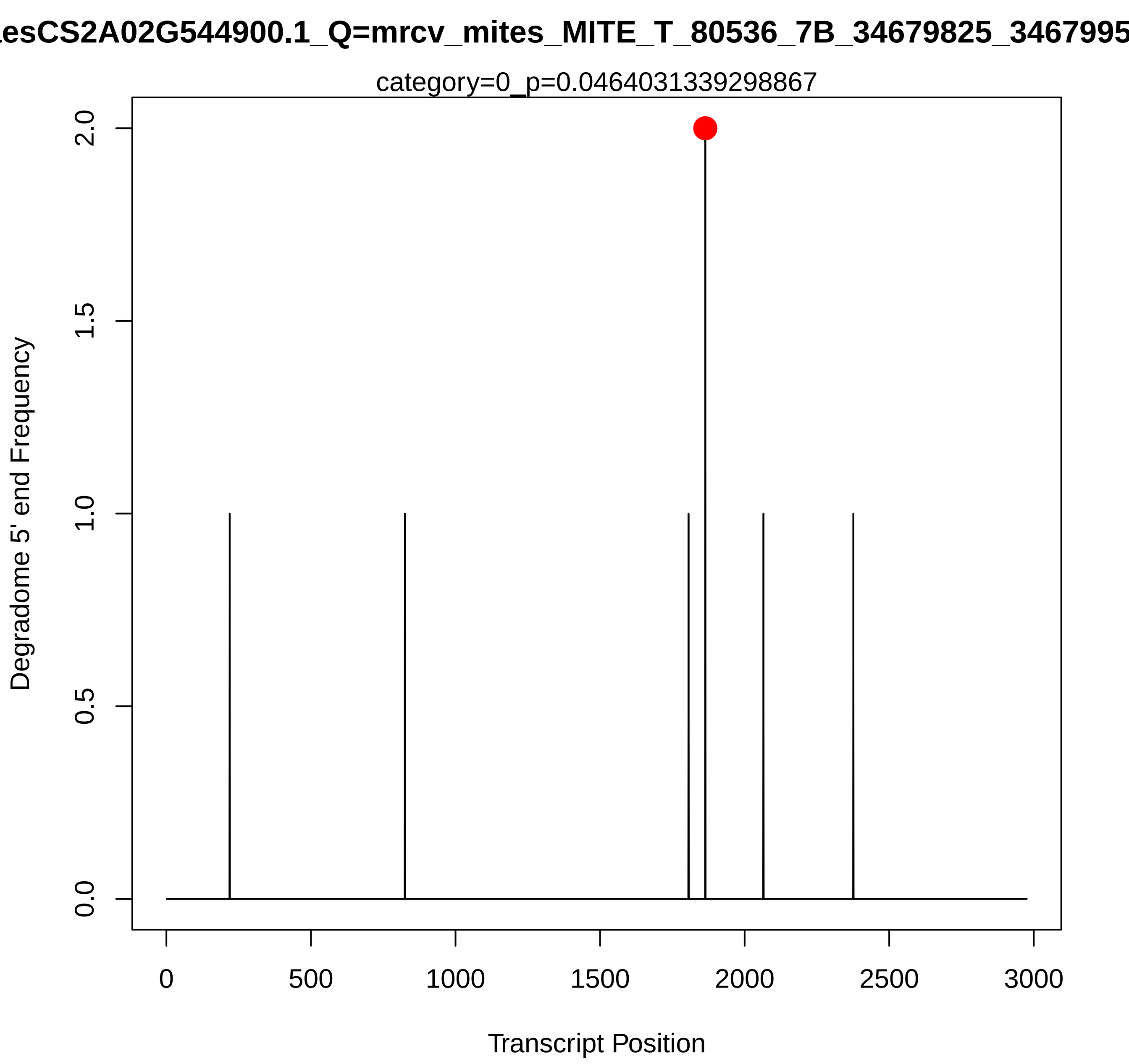

[illegible]esCS2A02G544900.1_Q=mrcv_mites_MITE_T_80536_7B_34679825_3467995[illegible]
category=0_p=0.0464031339298867
Degradome 5' end Frequency
0.0
0.5
1.0
1.5
2.0
0
500
1000
1500
2000
2500
3000
Transcript Position

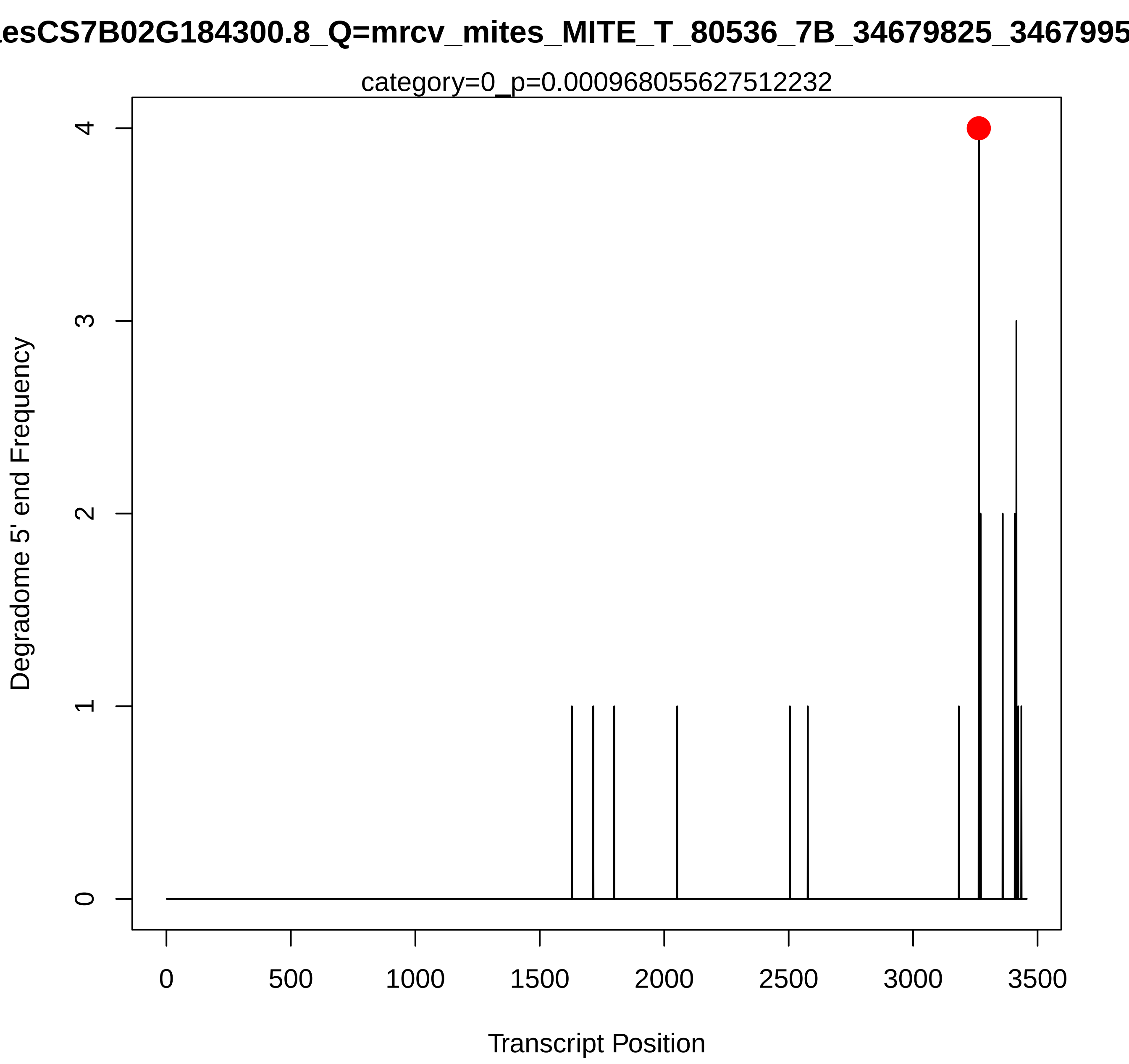

esCS7B02G184300.8_Q=mrcv_mites_MITE_T_80536_7B_34679825_3467995
category=0_p=0.000968055627512232
Degradome 5' end Frequency
0
1
2
3
4
0
500
1000
1500
2000
2500
3000
3500
Transcript Position

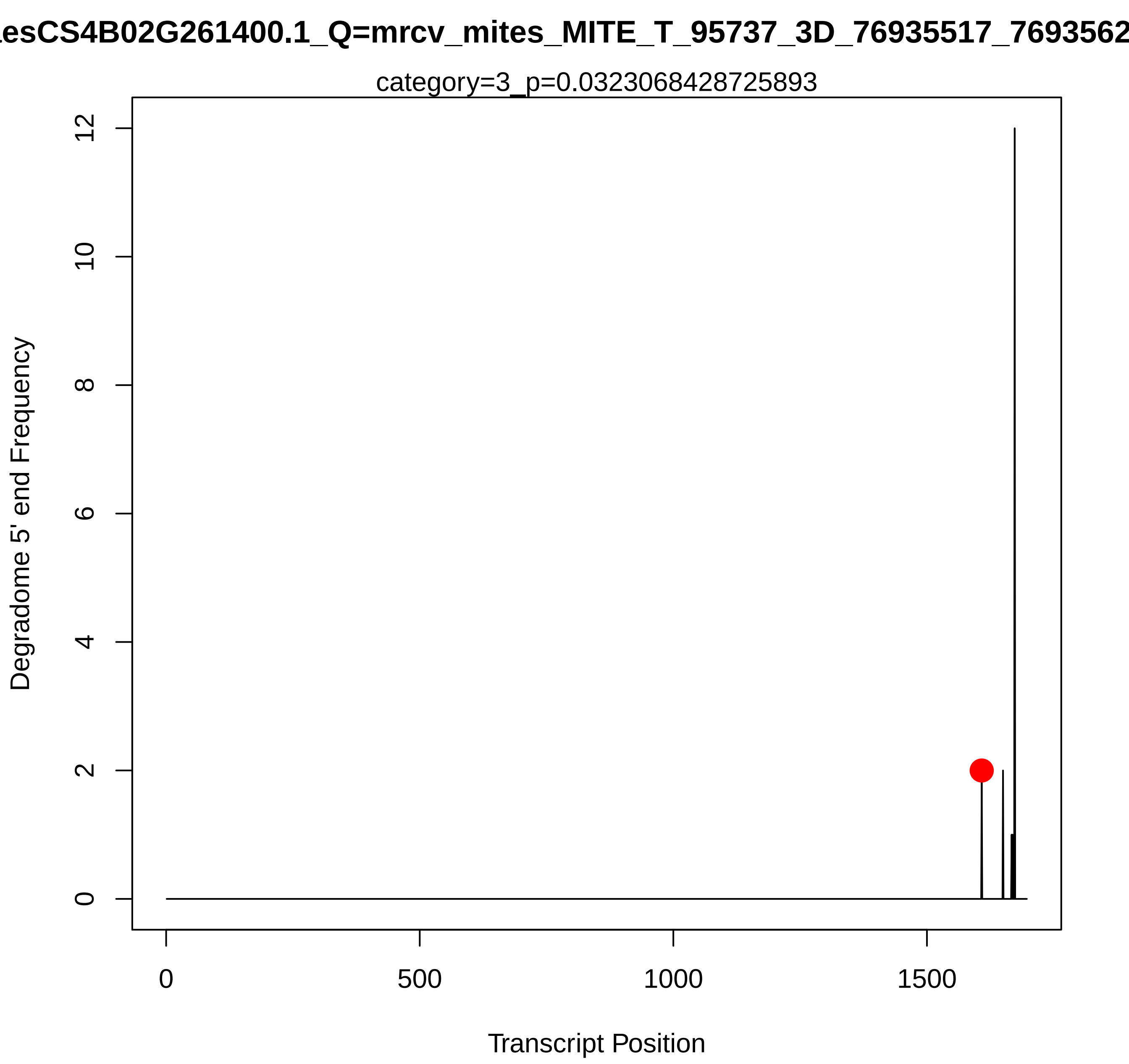

.esCS4B02G261400.1_Q=mrcv_mites_MITE_T_95737_3D_76935517_7693562
category=3_p=0.0323068428725893
Degradome 5' end Frequency
0
2
4
6
8
10
12
0
500
1000
1500
Transcript Position

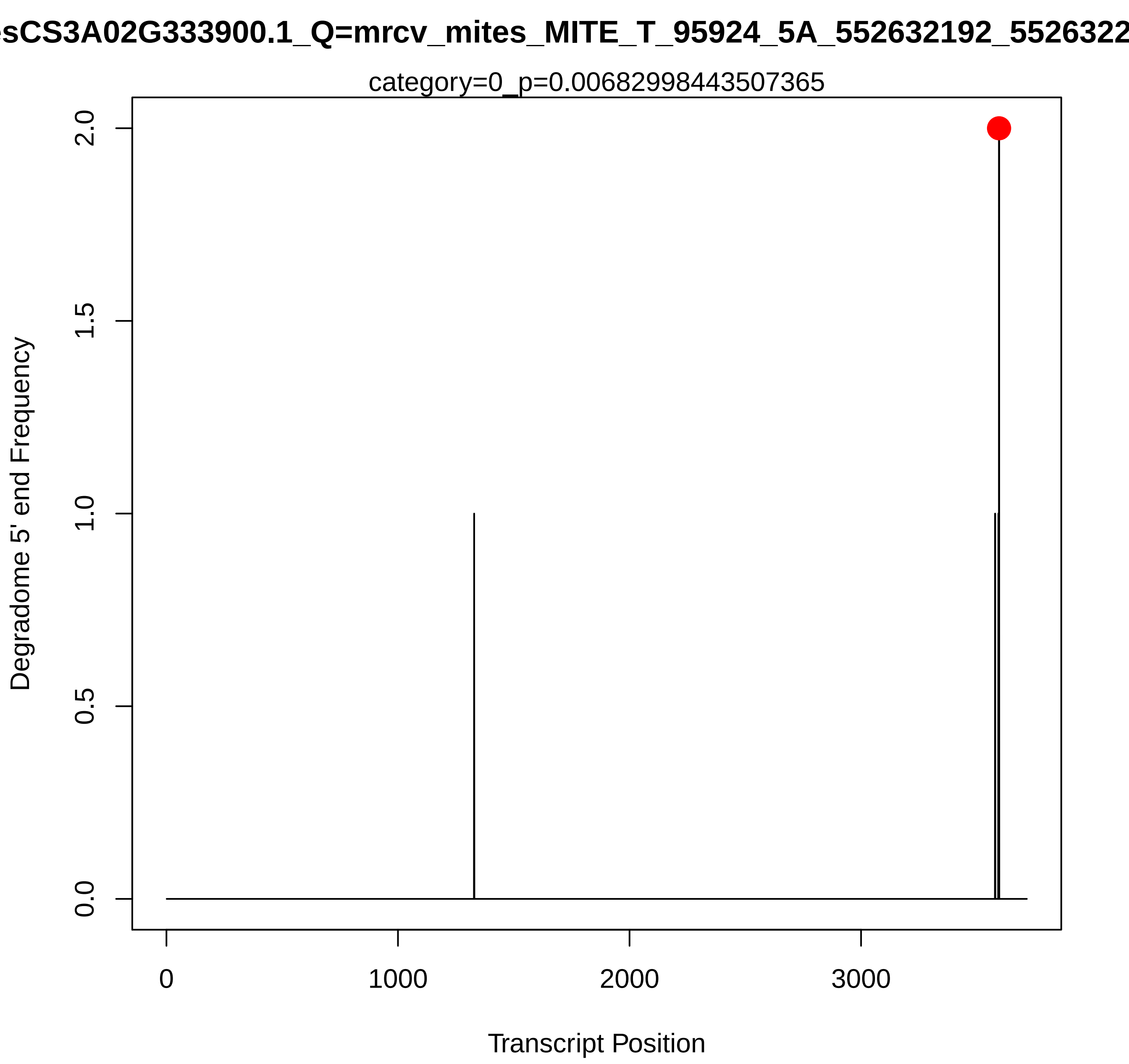
esCS3A02G333900.1_Q=mrcv_mites_MITE_T_95924_5A_552632192_5526322
category=0_p=0.00682998443507365
Degradome 5' end Frequency
Transcript Position
0.0
0.5
1.0
1.5
2.0
0
1000
2000
3000

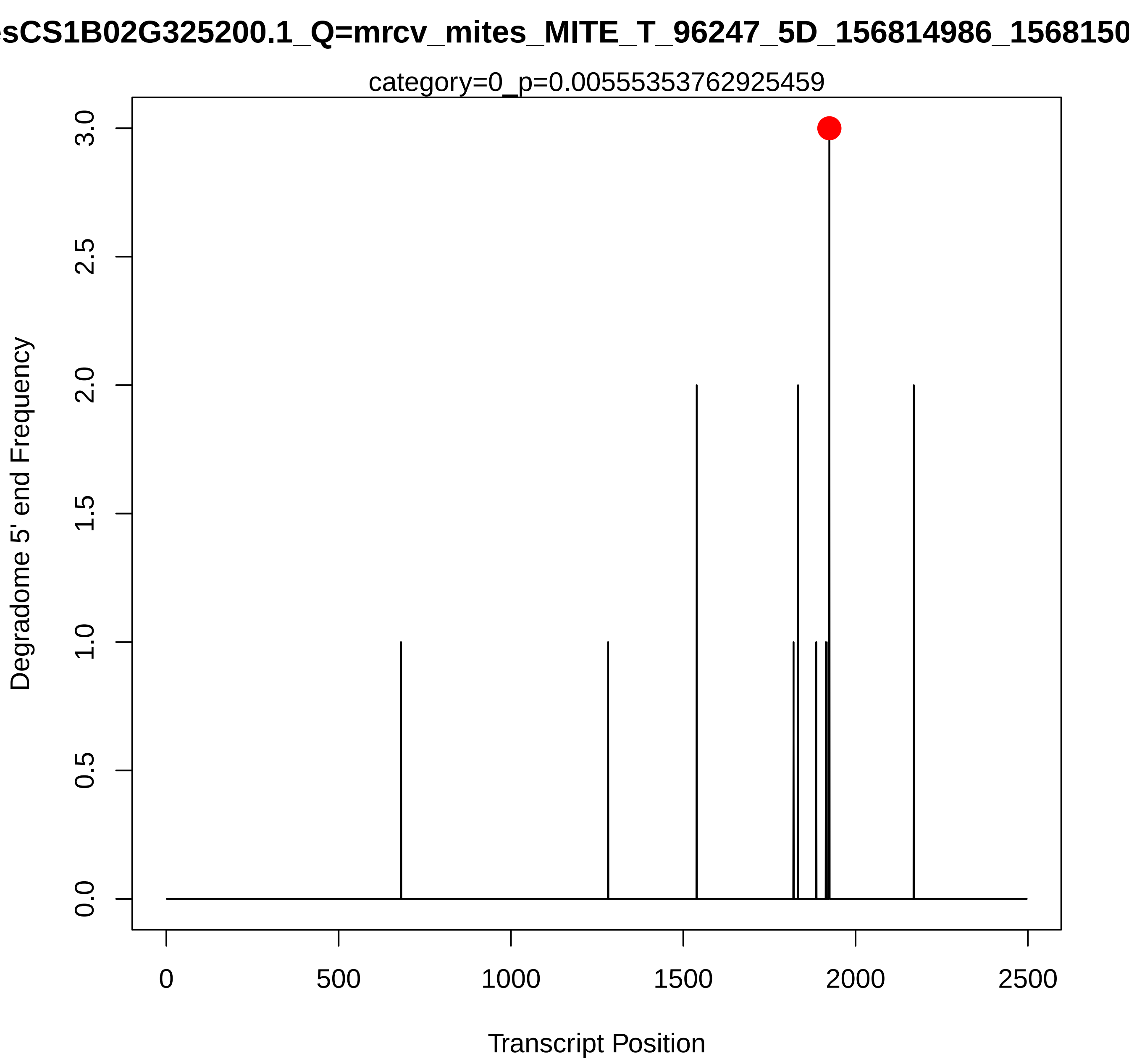

esCS1B02G325200.1_Q=mrcv_mites_MITE_T_96247_5D_156814986_1568150
category=0_p=0.00555353762925459
Degradome 5' end Frequency
0.0
0.5
1.0
1.5
2.0
2.5
3.0
0
500
1000
1500
2000
2500
Transcript Position

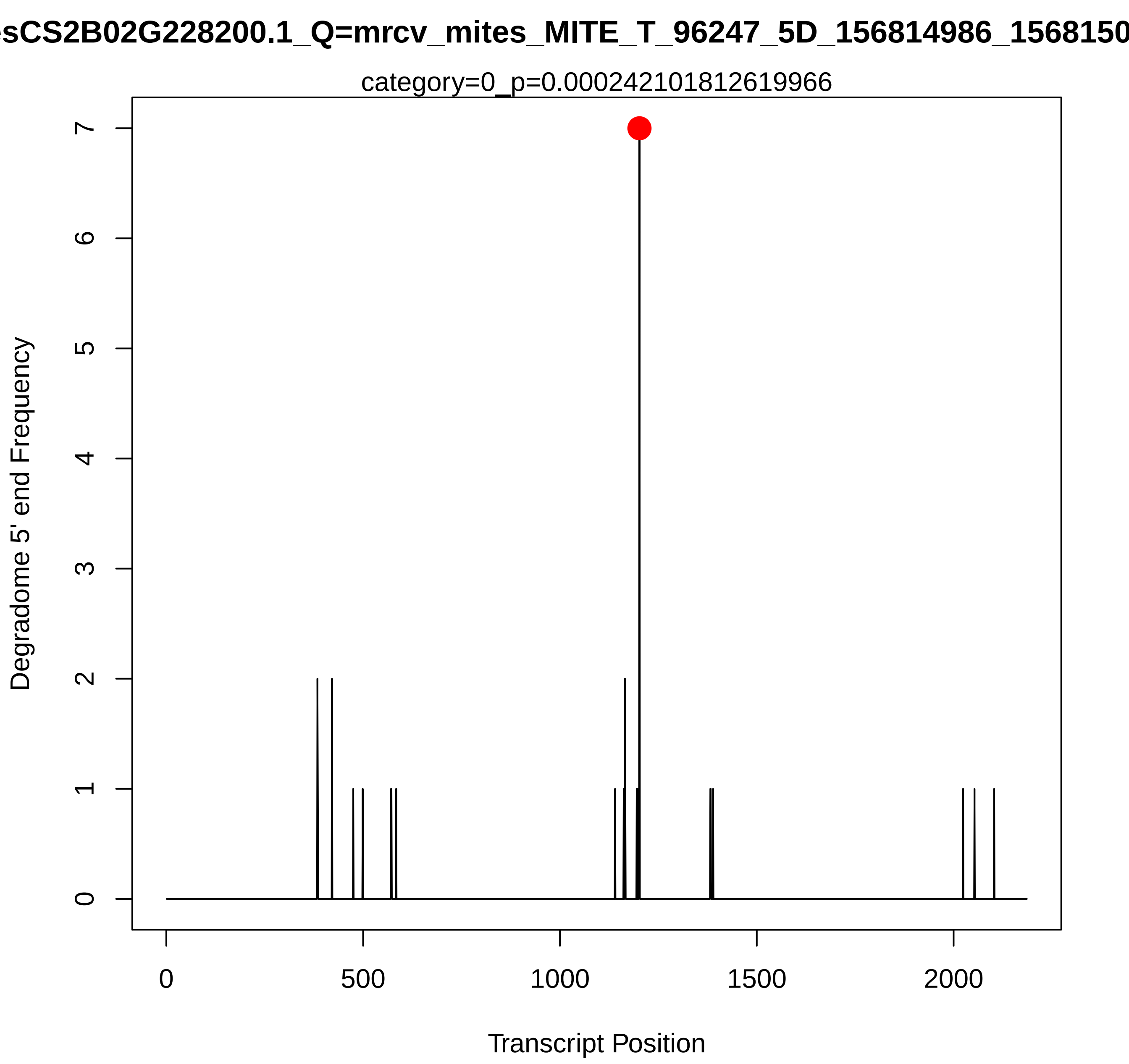

esCS2B02G228200.1_Q=mrcv_mites_MITE_T_96247_5D_156814986_1568150
category=0_p=0.000242101812619966
Degradome 5' end Frequency
Transcript Position
0
1
2
3
4
5
6
7
0
500
1000
1500
2000

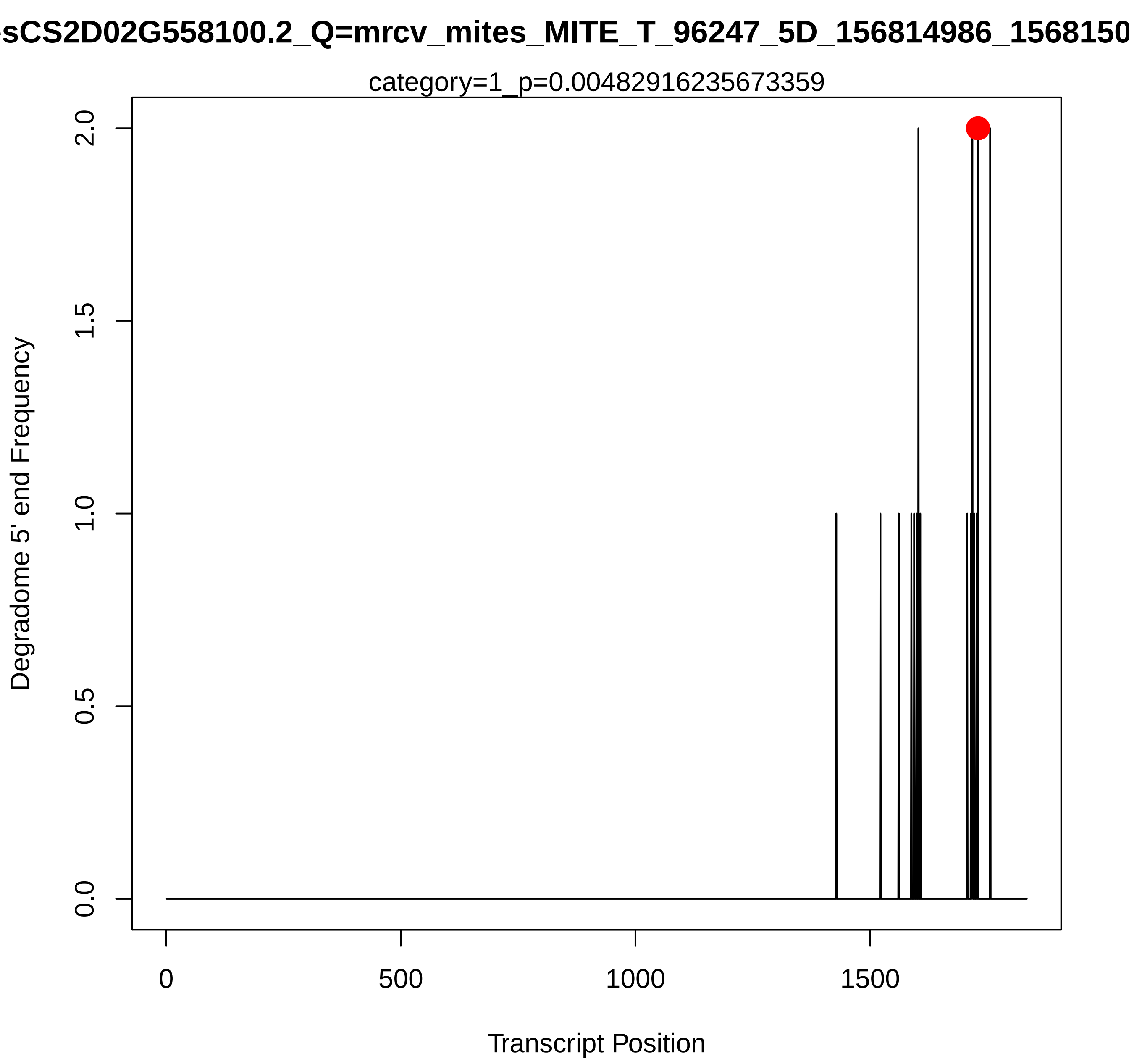

esCS2D02G558100.2_Q=mrcv_mites_MITE_T_96247_5D_156814986_1568150
category=1_p=0.00482916235673359
Degradome 5' end Frequency
Transcript Position
0.0
0.5
1.0
1.5
2.0
0
500
1000
1500

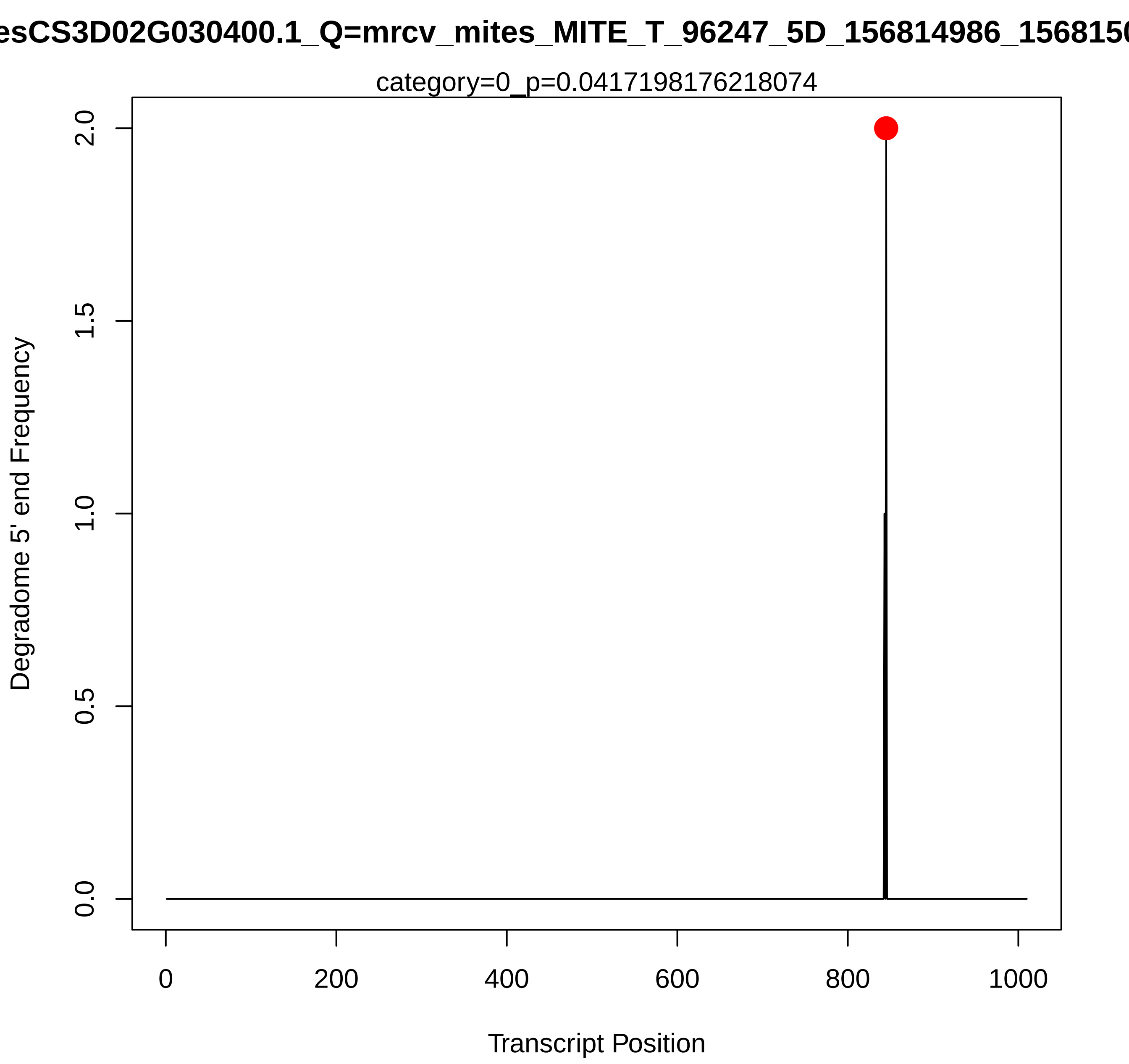

esCS3D02G030400.1_Q=mrcv_mites_MITE_T_96247_5D_156814986_1568150
category=0_p=0.0417198176218074
Degradome 5' end Frequency
0.0
0.5
1.0
1.5
2.0
0
200
400
600
800
1000
Transcript Position

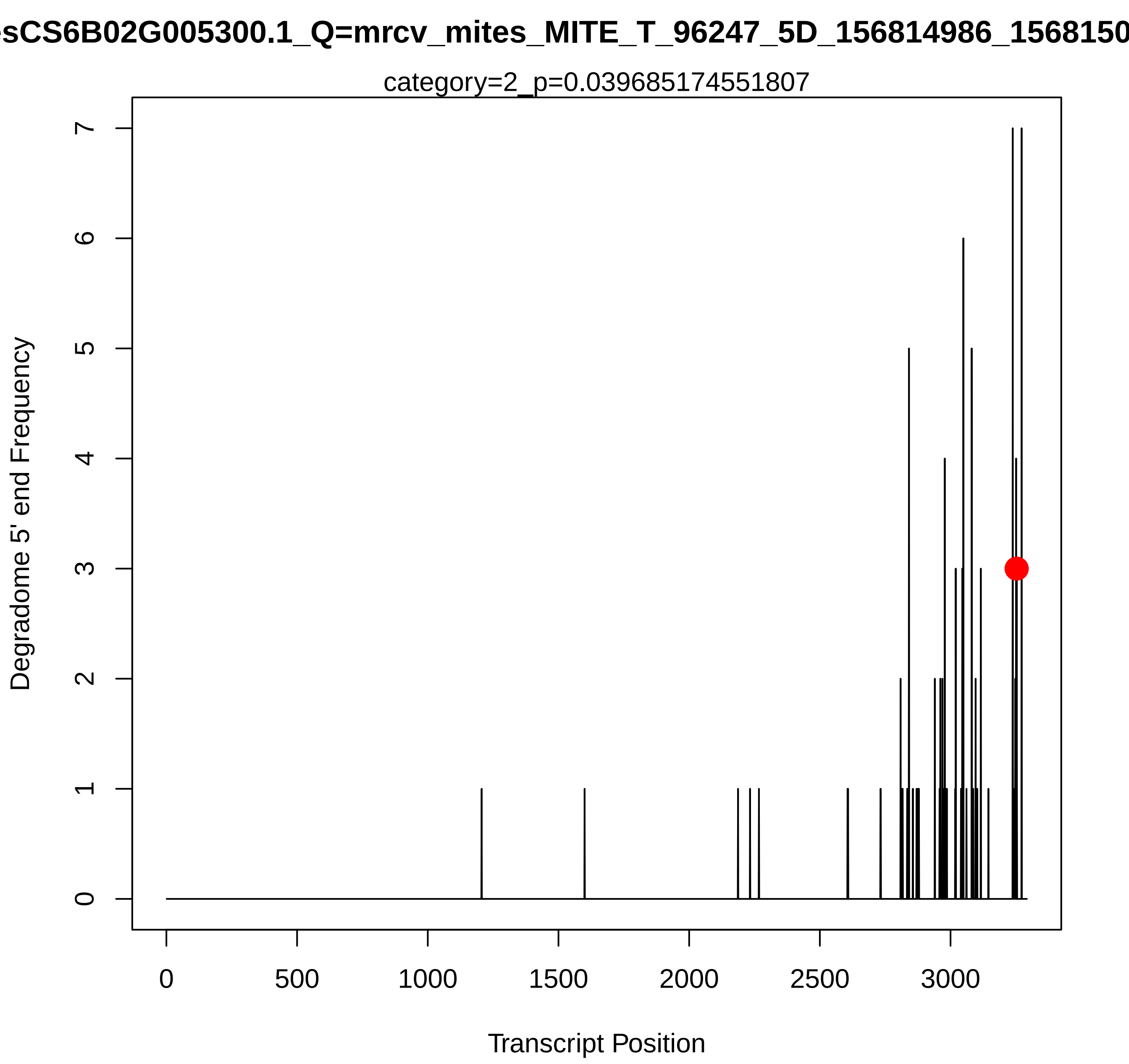

esCS6B02G005300.1_Q=mrcv_mites_MITE_T_96247_5D_156814986_1568150
category=2_p=0.039685174551807
Degradome 5' end Frequency
0
1
2
3
4
5
6
7
0
500
1000
1500
2000
2500
3000
Transcript Position

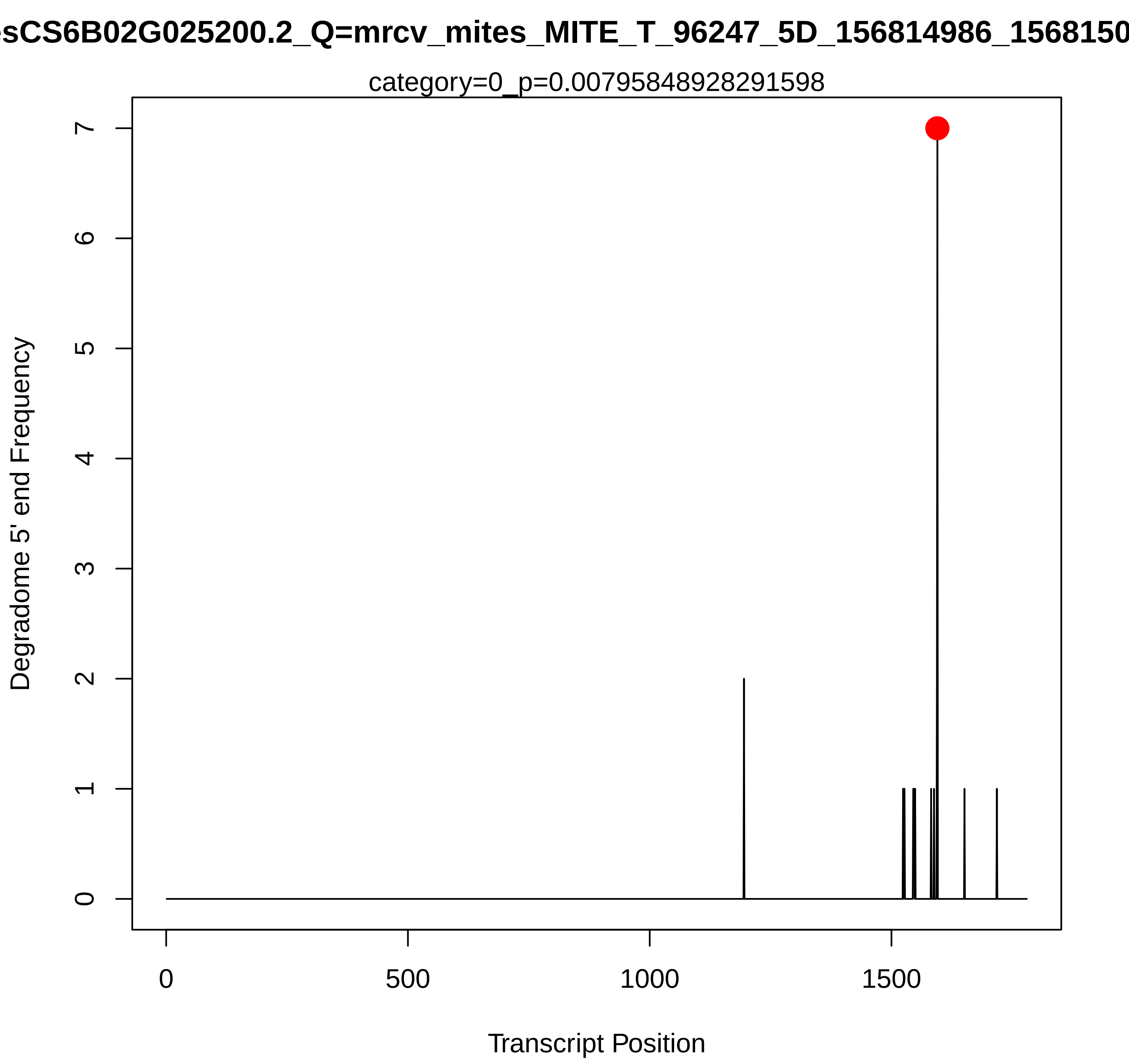

esCS6B02G025200.2_Q=mrcv_mites_MITE_T_96247_5D_156814986_156815
category=0_p=0.00795848928291598
Degradome 5' end Frequency
0
1
2
3
4
5
6
7
0
500
1000
1500
Transcript Position

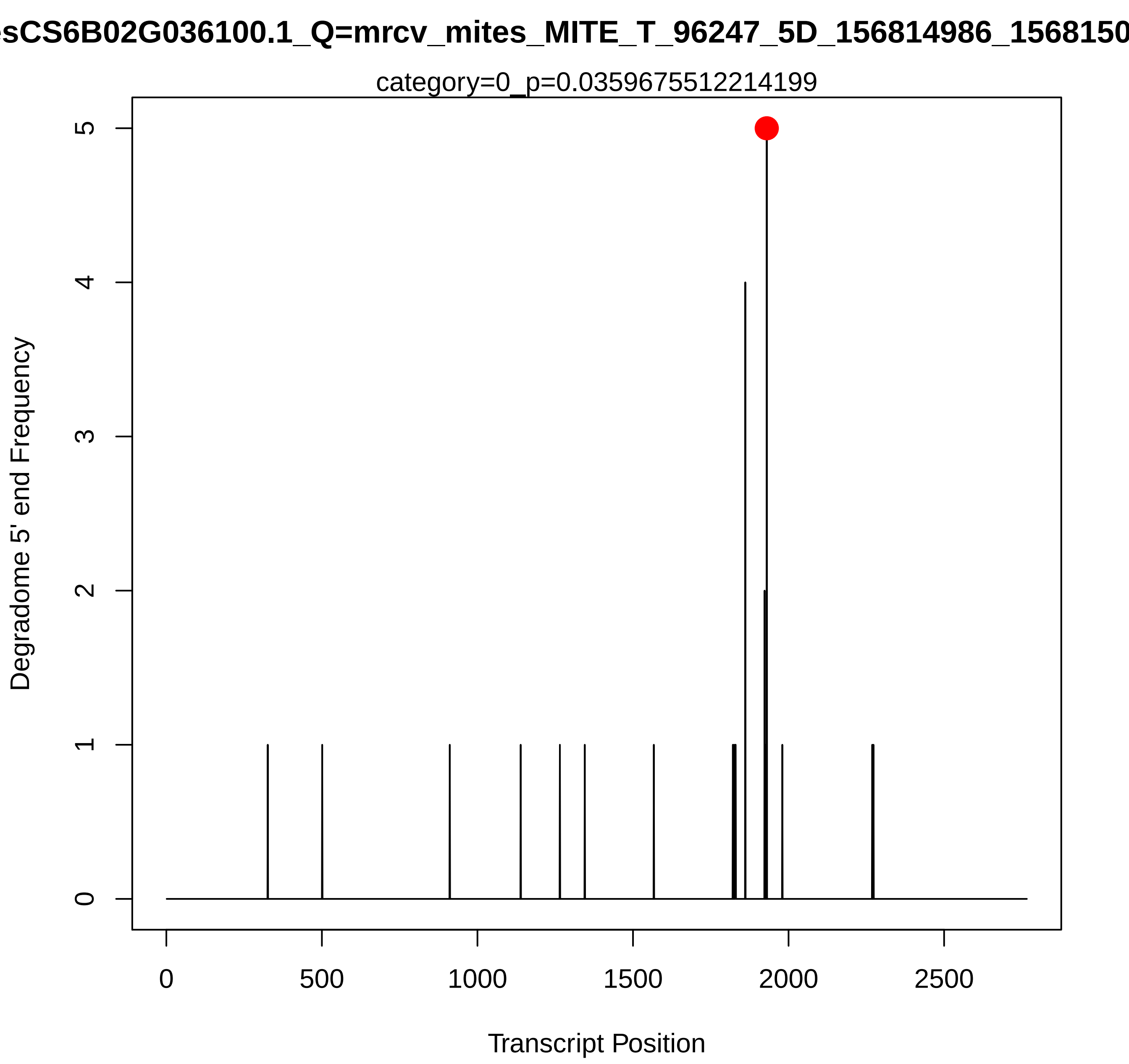

esCS6B02G036100.1_Q=mrcv_mites_MITE_T_96247_5D_156814986_1568150
category=0_p=0.0359675512214199
Degradome 5' end Frequency
Transcript Position
0
1
2
3
4
5
0
500
1000
1500
2000
2500

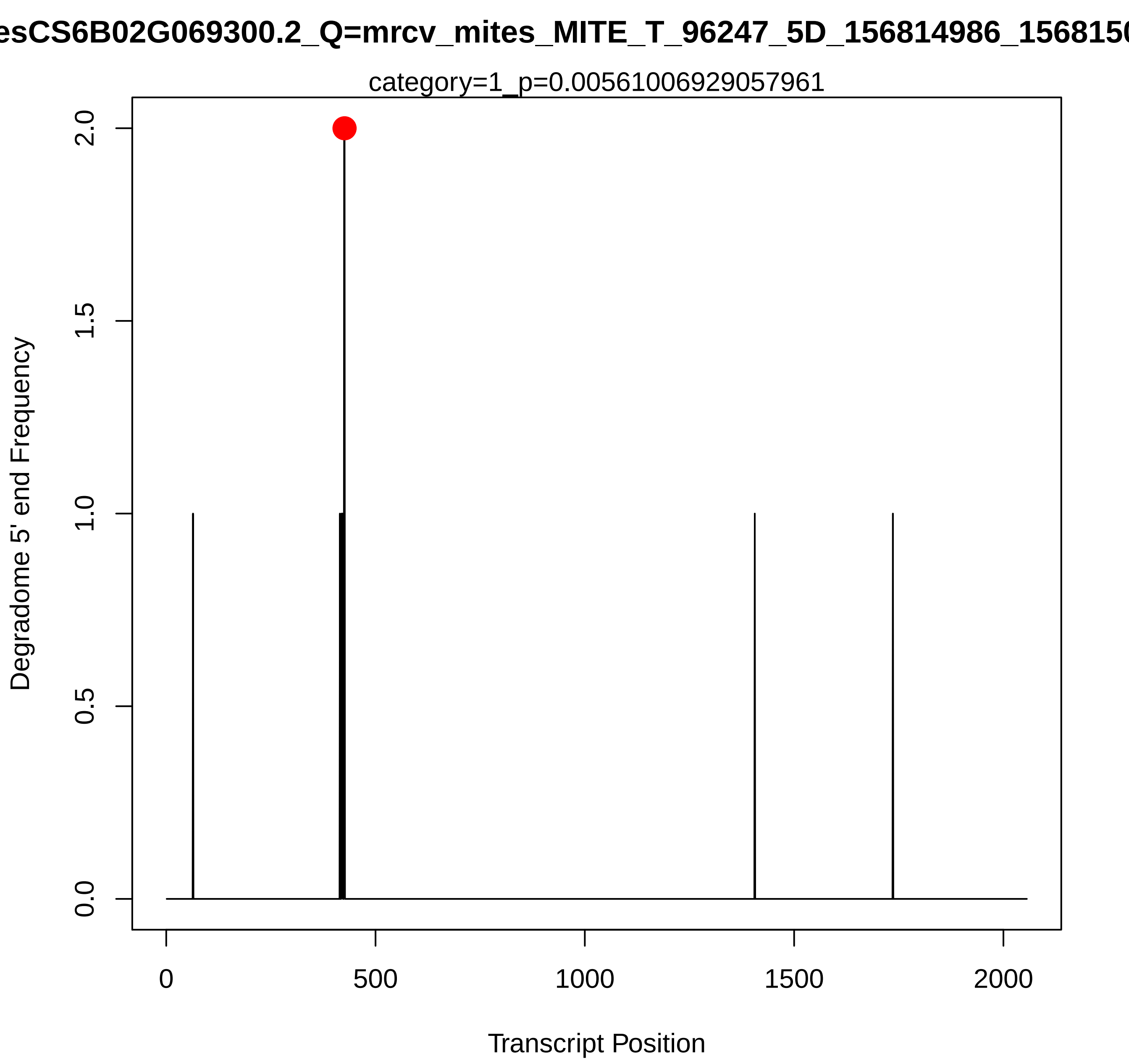
esCS6B02G069300.2_Q=mrcv_mites_MITE_T_96247_5D_156814986_156815
category=1_p=0.00561006929057961
Degradome 5' end Frequency
Transcript Position
0.0
0.5
1.0
1.5
2.0
0
500
1000
1500
2000

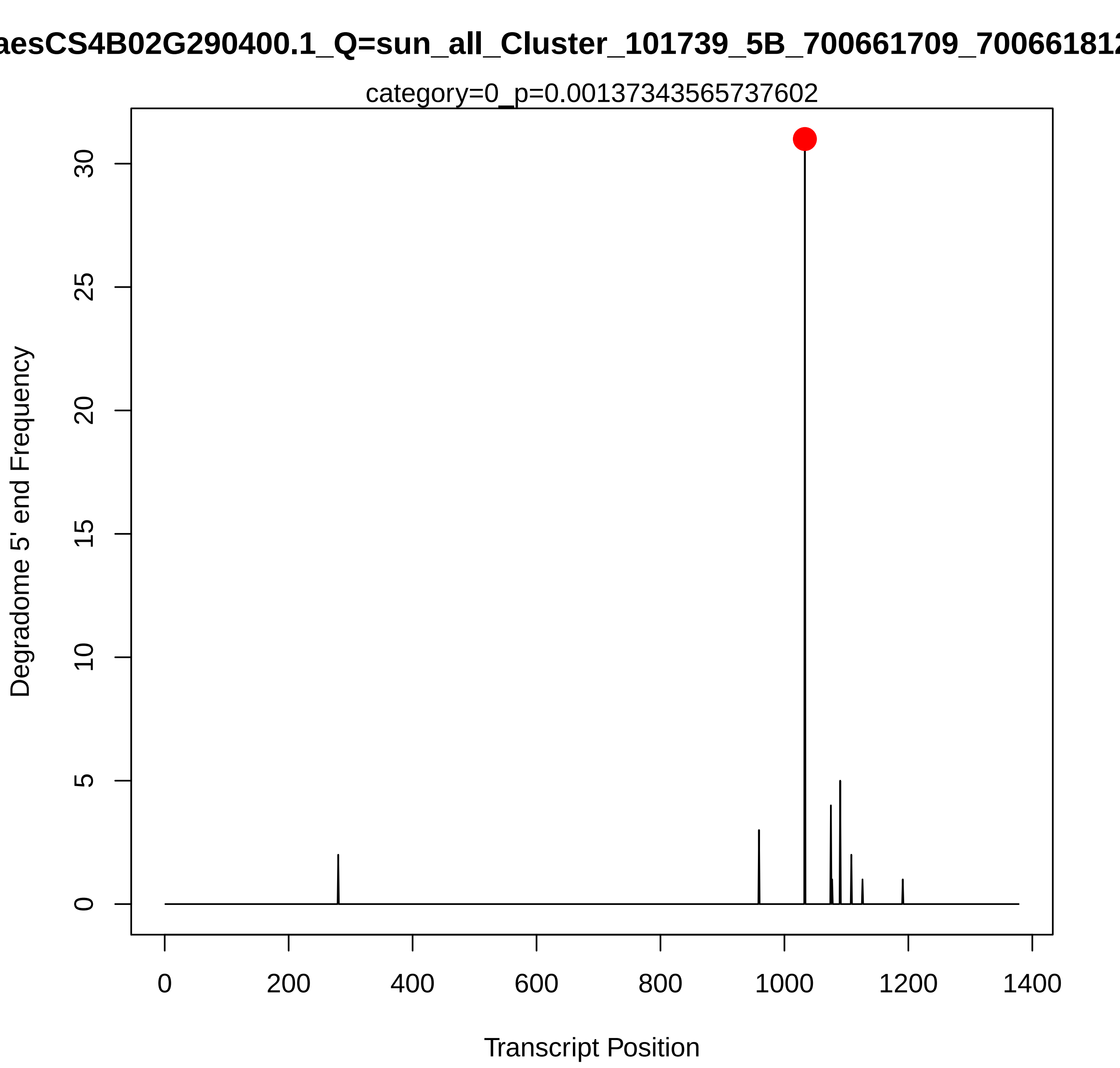

aesCS4B02G290400.1_Q=sun_all_Cluster_101739_5B_700661709_70066181
category=0_p=0.00137343565737602
Degradome 5' end Frequency
0
5
10
15
20
25
30
0
200
400
600
800
1000
1200
1400
Transcript Position

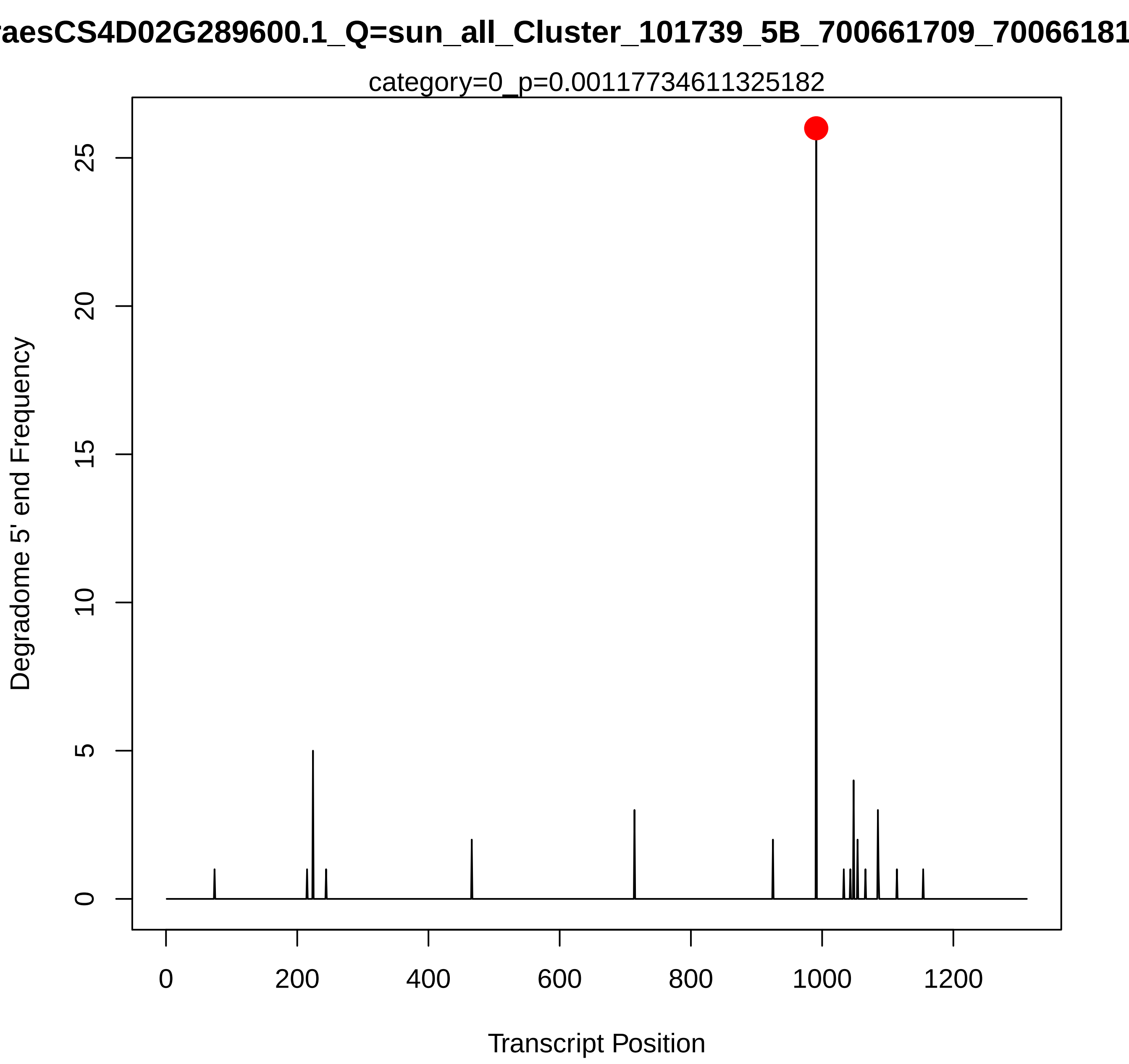

aesCS4D02G289600.1_Q=sun_all_Cluster_101739_5B_700661709_70066181
category=0_p=0.00117734611325182
Degradome 5' end Frequency
0
5
10
15
20
25
0
200
400
600
800
1000
1200
Transcript Position

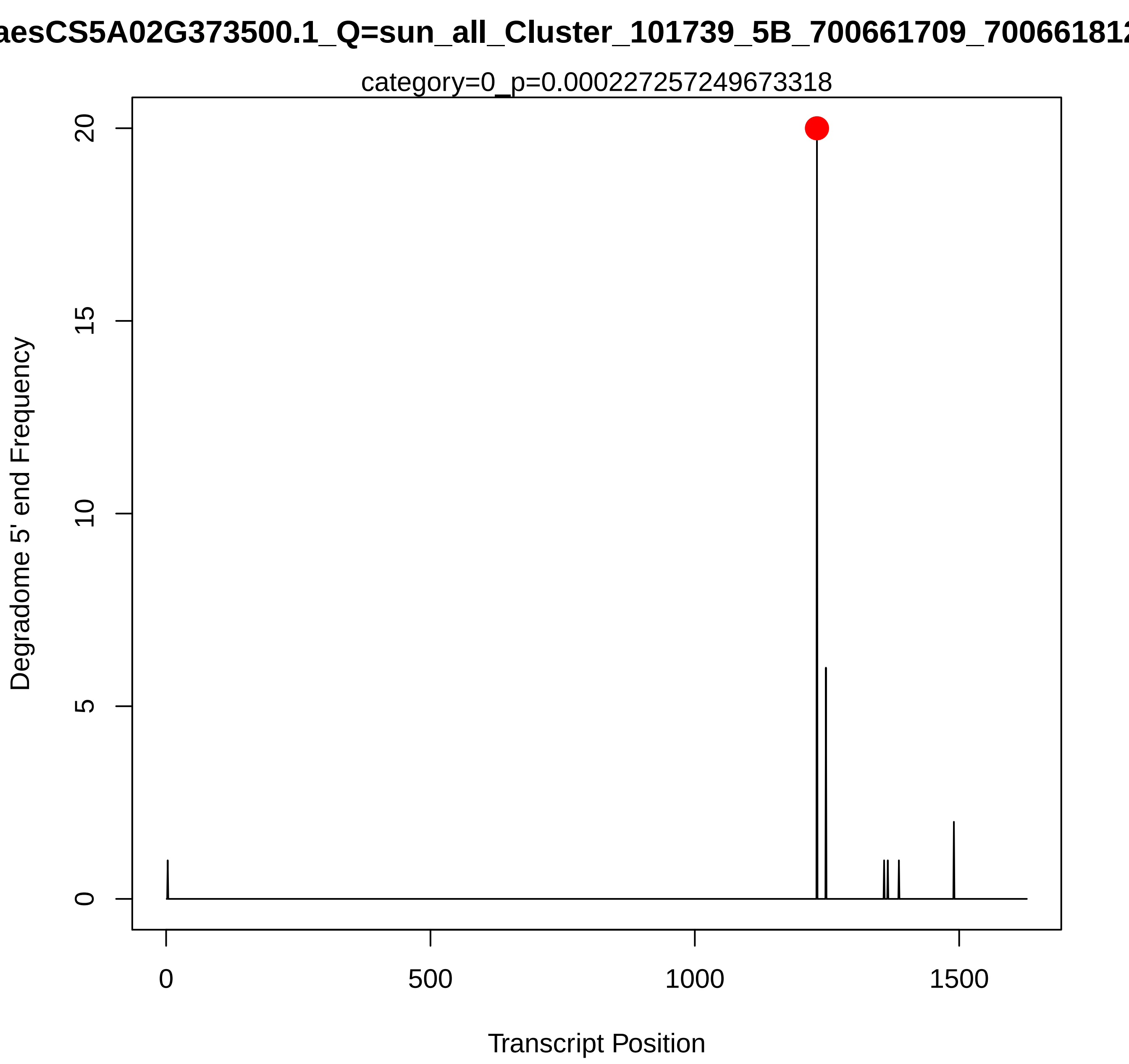
aesCS5A02G373500.1_Q=sun_all_Cluster_101739_5B_700661709_70066181
category=0_p=0.000227257249673318
Degradome 5' end Frequency
0
5
10
15
20
0
500
1000
1500
Transcript Position

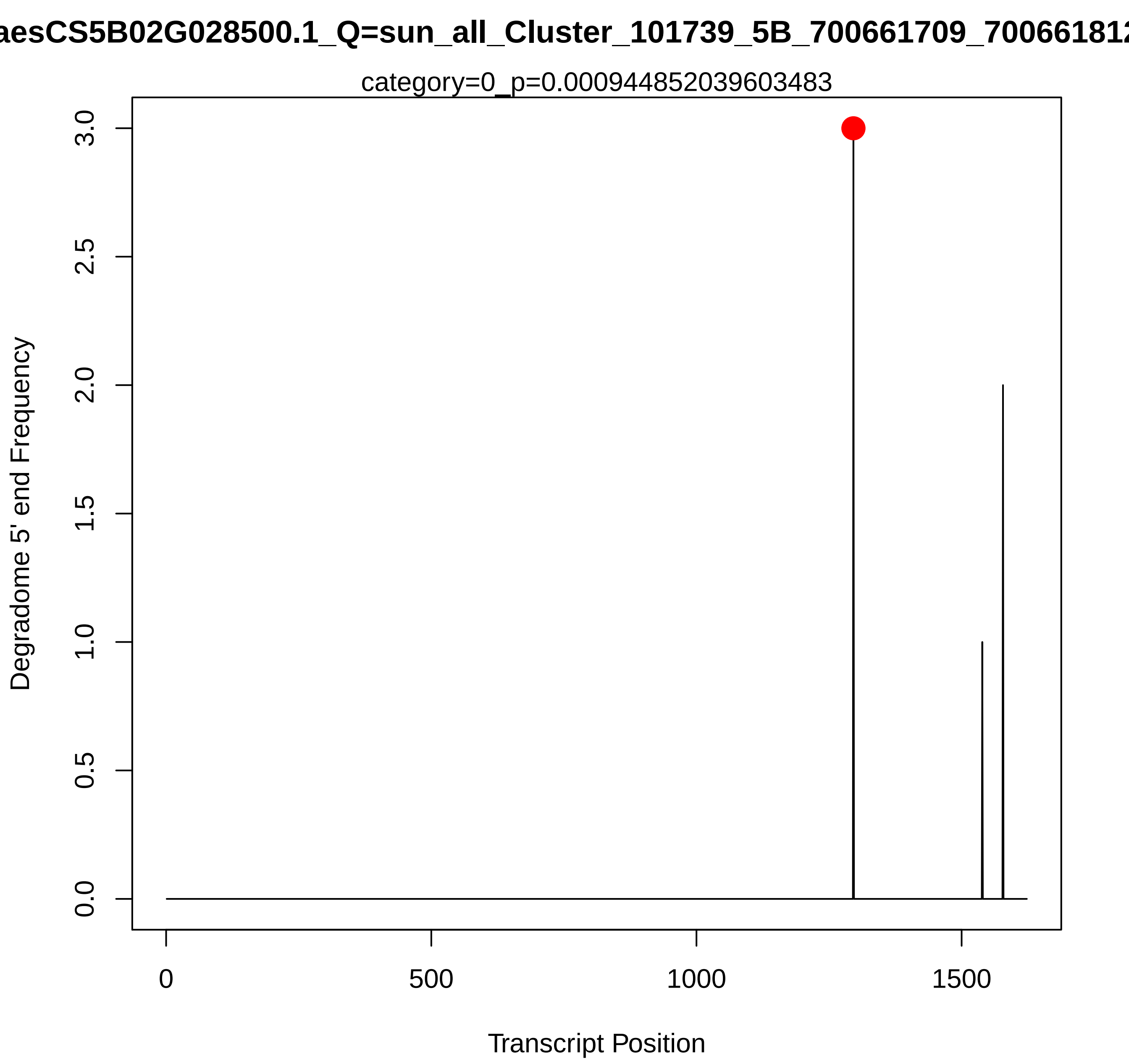

aesCS5B02G028500.1_Q=sun_all_Cluster_101739_5B_700661709_70066181
category=0_p=0.000944852039603483
Degradome 5' end Frequency
Transcript Position
0.0
0.5
1.0
1.5
2.0
2.5
3.0
0
500
1000
1500

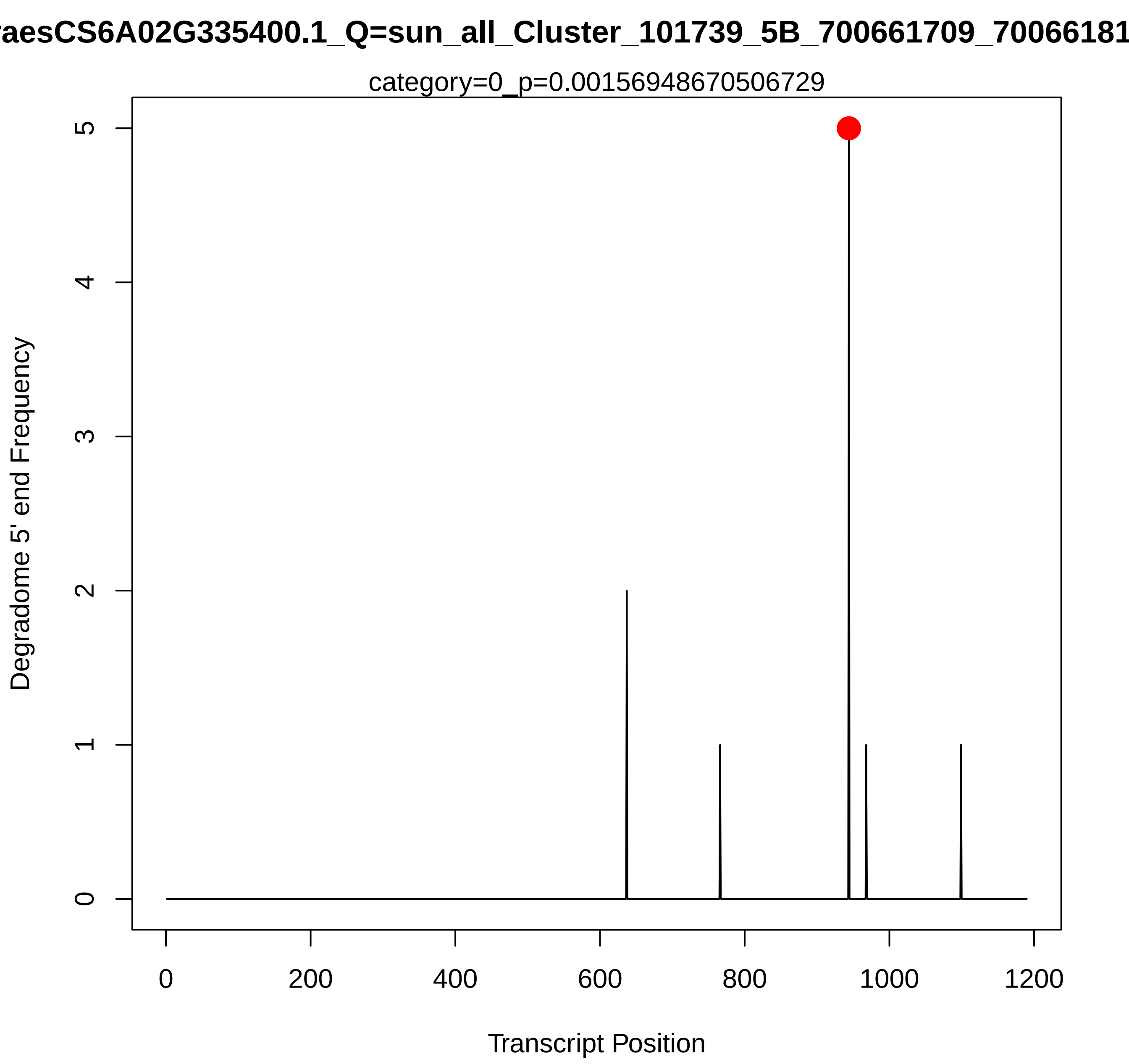

aesCS6A02G335400.1_Q=sun_all_Cluster_101739_5B_700661709_70066181
category=0_p=0.00156948670506729
Degradome 5' end Frequency
0
1
2
3
4
5
0
200
400
600
800
1000
1200
Transcript Position

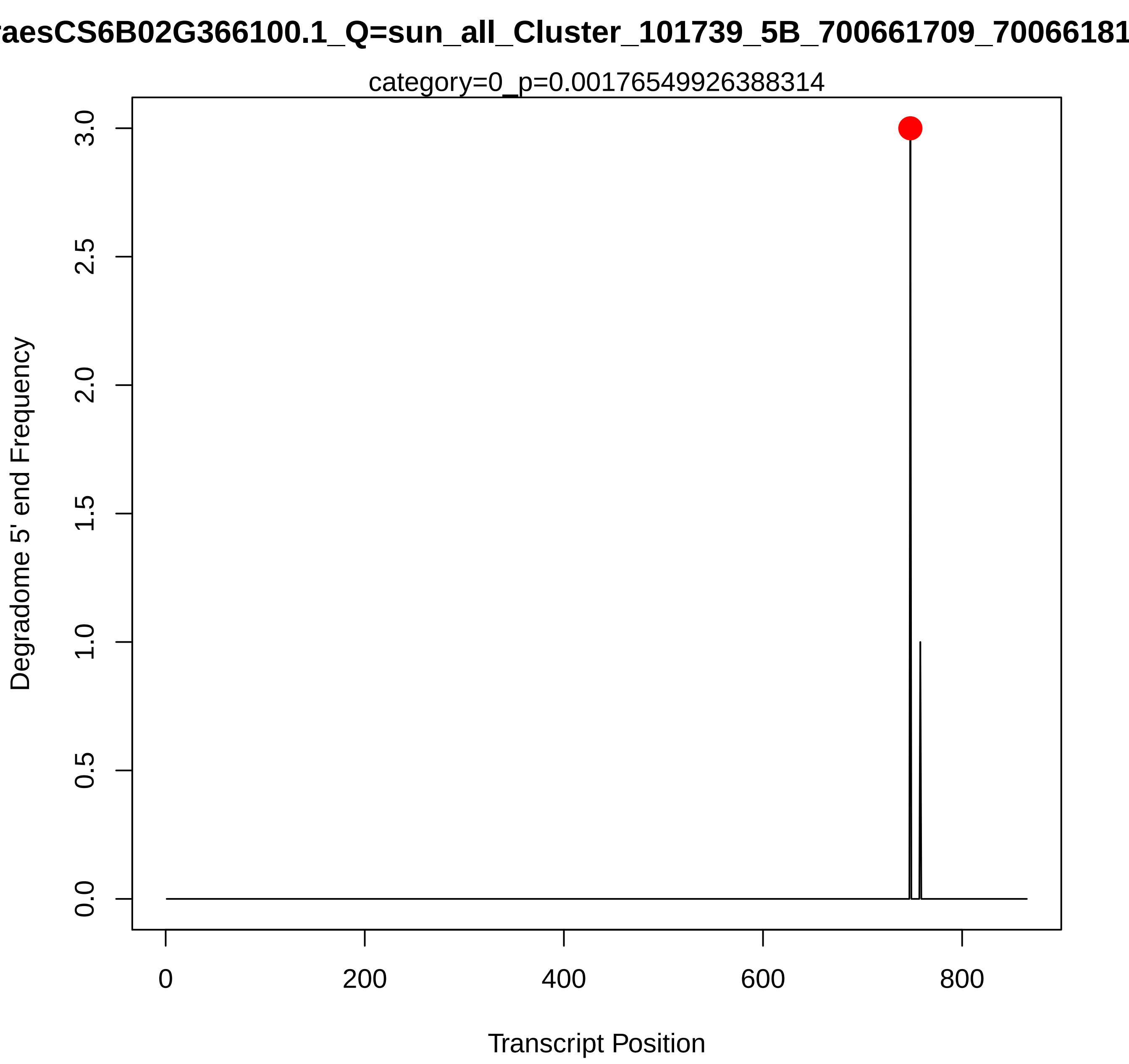

raesCS6B02G366100.1_Q=sun_all_Cluster_101739_5B_700661709_70066181
category=0_p=0.00176549926388314
Degradome 5' end Frequency
0.0
0.5
1.0
1.5
2.0
2.5
3.0
0
200
400
600
800
Transcript Position

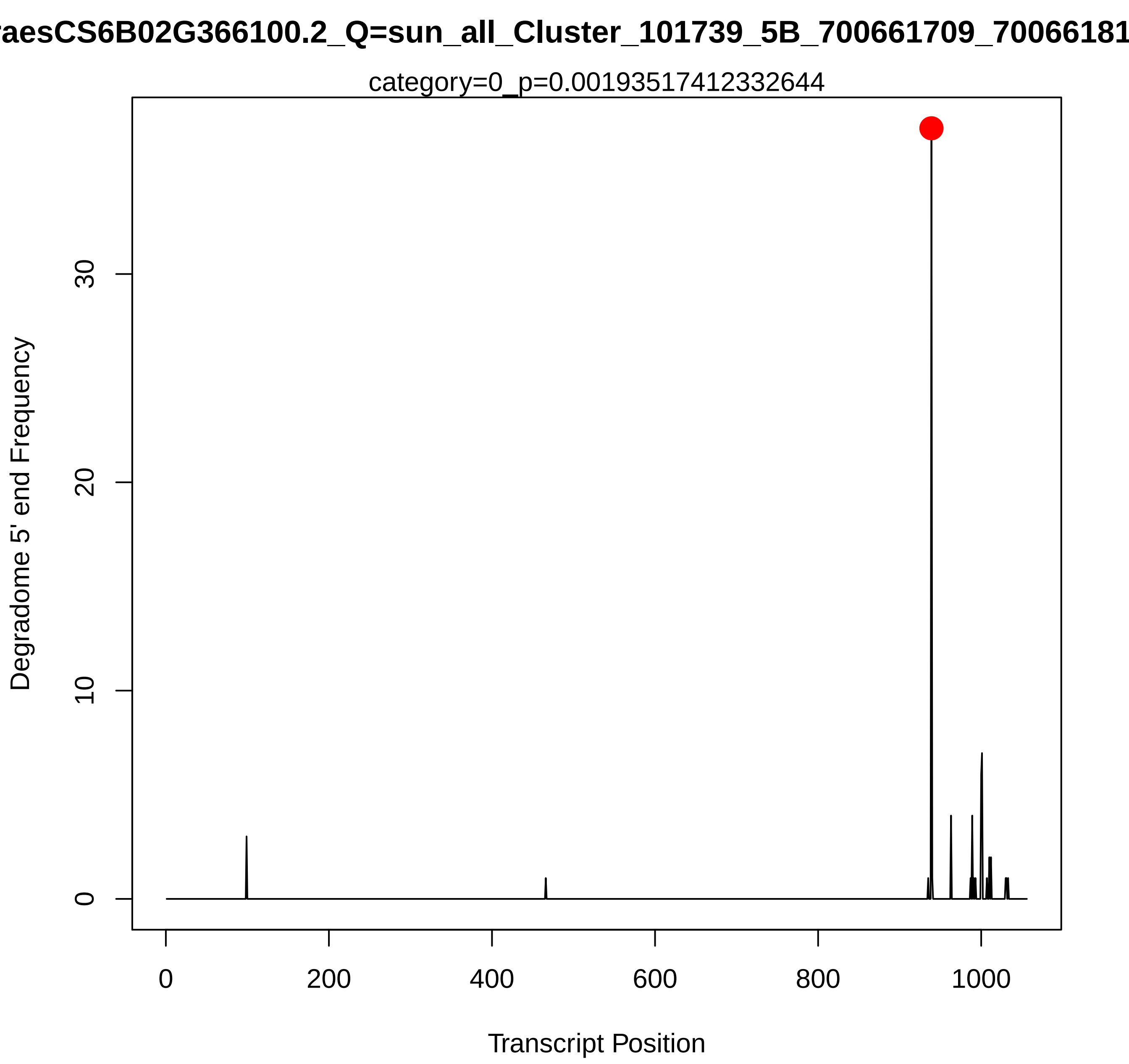
aesCS6B02G366100.2_Q=sun_all_Cluster_101739_5B_700661709_70066181
category=0_p=0.00193517412332644
Degradome 5' end Frequency
0
10
20
30
0
200
400
600
800
1000
Transcript Position

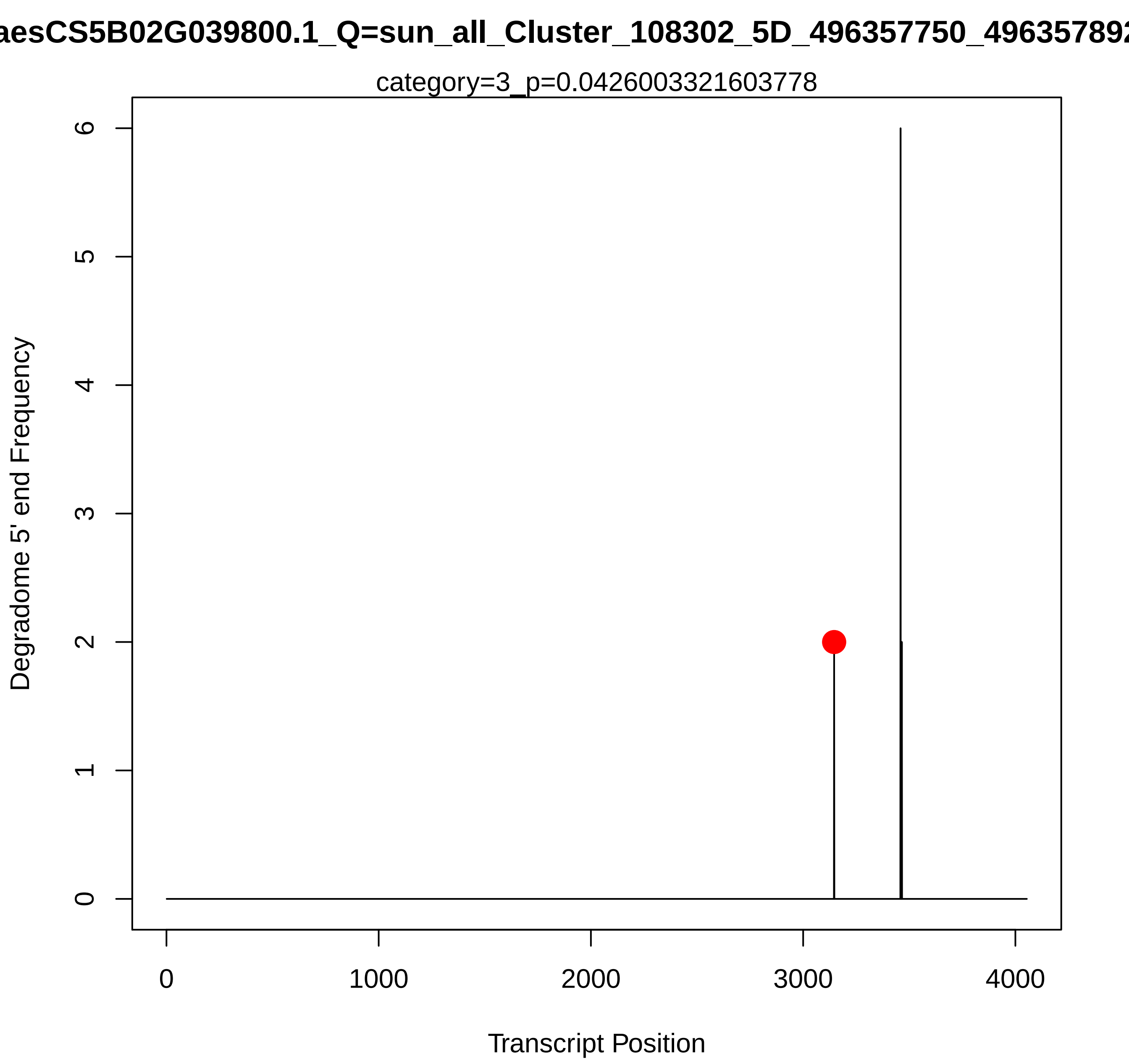

aesCS5B02G039800.1_Q=sun_all_Cluster_108302_5D_496357750_49635789
category=3_p=0.0426003321603778
Degradome 5' end Frequency
0
1
2
3
4
5
6
0
1000
2000
3000
4000
Transcript Position

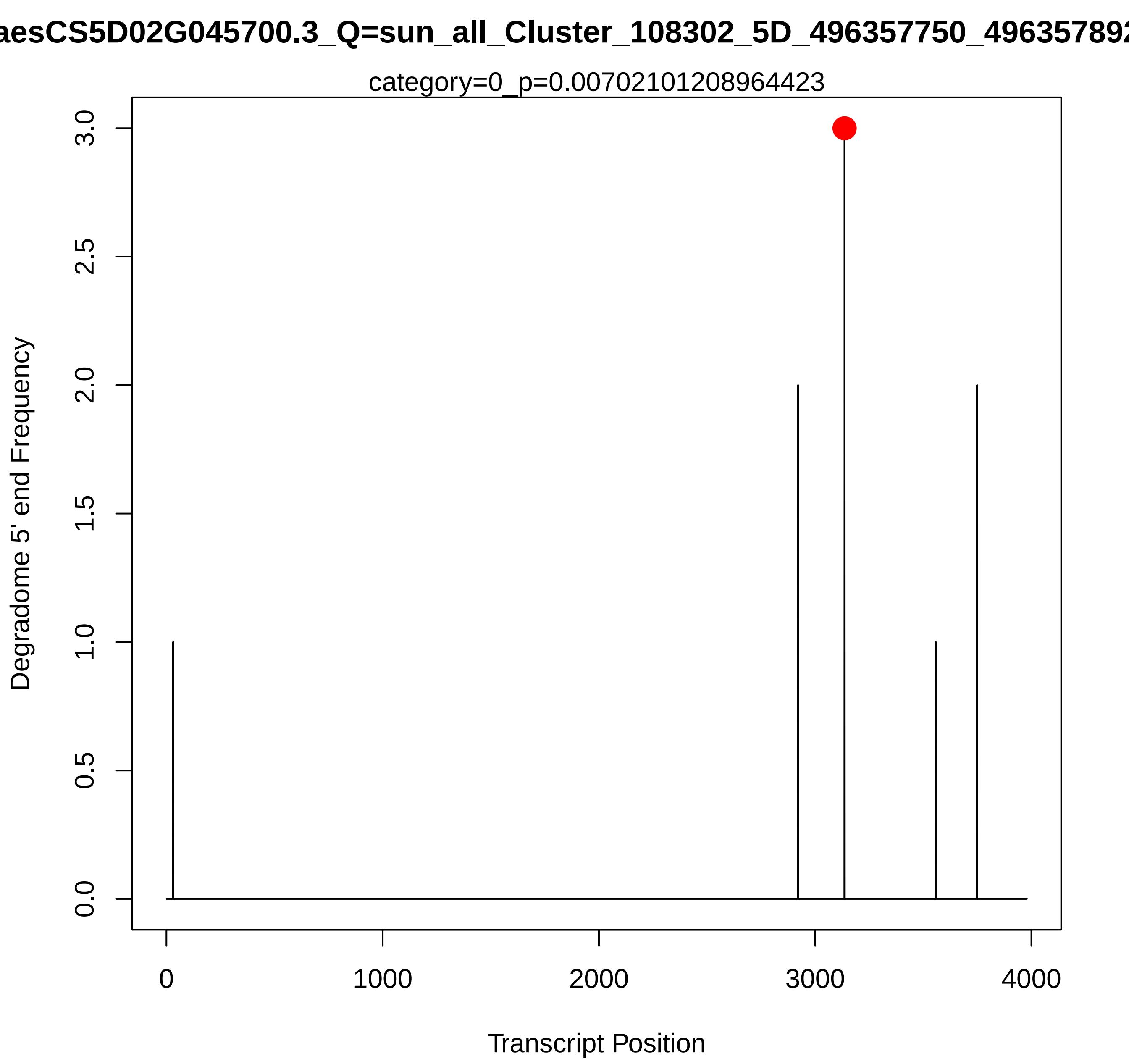

aesCS5D02G045700.3_Q=sun_all_Cluster_108302_5D_496357750_49635789
category=0_p=0.00702101208964423
Degradome 5' end Frequency
0.0
0.5
1.0
1.5
2.0
2.5
3.0
0
1000
2000
3000
4000
Transcript Position

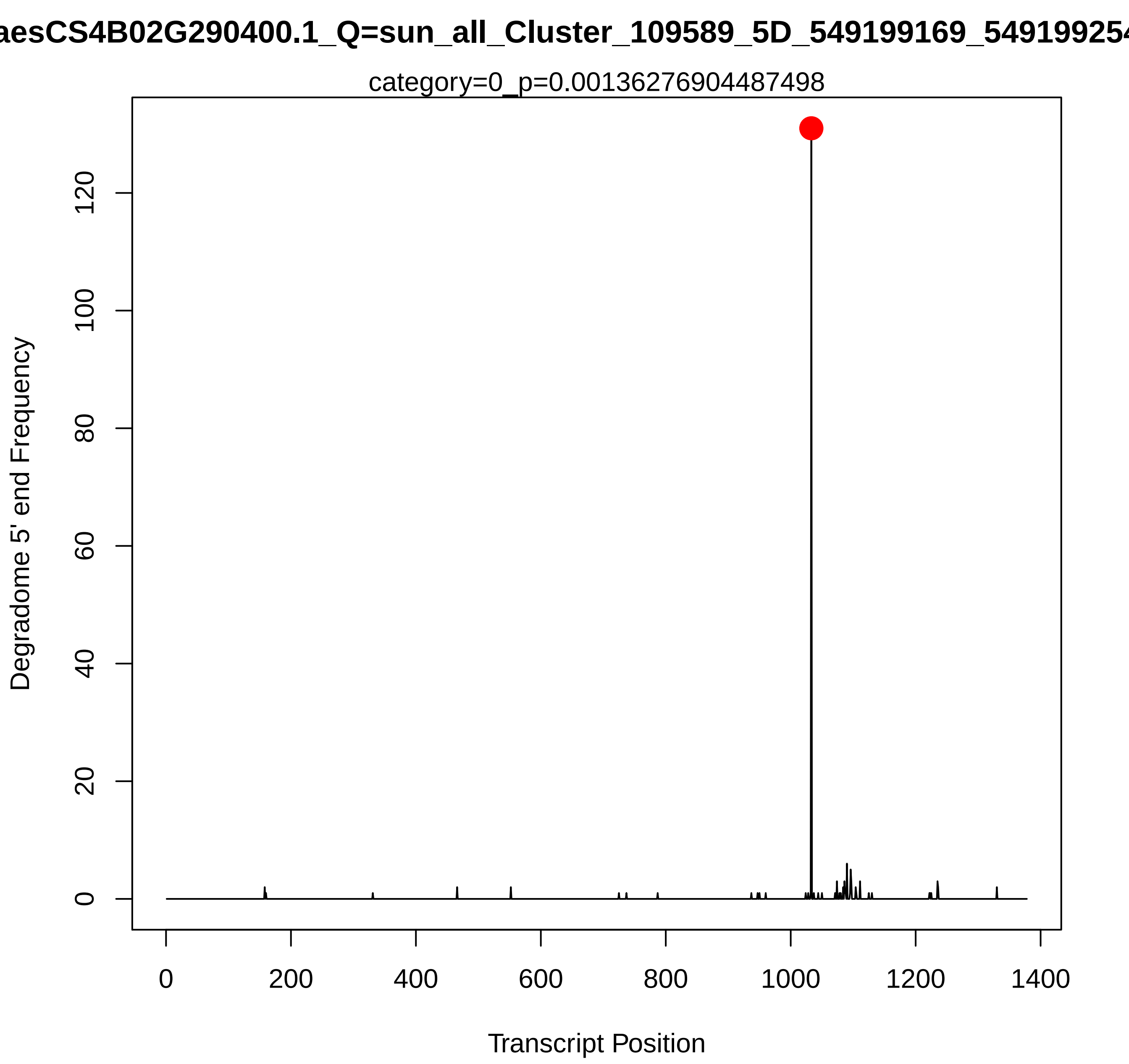

aesCS4B02G290400.1_Q=sun_all_Cluster_109589_5D_549199169_54919925
category=0_p=0.00136276904487498
Degradome 5' end Frequency
0
20
40
60
80
100
120
0
200
400
600
800
1000
1200
1400
Transcript Position

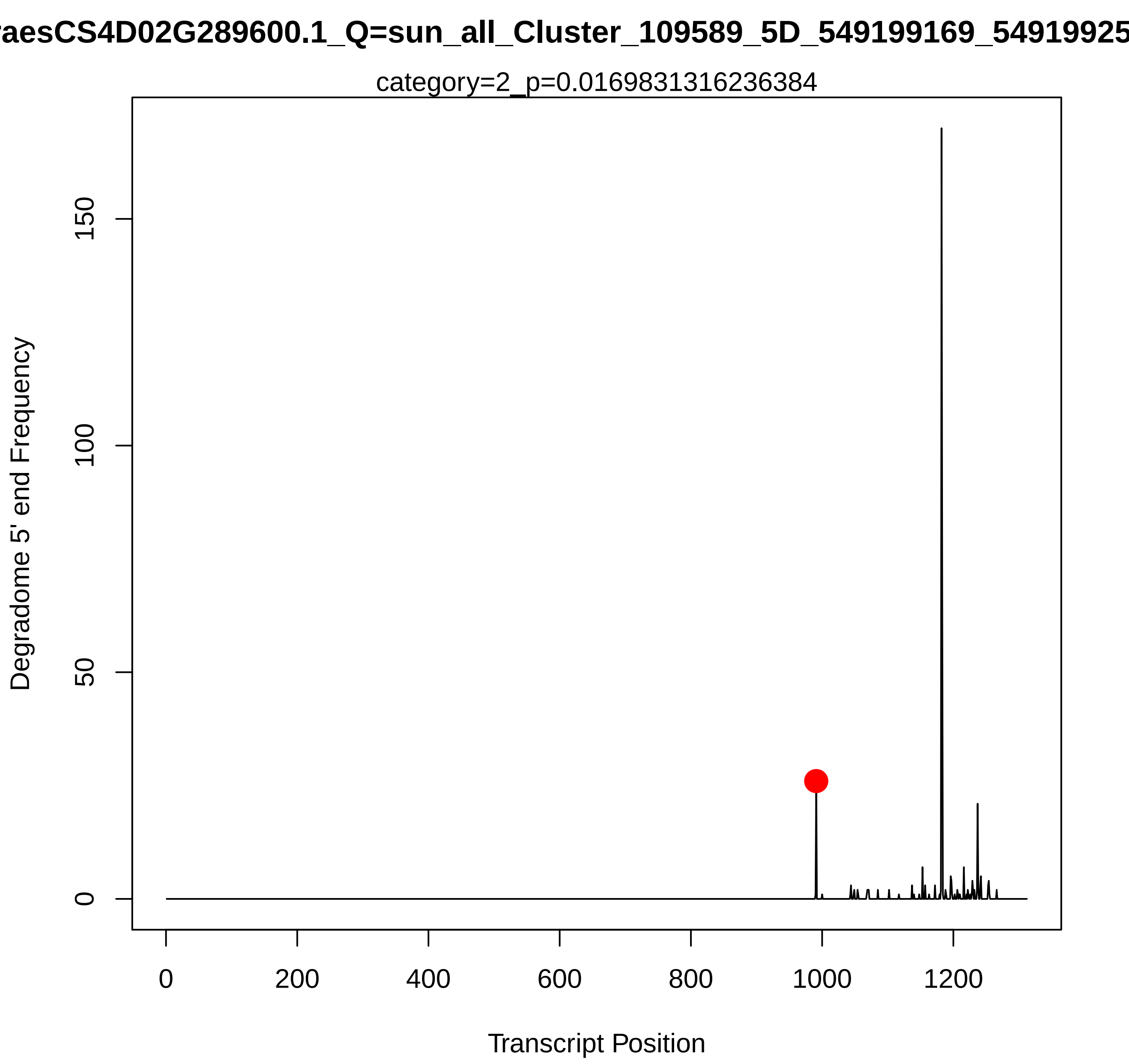
TraesCS4D02G289600.1_Q=sun_all_Cluster_109589_5D_549199169_54919925
category=2_p=0.0169831316236384
Degradome 5' end Frequency
0
50
100
150
0
200
400
600
800
1000
1200
Transcript Position

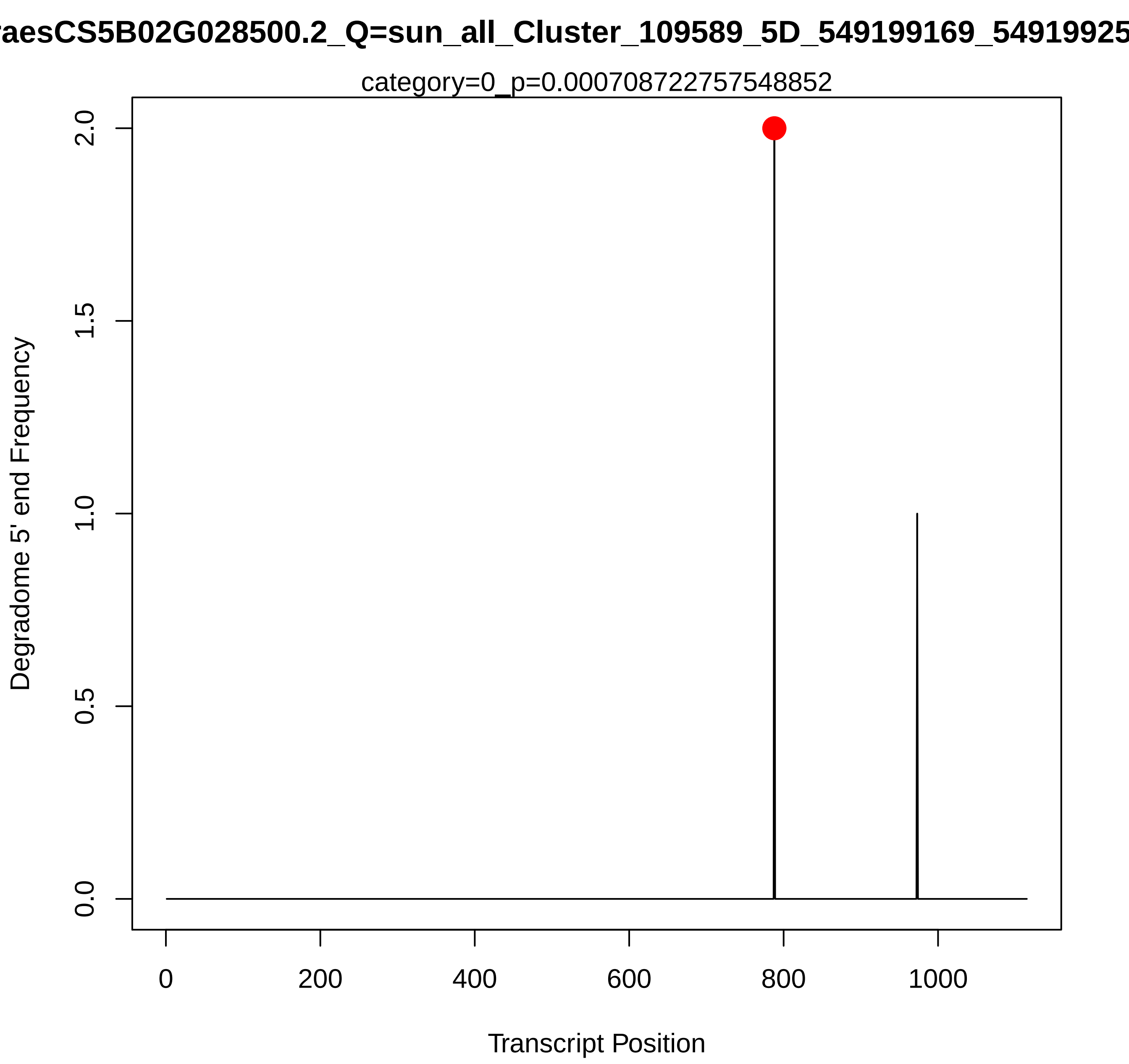
aesCS5B02G028500.2_Q=sun_all_Cluster_109589_5D_549199169_54919925
category=0_p=0.000708722757548852
Degradome 5' end Frequency
2.0
1.5
1.0
0.5
0.0
0
200
400
600
800
1000
Transcript Position

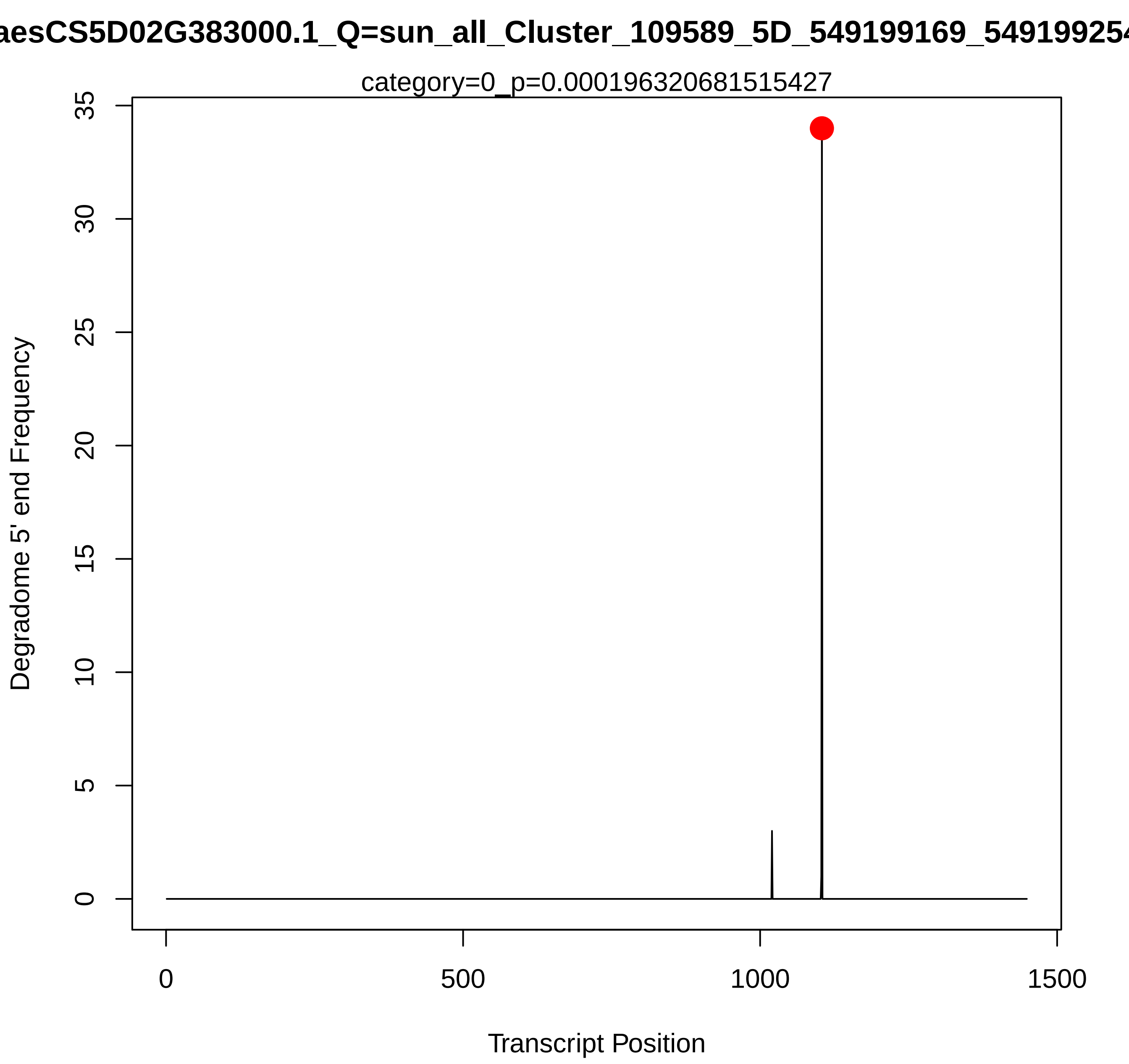
aesCS5D02G383000.1_Q=sun_all_Cluster_109589_5D_549199169_54919925
category=0_p=0.000196320681515427
Degradome 5' end Frequency
Transcript Position
0
5
10
15
20
25
30
35
0
500
1000
1500

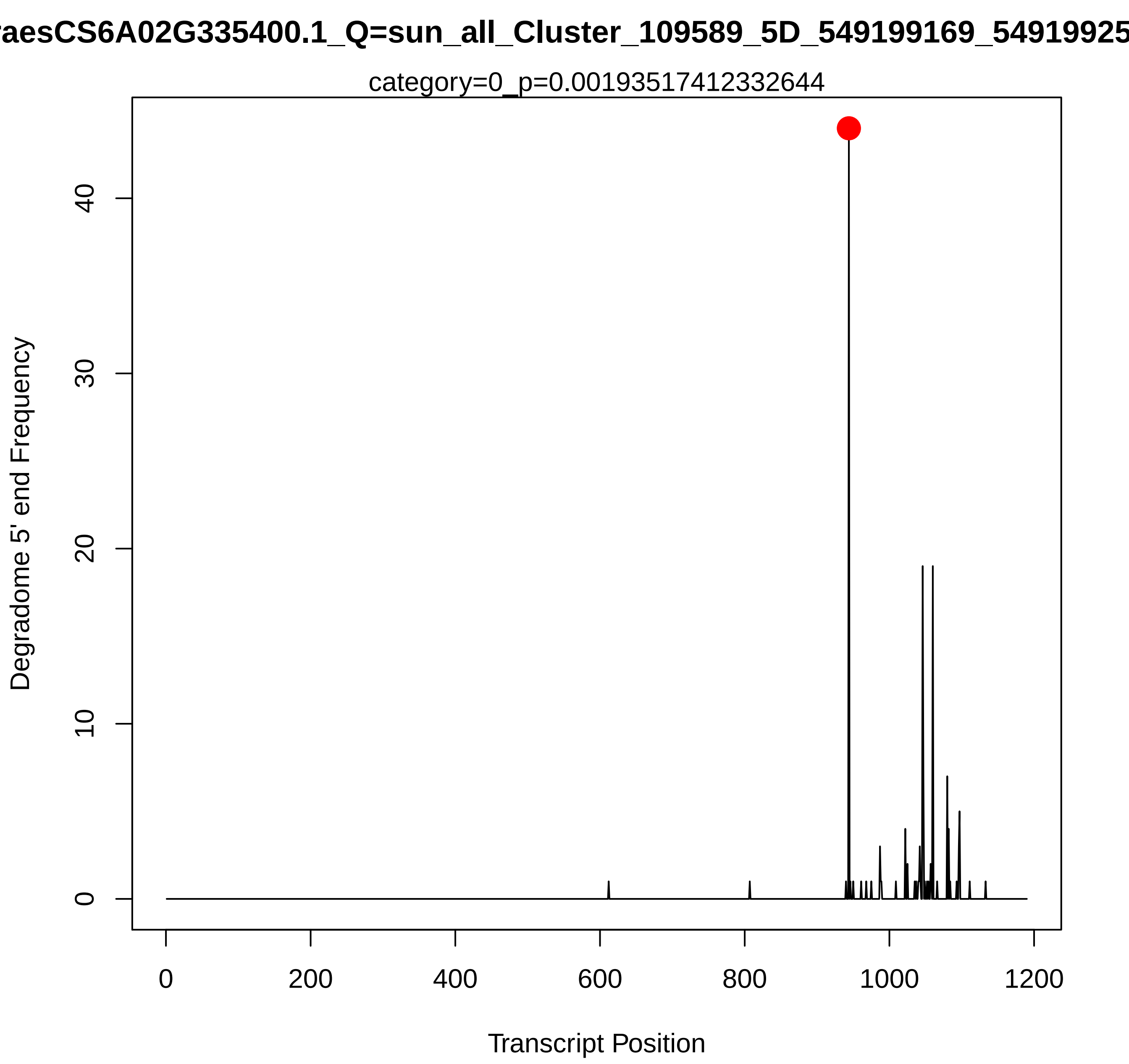

aesCS6A02G335400.1_Q=sun_all_Cluster_109589_5D_549199169_54919925
category=0_p=0.00193517412332644
Degradome 5' end Frequency
0
10
20
30
40
0
200
400
600
800
1000
1200
Transcript Position

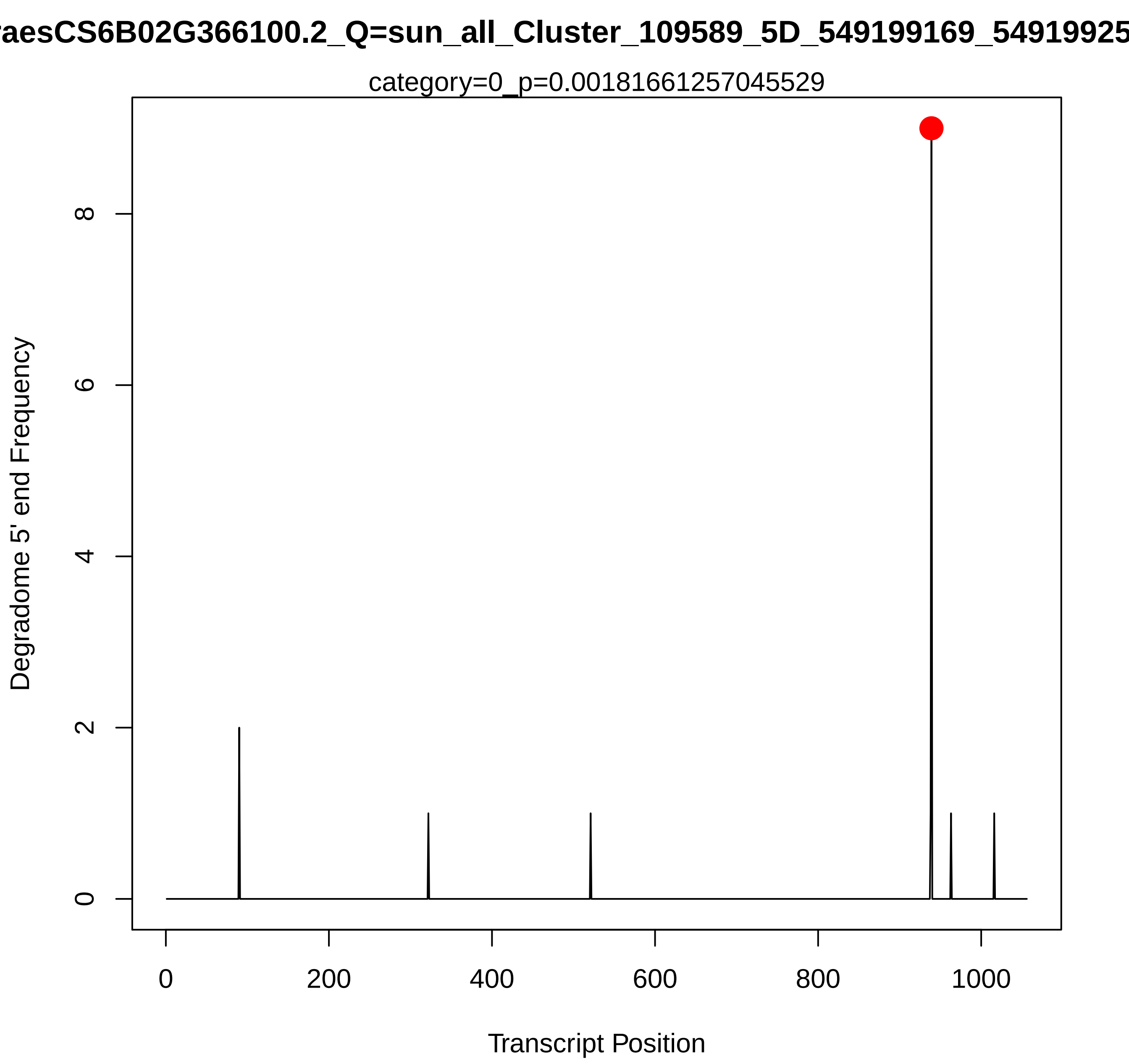

raesCS6B02G366100.2_Q=sun_all_Cluster_109589_5D_549199169_54919925
category=0_p=0.00181661257045529
Degradome 5' end Frequency
0
2
4
6
8
0
200
400
600
800
1000
Transcript Position

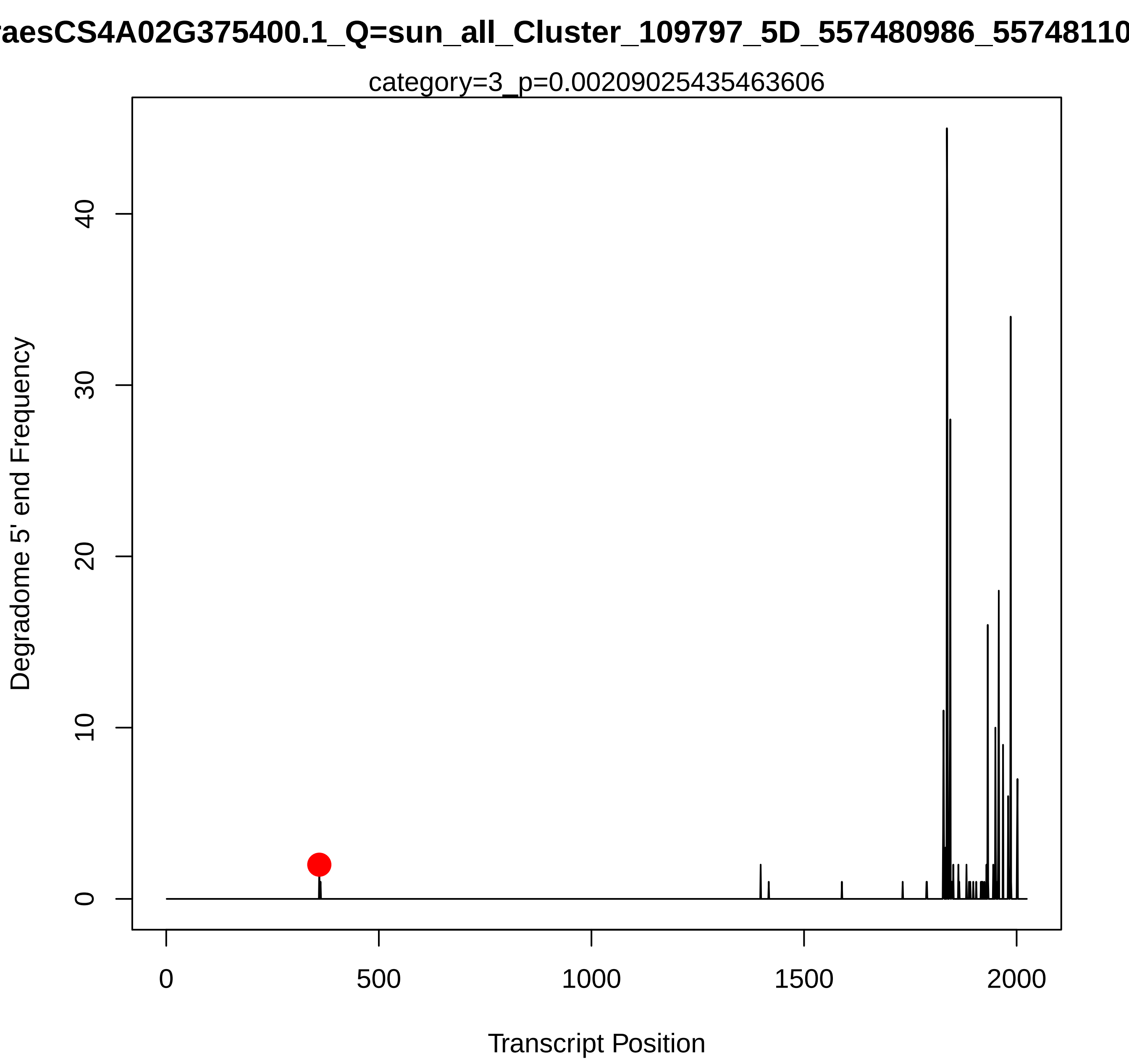
aesCS4A02G375400.1_Q=sun_all_Cluster_109797_5D_557480986_55748110
category=3_p=0.00209025435463606
Degradome 5' end Frequency
0
10
20
30
40
0
500
1000
1500
2000
Transcript Position

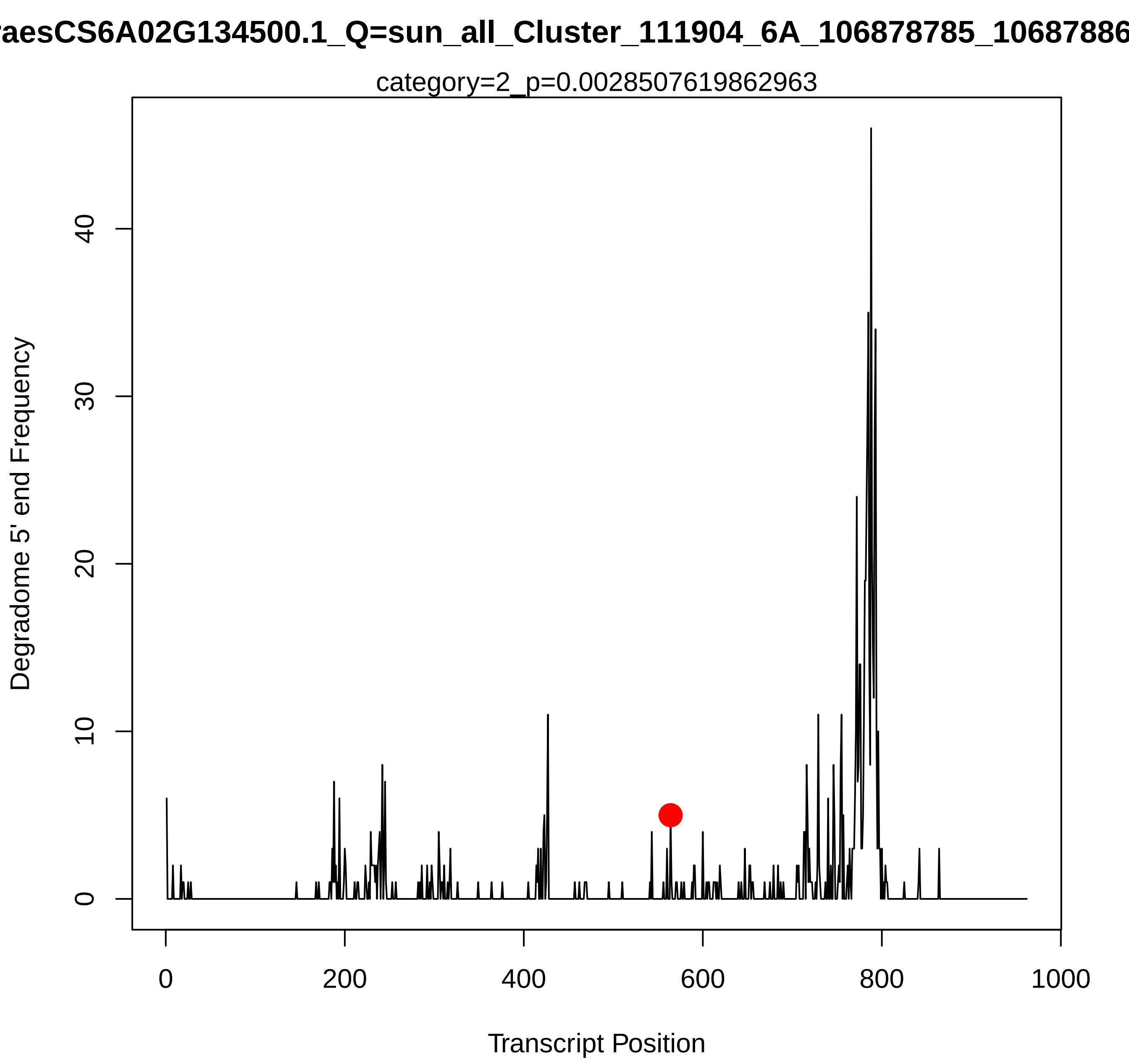

aesCS6A02G134500.1_Q=sun_all_Cluster_111904_6A_106878785_10687886
category=2_p=0.0028507619862963
Degradome 5' end Frequency
0
10
20
30
40
0
200
400
600
800
1000
Transcript Position

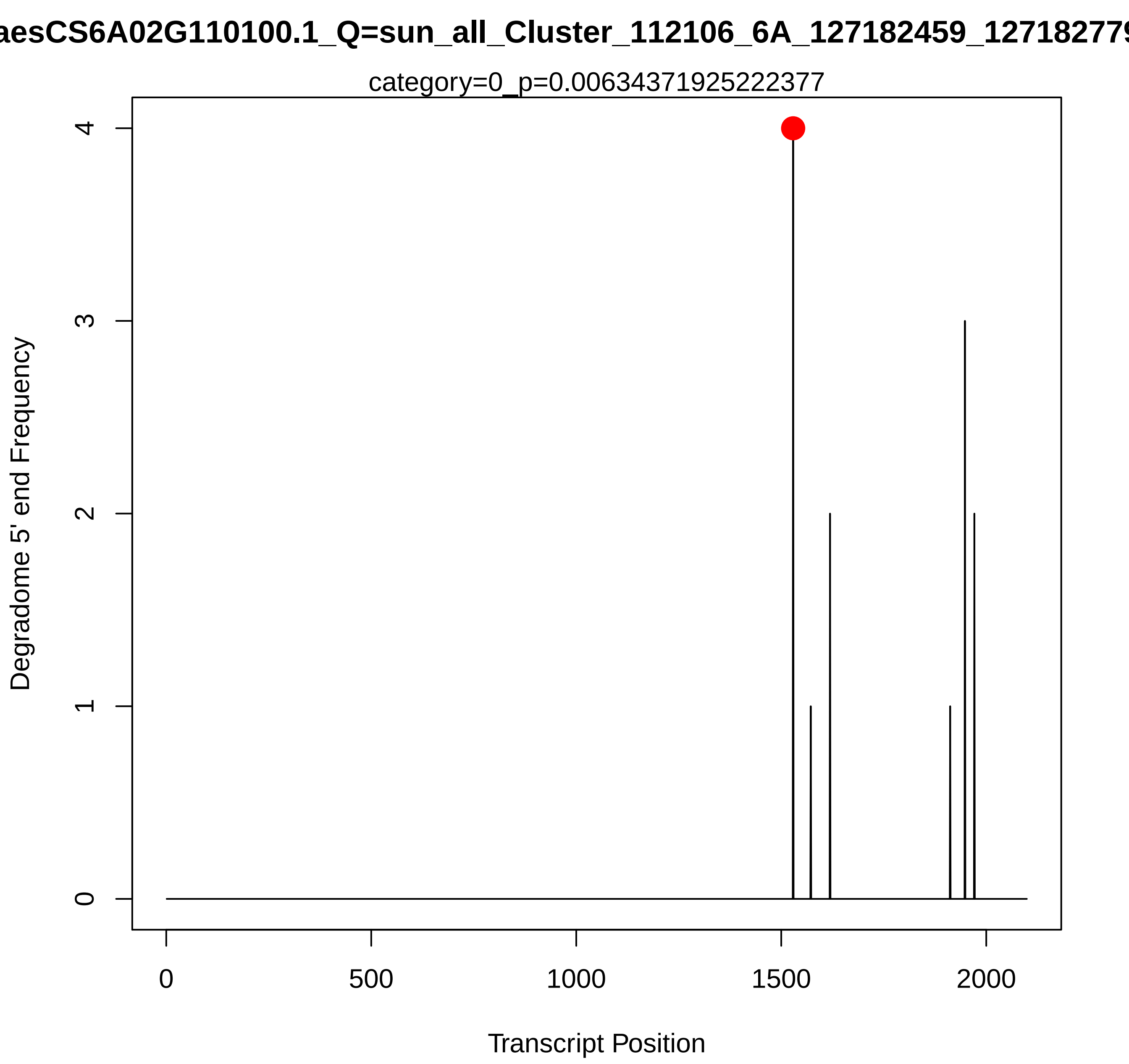
aesCS6A02G110100.1_Q=sun_all_Cluster_112106_6A_127182459_12718277
category=0_p=0.00634371925222377
Degradome 5' end Frequency
Transcript Position
0
1
2
3
4
0
500
1000
1500
2000

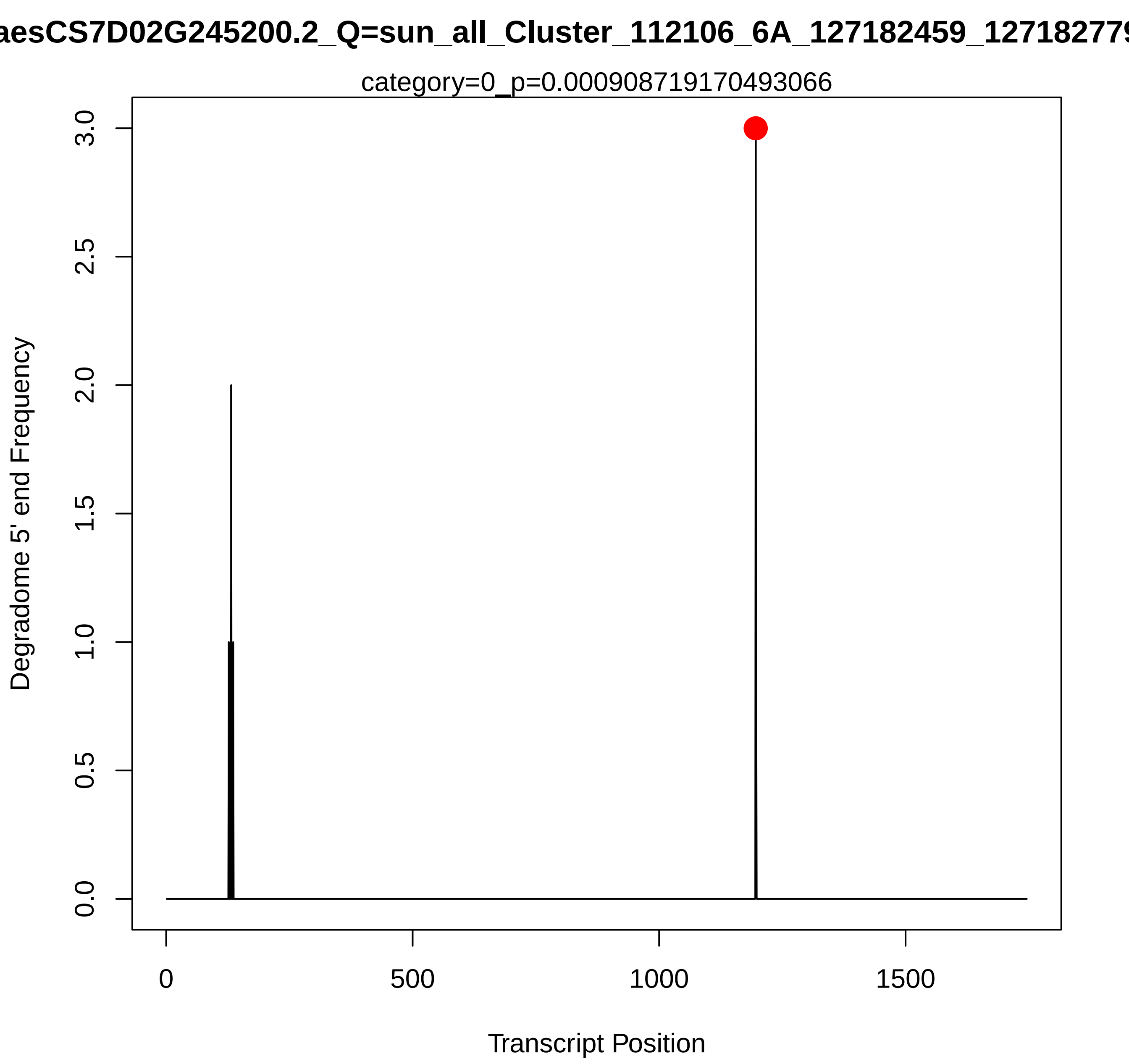
aesCS7D02G245200.2_Q=sun_all_Cluster_112106_6A_127182459_12718277
category=0_p=0.000908719170493066
Degradome 5' end Frequency
Transcript Position
0.0
0.5
1.0
1.5
2.0
2.5
3.0
0
500
1000
1500

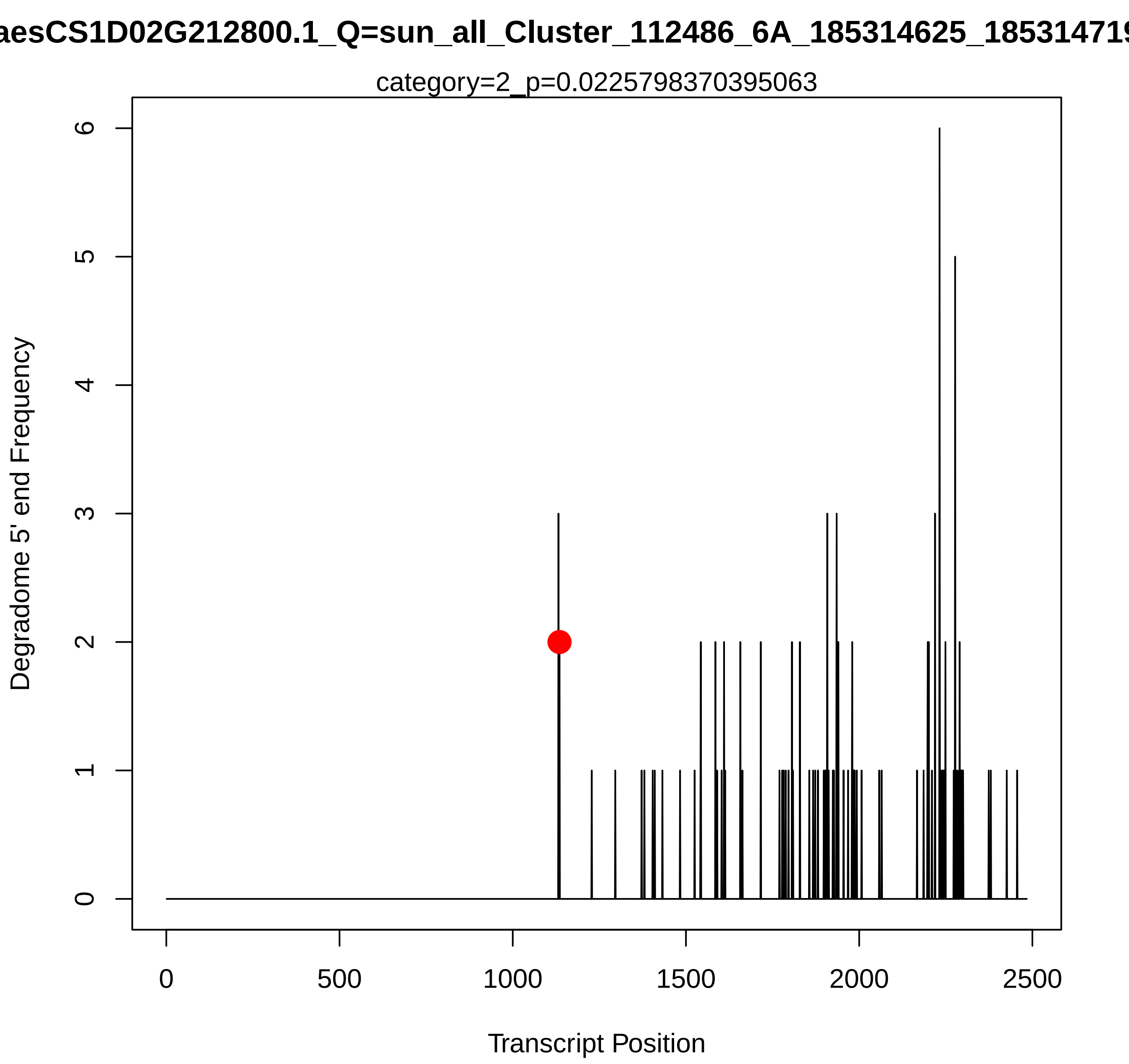
aesCS1D02G212800.1_Q=sun_all_Cluster_112486_6A_185314625_18531471
category=2_p=0.0225798370395063
Degradome 5' end Frequency
0
1
2
3
4
5
6
0
500
1000
1500
2000
2500
Transcript Position

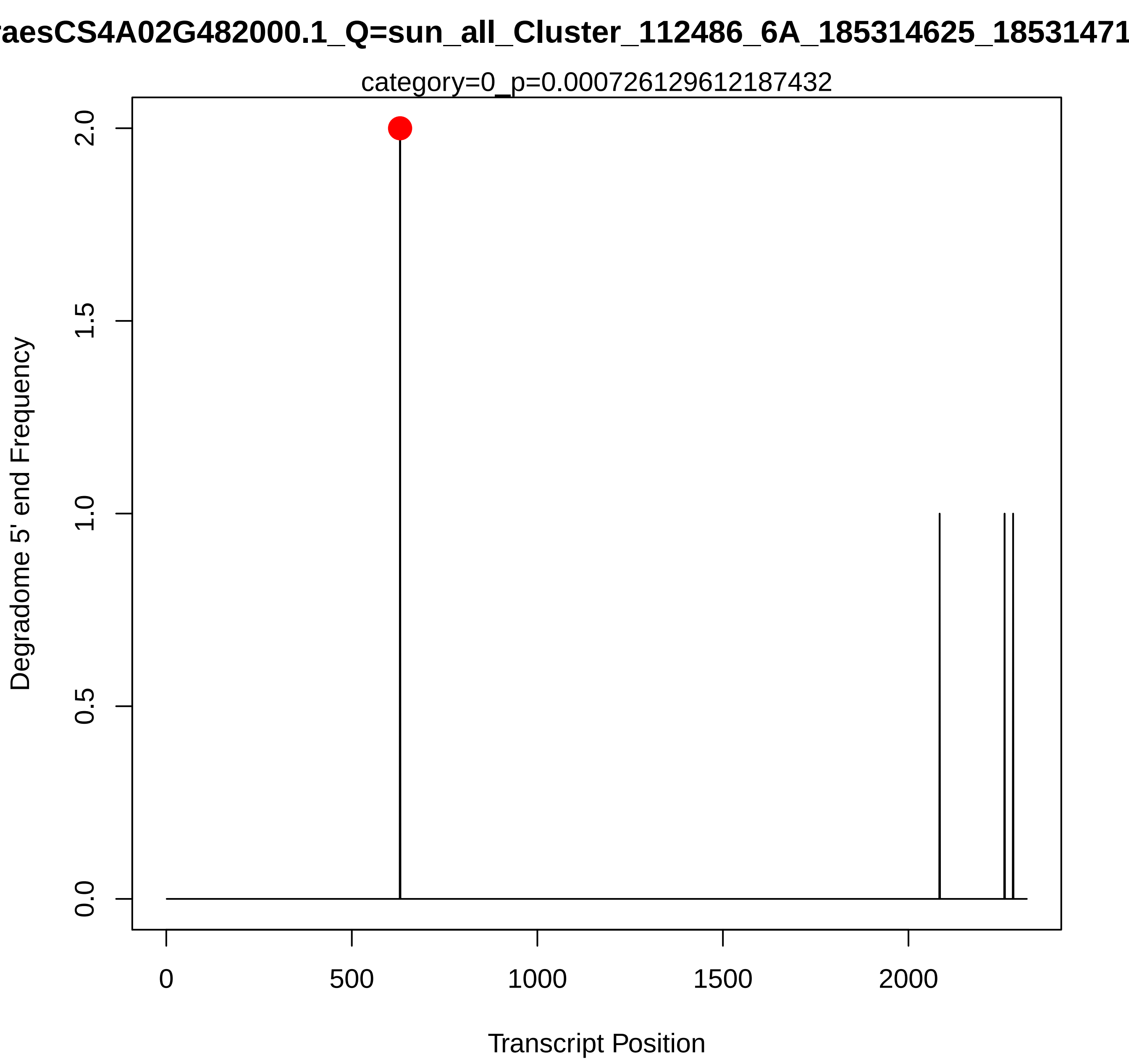

aesCS4A02G482000.1_Q=sun_all_Cluster_112486_6A_185314625_18531471
category=0_p=0.000726129612187432
Degradome 5' end Frequency
0.0
0.5
1.0
1.5
2.0
0
500
1000
1500
2000
Transcript Position

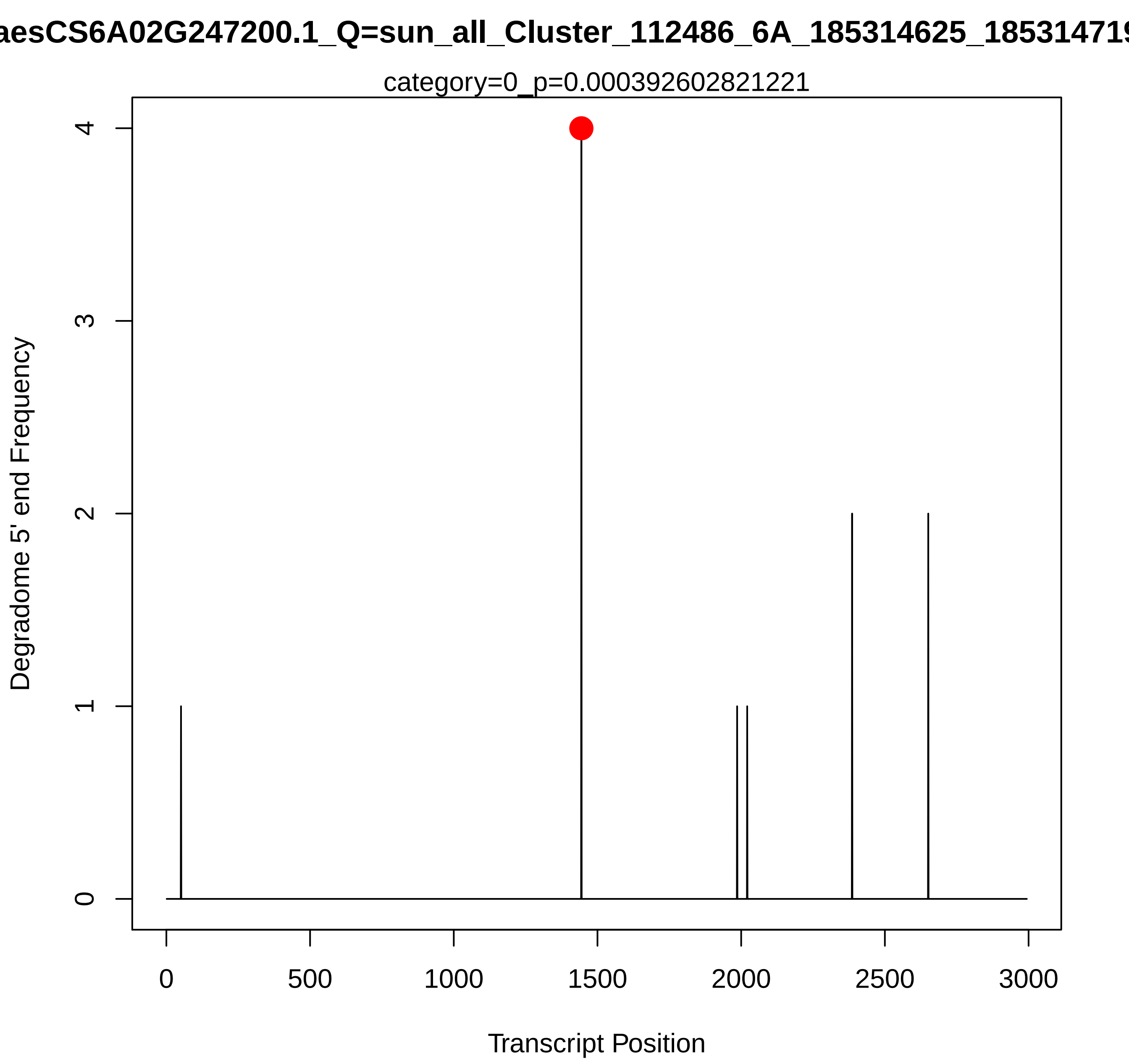
aesCS6A02G247200.1_Q=sun_all_Cluster_112486_6A_185314625_18531471
category=0_p=0.000392602821221
Degradome 5' end Frequency
0
1
2
3
4
0
500
1000
1500
2000
2500
3000
Transcript Position

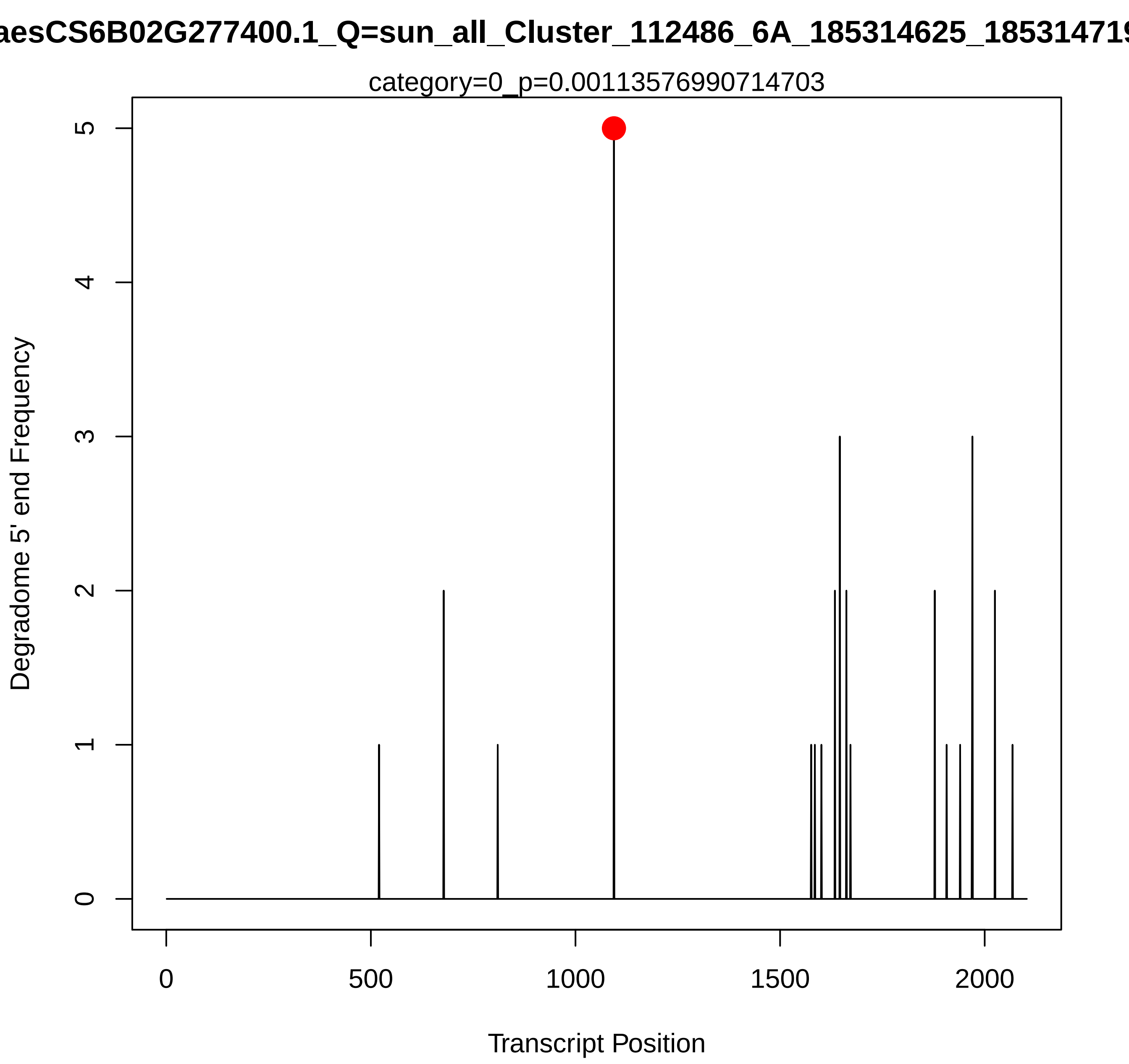
aesCS6B02G277400.1_Q=sun_all_Cluster_112486_6A_185314625_18531471
category=0_p=0.00113576990714703
Degradome 5' end Frequency
Transcript Position
0
1
2
3
4
5
0
500
1000
1500
2000

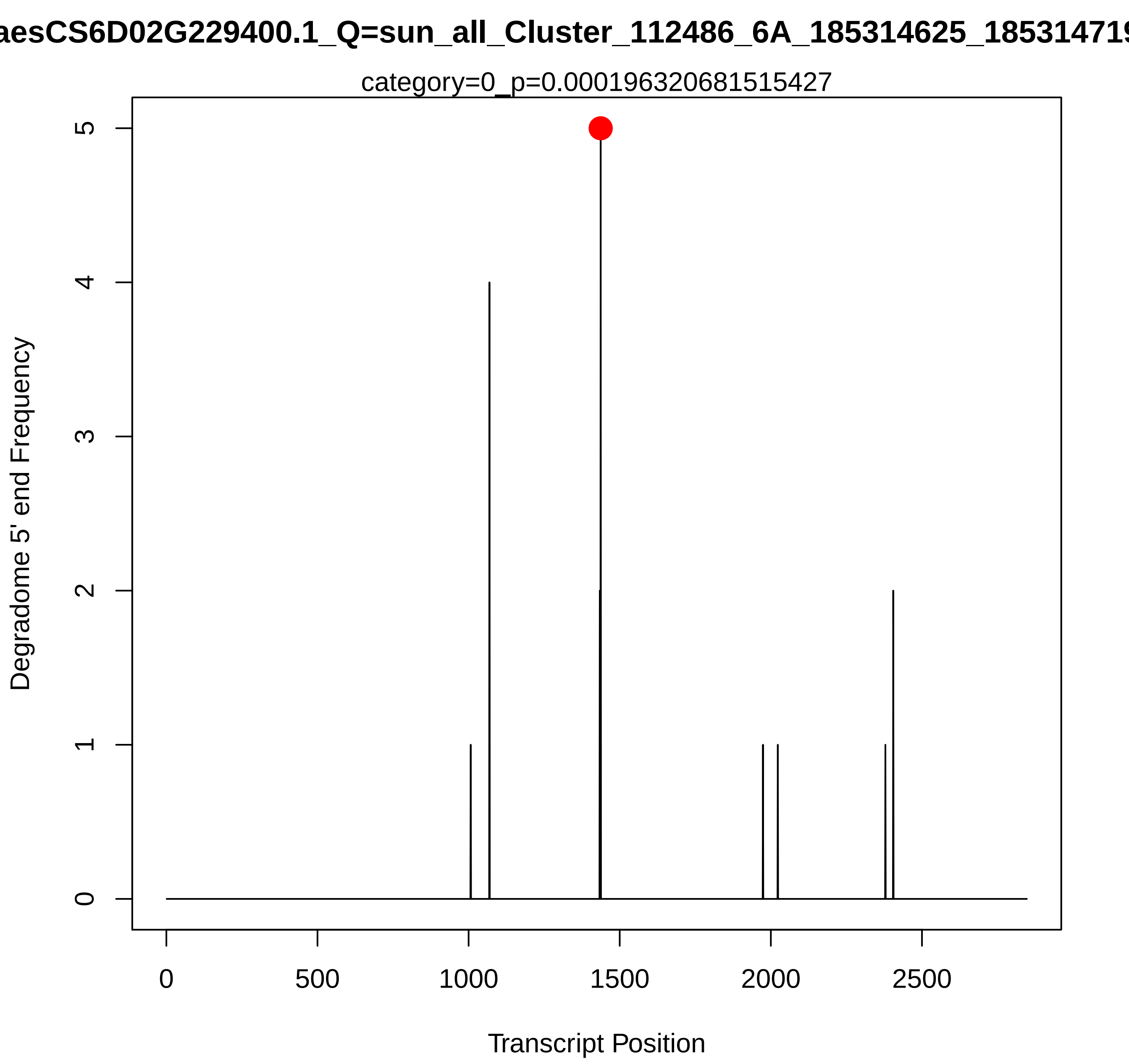
aesCS6D02G229400.1_Q=sun_all_Cluster_112486_6A_185314625_18531471
category=0_p=0.000196320681515427
Degradome 5' end Frequency
0
1
2
3
4
5
0
500
1000
1500
2000
2500
Transcript Position

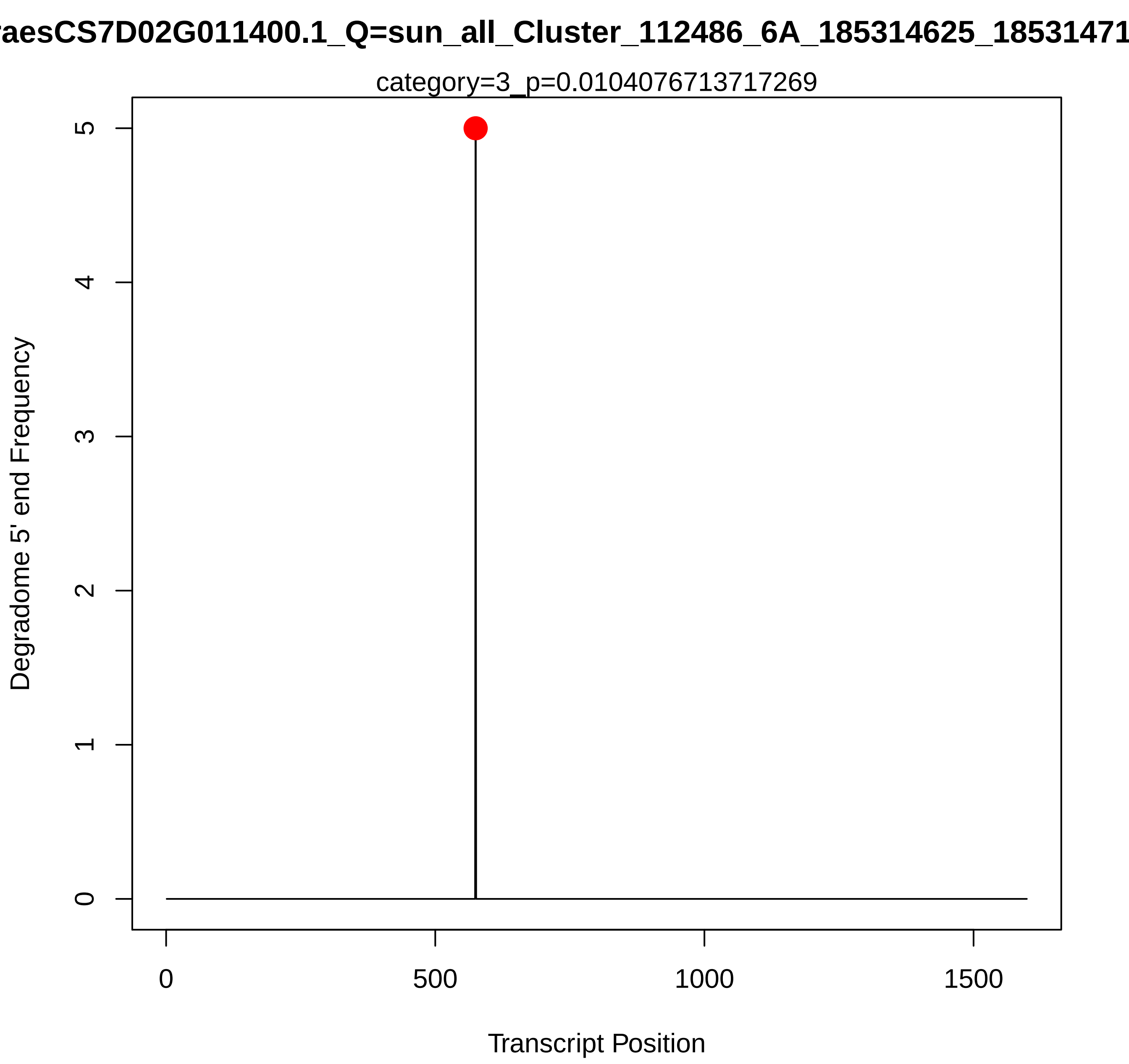
raesCS7D02G011400.1_Q=sun_all_Cluster_112486_6A_185314625_18531471
category=3_p=0.0104076713717269
Degradome 5' end Frequency
Transcript Position
0
1
2
3
4
5
0
500
1000
1500

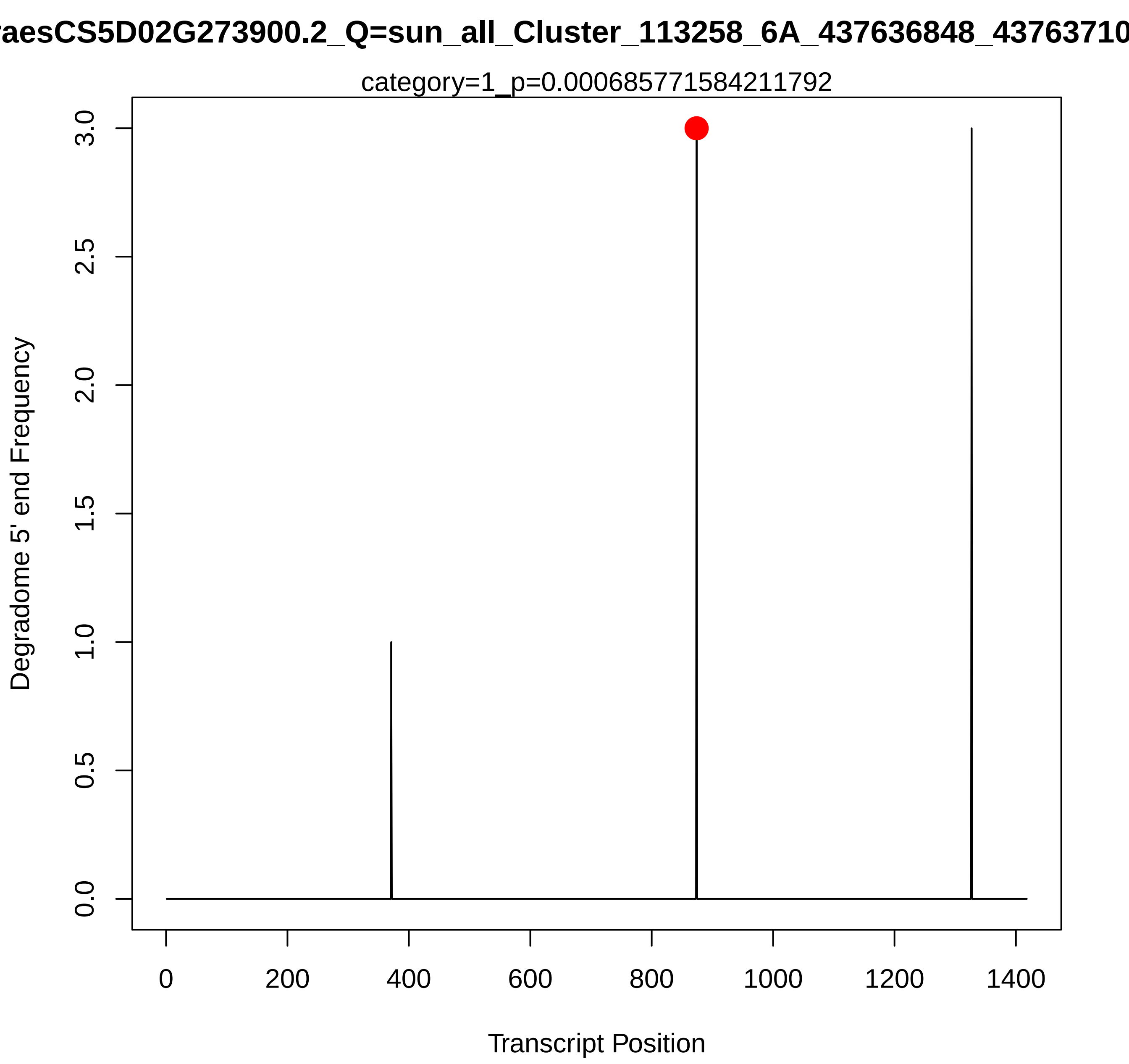

raesCS5D02G273900.2_Q=sun_all_Cluster_113258_6A_437636848_43763710
category=1_p=0.000685771584211792
Degradome 5' end Frequency
0.0
0.5
1.0
1.5
2.0
2.5
3.0
0
200
400
600
800
1000
1200
1400
Transcript Position

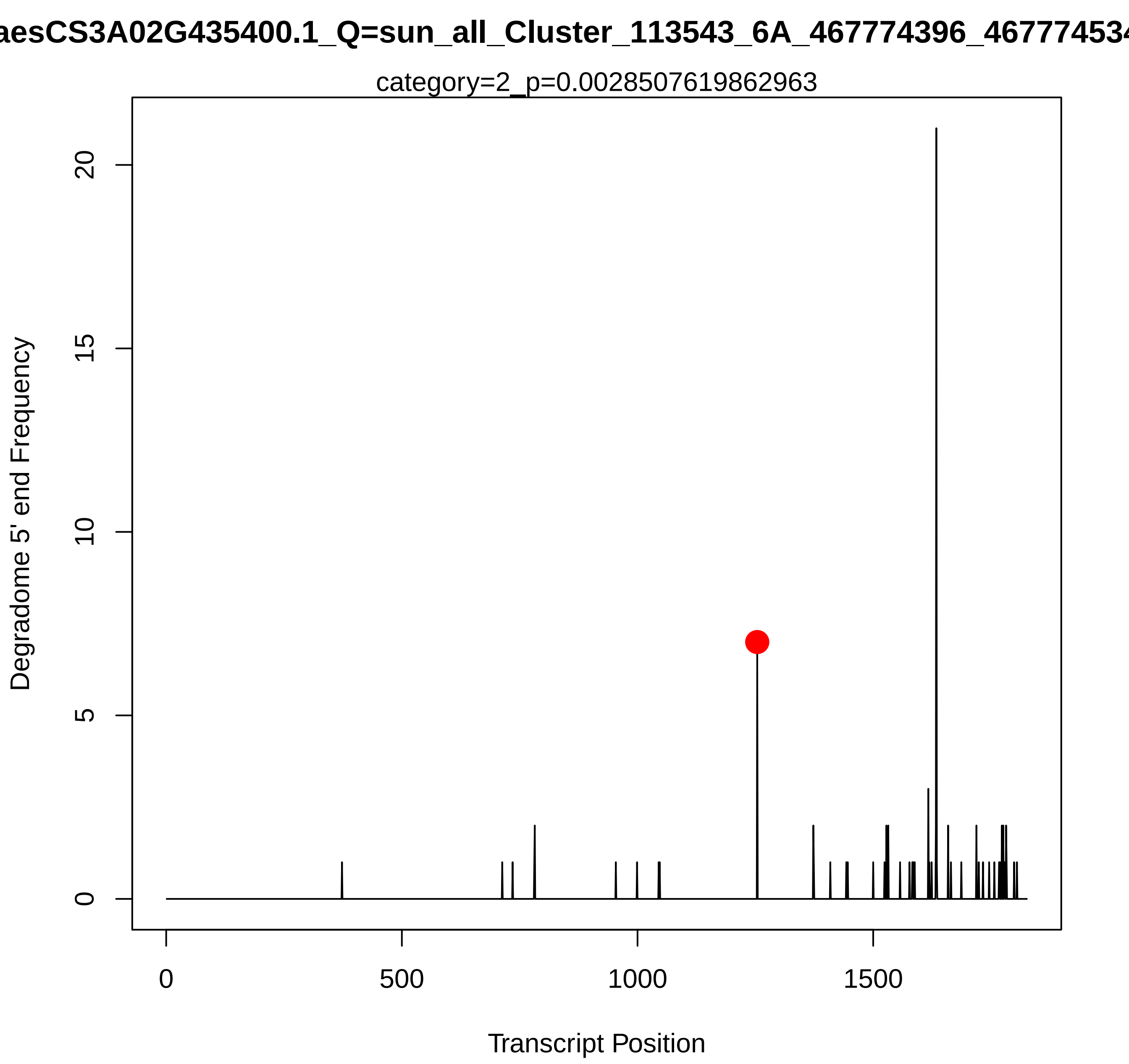

aesCS3A02G435400.1_Q=sun_all_Cluster_113543_6A_467774396_46777453
category=2_p=0.0028507619862963
Degradome 5' end Frequency
Transcript Position
0
5
10
15
20
0
500
1000
1500

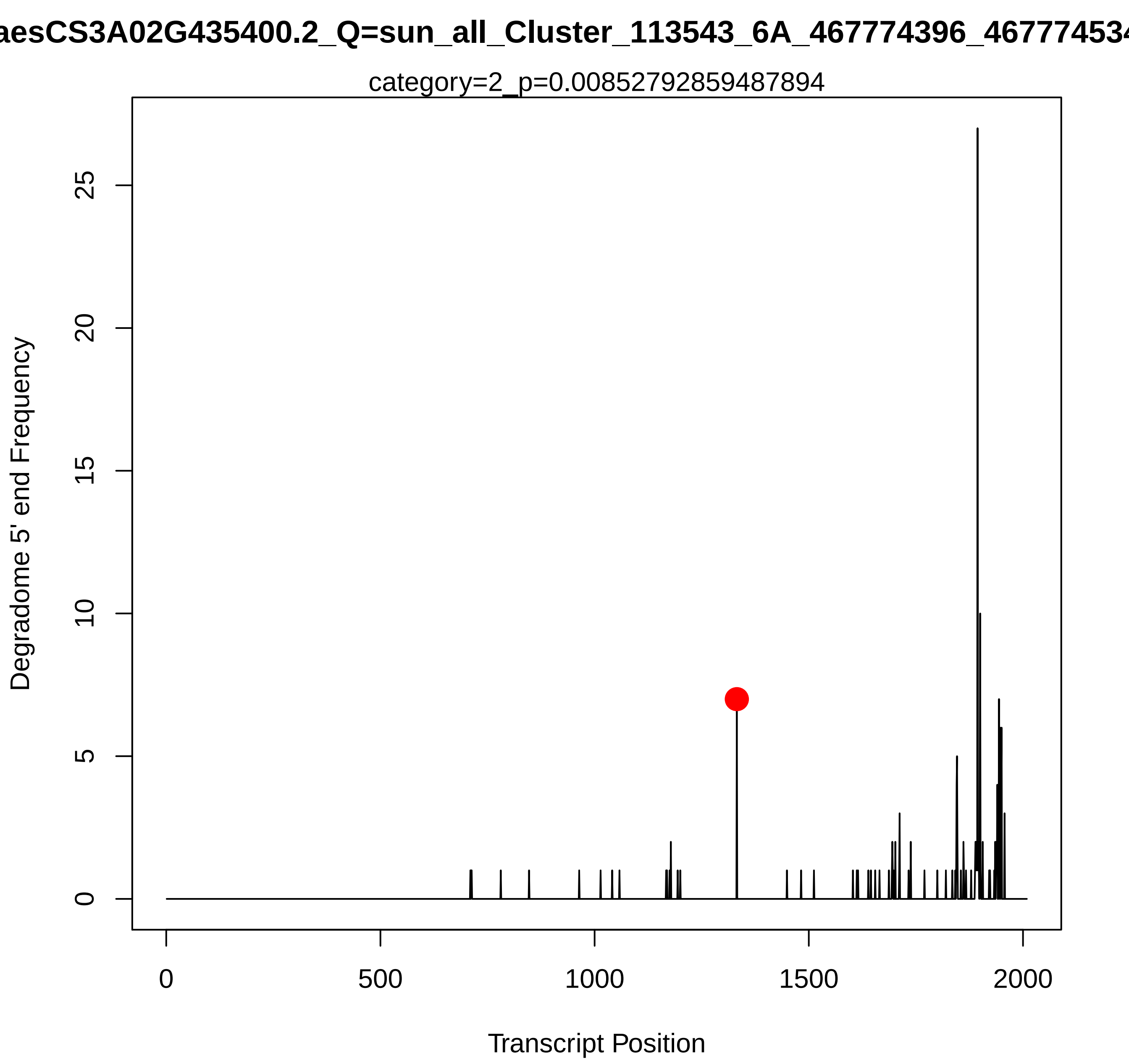
aesCS3A02G435400.2_Q=sun_all_Cluster_113543_6A_467774396_46777453
category=2_p=0.00852792859487894
Degradome 5' end Frequency
Transcript Position
0
5
10
15
20
25
0
500
1000
1500
2000

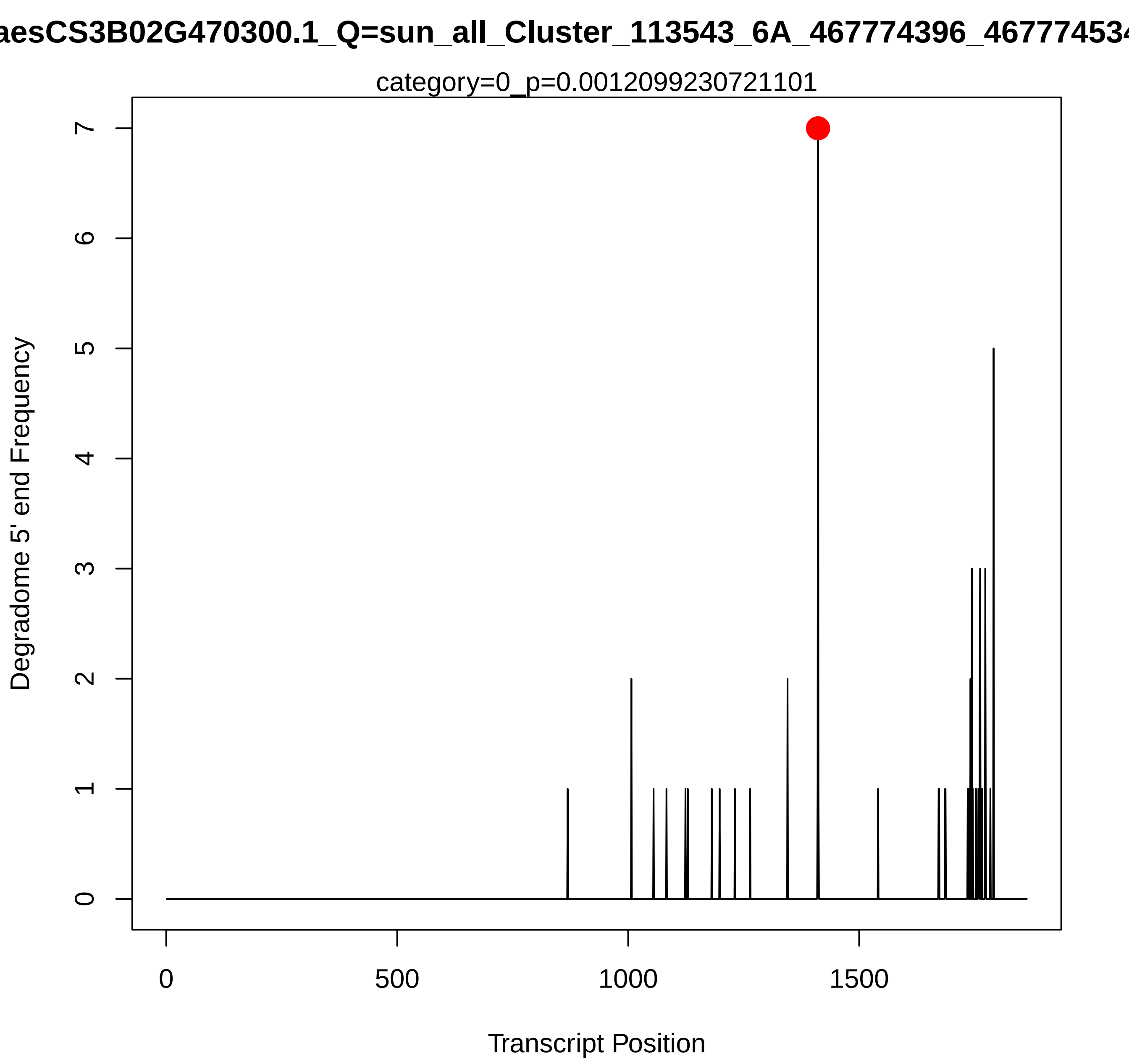
aesCS3B02G470300.1_Q=sun_all_Cluster_113543_6A_467774396_46777453
category=0_p=0.0012099230721101
Degradome 5' end Frequency
Transcript Position
0
1
2
3
4
5
6
7
0
500
1000
1500

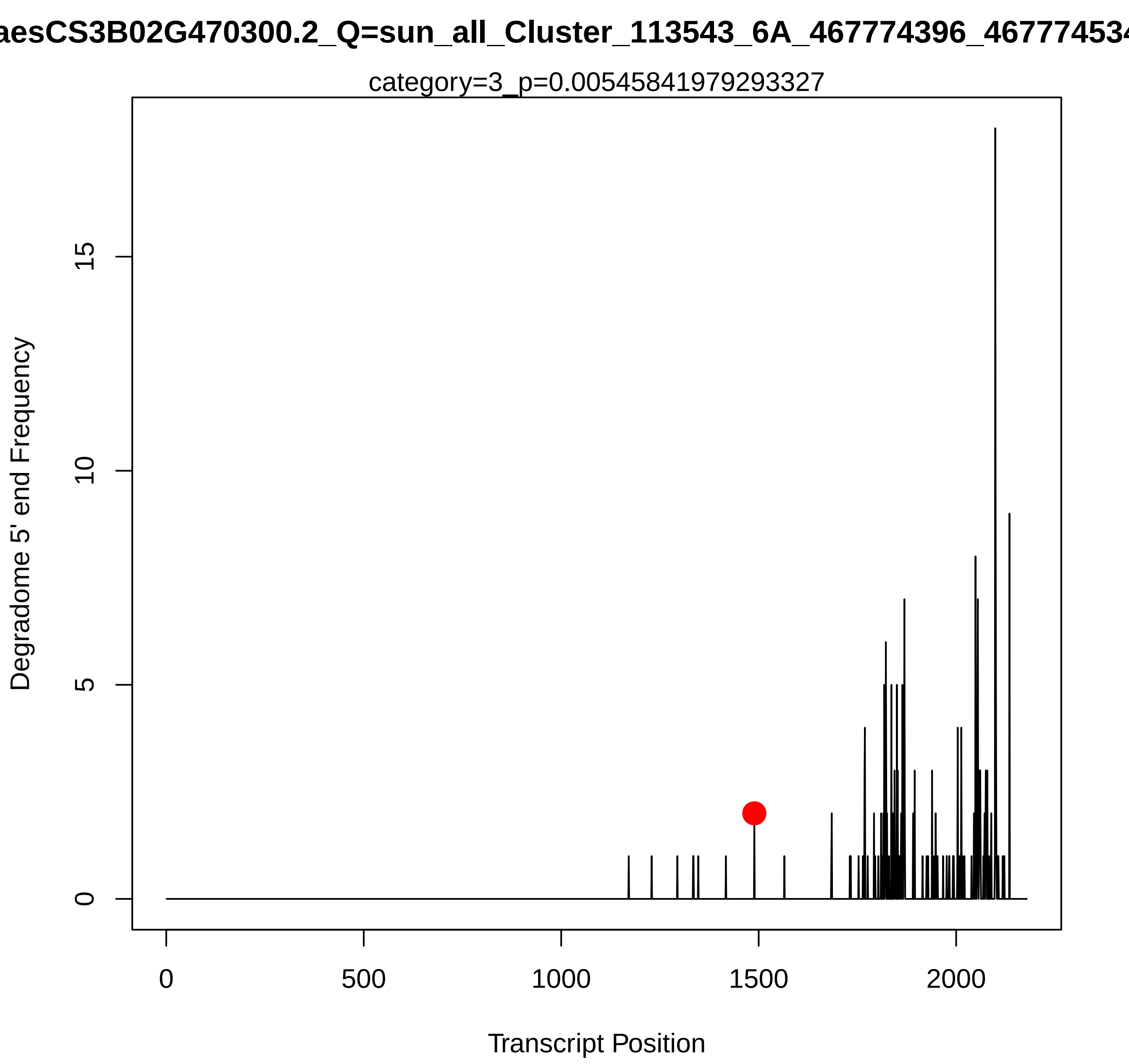
aesCS3B02G470300.2_Q=sun_all_Cluster_113543_6A_467774396_46777453
category=3_p=0.00545841979293327
Degradome 5' end Frequency
0
5
10
15
0
500
1000
1500
2000
Transcript Position

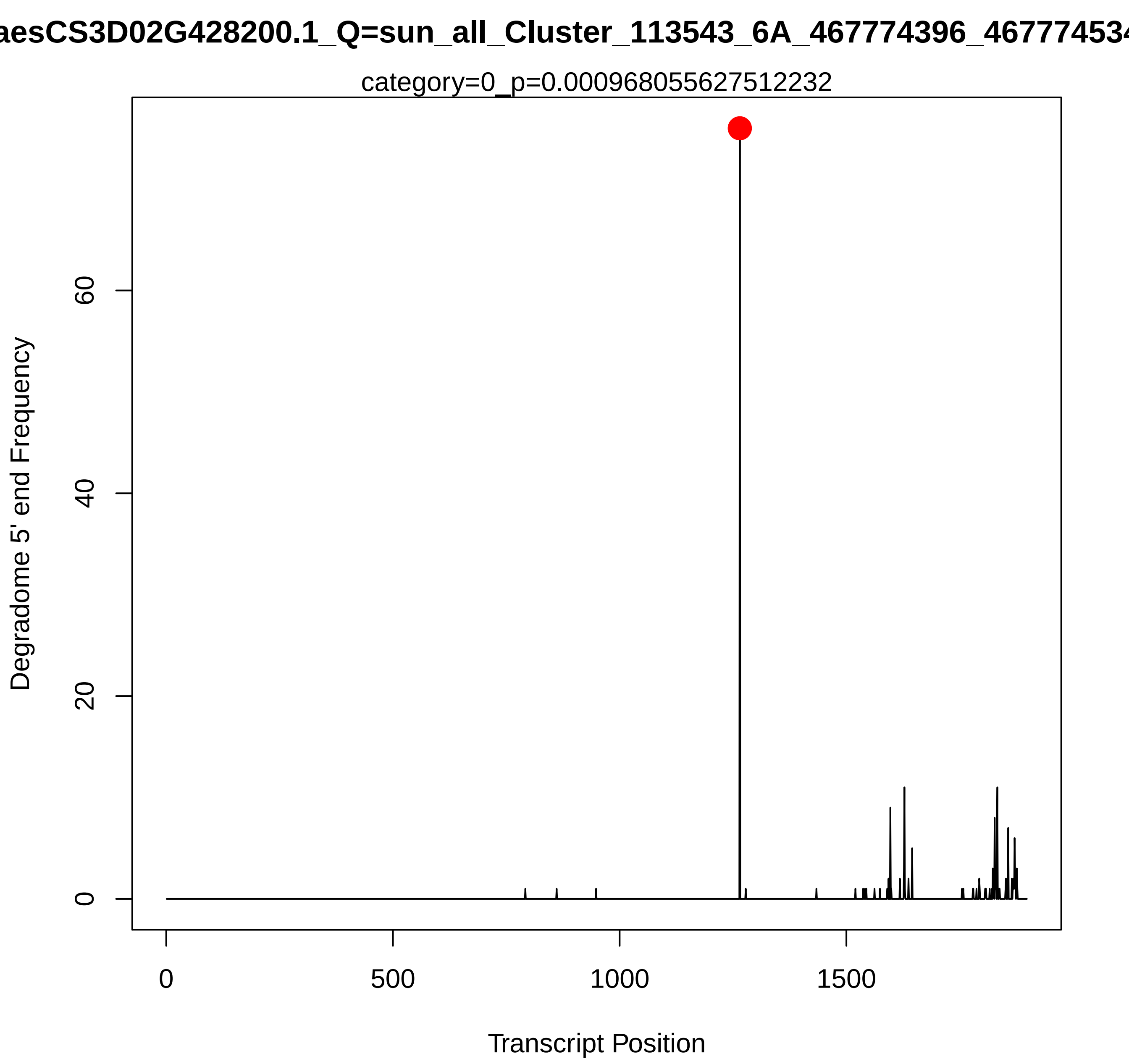
aesCS3D02G428200.1_Q=sun_all_Cluster_113543_6A_467774396_46777453
category=0_p=0.000968055627512232
Degradome 5' end Frequency
0
20
40
60
0
500
1000
1500
Transcript Position

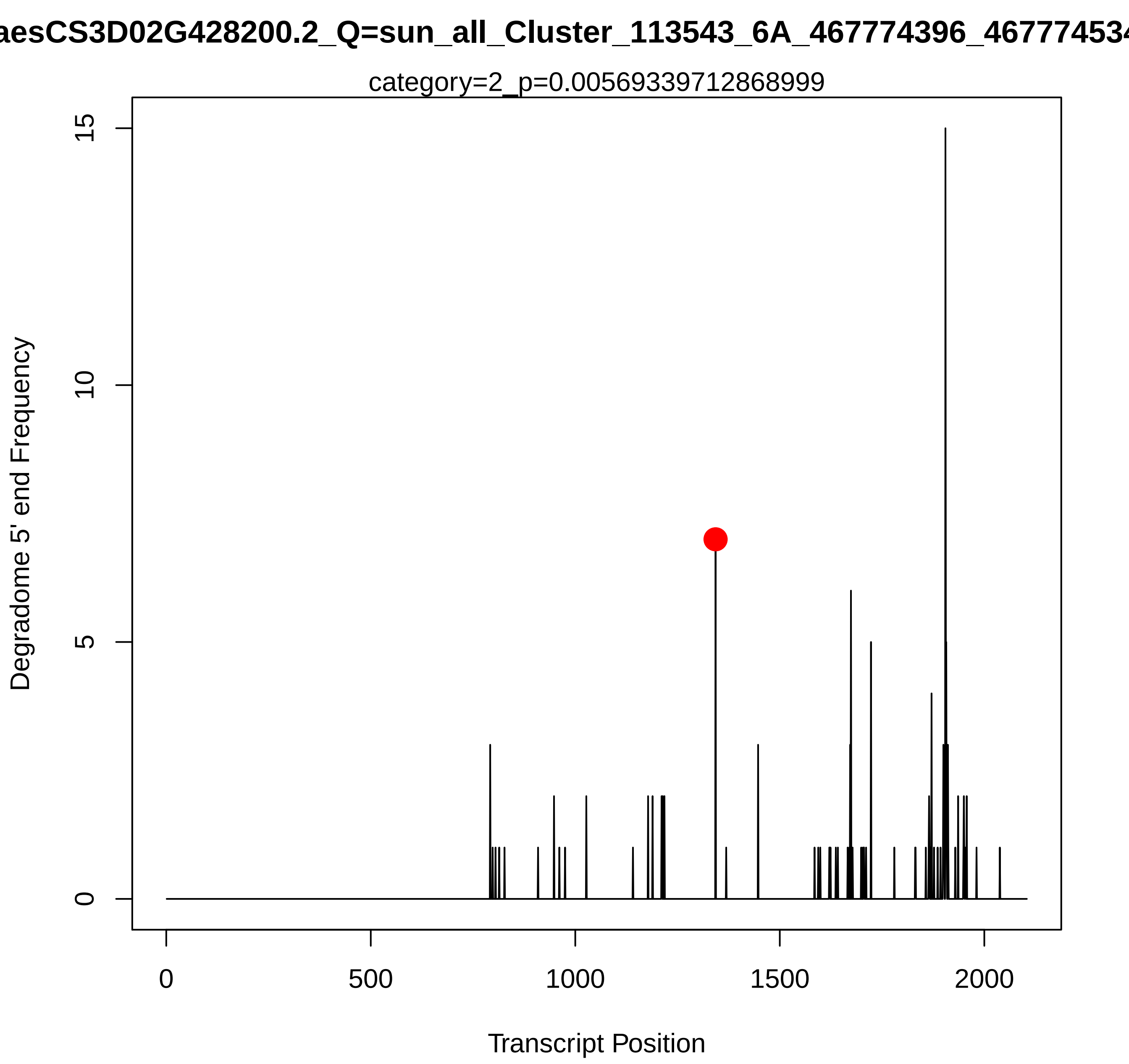

aesCS3D02G428200.2_Q=sun_all_Cluster_113543_6A_467774396_46777453
category=2_p=0.00569339712868999
Degradome 5' end Frequency
Transcript Position
0
5
10
15
0
500
1000
1500
2000

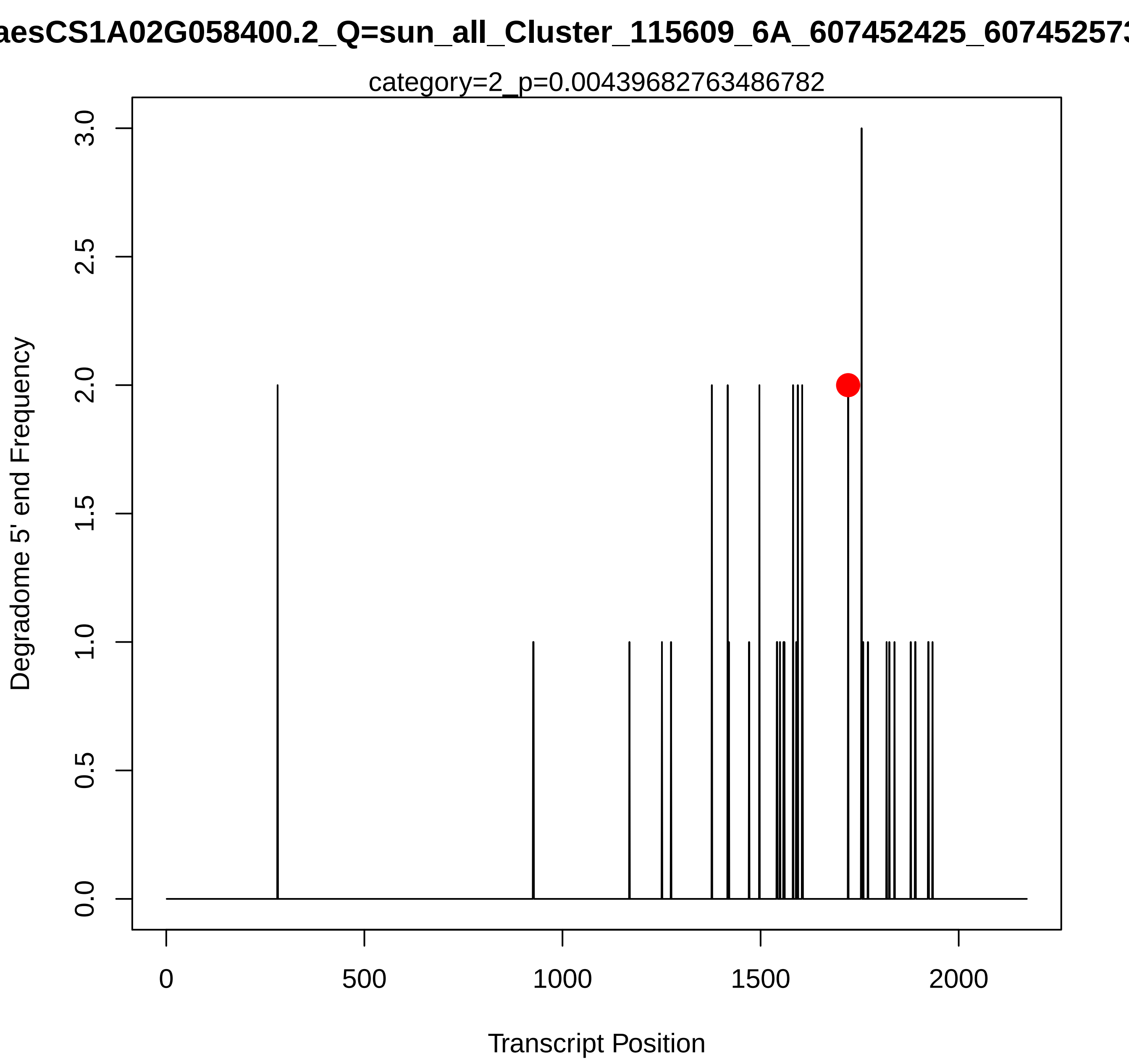

aesCS1A02G058400.2_Q=sun_all_Cluster_115609_6A_607452425_60745257
category=2_p=0.00439682763486782
Degradome 5' end Frequency
Transcript Position
0.0
0.5
1.0
1.5
2.0
2.5
3.0
0
500
1000
1500
2000

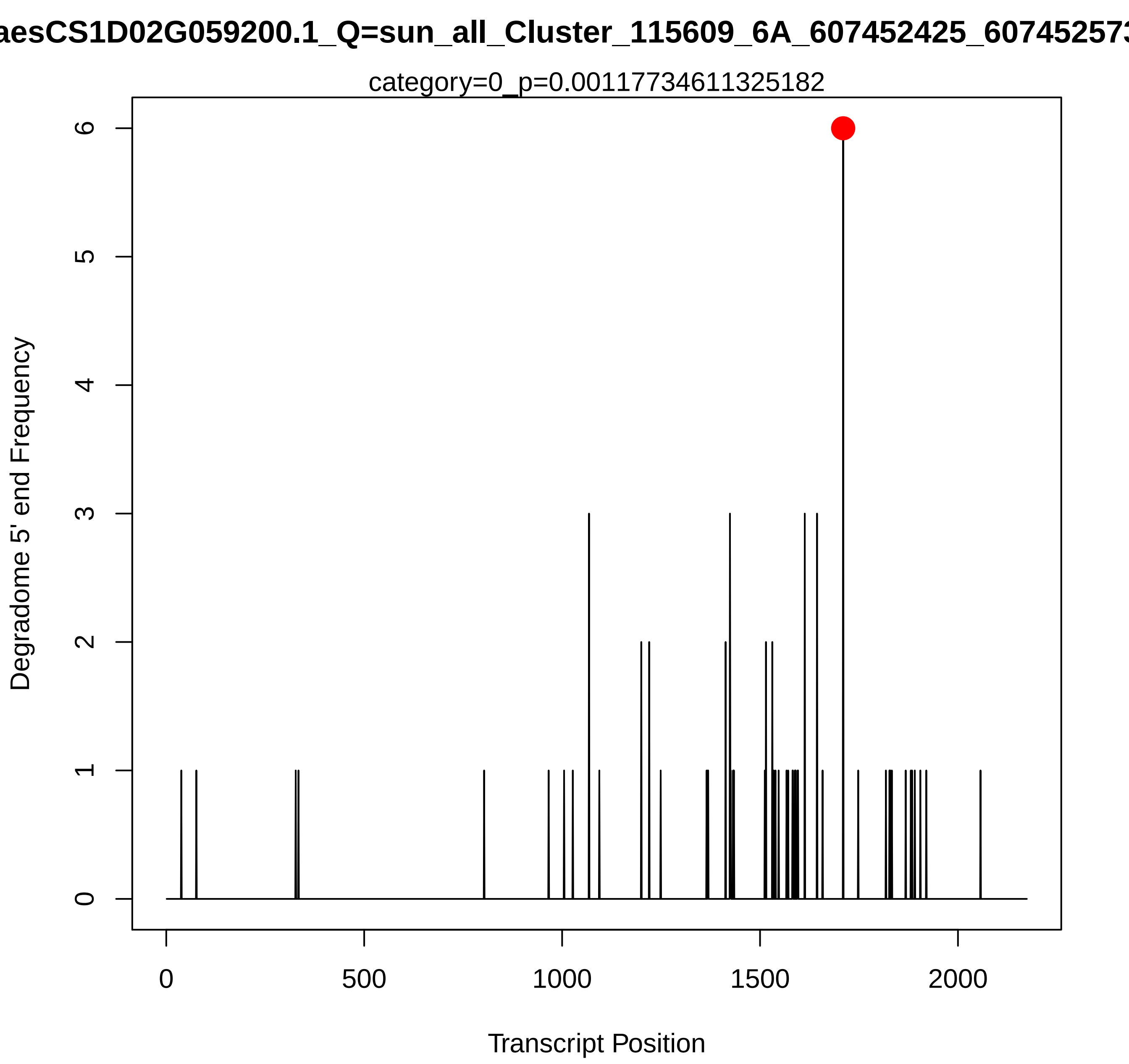

aesCS1D02G059200.1_Q=sun_all_Cluster_115609_6A_607452425_60745257
category=0_p=0.00117734611325182
Degradome 5' end Frequency
0
1
2
3
4
5
6
0
500
1000
1500
2000
Transcript Position

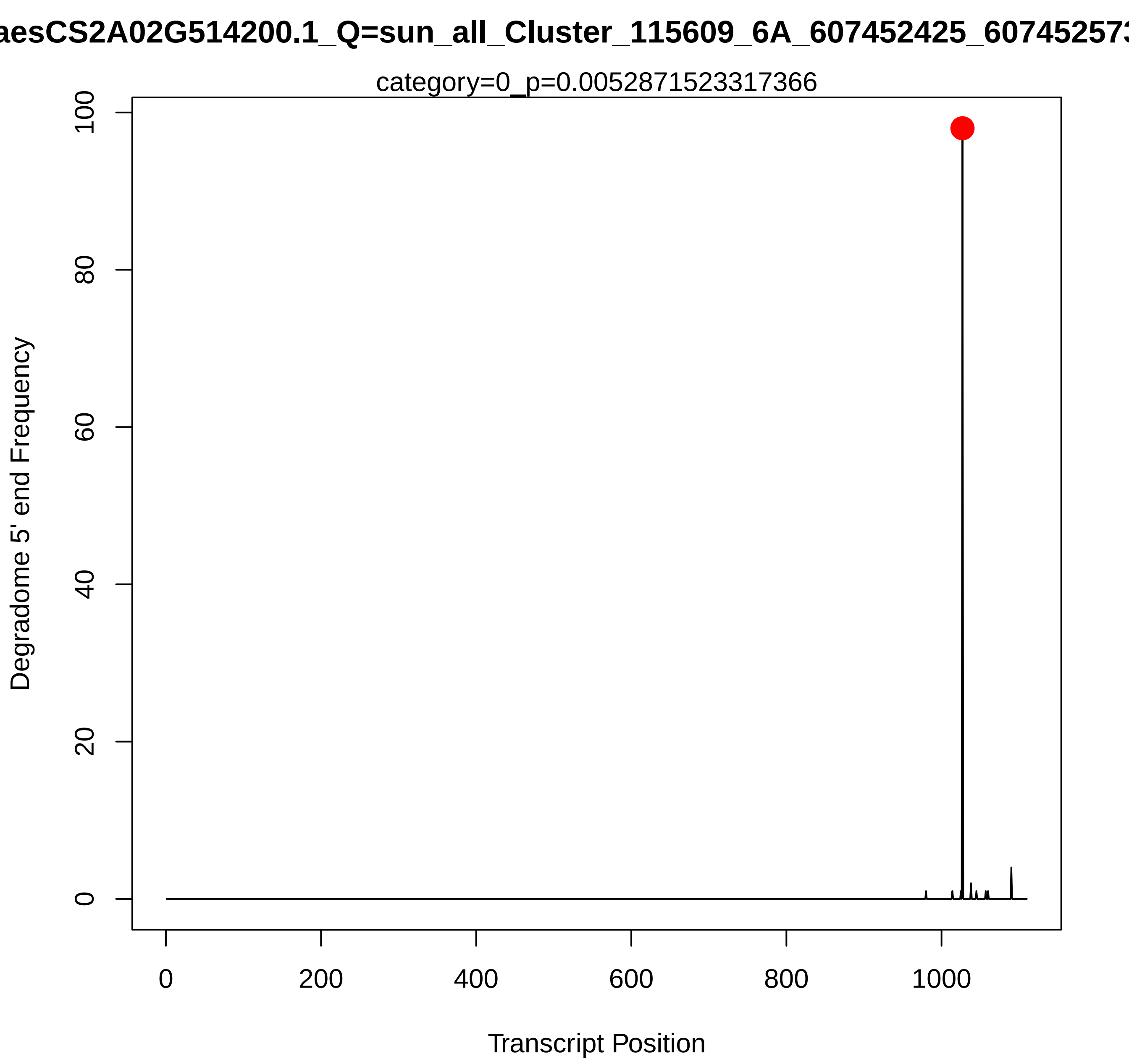
aesCS2A02G514200.1_Q=sun_all_Cluster_115609_6A_607452425_60745257
category=0_p=0.0052871523317366
Degradome 5' end Frequency
Transcript Position
0
20
40
60
80
100
0
200
400
600
800
1000

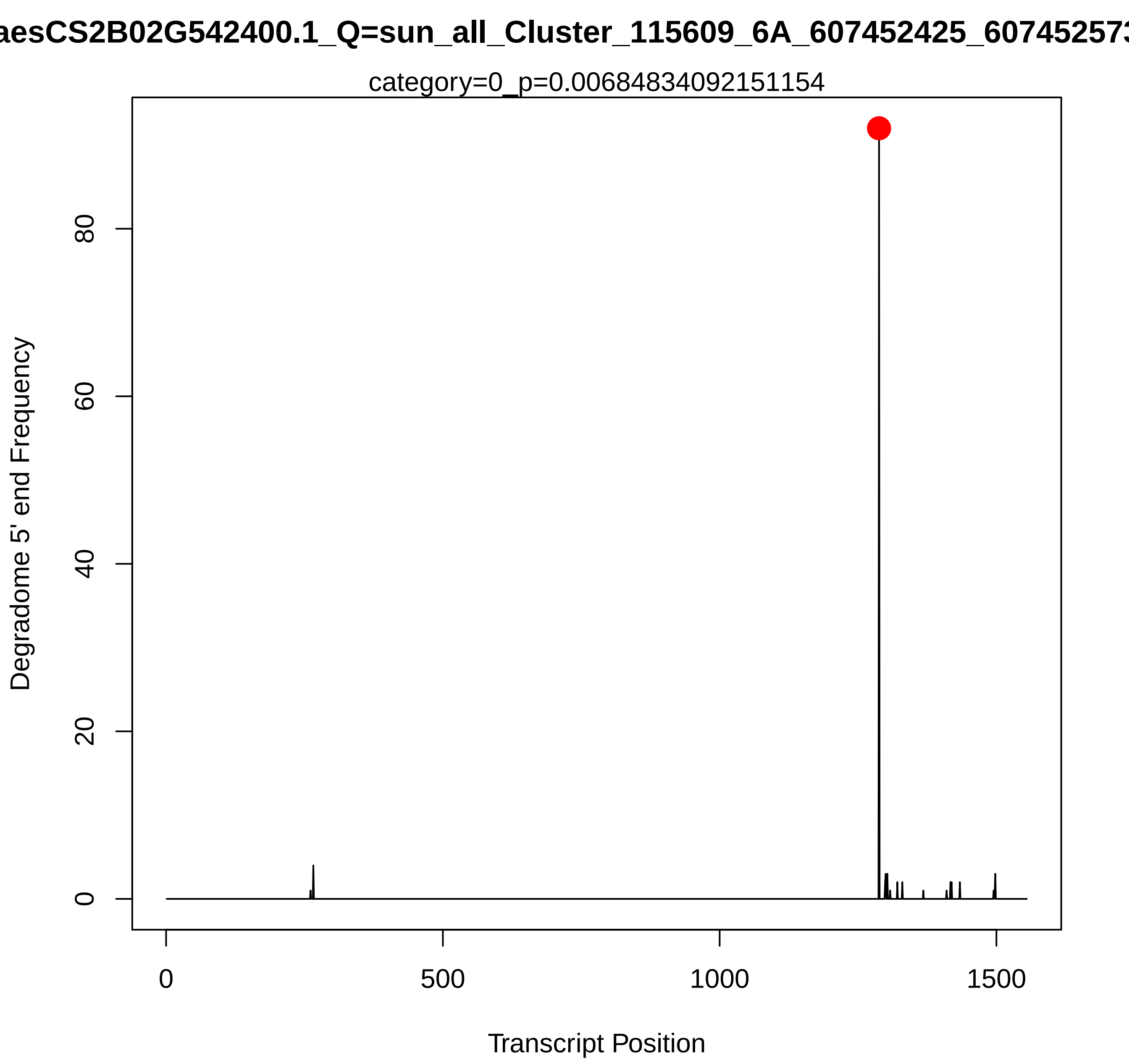

aesCS2B02G542400.1_Q=sun_all_Cluster_115609_6A_607452425_60745257
category=0_p=0.00684834092151154
Degradome 5' end Frequency
0
20
40
60
80
0
500
1000
1500
Transcript Position

aesCS2B02G542400.2_Q=sun_all_Cluster_115609_6A_607452425_60745257[illegible]

category=0_p=0.00792335113185516

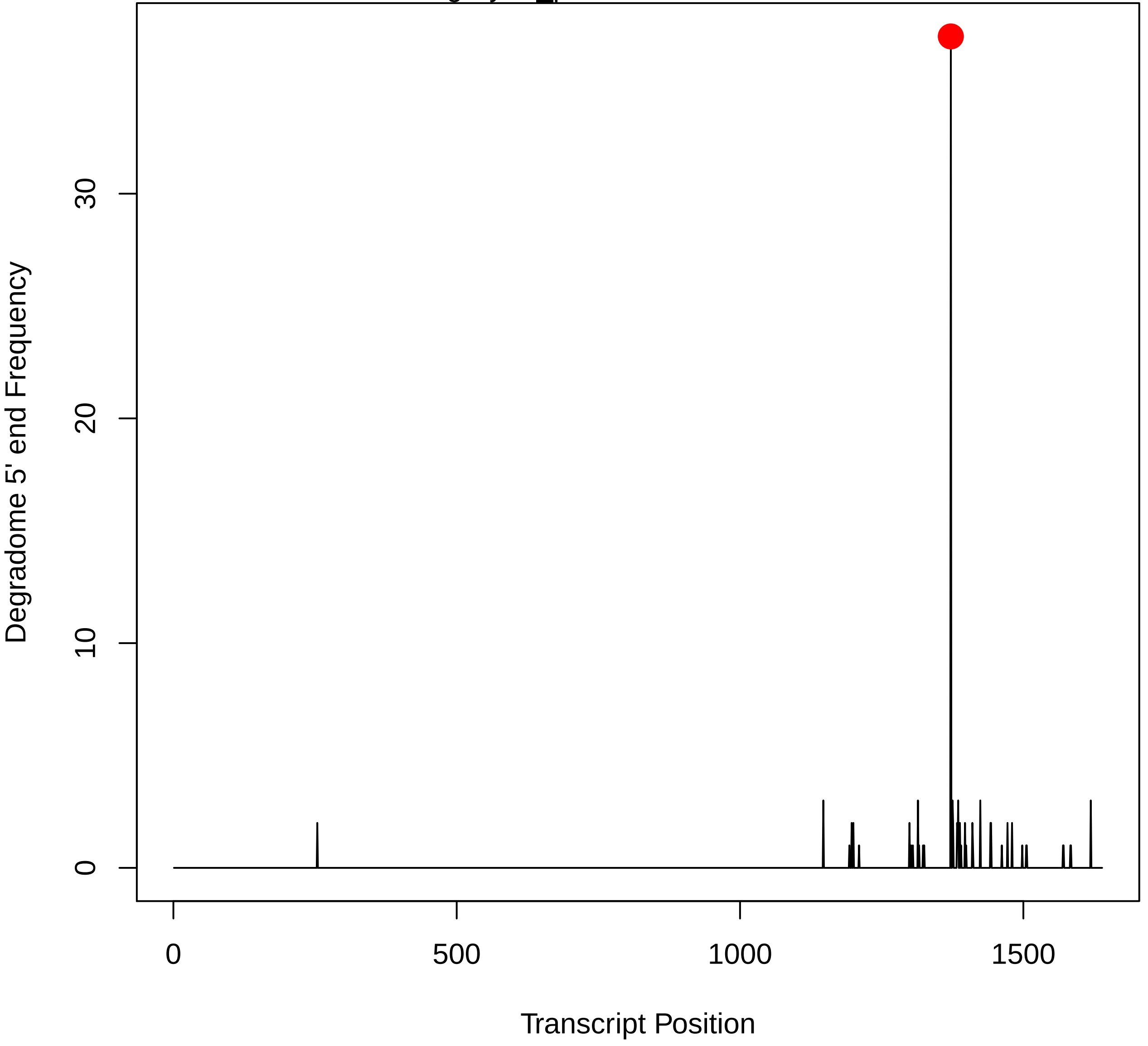

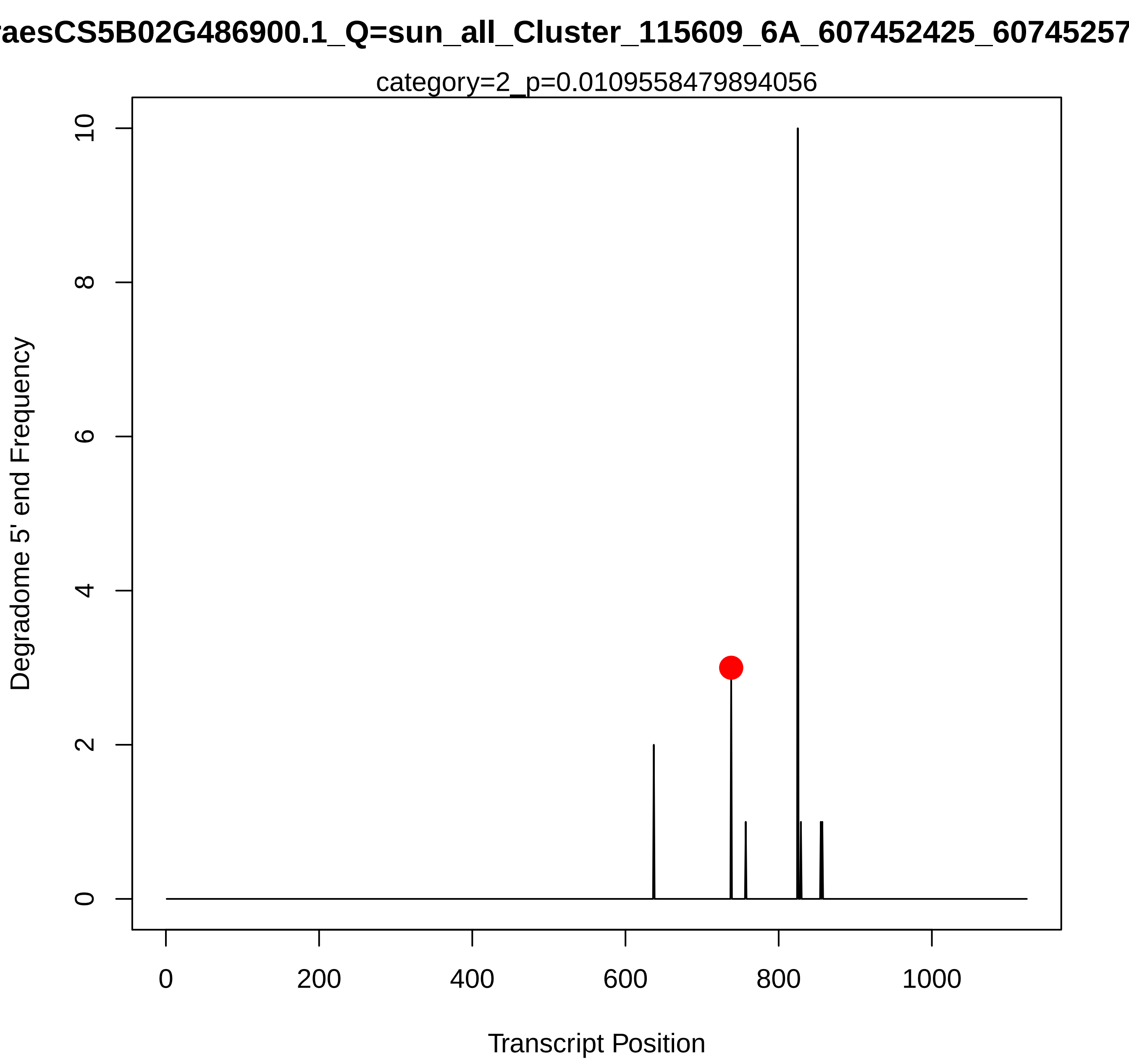
aesCS5B02G486900.1_Q=sun_all_Cluster_115609_6A_607452425_60745257
category=2_p=0.0109558479894056
Degradome 5' end Frequency
0
2
4
6
8
10
0
200
400
600
800
1000
Transcript Position

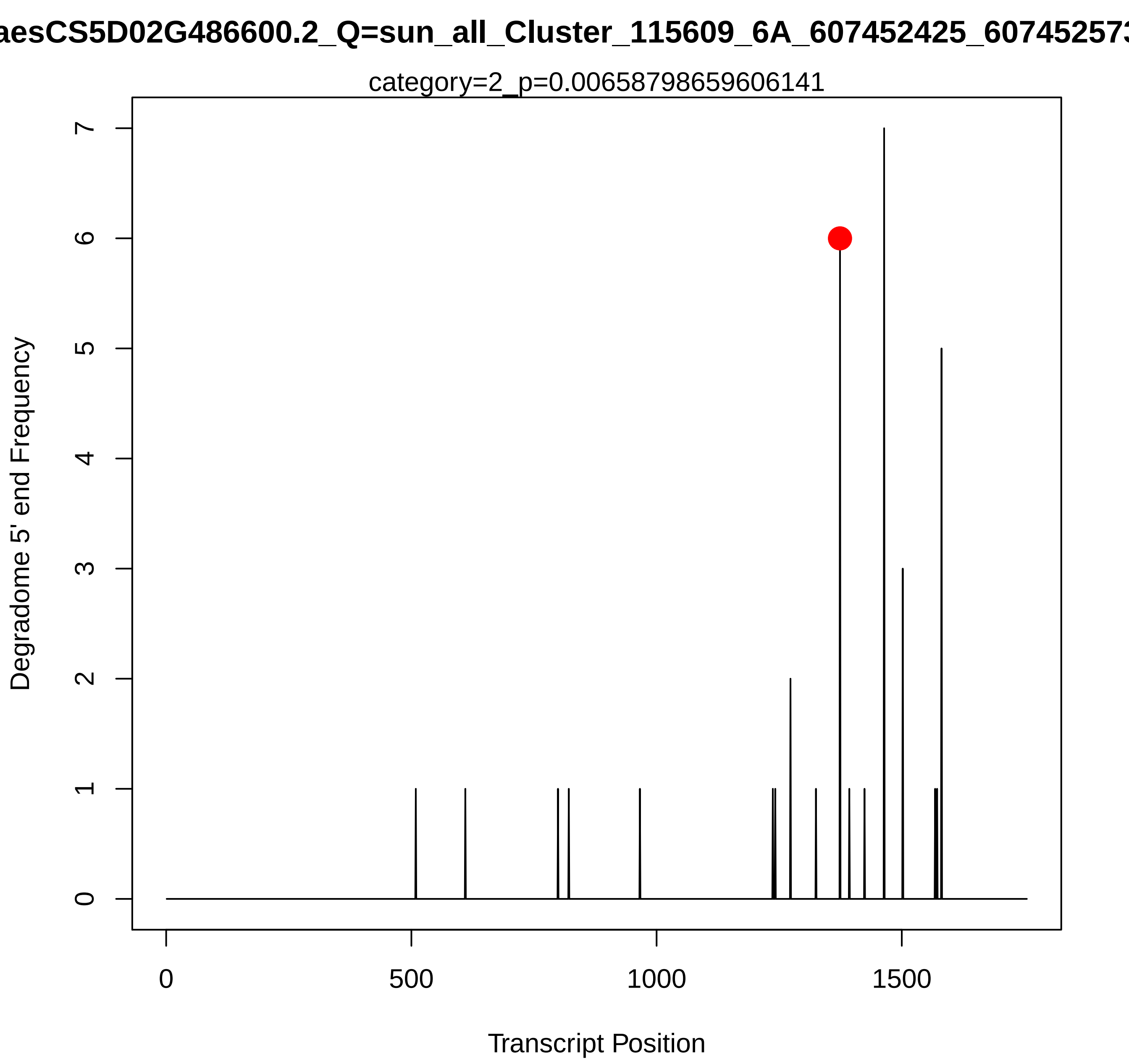
aesCS5D02G486600.2_Q=sun_all_Cluster_115609_6A_607452425_60745257
category=2_p=0.00658798659606141
Degradome 5' end Frequency
Transcript Position
0
1
2
3
4
5
6
7
0
500
1000
1500

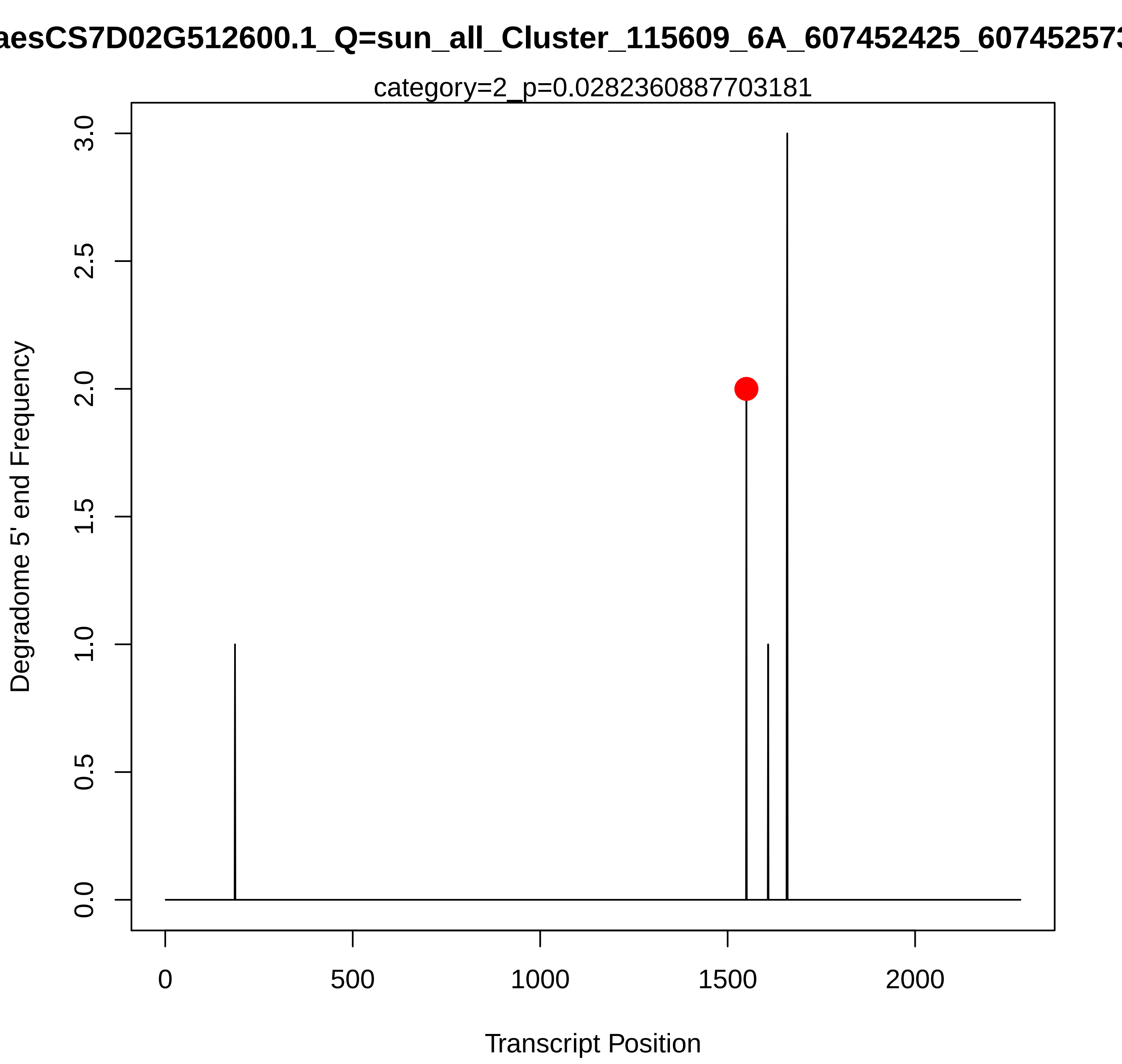
aesCS7D02G512600.1_Q=sun_all_Cluster_115609_6A_607452425_60745257
category=2_p=0.0282360887703181
Degradome 5' end Frequency
0.0
0.5
1.0
1.5
2.0
2.5
3.0
0
500
1000
1500
2000
Transcript Position

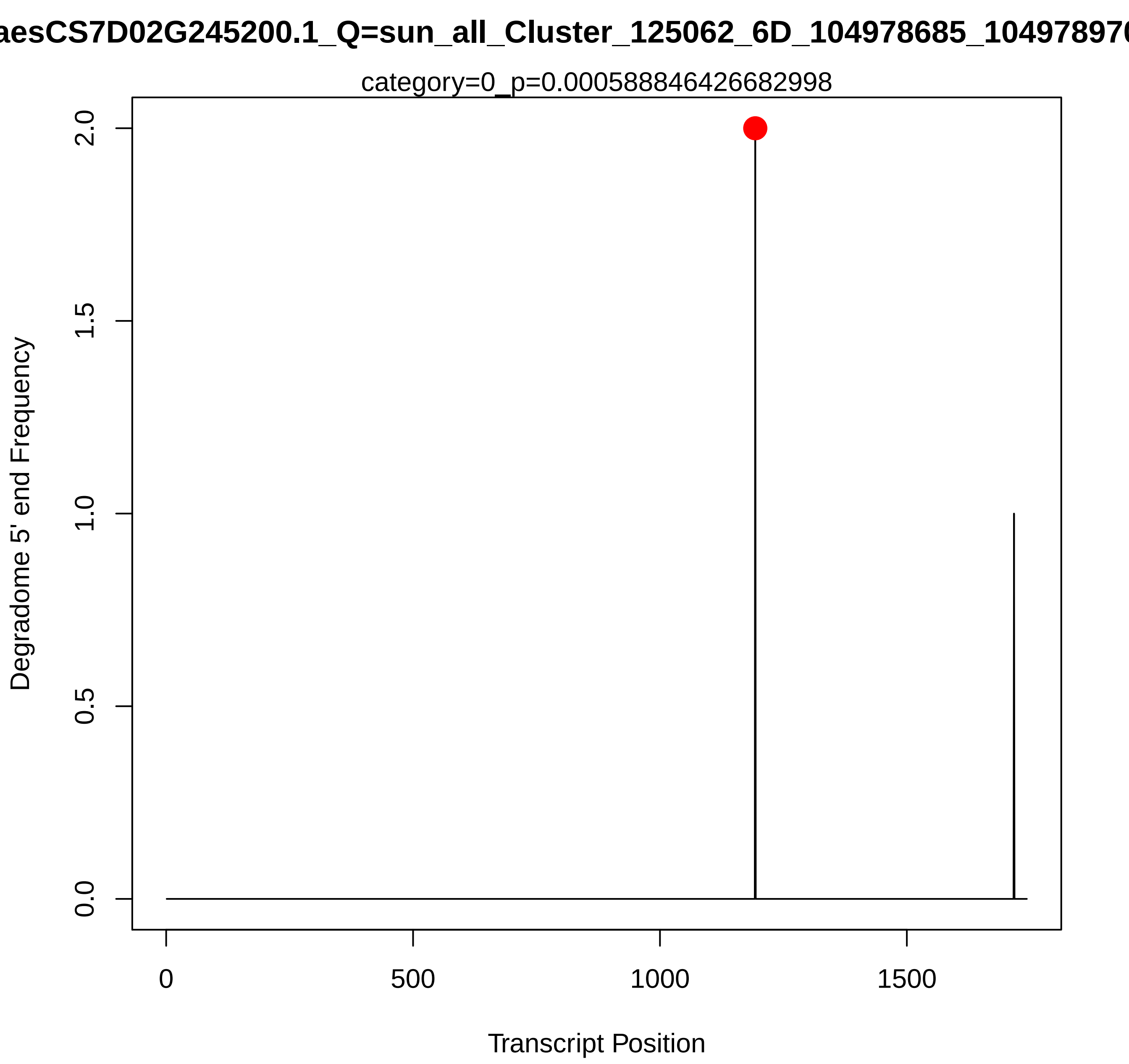
aesCS7D02G245200.1_Q=sun_all_Cluster_125062_6D_104978685_10497897
category=0_p=0.000588846426682998
Degradome 5' end Frequency
Transcript Position
0.0
0.5
1.0
1.5
2.0
0
500
1000
1500

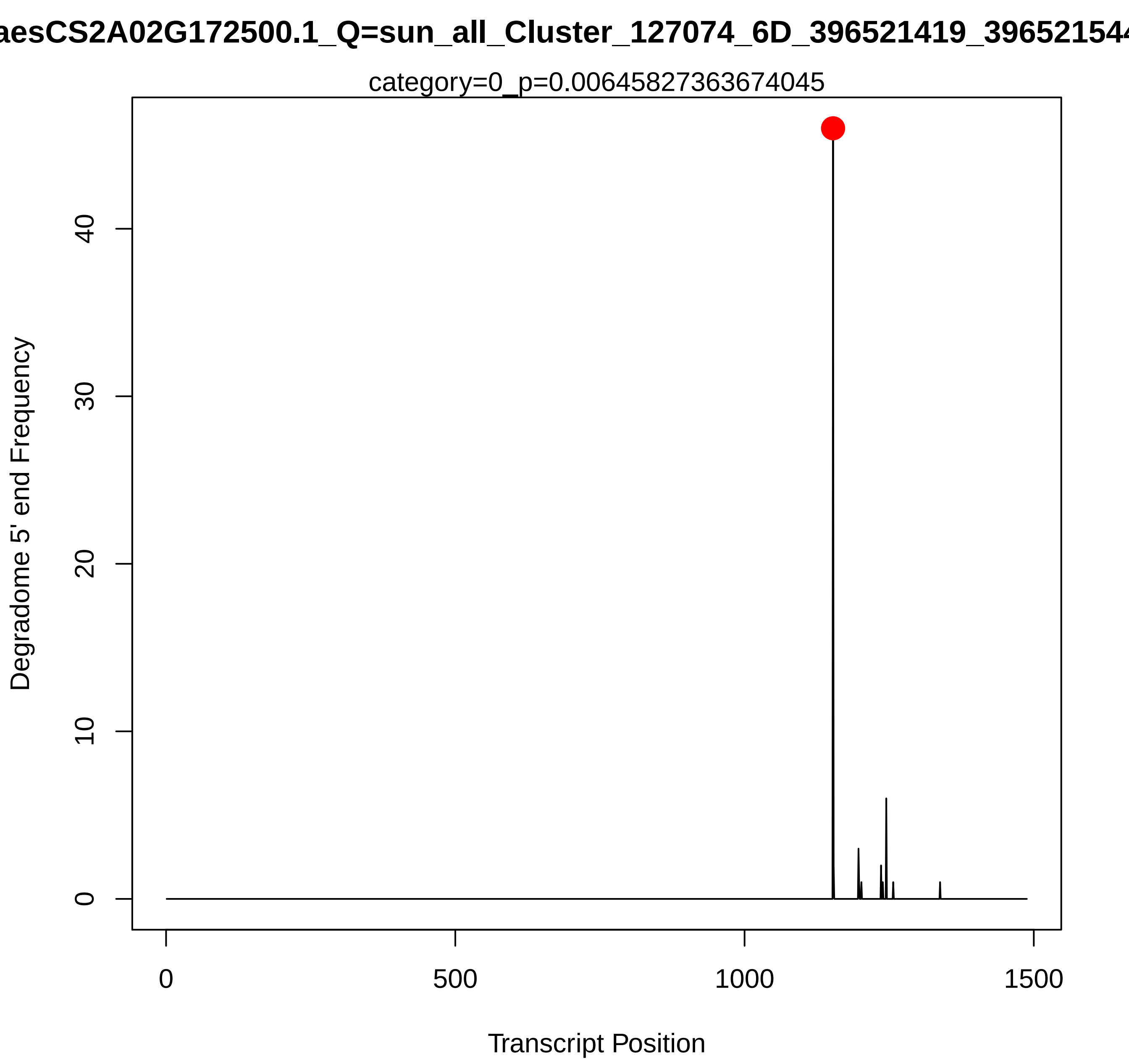
aesCS2A02G172500.1_Q=sun_all_Cluster_127074_6D_396521419_39652154
category=0_p=0.00645827363674045
Degradome 5' end Frequency
0
10
20
30
40
0
500
1000
1500
Transcript Position

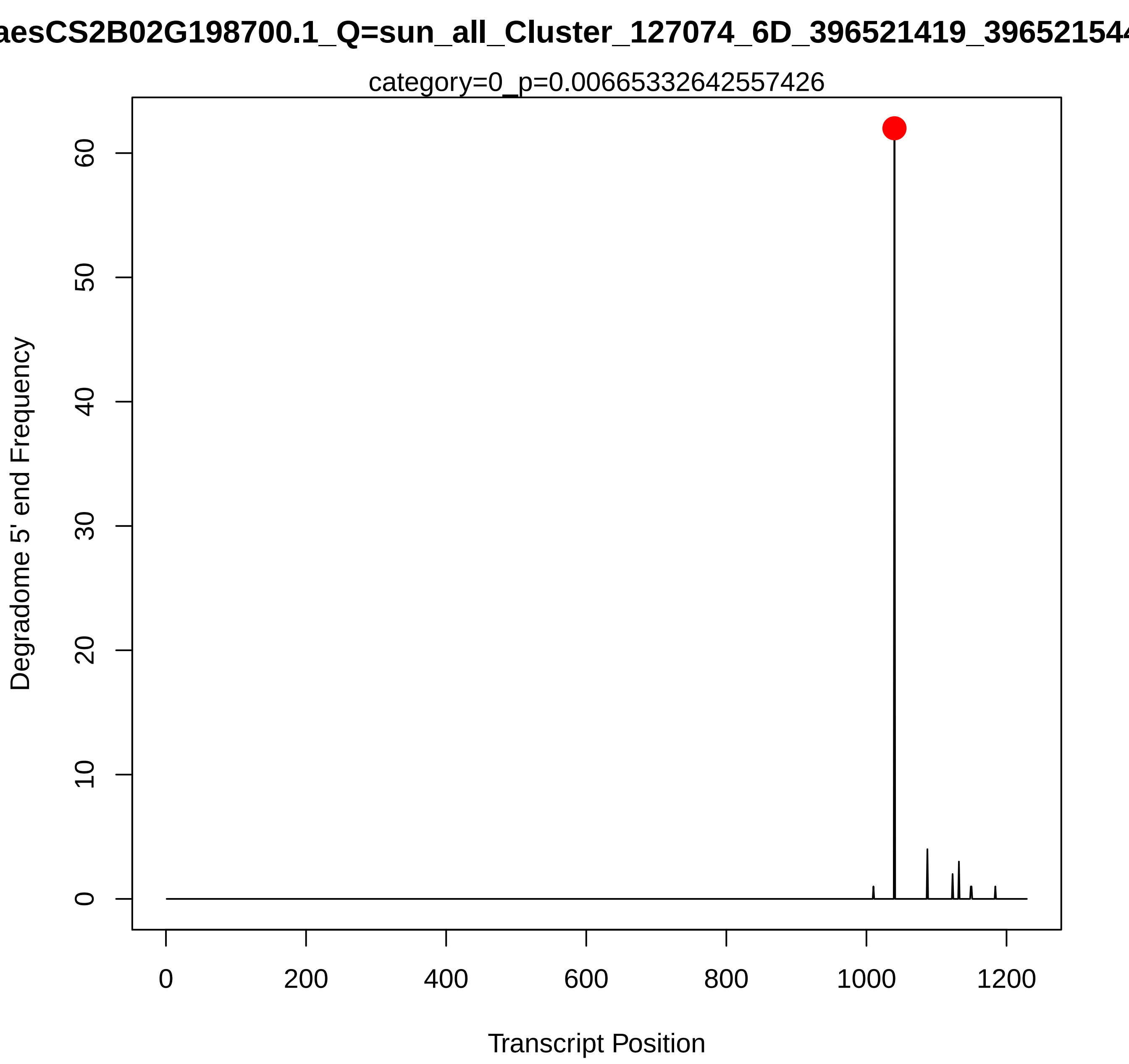
aesCS2B02G198700.1_Q=sun_all_Cluster_127074_6D_396521419_39652154
category=0_p=0.00665332642557426
Degradome 5' end Frequency
0
10
20
30
40
50
60
0
200
400
600
800
1000
1200
Transcript Position

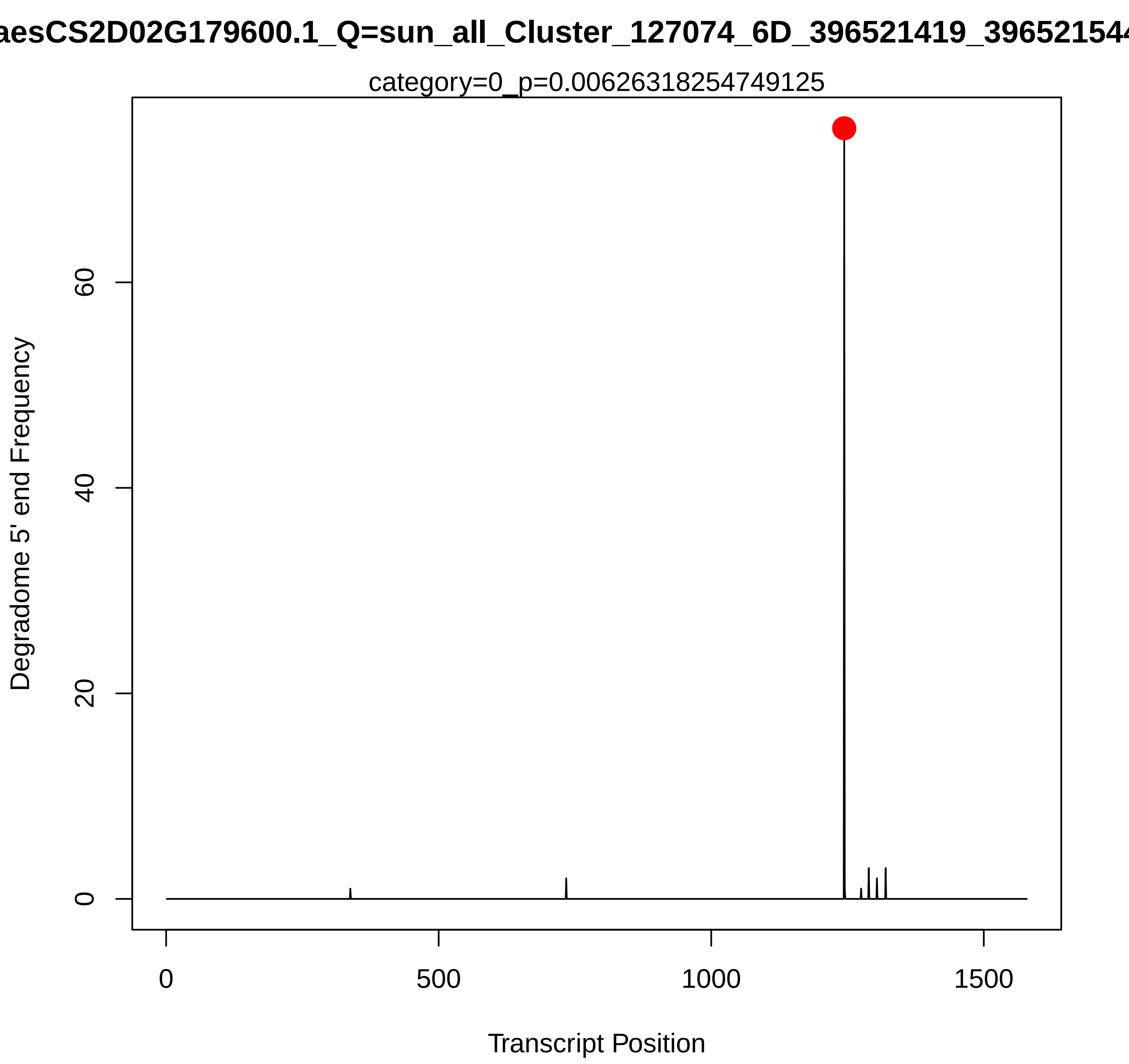

aesCS2D02G179600.1_Q=sun_all_Cluster_127074_6D_396521419_39652154
category=0_p=0.00626318254749125
Degradome 5' end Frequency
0
20
40
60
0
500
1000
1500
Transcript Position

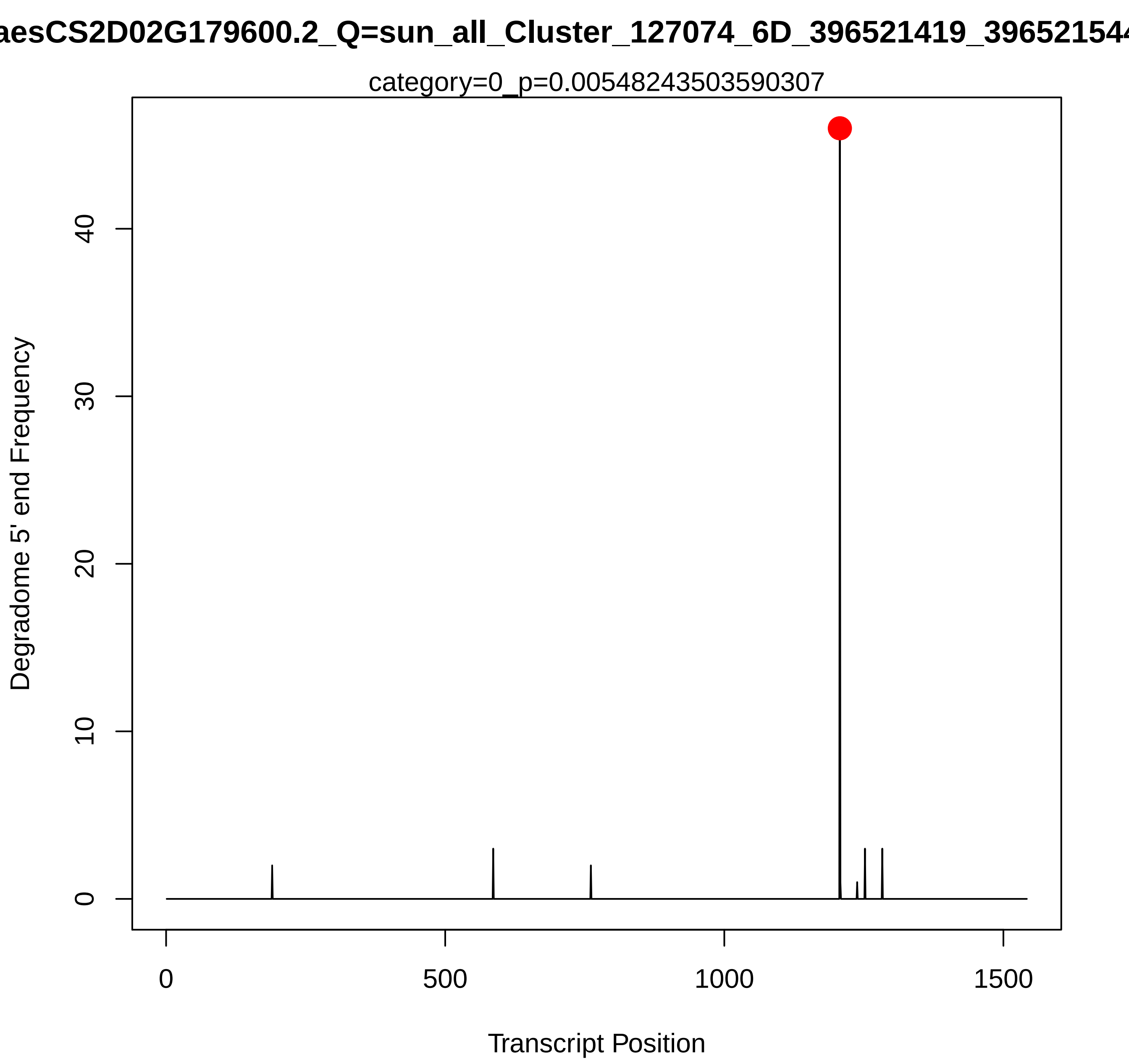

aesCS2D02G179600.2_Q=sun_all_Cluster_127074_6D_396521419_39652154
category=0_p=0.00548243503590307
Degradome 5' end Frequency
0
10
20
30
40
0
500
1000
1500
Transcript Position

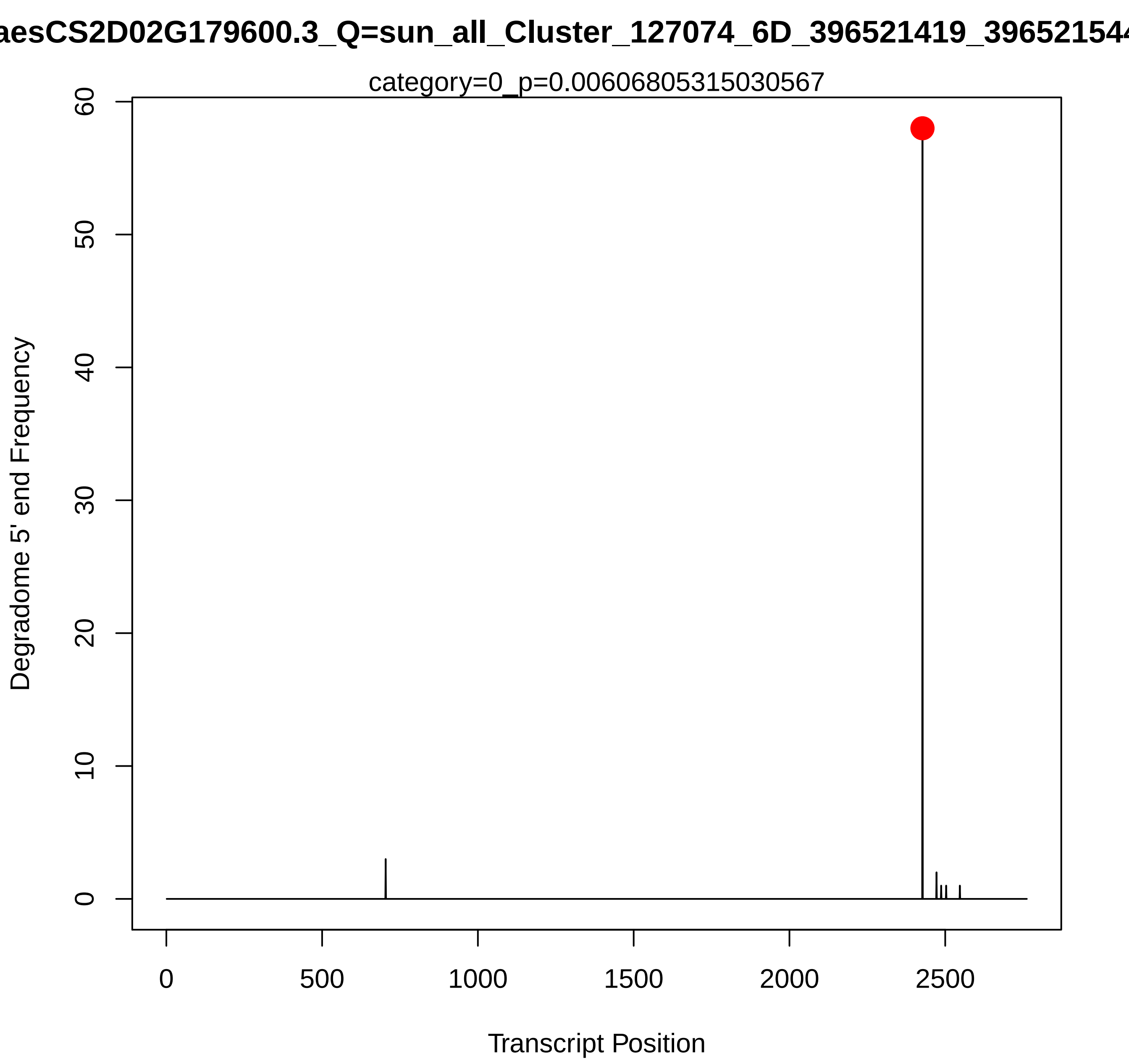

aesCS2D02G179600.3_Q=sun_all_Cluster_127074_6D_396521419_39652154
category=0_p=0.00606805315030567
Degradome 5' end Frequency
0
10
20
30
40
50
60
Transcript Position
0
500
1000
1500
2000
2500

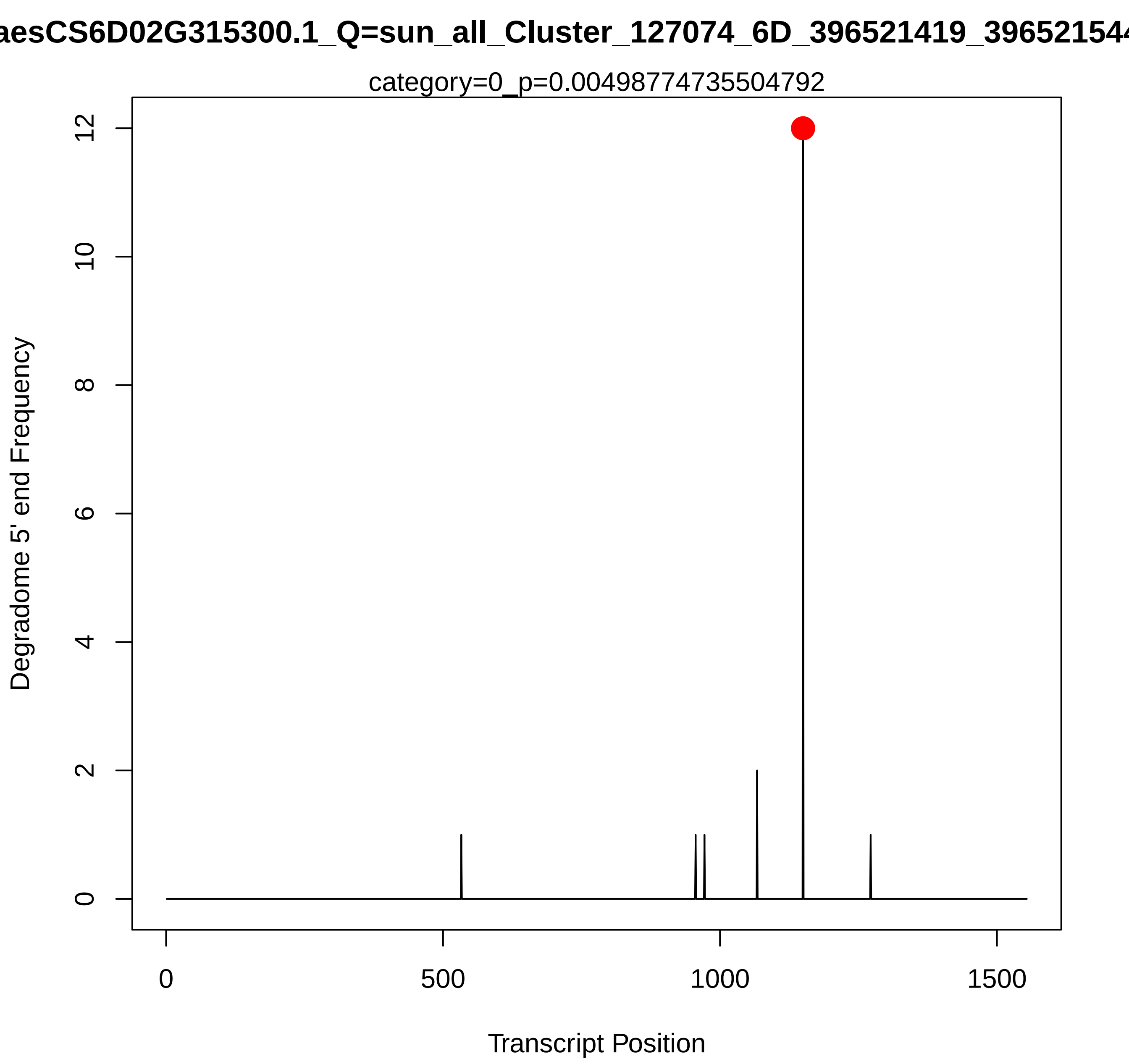
aesCS6D02G315300.1_Q=sun_all_Cluster_127074_6D_396521419_39652154
category=0_p=0.00498774735504792
Degradome 5' end Frequency
0
2
4
6
8
10
12
0
500
1000
1500
Transcript Position

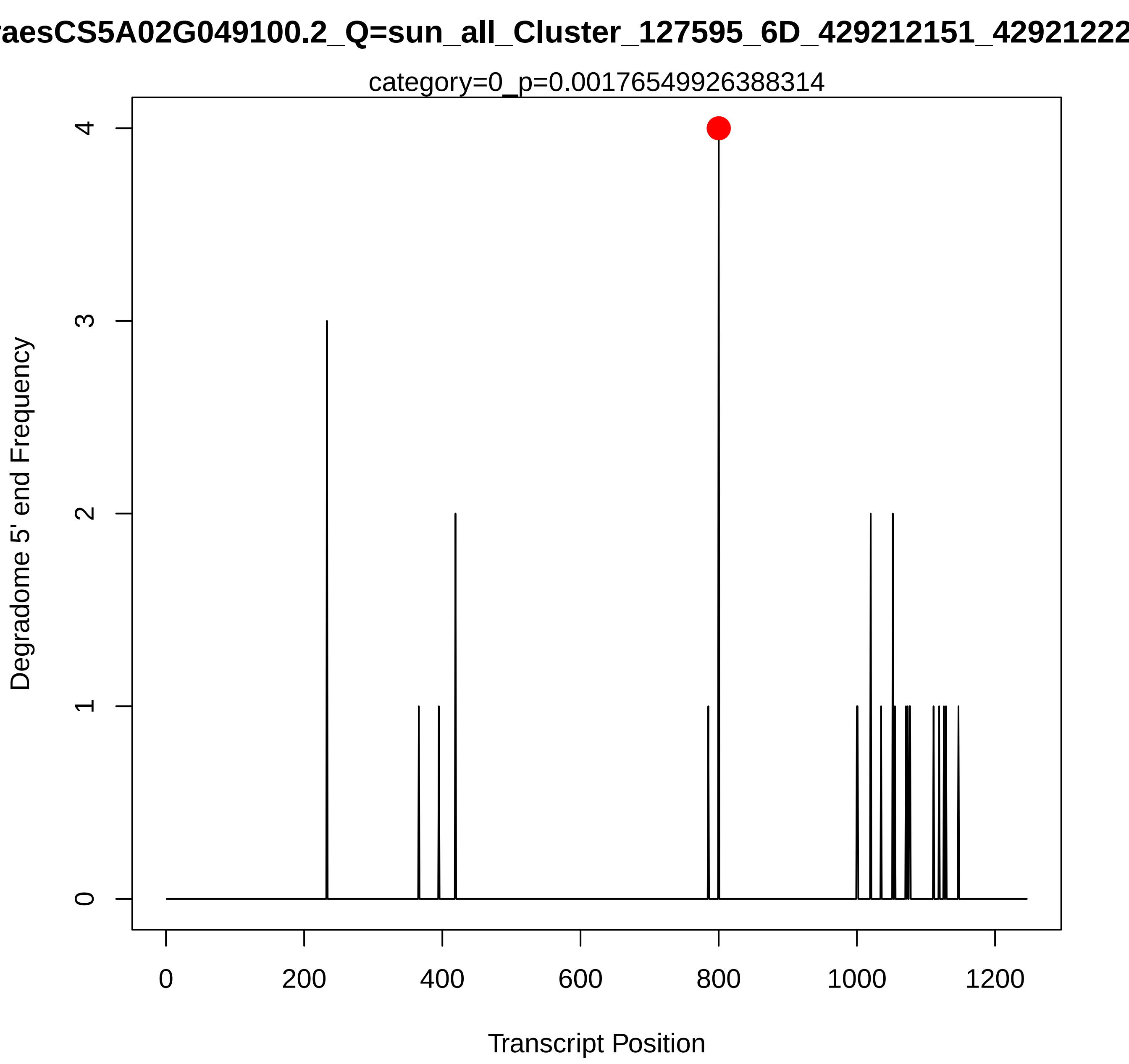

raesCS5A02G049100.2_Q=sun_all_Cluster_127595_6D_429212151_42921222
category=0_p=0.00176549926388314
Degradome 5' end Frequency
Transcript Position
0
1
2
3
4
0
200
400
600
800
1000
1200

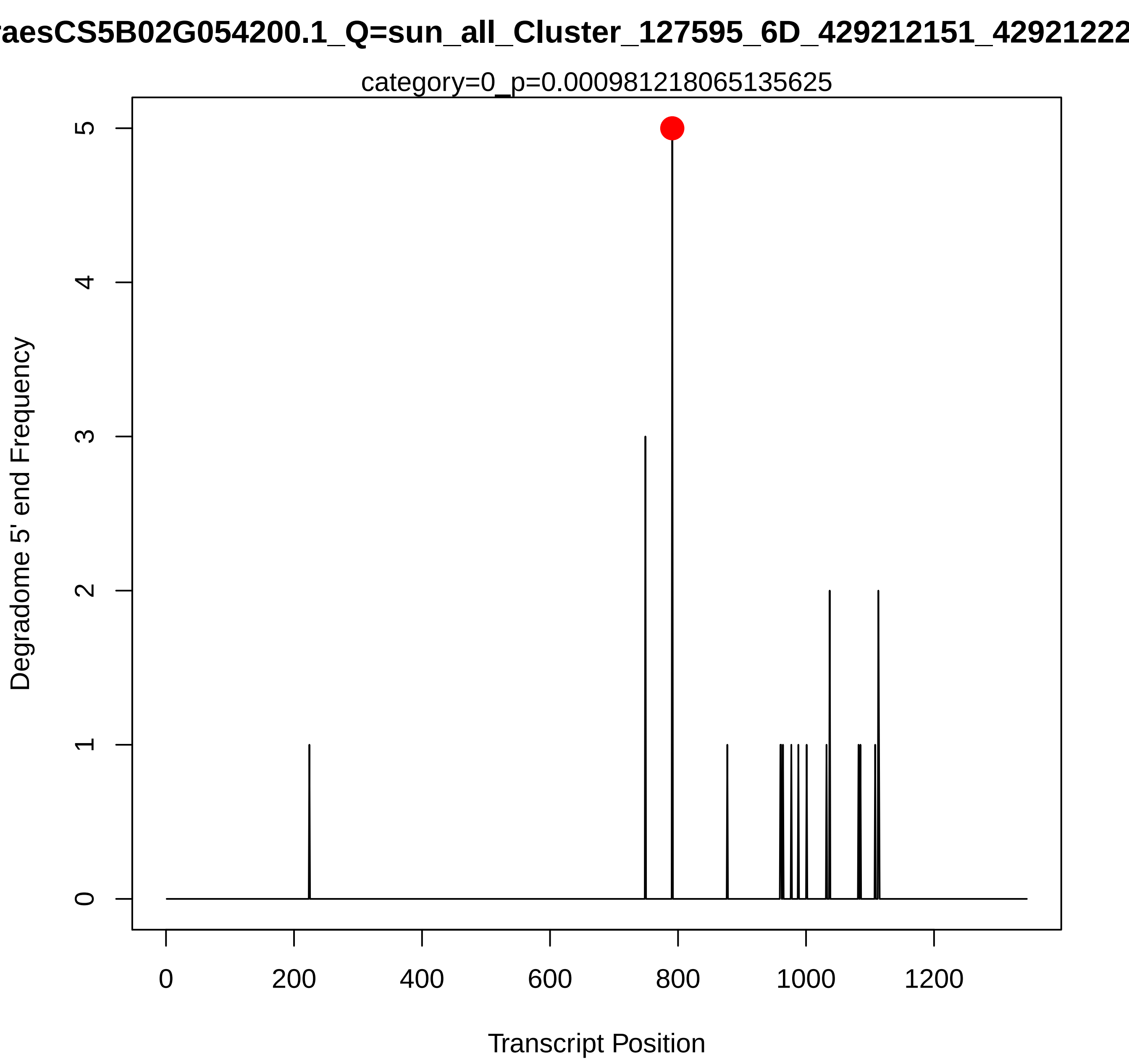

raesCS5B02G054200.1_Q=sun_all_Cluster_127595_6D_429212151_42921222
category=0_p=0.000981218065135625
Degradome 5' end Frequency
0
1
2
3
4
5
0
200
400
600
800
1000
1200
Transcript Position

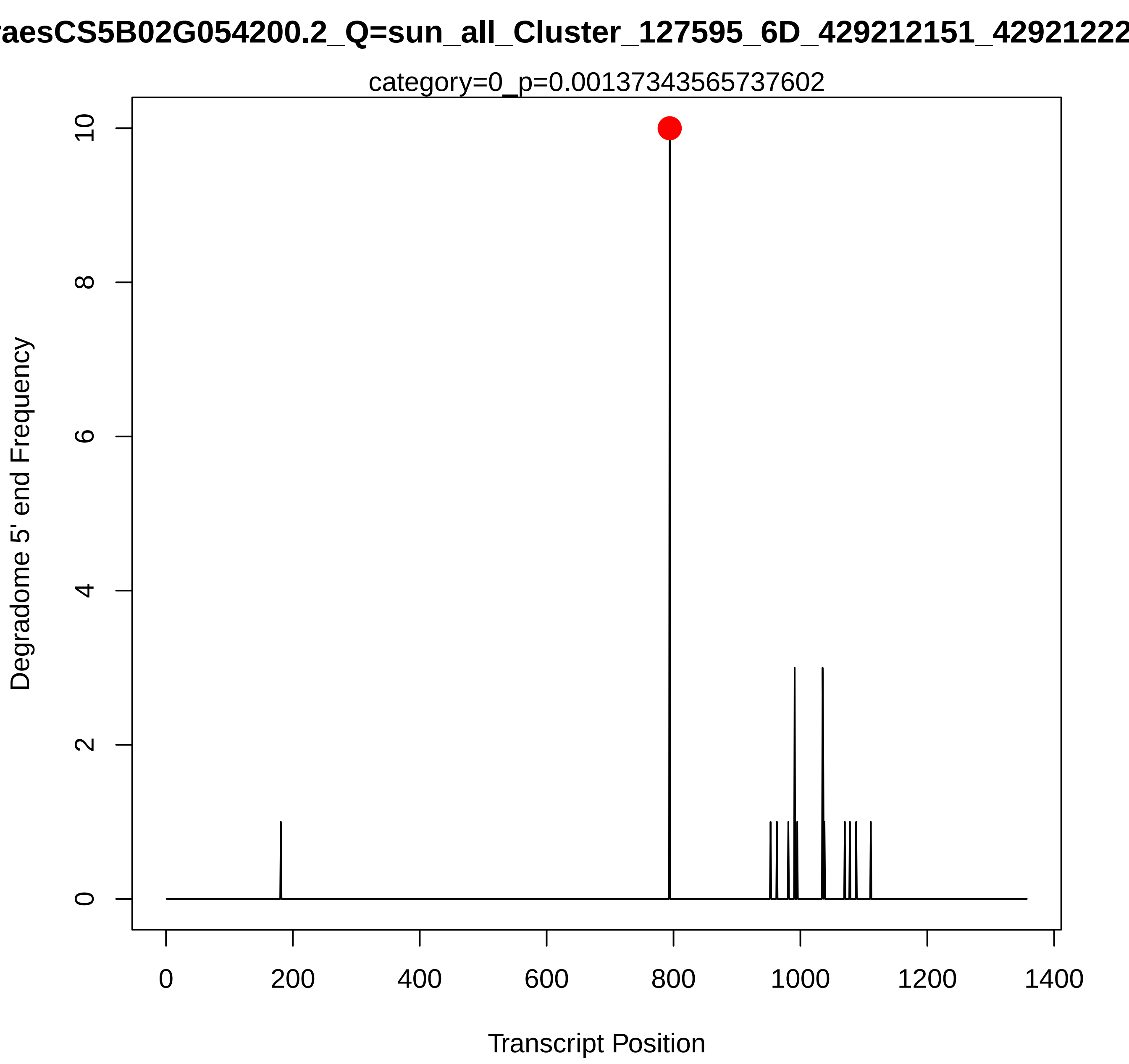

raesCS5B02G054200.2_Q=sun_all_Cluster_127595_6D_429212151_42921222
category=0_p=0.00137343565737602
Degradome 5' end Frequency
0
2
4
6
8
10
0
200
400
600
800
1000
1200
1400
Transcript Position

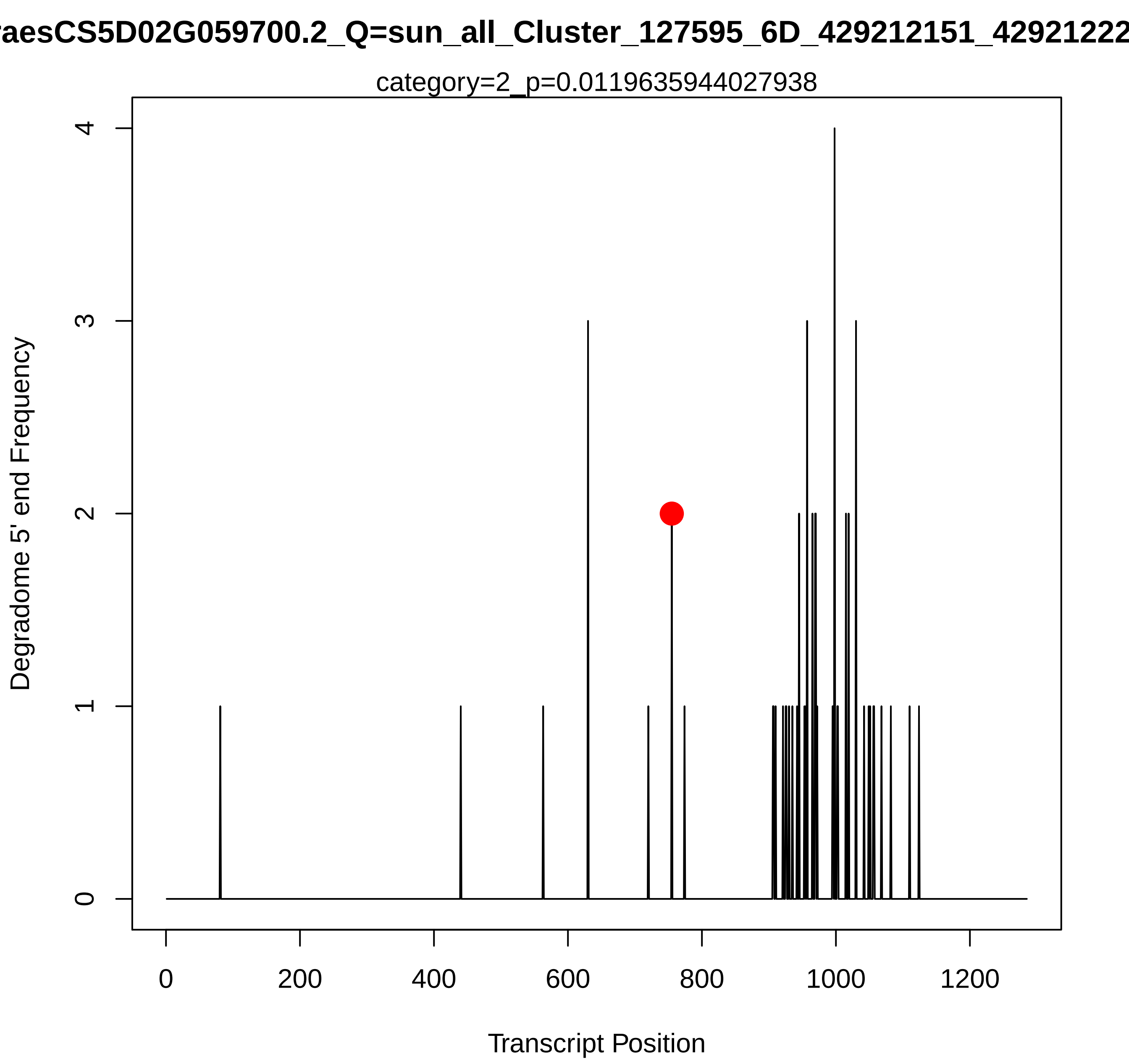

raesCS5D02G059700.2_Q=sun_all_Cluster_127595_6D_429212151_42921222
category=2_p=0.0119635944027938
Degradome 5' end Frequency
0
1
2
3
4
0
200
400
600
800
1000
1200
Transcript Position

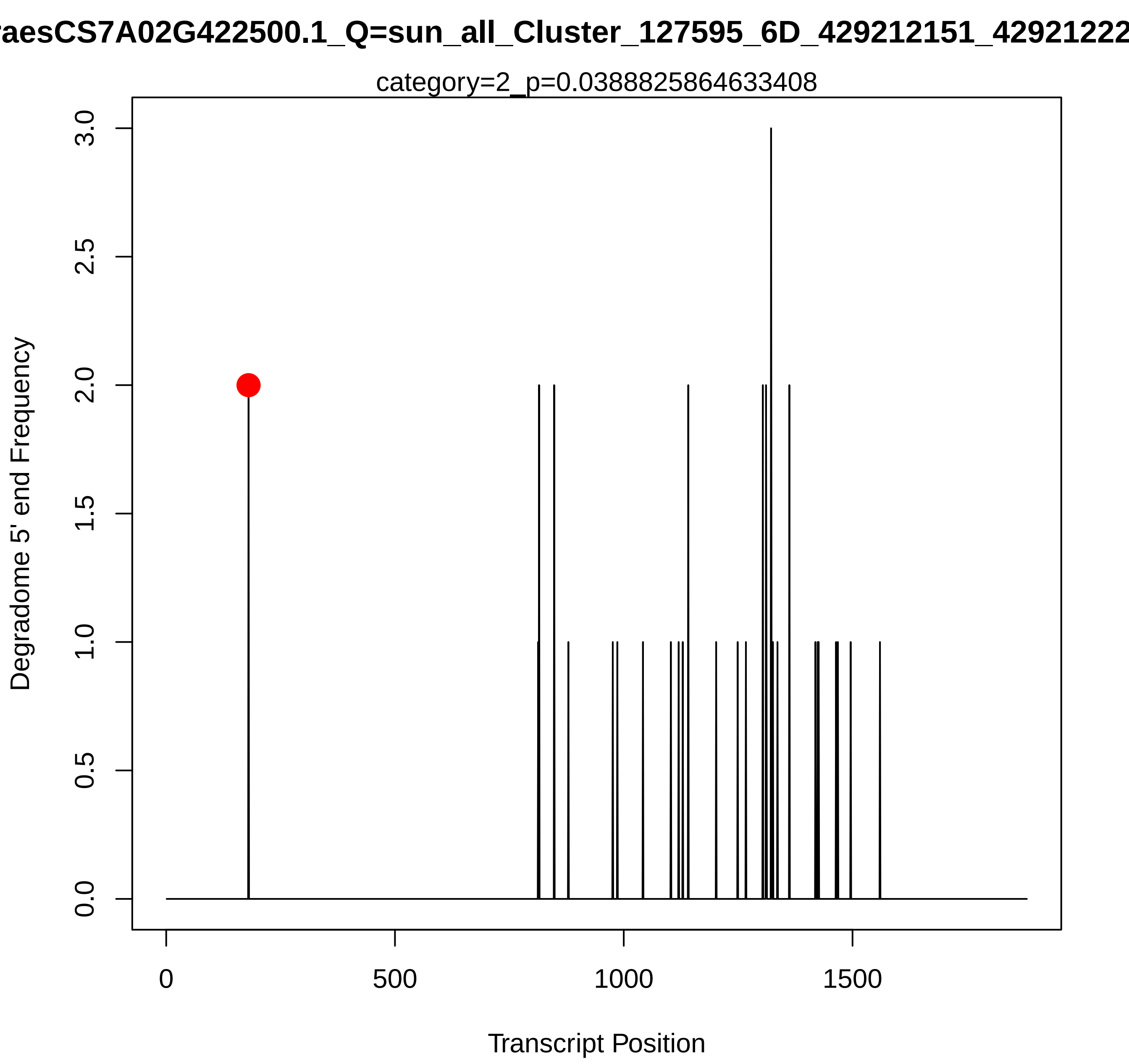
[illegible]aesCS7A02G422500.1_Q=sun_all_Cluster_127595_6D_429212151_42921222[illegible]
category=2_p=0.0388825864633408
Degradome 5' end Frequency
0.0
0.5
1.0
1.5
2.0
2.5
3.0
0
500
1000
1500
Transcript Position

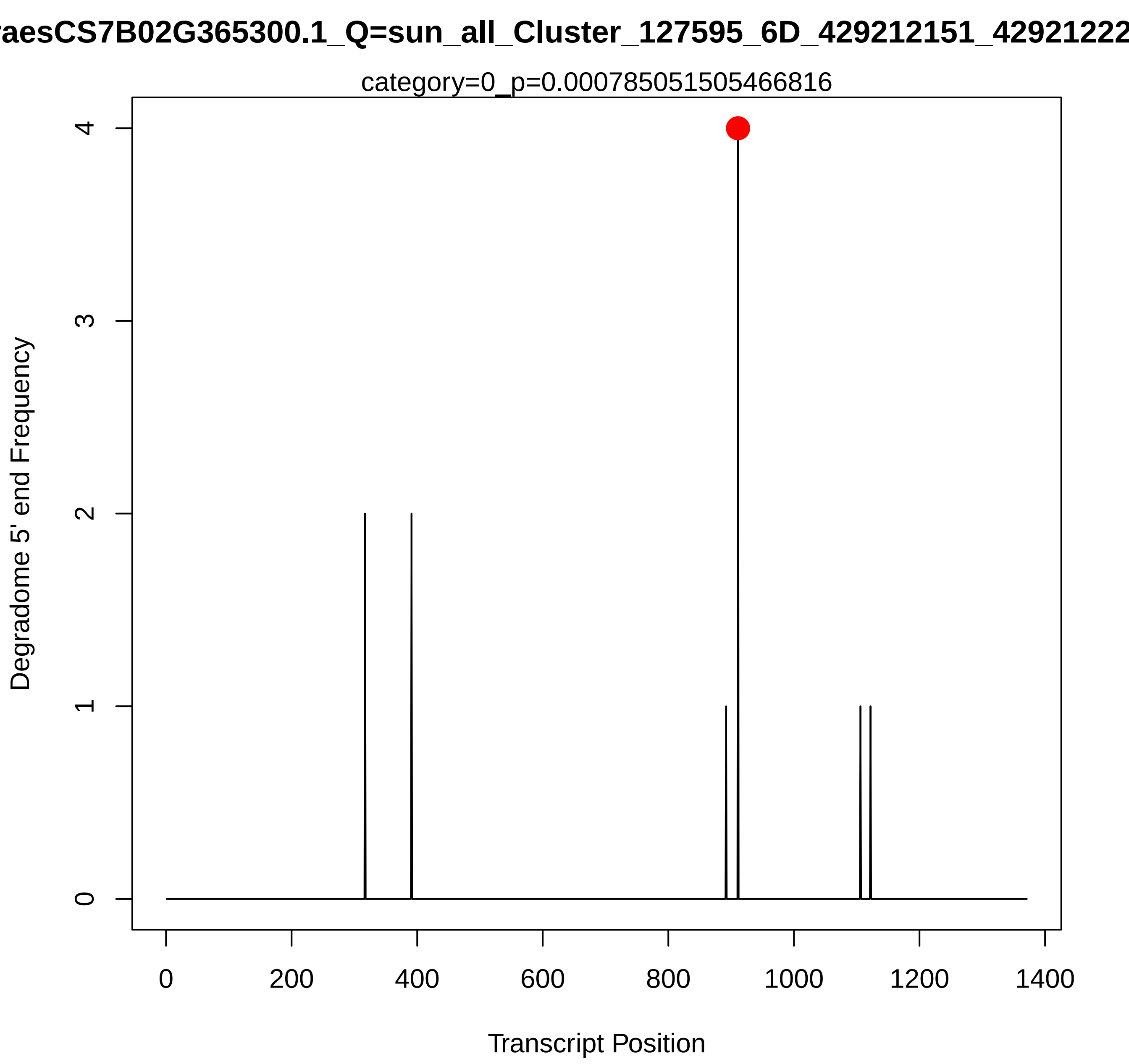

raesCS7B02G365300.1_Q=sun_all_Cluster_127595_6D_429212151_42921222
category=0_p=0.000785051505466816
Degradome 5' end Frequency
0
1
2
3
4
0
200
400
600
800
1000
1200
1400
Transcript Position

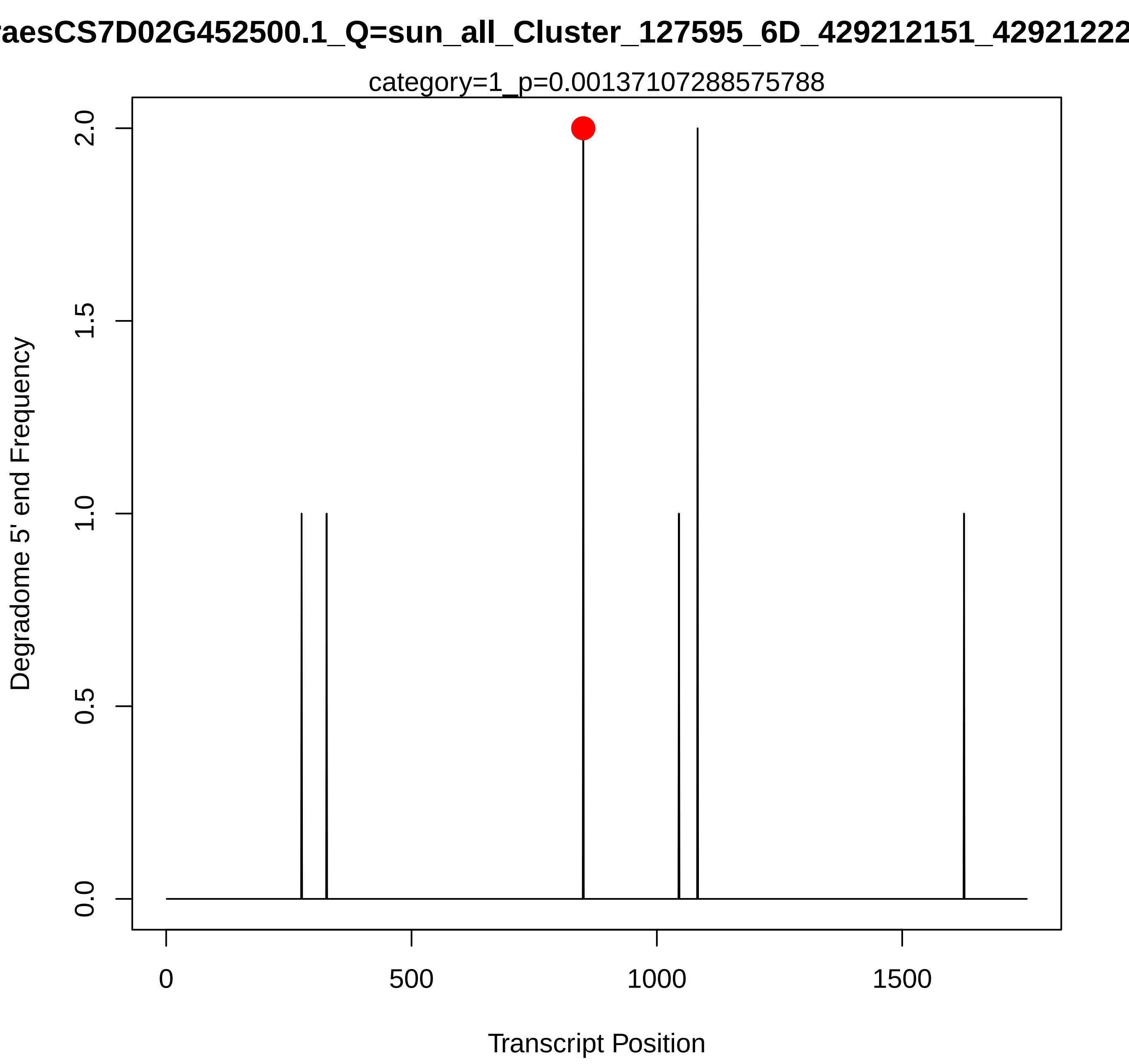

aesCS7D02G452500.1_Q=sun_all_Cluster_127595_6D_429212151_42921222
category=1_p=0.00137107288575788
Degradome 5' end Frequency
0.0
0.5
1.0
1.5
2.0
0
500
1000
1500
Transcript Position

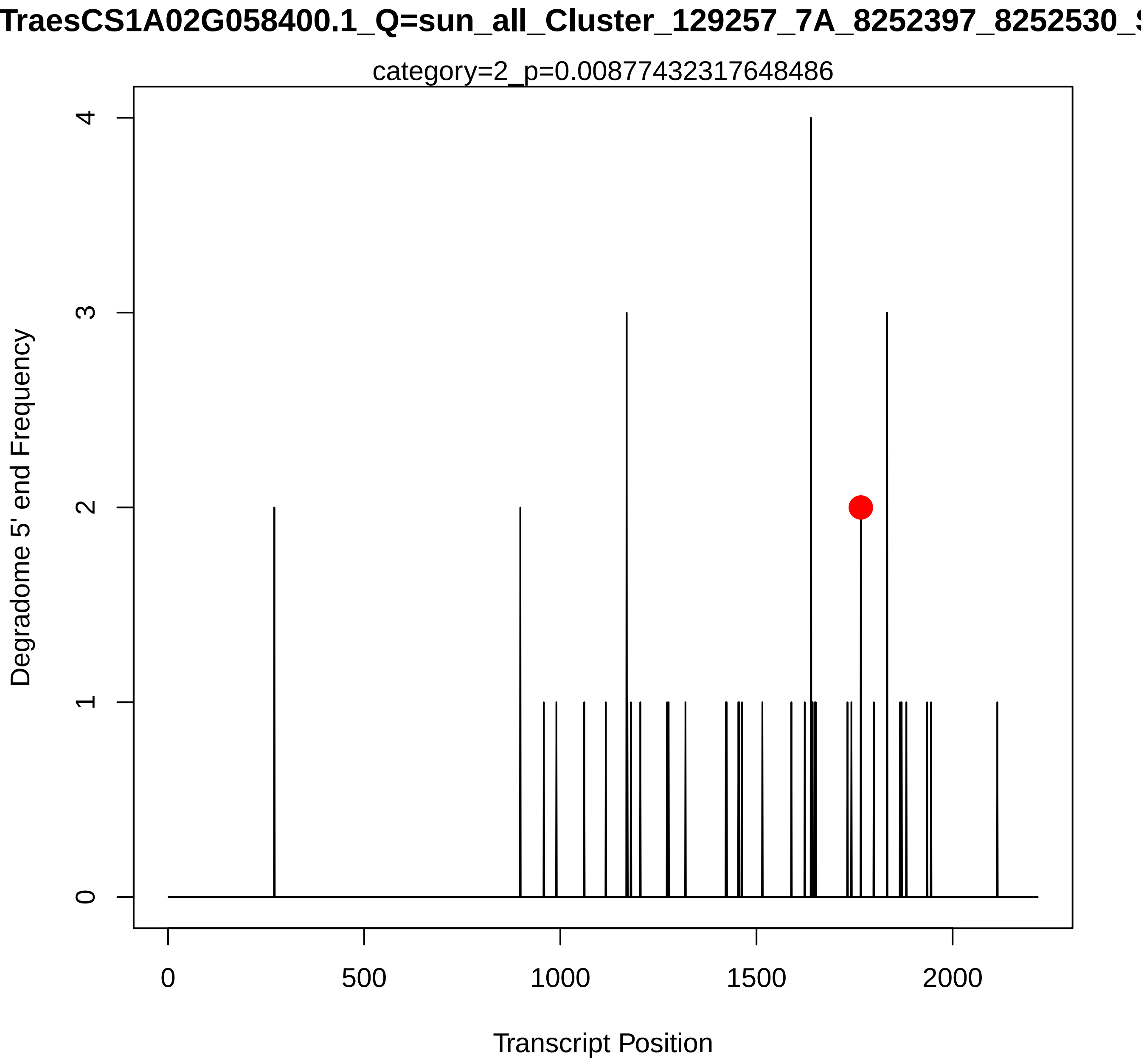

TraesCS1A02G058400.1_Q=sun_all_Cluster_129257_7A_8252397_8252530_S
category=2_p=0.00877432317648486
Degradome 5' end Frequency
Transcript Position
0
1
2
3
4
0
500
1000
1500
2000

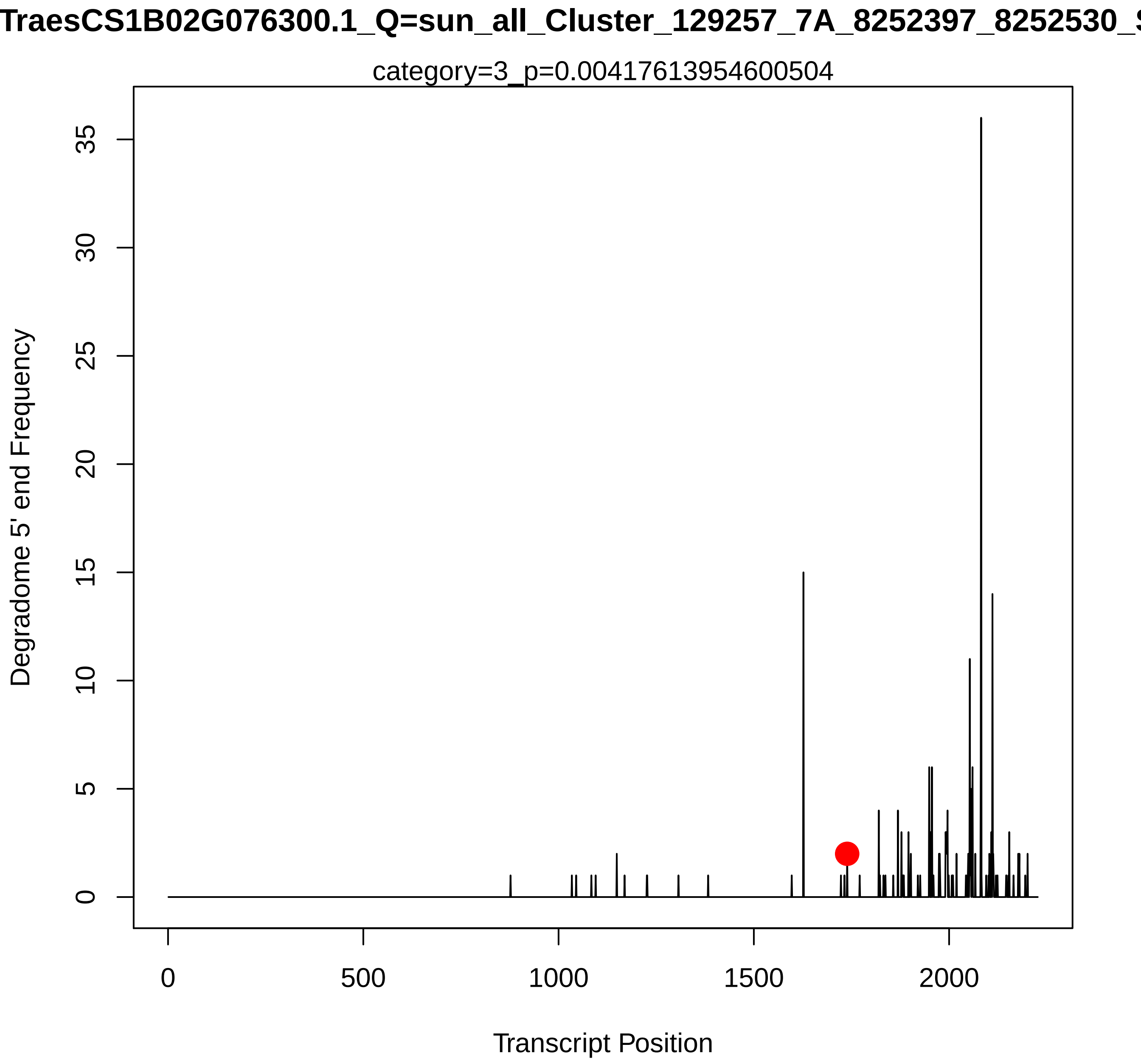
TraesCS1B02G076300.1_Q=sun_all_Cluster_129257_7A_8252397_8252530_S
category=3_p=0.00417613954600504
Degradome 5' end Frequency
Transcript Position
0
5
10
15
20
25
30
35
0
500
1000
1500
2000

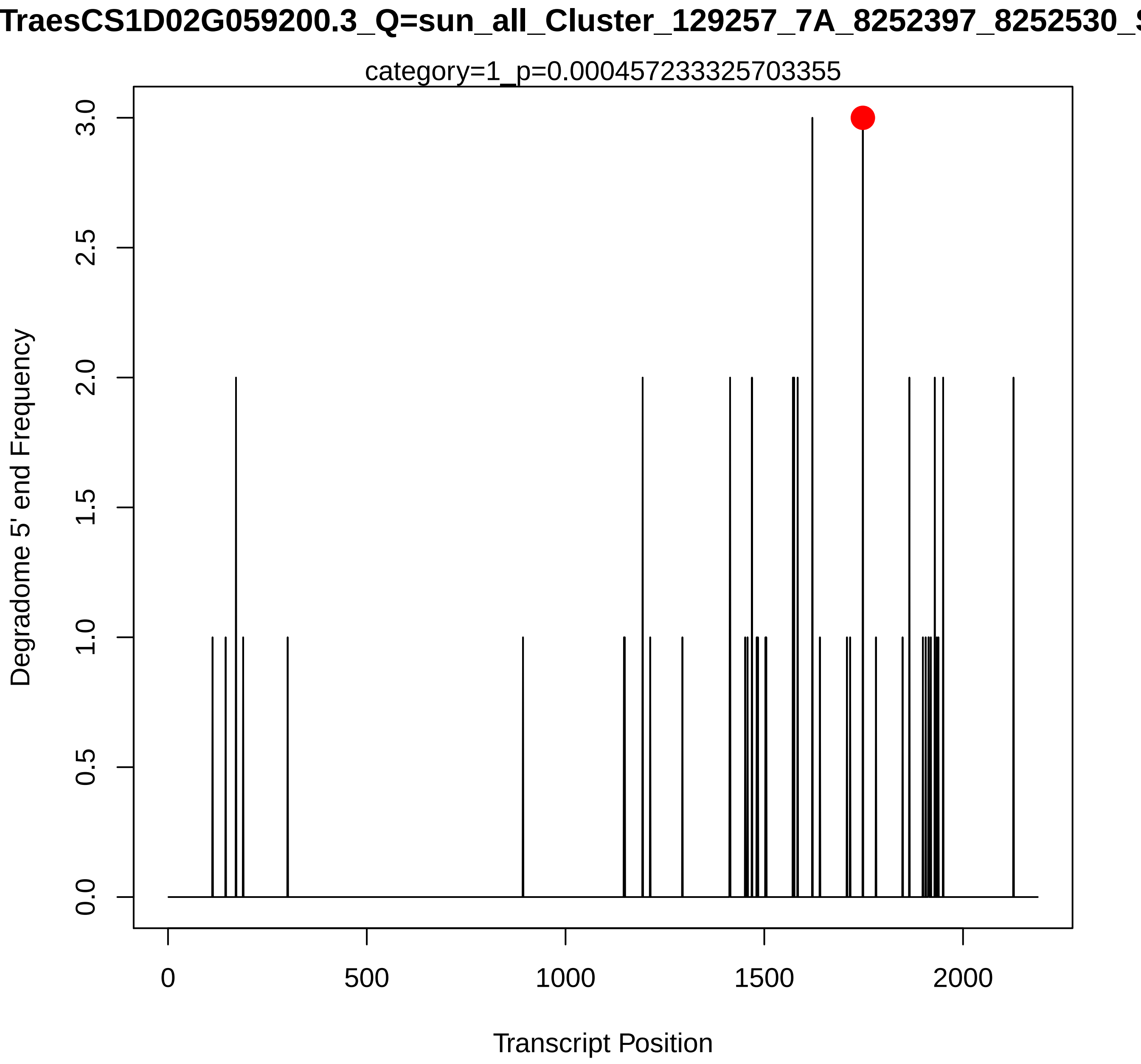
TraesCS1D02G059200.3_Q=sun_all_Cluster_129257_7A_8252397_8252530_S
category=1_p=0.000457233325703355
Degradome 5' end Frequency
Transcript Position
0.0
0.5
1.0
1.5
2.0
2.5
3.0
0
500
1000
1500
2000

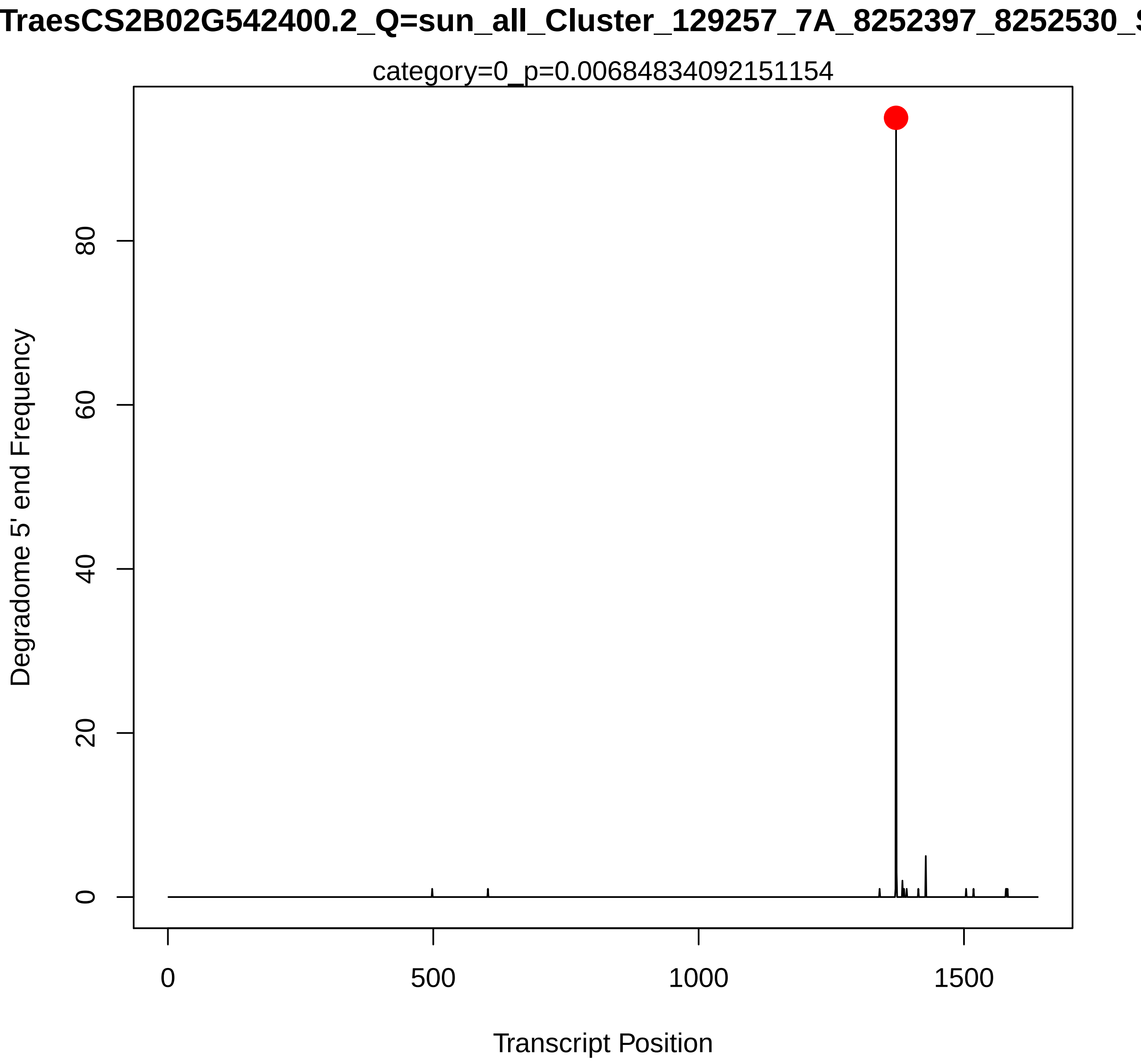
TraesCS2B02G542400.2_Q=sun_all_Cluster_129257_7A_8252397_8252530_S
category=0_p=0.00684834092151154
Degradome 5' end Frequency
0
20
40
60
80
0
500
1000
1500
Transcript Position

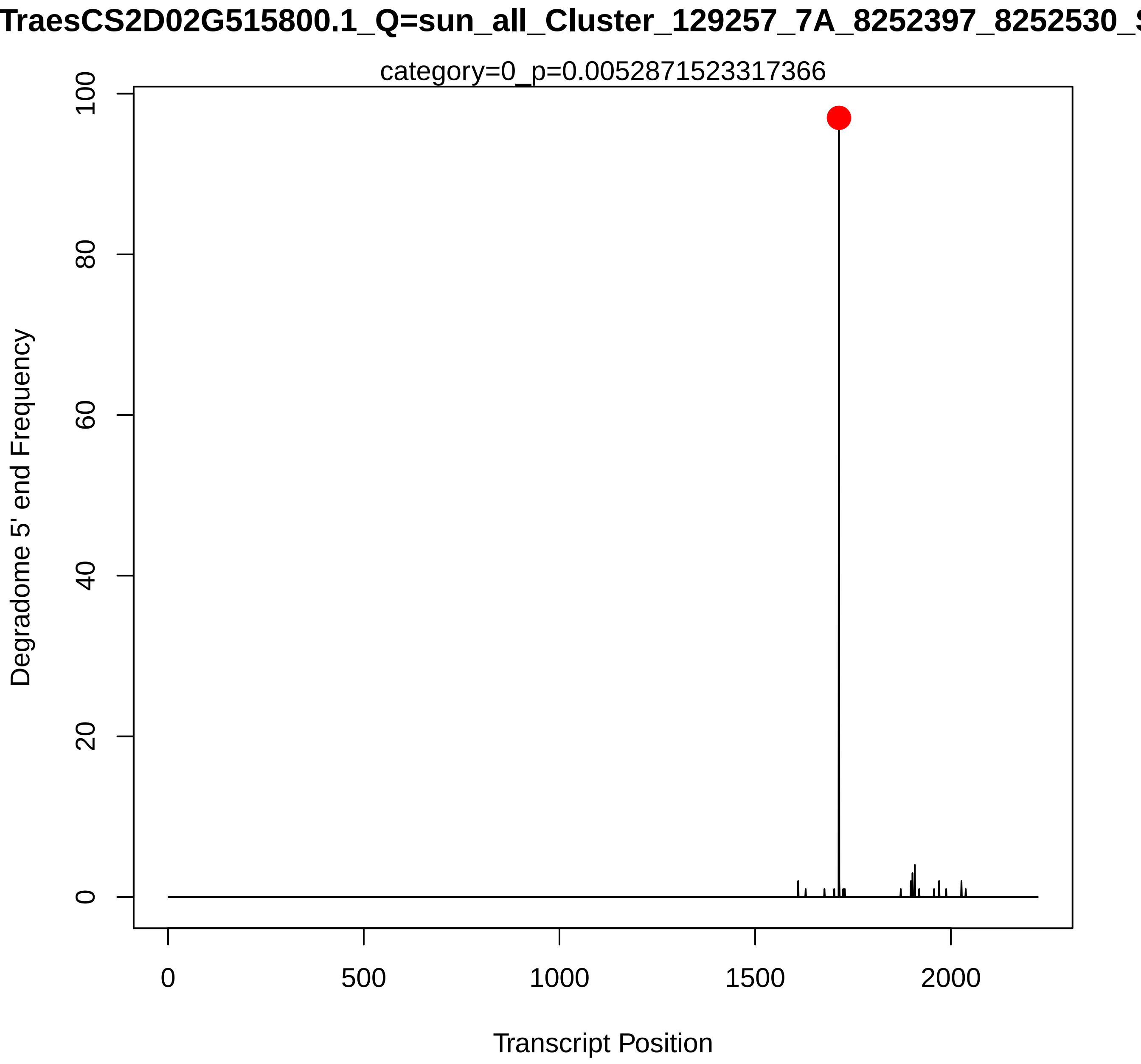

TraesCS2D02G515800.1_Q=sun_all_Cluster_129257_7A_8252397_8252530_S
category=0_p=0.0052871523317366
Degradome 5' end Frequency
Transcript Position
0
20
40
60
80
100
0
500
1000
1500
2000

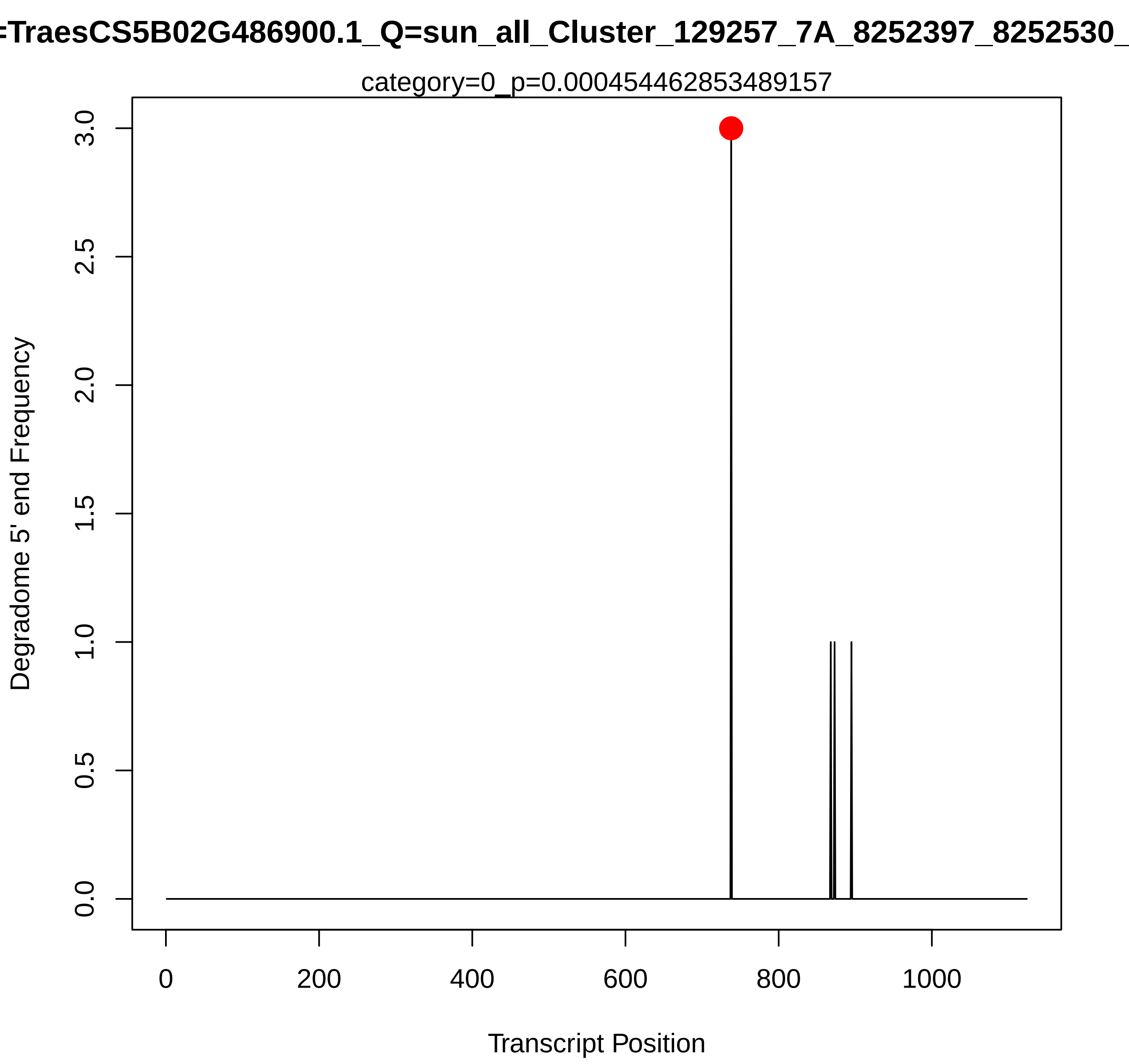
=TraesCS5B02G486900.1_Q=sun_all_Cluster_129257_7A_8252397_8252530_
category=0_p=0.000454462853489157
Degradome 5' end Frequency
0.0
0.5
1.0
1.5
2.0
2.5
3.0
0
200
400
600
800
1000
Transcript Position

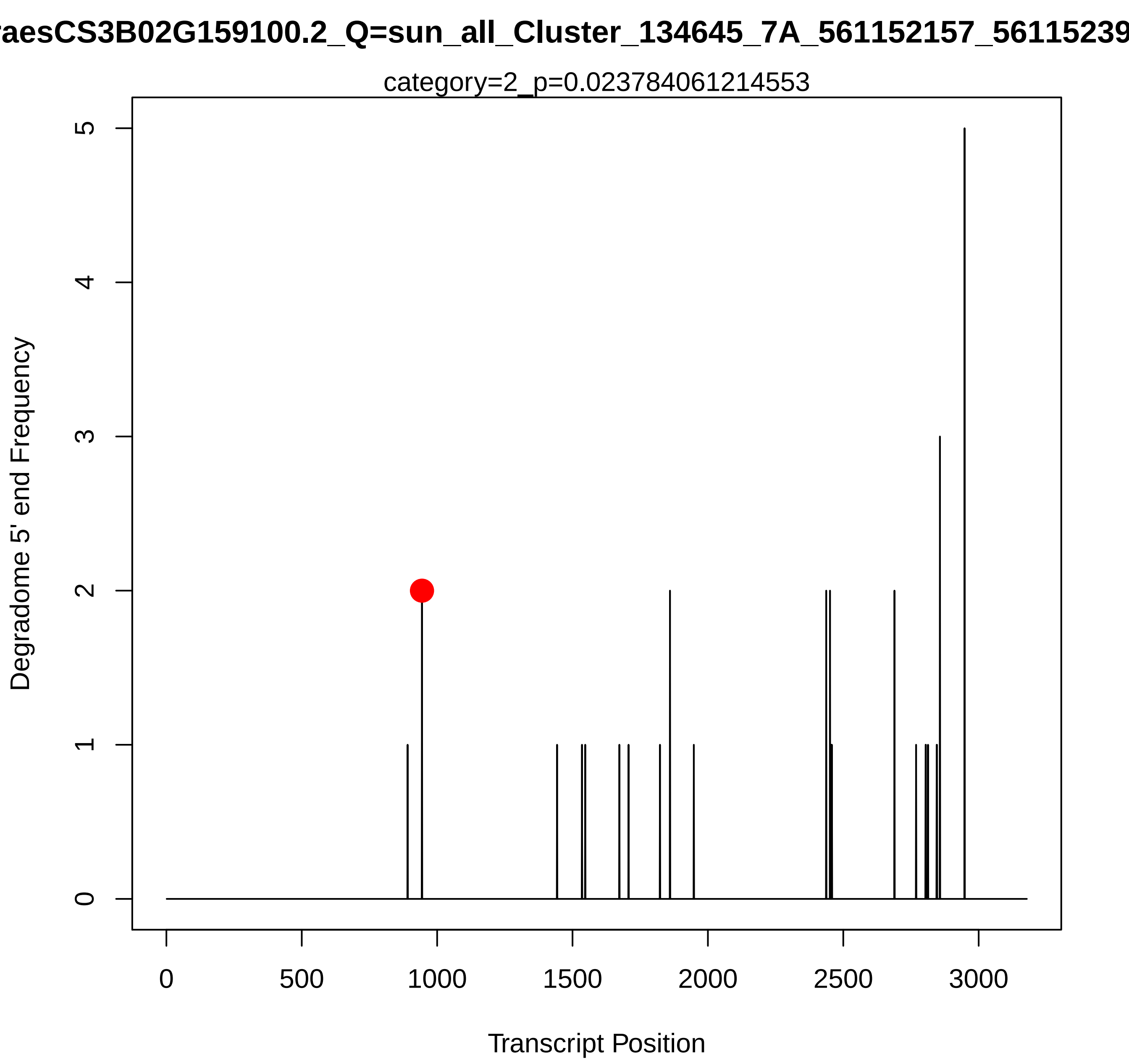

aesCS3B02G159100.2_Q=sun_all_Cluster_134645_7A_561152157_56115239
category=2_p=0.023784061214553
Degradome 5' end Frequency
0
1
2
3
4
5
0
500
1000
1500
2000
2500
3000
Transcript Position

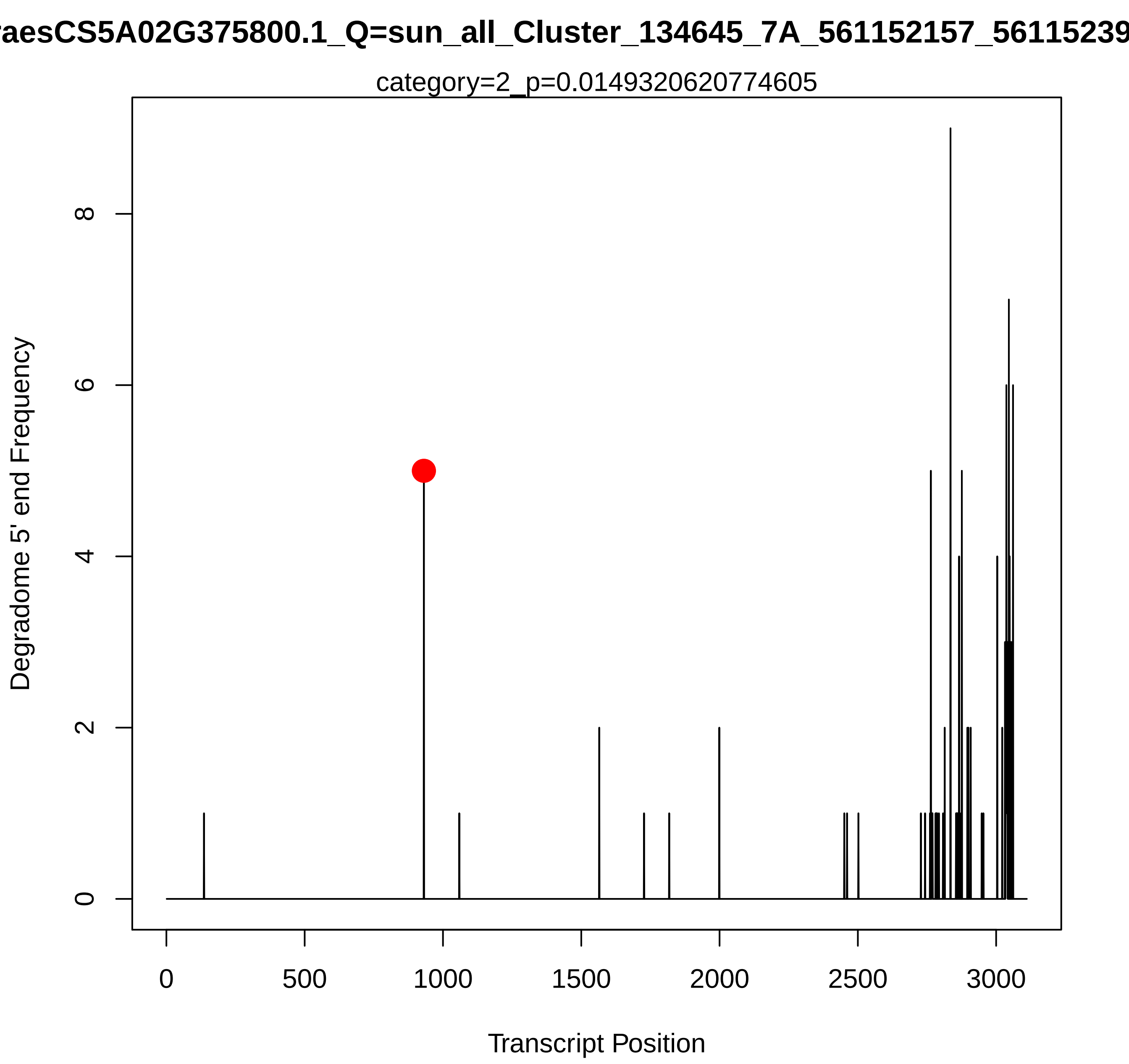

aesCS5A02G375800.1_Q=sun_all_Cluster_134645_7A_561152157_56115239
category=2_p=0.0149320620774605
Degradome 5' end Frequency
0
2
4
6
8
0
500
1000
1500
2000
2500
3000
Transcript Position

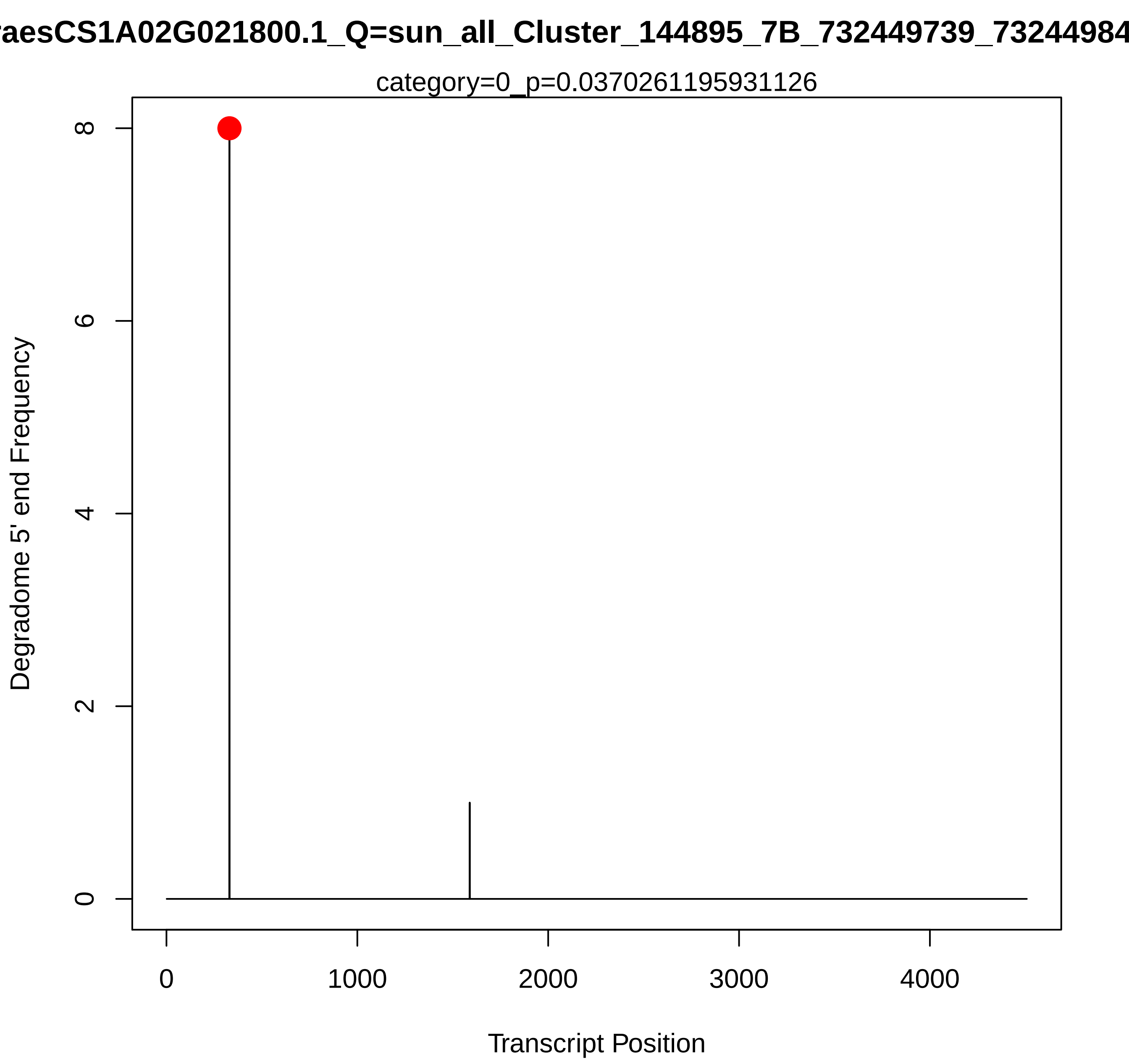
raesCS1A02G021800.1_Q=sun_all_Cluster_144895_7B_732449739_73244984
category=0_p=0.0370261195931126
Degradome 5' end Frequency
0
2
4
6
8
0
1000
2000
3000
4000
Transcript Position

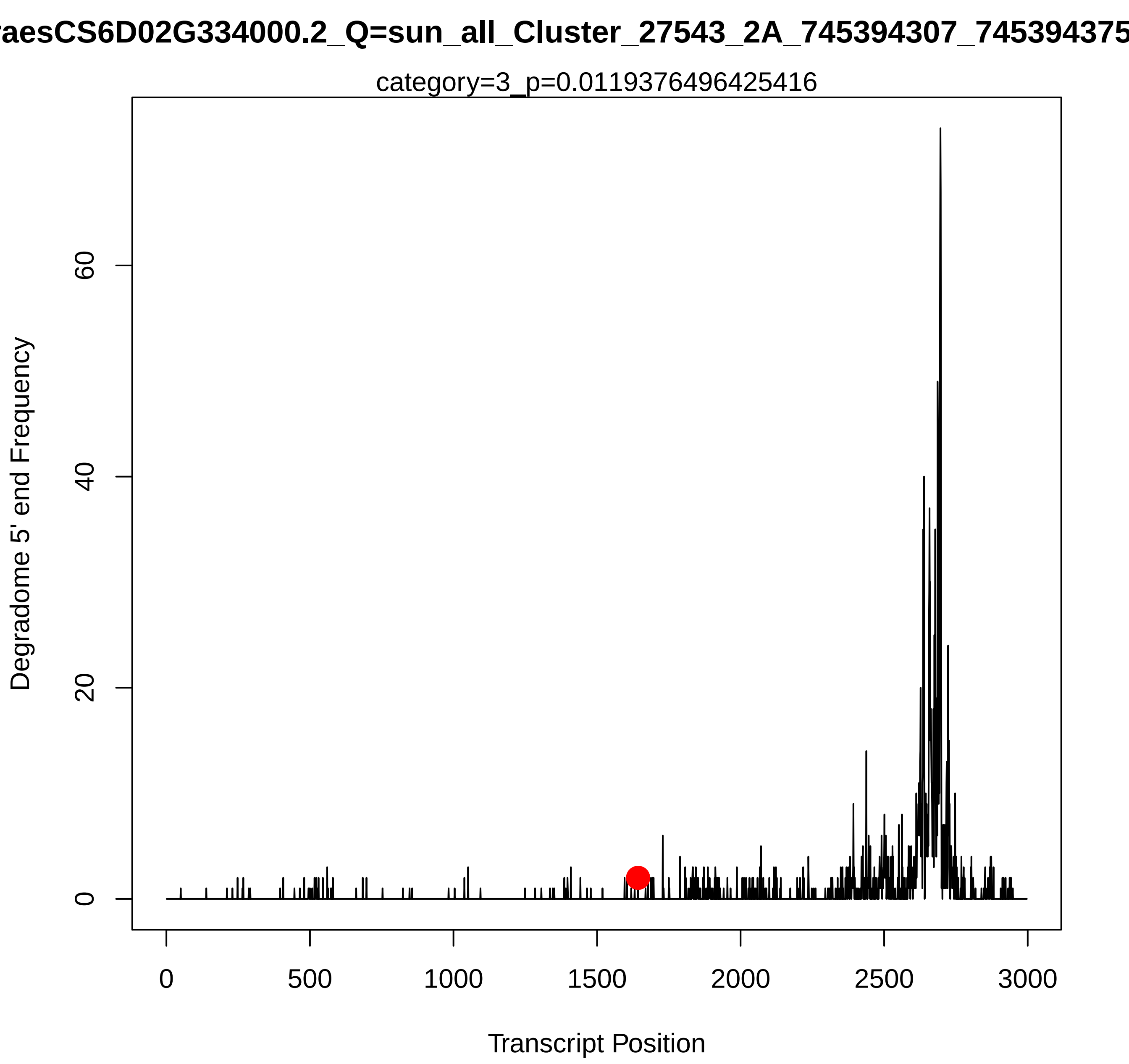

raesCS6D02G334000.2_Q=sun_all_Cluster_27543_2A_745394307_745394375
category=3_p=0.0119376496425416
Degradome 5' end Frequency
0
20
40
60
0
500
1000
1500
2000
2500
3000
Transcript Position

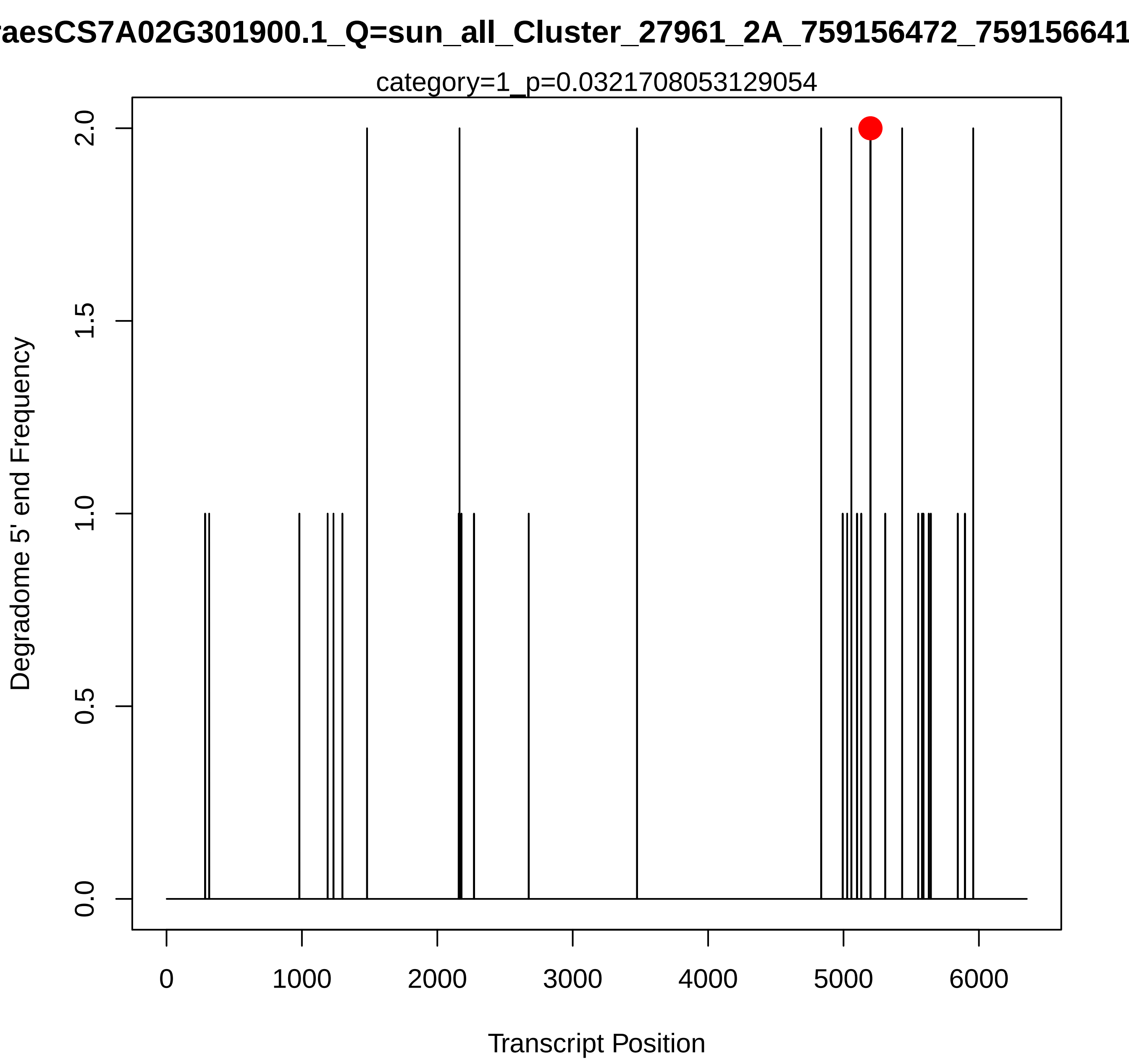

aesCS7A02G301900.1_Q=sun_all_Cluster_27961_2A_759156472_759156641
category=1_p=0.0321708053129054
Degradome 5' end Frequency
0.0
0.5
1.0
1.5
2.0
0
1000
2000
3000
4000
5000
6000
Transcript Position

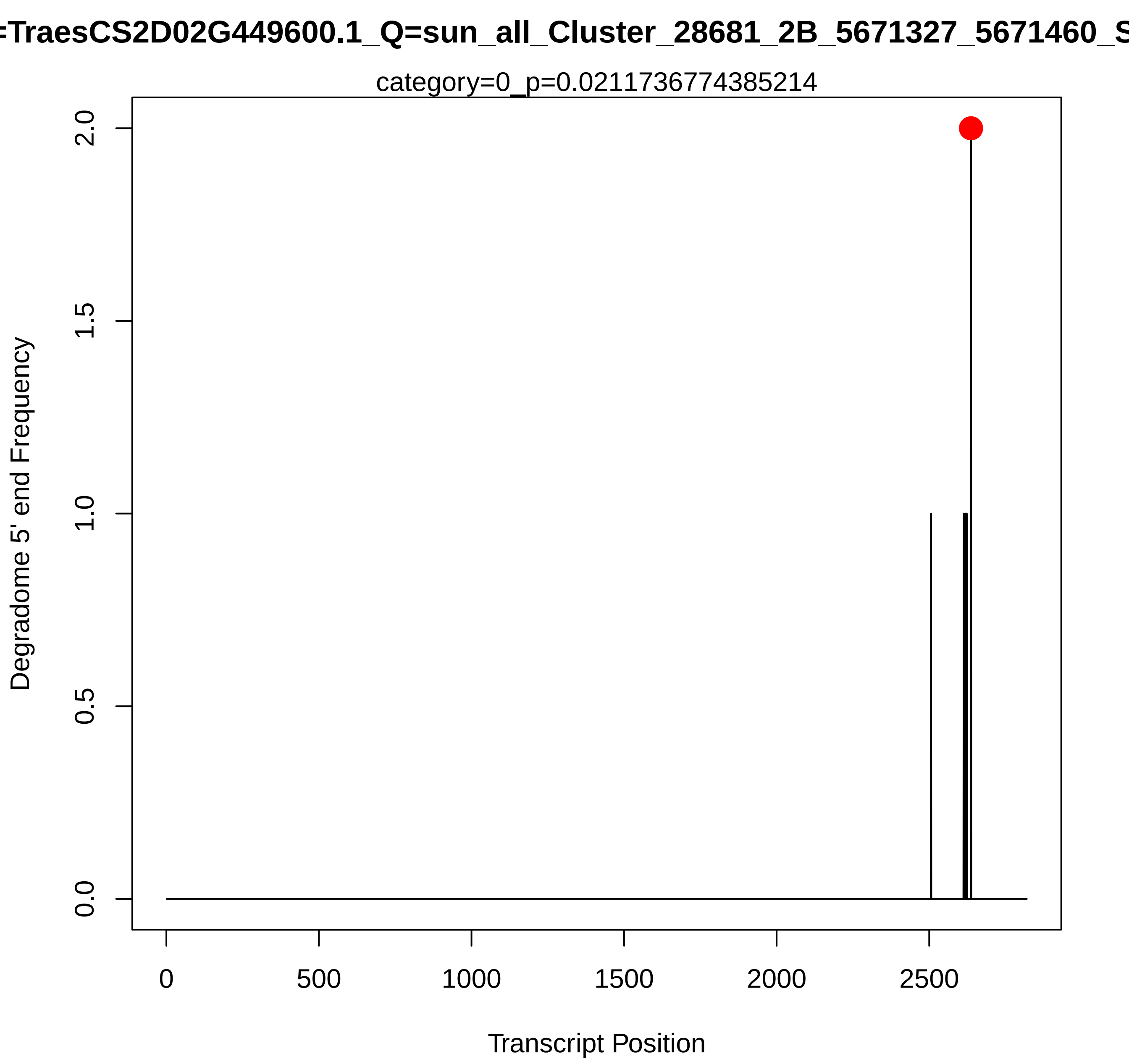
=TraesCS2D02G449600.1_Q=sun_all_Cluster_28681_2B_5671327_5671460_S
category=0_p=0.0211736774385214
Degradome 5' end Frequency
0.0
0.5
1.0
1.5
2.0
0
500
1000
1500
2000
2500
Transcript Position

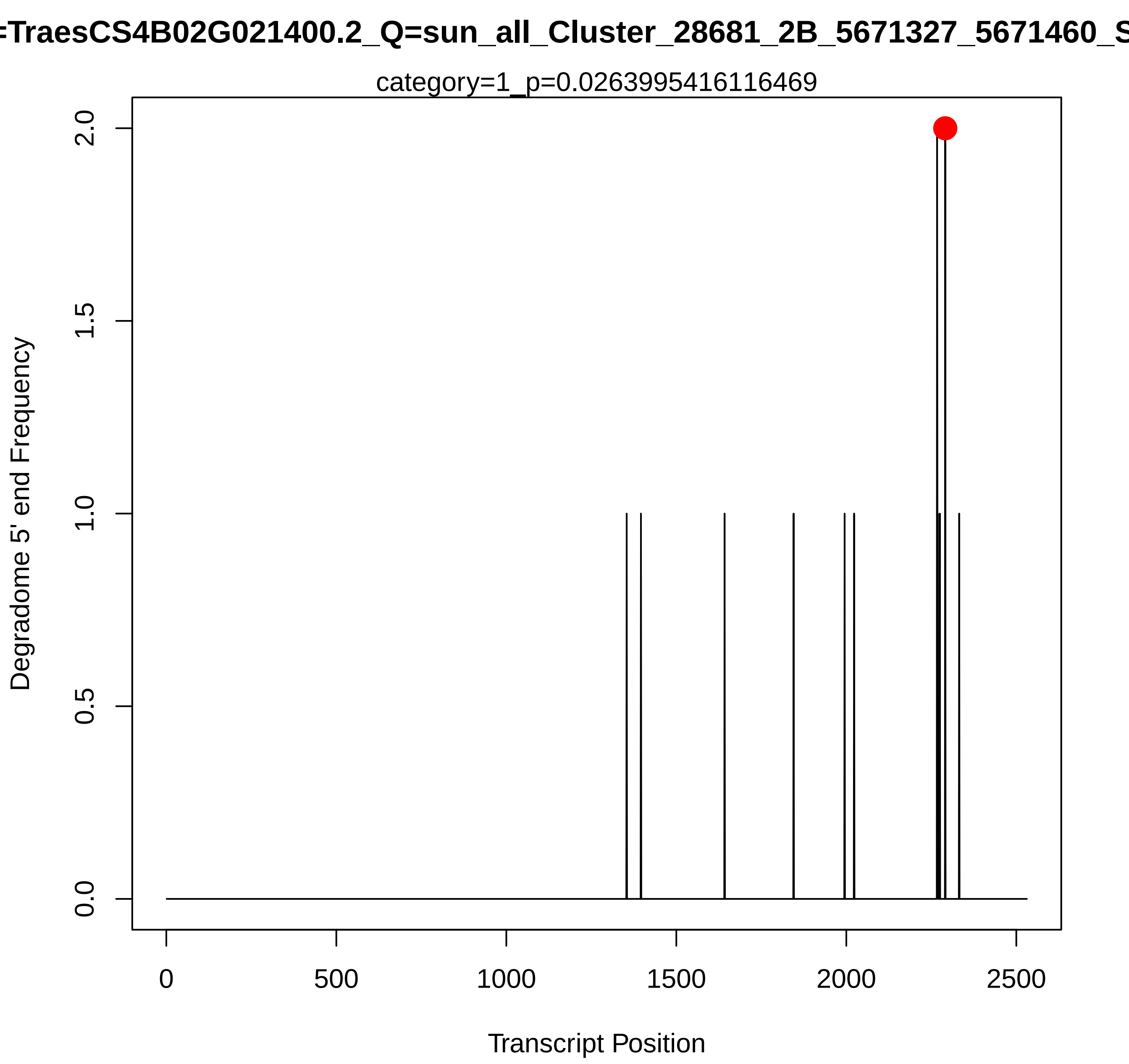

=TraesCS4B02G021400.2_Q=sun_all_Cluster_28681_2B_5671327_5671460_S
category=1_p=0.0263995416116469
Degradome 5' end Frequency
0.0
0.5
1.0
1.5
2.0
0
500
1000
1500
2000
2500
Transcript Position

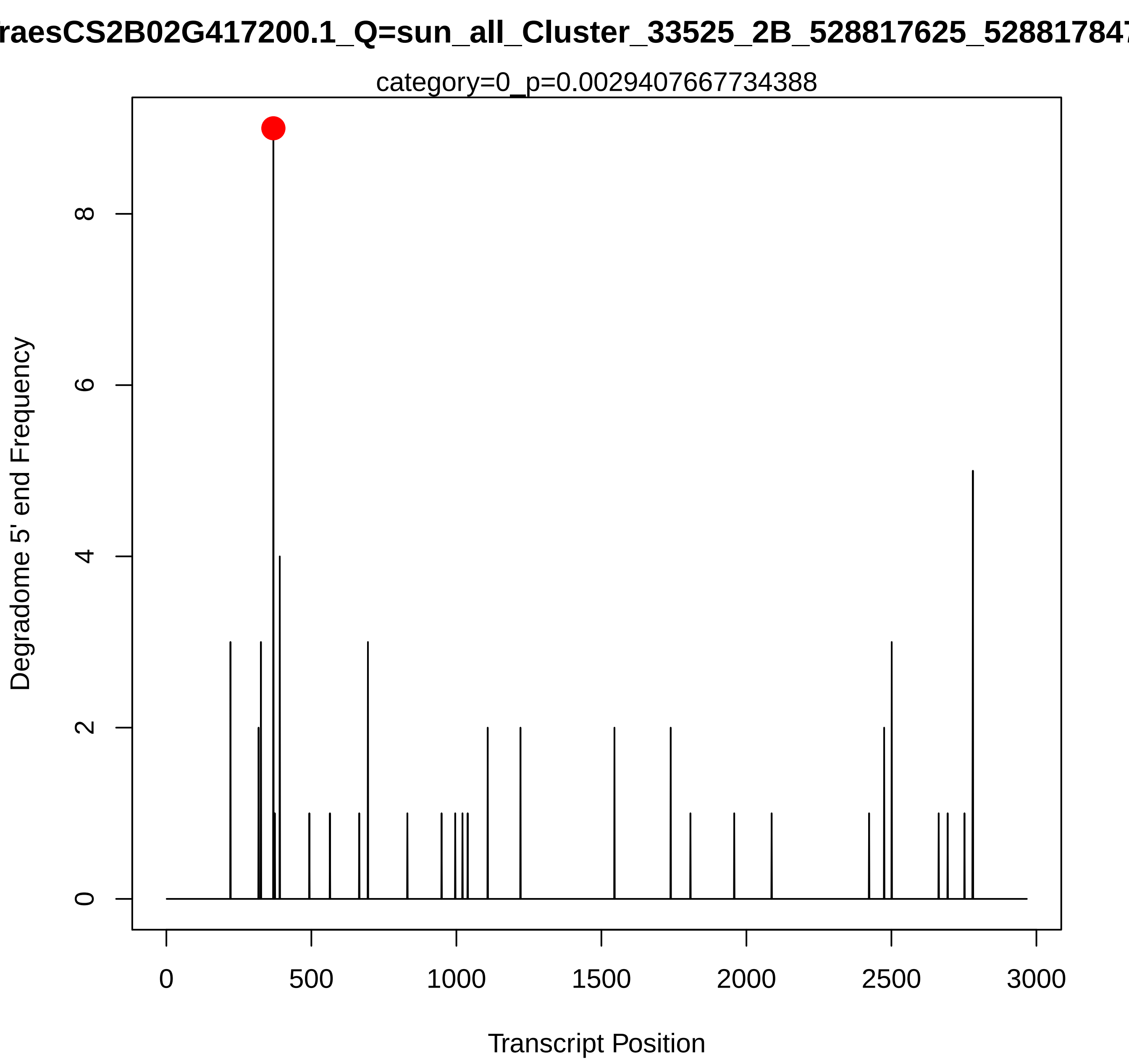

TraesCS2B02G417200.1_Q=sun_all_Cluster_33525_2B_528817625_52881784
category=0_p=0.0029407667734388
Degradome 5' end Frequency
0
2
4
6
8
0
500
1000
1500
2000
2500
3000
Transcript Position

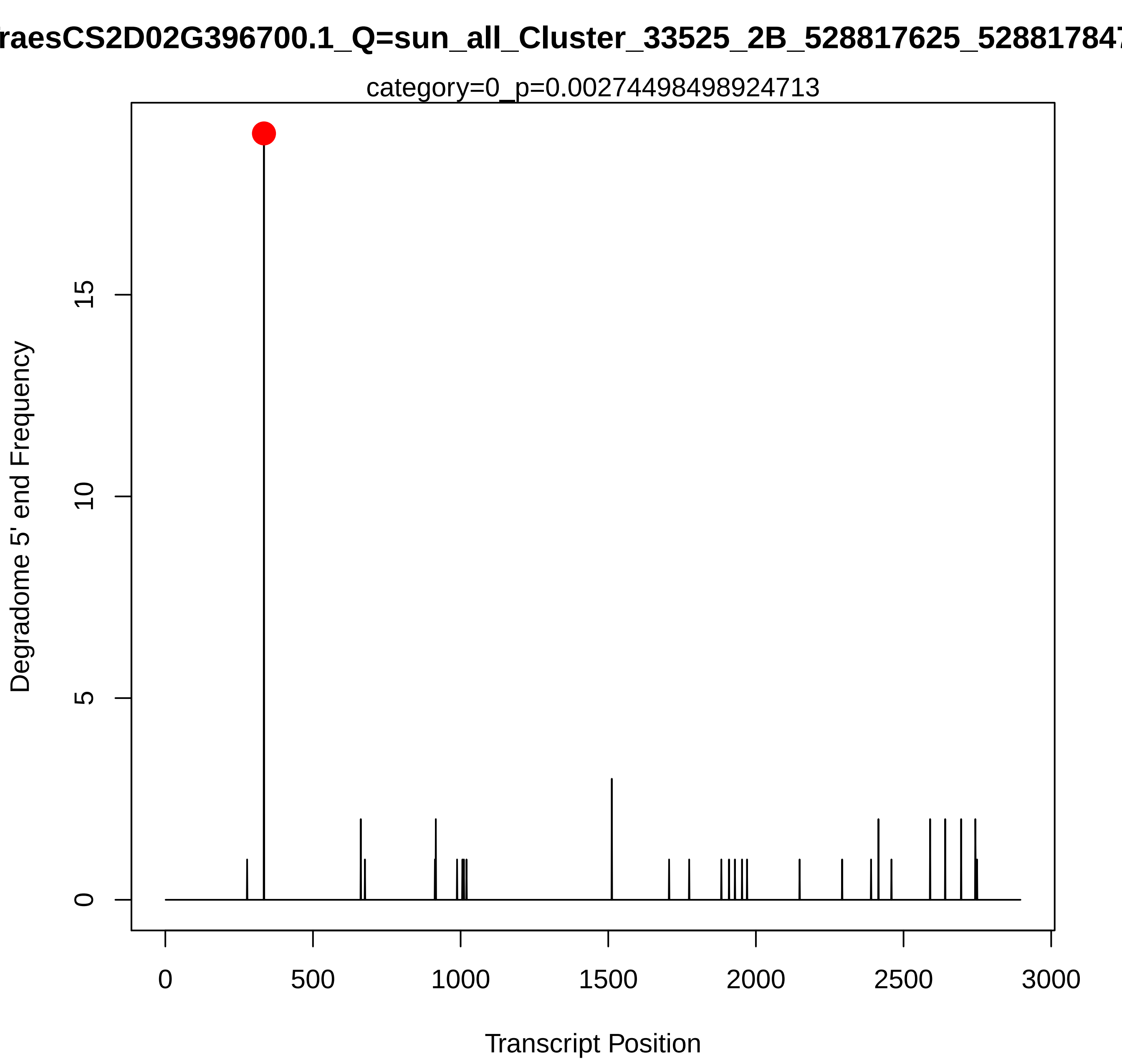

raesCS2D02G396700.1_Q=sun_all_Cluster_33525_2B_528817625_52881784
category=0_p=0.00274498498924713
Degradome 5' end Frequency
0
5
10
15
0
500
1000
1500
2000
2500
3000
Transcript Position

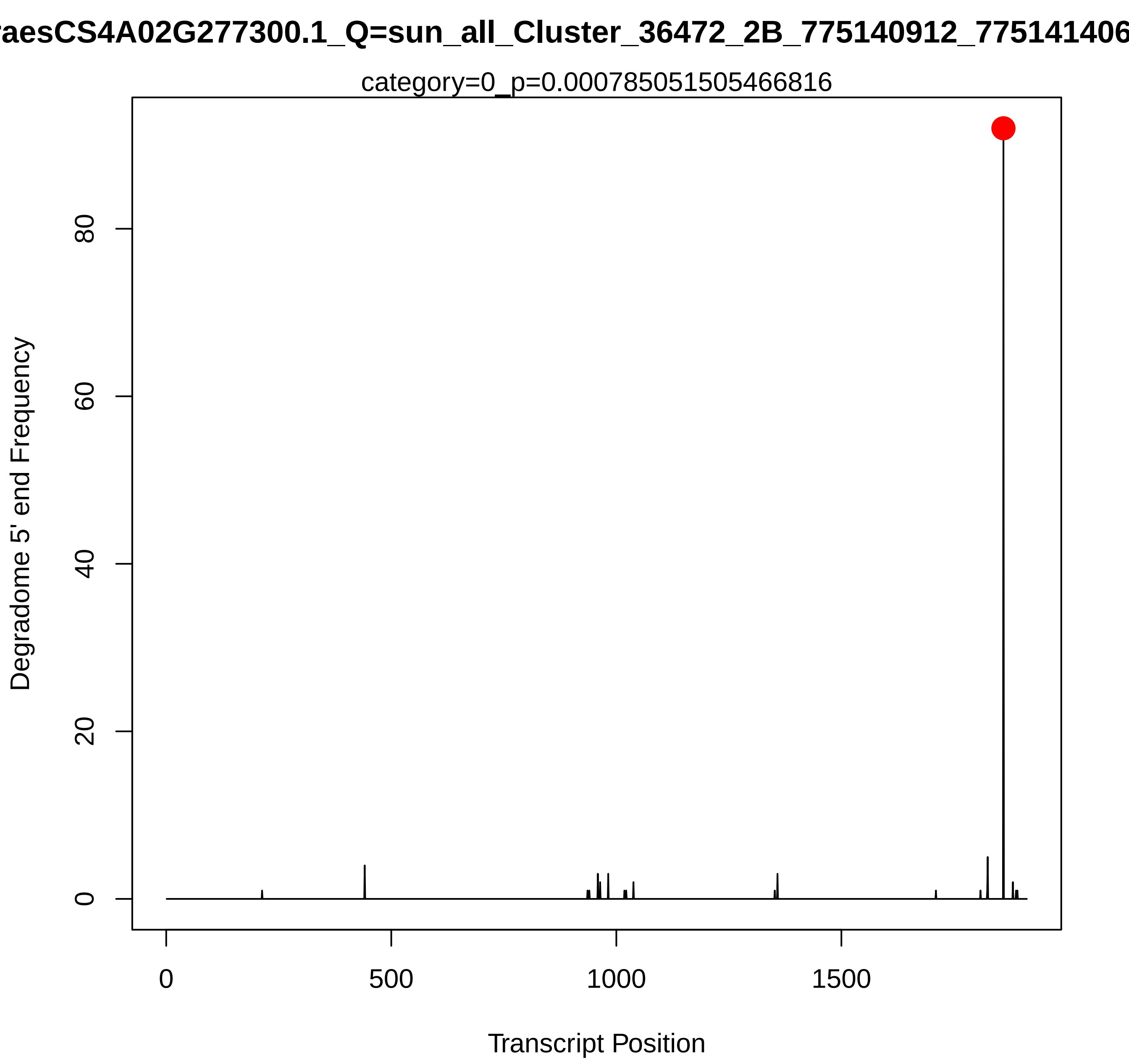

raesCS4A02G277300.1_Q=sun_all_Cluster_36472_2B_775140912_775141406
category=0_p=0.000785051505466816
Degradome 5' end Frequency
0
20
40
60
80
0
500
1000
1500
Transcript Position

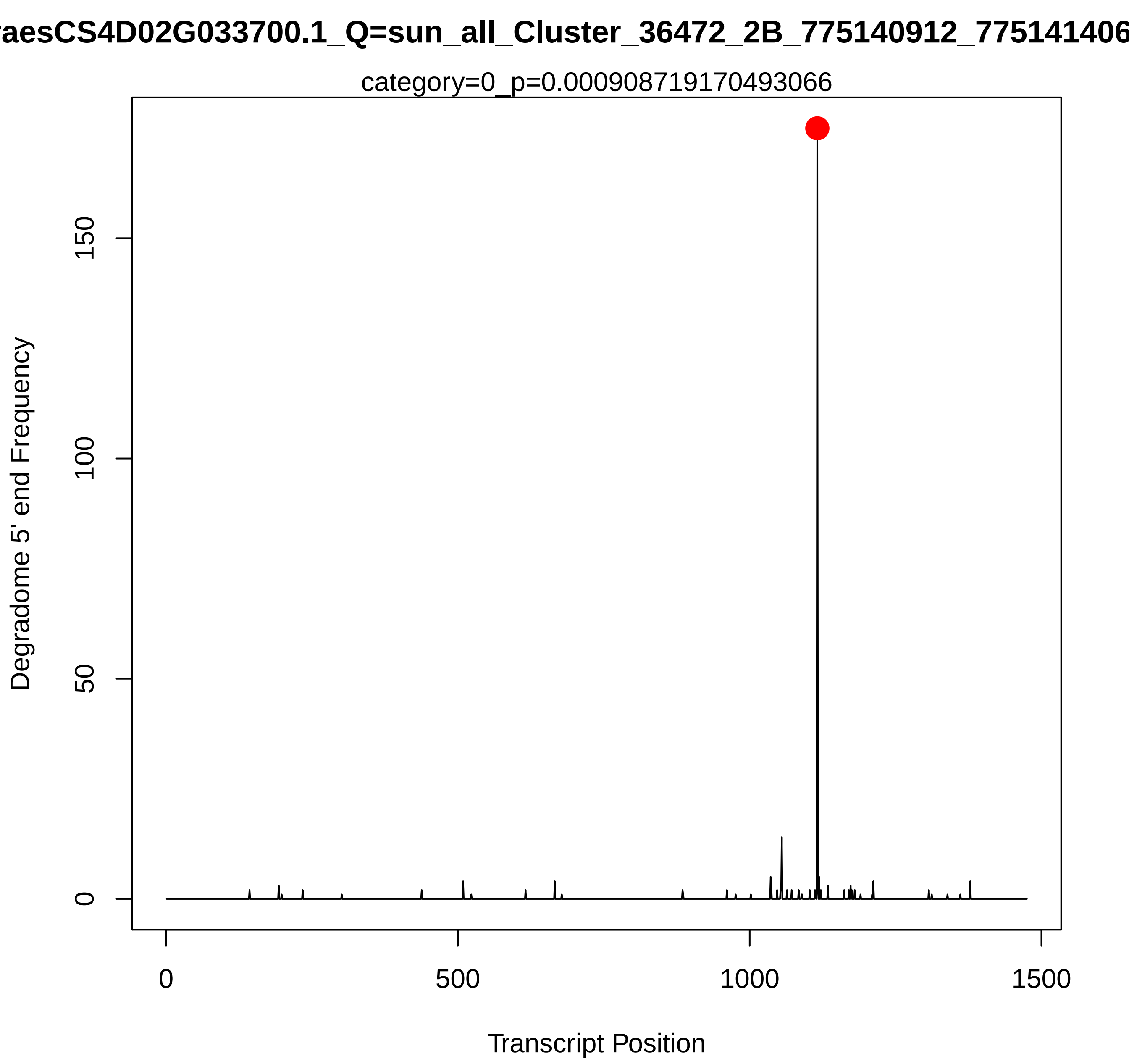
raesCS4D02G033700.1_Q=sun_all_Cluster_36472_2B_775140912_775141406
category=0_p=0.000908719170493066
Degradome 5' end Frequency
0
50
100
150
0
500
1000
1500
Transcript Position

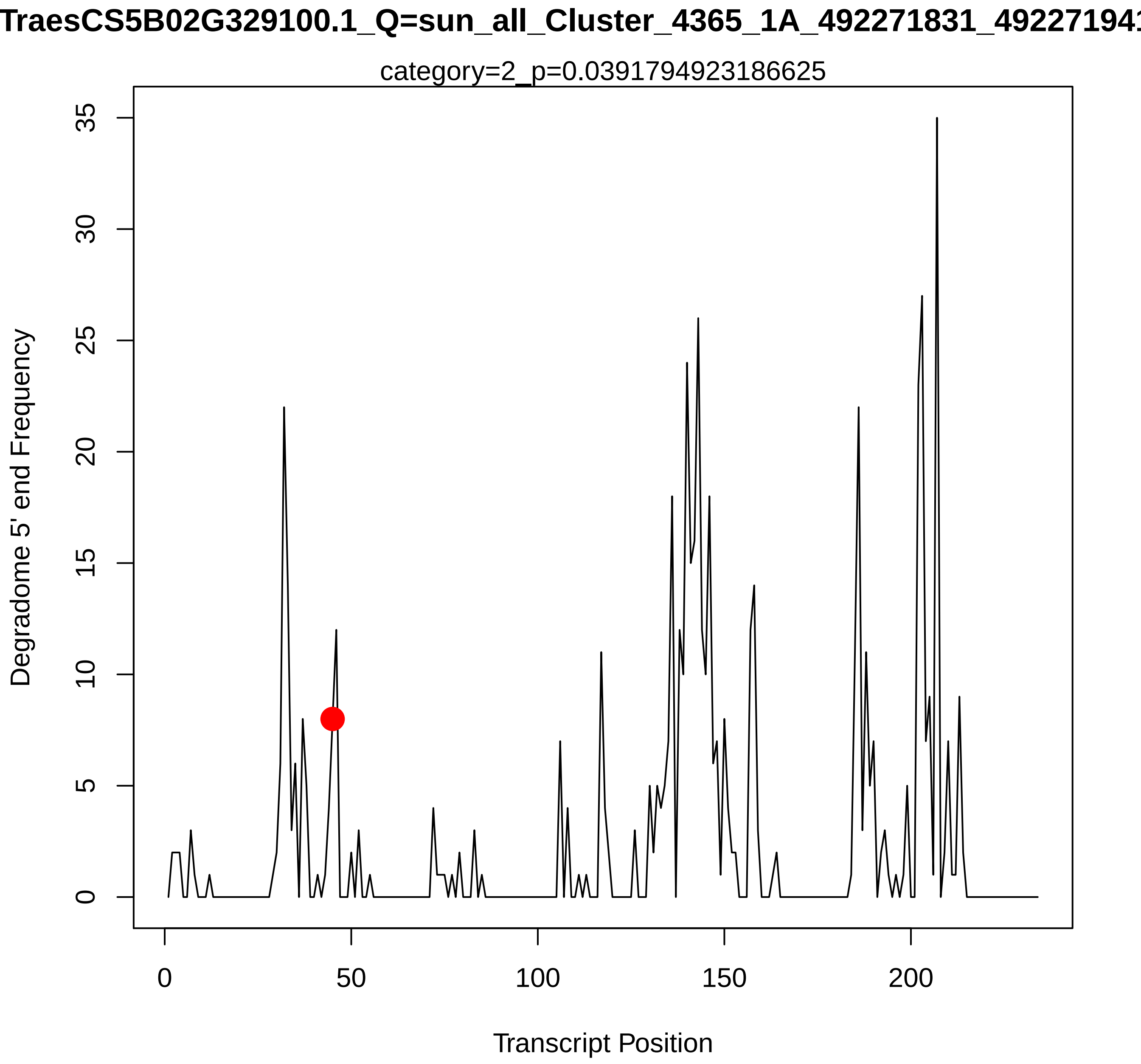
TraesCS5B02G329100.1_Q=sun_all_Cluster_4365_1A_492271831_49227194
category=2_p=0.0391794923186625
Degradome 5' end Frequency
Transcript Position
0
5
10
15
20
25
30
35
0
50
100
150
200

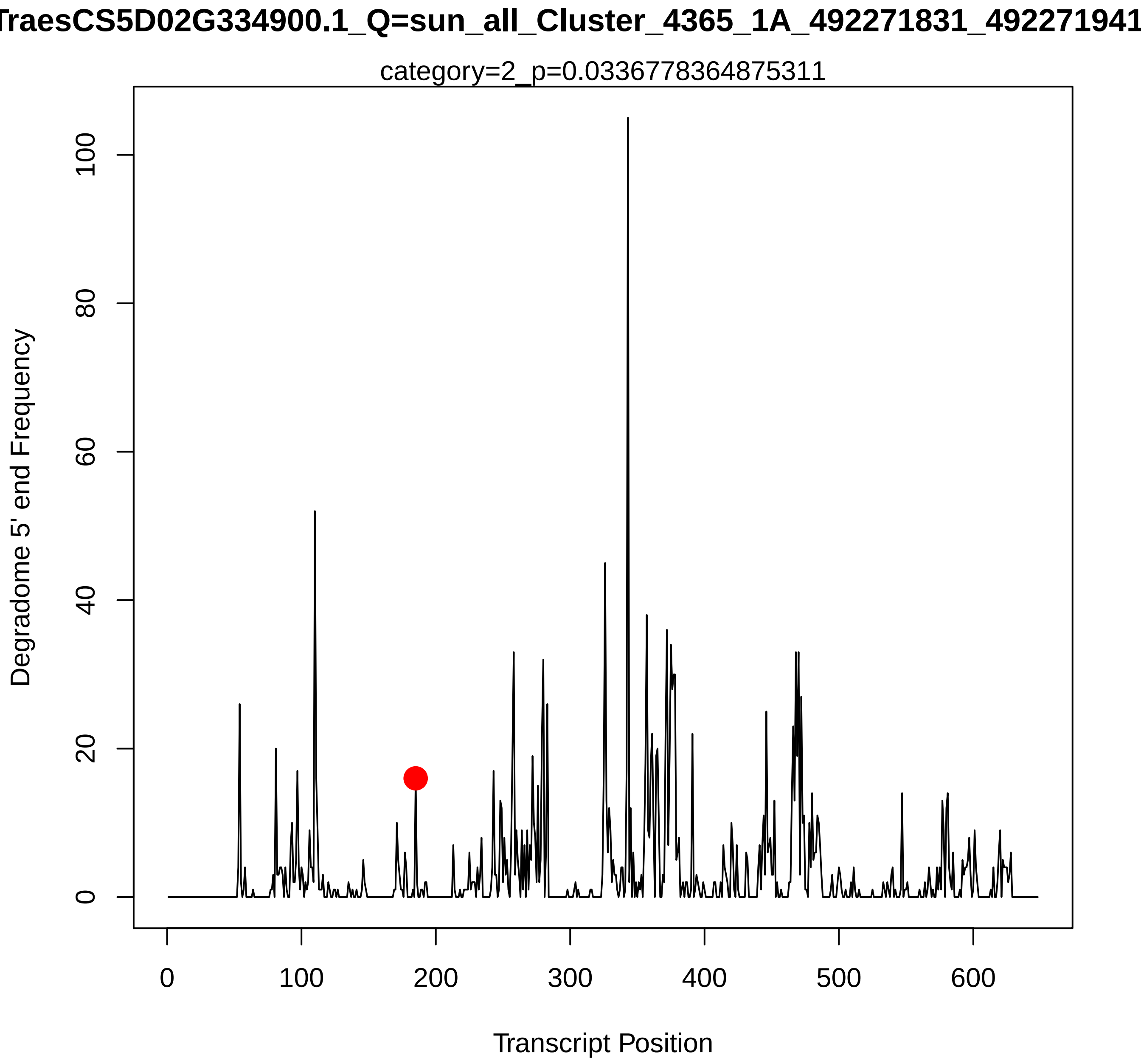
TraesCS5D02G334900.1_Q=sun_all_Cluster_4365_1A_492271831_492271941
category=2_p=0.0336778364875311
Degradome 5' end Frequency
Transcript Position
0
20
40
60
80
100
0
100
200
300
400
500
600

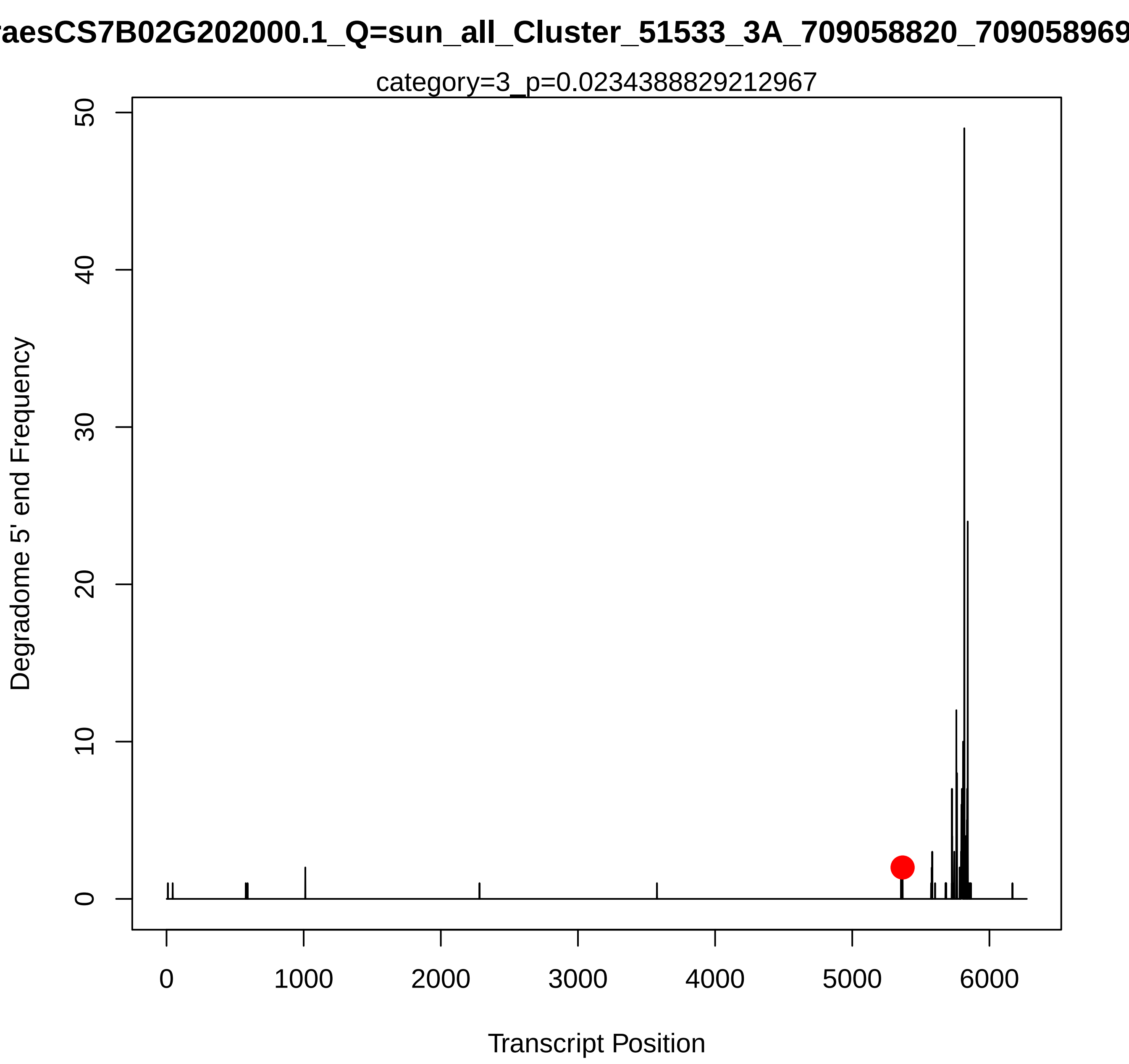

aesCS7B02G202000.1_Q=sun_all_Cluster_51533_3A_709058820_709058969
category=3_p=0.0234388829212967
Degradome 5' end Frequency
0
10
20
30
40
50
0
1000
2000
3000
4000
5000
6000
Transcript Position

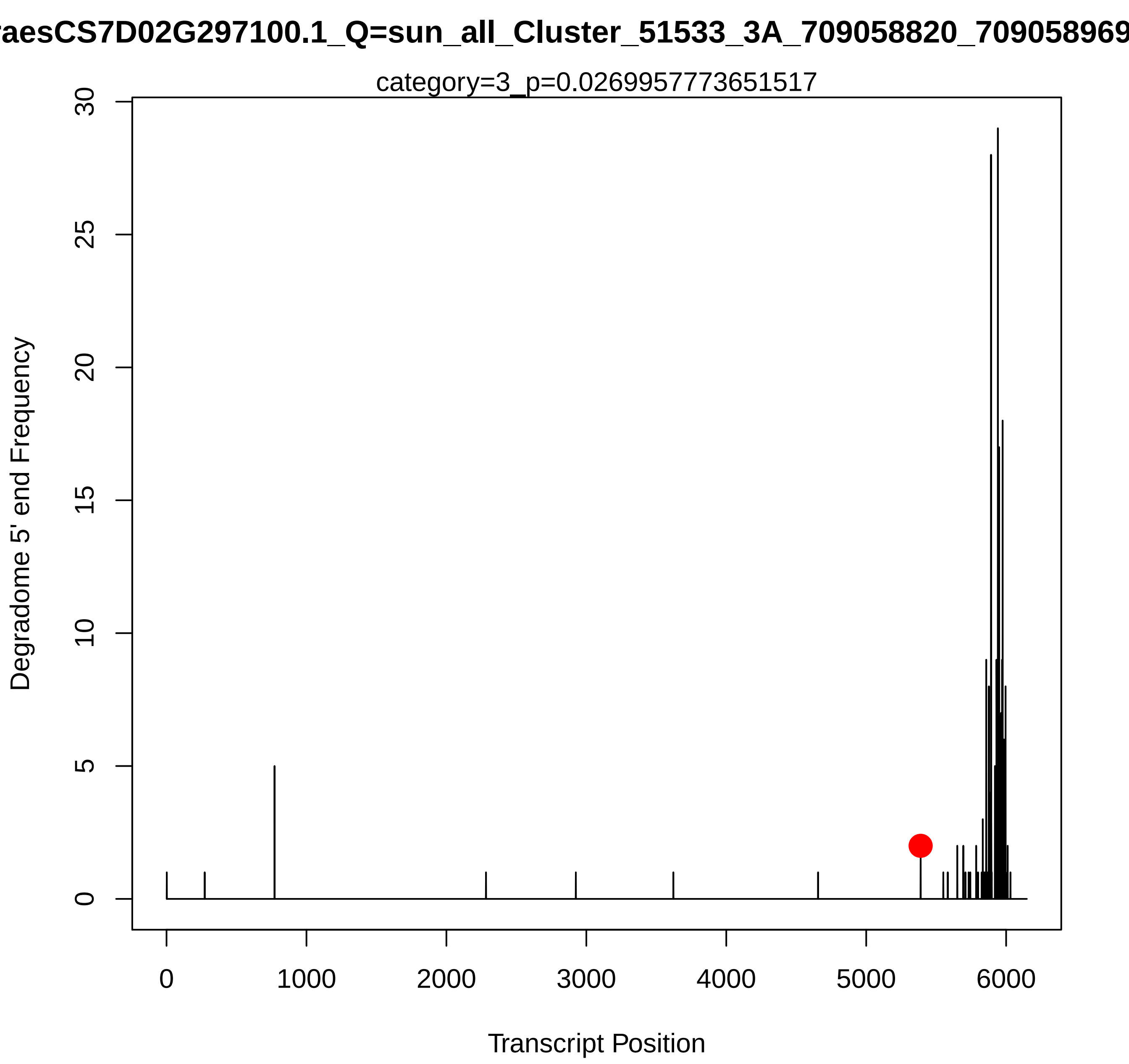

raesCS7D02G297100.1_Q=sun_all_Cluster_51533_3A_709058820_709058969
category=3_p=0.0269957773651517
Degradome 5' end Frequency
0
5
10
15
20
25
30
0
1000
2000
3000
4000
5000
6000
Transcript Position

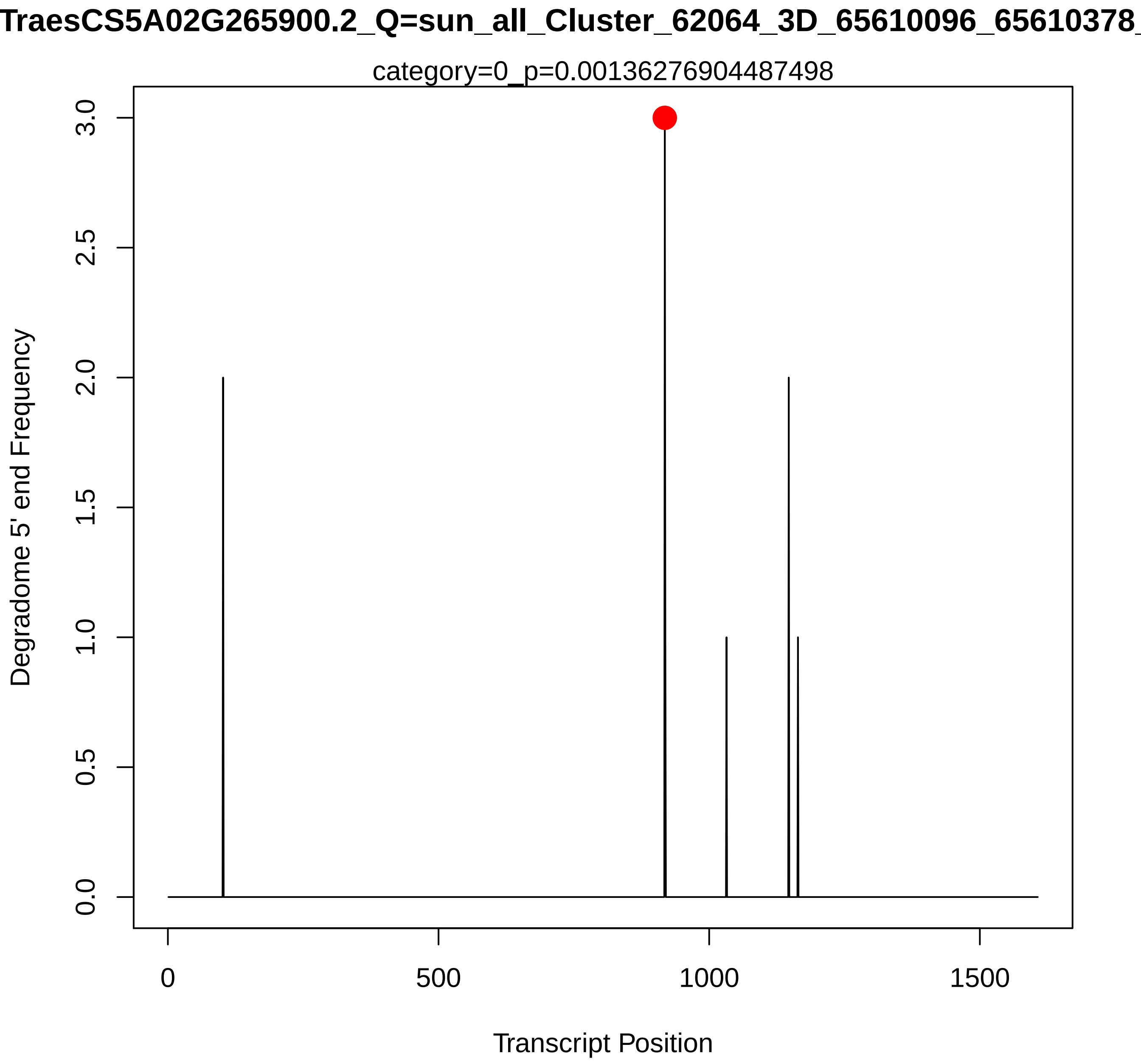
TraesCS5A02G265900.2_Q=sun_all_Cluster_62064_3D_65610096_65610378_
category=0_p=0.00136276904487498
Degradome 5' end Frequency
0.0
0.5
1.0
1.5
2.0
2.5
3.0
0
500
1000
1500
Transcript Position

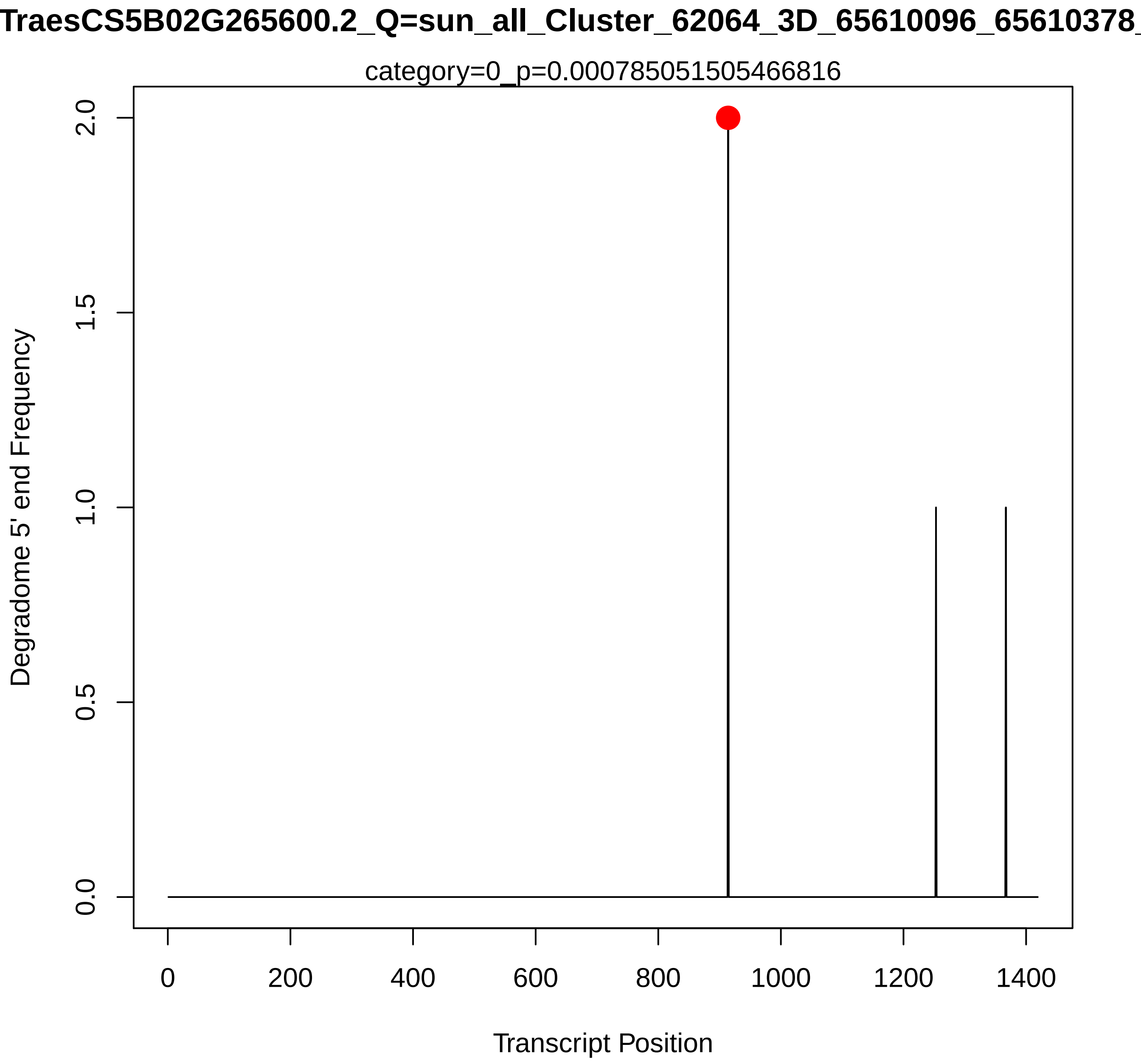
TraesCS5B02G265600.2_Q=sun_all_Cluster_62064_3D_65610096_65610378_
category=0_p=0.000785051505466816
Degradome 5' end Frequency
0.0
0.5
1.0
1.5
2.0
0
200
400
600
800
1000
1200
1400
Transcript Position

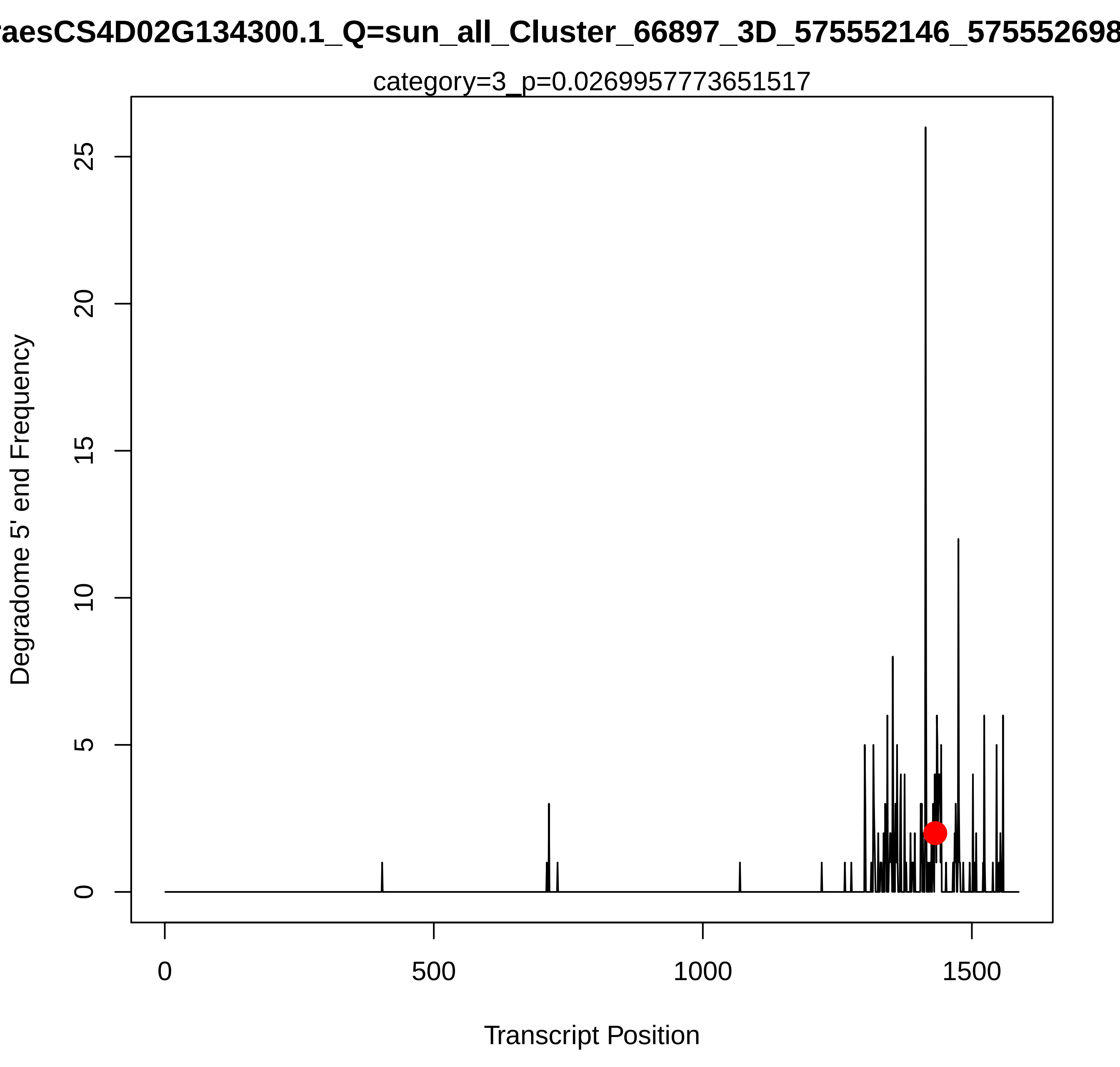

raesCS4D02G134300.1_Q=sun_all_Cluster_66897_3D_575552146_575552698
category=3_p=0.0269957773651517
Degradome 5' end Frequency
Transcript Position
0
5
10
15
20
25
0
500
1000
1500

aesCS5A02G029900.1_Q=sun_all_Cluster_70525_4A_494638262_494638437

category=0_p=0.0015897165954033

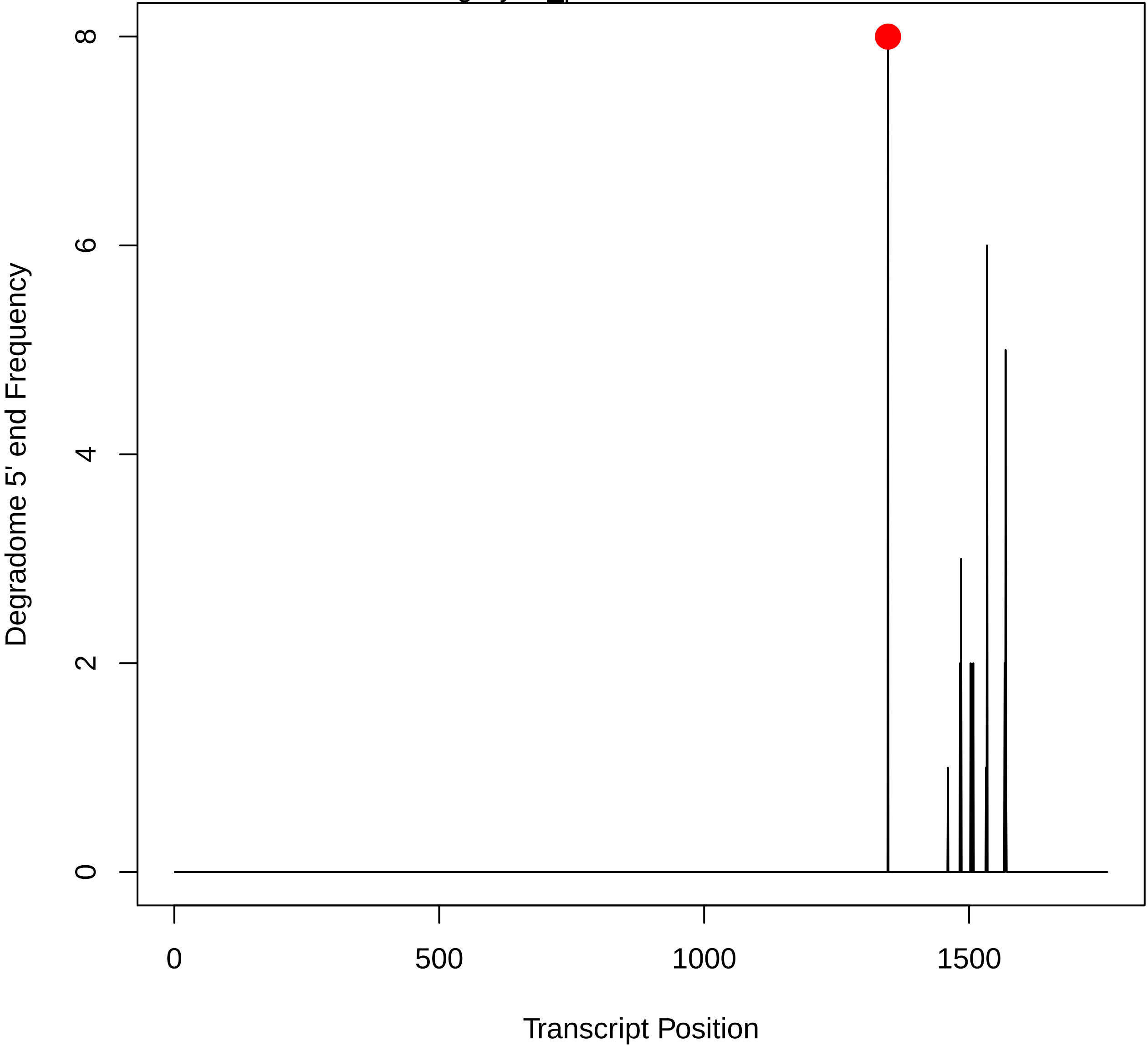

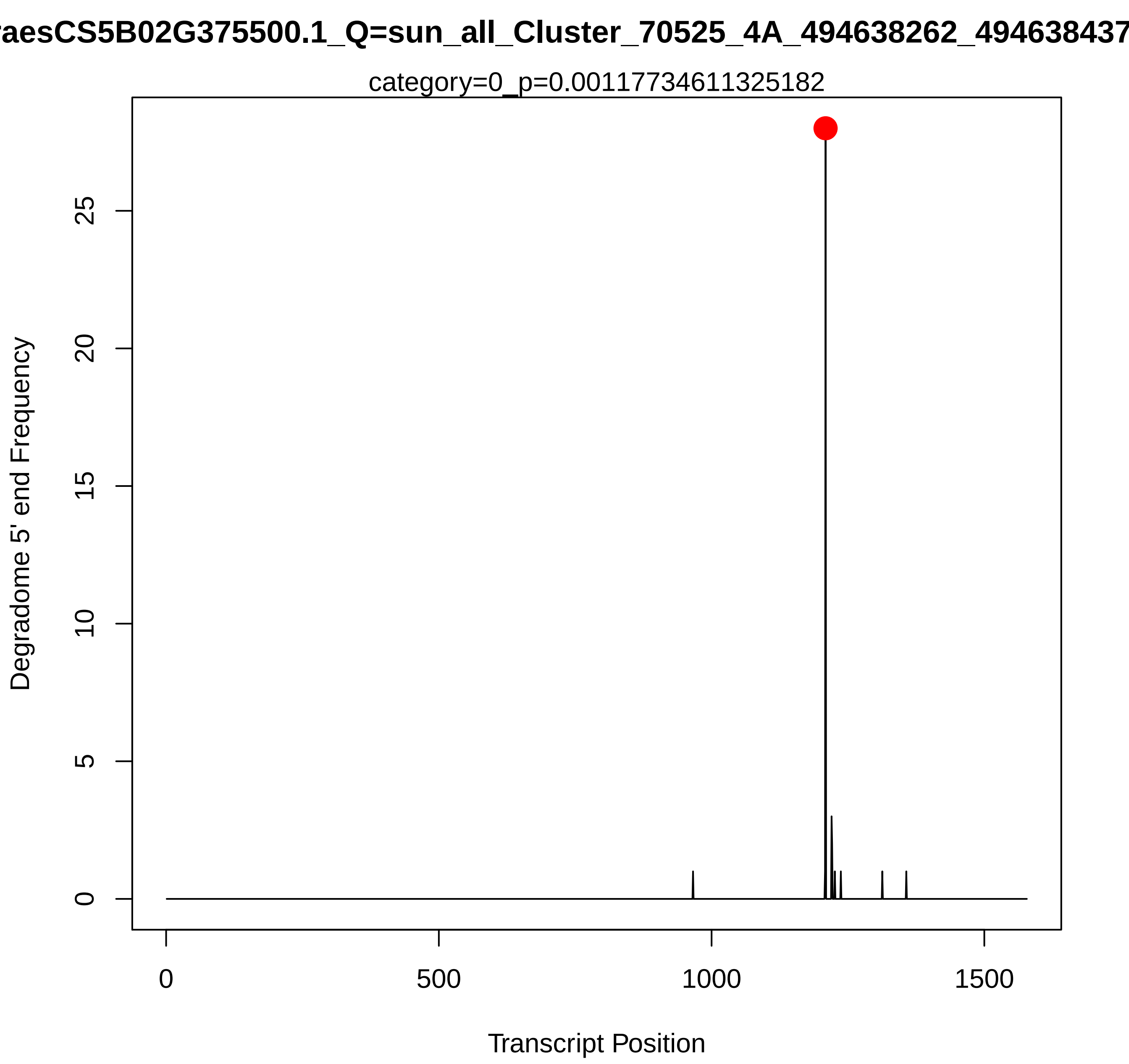

raesCS5B02G375500.1_Q=sun_all_Cluster_70525_4A_494638262_494638437
category=0_p=0.00117734611325182
Degradome 5' end Frequency
Transcript Position
0
5
10
15
20
25
0
500
1000
1500

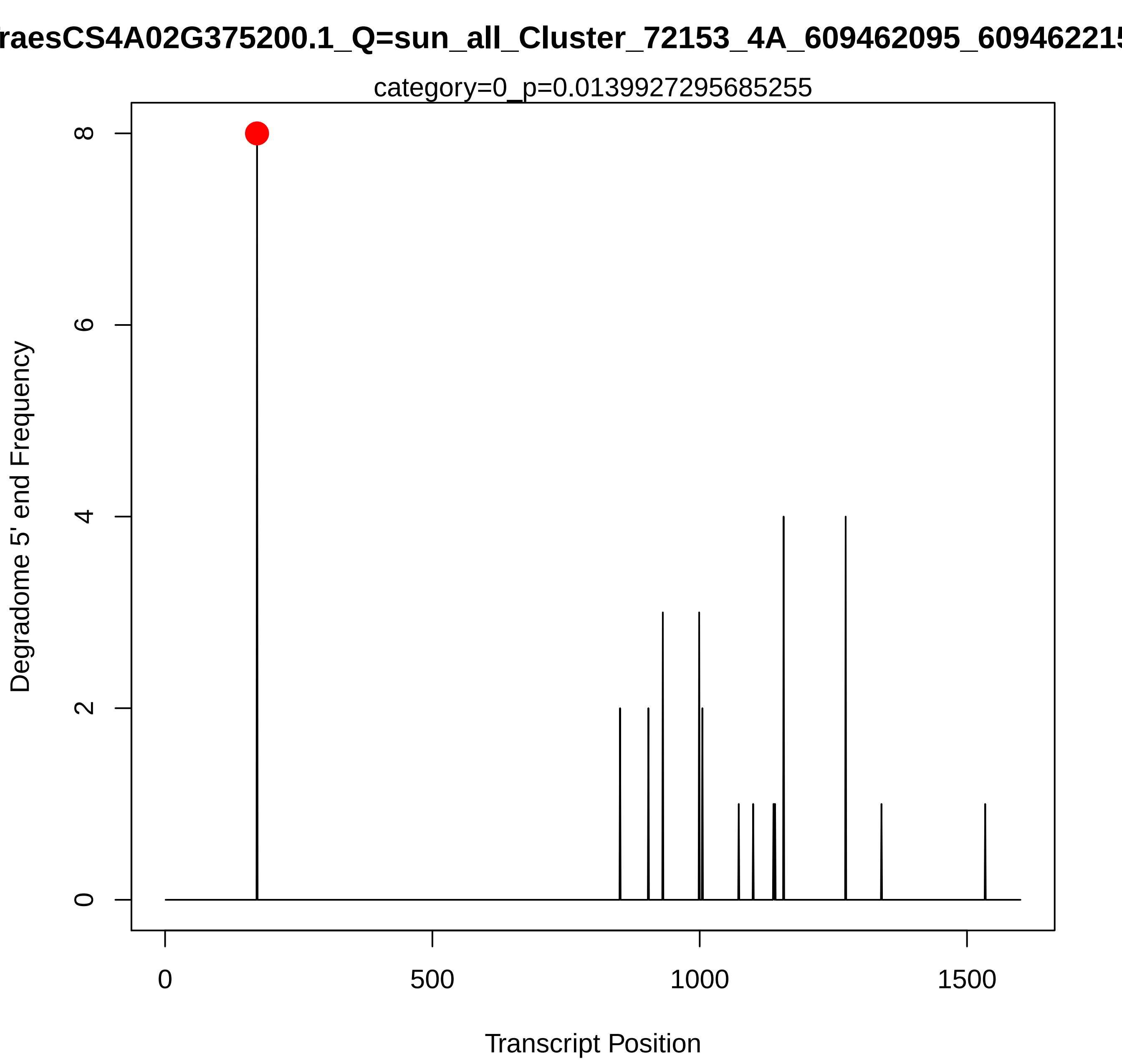
raesCS4A02G375200.1_Q=sun_all_Cluster_72153_4A_609462095_60946221
category=0_p=0.0139927295685255
Degradome 5' end Frequency
0
2
4
6
8
0
500
1000
1500
Transcript Position

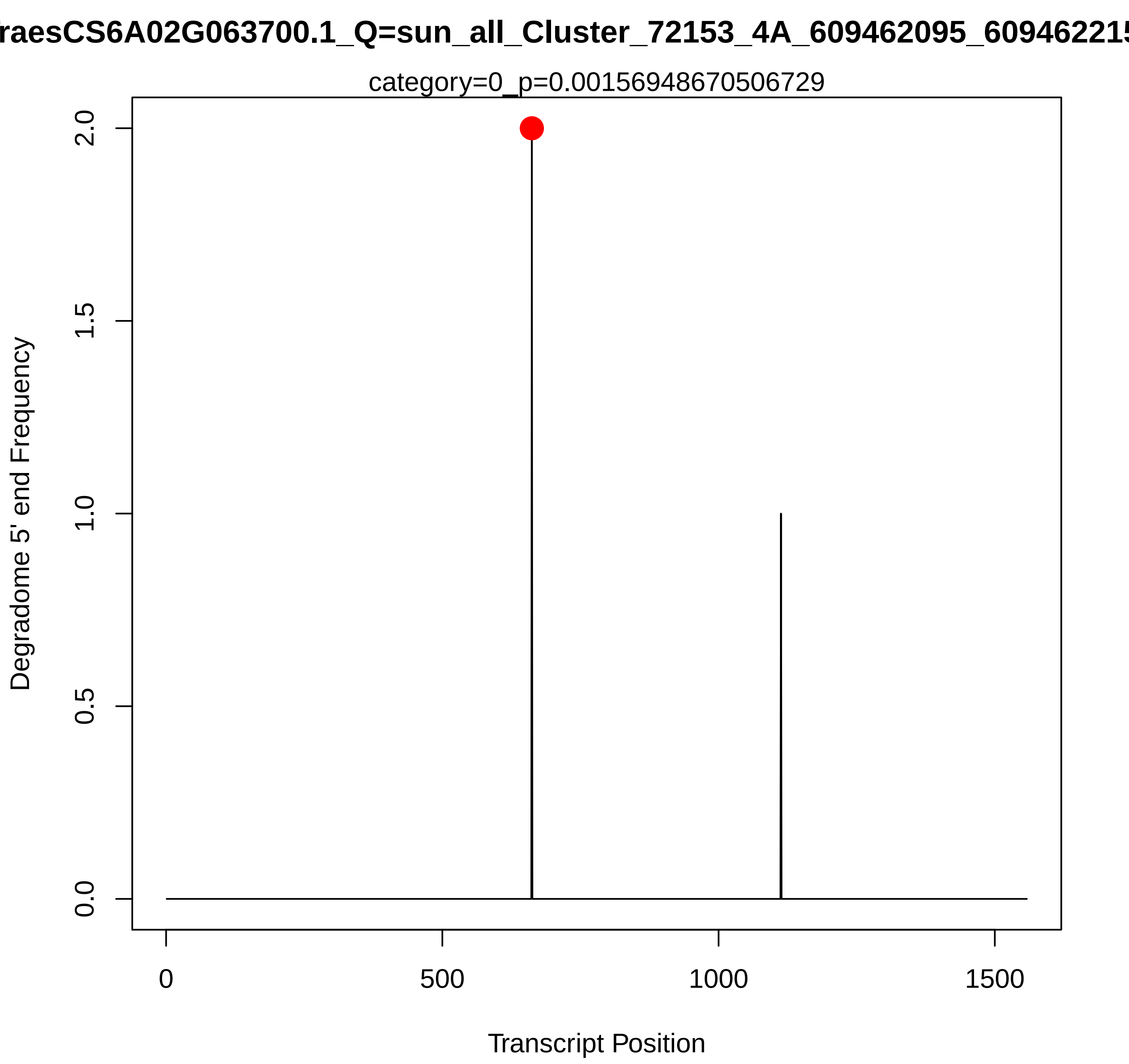
raesCS6A02G063700.1_Q=sun_all_Cluster_72153_4A_609462095_60946221
category=0_p=0.00156948670506729
Degradome 5' end Frequency
Transcript Position
0.0
0.5
1.0
1.5
2.0
0
500
1000
1500

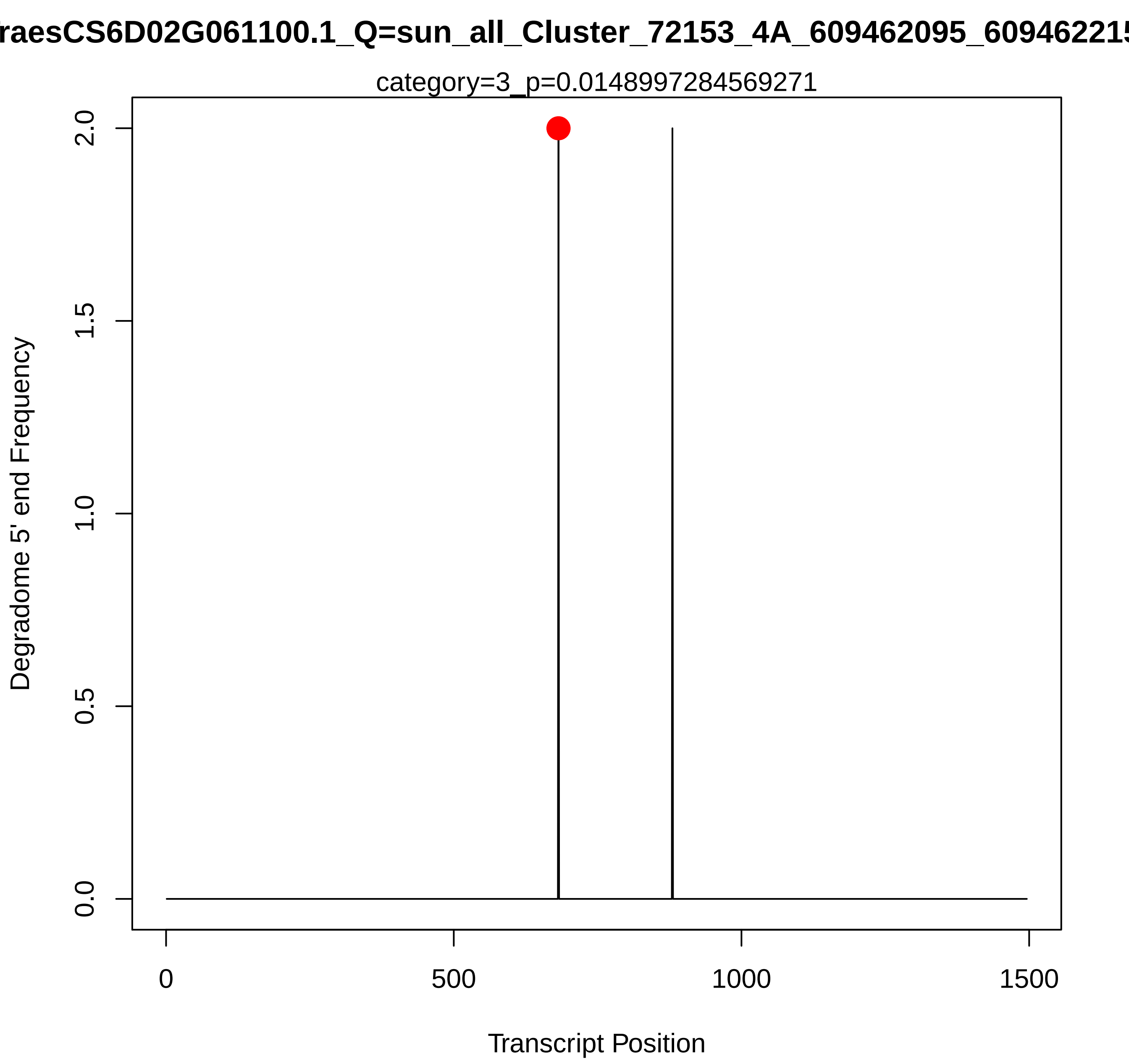
raesCS6D02G061100.1_Q=sun_all_Cluster_72153_4A_609462095_60946221
category=3_p=0.0148997284569271
Degradome 5' end Frequency
0.0
0.5
1.0
1.5
2.0
0
500
1000
1500
Transcript Position

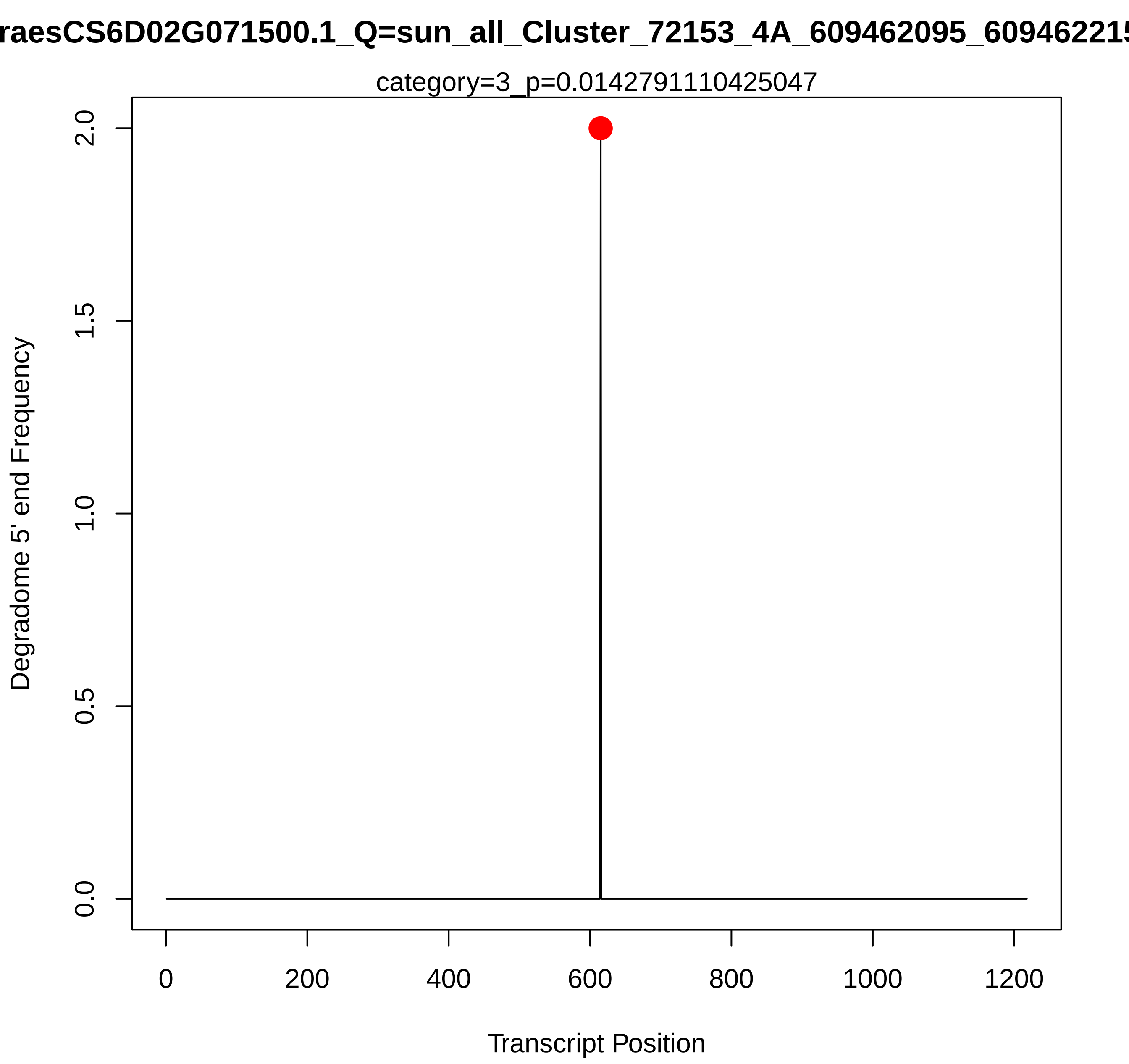

TraesCS6D02G071500.1_Q=sun_all_Cluster_72153_4A_609462095_60946221
category=3_p=0.0142791110425047
Degradome 5' end Frequency
0.0
0.5
1.0
1.5
2.0
0
200
400
600
800
1000
1200
Transcript Position

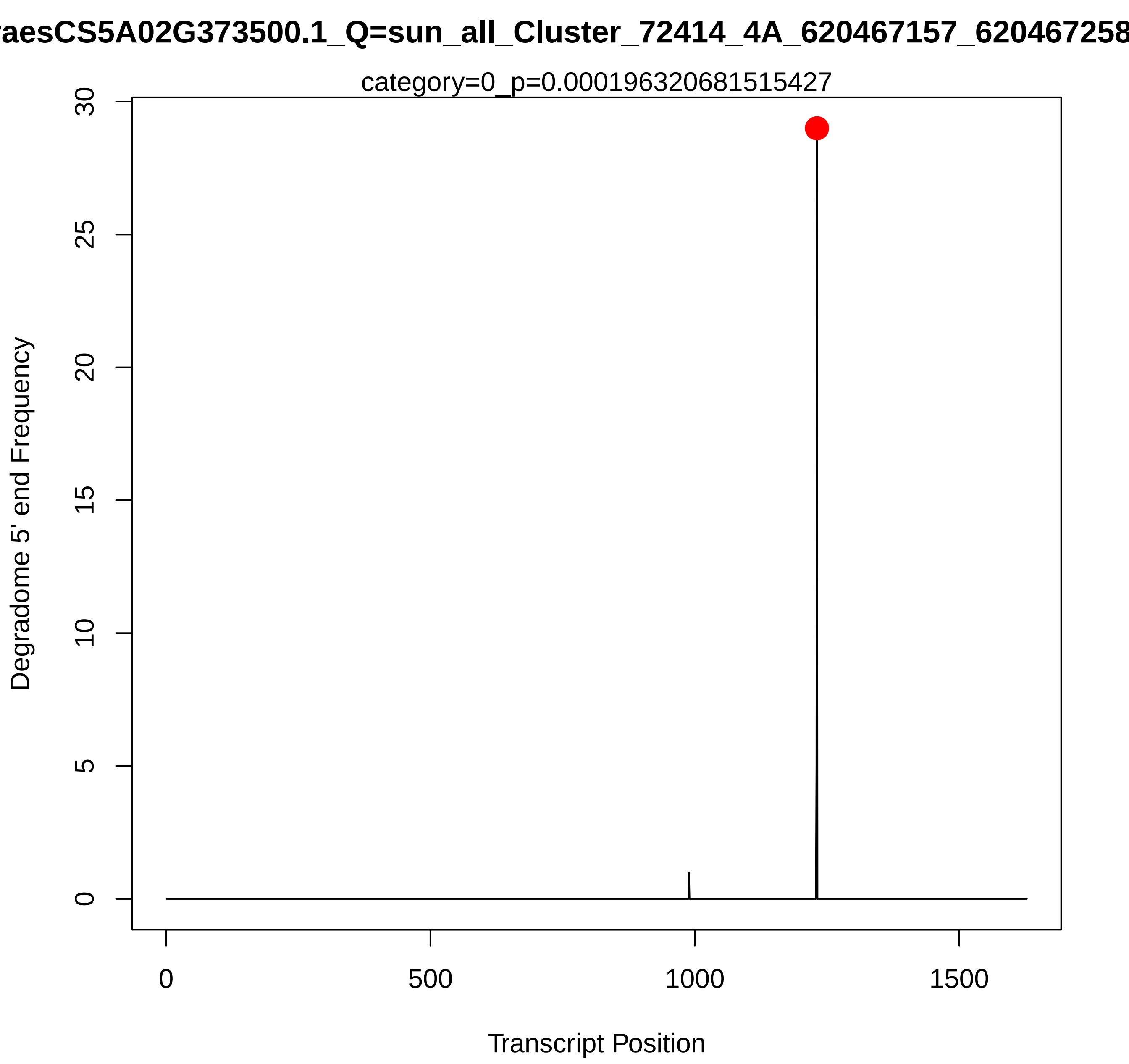

raesCS5A02G373500.1_Q=sun_all_Cluster_72414_4A_620467157_620467258
category=0_p=0.000196320681515427
Degradome 5' end Frequency
Transcript Position
0
5
10
15
20
25
30
0
500
1000
1500

aesCS5B02G028500.1_Q=sun_all_Cluster_72414_4A_620467157_620467258

category=0_p=0.000908719170493066

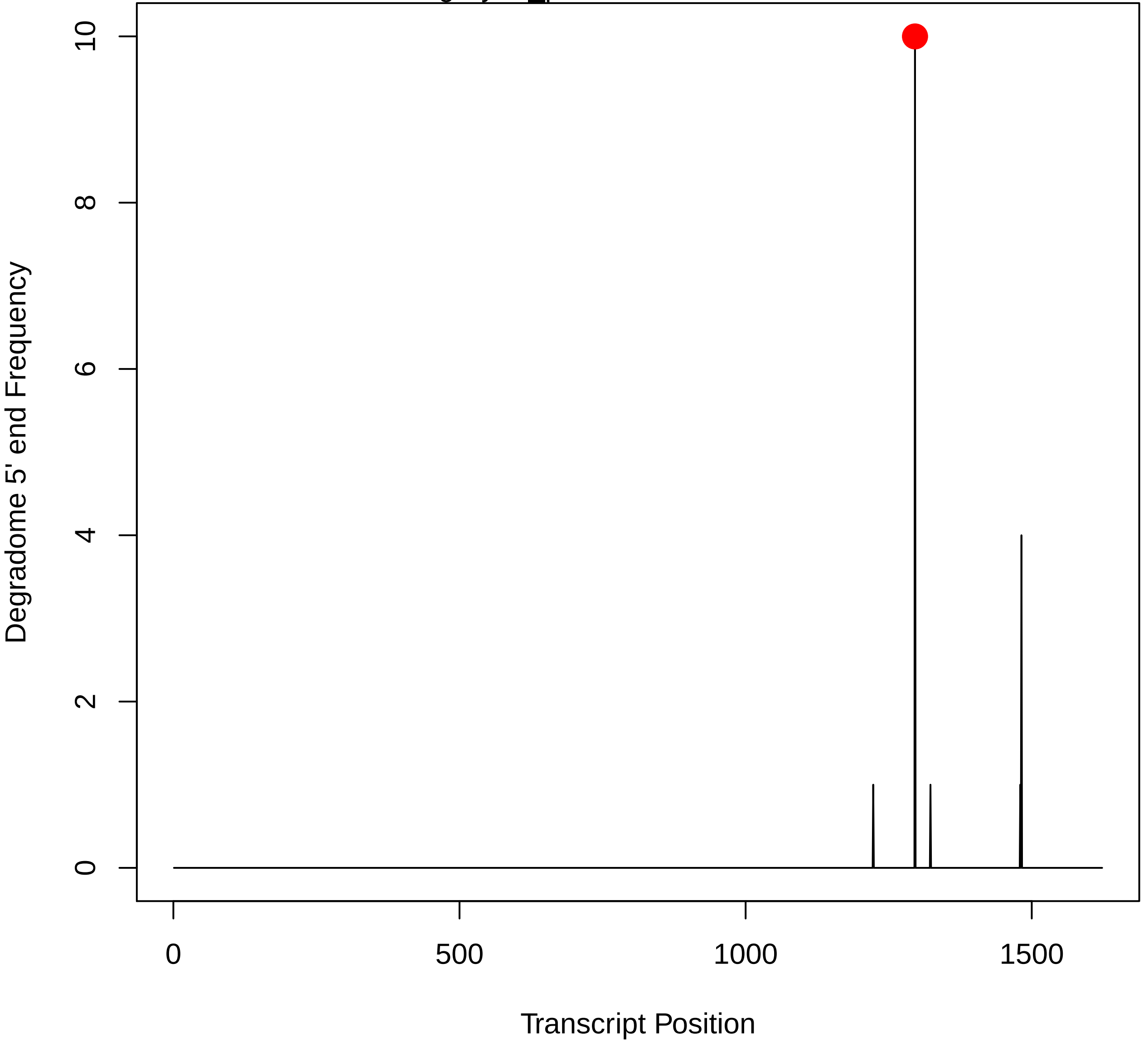

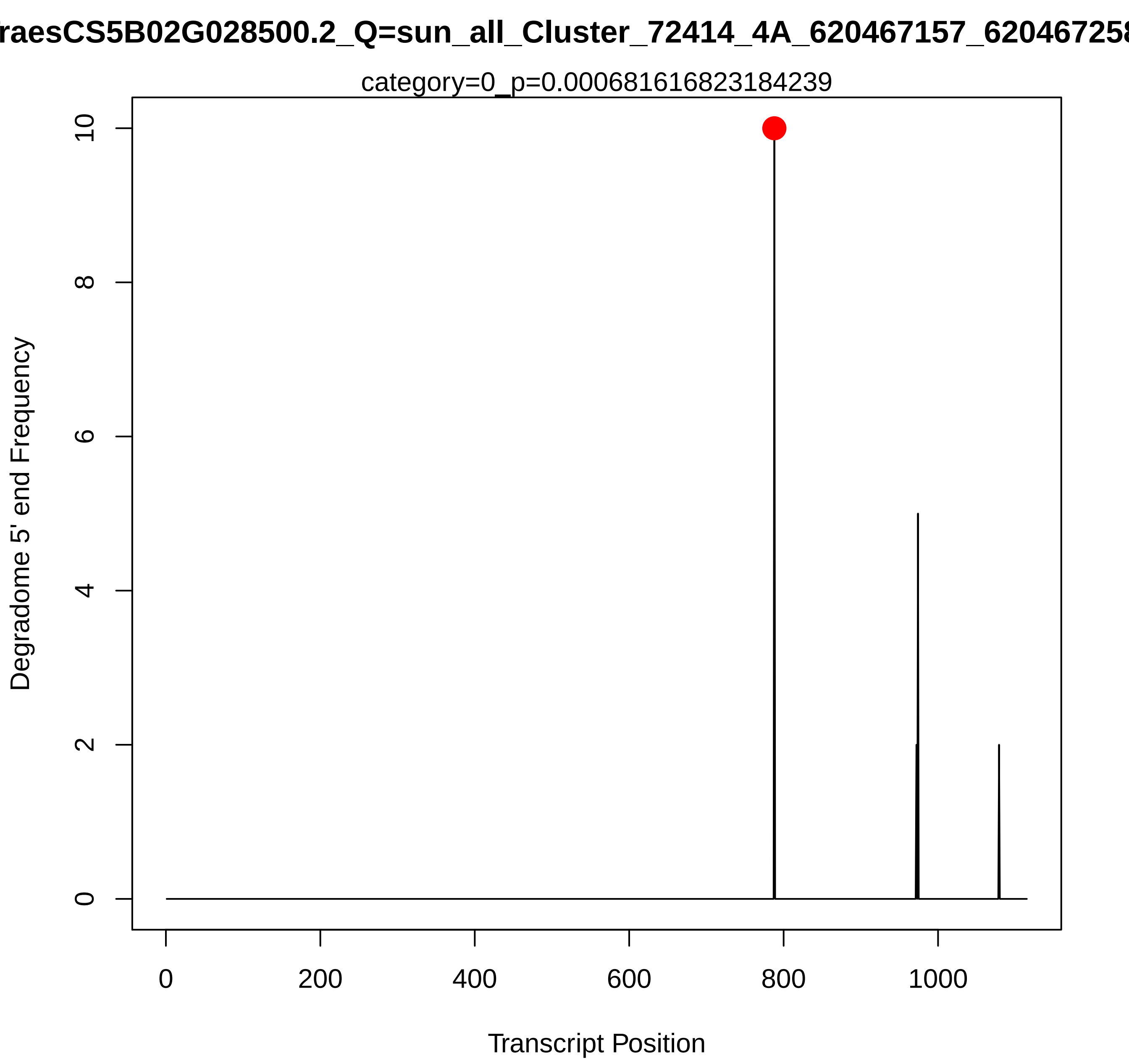

TraesCS5B02G028500.2_Q=sun_all_Cluster_72414_4A_620467157_620467258
category=0_p=0.000681616823184239
Degradome 5' end Frequency
0
2
4
6
8
10
0
200
400
600
800
1000
Transcript Position

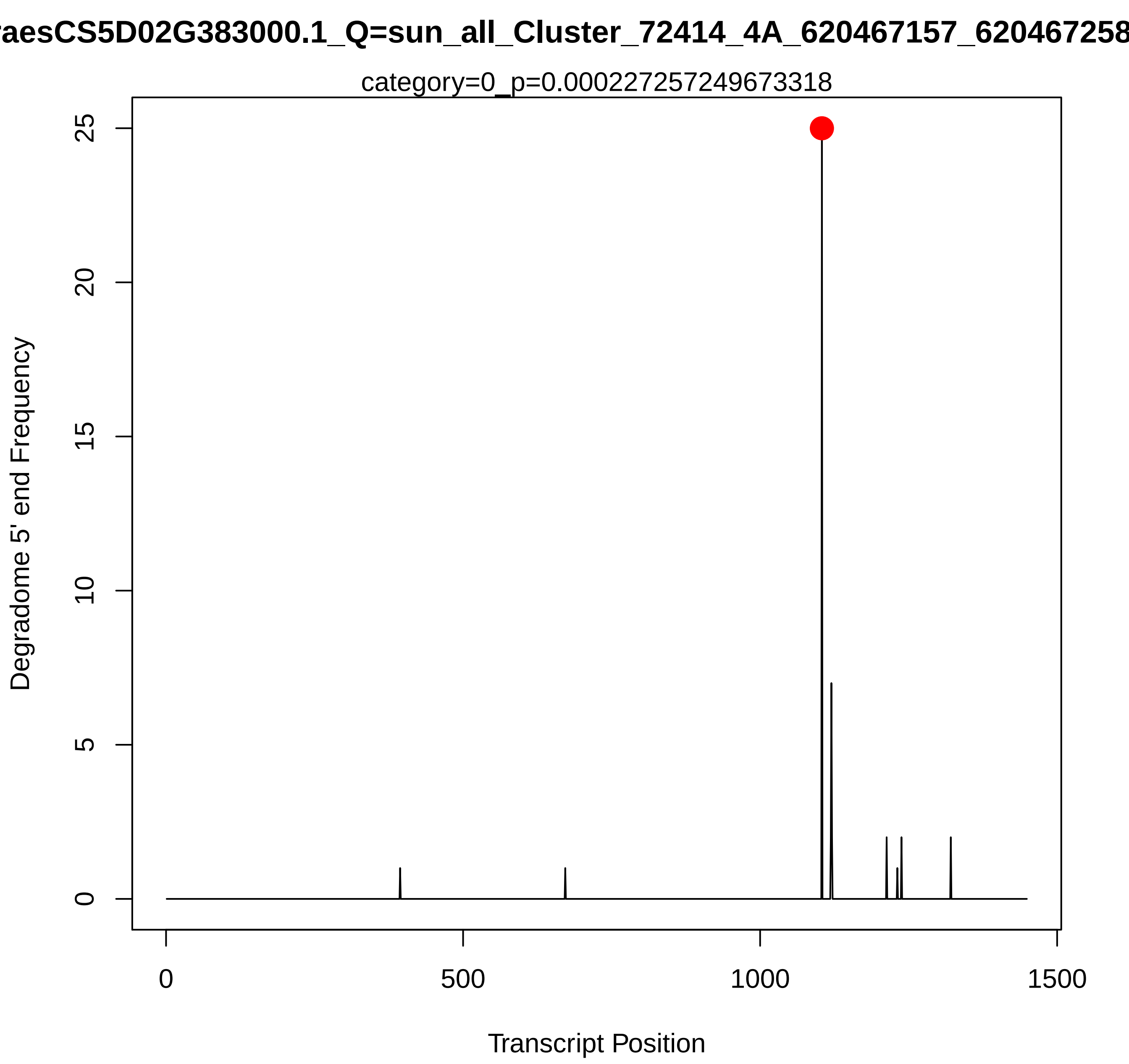

aesCS5D02G383000.1_Q=sun_all_Cluster_72414_4A_620467157_62046725
category=0_p=0.000227257249673318
Degradome 5' end Frequency
0
5
10
15
20
25
0
500
1000
1500
Transcript Position

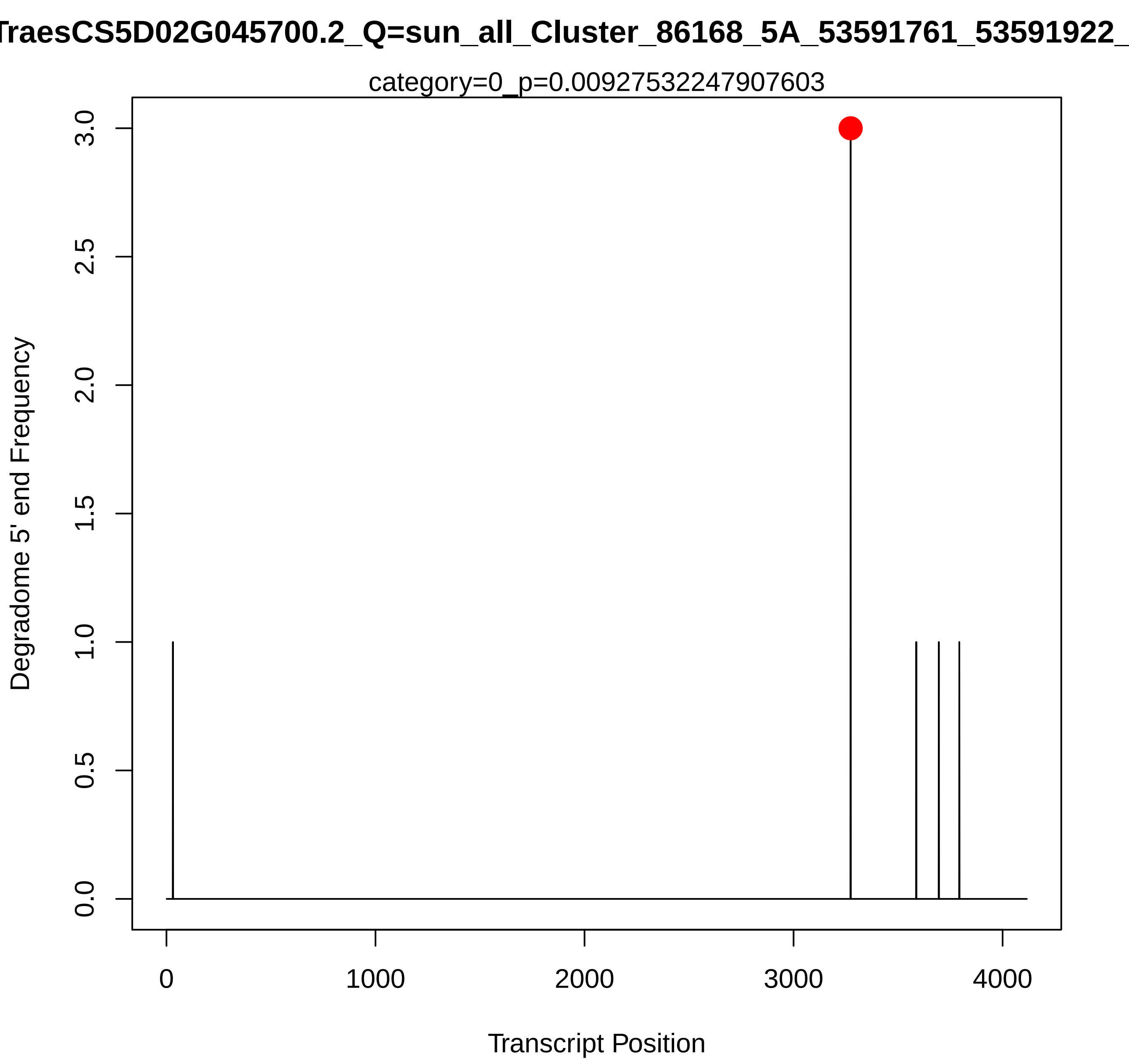
TraesCS5D02G045700.2_Q=sun_all_Cluster_86168_5A_53591761_53591922_
category=0_p=0.00927532247907603
Degradome 5' end Frequency
Transcript Position
0.0
0.5
1.0
1.5
2.0
2.5
3.0
0
1000
2000
3000
4000

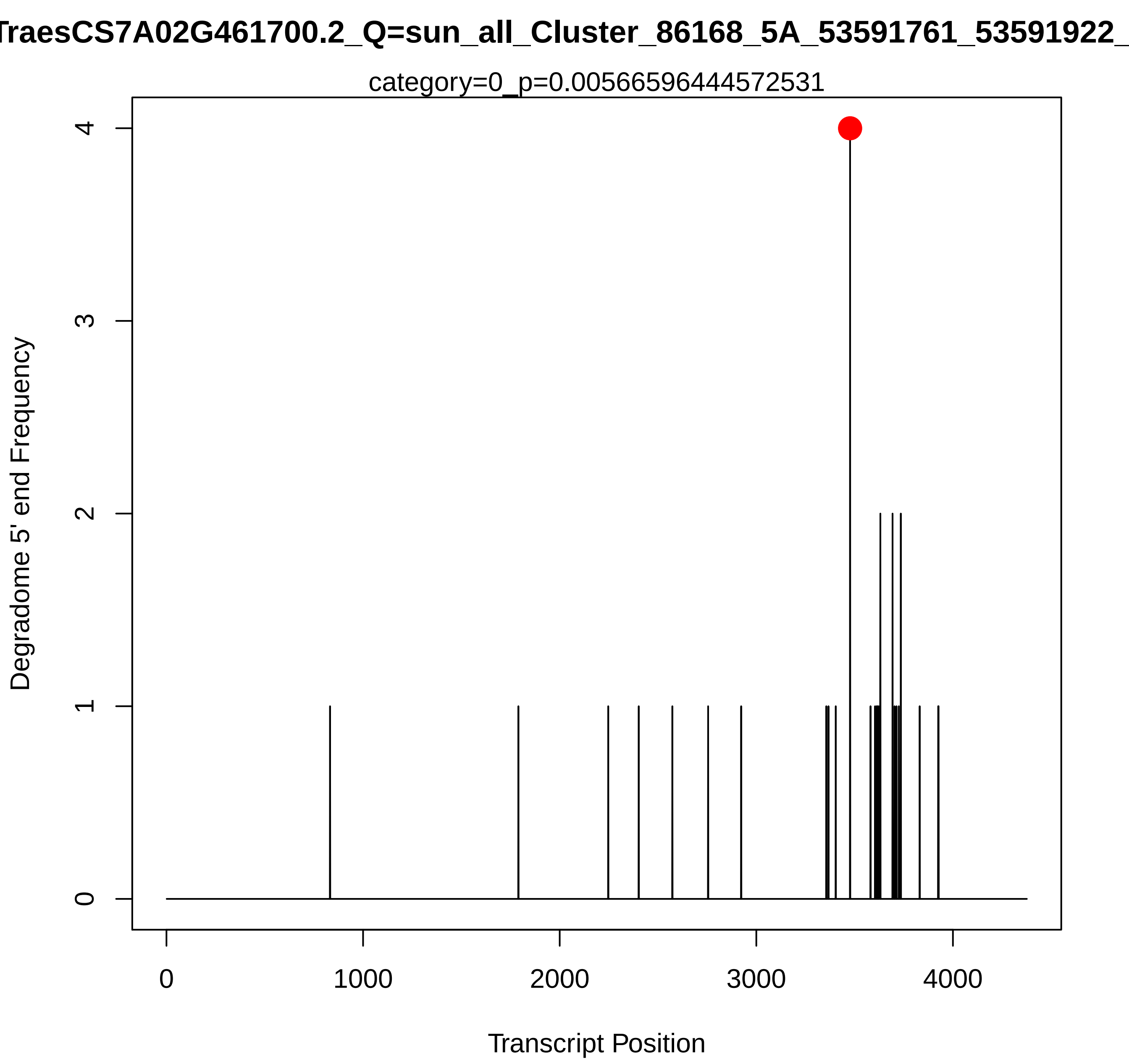
TraesCS7A02G461700.2_Q=sun_all_Cluster_86168_5A_53591761_53591922_
category=0_p=0.00566596444572531
Degradome 5' end Frequency
Transcript Position
0
1
2
3
4
0
1000
2000
3000
4000

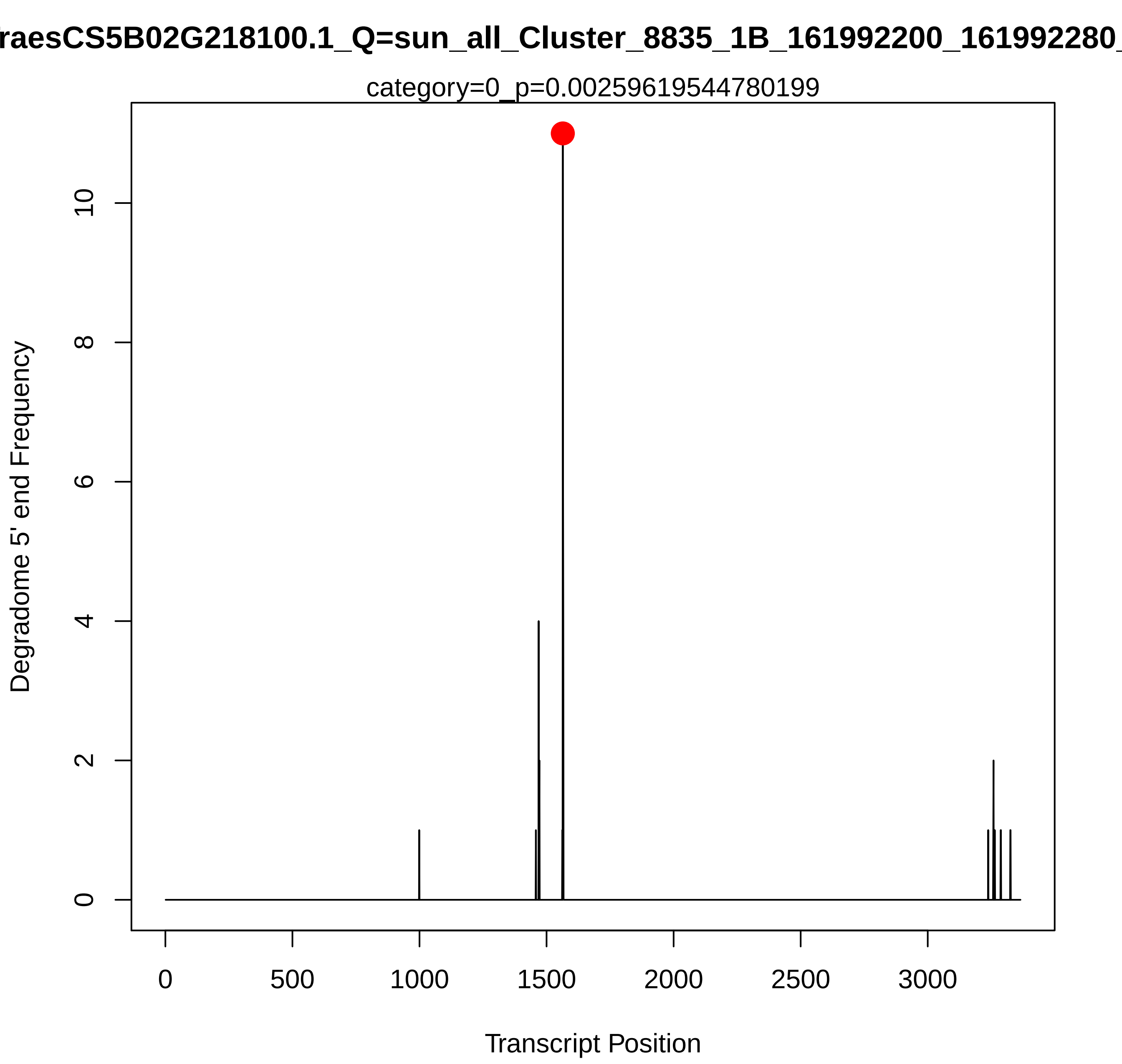

raesCS5B02G218100.1_Q=sun_all_Cluster_8835_1B_161992200_161992280_
category=0_p=0.00259619544780199
Degradome 5' end Frequency
0
2
4
6
8
10
0
500
1000
1500
2000
2500
3000
Transcript Position

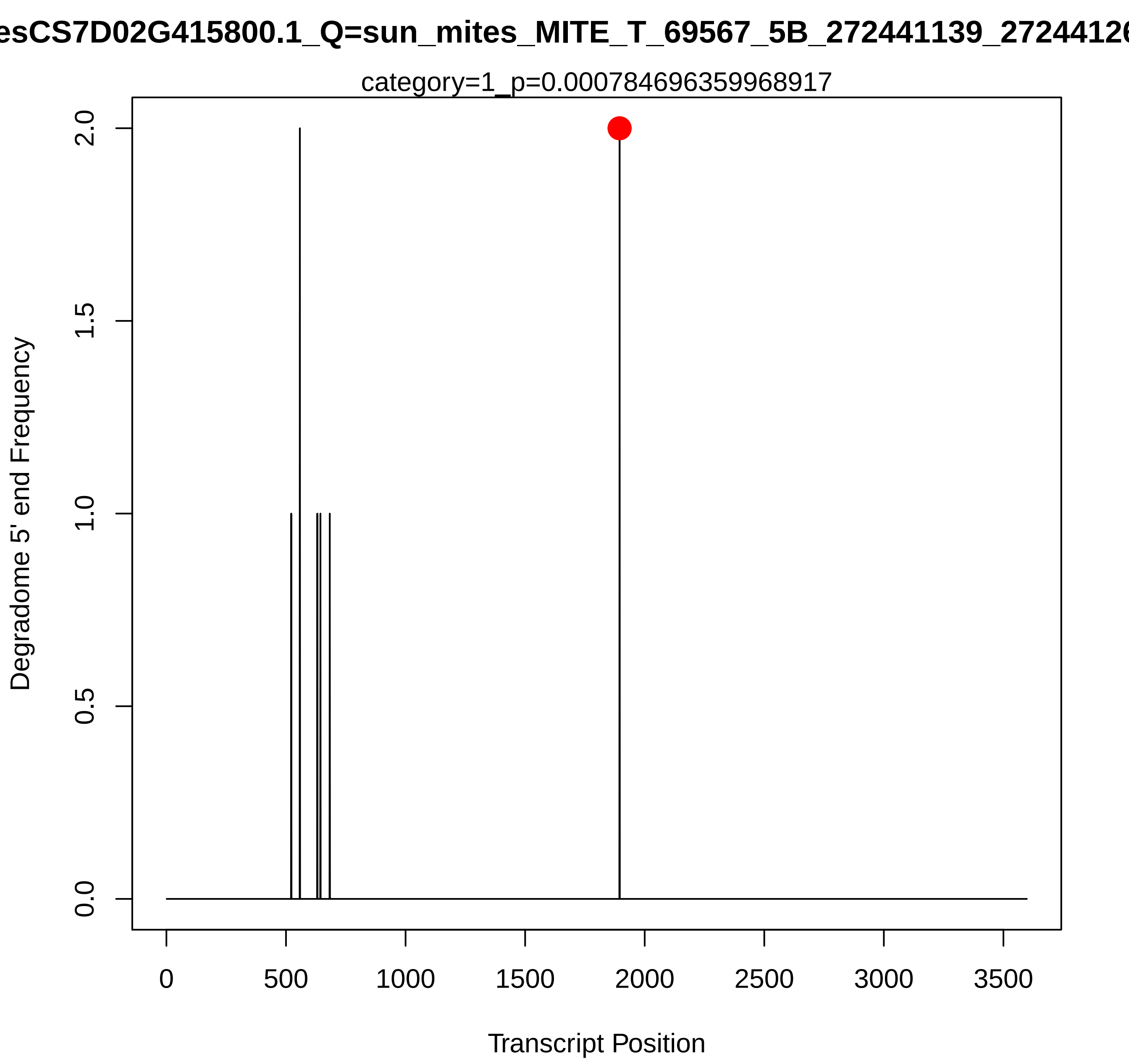
esCS7D02G415800.1_Q=sun_mites_MITE_T_69567_5B_272441139_27244126
category=1_p=0.000784696359968917
Degradome 5' end Frequency
0.0
0.5
1.0
1.5
2.0
0
500
1000
1500
2000
2500
3000
3500
Transcript Position

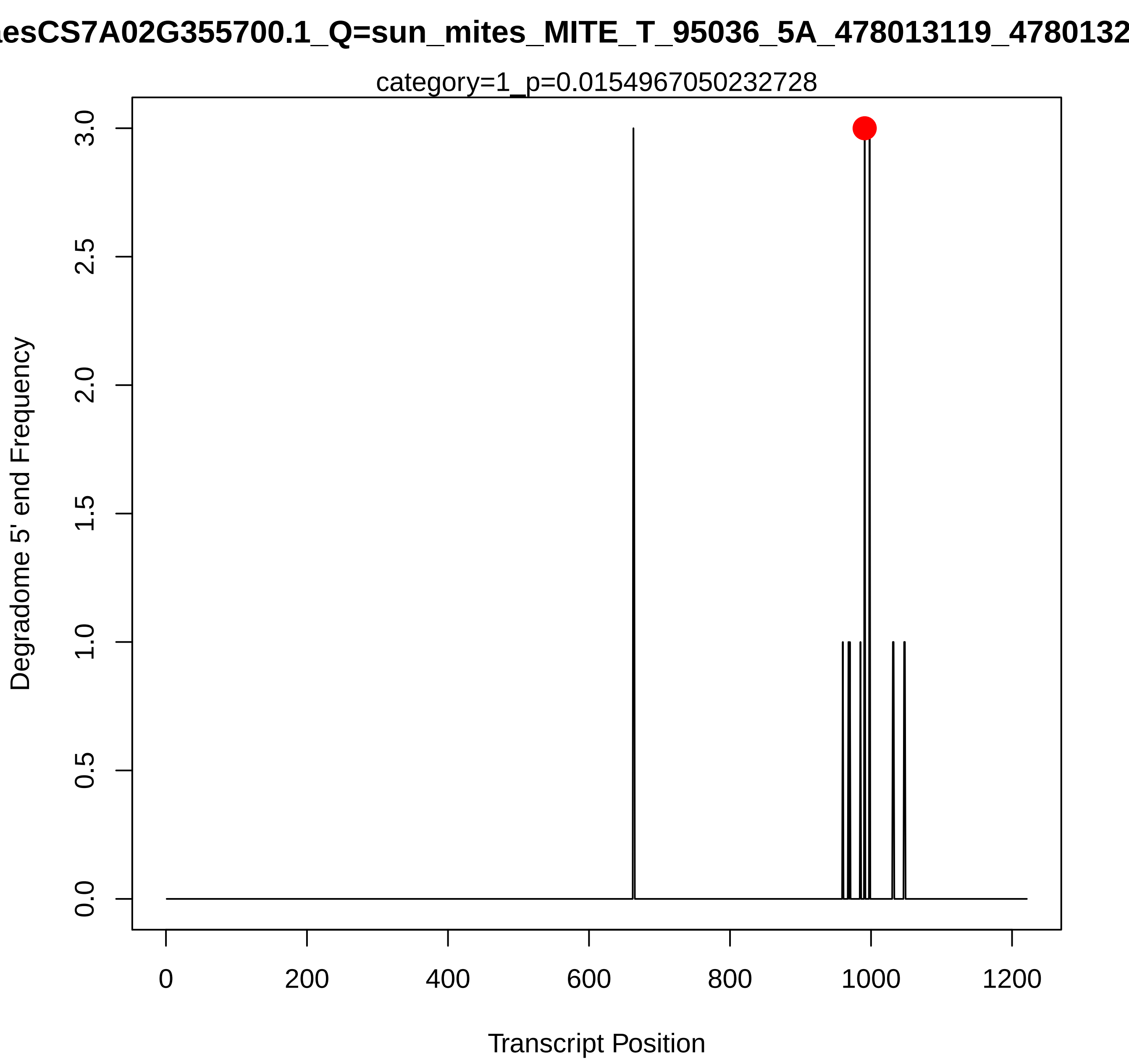
aesCS7A02G355700.1_Q=sun_mites_MITE_T_95036_5A_478013119_4780132
category=1_p=0.0154967050232728
Degradome 5' end Frequency
Transcript Position
0.0
0.5
1.0
1.5
2.0
2.5
3.0
0
200
400
600
800
1000
1200

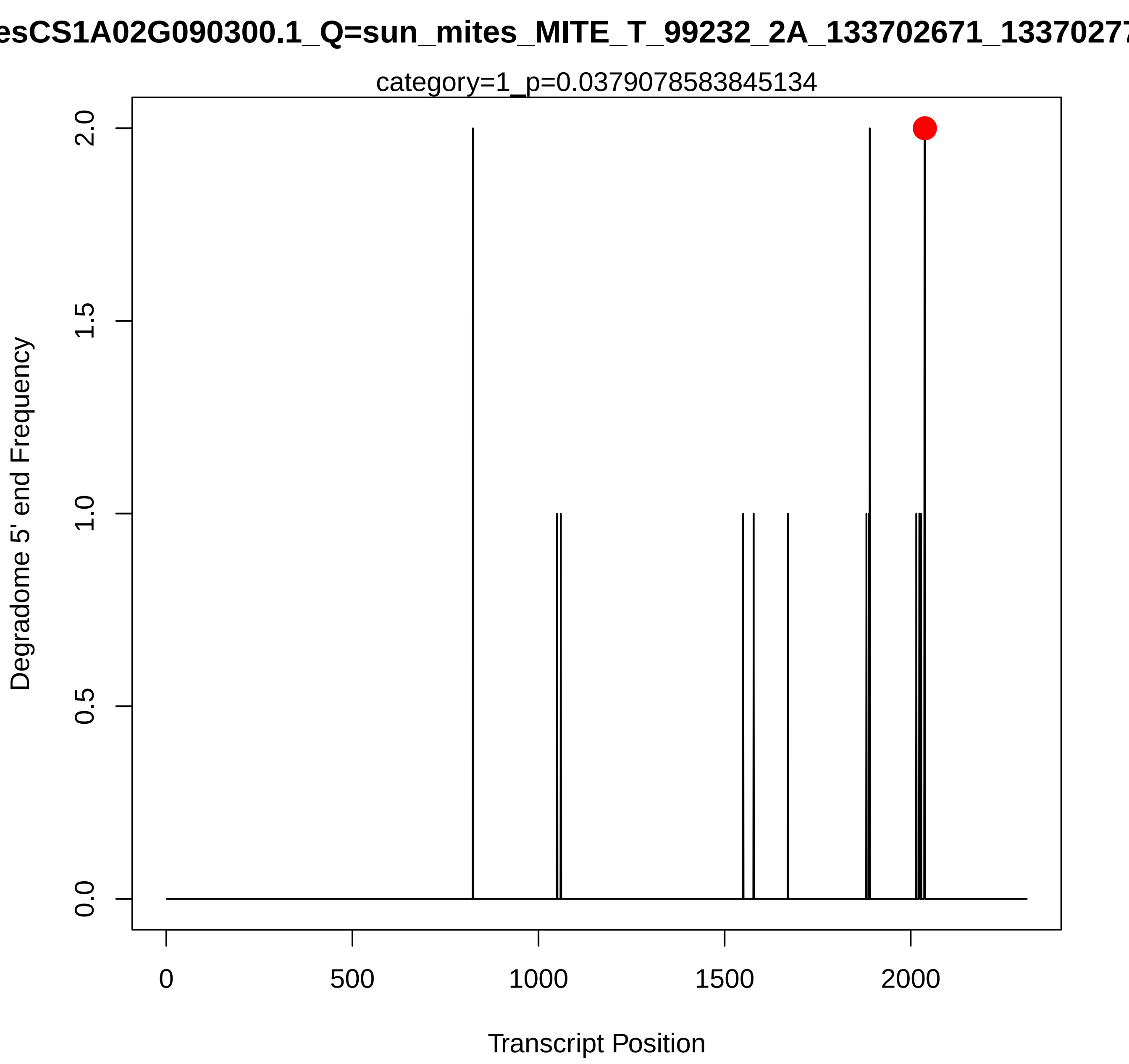
esCS1A02G090300.1_Q=sun_mites_MITE_T_99232_2A_133702671_13370277
category=1_p=0.0379078583845134
Degradome 5' end Frequency
0.0
0.5
1.0
1.5
2.0
0
500
1000
1500
2000
Transcript Position

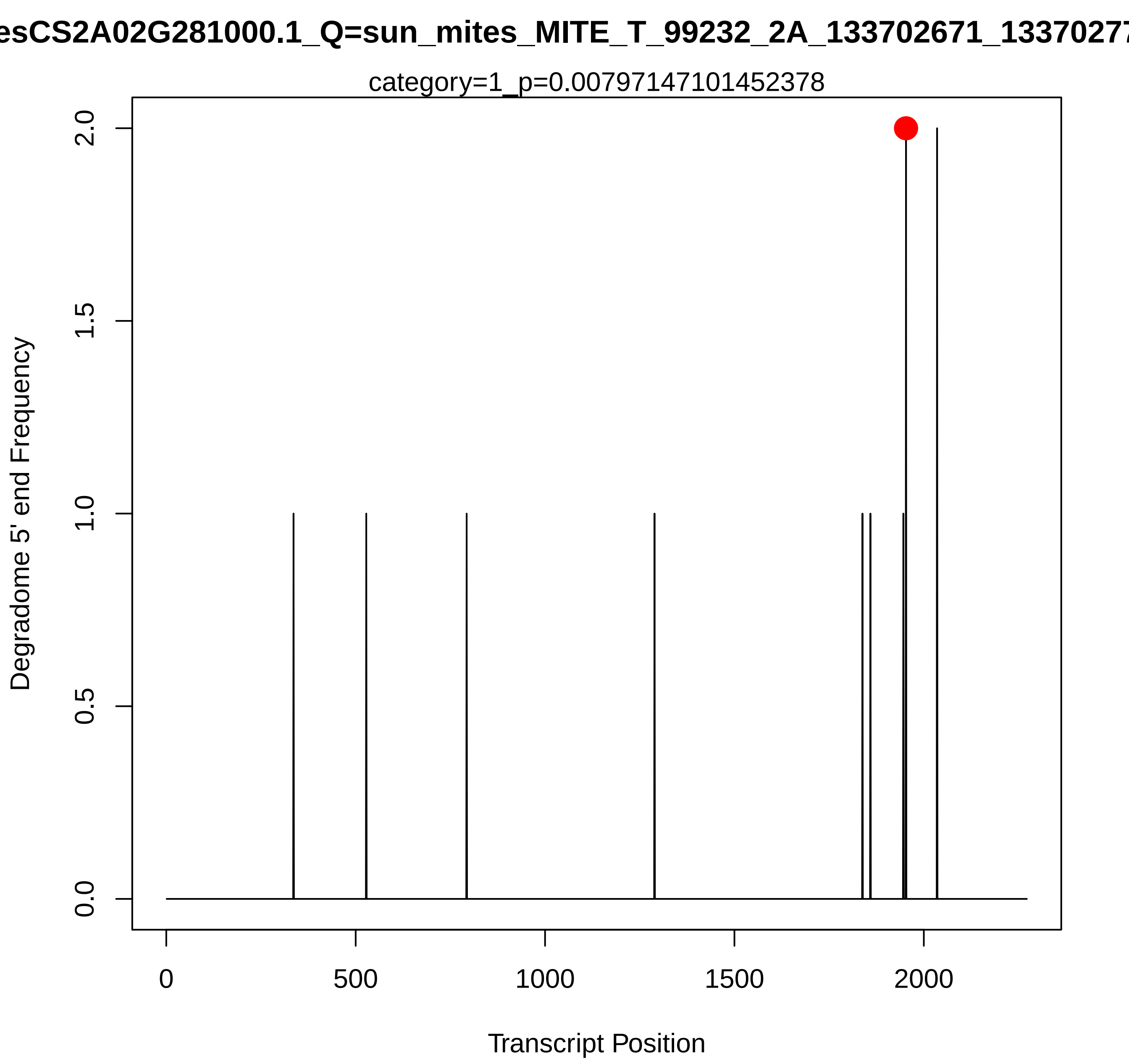

esCS2A02G281000.1_Q=sun_mites_MITE_T_99232_2A_133702671_1337027
category=1_p=0.00797147101452378
Degradome 5' end Frequency
0.0
0.5
1.0
1.5
2.0
0
500
1000
1500
2000
Transcript Position

esCS7B02G175300.2_Q=sun_mites_MITE_T_99232_2A_133702671_13370277

category=2_p=0.0449733456056327

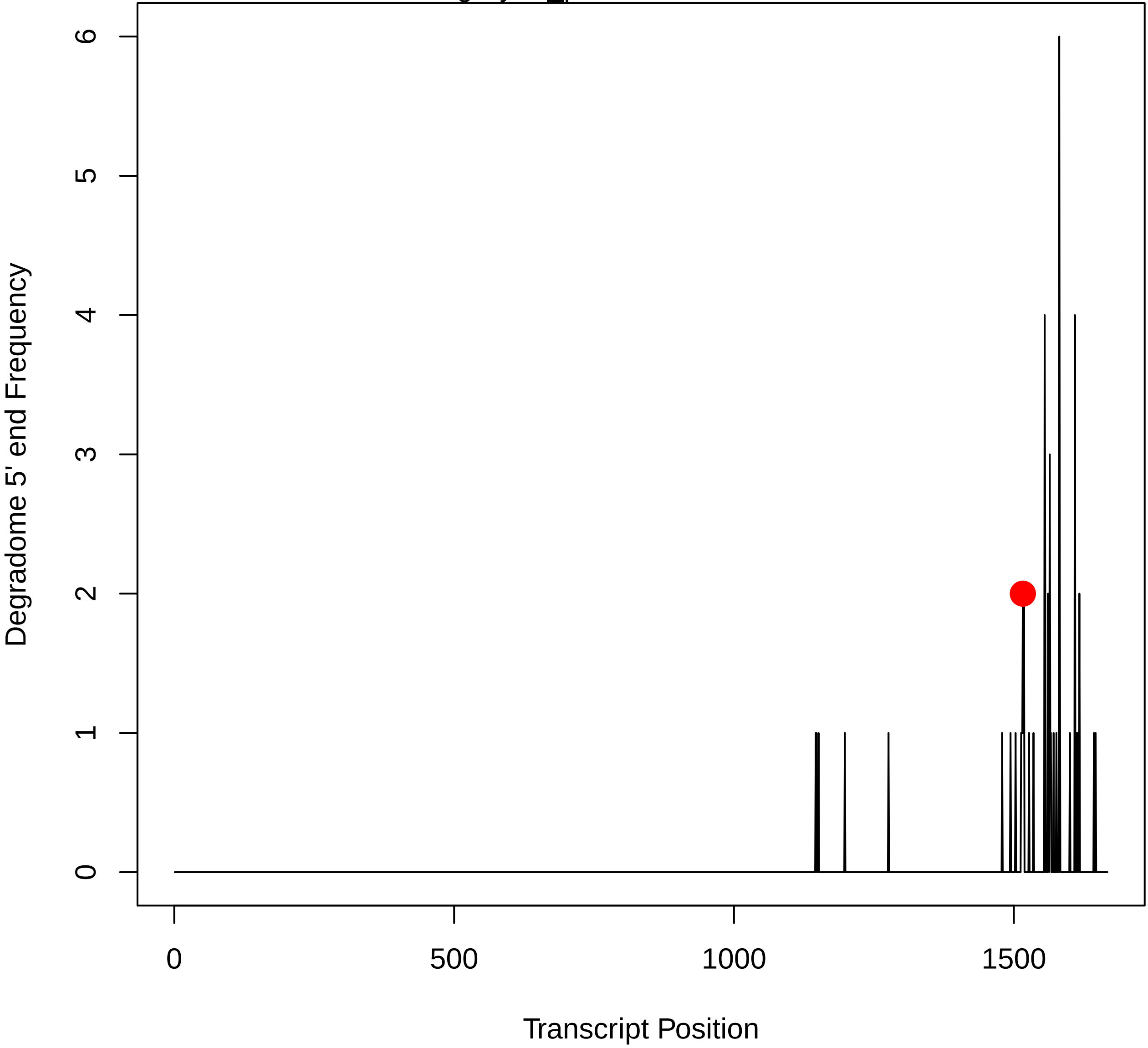

Supplement: Supplementary file 8 — Additional file 8 Degradome plots of all miRNA and targets discovered. [file 12864_2022_8364_MOESM8_ESM.pdf]
